# Disruption of ER-mitochondria contact sites by coronavirus replication organelles sustains viral replication via NSP3 stabilization

**DOI:** 10.1038/s44318-026-00816-x

**Published:** 2026-05-28

**Authors:** Xinyue Zheng, Liqiao Hu, Xubing Long, Zhilei Zhao, Rongrong Chen, Rong Bai, Buyun Tian, Maoge Zhou, Binbin Ding, Tao Xu, Zonghong Li

**Affiliations:** 1 Key Laboratory of Molecular Biophysics of the Ministry of Education, College of Life Science and Technology, Huazhong University of Science and Technology, Wuhan, China; 2 Guangzhou National Laboratory, Guangzhou, China; 3 The First Affiliated Hospital of Guangzhou Medical University, Guangzhou Medical University, Guangzhou, China; 4 National Laboratory of Biomacromolecules, CAS Center for Excellence in Biomacromolecules, Institute of Biophysics, Chinese Academy of Sciences, Beijing, China; 5 College of Life Sciences, University of Chinese Academy of Sciences, Beijing, China

**Keywords:** Membranes & Trafficking, Microbiology, Virology & Host Pathogen Interaction

## Abstract

Coronaviruses establish infection by reorganizing the host endoplasmic reticulum (ER) to form double-membrane vesicles (DMVs), which function as viral replication platforms. However, the role of other cellular organelles in this process remains incompletely understood. Here, we uncover a self-reinforcing cycle between viral replication organelles and mitochondrial damage that sustains coronavirus replication. We show that DMV formation disrupts ER-mitochondria contact sites (ERMCs), causing mitochondrial damage. This injury initiates a feed-forward mechanism wherein mitochondria release the matrix enzyme ECHS1 into the cytosol. Cytosolic ECHS1 then binds and stabilizes the DMV inducer protein NSP3 by blocking its K963 ubiquitination via the host E3 ligase RBBP6, thereby promoting further DMV formation. Disrupting this cycle, either through enhanced ER-mitochondria tethering or targeted interference with ECHS1-NSP3 binding, effectively suppresses viral replication. Our findings reveal that coronaviruses exploit an inter-organellar feedback loop linking mitochondrial damage to DMV formation, identifying new potential therapeutic targets for inhibition of coronaviral replication.

## Synopsis

Coronaviruses reorganize intracellular membranes into double-membrane vesicles (DMVs) inside which they replicate. This study identifies an interplay between these viral replication organelles and damage to mitochondria that sustains efficient coronavirus replication.

- DMV formation impairs mitochondrial function by disrupting ER-mitochondria contact sites (ERMCs).
- Enforcing ER-mitochondria tethering suppresses coronavirus replication.
- Cytosolic release of the mitochondrial matrix protein ECHS1 promotes DMV formation and coronavirus infection.
- ECHS1 stabilizes NSP3 by blocking its ubiquitination by the host E3 ligase RBBP6.

## Abstract (web summary)

Coronavirus infection-induced mitochondrial damage promotes viral replication by inhibiting ubiquitination-dependent degradation of NSP3.

## Graphical abstract

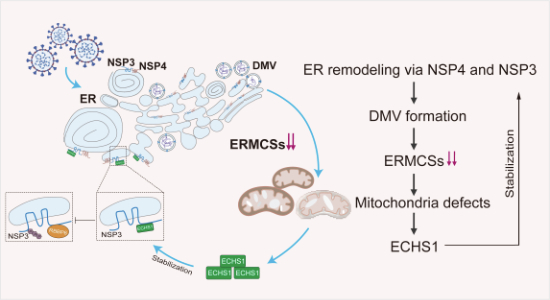

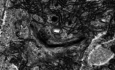

## Introduction

Coronaviruses (CoVs) are enveloped, positive-sense single-stranded RNA viruses that pose substantial threats to global health, as starkly exemplified by the severe acute respiratory syndrome coronavirus 2 (SARS-CoV-2) pandemic (Perlman and Netland, 2009; Su et al, 2016; V’kovski et al, 2021). A critical step in the CoV life cycle involves the extensive remodeling of host membranes into specialized cytoplasmic compartments termed double-membrane vesicles (DMVs). These structures provide a dedicated platform for viral RNA synthesis while simultaneously shielding viral components from cytosolic innate immune sensors (Gao et al, 2025; Knoops et al, 2008; Klein et al, 2020; Cortese et al, 2020). The assembly of these replication organelles is primarily driven by two viral nonstructural proteins: NSP3 and NSP4. NSP3, a multifunctional protein and core component of the DMV pore complex, interacts with NSP4 to promote membrane pairing, pore formation, and anchoring of the replication machinery. Notably, ectopic co-expression of NSP3 and NSP4 alone is sufficient to induce DMVs that structurally resemble those observed in infected cells (Zimmermann et al, 2023; Huang et al, 2024; Yang et al, 2025). Multiple studies have established that DMVs originate from rearranged ER membranes and have identified several ER-associated host factors, such as TMEM41B, VMP1, RTN3/4, REEP5, GRAMD1B, and TEX2, as essential for DMV biogenesis (Ji et al, 2022; Williams et al, 2023; Li et al, 2023a; Zhou et al, 2025). While this ER-centric model of DMV biogenesis is well established, whether and how other cellular organelles contribute to this process remains a fundamental open question.

Mitochondria, which function as central hubs for cellular metabolism and innate immunity, maintain dynamic communication with the ER via specialized membrane contact sites (ERMCSs). These junctions are critical for lipid exchange, calcium signaling, and the regulation of mitochondrial dynamics and homeostasis. Given these essential roles, it is unsurprising that numerous viruses target mitochondrial function to evade host immune defenses. Previous studies have demonstrated that coronavirus infection induces mitochondrial dysfunction, which is thought to represent a central pathological mechanism in COVID-19, contributing to systemic inflammation, multi-organ failure, and long-term sequelae (Foo et al, 2022; Che et al, 2025). Specifically, the viral protein ORF9b localizes to mitochondria-associated membranes (MAMs), driving mitochondrial fragmentation and suppressing innate immune signaling (Lenhard et al, 2023). Additionally, ORF3a, the membrane (M) protein, and the nucleocapsid (N) protein have been shown to disrupt mitochondrial architecture and function, thereby facilitating viral replication (López-Ayllón et al, 2024; Ramachandran et al, 2022; Li et al, 2024). However, these findings have largely focused on the direct effects of individual viral proteins. The potential impact of the massive, virus-driven reorganization of the ER into DMVs on mitochondrial integrity, and conversely, the role of mitochondrial fidelity in regulating the viral replication organelle landscape, remain unclear.

Here, we uncover a previously unrecognized feedback loop that couples DMV biogenesis with mitochondrial dysfunction. Specifically, DMV assembly disrupts mitochondrial function by disrupting ERMCSs, while damaged mitochondria release the matrix enzyme ECHS1, which accumulates in the cytosol and then stabilizes NSP3 by blocking polyubiquitination of lysine 963 (K963) mediated by the host E3 ligase RBBP6. This stabilization enhances NSP3 activity to promote further DMV formation, thereby establishing a self-reinforcing cycle wherein DMVs impair mitochondrial function and mitochondrial damage reinforces DMV biogenesis. This reciprocal potentiation is essential for efficient coronavirus replication.

## Results

### DMV formation impairs mitochondrial morphology and function

**Figure 1:**
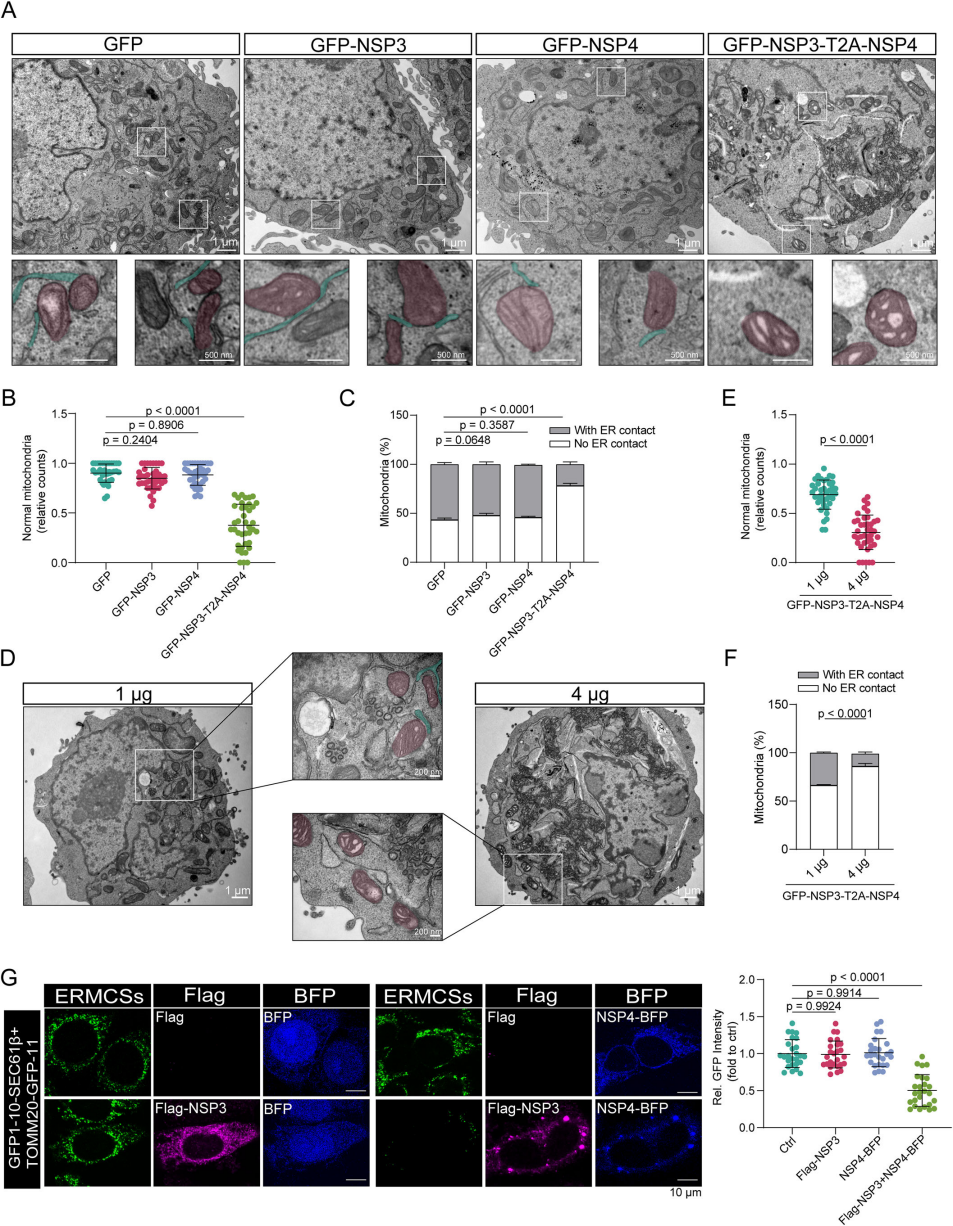
DMV formation impairs mitochondrial morphology and function. (**A**) Representative TEM images of mitochondrial morphology in HeLa cells following transfection with 3 μg of GFP-vector control, GFP-NSP3, GFP-NSP4, or GFP-NSP3-T2A-NSP4 overexpression plasmids. Scale bars, 1 μm (main images). Zoomed-in views highlight direct contacts between the ER and mitochondria. Scale bars, 500 nm (zoomed-in views). (**B**) Quantification of the proportion of normal mitochondria relative to total mitochondria from TEM images in (**A**). This analysis was performed using three independent experimental replicates. A total of > 35 cells were counted per group, pooling data from the three replicates. Data are presented as mean ± SD; *P* values were determined by one-way ANOVA. (**C**) Quantification of the proportion of mitochondria with or without ER contact from TEM images in (**A**). Data are presented as mean ± SD, with *n* > 20 cells per group in each of the three independent experimental replicates; *P* values were calculated by one-way ANOVA based on the proportion of mitochondria with ERMCSs. (**D**) Representative TEM images of mitochondria morphology in HeLa cells following transfection with 1 μg or 4 μg of the GFP-NSP3-T2A-NSP4 overexpression plasmid. Scale bars, 1 μm (main images). Zoomed-in views show mitochondrial structural changes. Scale bars, 200 nm (zoomed-in views). (**E**) Quantification of the proportion of normal mitochondria relative to total mitochondria from TEM images in (**D**). This analysis was performed using three independent experimental replicates. A total of >35 cells were counted per group, pooling data from the three replicates. Data are presented as mean ± SD; *P* values were determined by unpaired *t* test. (**F**) Quantification of the proportion of mitochondria with or without ER contact from TEM images in (**D**). Data are presented as mean ± SD, with *n* = 10 cells per group in each of the three independent experimental replicates; *P* value was calculated by unpaired *t* test based on the proportion of mitochondria with ERMCSs. (**G**) Representative immunofluorescence images of the ERMCSs reporter in HeLa cells co-expressing Flag-vector and BFP-vector (Ctrl), Flag-NSP3 and BFP-vector, Flag-vector and NSP4-BFP, or Flag-NSP3 and NSP4-BFP. The ERMCSs reporter is based on a split-GFP complementation assay, where co-expression of GFP1-10-SEC61β (ER marker) and TOMM20-GFP11 (mitochondrial marker) leads to fluorescent signal reconstitution specifically at ER-mitochondria interfaces. Quantification of GFP fluorescence intensity (right panel). Data are presented as mean ± SD, with *n* > 20 cells per group; *P* values were calculated by one-way ANOVA. Scale bars, 10 μm. Source data are available online for this figure.

To investigate the crosstalk between DMV formation and other cellular organelles, we examined cells co-expressing NSP3 and NSP4 using transmission electron microscopy (TEM). Strikingly, mitochondria in cells co-expressing NSP3 and NSP4 exhibited significant morphological abnormalities compared to control cells, including mitochondrial matrix vacuolization, blurred outer membrane boundaries, and collapsed cristae (Fig. 1A). Notably, these defects were absent in cells expressing either NSP3 or NSP4 individually, which indicates that DMV biogenesis, rather than expression of either protein alone, is the primary trigger for mitochondrial damage. Quantitative morphological analysis revealed that approximately 60% of mitochondria exhibited abnormalities during DMV formation (Fig. 1B). Consistent with our previous report that DMV abundance increases with increasing NSP3 and NSP4 expression levels (Yang et al, 2025), we further found that higher NSP3 and NSP4 expression levels cause more severe mitochondrial abnormalities, confirming that dose-dependent DMV accumulation is associated with progressive mitochondrial damage (Fig. 1D,E).

**Figure EV1:**
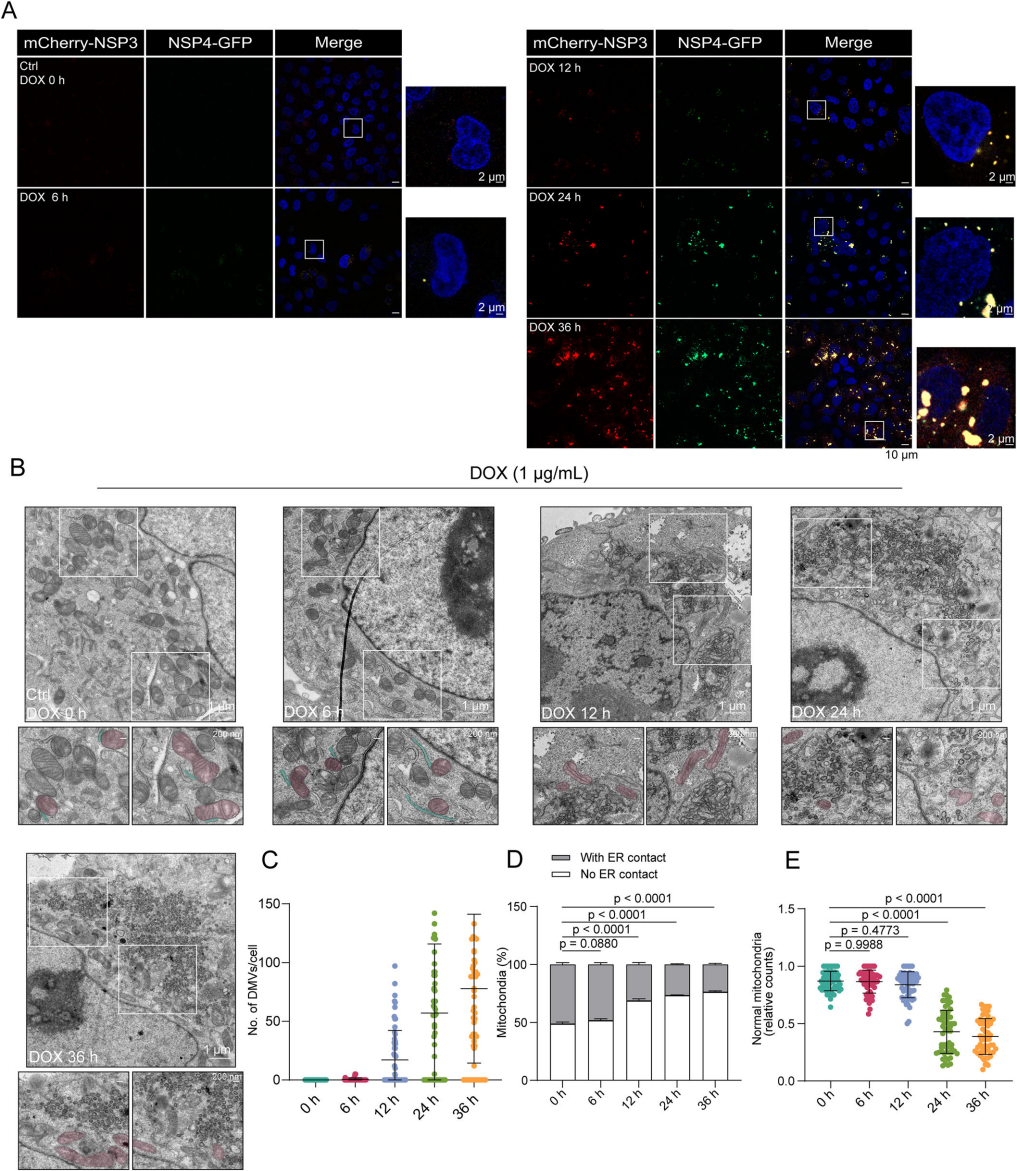
Time-course analysis of DMV biogenesis, mitochondrial morphological abnormalities, and ERMCS dynamics in DOX-induced NSP3/4 expression system. (**A**) Representative immunofluorescence images of stable HeLa cell lines with DOX (1 μg/mL)-inducible expression of mCherry-NSP3 and NSP4-GFP, at 0, 6, 12, 24, and 36 h post-DOX induction. Zoomed-in views highlight the puncta accumulation of mCherry-NSP3 and NSP4-GFP over time. Scale bars, 10 μm (main images); 2 μm (zoomed-in views). (**B**) Representative TEM images of mitochondrial morphology and DMV formation in these DOX (1 μg/mL)-inducible stable HeLa cell lines at 0, 6, 12, 24, and 36 h post-DOX induction. Zoomed-in views show mitochondrial structural changes during DMV biogenesis. Scale bars, 1 μm (main images); 200 nm (zoomed-in views). (**C**) Quantification of DMV numbers per cell from TEM images in (**B**). (**D**) Quantification of the proportion of mitochondria with or without ER contact from TEM images in (**B**). Data are presented as mean ± SD, with *n* > 15 cells per time point in each of three independent experimental replicates; *P* values were calculated by one-way ANOVA based on the proportion of mitochondria with ERMCSs. (**E**) Quantification of the proportion of normal mitochondria relative to total mitochondria from TEM images in (**B**). This analysis was performed using three independent experimental replicates. A total of >35 cells were counted per group, pooling data from the three replicates. Data are presented as mean ± SD; *P* values were calculated by one-way ANOVA. Source data are available online for this figure.

**Figure EV2:**
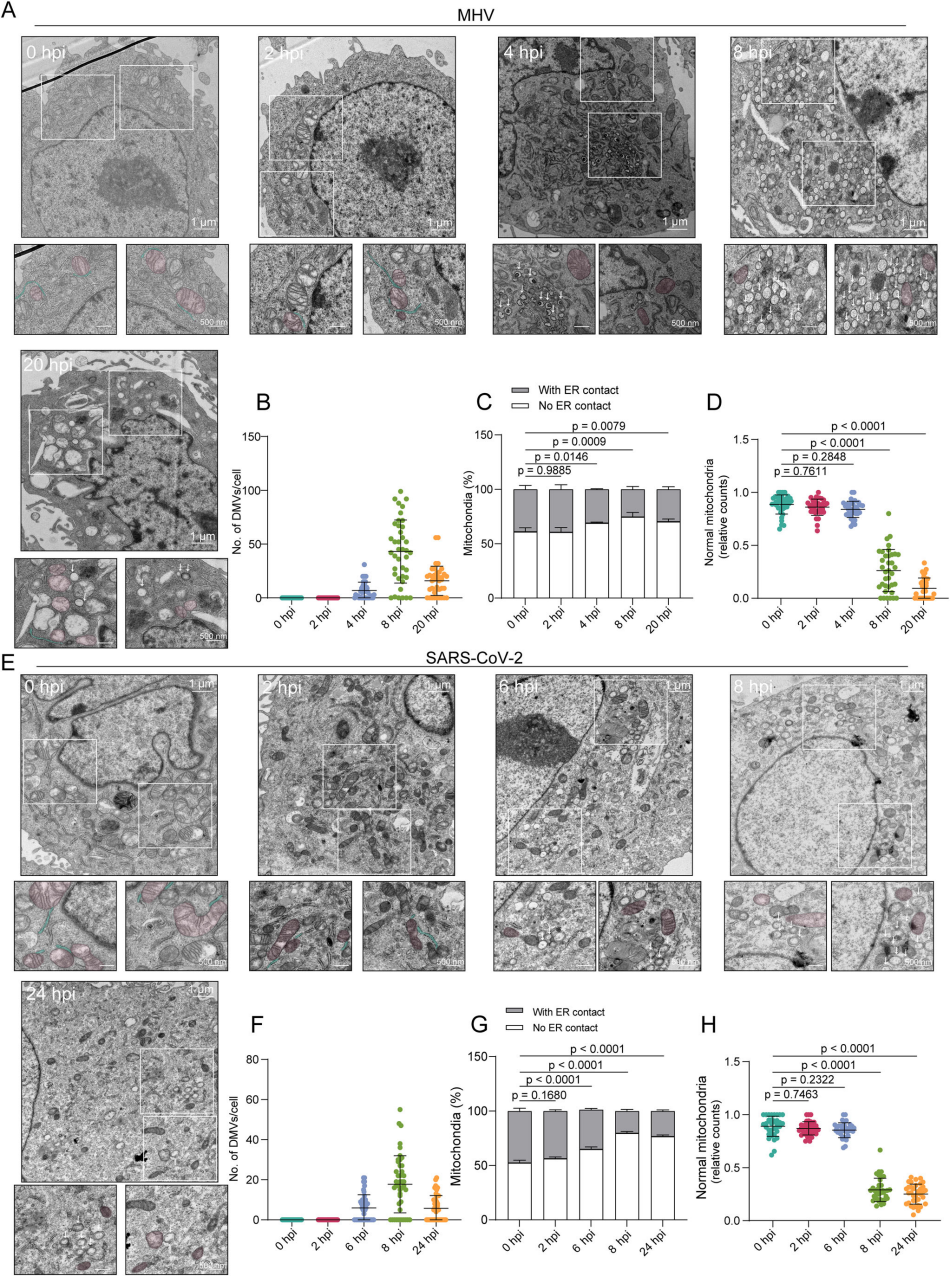
Time-course analysis of DMV biogenesis, mitochondrial morphological abnormalities, and ERMCS dynamics in MHV- and SARS-CoV-2-infected cells. (**A**) Representative TEM images of mitochondrial morphology and DMV formation in MHV-infected 17Cl-1 cells (MOI = 5; hpi = 0, 2, 4, 8, 20). Zoomed-in views (bottom) highlight mitochondrial structural changes and DMV accumulation over time. Scale bars, 1 μm (main images); 500 nm (zoomed-in views). (**B**) Quantification of DMV numbers per cell from TEM images in (**A**). Data are presented as mean ± SD (*n* > 30 cells per time point); *P* values were calculated by one-way ANOVA. (**C**) Quantification of the proportion of mitochondria with or without ER contact from TEM images in (**A**). Data are presented as mean ± SD (*n* > 30 cells per time point); *P* values were calculated by one-way ANOVA based on the proportion of mitochondria with ERMCSs. (**D**) Quantification of the proportion of normal mitochondria relative to total mitochondria from TEM images in (**A**). This analysis was performed using three independent experimental replicates. A total of >35 cells were counted per group, pooling data from the three replicates. Data are presented as mean ± SD; *P* values were calculated by one-way ANOVA. (**E**) Representative TEM images of mitochondrial morphology and DMV formation in SARS-CoV-2-infected Vero E6 cells (MOI = 2; hpi = 0, 2, 6, 8, 24). Zoomed-in views (bottom) highlight mitochondrial structural changes and DMV accumulation over time. Scale bars, 1 μm (main images); 500 nm (zoomed-in views). (**F**) Quantification of DMV numbers per cell from TEM images in (**E**). (**G**) Quantification of the proportion of mitochondria with or without ER contact from TEM images in (**E**). Data are presented as mean ± SD (*n* > 30 cells per time point); *P* values were calculated by one-way ANOVA based on the proportion of mitochondria with ERMCSs. (**H**) Quantification of the proportion of normal mitochondria relative to total mitochondria from TEM images in (**E**). This analysis was performed using three independent experimental replicates. A total of >35 cells were counted per group, pooling data from the three replicates. Data are presented as mean ± SD; *P* values were calculated by one-way ANOVA. Source data are available online for this figure.

To investigate the temporal relationship between DMV biogenesis and mitochondrial dysfunction, we employed a doxycycline (DOX)-inducible system for co-expressing NSP3 and NSP4. Immunofluorescence staining showed that NSP3 and NSP4 expression was barely detectable at 0 h post-DOX induction, with progressive accumulation over 12–36 h (Fig. EV1A). Consistent with this temporal pattern, TEM confirmed no DMVs at 0 h, and a marked increase in DMV numbers by 12–36 h post-induction (Fig. EV1B,C). Parallel quantitative analyses revealed a significant decrease in the proportion of structurally normal mitochondria during the 24-36 h induction period (Fig. EV1E). These results demonstrate mitochondrial damage is not an immediate consequence of viral protein expression, but a delayed, progressive consequence of DMV accumulation. SARS-CoV-2 and murine hepatitis virus (MHV)-infected cells similarly exhibited impaired mitochondrial structural integrity. DMVs were detectable during early stages of biogenesis (4 hpi for MHV, 6 hpi for SARS-CoV-2), with mitochondrial structure remaining normal in both models at these time points. Notably, mitochondrial structural damage was observed starting from 8 hpi in both infection models, coinciding with progressive DMV accumulation over the subsequent hours (Fig. EV2A,B,D–F,H).

**Figure EV3:**
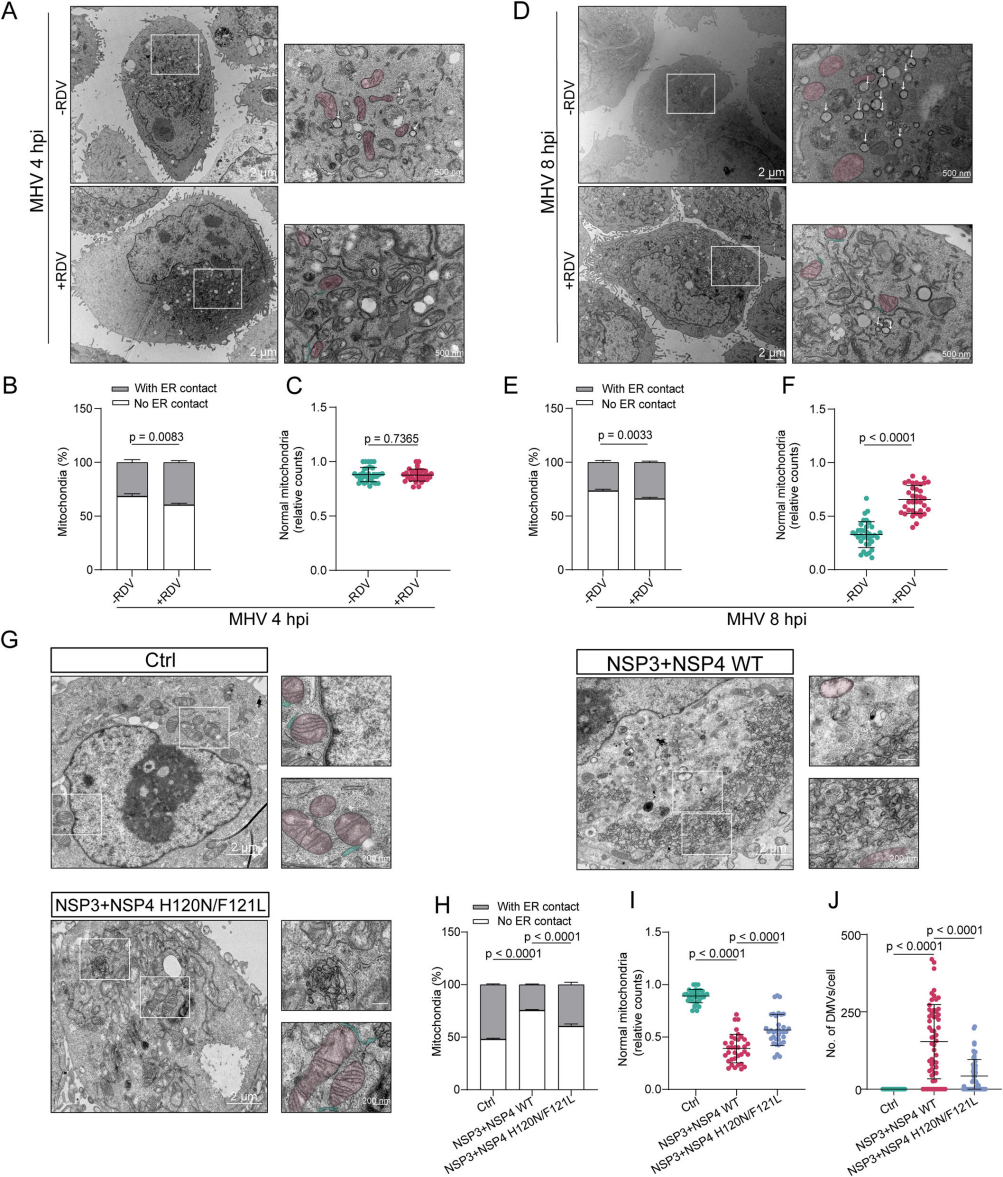
Inhibition of DMV biogenesis protects against mitochondrial morphological abnormalities and ERMCSs loss. (**A**) Representative TEM images of MHV-infected 17Cl-1 cells treated with or without RDV at 4 hpi. Zoomed-in views show mitochondrial morphology and DMV formation. Scale bars, 2 μm (main panels); 500 nm (zoomed panels). (**B**) Quantification of the proportion of mitochondria with or without ER contact from TEM images in (**A**). Data are presented as mean ± SD (*n* = 3 independent experimental replicates, *n* > 35 cells per group); *P* values were calculated by unpaired *t* test. (**C**) Quantification of the proportion of normal mitochondria relative to total mitochondria from TEM images in (A). This analysis was performed using three independent experimental replicates. A total of >35 cells were counted per group, pooling data from the three replicates. Data are presented as mean ± SD; *P* values were calculated by unpaired *t* test. (**D**) Representative TEM images of MHV-infected 17Cl-1 cells treated with or without RDV at 8 hpi. Zoomed-in views (right) show mitochondrial morphology and DMV formation. Scale bars, 2 μm (main images); 500 nm (zoomed-in views). (**E**) Quantification of the proportion of mitochondria with or without ER contact from TEM images in (**D**). Data are presented as mean ± SD (*n* = 3 independent experimental replicates, *n* > 35 cells per group); *P* values were calculated by unpaired *t* test. (**F**) Quantification of the proportion of normal to total mitochondria from TEM images in (**D**). This analysis was performed using three independent experimental replicates. A total of >35 cells were counted per group, pooling data from the three replicates. Data are presented as mean ± SD; *P* values were calculated by unpaired *t* test. (**G**) Representative TEM images of HeLa cells transfected with empty vector (Ctrl), WT GFP-NSP3 + NSP4-mCherry, or GFP-NSP3 + NSP4-mCherry (H120N/F121L mutant). Zoomed-in views (right) show mitochondrial morphology and DMV formation. Scale bars, 2 μm (main images); 200 nm (zoomed-in views). (**H**) Quantification of the proportion of mitochondria with or without ER contact from TEM images in (**G**). Data are presented as mean ± SD (*n* > 35 cells per group); *P* values were calculated by one-way ANOVA. (**I**) Quantification of the proportion of normal mitochondria relative to total mitochondria from TEM images in (**G**). This analysis was performed using three independent experimental replicates. A total of >35 cells were counted per group, pooling data from the three replicates. Data are presented as mean ± SD; *P* values were calculated by one-way ANOVA. (**J**) Quantification of DMV numbers per cell from TEM images in (**G**). Data are presented as mean ± SD (*n* > 40 cells per group); *P* values were calculated by one-way ANOVA. Source data are available online for this figure.

To confirm that DMV biogenesis directly induces mitochondrial damage, we used pharmacological treatment to reduce DMV formation. Consistent with our previous findings, remdesivir (RDV) treatment reduces DMV size and abundance at both 4 and 8 hpi in MHV-infected cells (Yang et al, 2025). Mitochondrial structure maintained intact in both RDV-treated and untreated groups at 4 hpi, which was consistent with the time-course results showing that the early stage of DMV biogenesis did not affect the mitochondrial morphology. At 8 hpi, untreated cells exhibited robust DMV accumulation and severe mitochondrial structural damage, whereas RDV-treated cells showed reduced DMV load and significantly attenuated mitochondrial impairment (Fig. EV3A,C,D,F).

We also validated this causal link using a mutant viral protein-driven DMV formation system. Previous study has demonstrated that NSP4 residues H120 and F121 are critical for inducing membrane rearrangements, and their mutation impairs virus production in SARS-CoV (Sakai et al, 2017), with these residues conserved across SARS-CoV, MHV and MERS-CoV. We then generated a SARS-CoV-2 NSP4 H120N/F121L mutant, and found that co-expression of NSP3 and the NSP4 H120N/F121L mutant led to a marked reduction in DMV formation, with mitochondrial structural integrity largely preserved (Fig. EV3G,I,J). Collectively, our temporal results in DOX-inducible and viral infection models, pharmacological inhibition, and genetic mutation of the conserved NSP4 membrane rearrangement motif together demonstrate that DMV biogenesis directly drives mitochondrial structural damage during coronavirus infection. This impairment is a delayed, progressive outcome of DMV accumulation rather than an immediate effect of viral protein expression or initial DMV formation.

### ERMCSs play a critical role in DMV-induced mitochondrial damage and coronavirus replication

**Figure EV4:**
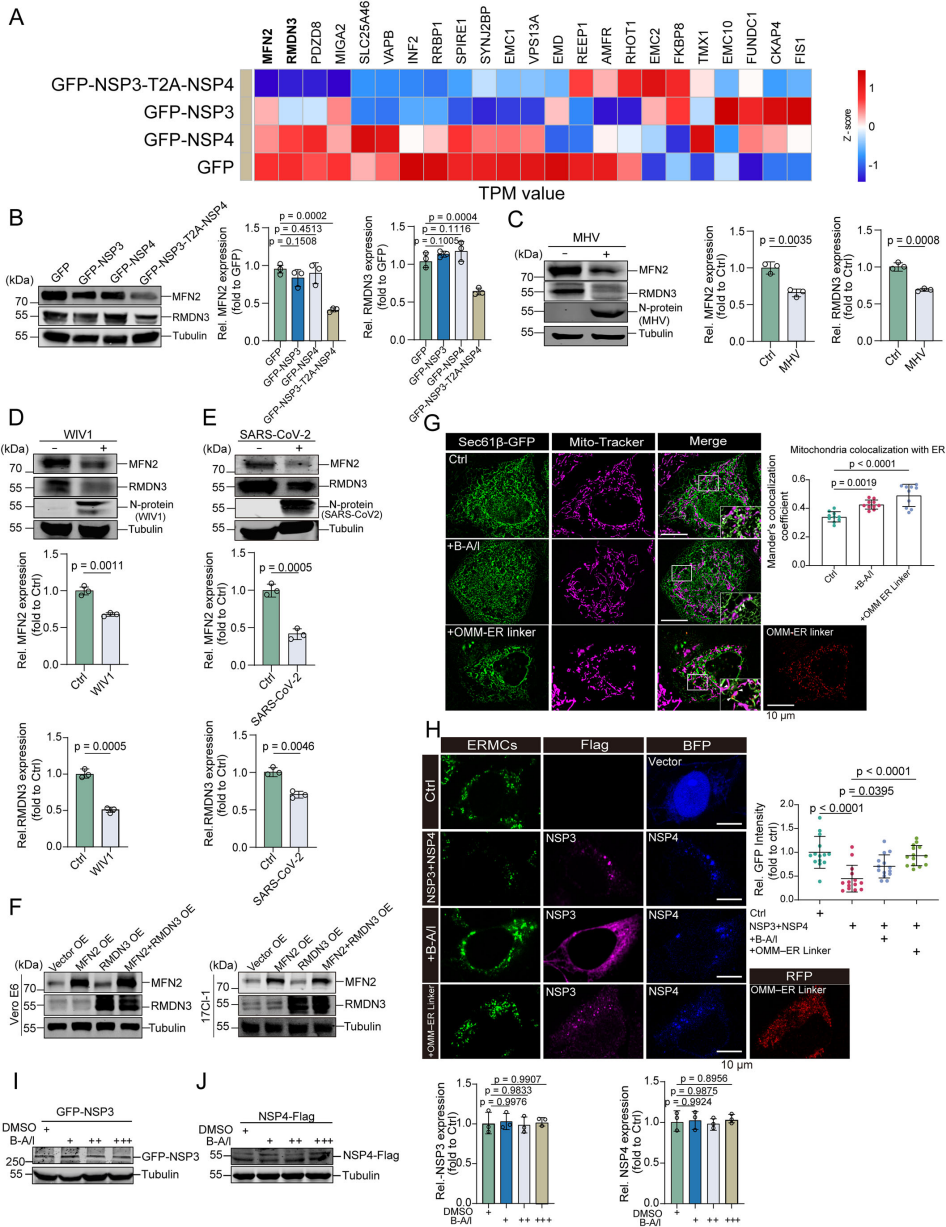
DMV formation downregulates the expression of ERMCS proteins MFN2 and RMDN3. (**A**) Heatmap displaying transcript levels (TPM values, z-score normalized) of ERMCSs-related genes in 293T cells expressing GFP-vector, GFP-NSP3, GFP-NSP4, or GFP-NSP3-T2A-NSP4. (**B**) Immunoblotting and quantification of MFN2 and RMDN3 protein levels in 293T cells expressing GFP-vector, GFP-NSP3, GFP-NSP4, or GFP-NSP3-T2A-NSP4. Data are presented as mean ± SD (*n* = 3 independent experimental replicates); *P* values were calculated by one-way ANOVA. (**C**) Immunoblotting and quantification of MFN2 and RMDN3 in uninfected or MHV-infected 17Cl-1 cells (MOI = 5, hpi = 24). Data are presented as mean ± SD (*n* = 3 independent experimental replicates); *P* values were calculated by unpaired *t* test. (**D**) Immunoblotting and quantification of MFN2 and RMDN3 in uninfected or WIV1-infected Vero E6 cells (MOI = 1, hpi = 24). Data are presented as mean ± SD (*n* = 3 independent experimental replicates); *P* values were calculated by unpaired *t* test. (**E**) Immunoblotting and quantification analysis of MFN2 and RMDN3 in uninfected or SARS-CoV-2-infected Vero E6 cells (MOI = 1, hpi = 24). Data are presented as mean ± SD (*n* = 3 independent experimental replicates); *P* values were calculated by unpaired *t* test. (**F**) Immunoblotting analysis of MFN2 and RMDN3 in cells stably overexpressing vector control (Vector OE), MFN2, RMDN3, or MFN2 + RMDN3. (**G**) Representative fluorescence images of HeLa cells expressing SEC61B-GFP (ER marker) and Mito-Tracker treated with B-A/I (10 μM) or transfected with an OMM-ER linker. Scale bar, 10 μm. Quantification of mitochondria-ER colocalization (assessed by Manders’ colocalization coefficient) is shown in the right panel. Data are presented as mean ± SD (*n* = 10 cells per group); *P* values were calculated by one-way ANOVA. (**H**) Representative fluorescence images of ERMCSs reporter in HeLa cells co-expressing Flag-NSP3 and NSP4-BFP, treated with B-A/l (10 μM) or transfected with an OMM-ER linker. Quantification of relative GFP fluorescence intensity is shown in the right panel. Data are presented as mean ± SD (*n* = 15 cells per group); *P* values were calculated by one-way ANOVA. (**I**) Immunoblotting analysis of GFP-NSP3 protein levels in HeLa cells treated with DMSO or increasing concentrations of B-AI (5 μM, 10 μM, 20 μM). Data are presented as mean ± SD (*n* = 3 independent experimental replicates); *P* values were calculated by one-way ANOVA. (**J**) Immunoblotting analysis of NSP4-Flag protein levels in HeLa cells treated with DMSO or increasing concentrations of B-AI (5 μM, 10 μM, 20 μM). Data are presented as mean ± SD (*n *= 3 independent experimental replicates); *P* values were calculated by one-way ANOVA. Source data are available online for this figure.

We next aimed to define the mechanism by which DMV formation drives mitochondrial dysfunction and morphological abnormalities. Transcriptome analysis revealed marked downregulation of key ERMCSs tethering factors, including MFN2, RMDN3, PDZD8, and MIGA2 in cells with DMV formation compared to controls (cells expressing NSP3 alone, NSP4 alone, or transfected with empty vector; Fig. EV4A). Given that DMVs are rearranged from ER-derived membranes, and the established role of ERMCSs in regulating mitochondrial homeostasis (Wolff et al, 2020; Krols et al, 2016; Csordás et al, 2018), we tested whether DMVs induce mitochondrial impairment via ERMCSs. Since MFN2 and RMDN3 are known to cooperate in maintaining ERMCS integrity (Shiiba et al, 2025), we focused on their potential role in DMV-induced mitochondrial defects. Immunoblotting confirmed progressive downregulation of MFN2 and RMDN3 in cells co-expressing NSP3 and NSP4, but not in cells expressing either protein alone. Moreover, MFN2 and RMDN3 downregulation was also observed in cells infected with coronaviruses, including MHV, the bat sarbecovirus WIV1-CoV (WIV1), and SARS-CoV-2 (Fig. EV4B–E).

We next quantified ERMCSs in TEM images, revealing a significant reduction in the proportion of ER-contacted mitochondria in cells co-expressing NSP3 and NSP4 (Fig. 1C). This impairment showed both time- and concentration-dependent patterns. In the DOX-inducible system, the proportion of ER-contacted mitochondria began to decrease from the early stage of DMV formation (DOX 12 h) and declined progressively over 12–36 h of induction, correlating with time-dependent DMV accumulation (Fig. EV1B,D). Meanwhile, higher NSP3 and NSP4 expression levels further enhanced DMV formation and exacerbated ERMCSs loss (Fig. 1D,F). Additionally, cells expressing the DMV biogenesis-defective NSP4 H120N/F121L mutant exhibited largely preserved ERMCSs (Fig. EV3G,H), confirming that NSP3-NSP4 co-expression impairs ERMCSs in a DMV-dependent manner, and that DMV biogenesis is the primary driver of this impairment. Consistent with TEM findings, the signal from our previously reported split-GFP ERMCSs reporter system (GFP1-10-SEC61B and TOMM20-GFP-11) (Zhou et al, 2023; Li et al, 2023b) was significantly reduced in cells co-expressing NSP3 and NSP4 (Fig. 1G).

Similarly, ERMCSs reduction was detected in viral infection models as well. In MHV- and SARS-CoV-2-infected cells, the proportion of ER-contacted mitochondria decreased as early as the initial stage of DMV formation (4 hpi for MHV, 6 hpi for SARS-CoV-2), coinciding with the first detection of DMVs in these models. Moreover, increased DMV accumulation during infection led to further ERMCSs loss (Fig. EV2A,C,E,G). In MHV-infected cells treated with RDV, where DMV load was reduced, the reduction in the proportion of ER-contacted mitochondria was significantly alleviated at both 4 hpi and 8 hpi (Fig. EV3A,B,D,E), demonstrating that DMV reduction can restore ERMCSs.

Collectively, this temporal correlation reinforces that ERMCSs loss is an early event linked to DMV biogenesis, rather than a late consequence of viral infection, and that DMV-induced ERMCSs loss occurs prior to DMV-mediated mitochondrial structural abnormalities.

**Figure 2:**
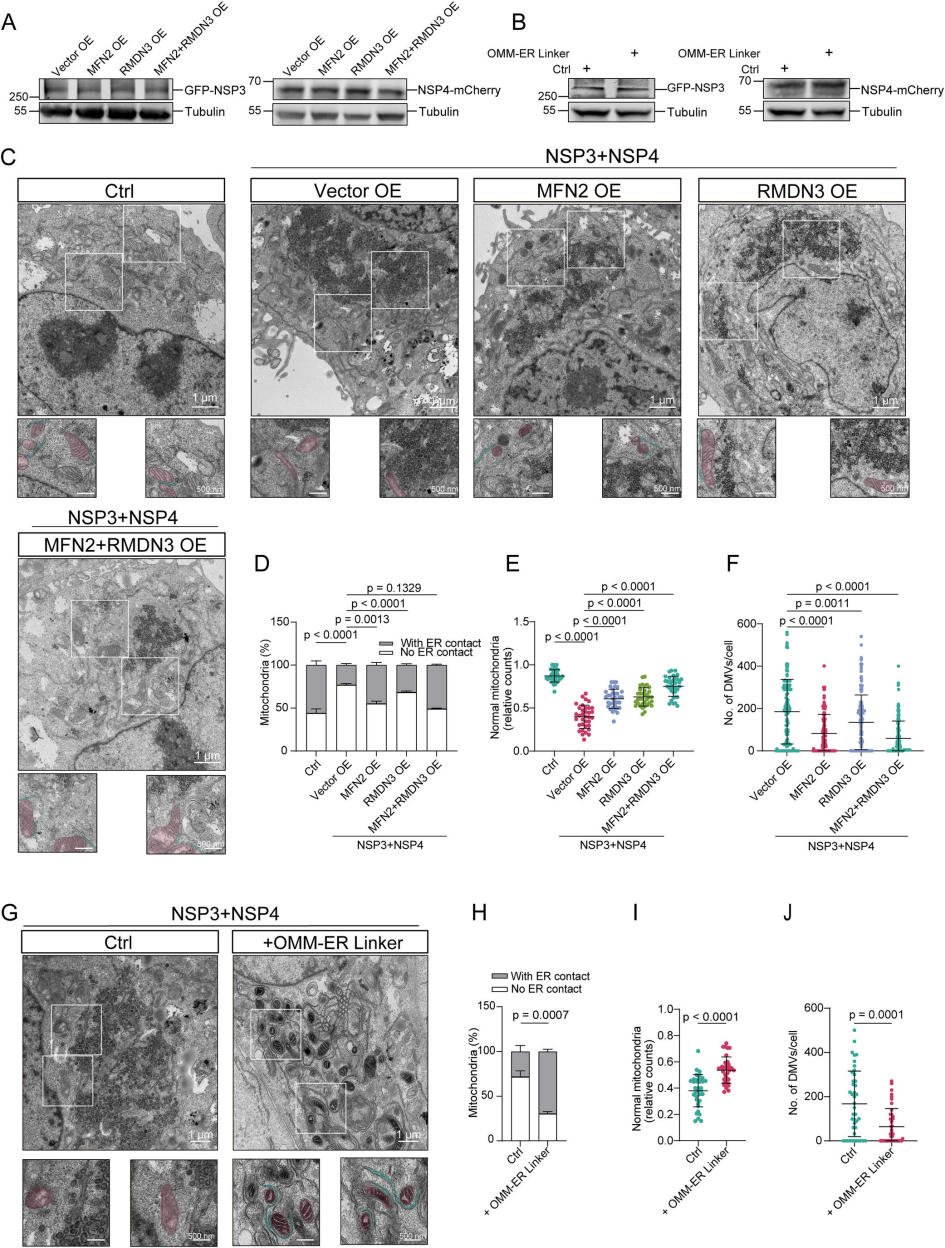
Enforcing ER-mitochondria tethering alleviates mitochondrial defects and DMV formation induced by NSP3 and NSP4 co-expression. (**A**) Immunoblotting analysis of GFP-NSP3 and NSP4-mCherry expression in HeLa cells stably overexpressing vector control (Vector OE), MFN2, RMDN3, or MFN2 + RMDN3, following transient transfection with GFP-NSP3 and NSP4-mCherry expression plasmids. (**B**) Immunoblotting analysis of GFP-NSP3 and NSP4-mCherry protein levels in HeLa cells overexpressing a control vector (Ctrl) or an outer mitochondrial membrane (OMM)-ER linker, following transient transfection with GFP-NSP3 and NSP4-mCherry expression plasmids. (**C**) Representative TEM images of mitochondrial morphology and DMV formation in HeLa cells stably overexpressing Vector OE, MFN2, RMDN3, or MFN2 + RMDN3, followed by transient transfection to co-express GFP-NSP3 and NSP4-mCherry. Scale bars, 1 μm (main images); 500 nm (zoomed-in views). Zoomed-in views highlight ER-mitochondria contact sites. (**D**) Quantification of the proportion of mitochondria with or without ER contact from TEM images in (**C**). Data are presented as mean ± SD, with *n* > 30 cells per group; *P* values were calculated by one-way ANOVA based on the proportion of mitochondria with ERMCSs. (**E**) Quantification of the proportion of normal mitochondria relative to total mitochondria from TEM images in (**C**). This analysis was performed using three independent experimental replicates. A total of >35 cells were counted per group, pooling data from the three replicates. Data are presented as mean ± SD; *P* values were calculated by one-way ANOVA. (**F**) Quantification of DMV numbers per cell from TEM images in (**C**). Data are presented as mean ± SD, with n > 100 cells per group; *P* values were calculated by one-way ANOVA. (**G**) Representative TEM images of mitochondrial morphology and DMV formation induced by co-expressing GFP-NSP3 and NSP4-mCherry in HeLa cells overexpressing a control vector or an OMM-ER linker. Scale bars, 1 μm (main images); 500 nm (zoomed-in views). Zoomed-in views highlight ER-mitochondria contact sites. (**H**) Quantification of the proportion of mitochondria with or without ER contact from TEM images in (**G**). Data are presented as mean ± SD, with *n* > 35 cells per group; *P* value was calculated by unpaired *t* test based on the proportion of mitochondria with ERMCSs. (**I**) Quantification of the proportion of normal mitochondria relative to total mitochondria from TEM images in (**G**). This analysis was performed using three independent experimental replicates. A total of >35 cells were counted per group, pooling data from the three replicates. Data are presented as mean ± SD; *P* value was calculated by unpaired *t* test. (**J**) Quantification of DMV numbers per cell from TEM images in (**G**). Data are presented as mean ± SD, with *n* > 35 cells per group; *P* value was calculated by unpaired *t* test. Source data are available online for this figure.

**Figure EV5:**
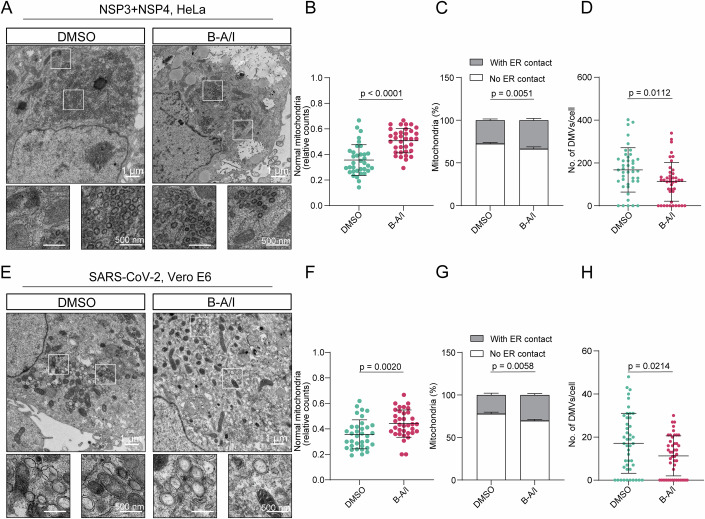
MFN2 agonist B-A/l alleviates mitochondrial defects and DMV formation induced by co-expressing NSP3 and NSP4 or SARS-CoV-2 infection. (**A**) Representative TEM images showing mitochondrial morphology and DMV formation in HeLa cells co-expressing NSP3 and NSP4, treated with DMSO or the MFN2 agonist B-A/l (10 μM). Scale bars, 1 μm (main images); 500 nm (zoomed-in views). Zoomed-in views highlight mitochondrial structures and DMV formations. (**B**) Quantification of the proportion of normal to total mitochondria from TEM images in (**A**). This analysis was performed using three independent experimental replicates. A total of >35 cells were counted per group, pooling data from the three replicates. Data are presented as mean ± SD; *P* values were calculated by unpaired *t* test. (**C**) Quantification of the proportion of mitochondria with or without ER contact from TEM images in (**A**). Data are presented as mean ± SD (*n* > 35 cells per group); *P* value was calculated by unpaired *t* test based on the proportion of mitochondria with ERMCSs. (**D**) Quantification of DMV numbers from TEM images in (**A**). Data are presented as mean ± SD (*n* > 35 cells per group); *P* value was calculated by unpaired *t* test. (**E**) Representative TEM images showing mitochondrial morphology and DMV formation in SARS-CoV-2-infected Vero E6 cells (MOI = 1; hpi = 8) treated with DMSO or MFN2 agonist B-A/l (10 μM). Scale bars, 1 μm (main images); 500 nm (zoomed-in views). Zoomed-in views highlight mitochondrial structures and DMV formations. (**F**) Quantification of the proportion of normal to total mitochondria from TEM images in (**E**). This analysis was performed using three independent experimental replicates. A total of >35 cells were counted per group, pooling data from the three replicates. Data are presented as mean ± SD; *P* values were calculated by unpaired *t* test. (**G**) Quantification of the proportion of mitochondria with or without ER contact from TEM images in (**E**). Data are presented as mean ± SD (*n* > 35 cells per group); *P* value was calculated by unpaired *t* test based on the proportion of mitochondria with ERMCSs. (**H**) Quantification of DMV numbers from TEM images in (**E**). Data are presented as mean ± SD (*n* > 35 cells per group); and *P* value was calculated by unpaired *t* test. Source data are available online for this figure.

Next, we investigated whether enhancing ERMCSs could mitigate the mitochondrial damage induced by DMV formation. First, in our established stable cell lines, we confirmed that overexpression of MFN2, RMDN3, or their combination did not alter the expression of NSP3 or NSP4 individually, ensuring that phenotypic changes were not caused by altered viral protein expression (Figs. 2A and EV4F). TEM analysis revealed that overexpression of either MFN2 or RMDN3 in NSP3 and NSP4 co-expressing cells significantly restored ERMCSs. Co-overexpression of MFN2 and RMDN3 yielded the most robust rescue effect (Fig. 2C,D). In turn, this restoration of ERMCSs led to a reduction in the proportion of mitochondria with abnormal morphology (Fig. 2E). Notably, strengthening of ERMCSs could counteract DMV formation in cells (Fig. 2F), implying that ERMCSs act as a key mediator modulating DMV formation and maintaining mitochondrial homeostasis under DMV-inducing conditions. To orthogonally validate these findings, we utilized the MFN2 agonist B-A/l (Liu et al, 2025; Rocha et al, 2018) and confirmed B-A/l treatment did not affect the expression of NSP3 or NSP4 (Fig. EV4I,J). Following treatment, we observed an increase in ERMCSs number, amelioration of abnormal mitochondrial morphology, and inhibition of DMV formation (Figs. EV4G,H and EV5A–D). Furthermore, we employed a synthetic outer mitochondrial membrane (OMM)-ER tethering linker, which enforces close apposition between the two membranes while preserving their identities (Csordás et al, 2006). We confirmed the OMM-ER tethering linker did not alter individual viral protein expression (Fig. 2B). Expression of the tether significantly restored ERMCSs, thereby reducing mitochondrial abnormalities and decreasing DMV formation (Figs. 2G–J and EV4G,H). Together, these results indicate that ERMCSs play a central role in mediating DMV-induced mitochondrial dysfunction and that the reduction of ERMCSs facilitates DMV biogenesis.

**Figure 3:**
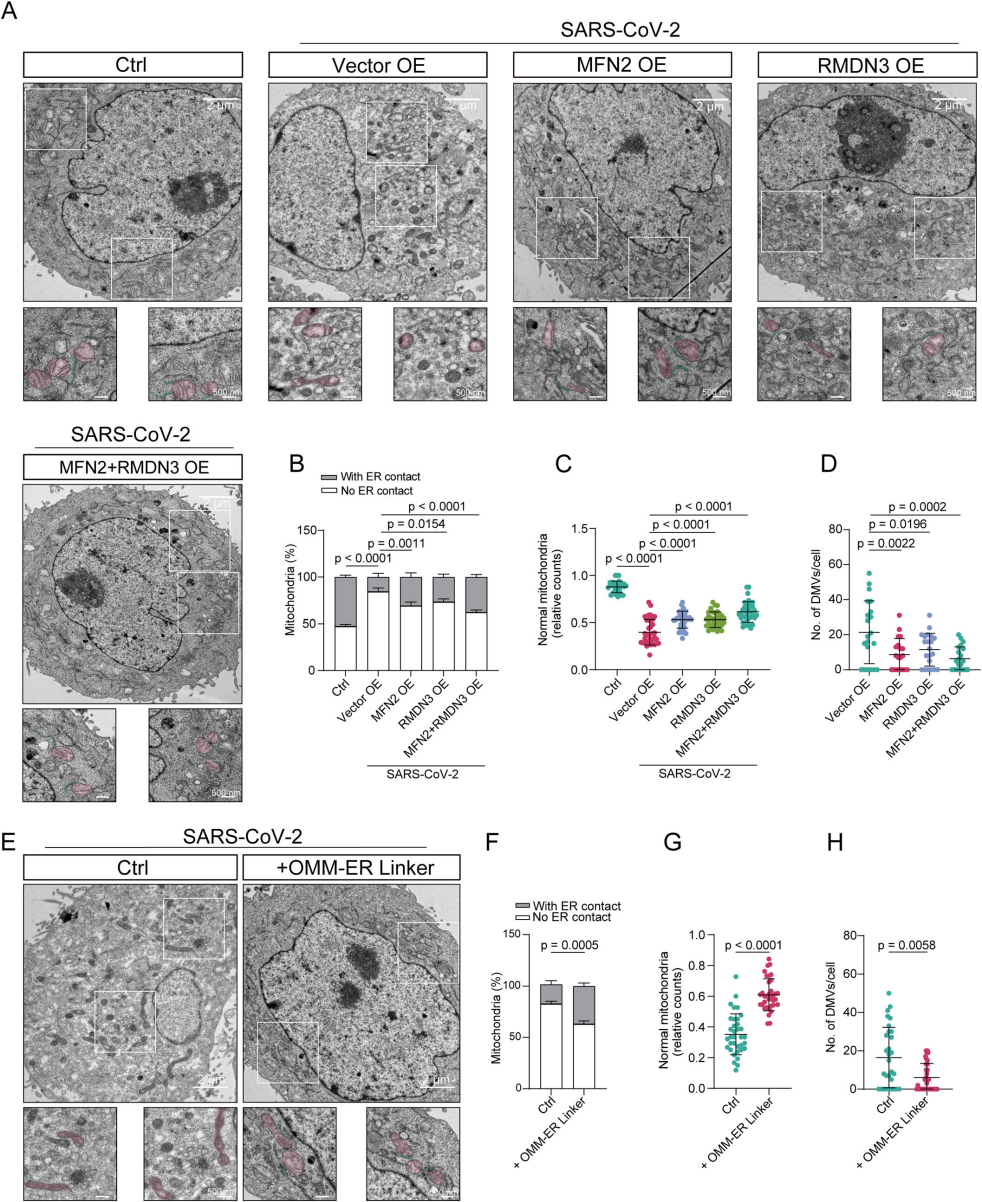
Enforcing ER-mitochondria tethering alleviates mitochondrial defects and DMV formation induced by SARS-CoV-2 infection. (**A**) Representative TEM images of mitochondrial morphology and DMV formation in SARS-CoV-2-infected Vero E6 cells (MOI = 2, hpi = 8) stably overexpressing vector control (Vector OE), MFN2, RMDN3, or MFN2 + RMDN3. Scale bars, 2 μm (main images); 500 nm (zoomed-in views). Zoomed-in views highlight direct ERMCSs. (**B**) Quantification of the proportion of mitochondria with or without ER contact from TEM images in (**A**). Data are presented as mean ± SD (*n* > 30 cells per group); *P* values were calculated by one-way ANOVA based on the proportion of mitochondria with ERMCSs. (**C**) Quantification of the proportion of normal mitochondria relative to total mitochondria from TEM images in (**A**). This analysis was performed using three independent experimental replicates. A total of >35 cells were counted per group, pooling data from the three replicates. Data are presented as mean ± SD; *P* values were calculated by one-way ANOVA. (**D**) Quantification of DMV numbers per cell from TEM images in (**A**). Data are presented as mean ± SD (*n* > 20 cells per group); *P* values were calculated by one-way ANOVA. (**E**) Representative TEM images of mitochondrial morphology and DMV formation in SARS-CoV-2-infected Vero E6 cells (MOI = 2, hpi = 8) overexpressing a vector control or an OMM-ER linker. Scale bars, 2 μm (main images); 500 nm (zoomed-in views). Zoomed-in views highlight direct ERMCSs. (**F**) Quantification of the proportion of mitochondria with or without ER contact from TEM images in (**E**). Data are presented as mean ± SD (*n* > 30 cells per group); *P* value was calculated by unpaired *t* test based on the proportion of mitochondria with ERMCSs. (**G**) Quantification of the proportion of normal mitochondria relative to total mitochondria from TEM images in (**E**). This analysis was performed using three independent experimental replicates. A total of >35 cells were counted per group, pooling data from the three replicates. Data are presented as mean ± SD; *P* value was calculated by unpaired *t* test. (**H**) Quantification of DMV numbers from TEM images in (**E**). Data are presented as mean ± SD (*n* > 30 cells per group); *P* value was calculated by unpaired *t* test. Source data are available online for this figure.

We further explored whether enforcing ER-mitochondria tethering could mitigate the mitochondrial damage induced by authentic virus infection. TEM analysis demonstrated that overexpression of MFN2 or RMDN3, or their co-overexpression, significantly increased the number of ERMCSs in SARS-CoV-2-infected cells (Fig. 3A,B). Consistent with observations in the NSP3 and NSP4 expression system, the restoration of ERMCSs was associated with a reduction in the proportion of mitochondria exhibiting abnormal morphology and fewer DMVs. The strongest suppression was again achieved with dual overexpression of MFN2 and RMDN3 (Fig. 3C,D). Similarly, treatment with the MFN2 agonist B-A/l or expression of the synthetic OMM-ER linker also effectively restored ERMCSs, ameliorated mitochondrial abnormalities and inhibited DMV formation in SARS-CoV-2-infected cells (Figs. 3E–H and EV5E–H).

**Figure 4:**
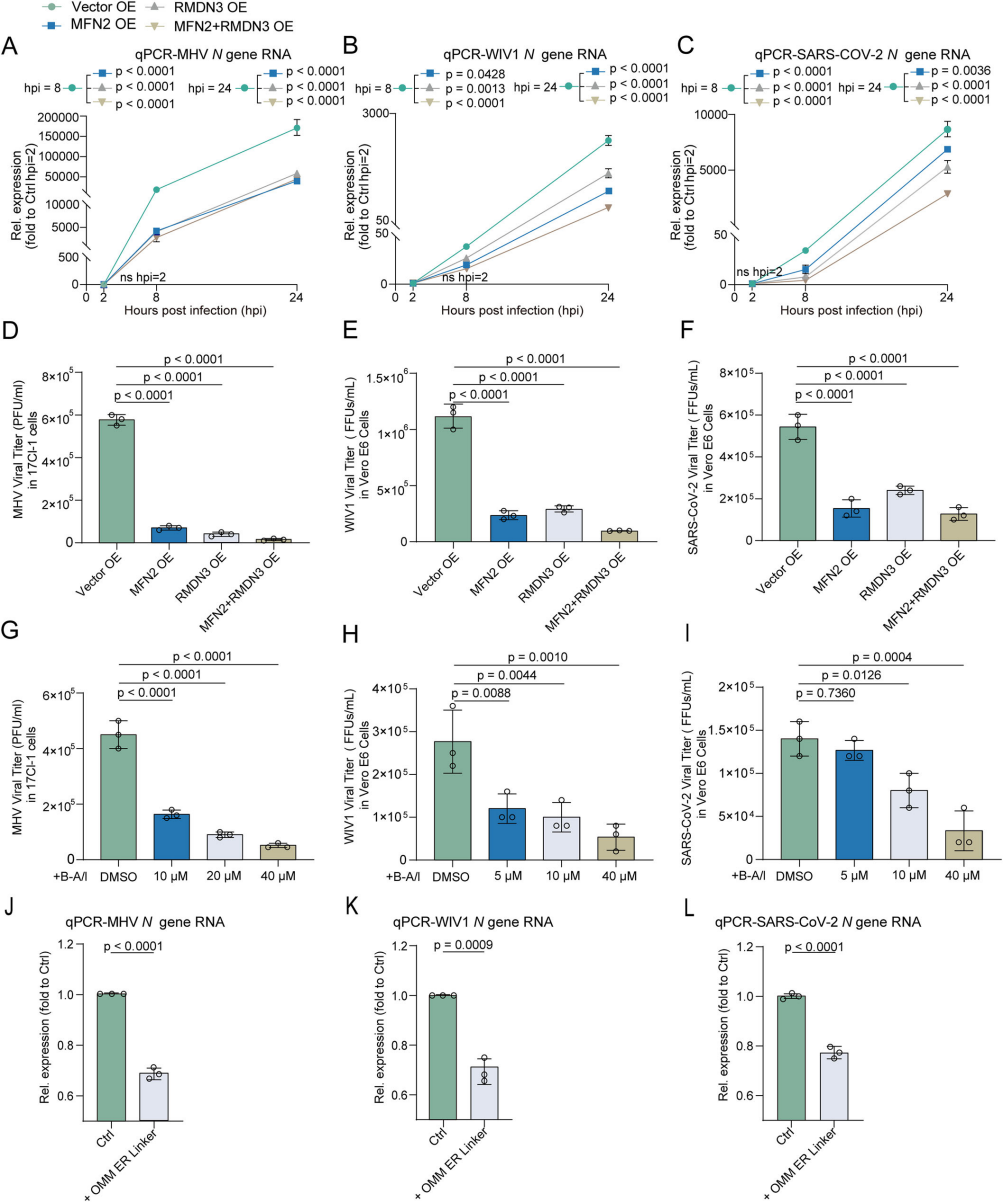
Enforcing ER-mitochondria tethering restricts coronavirus infection. (**A**–**C**) Time-course qPCR analysis of viral genomic RNA (gRNA) levels in: (**A**) MHV-infected 17Cl-1 cells (MOI = 10; hpi = 2, 8, 24) stably overexpressing vector control (Vector OE), MFN2, RMDN3, or MFN2 + RMDN3; (**B**) WIV1-infected Vero E6 cells (MOI = 1; hpi = 2, 8, 24) stably overexpressing the same constructs; (**C**) SARS-CoV-2-infected Vero E6 cells (MOI = 1; hpi = 2, 8, 24) stably overexpressing the same constructs. Data are presented as mean ± SD (*n* = 3 independent experimental replicates). No significant differences in viral gRNA levels were observed at 2 hpi. *P* values (one-way ANOVA) for 2 hpi comparisons: MHV: Vector vs. MFN2, *P* = 0.0509; Vector vs. RMDN3, *P* = 0.8260; Vector vs. MFN2 + RMDN3, *P* = 0.0966. WIV1: Vector vs. MFN2, *P* = 0.9492; Vector vs. RMDN3, *P* = 0.6107; Vector vs. MFN2 + RMDN3, *P* = 0.6251. SARS-CoV-2: Vector vs. MFN2, *P* = 0.8652; Vector vs. RMDN3, *P* = 0.9820; Vector vs. MFN2 + RMDN3, *P* = 0.7640. (**D**–**F**) Quantification of extracellular viral titer in: (**D**) MHV-infected 17Cl-1 cells (MOI = 10, hpi = 24); (**E**) WIV1-infected Vero E6 cells (MOI = 1, hpi = 24); (**F**) SARS-CoV-2-infected Vero E6 cells (MOI = 1, hpi = 48), all stably overexpressing vector control (Vector OE), MFN2, RMDN3, or MFN2 + RMDN3. Data are presented as mean ± SD (*n* = 3 independent experimental replicates); *P* values were calculated by one-way ANOVA. (**G**–**I**) Quantification of extracellular viral titer in: (**G**) MHV-infected 17Cl-1 cells (MOI = 10, hpi = 24) treated with B-A/I at concentrations of 10 μM, 20 μM, or 40 μM (vehicle control: DMSO). (**H**) WIV1-infected Vero E6 cells (MOI = 1, hpi = 24) treated with B-A/I at concentrations of 5 μM, 10 μM, or 40 μM (vehicle control: DMSO). (**I**) SARS-CoV-2-infected Vero E6 cells (MOI = 1, hpi = 36) treated with B-A/I at concentrations of 10 μM, 20 μM, or 40 μM (vehicle control: DMSO). Data are presented as mean ± SD (*n* = 3 independent experimental replicates); *P* values were calculated by one-way ANOVA. (**J**–**L**) qPCR analysis of viral gRNA levels in: (**J**) MHV-infected 17Cl-1 cells (MOI = 10; hpi = 24) transiently transfected with either a vector control or an OMM-ER linker; (**K**) WIV1-infected 293 T cells (MOI = 1; hpi = 24) transiently transfected with hACE2, and either a vector control or an OMM-ER linker; (**L**) SARS-CoV-2-infected Vero E6 cells (MOI = 1; hpi = 24) transiently transfected with either a vector control or an OMM-ER linker. Data are presented as mean ± SD (*n* = 3 independent experimental replicates); *P* values were calculated by unpaired *t* test. Source data are available online for this figure.

We next sought to determine whether enforcing ER-mitochondria tethering impacts viral replication. Time-course quantitative PCR (qPCR) analysis revealed no significant differences in viral genomic RNA (gRNA) levels across all three tested viruses at 2 hpi in cells overexpressing MFN2, RMDN3 or both, indicating enforcing ER-mitochondria tethering does not affect viral entry. However, overexpression of either MFN2 or RMDN3 significantly suppressed viral gRNA replication at 8 hpi, and this inhibitory effect was further enhanced by their co-overexpression and was more pronounced at 24 hpi (Fig. 4A–C). Consistent with this, viral titers of MHV, WIV1, and SARS-CoV-2 were significantly decreased in cells expressing either tether, with the greatest reduction observed in cells co-expressing both MFN2 and RMDN3 (Fig. 4D–F). Furthermore, both pharmacological activation of MFN2 and transient transfection of an artificial OMM-ER linker similarly increased ERMCSs and thereby reduced viral gRNA levels and titers (Fig. 4G–L). Collectively, these results demonstrate that enforcing ER-mitochondria tethering effectively restricts coronavirus replication, identifying ERMCSs as both a viral-exploited vulnerability and a critical point of host defense.

### ECHS1 released from damaged mitochondria is essential for DMV formation and coronavirus infection

Our prior findings revealed that mitochondrial proteins predominated among DMV-interacting components, alongside the expected ER-derived proteins (Zhou et al, 2025). This observation prompted us to investigate how DMV-induced mitochondrial damage orchestrates the retrograde regulation of DMV biogenesis and coronavirus infection. We thus hypothesized that DMV formation triggers mitochondrial damage, leading to the release of mitochondrial proteins that positively regulate DMV assembly through a feedback mechanism. To test this, we examined the translocation of mitochondrial proteins in response to DMV induction or coronavirus infection.

**Figure EV6:**
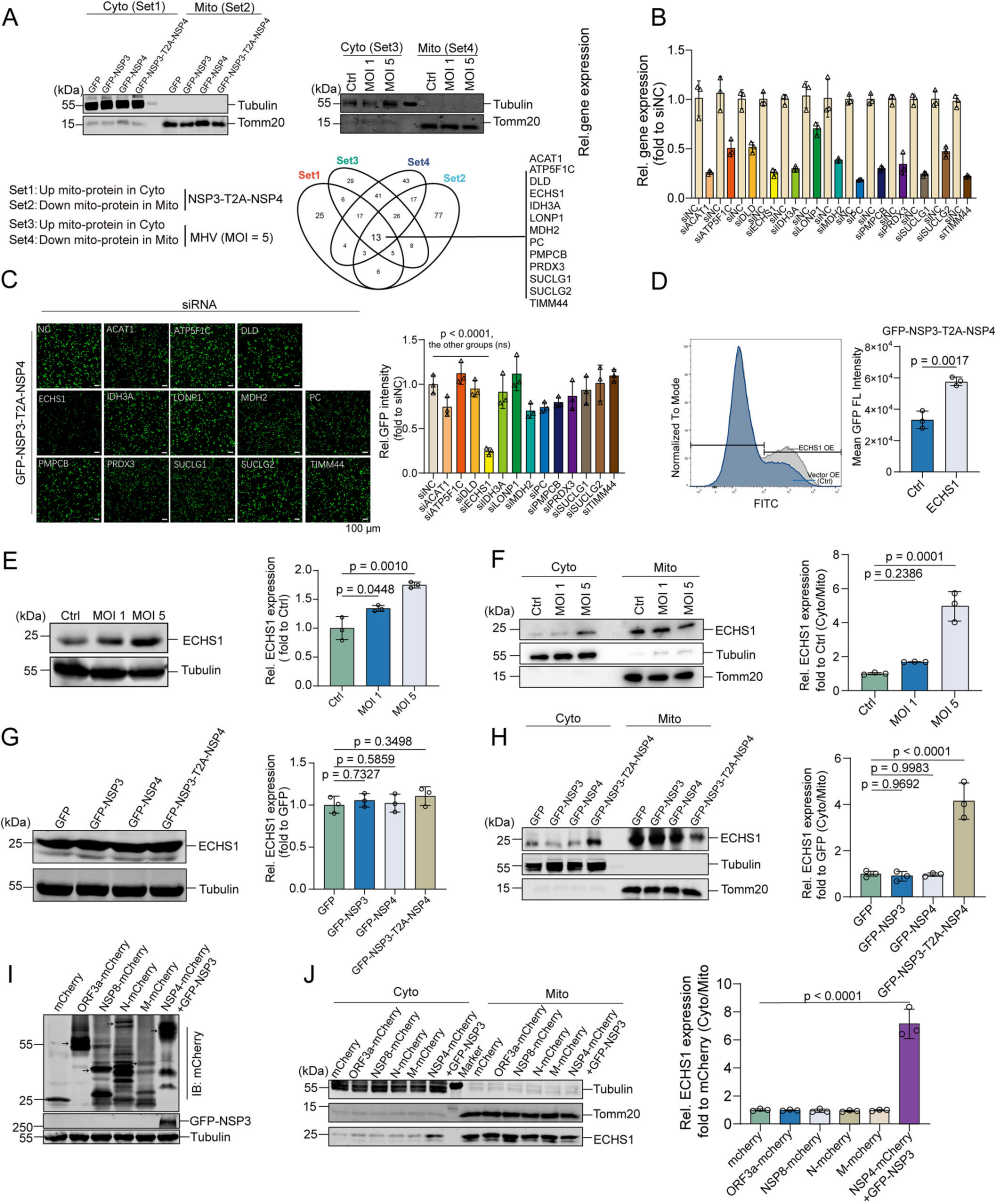
Proteomic analysis reveals that ECHS1 is released from mitochondria to the cytosol during DMV formation and coronavirus infection. (**A**) Immunoblotting analysis of cytosolic (Cyto) and mitochondrial (Mito) fractions. Left panel: 293T cells expressing GFP, GFP-NSP3, GFP-NSP4, or GFP-NSP3-T2A-NSP4. Right panel: 17Cl-1 cells uninfected (Ctrl) or infected with MHV (MOI = 5, hpi = 8). Tubulin (cytosolic marker) and Tomm20 (mitochondrial marker) were probed to verify subcellular purification efficiency. Isolated fractions were subsequently subjected to MS to identify differentially expressed mitochondrial proteins, defined as: Set1 (upregulated mitochondrial proteins in cytosol, identified by comparing GFP-NSP3-T2A-NSP4 with GFP, GFP-NSP3, or GFP-NSP4 controls); Set2 (downregulated mitochondrial proteins in mitochondria, identified by comparing GFP-NSP3-T2A-NSP4 with GFP, GFP-NSP3, or GFP-NSP4 controls); Set3 (upregulated mitochondrial proteins in cytosol during MHV infection, MOI = 5); Set4 (downregulated mitochondrial proteins in mitochondria during MHV infection, MOI = 5). Venn diagram illustrating the overlap of differentially expressed mitochondrial proteins across Sets 1–4. (**B**) qPCR analysis of the relative expression of candidate mitochondrial proteins (from (**A**)) in 293T cells treated with targeted siRNA. Expression levels were normalized to GAPDH. Data are presented as mean ± SD (*n* = 3 independent experiments); *P* values were calculated by unpaired *t* test. The corresponding *P* values are as follows: ACAT1 (*P* = 0.0019), ATP5F1C (*P* = 0.0067), DLD (*P* = 0.0015), ECHS1 (*P* < 0.0001), IDH3A (*P* < 0.0001), LONP1 (*P* = 0.0386), MDH2 (*P* = 0.0048), PC (*P* < 0.0001), PMPCB (*P* < 0.0001), PRDX3 (*P* = 0.0007), SUCLG1 (*P* < 0.0001), SUCLG2 (*P* = 0.0007), and TIMM44 (*P* < 0.0001). (**C**) Fluorescence images of GFP-NSP3-T2A-NSP4 expression in 293T cells with the indicated gene knockdowns (siNC: negative control, ACAT1, ATP5F1C, DLD, ECHS1, IDH3A, LONP1, MDH2, PC, PMPCB, PRDX3, SUCLG1, SUCLG2, TIMM44). The right panel presents quantification of relative GFP expression levels, with data shown as mean ± SD (*n *= 3 independent experimental replicates). *P* values were calculated by one-way ANOVA. Scale bar = 100 μm. (**D**) Flow cytometry histogram and quantification of GFP-NSP3-T2A-NSP4 expression. ECHS1 overexpression increases the mean GFP fluorescence intensity of GFP-NSP3-T2A-NSP4. 293T cells were transiently transfected with ECHS1 (or control vector) and GFP-NSP3-T2A-NSP4. Data are presented as mean ± SD (*n* = 3 independent experiments); *P* value was calculated by unpaired *t* test. (**E**) Immunoblotting analysis of ECHS1 in 17Cl-1 cells: uninfected (Ctrl) or infected with MHV at MOI = 1 or MOI = 5. Quantification of ECHS1 protein levels (right panel). Data are presented as Mean ± SD (*n* = 3 independent experiments); *P* values were calculated by one-way ANOVA. (**F**) Immunoblotting analysis of ECHS1 protein levels in Cyto and Mito fractions from 17Cl-1 cells: uninfected or infected with MHV at MOI = 1 or MOI = 5. Quantification of ECHS1 protein levels (normalized to the Cyto/Mito fold change of Ctrl) (right panel). Data are presented as mean ± SD (*n* = 3 independent experiments); *P* values were calculated by one-way ANOVA. (**G**) Immunoblotting analysis of ECHS1 protein levels in 293T cells expressing GFP, GFP-NSP3, GFP-NSP4, or GFP-NSP3-T2A-NSP4. Quantification of ECHS1 levels (right panel). Data are presented as mean ± SD (*n* = 3 independent experiments); *P* values were calculated by one-way ANOVA. (**H**) Immunoblotting analysis of ECHS1 protein levels in Cyto and Mito fractions from 293T cells expressing GFP, GFP-NSP3, GFP-NSP4, or GFP-NSP3-T2A-NSP4. Quantification of ECHS1 protein levels (normalized to the Cyto/Mito fold change of GFP) (right panel). Data are presented as mean ± SD (*n* = 3 independent experiments); *P* values were calculated by one-way ANOVA. (**I**) Immunoblotting analysis of 293T cells transiently transfected with mCherry (control), SARS-CoV-2 ORF3a-mCherry, SARS-CoV-2 NSP8-mCherry, SARS-CoV-2 N-mCherry, SARS-CoV-2 M-mCherry, or NSP4-mCherry + GFP-NSP3. (**J**) Immunoblotting analysis of ECHS1 protein levels in Cyto and Mito fractions from 293T cells transiently transfected with mCherry (control), ORF3a-mCherry, NSP8-mCherry, N-mCherry, M-mCherry, or NSP4-mCherry + GFP-NSP3. The right panel presents quantification of the Cyto/Mito fold change of ECHS1 (normalized to mCherry control). Data are presented as mean ± SD (*n* = 3 independent experimental replicates). *P* values were calculated by one-way ANOVA. Source data are available online for this figure.

Mitochondrial and cytosolic fractions were purified from cells either co-expressing NSP3 and NSP4 or infected with coronavirus and analyzed by mass spectrometry (MS). Thirteen mitochondrial proteins were found to translocate from mitochondria to the cytosol in both the NSP3 and NSP4 co-expression and coronavirus infection models (Fig. EV6A). We then performed a small-scale RNAi screen targeting these translocated candidates, which showed that knockdown of *ECHS1* (Enoyl-CoA Hydratase, Short Chain 1), a mitochondrial matrix enzyme involved in fatty acid metabolism, significantly reduced the fluorescence intensity of GFP-NSP3-T2A-NSP4 (Fig. EV6B,C). In contrast, overexpression of *ECHS1* increased the fluorescence signal of GFP-NSP3-T2A-NSP4 (Fig. EV6D). Collectively, these results indicate that ECHS1 might play a critical role in DMV formation. Subsequent immunoblotting analysis confirmed increased levels of ECHS1 in the cytosol relative to those in mitochondria in both cells co-expressing NSP3 and NSP4 and coronavirus-infected cells (Fig. EV6E–H). Moreover, overexpression of other viral proteins known to affect mitochondrial structure and function (SARS-CoV-2 ORF3a, NSP8, N, or M) (Ramachandran et al, 2022; Yang et al, 2022; Zong et al, 2023; Li et al, 2024) did not alter the subcellular localization of ECHS1 (Fig. EV6I,J). This demonstrates that the translocation of ECHS1 is specifically triggered by DMV formation.

**Figure 5:**
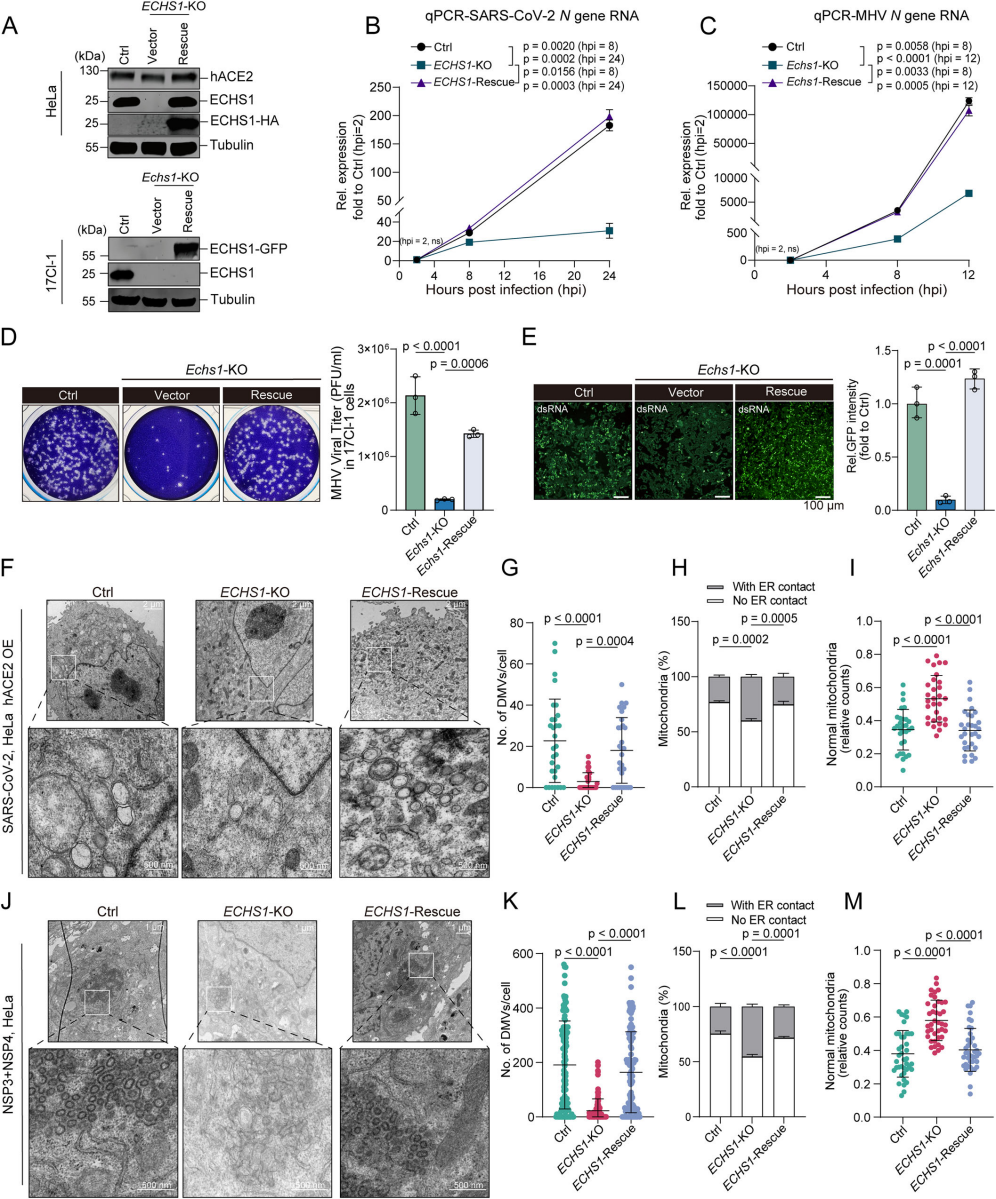
ECHS1 released from damaged mitochondria is essential for DMV formation and coronavirus infection. (**A**) Immunoblotting analysis of ECHS1 expression in *ECHS1*-KO and *ECHS1*-Rescued HeLa cells stably expressing hACE2, and in *Echs1*-KO and *Echs1*-Rescued 17Cl-1 cells. (**B**) qPCR analysis of viral gRNA levels in *ECHS1*-KO and *ECHS1*-Rescued HeLa cells stably expressing hACE2 infected with SARS-CoV-2 (MOI = 1, hpi = 2, 8, 24). Data are presented as mean ± SD, with *n* = 3 independent experiments. *P* values (calculated by two-way ANOVA) are indicated: *P* = 0.0020 (8 hpi), *P* = 0.0002 (24 hpi) for Ctrl vs *ECHS1*-KO; *P* = 0.0156 (8 hpi), *P* = 0.0003 (24 hpi) for *ECHS1*-Rescue vs *ECHS1*-KO; no significant differences at 2 hpi were observed between all groups. (**C**) qPCR analysis of viral gRNA levels in *Echs1*-KO and *Echs1*-Rescued 17Cl-1 cells infected with MHV (MOI = 5, hpi = 2, 8, 12). Data are presented as mean ± SD, with *n* = 3 independent experiments. *P* values (calculated by two-way ANOVA) are indicated: *P* = 0.0058 (8 hpi), *P* < 0.0001 (12 hpi) for Ctrl vs *Echs1*-KO; *P* = 0.0033 (8 hpi), *P* = 0.0005 (12 hpi) for *Echs1*-Rescue vs *Echs1*-KO; no significant differences at 2 hpi were observed between all groups. (**D**) Plaque assay and quantification of extracellular viral titer in *Echs1*-KO and *Echs1*-Rescued 17Cl-1 cells infected by MHV (MOI = 2, hpi = 16). Representative plaque images are shown in the left panel. Quantification of MHV viral titers (PFU/mL) is shown in the right panel. Data are presented as mean ± SD (*n* = 3 independent experimental replicates); *P* values were calculated by one-way ANOVA. (**E**) Fluorescence imaging and quantification of double-stranded RNA (dsRNA) in *Echs1*-KO and *Echs1*-Rescued 17Cl-1 cells infected by MHV (MOI = 2, hpi = 8). Representative fluorescence images of dsRNA are shown in the left panel; scale bar, 100 μm. Quantification of relative GFP intensity is shown in the right panel. Data are presented as mean ± SD (*n* = 3 independent experimental replicates); *P* values were calculated by one-way ANOVA. (**F**) Representative TEM images of DMV formation in *ECHS1*-KO and *ECHS1*-Rescued HeLa cells stably expressing hACE2 infected with SARS-CoV-2 (MOI = 1, hpi = 8). Scale bars, 2 μm (main images); 500 nm (zoomed-in views). Zoomed-in views highlight DMV structures. (**G**) Quantitative analysis of DMV numbers per cell from TEM images in (**F**). Data are presented as mean ± SD (*n* > 30 cells per group); *P* values were calculated by one-way ANOVA. (**H**) Quantification of the proportion of mitochondria with or without ER contact from TEM images in (**F**). Data are presented as mean ± SD (*n* > 30 cells per group); *P* values were calculated by one-way ANOVA. (**I**) Quantification of the proportion of normal mitochondria relative to total mitochondria from TEM images in (**F**). Data are presented as mean ± SD (*n* > 30 cells per group); *P* values were calculated by one-way ANOVA. (**J**) Representative TEM images of DMV formation induced by co-expression of NSP3 and NSP4 in *ECHS1*-KO and *ECHS1*-Rescued HeLa cells. Scale bars, 1 μm (main images); 500 nm (zoomed-in views). Zoomed-in views highlight DMV structures. (**K**) Quantitative analysis of DMV numbers per cell from TEM images in (**J**). Data are presented as mean ± SD (*n* > 80 cells per group); *P* values were calculated by one-way ANOVA. (**L**) Quantification of the proportion of mitochondria with or without ER contact from TEM images in (**J**). Data are presented as mean ± SD (*n* > 40 cells per group); *P* values were calculated by one-way ANOVA. (**M**) Quantification of the proportion of normal mitochondria relative to total mitochondria from TEM images in (**J**). Data are presented as mean ± SD (*n* > 40 cells per group); *P* values were calculated by one-way ANOVA. Source data are available online for this figure.

**Figure EV7:**
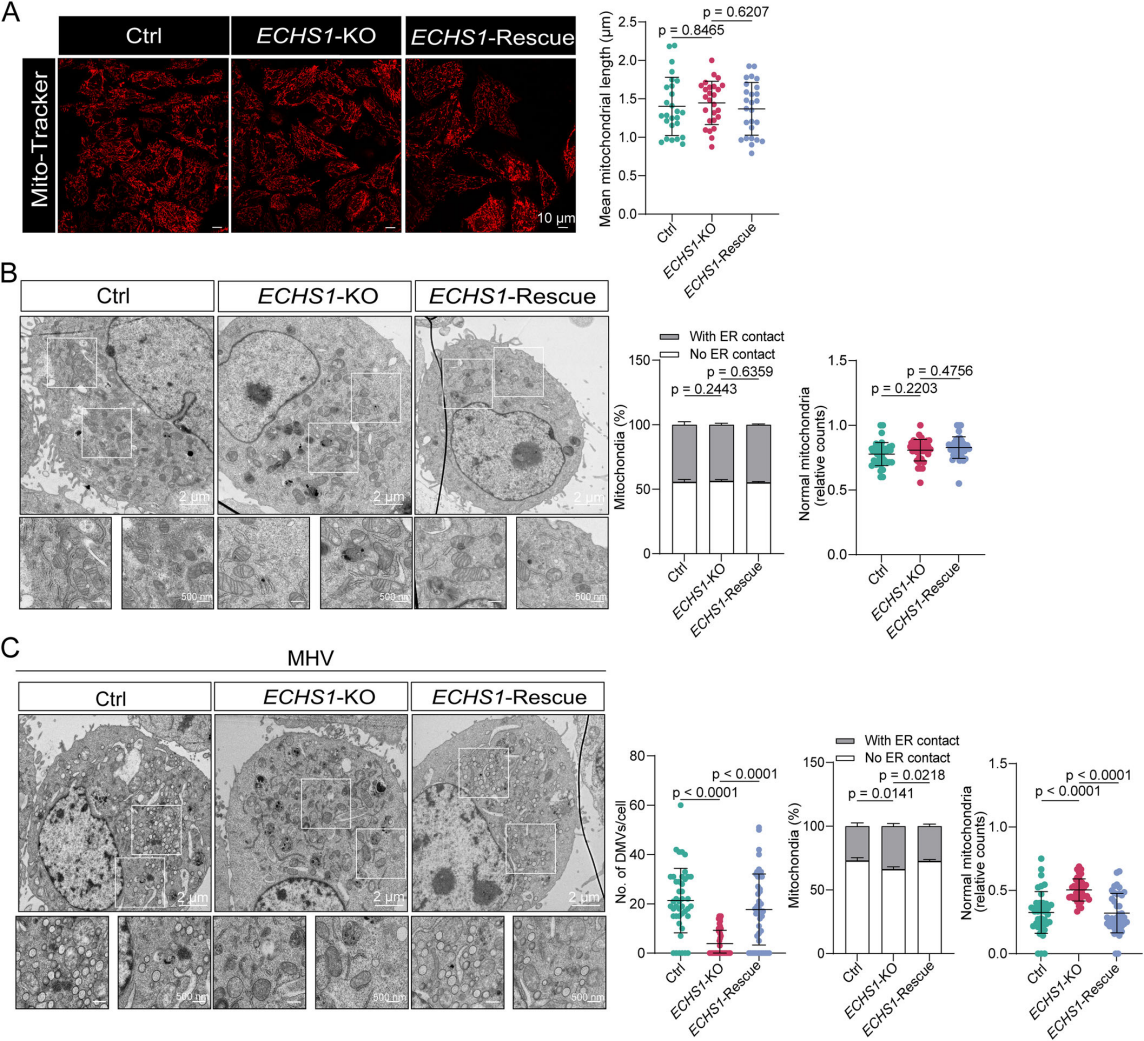
ECHS1 deficiency protects against mitochondrial morphological abnormalities and ERMCSs loss during MHV infection. (**A**) Representative fluorescence images of mitochondria (stained with Mito-Tracker Red) in Ctrl, *ECHS1*-KO, and *ECHS1*-Rescue HeLa cells under uninfected conditions. Scale bar, 10 μm. The right panel shows quantification of mean mitochondrial length, which was determined using the Mitochondrial Analyzer plugin in ImageJ with 2D threshold. Data are presented as mean ± SD; *P* values were calculated by one-way ANOVA. (**B**) Representative TEM images of Ctrl, *ECHS1*-KO, and *ECHS1*-Rescue HeLa cells under uninfected conditions, showing mitochondrial morphology and ERMCSs. Scale bars, 2 μm (main images) and 500 nm (zoomed-in views). Data are presented as mean ± SD; *P* values were calculated by one-way ANOVA. (**C**) Representative TEM images of Ctrl, *ECHS1*-KO, and *ECHS1*-Rescue 17Cl-1 cells infected with MHV (MOI = 1, hpi = 8), showing mitochondrial morphology, ERMCSs, and DMV formation. Scale bars, 2 μm (main images) and 500 nm (zoomed-in views). Data are presented as mean ± SD (*n* > 30 cells per group). *P* values were calculated by one-way ANOVA. Source data are available online for this figure.

To investigate the role of ECHS1 in coronavirus infection and DMV formation, *ECHS1* knockout (KO) HeLa-human angiotensin-converting enzyme 2 (hACE2) and 17Cl-1 cells generated using CRISPR-Cas9 were infected with SARS-CoV-2 and MHV, respectively (Fig. 5A). Time-course qPCR revealed no significant differences in viral gRNA levels among the cell lines at 2 hpi, indicating ECHS1 deficiency does not affect viral entry. However, viral gRNA levels were markedly reduced in *ECHS1*-KO cells at later infection time points: at 8 hpi and 24 hpi for SARS-CoV-2, as well as 8 hpi and 12 hpi for MHV. This suppression was reversed in *ECHS1*-rescued cells (Fig. 5B,C). Similarly, infectious virus titers and intracellular double-stranded RNA (dsRNA) levels, hallmarks of active viral replication, were drastically diminished in *Echs1*-KO cells and restored upon *Echs1* rescue, indicating that ECHS1 plays a critical role in coronavirus infection (Fig. 5D,E). Furthermore, TEM revealed infection with both SARS-CoV-2 and MHV significantly decreased DMV abundance in *ECHS1-*KO cells, with restoration in *ECHS1-*rescued cells (Figs. 5F,G and EV7C). Consistent results were obtained in the NSP3 and NSP4 co-expression system, demonstrating that ECHS1 is required for efficient viral replication and essential for DMV formation (Fig. 5J, K). Additionally, we assessed ERMCSs and mitochondrial morphology in these cell lines. Under normal conditions, *ECHS1* KO or rescue had no effect on ERMCSs and mitochondrial morphology (Fig. EV7A,B). However, upon viral infection or co-expression of NSP3 and NSP4, *ECHS1*-KO cells exhibited preserved ERMCSs and an intact mitochondrial phenotype compared to control cells, which correlated with the reduced DMV numbers observed in these cells (Figs. 5H,I,L,M and EV7C). Together, these results indicate that ECHS1 released from damaged mitochondria is essential for DMV formation and coronavirus infection.

### ECHS1 stabilizes NSP3 by blocking its ubiquitination at K963

**Figure EV8:**
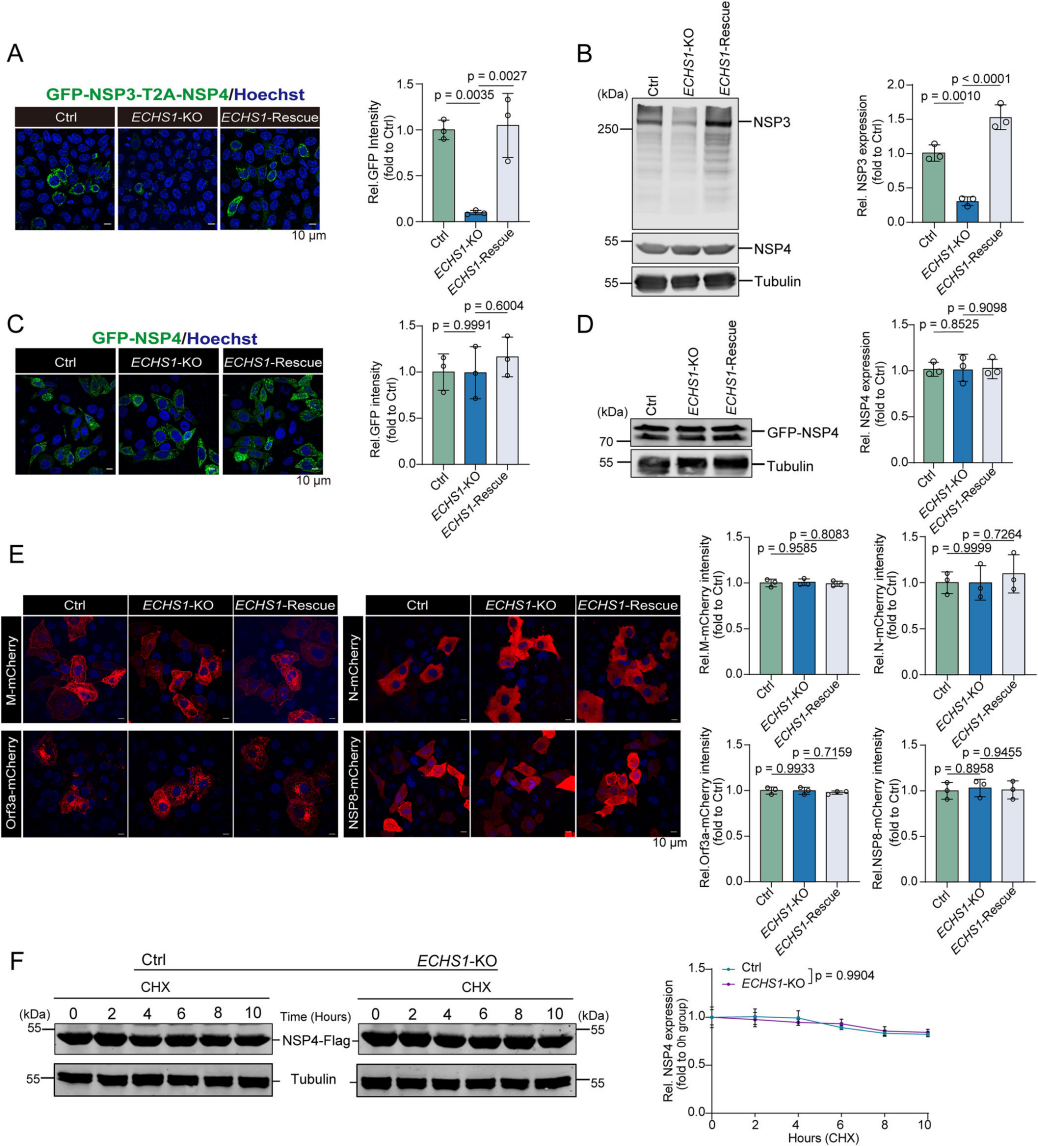
ECHS1 specifically regulates the expression of NSP3 without affecting the levels of other viral proteins. (**A**) Representative immunofluorescence images showing the expression of GFP-NSP3-T2A-NSP4 in Ctrl, *ECHS1*-KO, and *ECHS1*-Rescue HeLa cells. Scale bar, 10 μm. The right panel presents quantification of relative GFP fluorescence intensity. Data are presented as mean ± SD (*n* = 3 independent experimental replicates); *P* values were calculated by one-way ANOVA. (**B**) Immunoblotting analysis of NSP3 and NSP4 protein levels in Ctrl, *ECHS1*-KO, and *ECHS1*-Rescue HeLa cells. Data are presented as mean ± SD (*n* = 3 independent experimental replicates); *P* values were calculated by one-way ANOVA. (**C**) Representative immunofluorescence images showing the expression of GFP-NSP4 in Ctrl, *ECHS1*-KO, and *ECHS1*-Rescue HeLa cells. Scale bar, 10 μm. Data are presented as mean ± SD (*n* = 3 independent experimental replicates); *P* values were calculated by one-way ANOVA. (**D**) Immunoblotting analysis of GFP-NSP4 protein levels in Ctrl, *ECHS1*-KO, and *ECHS1*-Rescue HeLa cells. Data are presented as mean ± SD (*n* = 3 independent experimental replicates); *P* values were calculated by one-way ANOVA. (**E**) Representative immunofluorescence images showing the expression of mCherry-tagged viral proteins (M, N, ORF3a, NSP8) in Ctrl, *ECHS1*-KO, and *ECHS1*-Rescue HeLa cells. Scale bar, 10 μm. The right panels present quantification of relative mCherry fluorescence intensity for each viral protein. Data are presented as mean ± SD (*n* = 3 independent experimental replicates); *P* values were calculated by one-way ANOVA. (**F**) Immunoblotting analysis of GFP-NSP4 stability in HeLa Ctrl and *ECHS1*-KO cells treated with CHX for 0–10 h. The right panel shows quantification of relative GFP-NSP4 protein levels over time. Data are presented as mean ± SD (*n* = 3 independent experimental replicates). *P* values for the main effect of group (Ctrl vs *ECHS1*-KO) were calculated by two-way ANOVA. Source data are available online for this figure.

**Figure 6:**
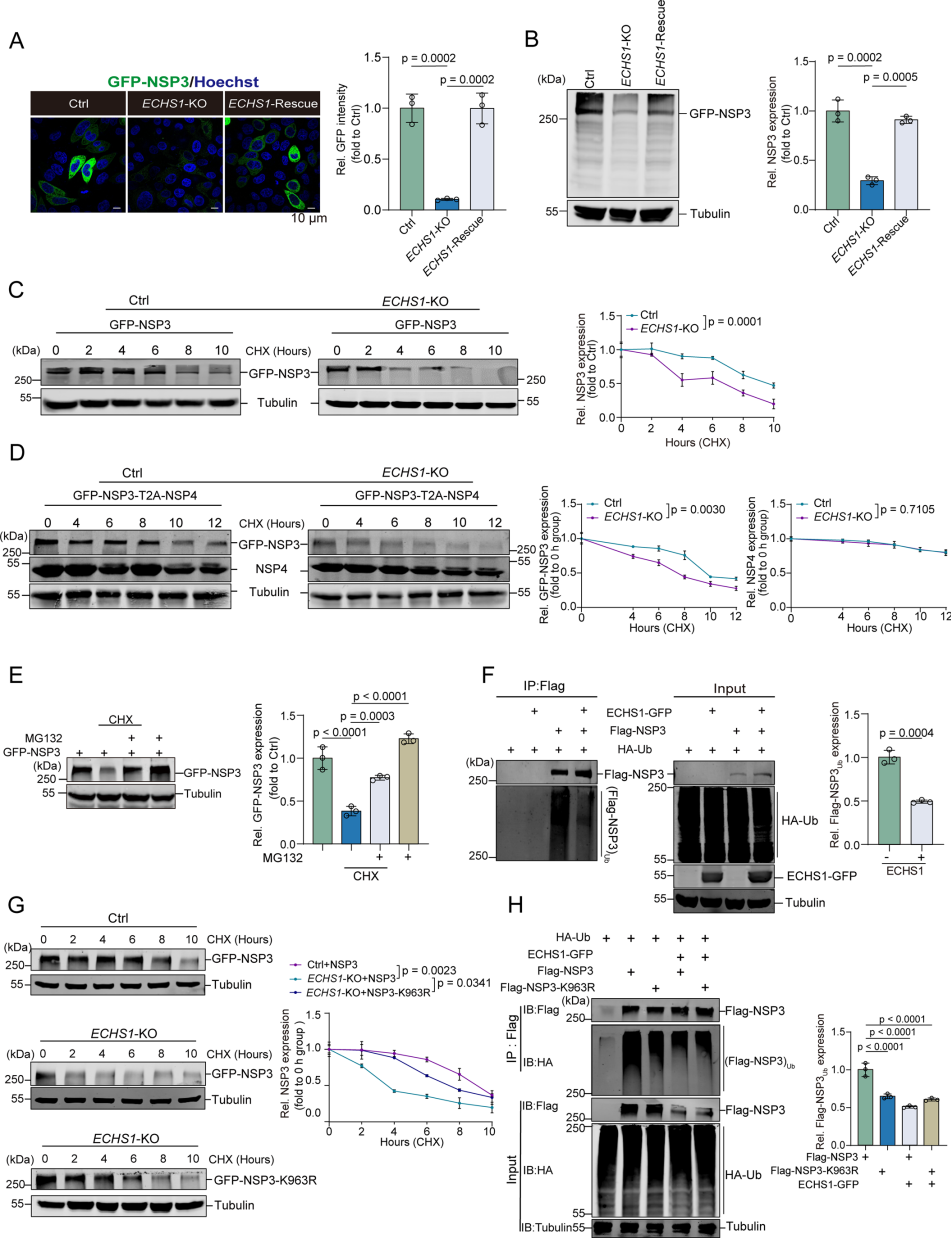
ECHS1 stabilizes NSP3 by blocking its ubiquitination at K963 site. (**A**) Representative immunofluorescence images showing GFP-NSP3 expression in Ctrl, *ECHS1*-KO, and *ECHS1*-Rescued HeLa cells; scale bar, 10 μm. The right panel quantifies GFP-NSP3 fluorescence intensity. Data are presented as mean ± SD (*n* = 3 independent experimental replicates); *P* values were calculated by one-way ANOVA. (**B**) Immunoblotting analysis of GFP-NSP3 protein levels in *ECHS1*-KO and *ECHS1*-Rescued HeLa cells. Data are presented as mean ± SD (*n* = 3 independent experimental replicates); *P* values were calculated by one-way ANOVA. (**C**) Immunoblotting analysis of GFP-NSP3 stability in Ctrl and *ECHS1*-KO HeLa cells treated with cycloheximide (CHX) for 0–10 h. The right panel quantifies relative GFP-NSP3 protein levels over time. Data are presented as mean ± SD (*n* = 3 independent experimental replicates); the *P* value for the main effect of group (Ctrl vs *ECHS1*-KO, *P* = 0.0001) was calculated by two-way ANOVA. (**D**) Immunoblotting analysis of GFP-NSP3 and NSP4 stability in Ctrl and *ECHS1*-KO HeLa cells co-expressing GFP-NSP3-T2A-NSP4, treated with CHX for 0–12 h. The right panels quantify relative GFP-NSP3 and NSP4 protein levels over time. Data are presented as mean ± SD (*n* = 3 independent experimental replicates); *P* values for the main effect of group (Ctrl vs *ECHS1*-KO on GFP-NSP3, *P* = 0.0030; Ctrl vs *ECHS1*-KO on NSP4, *P* = 0.7105) were calculated by two-way ANOVA. (**E**) Immunoblotting analysis of GFP-NSP3 protein stability in cells transfected with GFP-NSP3, following 10 h treatment with CHX alone or in combination with the proteasome inhibitor MG132. The right panel quantifies relative GFP-NSP3 expression. Data are presented as mean ± SD (*n* = 3 independent biological replicates); *P* values were calculated by one-way ANOVA. (**F**) Immunoblotting analysis demonstrating that ECHS1 reduces the ubiquitination level of NSP3. HeLa cells were co-transfected with HA-Ub (HA-tagged ubiquitin), Flag-NSP3, and ECHS1-GFP or empty vector. Flag-NSP3 was immunoprecipitated using a Flag antibody, and its ubiquitination level was detected by immunoblotting with HA antibody (Flag-NSP3_Ub_, ubiquitinated Flag-NSP3). Input controls confirm the expression of Flag-NSP3, ECHS1-GFP, HA-Ub, and Tubulin (loading control). The right panel presents quantitative analysis of relative Flag-NSP3 ubiquitination levels. Data are presented as mean ± SD (*n* = 3 independent experimental replicates); *P* value was calculated by unpaired *t* test. (**G**) Immunoblotting analysis assessing the stability WT NSP3 and NSP3-K963R (K963R mutant) in Ctrl and *ECHS1*-KO HeLa cells following CHX treatment. The right panel quantifies relative NSP3 protein levels over time. Data are presented as mean ± SD (*n* = 3 independent experimental replicates); *P* values for the main effect of group (Ctrl + NSP3 vs. *ECHS1*-KO + NSP3, *P* = 0.0023; *ECHS1*-KO + NSP3 vs. *ECHS1*-KO + NSP3-K963R, *P* = 0.0341) were calculated by two-way ANOVA. (**H**) Immunoblotting analysis of the ubiquitination level of WT or K963R mutant NSP3 in the Ctrl or ECHS1 overexpressing HeLa cells. HeLa cells were transfected with HA-Ub, along with Flag-NSP3 (WT) or Flag-NSP3-K963R, and either ECHS1-GFP or vector control. Flag-NSP3 was immunoprecipitated using a Flag antibody, and its ubiquitination level was detected by immunoblotting with HA antibody (Flag-NSP3_Ub_). Input controls confirm the expression of Flag-NSP3, Flag-NSP3-K963R, ECHS1-GFP, HA-Ub, and Tubulin. The right panel presents quantitative analysis of relative Flag-NSP3 ubiquitination levels. Data are presented as mean ± SD (*n* = 3 independent experimental replicates). *P* values were calculated by one-way ANOVA. Source data are available online for this figure.

We next examined how ECHS1 regulates DMV formation. Previous screening revealed that ECHS1 could regulate the expression of GFP-NSP3-T2A-NSP4 (Fig. EV6C). Consistently, GFP fluorescence was markedly reduced in *ECHS1*-KO cells expressing GFP-NSP3-T2A-NSP4 but restored in *ECHS1*-rescued cells. Immunoblotting confirmed that NSP3 expression was selectively decreased in *ECHS1*-KO cells, while NSP4 remained unaffected. This reduction was reversed in *ECHS1*-rescued cells (Fig. EV8A,B). Critically, both immunofluorescence and immunoblotting analyses confirmed that only NSP3 expression, not that of NSP4, was reduced in *ECHS1*-KO cells expressing NSP3 or NSP4 alone, demonstrating that ECHS1 specifically regulates NSP3 expression (Figs. 6A,B and EV8C,D). Notably, this effect was specific to NSP3, as no significant fluorescence differences were observed for other viral proteins (M, N, ORF3a, NSP8) linked to mitochondrial structure and function in *ECHS1*-KO cells (Fig. EV8E).

**Figure EV9:**
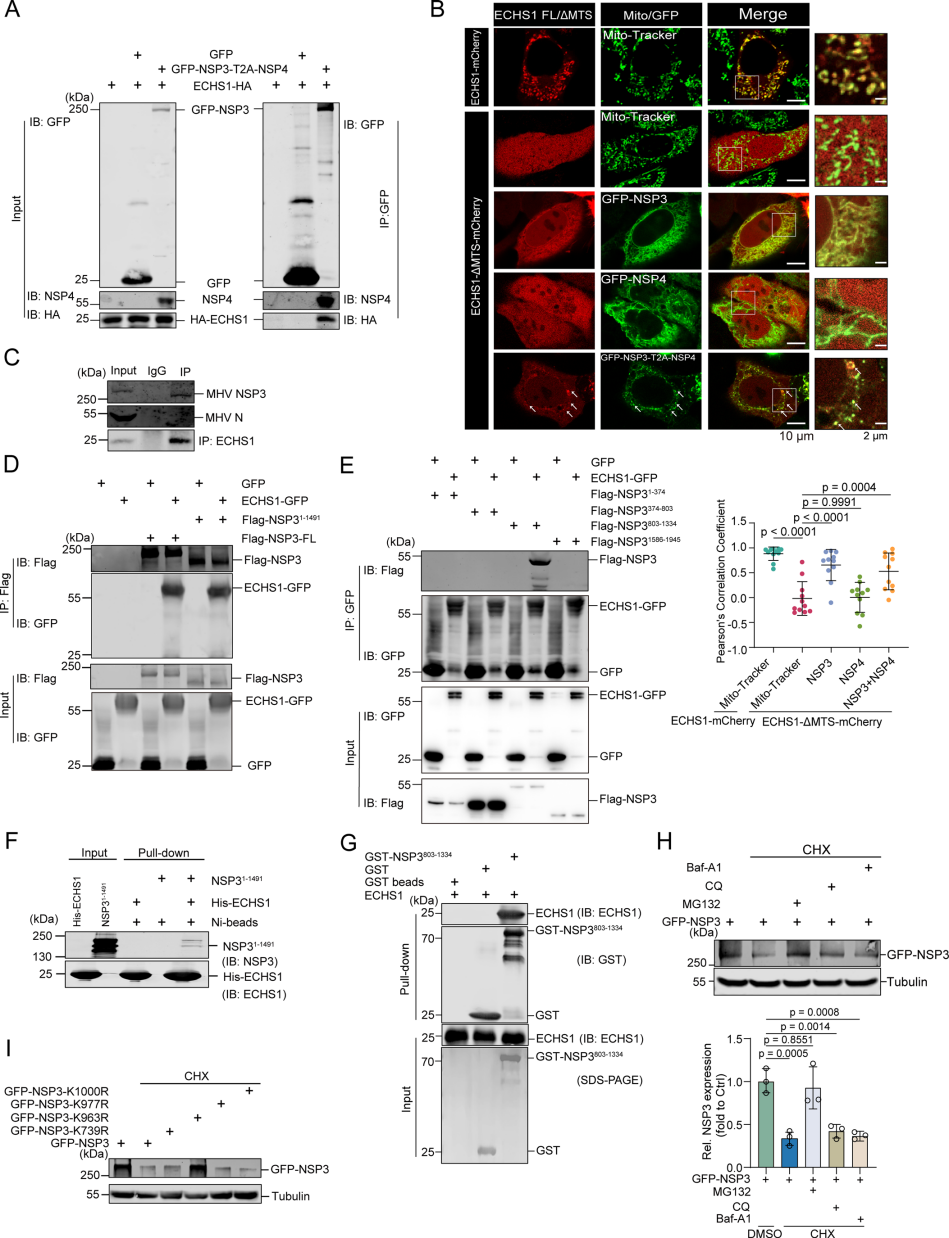
ECHS1 directly interacts with NSP3^803-1334^ domain. (**A**) Co-IP analysis of the interaction between ECHS1-HA and GFP-NSP3-T2A-NSP4 in 293T cells. Cell lysates were immunoprecipitated with GFP antibody (IP: GFP), followed by immunoblotting with GFP, NSP4 or HA antibodies. (**B**) Representative fluorescence images showing the subcellular localization of ECHS1-mCherry (full-length, FL) and ECHS1-ΔMTS-mCherry (mitochondrial targeting sequence, MTS, deleted) in HeLa cells. Scale bars, 10 μm (main images) and 2 μm (zoomed-in views). The lower panel quantifies colocalization using Pearson’s correlation coefficient. Data are presented as mean ± SD (*n* = 10 independent fields of view per group); *P* values were calculated by one-way ANOVA. (**C**) Endogenous co-IP analysis of the interaction between ECHS1 and MHV NSP3 in MHV-infected 17Cl-1 cells (MOI = 10, hpi = 8). Cell lysates were immunoprecipitated with an ECHS1 antibody (IP: ECHS1) or IgG control, followed by immunoblotting with MHV NSP3 and ECHS1 antibodies. (**D**) Co-IP analysis of the interaction between ECHS1-GFP and Flag-NSP3 truncation mutants (full-length Flag-NSP3-FL and truncated Flag-NSP3^1-1491^) in 293 T cells. Cell lysates were immunoprecipitated with Flag antibody (IP: Flag), followed by immunoblotting with Flag and GFP antibodies. (**E**) Co-IP analysis of the interaction between ECHS1-GFP and Flag-NSP3 variants (residues 1–374, 374–803, 803–1334, 1586–1945) in 293T cells. Cell lysates were immunoprecipitated with GFP antibody (IP: GFP), followed by immunoblotting with Flag or GFP antibodies. (**F**, **G**) In vitro binding assay analysis of the direct interaction between ECHS1 and NSP3^1-1491^ or NSP3^803-1334^. Samples were analyzed by SDS-PAGE and immunoblotting with NSP3, ECHS1 or GST antibodies, with *n* = 3 independent biological replicates. (**H**) Cells were transfected with GFP-NSP3 and treated with the proteasome inhibitor MG132, lysosomal inhibitors chloroquine (CQ) or bafilomycin A1 (Baf-A1), with or without CHX, for 10 h. The lower panel quantifies relative GFP-NSP3 expression. Data are presented as mean ± SD (*n* = 3 independent biological replicates); *P* values were calculated by one-way ANOVA. (**I**) Immunoblotting analysis of GFP-NSP3 stability (WT and K1000R/K977R/K963R/K739R ubiquitination site mutants) in 293T cells treated with cycloheximide (CHX) for 10 h. Source data are available online for this figure.

Given prior reports that ECHS1 stabilizes client proteins (Burgin et al, 2023; Xiao et al, 2013; Cai et al, 2022), we investigated whether it stabilizes NSP3. Protein degradation assays in cells expressing either GFP-NSP3 alone or GFP-NSP3-T2A-NSP4 demonstrated accelerated decay of NSP3 in *ECHS1*-KO cells, while NSP4 expression remained unchanged, confirming our earlier observation of reduced NSP3 levels (Figs. 6C,D and EV8F). Furthermore, co-immunoprecipitation (co-IP) assays demonstrated that ECHS1 was specifically co-precipitated by NSP3, indicating a direct and selective interaction with NSP3 (Fig. EV9A). Immunofluorescence assays showed that ECHS1 localizes primarily to mitochondria. Deletion of its mitochondrial targeting sequence (MTS, ECHS1-ΔMTS) retained the protein exclusively in the cytoplasm, abolishing its mitochondrial localization. When co-expressed with NSP3 or NSP4, ECHS1-ΔMTS exhibited significant colocalization with NSP3, but not NSP4, and was recruited to punctate DMVs formed by NSP3 and NSP4 co-expression. These results demonstrate that ECHS1 is recruited to viral replication complexes via interaction with NSP3 (Fig. EV9B). To confirm this interaction, we performed endogenous co-IP in MHV-infected cells, revealing that ECHS1 specifically precipitated MHV NSP3, validating their interaction (Fig. EV9C). To define the interaction interface, we generated a series of truncated NSP3 variants. Co-IP results identified the N-terminal region (residues 803–1334) of NSP3 as sufficient for precipitation by ECHS1 (Fig. EV9D,E). Moreover, in vitro binding assays confirmed that this segment (residues 803–1334) directly interacts with ECHS1, demonstrating that NSP3 binds to ECHS1 through this domain (Fig. EV9F,G).

We then explored how ECHS1 stabilizes NSP3. The proteasome inhibitor MG132, and the lysosomal inhibitors Chloroquine (CQ) and Bafilomycin A1 (Baf-A1) were used to explore the degradation pathway of NSP3. Treatment with the proteasome inhibitor MG132, but not the lysosome inhibitors CQ or Baf-A1, could significantly restore the cycloheximide (CHX)-induced NSP3 downregulation (Fig. EV9H) and MG132 alone also elevated basal GFP-NSP3 expression (Fig. 6E). Consistently, the ubiquitination of NSP3 was significantly decreased in the *ECHS1*-overexpressing cells, indicating that ECHS1 regulates the ubiquitination-dependent proteasomal degradation of NSP3 (Fig. 6F).

To further identify the exact ubiquitination sites on NSP3, we conducted an unbiased IP coupled with MS (IP-MS) analysis. The MS results revealed four potential ubiquitination sites in NSP3 (K739, K963, K977, and K1000). We then introduced point mutations at each site to determine the major ubiquitination residue responsible for regulating NSP3 stability. Among these, only the K963R mutation restored NSP3 protein levels following CHX-induced degradation, whereas the K739R, K977R, and K1000R mutations did not (Fig. EV9I). Consistently, degradation assays showed that the K963R mutant exhibited a longer half-life than wild-type (WT) NSP3 in *ECHS1*-KO cells. Moreover, ubiquitination assays demonstrated that the K963R mutation markedly reduced NSP3 polyubiquitination. Importantly, overexpression of *ECHS1* failed to further decrease the already reduced polyubiquitination of the K963R mutant (Fig. 6G,H). Together, these results indicate that ECHS1 interacts with NSP3 and stabilizes it by inhibiting its polyubiquitination at the K963 site.

### E3 ubiquitin ligase RBBP6 mediates the NSP3 polyubiquitination and restricts coronavirus replication

**Figure 7:**
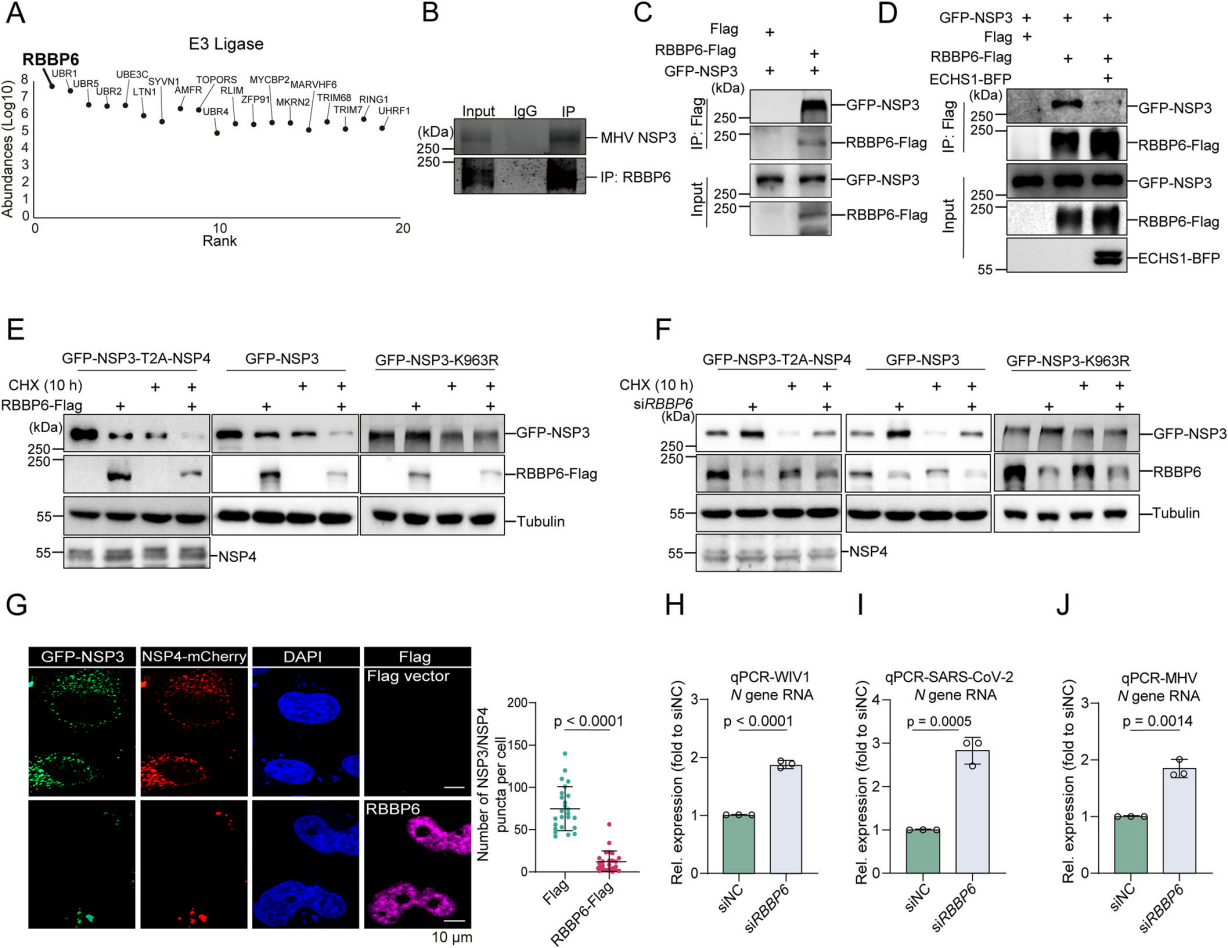
E3 ubiquitin ligase RBBP6 mediates the NSP3 polyubiquitination and restricts coronavirus replication. (**A**) Abundance ranking plot (log_10_ scale) of E3 ubiquitin ligases identified by MS from GFP bead-based IP of GFP-NSP3 transfected into HeLa cells. (**B**) Endogenous co-IP analysis of the interaction between RBBP6 and MHV NSP3 in MHV-infected 17Cl-1 cells (MOI = 10, hpi = 8). Cell lysates were immunoprecipitated with a RBBP6 antibody (IP: RBBP6) or IgG control, followed by immunoblotting with MHV NSP3 and RBBP6 antibodies. (**C**) Co-IP analysis of the interaction between RBBP6-Flag and GFP-NSP3. 293T cells lysates expressing GFP-NSP3 with Flag vector control or RBBP6-Flag were immunoprecipitated using a Flag antibody, followed by immunoblotting with GFP or Flag antibodies. (**D**) Co-IP analysis of the interaction between RBBP6-Flag and GFP-NSP3 upon *ECHS1* overexpression. 293T cells were transfected with plasmids encoding GFP-NSP3, RBBP6-FLAG, and ECHS1-BFP at a ratio of 2:1.5:1. Transfection efficiency analysis confirmed comparable positive rates, with ~18–20% of cells co-expressing all three proteins, ~20% double-positive for GFP-NSP3 and RBBP6-FLAG, and ~20% double-positive for GFP-NSP3 and ECHS1-BFP. Cell lysates co-expressing GFP-NSP3 and RBBP6-Flag with or without ECHS1-BFP were immunoprecipitated using a Flag antibody, followed by immunoblotting with GFP or Flag antibodies. (**E**) 293 T cells transfected with GFP-NSP3-T2A-NSP4, GFP-NSP3, or GFP-NSP3-K963R were subjected to RBBP6 overexpression or vector control treatment, followed by CHX treatment (10 h). (**F**) 293T cells were transfected with constructs encoding GFP-NSP3-T2A-NSP4, GFP-NSP3, or GFP-NSP3-K963R followed by RBBP6 knockdown or vector control treatment, followed by CHX treatment (10 h). (**G**) Immunofluorescence analysis of the effect of RBBP6 on NSP3/NSP4 puncta formation in HeLa cells. Scale bar = 10 μm. The right panel quantifies puncta number. Data are presented as mean ± SD (*n* = 25 cells per group); *P* values were calculated by unpaired *t* test. (**H**–**J**) qPCR analysis of viral gRNA levels in the siNC and *RBBP6* knockdown 293 T cells transiently transfected with hACE2 and then infected with WIV1 (MOI = 1, hpi = 24, (**H**)) and SARS-CoV-2 (MOI = 1, hpi = 24, (**I**)), or in siNC and *RBBP6* knockdown 17Cl-1 cells infected with MHV (MOI = 1, hpi = 24, (**J**)). Viral gRNA levels were normalized to those in siNC-treated cells. Data are presented as mean ± SD (*n* = 3 independent experimental replicates). *P* values were calculated by unpaired *t* test. Source data are available online for this figure.

To identify the E3 ubiquitin ligase responsible for NSP3 ubiquitination, we immunoprecipitated GFP-NSP3 from HeLa cells and analyzed the precipitates by MS. Among all the candidate E3 ligases identified, RBBP6 was ranked as the most abundant interacting partner (Fig. 7A). We validated this interaction under physiological viral infection conditions, confirming that endogenous RBBP6 interacts with NSP3 in MHV-infected 17 Cl-1 cells (Fig. 7B). Additional co-IP assays in an overexpression system further confirmed the interaction (Fig. 7C). Interestingly, overexpression of *ECHS1* impaired this interaction, suggesting that ECHS1 stabilizes NSP3 by shielding it from RBBP6-mediated ubiquitination (Fig. 7D). As expected, when NSP3 was expressed alone or co-expressed with NSP4, overexpression of RBBP6 significantly reduced NSP3 protein levels in both control and CHX-treated cells, whereas RBBP6 knockdown increased NSP3 expression under the same conditions. Furthermore, the stability of the NSP3 K963R mutant remained unaffected by either *RBBP6* overexpression or knockdown, strongly supporting K963 as the primary ubiquitination site targeted by RBBP6. (Fig. 7E,F). Functionally, *RBBP6* overexpression impaired the formation of NSP3/NSP4 puncta, which are critical for viral replication (Fig. 7G). Correspondingly, *RBBP6* knockdown enhanced several coronaviruses infection, including SARS-CoV-2, MHV, and WIV1 (Fig. 7H–J). Taken together, these findings demonstrate that the E3 ubiquitin ligase RBBP6 mediates polyubiquitination of NSP3 and thereby restricts coronavirus infection.

## Discussion

Biogenesis of viral replication organelles is a defining hallmark of positive-sense RNA virus infection. While the ER-derived nature of coronavirus DMVs is well-established, our understanding of their functional integration into the broader inter-organellar landscape of the host cell has remained scanty. Here, we elucidate a self-reinforcing cycle that transcends the conventional view of static viral factories. We demonstrate that DMV formation impairs mitochondria via disruption of ERMCSs, and in turn, damaged mitochondria release ECHS1 to stabilize NSP3, thereby promoting further DMV formation. This self-reinforcing cycle is thus essential for efficient coronavirus infection.

During the early stages of coronavirus infection, mitochondria have been reported to undergo horizontal thinning and longitudinal elongation, accompanied by increased matrix density (Shang et al, 2022), a phenomenon also observed in our TEM analyses. Beyond these previously described changes, our study uncovered additional mitochondrial abnormalities, including cristae disruption, blurred outer membrane boundaries, and matrix vacuolization. We further demonstrated that the formation of DMVs by NSP3 and NSP4 contributes to these mitochondrial alterations by reducing ERMCSs, which are known to regulate mitochondrial morphology and function (Krols et al, 2016; Csordás et al, 2018; Rowland and Voeltz, 2012). Over the past few decades, several protein complexes bridging the ER and mitochondria have been identified, including VAPB-RMDN3 (PTPIP51), MFN2-MFN1/2, IP3R-VDAC1, and BAP31-FIS1(Csordás et al, 2018; De Brito and Scorrano, 2008; De vos et al, 2012). In this study, we demonstrate that DMV formation significantly decreases the expression of MFN2 and RMDN3. Ectopic expression of either MFN2 or RMDN3 restored ERMCSs, mitochondrial morphology, and functionality. Interestingly, this also reduced DMV abundance and suppressed viral infection. Recent studies have shown that infection with coronavirus HCoV-OC43 leads to marked reductions in nearly all ERMCSs proteins, correlating with disrupted ER and mitochondrial network architecture and impaired inter-organellar communication (Cook et al, 2022). Similar ERMCSs disruption has been reported during infection with hepatitis C virus, influenza virus, and flaviviruses (Freppel et al, 2025; Pila-Castellanos et al, 2021; Cook et al, 2022). Together, these findings suggest that ERMCSs are a common target during viral infection, and that enhancing ERMCSs formation may represent a broadly applicable antiviral strategy. However, the precise mechanism underlying the transcriptional downregulation of ERMCS components remains unclear. Previous work suggests that early viral gene products are responsible for suppressing the expression of ERMCS proteins during infection (Cook et al, 2022). Our results advance this understanding by demonstrating that the viral proteins NSP3 and NSP4 act as direct transcriptional repressors of ERMCS components. Thus, future work to identify the intermediary transcriptional or post-transcriptional regulators that mediate the effect of NSP3 and NSP4 co-expression on ERMCSs may reveal promising broad-spectrum antiviral targets.

Mitochondrial morphology plays a central role in regulating innate immune responses, and numerous viruses have been reported to disrupt mitochondrial architecture to evade host immunity (Pila-Castellanos et al, 2021; Chatel-Chaix et al, 2016). Beyond its established immunoregulatory functions, we have identified a novel role for mitochondria in coronavirus infection: the release of the mitochondrial matrix protein ECHS1, which stabilizes viral NSP3 by blocking its RBBP6-mediated polyubiquitination. Both ECHS1 and RBBP6 were shown to modulate coronavirus infection.

ECHS1 is a key enzyme in mitochondrial fatty acid β-oxidation, which is essential for converting long-chain fatty acids into acetyl-CoA (Burgin et al, 2023). However, previous studies have shown that fatty acid β-oxidation is dispensable for SARS-CoV-2 replication, as inhibitors such as etomoxir (targeting CPT1A) and trimetazidine (inhibiting long-chain 3-ketoacyl-CoA thiolase) fail to suppress viral infection (Williams et al, 2021). In contrast, fatty acid synthesis is known to be required for coronavirus replication (Chu et al, 2021). Thus, we speculate that ECHS1 regulates infection through a mechanism independent of its role in fatty acid β-oxidation.

It has been reported that ECHS1 can translocate between mitochondria and the cytosol depending on nutrient conditions (Zhang et al, 2017). In this study, we demonstrate that coronavirus infection, or DMV biogenesis, induces the translocation of ECHS1 from mitochondria to the cytosol. Subsequently, ECHS1 directly interacts with NSP3, a key driver of DMV formation, and stabilizes NSP3 by preventing its ubiquitin-mediated degradation. We further identified K963 as the ubiquitination site on NSP3 and RBBP6 as its E3 ubiquitin ligase. Overexpression of *ECHS1* disrupted the interaction between NSP3 and RBBP6, while *RBBP6* overexpression or knockdown had no effect on the stability of the NSP3 K963R mutant. These results strongly suggest that ECHS1 and RBBP6 compete for binding to NSP3 to regulate its abundance. Interestingly, RBBP6 has also been identified as a negative regulator of Ebola virus replication, where it competes with NP for VP30 binding (Batra et al, 2018). Together with our findings, this suggests that RBBP6 may function as a broad-spectrum host restriction factor against multiple viruses.

In conclusion, we propose a refined model of coronavirus replication organelle dynamics, centered on a self-amplifying inter-organellar feedback loop. The cycle begins with DMV biogenesis disrupting ER-mitochondria contacts, leading to mitochondrial injury. The damaged organelle then releases ECHS1, which translocates to the cytosol where it stabilizes NSP3 by antagonizing its RBBP6-mediated ubiquitination. This stabilization drives further DMV assembly, perpetuating the cycle. This work fundamentally advances the concept of viral replication organelles from isolated entities to integrated hubs within a dynamic cellular network. It illustrates that the battle between virus and host is not only fought on the level of individual proteins or pathways but is also waged across the spatial architecture of the cell itself. Targeting the critical nodes of this inter-organellar dialogue, such as the ECHS1-NSP3 interface, offers a compelling and previously unexplored therapeutic avenue to disrupt the intricate economy of viral replication.

## Methods

**Table:** Reagents and tools table

Reagent/resource	Reference or source	Identifier or catalog number
**Experimental models**		
Human: HEK293T	ATCC	Cat# CRL3216
Mouse: 17Cl-1	Gifted by Hongyu Deng	N/A
African green monkey: Vero E6	Gifted by Xiancai Ma	N/A
Human: Hela	Gifted by Binbin Ding	N/A
GFP1-10-SEC61β + TOMMO20-GFP11 (HeLa)	Gifted by Maoge Zhou	N/A
Dox inducible mCherry NSP3 + NSP4-GFP (HeLa)	Gifted by Binbin Ding	N/A
Vector OE (Vero E6/17Cl-1)	This paper	N/A
MFN2 OE (Vero E6/17Cl-1)	This paper	N/A
RMDN3 OE (Vero E6/17Cl-1)	This paper	N/A
MFN2 + RMDN3 OE (Vero E6/17Cl-1)	This paper	N/A
Ctrl (HeLa/17Cl-1)	This paper	N/A
*ECHS1*-KO (HeLa/17Cl-1)	This paper	N/A
*ECHS1*-Rescue (HeLa//17Cl-1)	This paper	N/A
**Recombinant DNA**		
CMV-GFP-NSP3	Hong Zhang lab	Ji et al, 2022, 10.1083/jcb.202112081
CMV-Flag-NSP3	Hong Zhang lab	Ji et al, 2022, 10.1083/jcb.202112081
CMV-GFP-NSP4	Hong Zhang lab	Ji et al, 2022, 10.1083/jcb.202112081
CMV-Flag-NS4	Hong Zhang lab	Ji et al, 2022, 10.1083/jcb.202112081
CMV-HA-ubiquitin	This paper	N/A
CMV-RBBP6-Flag	This paper	N/A
CMV-NSP4-mCherry	This Lab	Yang et al, 2025, 10.1083/jcb.202306101
CMV-NSP4-BFP	This Lab	Yang et al, 2025, 10.1083/jcb.202306101
CMV-Flag-NSP3 1-1491	This paper	N/A
CMV-Flag-NSP3 1-374	This paper	N/A
CMV-Flag-NSP3 374-803	This paper	N/A
CMV-Flag-NSP3 803-1334	This paper	N/A
CMV-Flag-NSP3 1546-1945	This paper	N/A
CMV-GFP-NSP3-T2A-NSP4	This paper	N/A
CMV-SEC61B-GFP	This paper	N/A
CMV-KDEL-RFP	This paper	N/A
CMV-ORF3a-mCherry	This paper	N/A
CMV-NSP8-mCherry	This paper	N/A
CMV-N-mCherry	This paper	N/A
CMV-M-mCherry	This paper	N/A
CMV-HA-RMDN3	This paper	N/A
CMV-Flag-MFN2	This paper	N/A
CMV-OMM-RFP-ER Linker	This paper	Csordás et al, 2006, 10.1083/jcb.200604016
CMV-ECHS1-GFP	This paper	N/A
CMV-ECHS1-HA	This paper	N/A
Lenti-CRISPR-V2	Addgene	Cat# 52961
pET28a-SARS-COV-2 NSP3 1-1491	This paper	N/A
pGEX-6p-1-SARS-COV-2 NSP3 803-1334	This paper	N/A
pET28a-ECHS1 28-290	This paper	N/A
**Antibodies**		
Rabbit anti-MFN2	Proteintech	Cat# 12186-1-AP
Rabbit anti-PTPIP51(RMDN3)	Proteintech	Cat# 20641-1-AP
Rabbit anti-SARS-CoV-2 NSP3 antibody	Genetex	Cat# GTX135589
Rabbit anti-SARS-CoV-2 NSP4 antibody	Prosci	Cat# 9175
Rabbit anti-SARS-CoV-2 N antibody	Sino Biological	Cat# 40143-T62
Rabbit anti-TOMM20	Abcam	Cat# ab186735
Rabbit anti-GFP	Proteintech	Cat# 50430-2-AP
Mouse anti-Flag	Proteintech	Cat# 66008-4-Ig
Rabbit anti-ECHS1	Proteintech	Cat# 11305-1-AP
Rabbit anti-RBBP6	Proteintech	Cat# 11882-1-AP
Rabbit anti-RBBP6	Thermo Fisher Scientific	Cat# PA5-23054
Mouse anti-α-Tubulin	Proteintech	Cat# 66031-1-Ig
Mouse anti-MHV NSP3	InfectoBio	Cat# 4C12
Mouse anti-MHV N	InfectoBio	Cat# 2G2
Rabbit IRDye® 800CW	LI-COR	Cat# 926-32211
Mouse IRDye® 680RD	LI-COR	Cat# 926-68070
**Oligonucleotides and other sequence-based reagents**		
Primer sequences for qPCR	This paper	Table EV1
Sequences of oligonucleotides for siRNA	This paper	Table EV2
**Chemicals, enzymes and other reagents**		
Anti-FLAG affinity beads	Smart-lifesciences	Cat# SA042005
Anti-GFP Magarose Beads	Smart-lifesciences	Cat# SM38005
Lipofectamine™ 3000 Transfection Reagent	Invitrogen	Cat# L3000015
Opti-MEM® I Reduced Serum Medium	Gibco	Cat# 31985070
TrypLE™ Express Enzyme (1×), phenol red	Thermo Fisher	Cat# 12605028
Fetal Bovine Serum, Premium Plus	Thermo Fisher	Cat# A5669701
Penicillin-Streptomycin, Liquid (100×)	Invitrogen	Cat# 15140122
Polybrene (Hexadimethrine Bromide)	Beyotime	Cat# C0351
Hoechst 33342	Thermo Fisher	Cat# H1399
Puromycin Dihydrochloride	Beyotime	Cat# ST551
Hygromycin B	Beyotime	Cat# ST1389
TRIzol™ Reagent	Invitrogen	Cat# 15596018
Cycloheximide	MedChemExpress	Cat# Y-12320
MG-132	MedChemExpress	Cat# HY-13259
Chloroquine	MedChemExpress	Cat# HY-17589A
Bafilomycin A1	MedChemExpress	Cat# HY-100558
MFN2 agonist-1 (B-A/l)	MedChemExpress	Cat# HY-123985
Crystal Violet	Sangon Biotech	Cat# A100528-0025
Methyl cellulose	Sigma-Aldrich	Cat# M6385
Glutaraldehyde	Electron Microscopy Sciences	Cat# 16200
MitoTracker Deep Red FM	Thermo Fisher	Cat# M22426
PK Mito Red	Genvivotech	Cat# PKMR-1
PK Mito Orange Fix	Genvivotech	Cat# PKMOF-1
Hank’s Balanced Salt Solution (HBSS)	Gibco	Cat# 14025076
LDS loading buffer	GeneScript	Cat# M00676
**Software**		
GraphPad Prism v8.2.1	https://www.graphpad.com/features	
Snapgene v4.1.8	https://www.snapgene.com	
ImageJ (Fiji)	https://imagej.net	
**Other**		
Orbitrap Exploris 480	Thermo Fisher Scientific	
Mitochondria Isolation Kit	Thermo Fisher Scientific	Cat# 89874

### Cell culture, virus strains

All cell lines were maintained in Dulbecco’s Modified Eagle Medium (DMEM) (Invitrogen) supplemented with 10% fetal bovine serum (FBS) and 1% penicillin–streptomycin at 37 °C in a 5% CO_2_ atmosphere. Virus strains used in this study are as follows: MHV A59 (gift from Professor Hongyu Deng, Institute of Biophysics, Chinese Academy of Sciences), WIV1 (gift from Professor Peng Zhou, Guangzhou National Laboratory) and SARS-CoV-2 (Omicron-BA.5).

MHV-A59 was propagated in 17Cl-1 cells, WIV1 and SARS-CoV-2 were propagated in Vero E6 cells. Viral stocks were stored at -80 °C. SARS-CoV-2 infection was conducted in Guangzhou Customs District Technology Center BSL-3 Laboratory and Guangzhou National Laboratory BSL-3 Laboratory, while other infections were carried out in BSL-2 laboratory of Guangzhou National Laboratory.

### Plasmids, transfection and chemicals

Plasmids of GFP-NSP3, Flag-NSP3, NSP4-GFP, NSP4-Flag, which encode the entire NSP3 or NSP4 coding sequence of SARS-CoV-2, were kindly provided by Prof. Hong Zhang (Institute of Biophysics, Chinese Academy of Sciences) (Ji et al, 2022). N-terminal HA-tagged ubiquitin (HA-Ub) and C-terminal Flag-tagged RBBP6 (RBBP6-Flag) expression plasmids were kindly provided by Prof. Binbin Ding (Guangzhou National Laboratory). NSP4-mCherry and NSP4-BFP were modified from NSP4-GFP and have been previously described (Yang et al, 2025). Flag-NSP3^1-1491^, Flag-NSP3^1-374^, Flag-NSP3^374-803^, Flag-NSP3^803-1334^, Flag-NSP3^1546-1945^ were constructed from Flag-NSP3. GFP-NSP3-T2A-NSP4 was modified from GFP-NSP3 in our laboratory by inserting T2A-NSP4 into the C-terminus of GFP-NSP3, enabling co-expression of NSP3 and NSP4 from a single vector (Zhou et al, 2025). Plasmids of SEC61B-GFP, KDEL-RFP, ORF3a-mCherry, NSP8-mCherry, N-mCherry, and M-mCherry were kept in our laboratory. To construct the MFN2 plasmid with a C-terminal Flag epitope tag, the PCR-amplified MFN2 coding sequence was subcloned into the PCDH-CMV-Flag-EF1-Puro vector. The RMDN3 expression plasmid was constructed by inserting the RMDN3 coding sequence into the Plenti-CMV-HA-Hygro vector. The OMM-ER linker was synthesized based on sequences designed and constructed in previous reports (Csordás et al, 2006). ECHS1-GFP was constructed by inserting the ECHS1 coding sequence into the Plenti-CMV-GFP-Hygro vector. ECHS1-HA was generated by inserting the ECHS1 coding sequence into the Plenti-CMV-HA-Hygro vector.

To establish stable overexpression cell lines, control plasmids expressing a Flag tag (PCDH-CMV-Flag-EF1-Puro) or HA tag (Plenti-CMV-HA-Hygro) were transfected alongside experimental constructs as negative controls. To establish stable knockout cell lines, a non-targeting lenti-CRISPR-Cas9 v2 plasmid was used as a negative control.

All PCR reactions were conducted using Phanta Max Super-Fidelity DNA Polymerase (Vazyme, P505-d1). Cells were transfected with plasmid DNA using Lipofectamine 3000 (Invitrogen, L3000015) when they reached 50–75% confluence, according to the manufacturer’s instructions.

### TEM

TEM was performed as described previously (Yang et al, 2025). Briefly, cells were trypsinized and harvested by centrifugation at 800 × *g* for 5 min. Cell pellets were fixed with 2.5% glutaraldehyde (in PBS, pH 7.4) at 4 °C overnight. For samples transfected with GFP-NSP3 and NSP4-mCherry (to induce DMV formation), double-positive cells (GFP/mCherry) were sorted using a flow cytometer (MA900, Sony), seeded onto ACLAR films and incubated overnight to allow cell adhesion. Adhered cells were then fixed in 2.5% glutaraldehyde (in PBS, pH 7.4) at 4 °C for subsequent TEM processing. After fixation, all samples were washed with double-distilled water (ddH_2_O) and post-fixed in 1% osmium tetroxide (OsO_4_) at 4 °C for 1.5 h to enhance membrane contrast. Samples were then washed with ddH_2_O, incubated in chilled 2% aqueous uranyl acetate for 1 h at room temperature. After rinsing with ddH_2_O, cells underwent dehydration through a graded series of ethanol solutions (30%, 50%, 70%, 80%, 90%, 100%) and were subsequently embedded in epoxy EMBED-812 resin. The embedding mixture was polymerized at 60 °C for 48 h to form solid blocks suitable for ultrathin sectioning. TEM was performed using a Talos L120C electron microscope (Thermo Fisher Scientific) operated at an accelerating voltage of 100 kV.

Mitochondrial morphological normality was evaluated with TEM images, focusing on three key ultrastructural features: vacuolation, outer membrane boundary clarity, and cristae integrity. Given the inherent morphological heterogeneity of mitochondria in normal cells, mitochondria were defined as “impaired” only when exhibiting moderate-to-severe damage, characterized by multiple vacuoles or a single large vacuole (occupying 20–50% of mitochondrial volume); outer membrane abnormalities, including blurred boundaries in multiple regions, local discontinuity, or widespread blurring with complete discontinuity; and cristae damage, manifested as moderate cristae loss (20–50% of the area) or severe cristae loss (>50% of the area) or complete cristae disappearance. Mitochondria not exhibiting any of these features simultaneously were categorized as “morphologically normal”. For quantitative analysis of the mitochondrial morphology, the analysis was based on three independent experimental replicates. Across the three replicates, a total of >30 cells were counted per experimental group. The proportion of morphologically normal mitochondria was calculated as (number of mitochondria without structural abnormalities/total number of counted mitochondria) × 100%, which served as the quantitative indicator for evaluating mitochondrial ultrastructural integrity.

### Immunofluorescence microscopy

Mitochondria were labeled using the fluorescent dyes PK Mito Red (Genvivotech, PKMR-1), PK Mito Orange Fix (Genvivotech, PKMOF-1), and Mito-Tracker Deep Red (Thermo Fisher Scientific, M22426) according to the manufacturers’ instructions. Additionally, SEC61B-GFP and KDEL-RFP were constructed via a lentiviral vector and used to label ER. For fixed samples: Cells were first fixed with 4% paraformaldehyde (PFA) for 10 min at room temperature. After fixation, cell membranes were permeabilized with 0.1% Triton X-100 in phosphate-buffered saline (PBS, pH 7.4) for 10 min, followed by blocking nonspecific binding sites with 3% bovine serum albumin (BSA; Sigma-Aldrich, A2153) in PBS for 1 h at room temperature. Primary antibodies used were as follows: rabbit anti-GFP (1:500; Proteintech, 50430-2-AP), mouse anti-Flag (1:500; Proteintech, 66008-4-Ig), mouse anti-dsRNA (1:1000; Scicons, 10010200), and rabbit anti-SARS-CoV-2 N antibody (cross-reactive with the N protein of WIV1, 1:500; Sino Biological, 40143-T62). Cells were incubated with primary antibodies at 4 °C overnight. The following secondary antibodies were used: goat anti-rabbit IgG (H + L) cross-adsorbed secondary antibody conjugated to Alexa Fluor 568 (1:500; Invitrogen, A11011) and goat anti-mouse IgG (H + L) highly cross-adsorbed secondary antibody conjugated to Alexa Fluor Plus 647 (1:500; Invitrogen, A32728). Cells were incubated with secondary antibodies for 1 h at room temperature in the dark. Images were obtained using a Nikon A1 confocal microscope or a Carl Zeiss Elyra7 confocal microscope. Fluorescence intensity analysis of the images was performed using ImageJ software.

### Mitochondrial isolation

Mitochondrial and cytosolic fractions were isolated from 17Cl-1 and HeLa cells using the Mitochondria Isolation Kit (Thermo Fisher Scientific, 89874) according to the manufacturer’s instructions. Briefly, 2 × 10^7^ cells were washed with ice-cold PBS, detached via scraping, and pelleted at 850 × *g* for 5 min at 4 °C. The pellet was resuspended in 800 μL ice-cold Isolation Buffer A (supplemented with fresh protease inhibitors) and homogenized thoroughly on ice using a Dounce Tissue Grinder. After homogenization, 800 μL of Isolation Buffer C was added to the lysed cells, which were then centrifuged at 700 × *g* for 10 min at 4 °C to remove nuclei. The supernatant was transferred and centrifuged at 3000 × *g* for 15 min at 4 °C to pellet mitochondria. The supernatant (cytosolic fraction) was transferred, and the pellet was resuspended in 500 μL ice-cold Isolation Buffer C. Lastly, the mixture was centrifuged at 12,000 × *g* for 5 min at 4 °C, and mitochondria were recovered as the pellet following this centrifugation. Isolated mitochondria were used immediately for the following assays. Mitochondrial integrity and purity were verified by immunoblotting using anti-TOMM20 (1:1000; Abcam, ab186735) and anti-α-Tubulin (1:5000; Proteintech, 66031-1-Ig) antibodies.

### Immunoblotting

Cells (~5× 10^5^ per sample) were harvested and lysed in ice-cold RIPA buffer (Beyotime, P0013B), supplemented with a protease inhibitor cocktail (Sigma-Aldrich, P8340). For co-IP analysis, cells were harvested and lysed in ice-cold IP buffer (150 mM NaCl, 10% glycerol, 50 mM Tris-HCl pH 7.4, 5 mM EDTA, 1% NP-40, 0.5% Triton X-100, protease inhibitor) for 30 min on ice. The homogenates were centrifuged at 12,000 × *g* for 15 min at 4 °C, and the supernatants were incubated with anti-Flag affinity beads (Smart-Lifesciences, SA042005) or anti-GFP Magarose Beads (Smart-Lifesciences, SM38005) overnight at 4 °C on a rotator. The beads were washed 5 times with IP buffer, then mixed with 4× LDS loading buffer (GeneScript, M00676), boiled at 95 °C for 10 min, and the samples were analyzed by immunoblotting. Cellular protein samples were separated by 10% SDS-polyacrylamide gel electrophoresis using a homemade gel, followed by electrophoretic transfer onto nitrocellulose membranes (Merck Millipore, HATF00010) for 1.5 h. The membranes were blocked with 5% nonfat dry milk in Tris-buffered saline containing 0.1% Tween-20 (Sigma-Aldrich, P1379) (TBST) for 1 h at room temperature. Then primary antibodies were added and incubated for 16 h at 4 °C. Antibodies were purchased from the following vendors: rabbit anti-MFN2 (1:3000; Proteintech, 12186-1-AP), rabbit anti-PTPIP51 (also known as RMDN3, 1:1000; Proteintech, 20641-1-AP), rabbit anti-SARS-CoV-2 NSP3 antibody (1:1000; Genetex, GTX135589), rabbit anti-SARS-CoV-2 NSP4 antibody (1:1000; Prosci, 9175), rabbit anti-SARS-CoV-2 N antibody (1:2000, Sino Biological, 40143-T62), rabbit anti-TOMM20 (1:1000; Abcam, ab186735), rabbit anti-GFP (1:3000; Proteintech, 50430-2-AP), mouse anti-Flag (1:5000; Proteintech, 66008-4-Ig), rabbit anti-ECHS1(1:1000; Proteintech, 11305-1-AP), rabbit anti-RBBP6 (1:3000; Proteintech, 11882-1-AP, for WB), rabbit anti-RBBP6 (1:200; Thermo Fisher Scientific, PA5-23054, for IP), mouse anti-α-Tubulin (1:5000; Proteintech, 66031-1-Ig), mouse anti-MHV NSP3 (1:1000; InfectoBio, 4C12), mouse anti- MHV N (1:1000; InfectoBio, 2G2).

Fluorescent secondary antibodies IRDye® 800CW (rabbit, 1:10,000; LI-COR, 926-32211) or IRDye® 680RD (mouse, 1:10,000; LI-COR, 926-68070) were incubated for 1 h at room temperature in the dark. Protein bands were scanned and quantified using an Odyssey M Imager (LI-COR). Densitometric analysis of target protein band intensities was performed using ImageJ software.

### qPCR

Total RNA was extracted using TRIzol reagent (Invitrogen, 15596018CN) followed by an isopropanol precipitation protocol. The RNA pellet was resuspended in DEPC-treated water and quantified using a NanoDrop One spectrophotometer (Thermo Fisher Scientific). RNA purity was assessed by measuring the 260/280 absorbance ratio. cDNA was synthesized from 1000 ng of total RNA using the HiScript II 1st Strand cDNA Synthesis Kit (Vazyme, R212-01) following the manufacturer’s instructions. qPCR was performed using ChamQ Universal SYBR qPCR Master Mix (Vazyme, Q711-02) on a QuantStudio 6 Flex Real-Time PCR System (Applied Biosystems). The sequences of primers used for detection are listed in Table EV1. The relative expression levels of individual genes were normalized to *GAPDH* using the 2^-ΔΔCt^ method. All qPCR reactions were performed in technical triplicates for each biological sample, and experiments included at least three independent biological replicates.

### RNA interference and transfection

Small interfering RNAs (siRNAs) targeting genes were designed and synthesized by Ruibiotech (Beijing, China). siRNA transfections were performed using Lipofectamine RNAiMAX Reagent (Thermo Fisher Scientific, 13778150) and Opti-MEM Medium (Thermo Fisher Scientific, 31985-062) according to the manufacturer’s protocol. Cells were plated in a 12-well plate and transfected with 50 nM siRNA. Cells were then incubated under standard culture conditions for 36–48 h. The specific siRNA sequences are listed in Table EV2.

### RNA sequencing

For transcriptional analysis, total RNA from cultured cell were prepared with TRIzol regent, and the sequencing libraries were synthesized using MGIEasy Fast RNA Library Prep Kit for DNBSEQ platform according to the manufacturer’s instructions. The libraries were sequenced with PE150 (IGE BIO, Guangzhou, China).

### Protein purification and in vitro binding assay

For recombinant SARS-COV-2 NSP3^1-1491^ protein, the coding sequence was subcloned into pET28a vector, which contains an N-terminal SUMO-His-tag and Ulp1 cleavage site. The recombinant protein was expressed in *E.coli* BL21 (DE3) induced with 0.2 mM IPTG at 16 °C for 18 h. Cells were collected and lysed by sonication in PBS buffer containing 200 mM NaCl, 20 mM imidazole and 1 mM EDTA. The supernatant was collected by centrifugation at 12,000 × *g* for 45 min at 4 °C, which was then incubated with Ni-NTA beads (GE Healthcare) for 2 h at 4 °C. SUMO-NSP3^1-1491^ was eluted by elution buffer (PBS containing 400 mM NaCl and 250 mM imidazole). The protein was subjected to buffer exchanged and tag cleavage using Ulp1 protease with PBS containing 1 mM EDTA and 5% glycerol, and stored at -80 °C.

For recombinant SARS-COV-2 NSP3^803-1334^ protein, the NSP3 (residues 803–1334, NSP3^803-1334^) were subcloned into the pGEX-6p-1 vector with an N terminus GST tag. The recombinant protein was expressed in *E. coli* BL21 (DE3) with 0.2 mM IPTG at 16 °C for 18 h. Cell pellets were lysed by sonication in PBS buffer containing 400 mM NaCl, 5% glycerol and 1 mM DTT. GST beads (GE Healthcare) were incubated with clarified supernatant and further eluted by 10 mM GSH. The eluted GST-NSP3^803-1334^ was dialyzed against to buffer (20 mM HEPES pH 7.5, 150 mM NaCl, and 1 mM DTT) at 4 °C, and further purified by size exclusion chromatography using a Superdex 200 column (GE Healthcare) in buffer (20 mM HEPES pH 7.5, 100 mM NaCl, 5% glycerol, 1 mM DTT). The purified protein was aliquoted and stored at −80 °C.

For recombinant human ECHS1^28-290^ protein, the coding sequence was subcloned into pET28a vector with N-terminal 6 × His-tag. The recombinant protein was expressed in *E.coli* BL21 (DE3) induced with 1 mM IPTG at 37 °C for 4 h. Cells were collected and lysed by sonication in PBS containing 1% Triton X-100. The cell lysate was centrifuged at 12,000 × *g* for 45 min at room temperature, and pellet was incubated with PBS buffer containing 200 mM NaCl, 8 M urea and 0.1% Triton X-100, followed by incubation for 20 min at room temperature. The supernatant was collected by centrifugation at 12,000 × *g* for 45 min, then was incubated with Ni-NTA beads (GE Healthcare) for 1 h at room temperature with gentle rotation. ECHS1^28-290^ was eluted with elution buffer (PBS containing 200 mM NaCl, 250 mM imidazole and 0.1% Triton X-100). The His-ECHS1 were then refolded by dialysis method, and subjected to buffer exchange with PBS containing 1 mM EDTA and 5% glycerol, and stored at -80 °C.

For in vitro binding assay, pre-equilibrium Ni-NTA beads were incubated with 5 μg His-ECHS1 for 45 min at 4 °C. The beads were washed with PBS buffer containing 200 mM NaCl, 20 mM imidazole and 1 mM EDTA, then re-incubated with 5 μg NSP3^1-1491^ protein for 2 h at 4 °C. Nonspecifically bound proteins were removed by washing the beads three times with the same buffer. The samples were then resolved by SDS-PAGE and analyzed by immunoblotting.

### Virus plaque assay for MHV titration

The plaque assay was performed using 17Cl-1 cells as follows: 17Cl-1 cells were seeded in 12-well plates and cultured until reaching 70-80% confluence. At this point, they were infected with serially diluted MHV stock (tenfold dilutions prepared in fresh medium). After aspirating 600 μL of medium, 100 μL of viral dilution was added per well, followed by incubation at 37 °C for 1–2 h with gentle rocking every 15 min. Subsequently, cells were overlaid with 4 mL of methylcellulose-based medium per well and cultured for 72 h. After incubation, the overlay medium was discarded, and cells were stained with 1 mL of 0.2% crystal violet in 20% ethanol for 2 h. Cells were then washed and air-dried. Plaques were enumerated, and viral titer (Plaque Forming Unit, PFU/mL) was calculated as: PFU/mL = (Plaque count)/(Dilution factor × 0.1 mL inoculum volume).

### Fluorescent focus assay for SARS-CoV-2/WIV1

Vero E6 cells were seeded in 96-well plates at a density of 1 × 10^4^ cells/well and cultured in DMEM until reaching 90–100% confluency. Serially diluted samples (tenfold dilutions prepared in fresh medium) were prepared by diluting the supernatant from SARS-CoV-2/WIV1-infected cells. Prior to infection, the culture medium in the 96-well plates was completely aspirated and 50 μL of each dilution was added to triplicate wells. The plates were incubated at 37 °C with 5% CO_2_ for 1 h, with gentle rocking every 15 min. After virus adsorption, the supernatant was removed and replaced with 100 μL/well of overlay medium consisting of 1.6% methylcellulose in DMEM supplemented with 2% FBS and 1% penicillin–streptomycin (prepared by mixing equal volumes of 3.2% methylcellulose and 2× DMEM). The plates were then returned to the 37 °C, 5% CO_2_ incubator and cultured for 24 hpi. The overlay medium was carefully aspirated, and cells were fixed with 4% PFA for 10 min at room temperature. After fixation, PFA was removed and cells were permeabilized with 0.1% Triton X-100 in PBS for 30 min, followed by blocking with 3% BSA in PBS for 1 h at room temperature. The plates were sequentially stained with 50 μL rabbit anti-SARS-CoV-2 N antibody (1:500, Sino Biological, 40143-T62) as the primary antibody and goat anti-rabbit IgG Alexa Fluor Plus 488 as the secondary antibody (1:500, Invitrogen, A32731). The number of fluorescent foci was visualized and counted using fluorescence microscopy. Viral titer (focus-forming unit, FFU/mL) was calculated: Average number of foci per well/(Dilution factor  × Volume of inoculum in mL).

### Data-independent acquisition mass spectrometry (DIA-MS)

Sample preparation: prepared samples were resolved by SDS-PAGE, and gel bands were cut into 1–2 mm^3^ cubes. For Coomassie-stained gels, gel cubes were destained with 50 mM ammonium bicarbonate/acetonitrile (2:3, vol/vol). Gel cubes were then dehydrated with neat acetonitrile with shaking until the gel pieces turned white and shrink, followed by acetonitrile removal. Next, the protein gel cubes were reduced with 10 mM dithiothreitol at 56 °C for 30 min, alkylated with 55 mM iodoacetamide at room temperature in the dark for 30 min, and digested with trypsin at 50 °C for 1 h in 50 mM ammonium bicarbonate containing 0.01% ProteaseMAX Surfactant. Peptide digestion products were collected from the gel supernatant and desalted using homemade C18 StageTips. The eluted peptide solutions were combined and evaporated to dryness using a vacuum centrifugal concentrator (CV600, Beijing JMTechnology Co., Ltd.).

LC-MS/MS analysis: peptide samples were analyzed by liquid chromatography-tandem MS (LC-MS/MS) by combining a Vanquish Neo UHPLC connected online to an Orbitrap Exploris 480 mass spectrometer. A 250 mm Acclaim PepMap100 C18 column (Thermo Fisher Scientific) with an internal diameter of 75 μm was used to separate the peptides with mobile phase A (0.1% FA in water) and mobile phase B (0.1% FA in 80% ACN) using a 62 min gradient: 2–32% in 48 min at a flow rate of 300 nL/min, 32–50% in 9 min at a flow rate of 300 nL/min, 50–95% B in 1 min at a flow rate of 330 nL/min, and then kept B at 95% for 4 min at a flow rate of 400 nL/min.

The Orbitrap Exploris 480 mass spectrometer was operated in a data-independent acquisition (DIA) mode. The nano electrospray ion source spray voltage was 2.2 kV; no sheath gas flow; the heated capillary temperature was 320 °C. For DIA experiments, full MS resolutions were set to 60,000 with a full scan range of 350–1400 *m/z* and full MS AGC target was 300% with an IT of 45 ms. For MS/MS spectra, AGC target value for fragment spectra was set at 1000%; 20 variable windows from 17 Da to 558 Da were used; resolution was set to 30,000 and IT to 45 ms; HCD normalized collision energy was set at 30%. FAIMS was used and voltage was set as −45 V and −65 V. The other parameters were set as default.

Data analysis: the raw data were processed by Spectronaut v18 with the “Direct-DIA” mode for protein identification and quantification. The data were searched against the *Homo sapiens* or *Mus musculus* database downloaded from UniProt. Trypsin was chosen as an enzyme with a maximum of two missed cleavages. Carbamidomethylation on cysteine was considered as a static modification; Oxidation on methionine and protein N-terminal acetylation were selected as dynamic modifications. For the identification of DIA analysis, precursor *q*-value (FDR) and PEP cutoff were set to 0.001 and 0.001, protein q-value (FDR) and PEP cutoff were set to 0.001 and 0.01. The other parameters were set as default.

### Statistical analysis

All quantitative data are presented as the mean ± SD from at least 3 independent experiments. Significance was assessed using unpaired two-tailed Student’ s *t* tests or analysis of variance (ANOVA) with GraphPad Prism (version 8), and the *P* ≤ 0.05 was considered statistically significant.

RNA raw sequence reads were aligned to the reference genome using STAR version 2.7.2b, and the raw and transcripts per million (TPM) count values were determined using RSEM version 1.3.3.

## Supplementary information

Table EV1

Table EV2

Peer Review File

Source data Fig. 1

Source data Fig. 2

Source data Fig. 3

Source data Fig. 4

Source data Fig. 5

Source data Fig. 6

Source data Fig. 7

Figure EV1 Source Data

Figure EV2 Source Data

Figure EV3 Source Data

Figure EV4 Source Data

Figure EV5 Source Data

Figure EV6 Source Data

Figure EV7 Source Data

Figure EV8 Source Data

Figure EV9 Source Data

Expanded View Figures

## Data Availability

This study includes no data deposited in external repositories. The source data of this paper are collected in the following database record: biostudies:S-SCDT-10_1038-S44318-026-00816-x.

## References

[CR1] Batra J, Hultquist JF, Liu D, Shtanko O, Von Dollen J, Satkamp L, Jang GM, Luthra P, Schwarz TM, Small GI et al (2018) Protein interaction mapping identifies RBBP6 as a negative regulator of Ebola virus replication. Cell 175:1917–1930.e13 30550789 10.1016/j.cell.2018.08.044 PMC6366944

[CR2] Burgin H, Sharpe AJ, Nie S, Ziemann M, Crameri JJ, Stojanovski D, Pitt J, Ohtake A, Murayama K, McKenzie M (2023) Loss of mitochondrial fatty acid β-oxidation protein short-chain Enoyl-CoA hydratase disrupts oxidative phosphorylation protein complex stability and function. FEBS J 290:225–246 35962613 10.1111/febs.16595 PMC10087869

[CR3] Cai K, Wang F, Lu JQ, Shen AN, Zhao SM, Zang WD, Gui YH, Zhao JY (2022) Nicotinamide mononucleotide alleviates cardiomyopathy phenotypes caused by short-chain enoyl-CoA hydratase 1 deficiency. JACC Basic Transl Sci 7:348–362 35540099 10.1016/j.jacbts.2021.12.007 PMC9079797

[CR4] Chatel-Chaix L, Cortese M, Romero-Brey I, Bender S, Neufeldt CJ, Fischl W, Scaturro P, Schieber N, Schwab Y, Fischer B et al (2016) Dengue virus perturbs mitochondrial morphodynamics to dampen innate immune responses. Cell Host Microbe 20:342–356 27545046 10.1016/j.chom.2016.07.008 PMC7105029

[CR5] Che W, Guo S, Wang Y, Wan X, Tan B, Li H, Alifu J, Zhu M, Chen Z, Li P, Zhang L, Zhang Z, Wang Y, Huang X, Wang X, Zhu J, Pan X, Zhang F, Wang P, Sui SF, Zhao J, Xu Y, Liu Z (2025) SARS-CoV-2 damages cardiomyocyte mitochondria and implicates long COVID-associated cardiovascular manifestations. J Adv Res 80:743–758 10.1016/j.jare.2025.05.013 PMC12869202 40354933

[CR6] Chu J, Xing C, Du Y, Duan T, Liu S, Zhang P, Cheng C, Henley J, Liu X, Qian C et al (2021) Pharmacological inhibition of fatty acid synthesis blocks SARS-CoV-2 replication. Nat Metab 3:1466–1475 34580494 10.1038/s42255-021-00479-4 PMC8475461

[CR7] Cook KC, Tsopurashvili E, Needham JM, Thompson SR, Cristea IM (2022) Restructured membrane contacts rewire organelles for human cytomegalovirus infection. Nat Commun 13:1–20 35953480 10.1038/s41467-022-32488-6 PMC9366835

[CR8] Cortese M, Lee JY, Cerikan B, Neufeldt CJ, Oorschot VMJ, Köhrer S, Hennies J, Schieber NL, Ronchi P, Mizzon G et al (2020) Integrative imaging reveals SARS-CoV-2-induced reshaping of subcellular morphologies. Cell Host Microbe 28:853–866.e5 33245857 10.1016/j.chom.2020.11.003 PMC7670925

[CR9] Csordás G, Renken C, Várnai P, Walter L, Weaver D, Buttle KF, Balla T, Mannella CA, Hajnóczky G (2006) Structural and functional features and significance of the physical linkage between ER and mitochondria. J Cell Biol 174:915–921 16982799 10.1083/jcb.200604016 PMC2064383

[CR10] Csordás G, Weaver D, Hajnóczky G (2018) Endoplasmic reticulum–mitochondrial contactology: structure and signaling functions. Trends Cell Biol 28:523–540 29588129 10.1016/j.tcb.2018.02.009 PMC6005738

[CR11] De Brito OM, Scorrano L (2008) Mitofusin 2 tethers endoplasmic reticulum to mitochondria. Nature 456:605–610 19052620 10.1038/nature07534

[CR12] De vos KJ, Mórotz GM, Stoica R, Tudor EL, Lau KF, Ackerley S, Warley A, Shaw CE, Miller CCJ (2012) VAPB interacts with the mitochondrial protein PTPIP51 to regulate calcium homeostasis. Hum Mol Genet 21:1299–1311 22131369 10.1093/hmg/ddr559 PMC3284118

[CR13] Foo J, Bellot G, Pervaiz S, Alonso S (2022) Mitochondria-mediated oxidative stress during viral infection. Trends Microbiol 30:679–692 35063304 10.1016/j.tim.2021.12.011

[CR14] Freppel W, Barragan Torres VA, Uyar O, Anton A, Nouhi Z, Broquière M, Mazeaud C, Sow AA, Léveillé A, Gilbert C et al (2025) Dengue virus and Zika virus alter endoplasmic reticulum-mitochondria contact sites to regulate respiration and apoptosis. iScience 28:111599 39834870 10.1016/j.isci.2024.111599 PMC11743106

[CR15] Gao X, Chen X, Yu L, Zhao S, Jiu Y (2025) Host cytoskeleton and membrane network remodeling in the regulation of viral replication. Biophys Rep 11:34–45 40070659 10.52601/bpr.2024.240040 PMC11891074

[CR16] Huang Y, Wang T, Zhong L, Zhang W, Zhang Y, Yu X, Yuan S, Ni T (2024) Molecular architecture of coronavirus double-membrane vesicle pore complex. Nature 633:224–231 39143215 10.1038/s41586-024-07817-y PMC11374677

[CR17] Ji M, Li M, Sun L, Zhao H, Li Y, Zhou L, Yang Z, Zhao X, Qu W, Xue H et al (2022) VMP1 and TMEM41B are essential for DMV formation during β-coronavirus infection. J Cell Biol 221:e202112081 35536318 10.1083/jcb.202112081 PMC9097365

[CR18] Klein S, Cortese M, Winter SL, Wachsmuth-Melm M, Neufeldt CJ, Cerikan B, Stanifer ML, Boulant S, Bartenschlager R, Chlanda P (2020) SARS-CoV-2 structure and replication characterized by in situ cryo-electron tomography. Nat Commun 11:1–10 33208793 10.1038/s41467-020-19619-7 PMC7676268

[CR19] Knoops K, Kikkert M, Van Den Worm SHE, Zevenhoven-Dobbe JC, Van Der Meer Y, Koster AJ, Mommaas AM, Snijder EJ (2008) SARS-coronavirus replication is supported by a reticulovesicular network of modified endoplasmic reticulum. PLoS Biol 6:1957–1974 10.1371/journal.pbio.0060226 PMC2535663 18798692

[CR20] Krols M, Bultynck G, Janssens S (2016) ER-Mitochondria contact sites: a new regulator of cellular calcium flux comes into play. J Cell Biol 214:367–370 27528654 10.1083/jcb.201607124 PMC4987300

[CR21] Lenhard S, Gerlich S, Khan A, Rödl S, Bökenkamp JE, Peker E, Zarges C, Faust J, Storchova Z, Räschle M et al (2023) The Orf9b protein of SARS-CoV-2 modulates mitochondrial protein biogenesis. J Cell Biol 222:e202303002 37682539 10.1083/jcb.202303002 PMC10491932

[CR22] Li J, Gui Q, Liang F-X, Sall J, Zhang Q, Duan Y, Dhabaria A, Askenazi M, Ueberheide B, Stapleford KA et al (2023a) The REEP5/TRAM1 complex binds SARS-CoV-2 NSP3 and promotes virus replication. J Virol 97:e0050723 37768083 10.1128/jvi.00507-23 PMC10617467

[CR23] Li W, Lu J, Xiao K, Zhou M, Li Y, Zhang X, Li Z, Gu L, Xu X, Guo Q et al (2023b) Integrated multimodality microscope for accurate and efficient target-guided cryo-lamellae preparation. Nat Methods 20:268–275 36646896 10.1038/s41592-022-01749-z PMC9911353

[CR24] Li Y, Li C, Zhao C, Wu J, Zhu Y, Wang F, Zhong J, Yan Y, Jin Y, Dong W et al (2024) Coronavirus M protein promotes mitophagy over virophagy by recruiting PDPK1 to phosphorylate SQSTM1 at T138. Nat Commun 15:8927 39414765 10.1038/s41467-024-53100-z PMC11484861

[CR25] Liu Y, Yan C, Cao B, Kong D, Li J, Li W, Guo Y, Yuan Z, Gao Y, Zhang Y et al (2025) Modulating mitochondrial dynamics in CMT2A: a multifaceted platform for drug discovery and evaluation. Biophys Rep 11:143–155 40612231 10.52601/bpr.2024.240037 PMC12213711

[CR26] López-Ayllón BD, Marin S, Fernández MF, García-García T, Fernández-Rodríguez R, de Lucas-Rius A, Redondo N, Mendoza-García L, Foguet C, Grigas J et al (2024) Metabolic and mitochondria alterations induced by SARS-CoV-2 accessory proteins ORF3a, ORF9b, ORF9c and ORF10. J Med Virol 96:e29752 38949191 10.1002/jmv.29752

[CR27] Perlman S, Netland J (2009) Coronaviruses post-SARS: update on replication and pathogenesis. Nat Rev Microbiol 7:439 19430490 10.1038/nrmicro2147 PMC2830095

[CR28] Pila-Castellanos I, Molino D, McKellar J, Lines L, da Graca J, Tauziet M, Chanteloup L, Mikaelian I, Meyniel-Schicklin L, Codogno P et al (2021) Mitochondrial morphodynamics alteration induced by influenza virus infection as a new antiviral strategy. PLoS Pathog 17:e1009340 33596274 10.1371/journal.ppat.1009340 PMC7920353

[CR29] Ramachandran K, Maity S, Muthukumar AR, Kandala S, Tomar D, Abd El-Aziz TM, Allen C, Sun Y, Venkatesan M, Madaris TR et al (2022) SARS-CoV-2 infection enhances mitochondrial PTP complex activity to perturb cardiac energetics. iScience 25:103722 35005527 10.1016/j.isci.2021.103722 PMC8720045

[CR30] Robb EL, Gawel JM, Aksentijević D, Cochemé HM, Stewart TS, Shchepinova MM, Qiang H, Prime TA, Bright TP, James AM et al (2015) Selective superoxide generation within mitochondria by the targeted redox cycler MitoParaquat. Free Radic Biol Med 89:883–894 26454075 10.1016/j.freeradbiomed.2015.08.021

[CR31] Robinson KM, Janes MS, Pehar M, Monette JS, Ross MF, Hagen TM, Murphy MP, Beckman JS (2006) Selective fluorescent imaging of superoxide in vivo using ethidium-based probes. Proc Natl Acad Sci USA 103:15038–15043 17015830 10.1073/pnas.0601945103 PMC1586181

[CR32] Rocha AG, Franco A, Krezel AM, Rumsey JM, Alberti JM, Knight WC, Biris N, Zacharioudakis E, Janetka JW, Baloh RH et al (2018) MFN2 agonists reverse mitochondrial defects in preclinical models of Charcot-Marie-Tooth disease type 2A. Science 360:336–341 29674596 10.1126/science.aao1785 PMC6109362

[CR33] Rowland AA, Voeltz GK (2012) Endoplasmic reticulum–mitochondria contacts: function of the junction. Nat Rev Mol Cell Biol 13:607 22992592 10.1038/nrm3440 PMC5111635

[CR34] Sakai Y, Kawachi K, Terada Y, Omori H, Matsuura Y, Kamitani W (2017) Two-amino acids change in the nsp4 of SARS coronavirus abolishes viral replication. Virology 510:165–174 28738245 10.1016/j.virol.2017.07.019 PMC7111695

[CR35] Shang C, Liu Z, Zhu Y, Lu J, Ge C, Zhang C, Li N, Jin N, Li Y, Tian M et al (2022) SARS-CoV-2 causes mitochondrial dysfunction and mitophagy impairment. Front Microbiol 12:780768 35069483 10.3389/fmicb.2021.780768 PMC8770829

[CR36] Shiiba I, Ito N, Oshio H, Ishikawa Y, Nagao T, Shimura H, Oh KW, Takasaki E, Yamaguchi F, Konagaya R et al (2025) ER-mitochondria contacts mediate lipid radical transfer via RMDN3/PTPIP51 phosphorylation to reduce mitochondrial oxidative stress. Nat Commun 16:1–18 39929810 10.1038/s41467-025-56666-4 PMC11811300

[CR37] Su S, Wong G, Shi W, Liu J, Lai ACK, Zhou J, Liu W, Bi Y, Gao GF (2016) Epidemiology, genetic recombination, and pathogenesis of coronaviruses. Trends Microbiol 24:490 27012512 10.1016/j.tim.2016.03.003 PMC7125511

[CR38] V’kovski P, Kratzel A, Steiner S, Stalder H, Thiel V (2021) Coronavirus biology and replication: implications for SARS-CoV-2. Nat Rev Microbiol 19:155–170 33116300 10.1038/s41579-020-00468-6 PMC7592455

[CR39] Williams CG, Jureka AS, Silvas JA, Nicolini AM, Chvatal SA, Carlson-Stevermer J, Oki J, Holden K, Basler CF (2021) Inhibitors of VPS34 and fatty-acid metabolism suppress SARS-CoV-2 replication. Cell Rep 36:109479 34320401 10.1016/j.celrep.2021.109479 PMC8289695

[CR40] Williams JM, Chen YJ, Cho WJ, Tai AW, Tsai B (2023) Reticulons promote formation of ER-derived double-membrane vesicles that facilitate SARS-CoV-2 replication. J Cell Biol 222:e202203060 37093123 10.1083/jcb.202203060 PMC10130743

[CR41] Wolff G, Melia CE, Snijder EJ, Bárcena M (2020) Double-membrane vesicles as platforms for viral replication. Trends Microbiol 28:1022–1033 32536523 10.1016/j.tim.2020.05.009 PMC7289118

[CR42] Xiao CX, Yang XN, Huang QW, Zhang YQ, Lin BY, Liu JJ, Liu YP, Jazag A, Guleng B, Ren JL (2013) ECHS1 acts as a novel HBsAg-binding protein enhancing apoptosis through the mitochondrial pathway in HepG2 cells. Cancer Lett 330:67–73 23178449 10.1016/j.canlet.2012.11.030

[CR43] Yang J, Tian B, Wang P, Chen R, Xiao K, Long X, Zheng X, Zhu Y, Sun F, Shi Y et al (2025) SARS-CoV-2 NSP3/4 control formation of replication organelle and recruitment of RNA polymerase NSP12. J Cell Biol 224:e202306101 39737877 10.1083/jcb.202306101 PMC11687299

[CR44] Yang Y, Wu Y, Meng X, Wang Z, Younis M, Liu Y, Wang P, Huang X (2022) SARS-CoV-2 membrane protein causes the mitochondrial apoptosis and pulmonary edema via targeting BOK. Cell Death Differ 29:1395–1408 35022571 10.1038/s41418-022-00928-x PMC8752586

[CR45] Zhang YK, Qu YY, Lin Y, Wu XH, Chen HZ, Wang X, Zhou KQ, Wei Y, Guo F, Yao CF et al (2017) Enoyl-CoA hydratase-1 regulates mTOR signaling and apoptosis by sensing nutrients. Nat Commun 8:1–16 28878358 10.1038/s41467-017-00489-5 PMC5587591

[CR46] Zhou M, Kong B, Zhang X, Xiao K, Lu J, Li W, Li M, Li Z, Ji W, Hou J et al (2023) A proximity labeling strategy enables proteomic analysis of inter-organelle membrane contacts. iScience 26:107159 37485370 10.1016/j.isci.2023.107159 PMC10362359

[CR47] Zhou M, Yang J, Tian B, Wang X, Chen Z, Yang H, Kong B, Xiao L, Li Z (2025) Spatial proteomics using BiFCPL identifies regulators of DMV formation involved in coronavirus replication. J Med Virol 97:e70547 40838854 10.1002/jmv.70547

[CR48] Zimmermann L, Zhao X, Makroczyova J, Wachsmuth-Melm M, Prasad V, Hensel Z, Bartenschlager R, Chlanda P (2023) SARS-CoV-2 nsp3 and nsp4 are minimal constituents of a pore spanning replication organelle. Nat Commun 14:7894 38036567 10.1038/s41467-023-43666-5 PMC10689437

[CR49] Zong S, Wu Y, Li W, You Q, Peng Q, Wang C, Wan P, Bai T, Ma Y, Sun B et al (2023) SARS-CoV-2 Nsp8 induces mitophagy by damaging mitochondria. Virol Sin 38:520–530 37156297 10.1016/j.virs.2023.05.003 PMC10163945

