# Supplementary material for: Disruption of ER-mitochondria contact sites by coronavirus replication organelles sustains viral replication via NSP3 stabilization
Source: EMBO J. 2026 May 28;45(13):4379–416. doi: 10.1038/s44318-026-00816-x (PMC13323368; doi:10.1038/s44318-026-00816-x)
Supplement: Supplementary file 1 — Table EV1 [file 44318_2026_816_MOESM1_ESM.docx]

**Table EV1. List of qPCR primers.**

| **Gene** | **Sequence (5′ >>> 3′)** | |
| --- | --- | --- |
| WIV1 *RdRp* | Forward | GGTCATGTGTGGCGGCTC |
|  | Reverse | GCTGTAACAGCTTGACAAATGTTAAAG |
| SARS-CoV-2 *N* | Forward | CACATTGGCACCCGCAATC |
|  | Reverse | GAGGAACGAGAAGAGGCTTG |
| MHV *N* | Forward | TGGAAGGTCTGCACCTGCTA |
|  | Reverse | TTTGGCCCACGGGATTG |
| Human *GAPDH* | Forward | GTCTCCTCTGACTTCAACAGCG |
|  | Reverse | ACCACCCTGTTGCTGTAGCCAA |
| Mouse *GAPDH* | Forward | CATCACTGCCACCCAGAAGACTG |
|  | Reverse | ATGCCAGTGAGCTTCCCGTTCAG |
| Human *ECHS1* | Forward | GCGATGGAGATGGTCCTCAC |
|  | Reverse | TCTTGCTGACAAGACCTGCT |
| Human *DLD* | Forward | GGACTTTCTGCAGTGCCTCT |
|  | Reverse | CAATGCAGACTGTCTTGAAGC |
| Human *ATP5F1C* | Forward | GGTGCTGCAGCTCTGGATTA |
|  | Reverse | TTCTTCGGACAAAGGCAGCA |
| Human *ACAT1* | Forward | GCAGTATTGGGTGCAGGCTT |
|  | Reverse | CTGCCACCATCACATCCTGA |
| Human *IDH3A* | Forward | CCACACCTCTCATCCGCTG |
|  | Reverse | AGCCGAGAGACCTGGGAAA |
| Human *LONP1* | Forward | GGTTGCTCCGAGGCCC |
|  | Reverse | CTTGATAAAGCGCGGGAACA |
| Human *MDH2* | Forward | GGACGACCTGTTCAACACCA |
|  | Reverse | GCTGGATCCAAACCCGGA |
| Human *PC* | Forward | GACGGCGAGGAGATAGTGTC |
|  | Reverse | GGACTGTTCGGAACTTCAGC |
| Human *PMPCB* | Forward | CCATCCCTGAGCTTGAAGCAA |
|  | Reverse | GCTTAATGGGACCAACAGCA |
| Human *PRDX3* | Forward | AATACACCAAGAAAGAATGGTGG |
|  | Reverse | TGATCTTAGTGCAAGACCAGAA |
| Human *SUCLG1* | Forward | CTTTGTGCGTTGGCATTGGA |
|  | Reverse | GCCTTCTGTGGCAGAATCGT |
| Human *SUCLG2* | Forward | TGGTCCGGCTTGAAGGTACT |
|  | Reverse | GATTTTGGTGGGCGACGTTC |
| Human *TIMM44* | Forward | GTGTTTGAGCCAAACGAGGAG |
|  | Reverse | GCGTTGTCGCTTTCGTCATA |
