# Supplementary material for: Disruption of ER-mitochondria contact sites by coronavirus replication organelles sustains viral replication via NSP3 stabilization
Source: EMBO J. 2026 May 28;45(13):4379–416. doi: 10.1038/s44318-026-00816-x (PMC13323368; doi:10.1038/s44318-026-00816-x)
Supplement: Supplementary file 2 — Table EV2 [file 44318_2026_816_MOESM2_ESM.docx]

**Table EV2. List of siRNA sequences**

| **Gene** | **siRNA(5′ >>> 3′)** |
| --- | --- |
| Human *ACAT1* | GCCUUUAGUCUGGUUGUACUATT |
| Human *ATP5F1C* | GCUUCUGAGAUGAUUGACAAATT |
| Human *BCAT2* | GUGCACCGAAUCCUGUACAAATT |
| Human *DLD-1* | GCAAAUCUUGCUGCGUCAUUUTT |
| Human *ECHS1* | CCUGAGUUUCCAGGACUGUUATT |
| Human *IDH3A* | GCUCAGACUUCACAGAGGAAATT |
| Human *LONP1* | CCAGCCUUAUGUCGGCGUCUUTT |
| Human *MDH2* | GACGACCUGUUCAACACCAAUTT |
| Human *PC* | GAUGCACCGGCAGAAAGCAGAUGAA |
| Human *PMPCB* | GAUCCCAGUUAGAUCUGGAACUUGA |
| Human *PRDX3* | AGCAAAUGUGAAUCGUAUUAUTT |
| Human *SUCLG1* | UGGAAUGGAUCACGUAGACAUTT |
| Human *SUCLG2* | AGGCUGCUAAGAGACUAAAUGCAAA |
| Human *TIMM44* | CCUGUUCUCCAAGACAGAGAUTT |
| NC | UUCUCCGAACGUGUCACGUTT |
