# Supplementary figures and images for: Disruption of ER-mitochondria contact sites by coronavirus replication organelles sustains viral replication via NSP3 stabilization (part 1 of 2)
Source: EMBO J. 2026 May 28;45(13):4379–416. doi: 10.1038/s44318-026-00816-x (PMC13323368; doi:10.1038/s44318-026-00816-x)

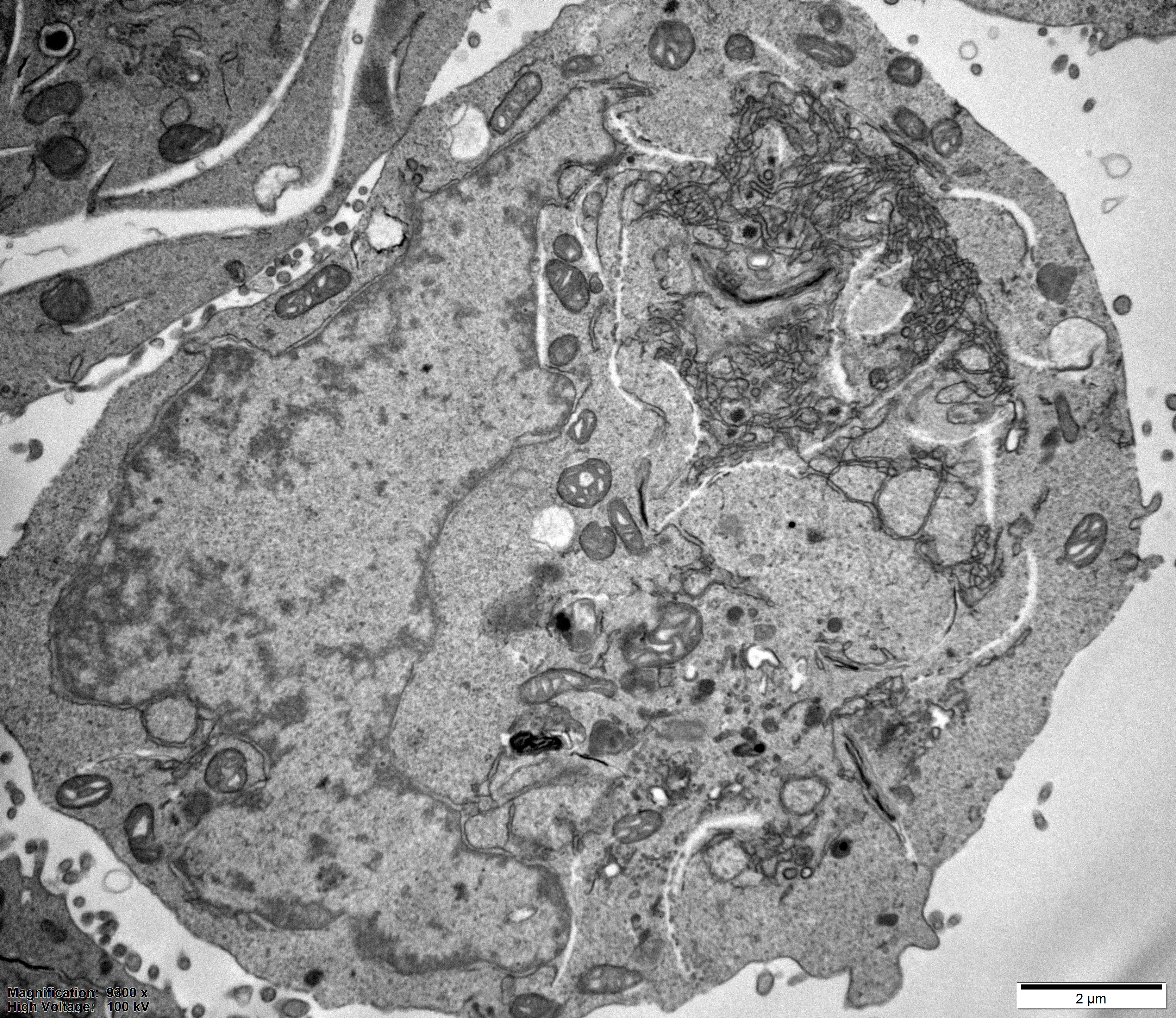

Supplement: Supplementary file 4 — Source data Fig. 1 [file 44318_2026_816_MOESM4_ESM.zip › A/Figure 1A GFP-NSP3-T2A-NSP4.tif]

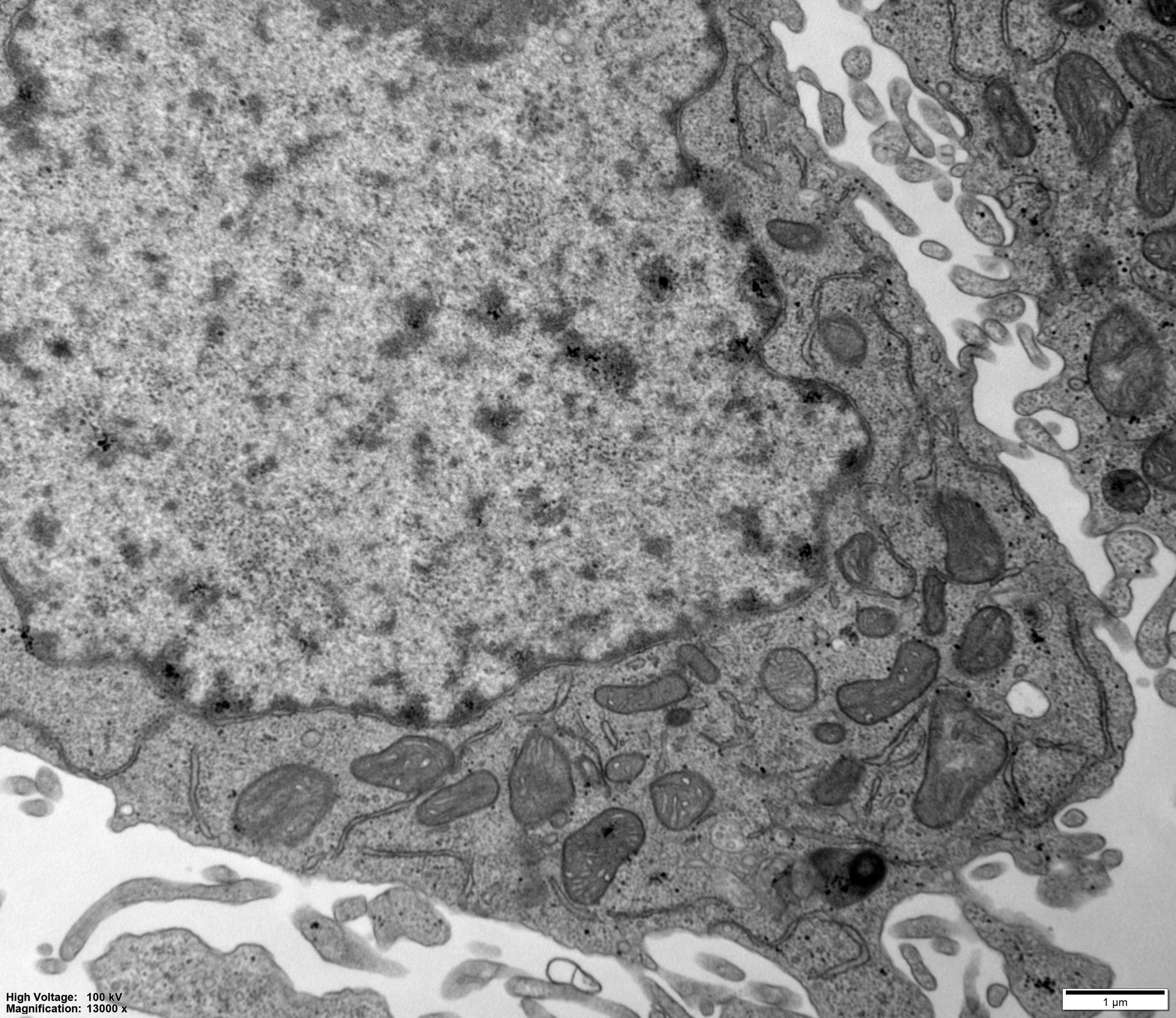

Supplement: Supplementary file 4 — Source data Fig. 1 [file 44318_2026_816_MOESM4_ESM.zip › A/Figure 1A GFP-NSP3.tif]

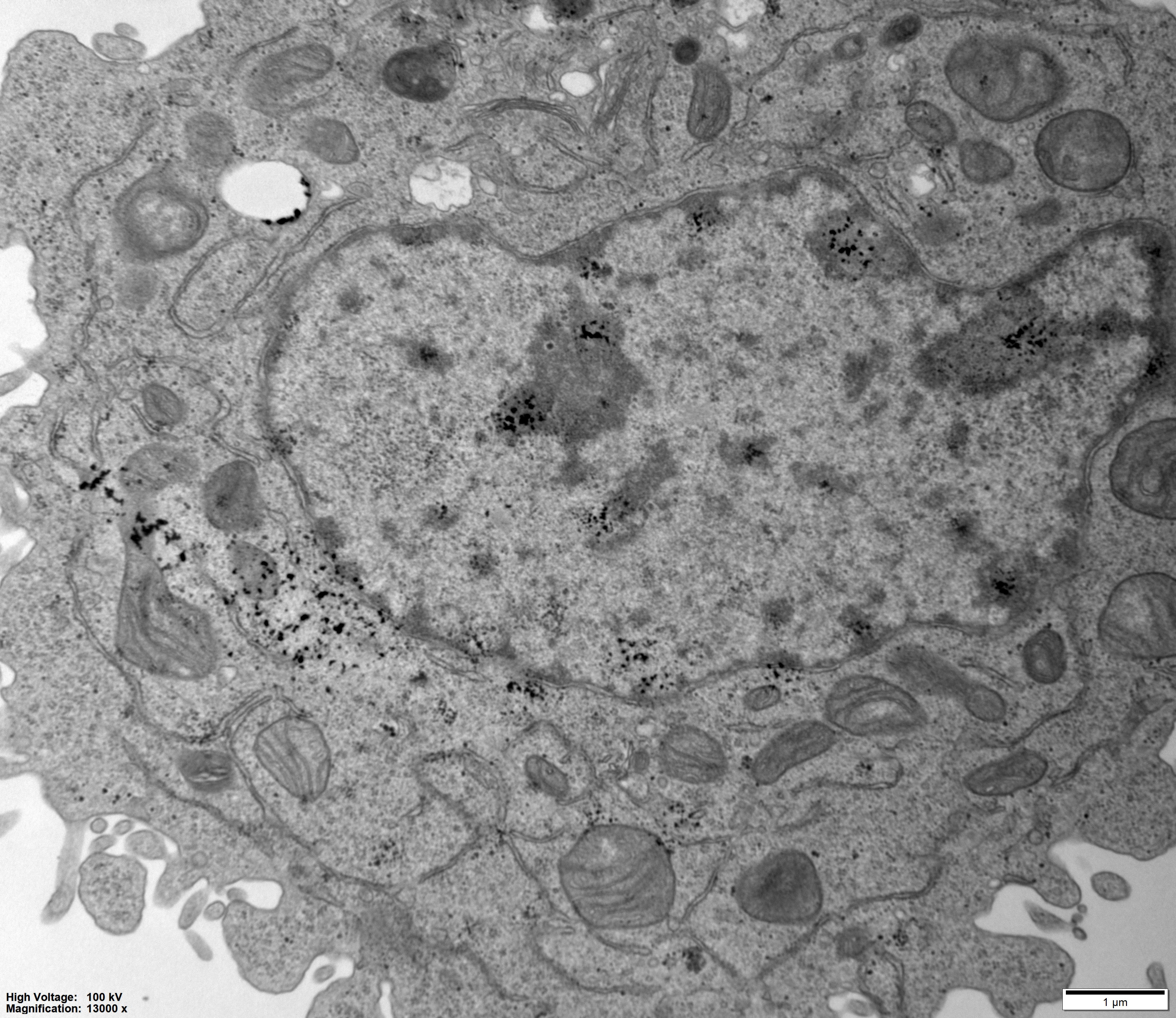

Supplement: Supplementary file 4 — Source data Fig. 1 [file 44318_2026_816_MOESM4_ESM.zip › A/Figure 1A GFP-NSP4.tif]

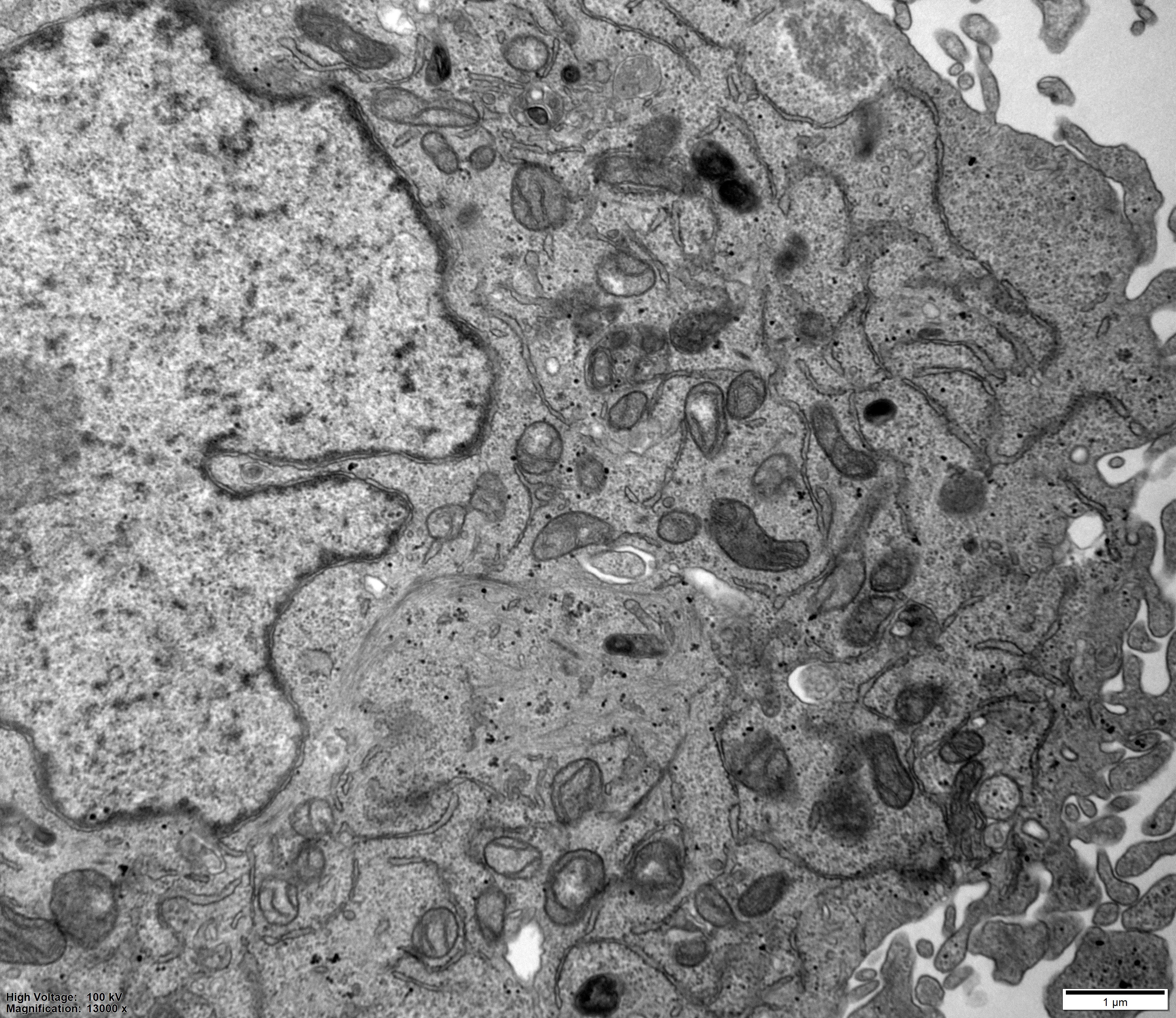

Supplement: Supplementary file 4 — Source data Fig. 1 [file 44318_2026_816_MOESM4_ESM.zip › A/Figure1A-GFP.tif]

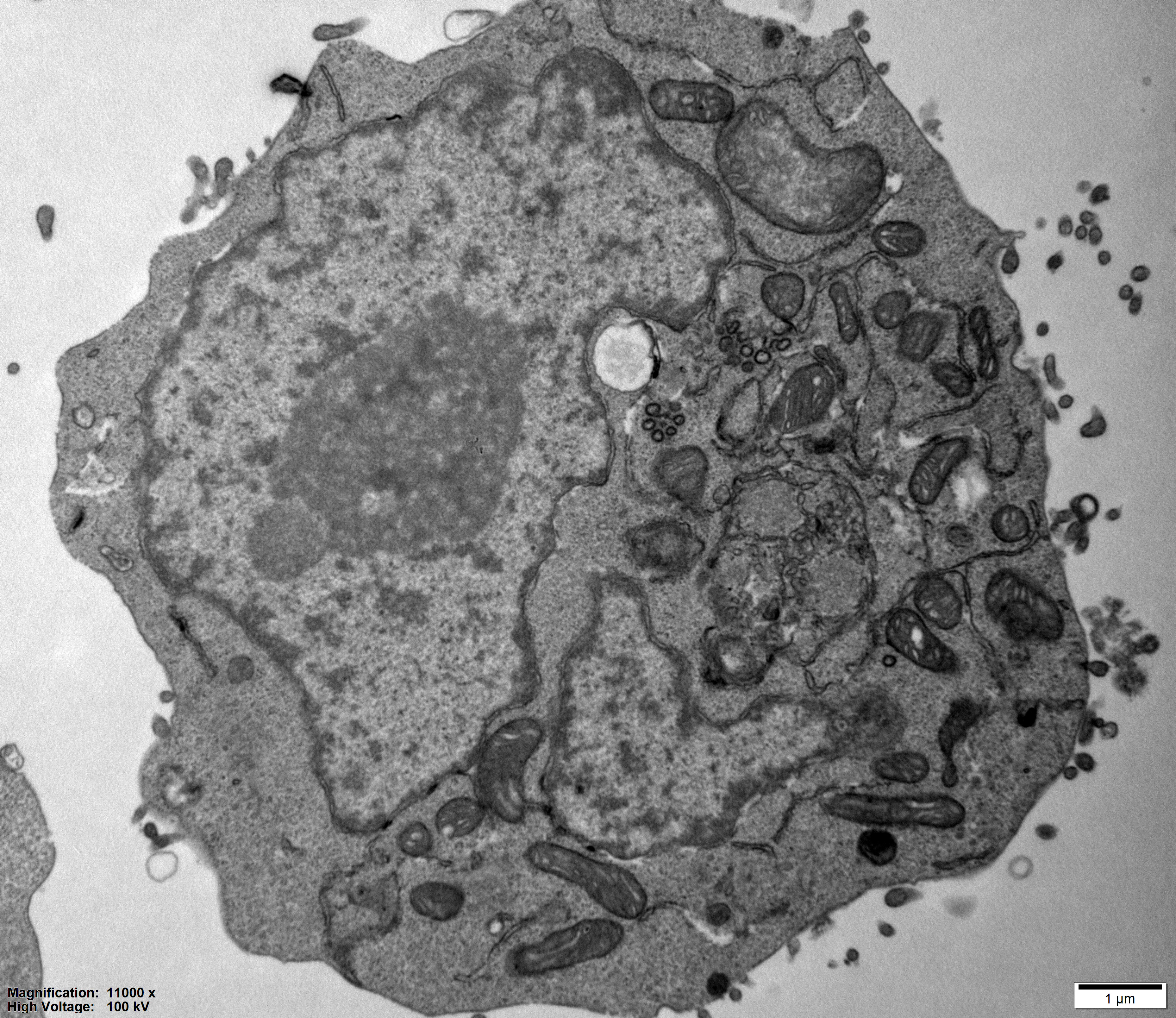

Supplement: Supplementary file 4 — Source data Fig. 1 [file 44318_2026_816_MOESM4_ESM.zip › D/1 μg GFP-NSP3-T2A-NSP4.tif]

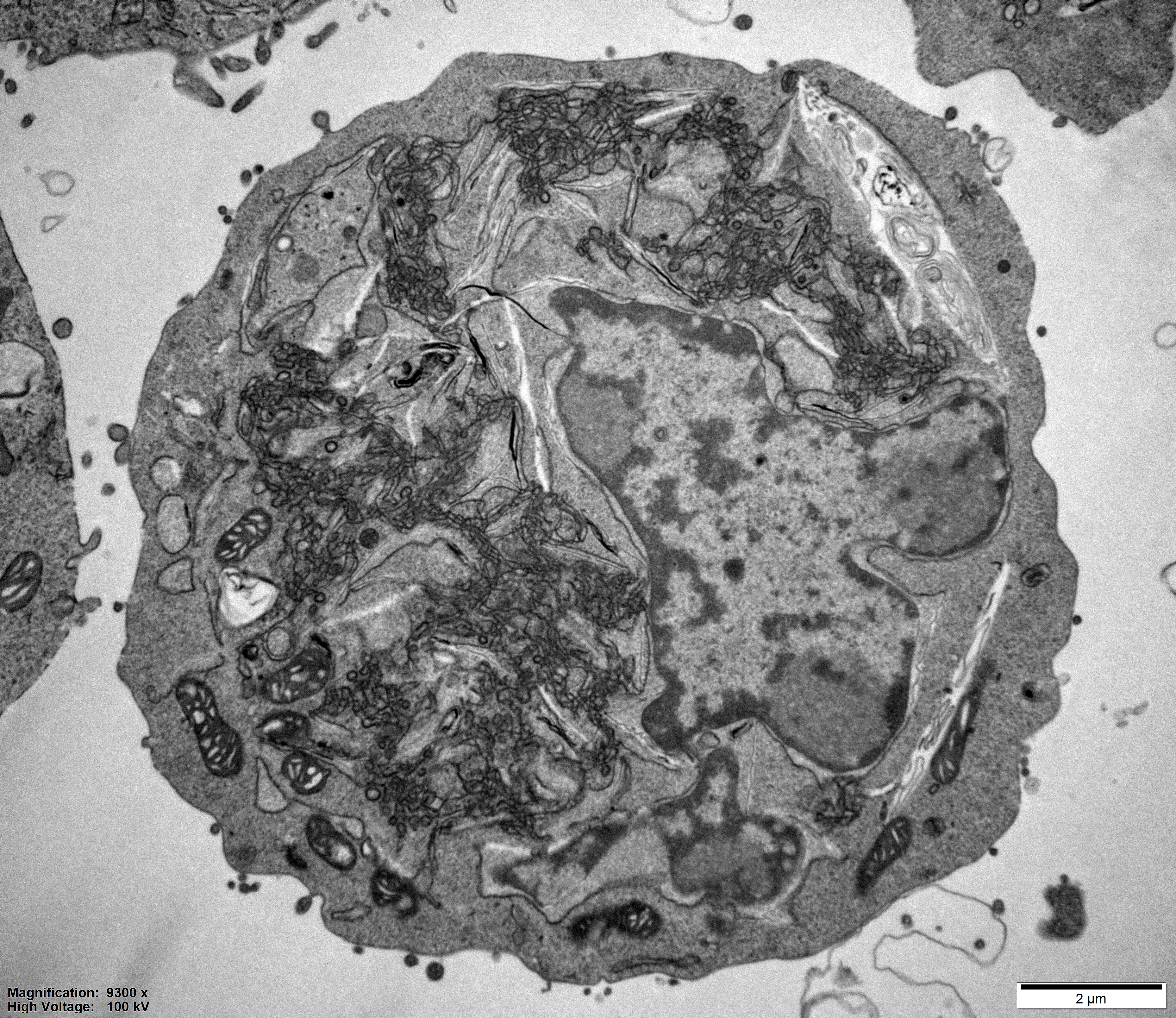

Supplement: Supplementary file 4 — Source data Fig. 1 [file 44318_2026_816_MOESM4_ESM.zip › D/4 μg GFP-NSP3-T2A-NSP4.tif]

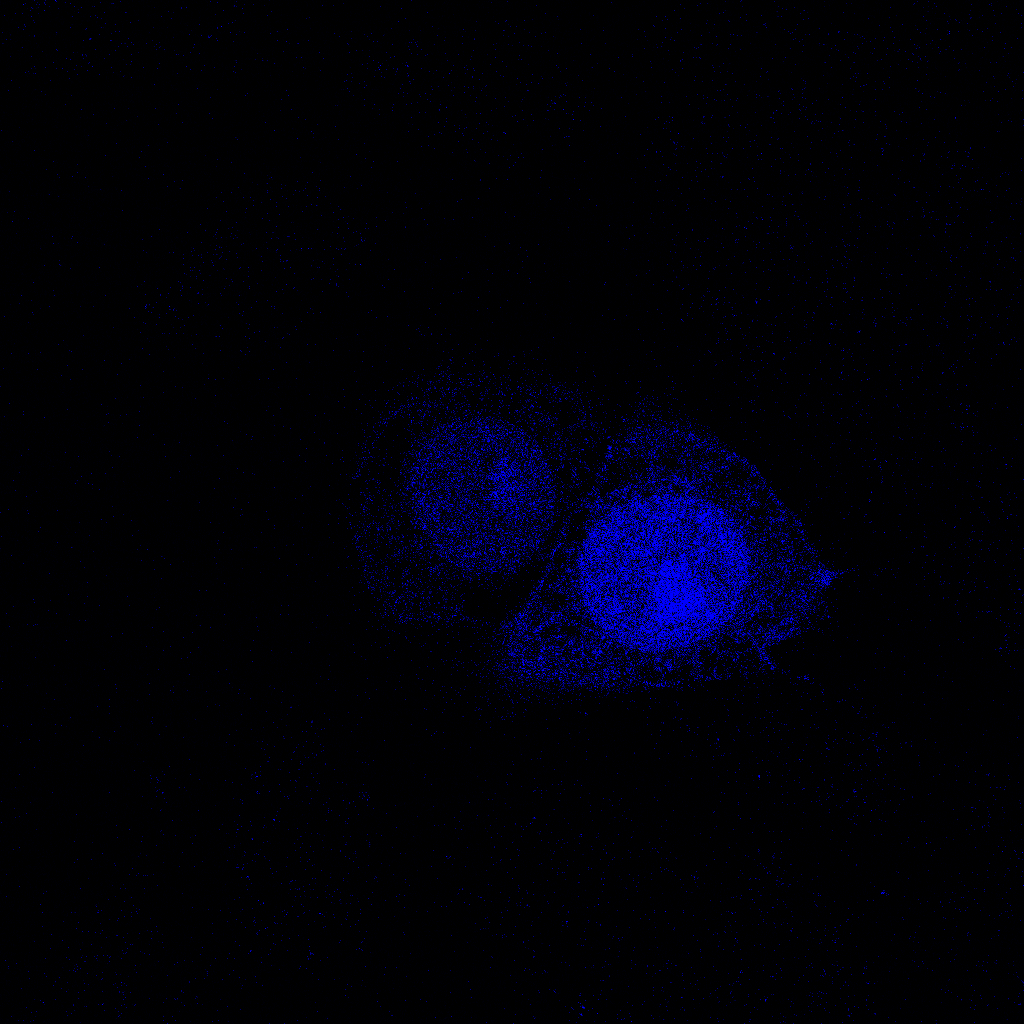

Supplement: Supplementary file 4 — Source data Fig. 1 [file 44318_2026_816_MOESM4_ESM.zip › G/ctrl-BFP.tif]

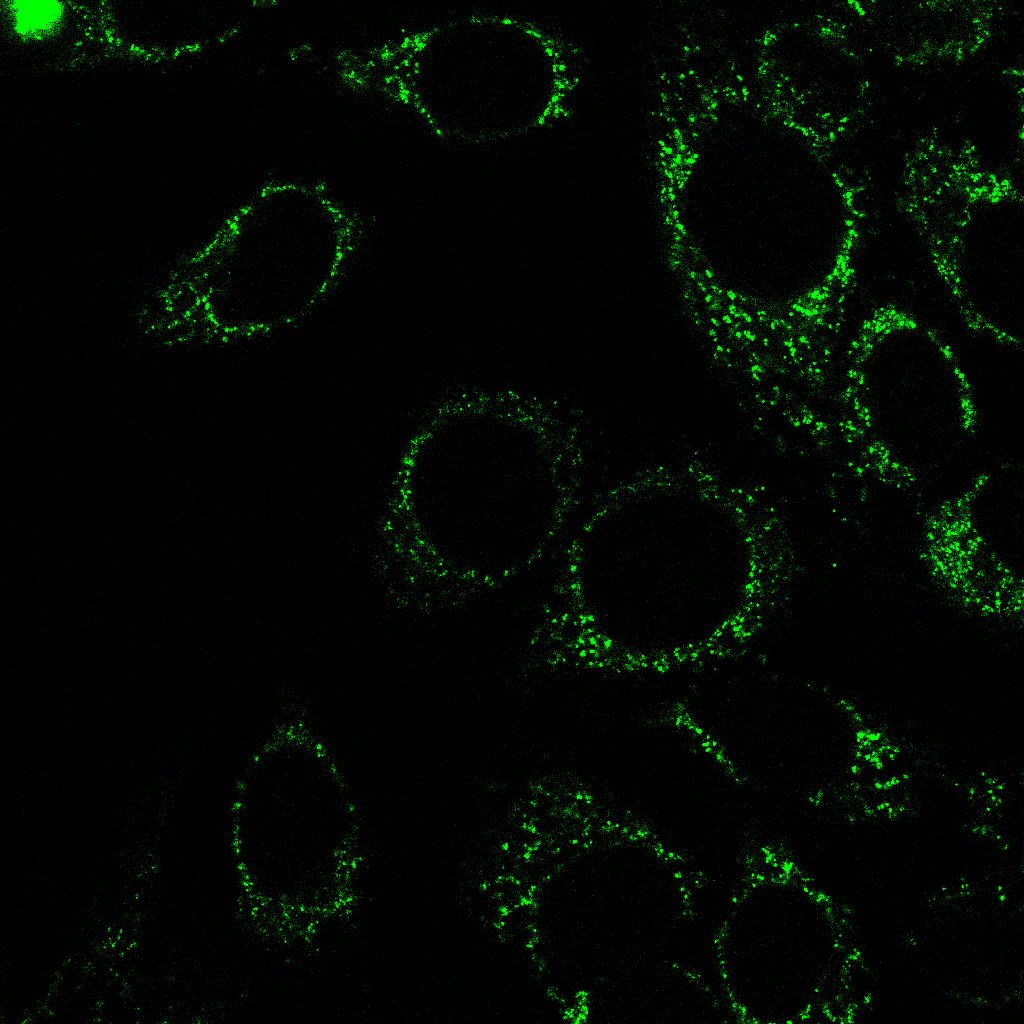

Supplement: Supplementary file 4 — Source data Fig. 1 [file 44318_2026_816_MOESM4_ESM.zip › G/ctrl-ERMCSs.tif]

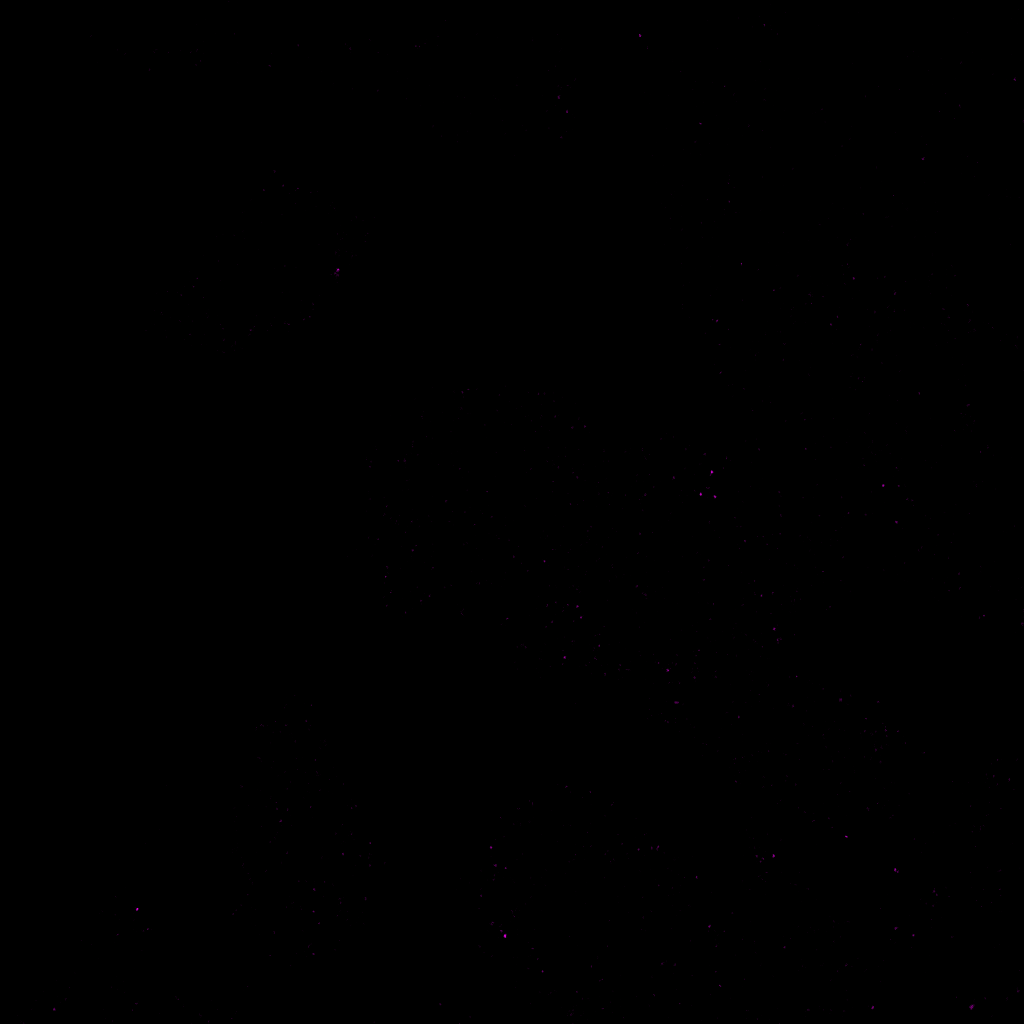

Supplement: Supplementary file 4 — Source data Fig. 1 [file 44318_2026_816_MOESM4_ESM.zip › G/ctrl-Flag.tif]

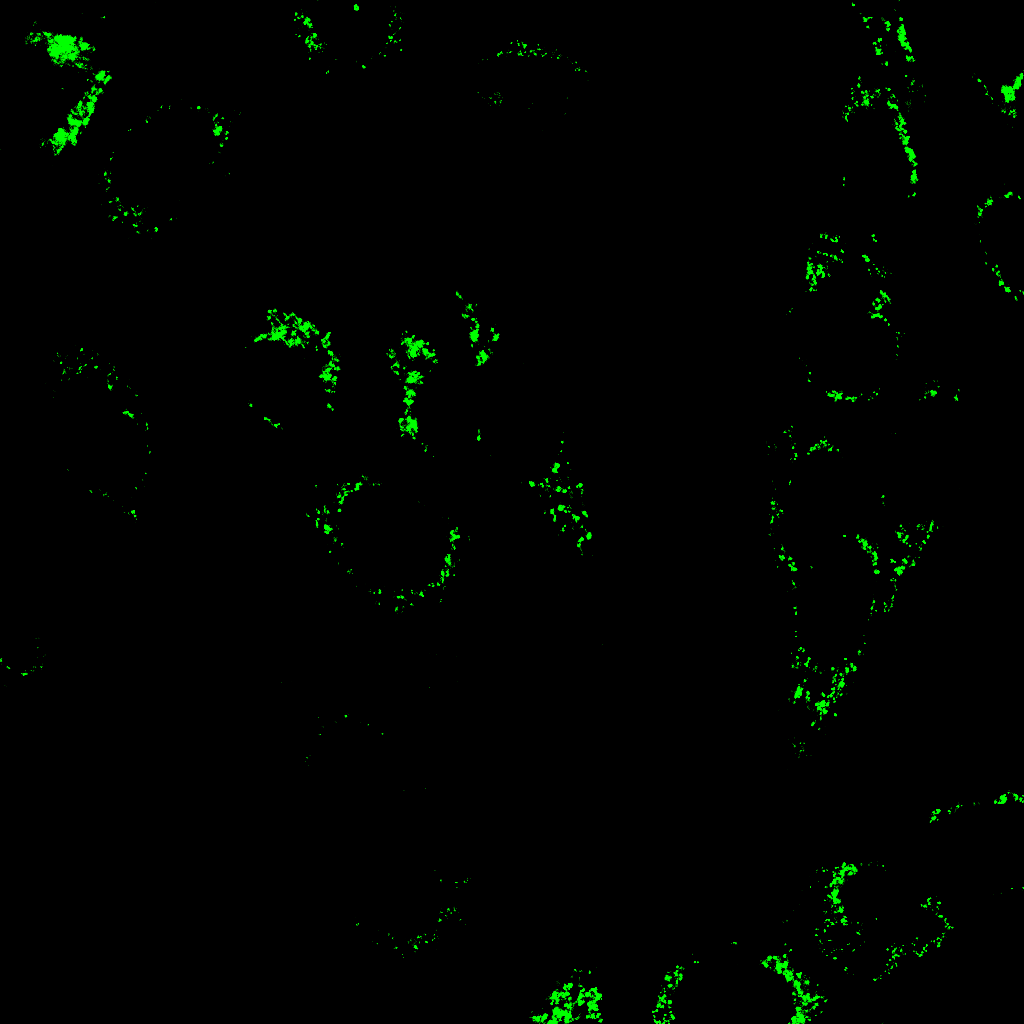

Supplement: Supplementary file 4 — Source data Fig. 1 [file 44318_2026_816_MOESM4_ESM.zip › G/NSP3+NSP4-ERMCSs.tif]

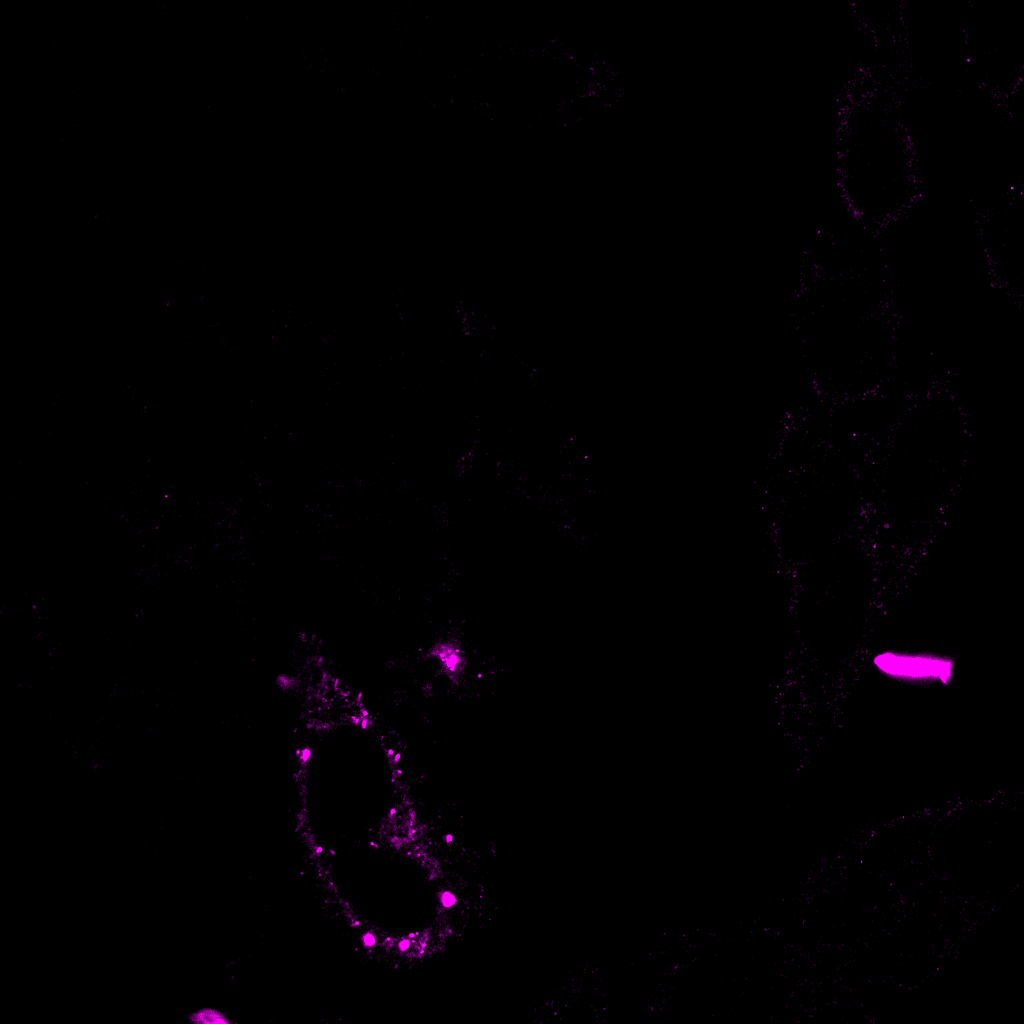

Supplement: Supplementary file 4 — Source data Fig. 1 [file 44318_2026_816_MOESM4_ESM.zip › G/NSP3+NSP4-Flag-NSP3.tif]

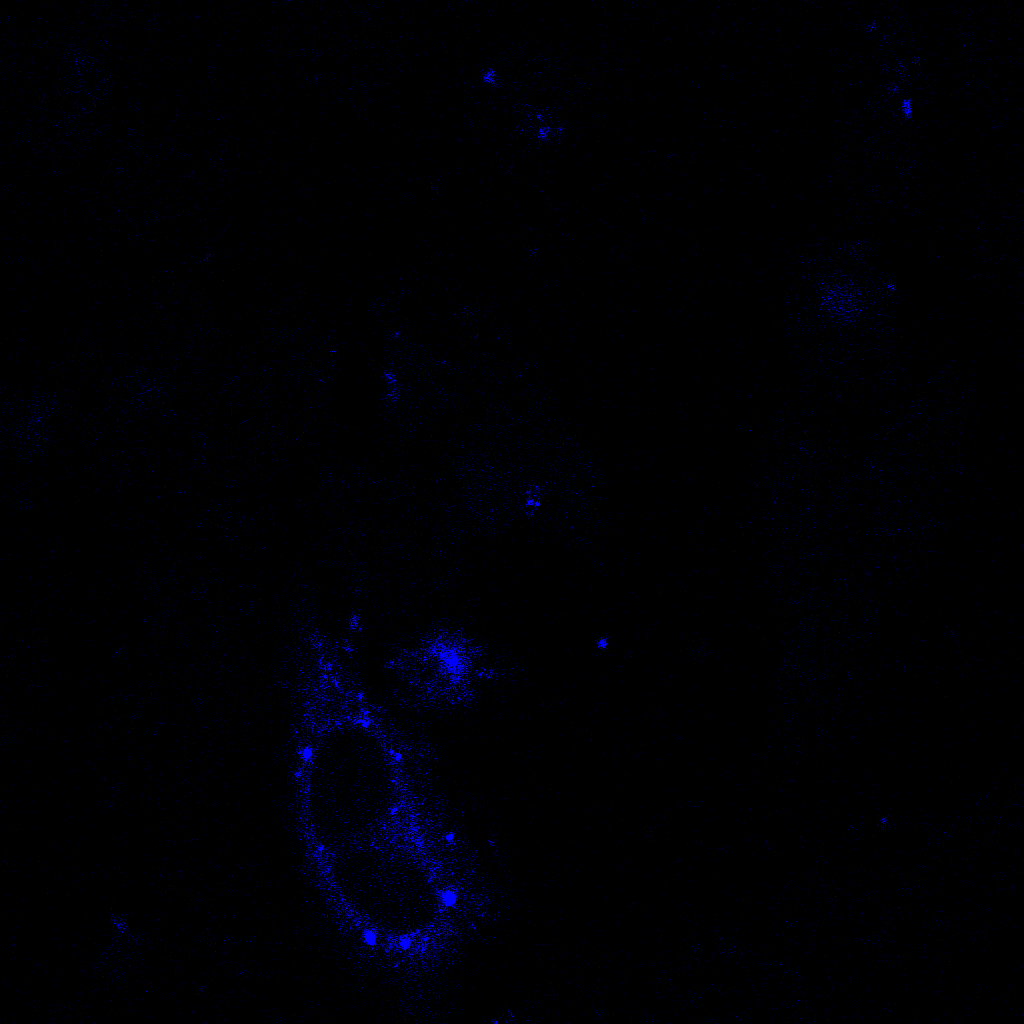

Supplement: Supplementary file 4 — Source data Fig. 1 [file 44318_2026_816_MOESM4_ESM.zip › G/NSP3+NSP4-NSP4-BFP.tif]

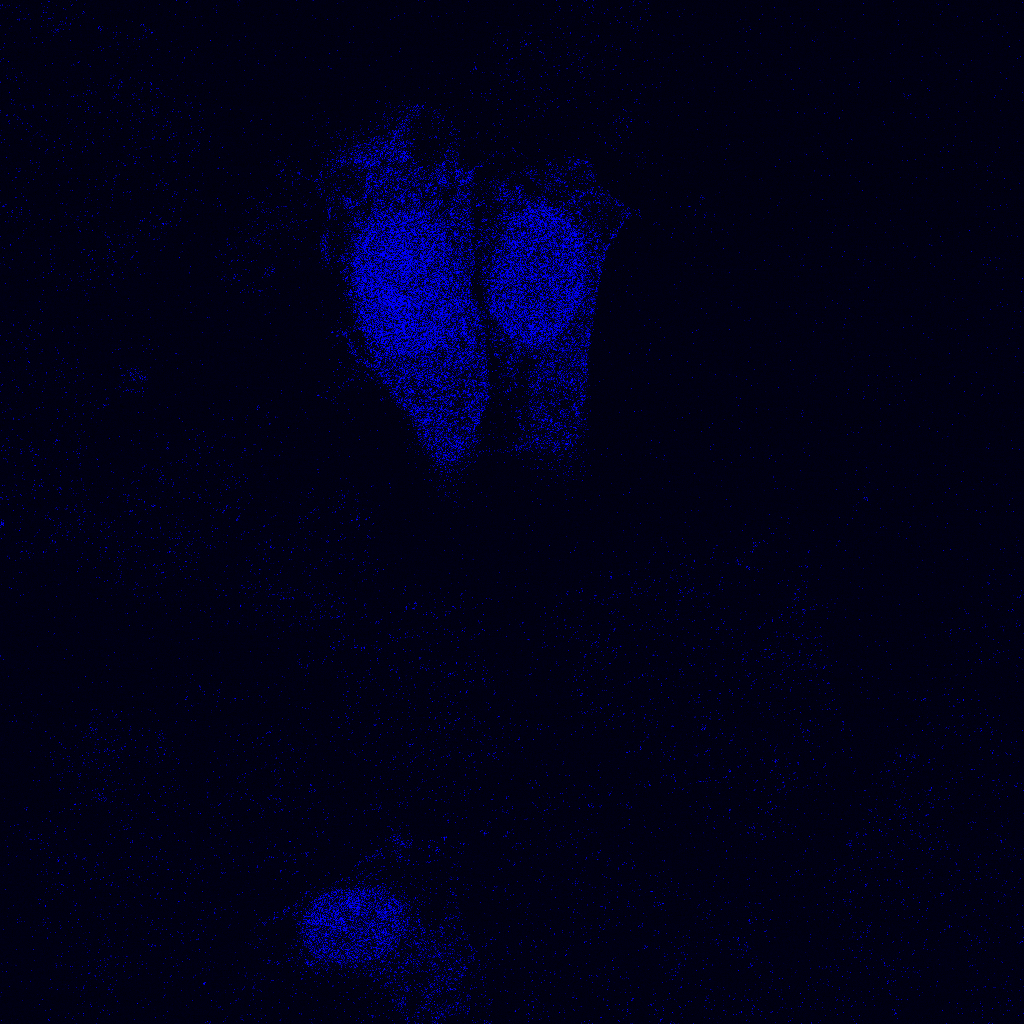

Supplement: Supplementary file 4 — Source data Fig. 1 [file 44318_2026_816_MOESM4_ESM.zip › G/NSP3-BFP.tif]

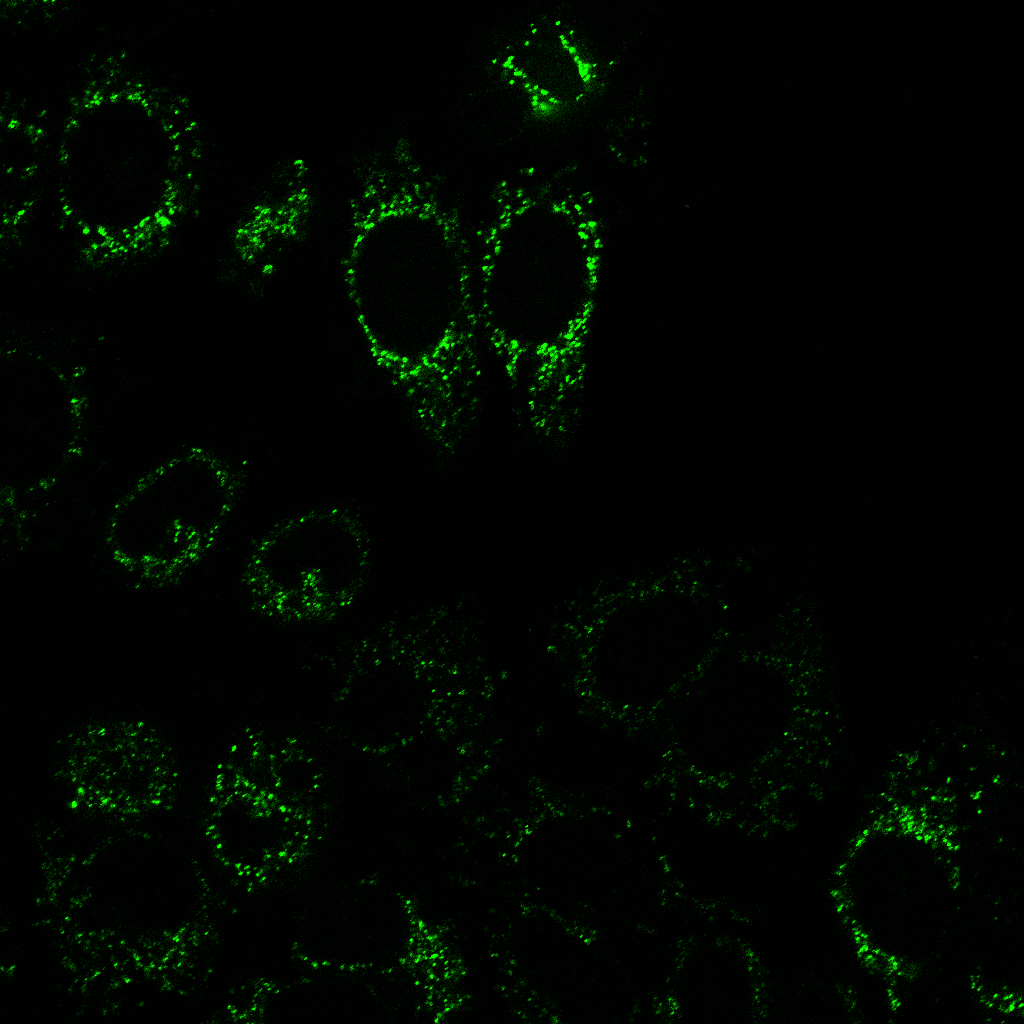

Supplement: Supplementary file 4 — Source data Fig. 1 [file 44318_2026_816_MOESM4_ESM.zip › G/NSP3-ERMCSs.tif]

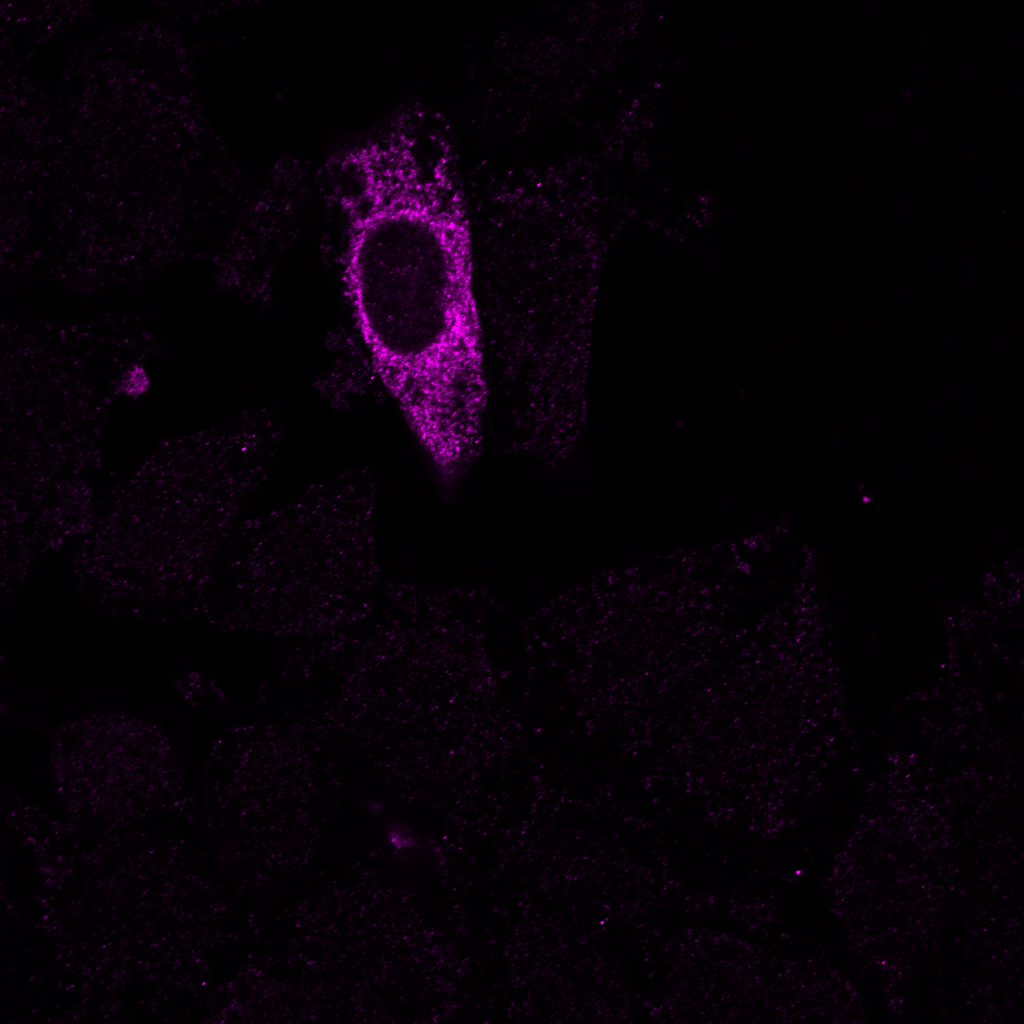

Supplement: Supplementary file 4 — Source data Fig. 1 [file 44318_2026_816_MOESM4_ESM.zip › G/NSP3-Flag NSP3.tif]

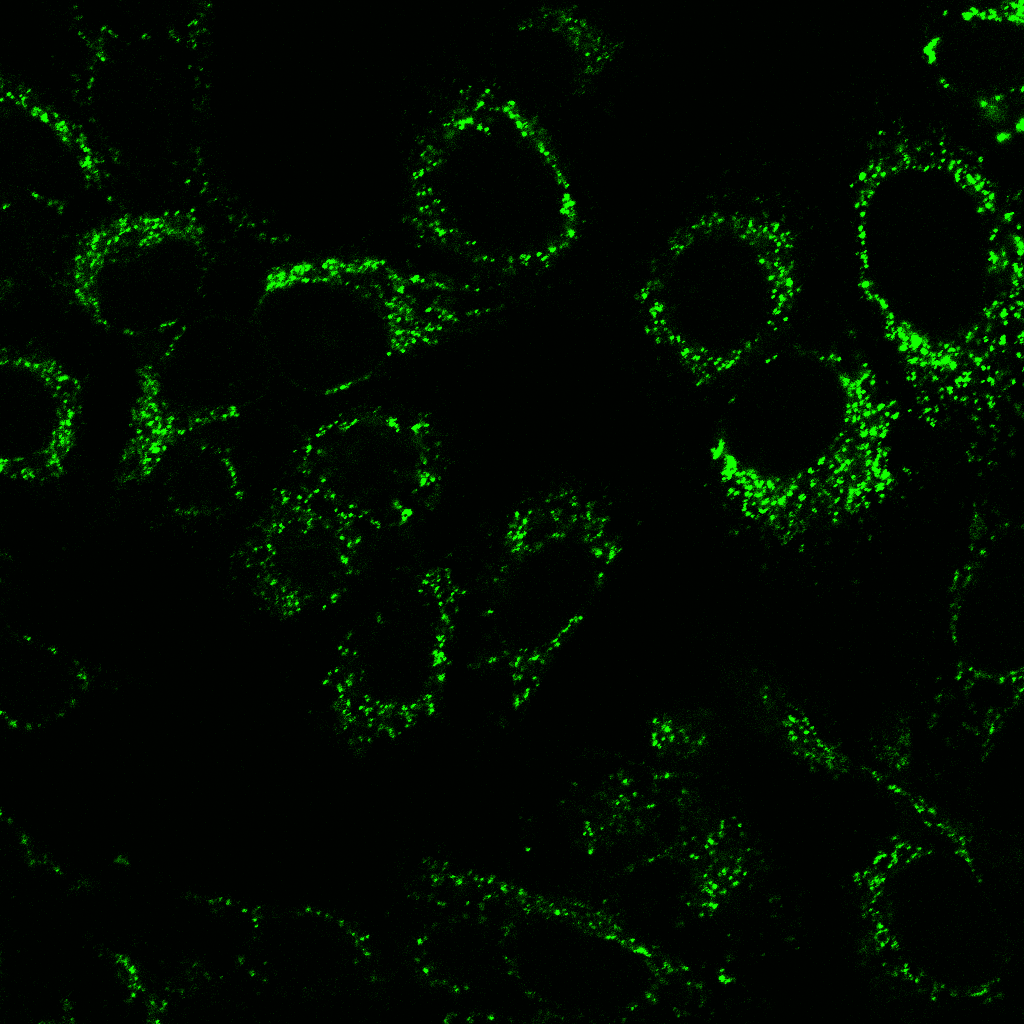

Supplement: Supplementary file 4 — Source data Fig. 1 [file 44318_2026_816_MOESM4_ESM.zip › G/NSP4-ERMCSs.tif]

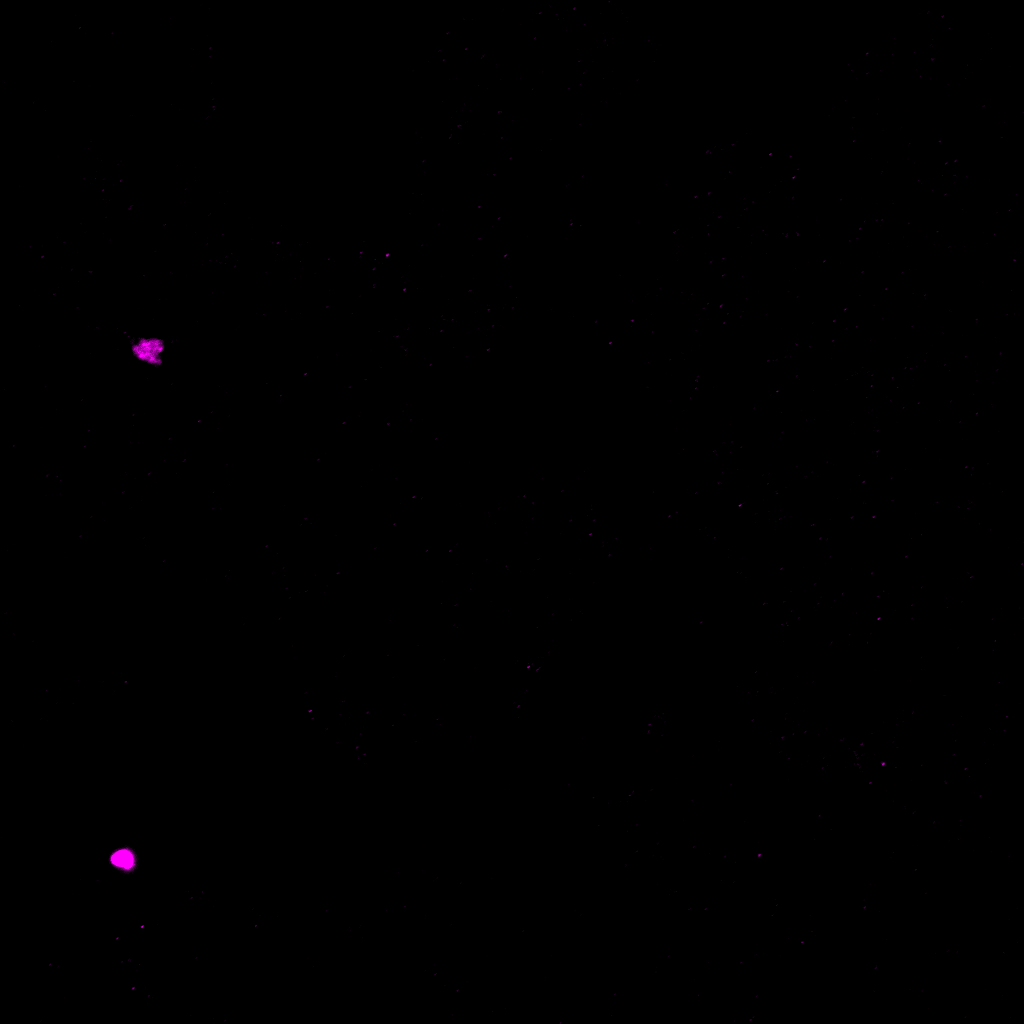

Supplement: Supplementary file 4 — Source data Fig. 1 [file 44318_2026_816_MOESM4_ESM.zip › G/NSP4-Flag.tif]

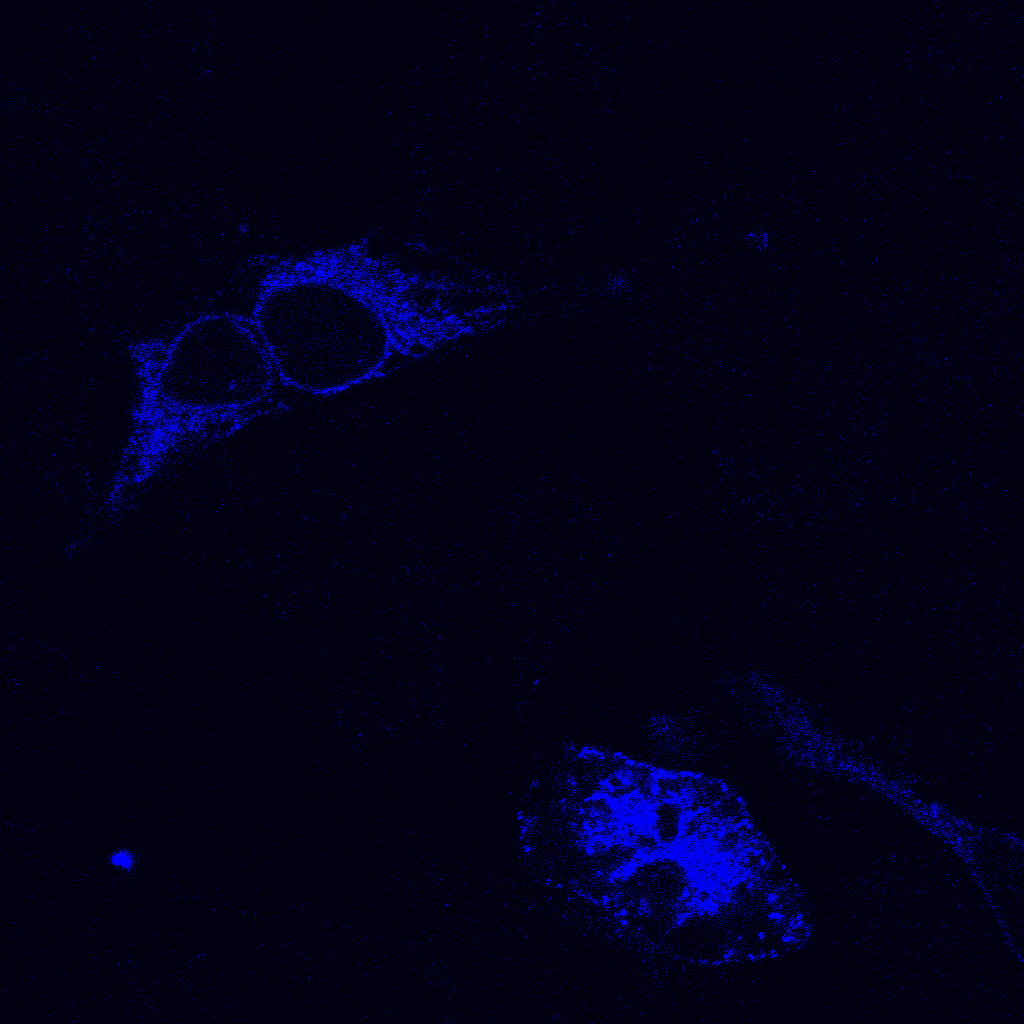

Supplement: Supplementary file 4 — Source data Fig. 1 [file 44318_2026_816_MOESM4_ESM.zip › G/NSP4-NSP4-BFP.tif]

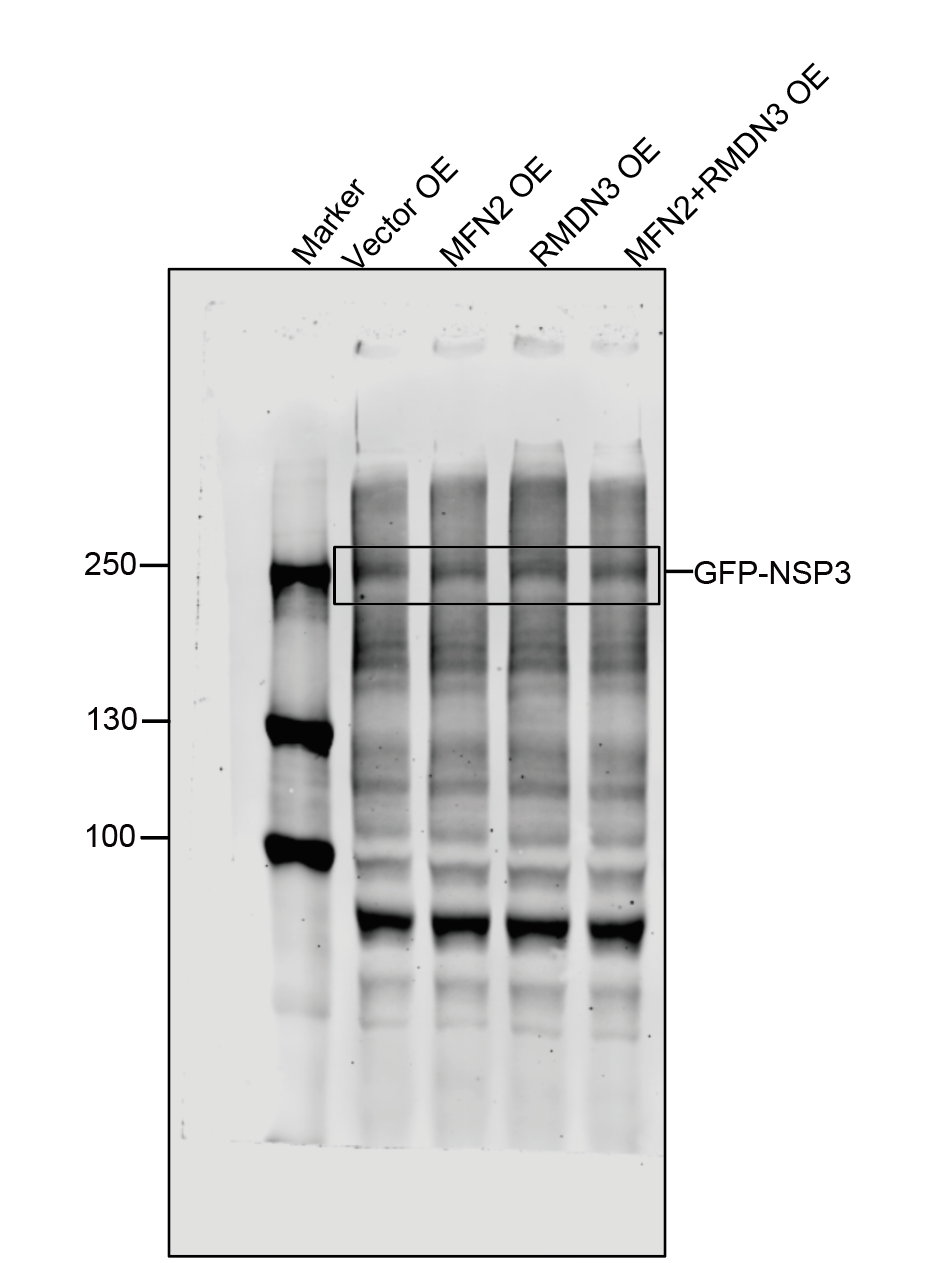

Supplement: Supplementary file 5 — Source data Fig. 2 [file 44318_2026_816_MOESM5_ESM.zip › A/GFP-NSP3.tif]

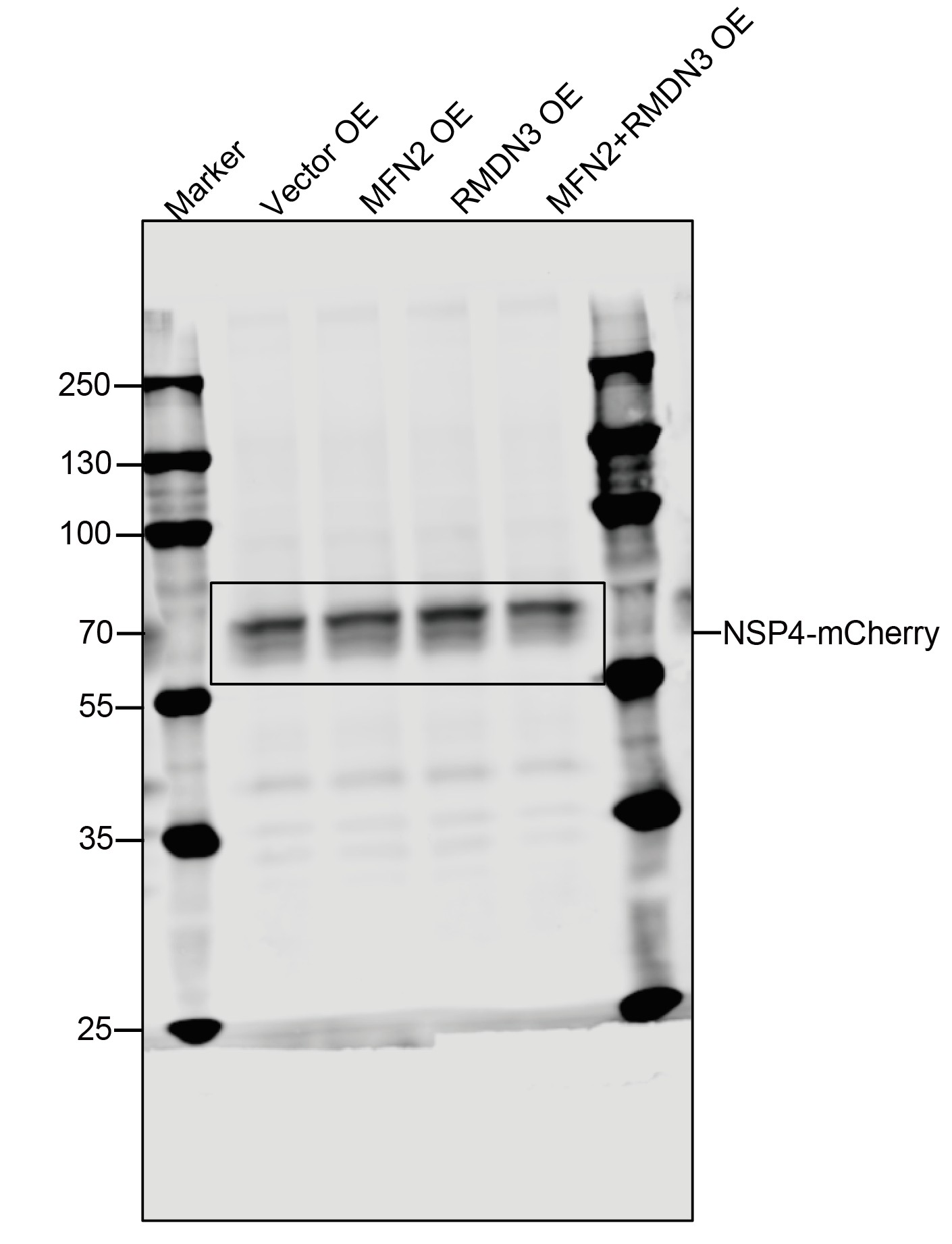

Supplement: Supplementary file 5 — Source data Fig. 2 [file 44318_2026_816_MOESM5_ESM.zip › A/NSP4-mCherry.tif]

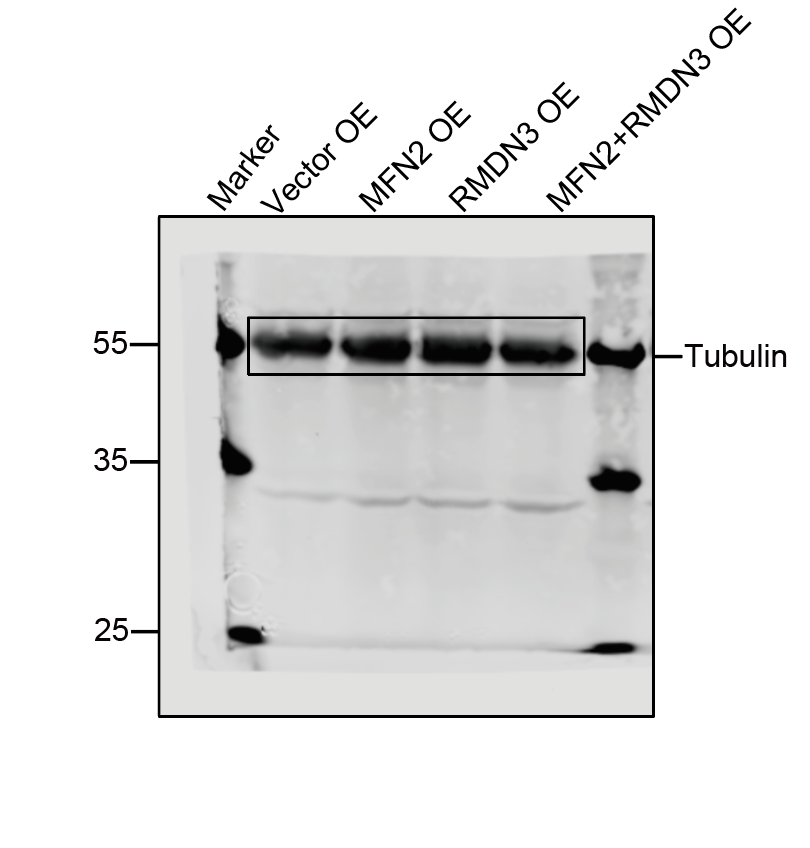

Supplement: Supplementary file 5 — Source data Fig. 2 [file 44318_2026_816_MOESM5_ESM.zip › A/tubulin-1.tif]

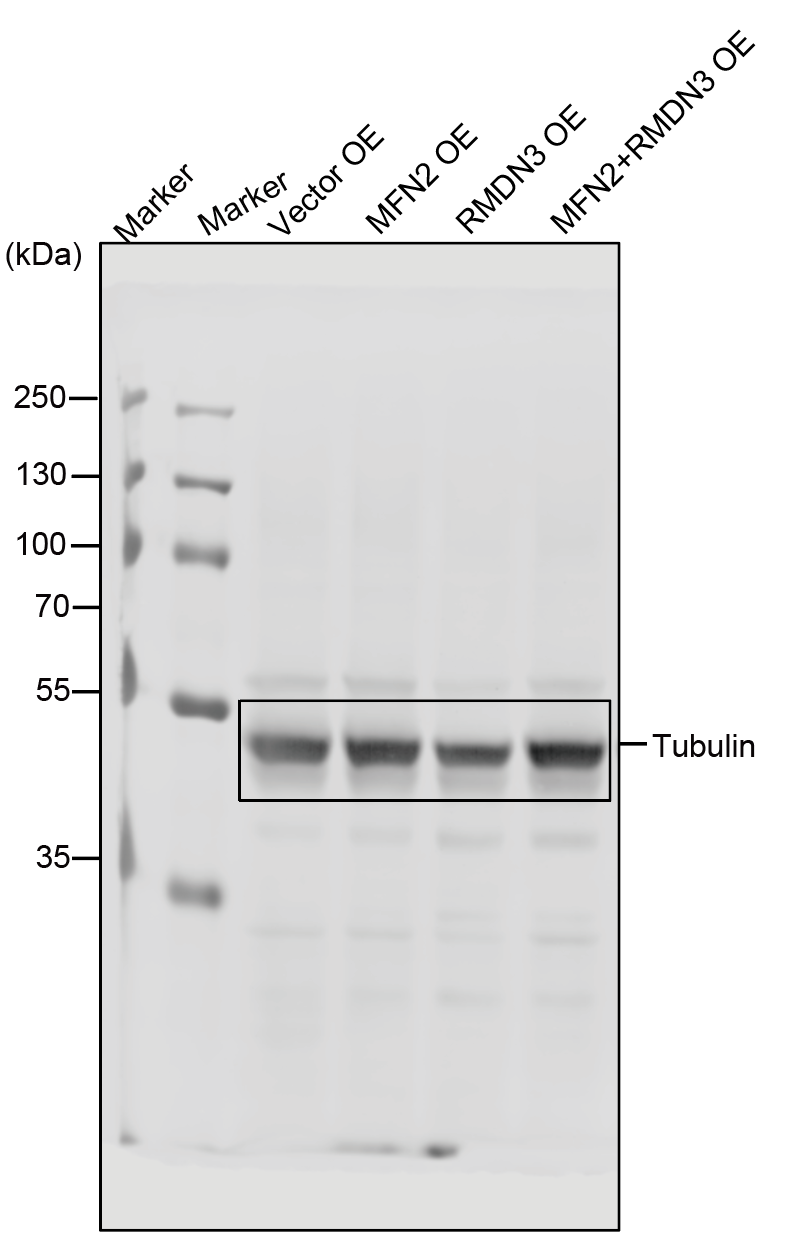

Supplement: Supplementary file 5 — Source data Fig. 2 [file 44318_2026_816_MOESM5_ESM.zip › A/tubulin-2.tif]

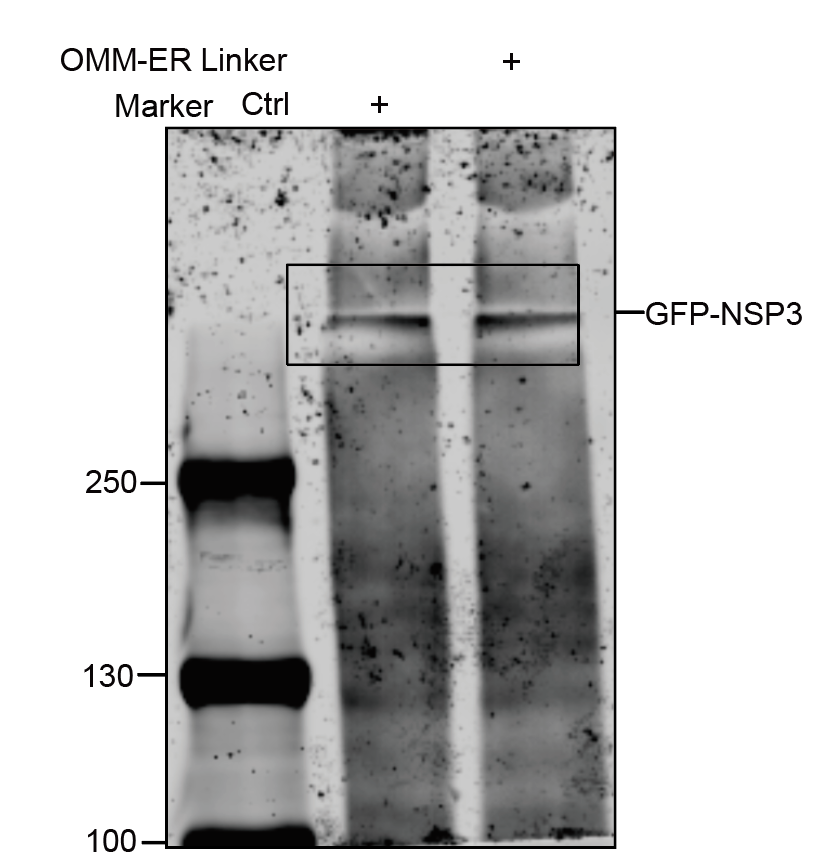

Supplement: Supplementary file 5 — Source data Fig. 2 [file 44318_2026_816_MOESM5_ESM.zip › B/GFP-NSP3.tif]

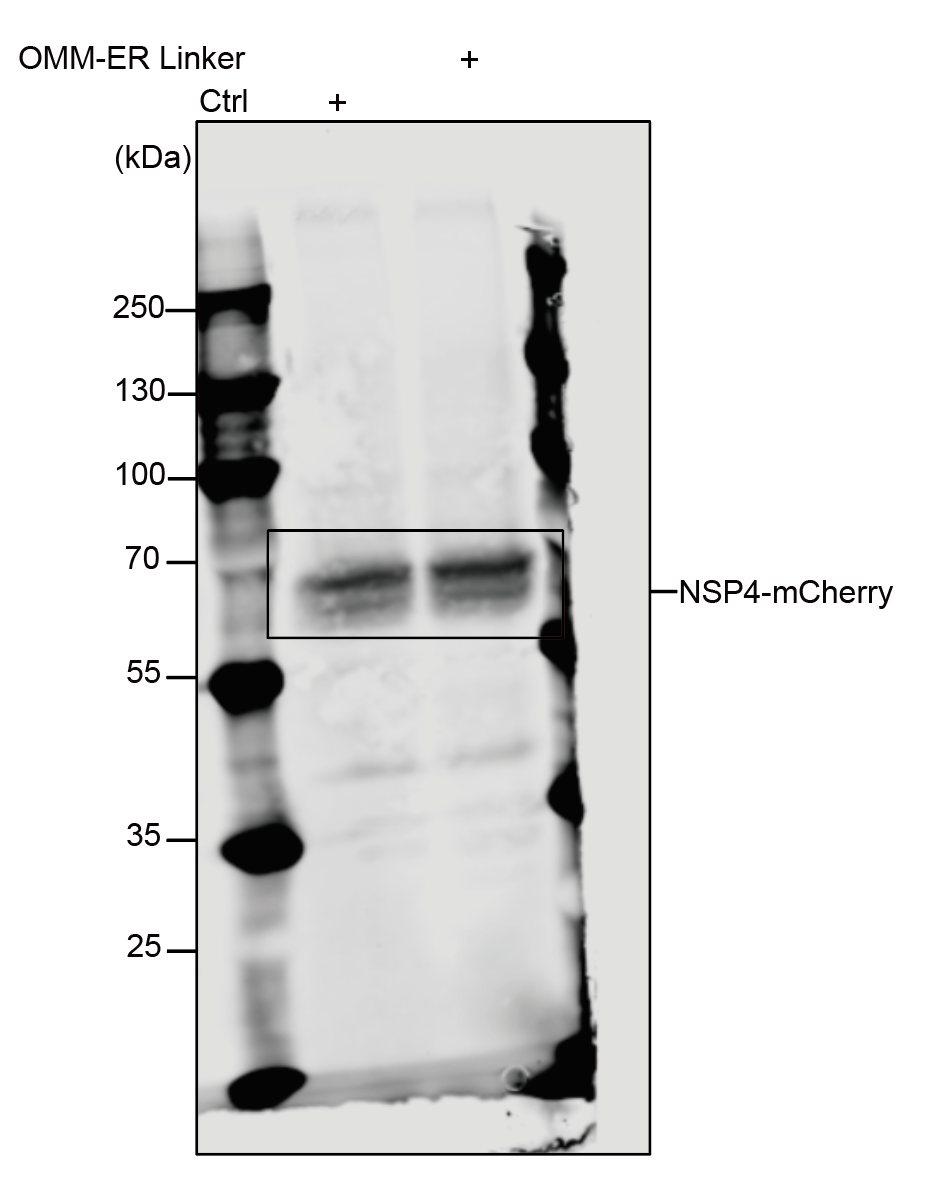

Supplement: Supplementary file 5 — Source data Fig. 2 [file 44318_2026_816_MOESM5_ESM.zip › B/NSP4-mCherry.tif]

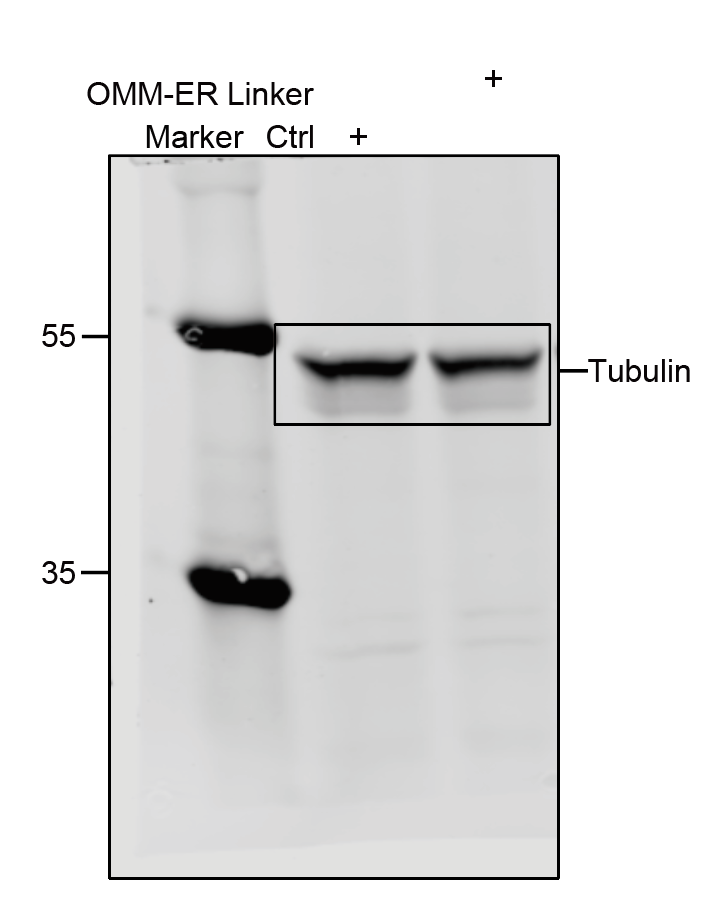

Supplement: Supplementary file 5 — Source data Fig. 2 [file 44318_2026_816_MOESM5_ESM.zip › B/Tubulin-1.tif]

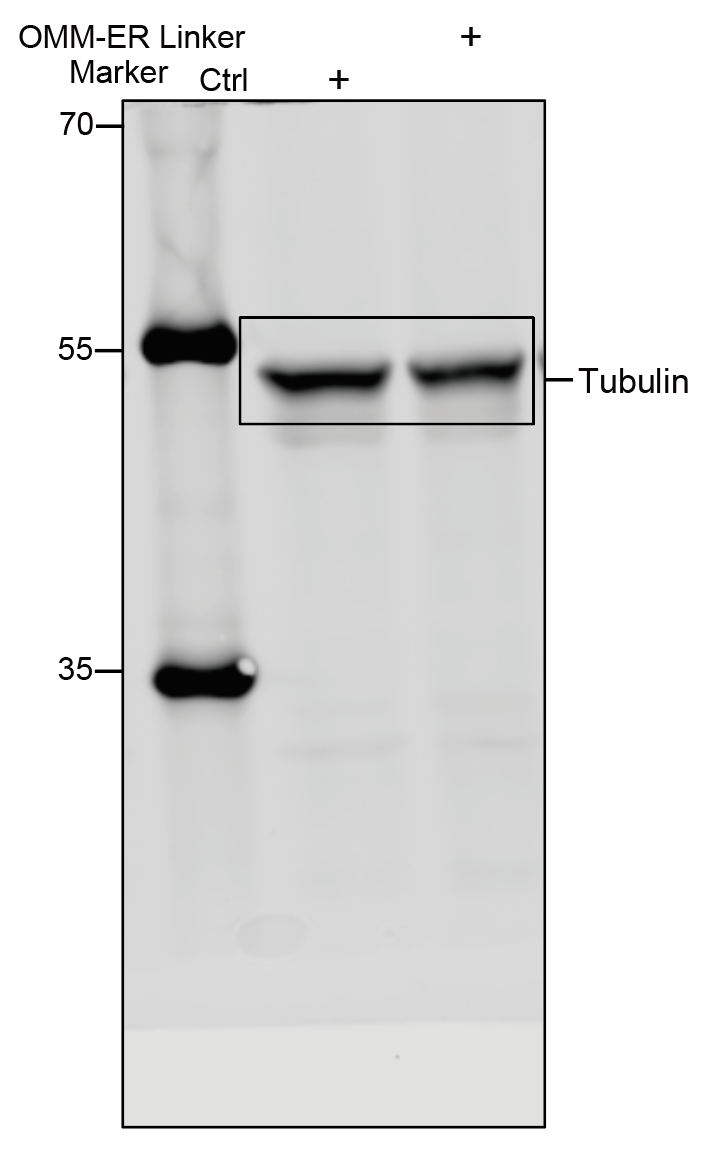

Supplement: Supplementary file 5 — Source data Fig. 2 [file 44318_2026_816_MOESM5_ESM.zip › B/Tubulin-2.tif]

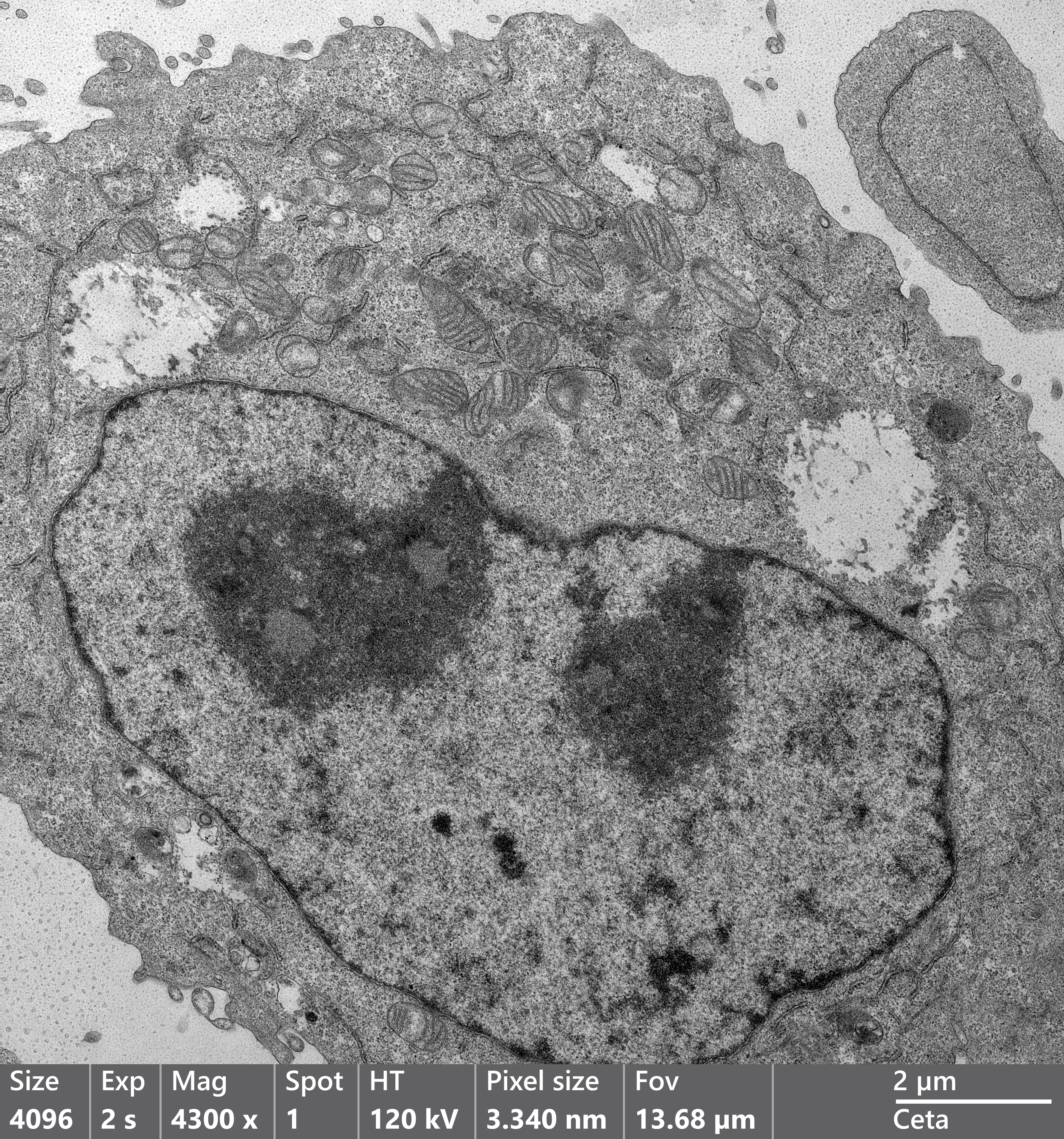

Supplement: Supplementary file 5 — Source data Fig. 2 [file 44318_2026_816_MOESM5_ESM.zip › C/Ctrl.tif]

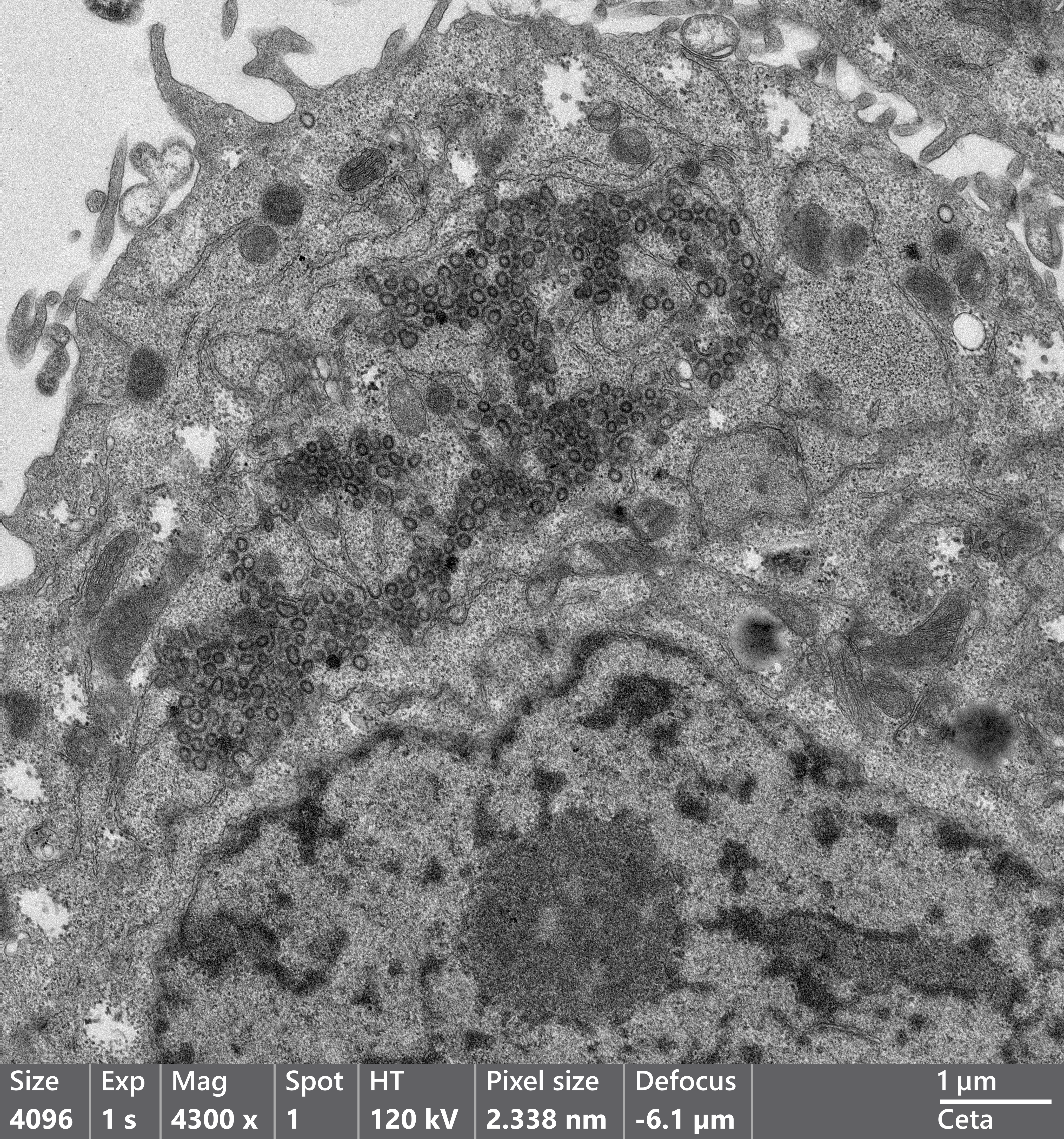

Supplement: Supplementary file 5 — Source data Fig. 2 [file 44318_2026_816_MOESM5_ESM.zip › C/MFN2 OE.tif]

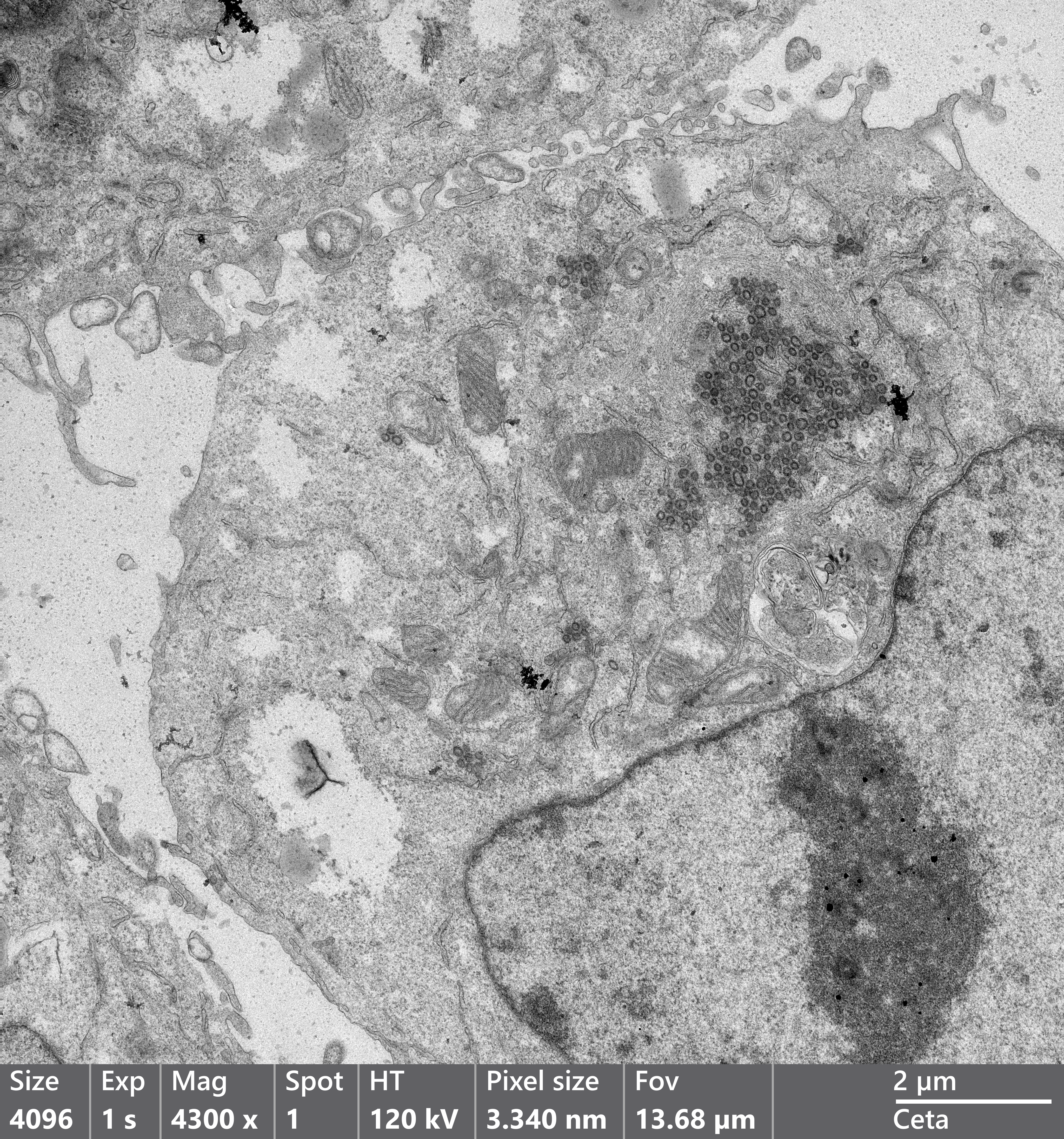

Supplement: Supplementary file 5 — Source data Fig. 2 [file 44318_2026_816_MOESM5_ESM.zip › C/MFN2+RMDN3 OE.tif]

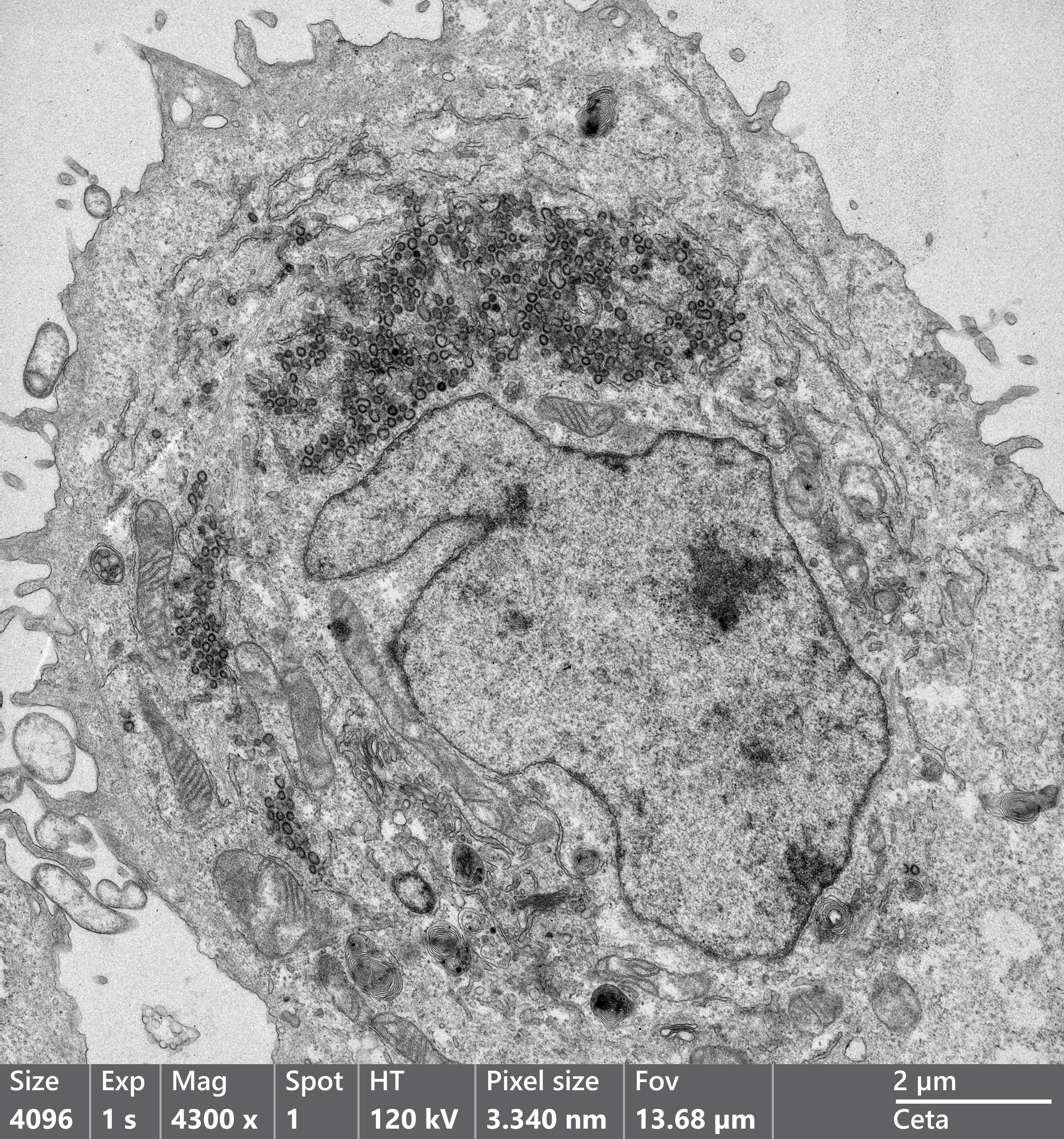

Supplement: Supplementary file 5 — Source data Fig. 2 [file 44318_2026_816_MOESM5_ESM.zip › C/RMDN3 OE.tif]

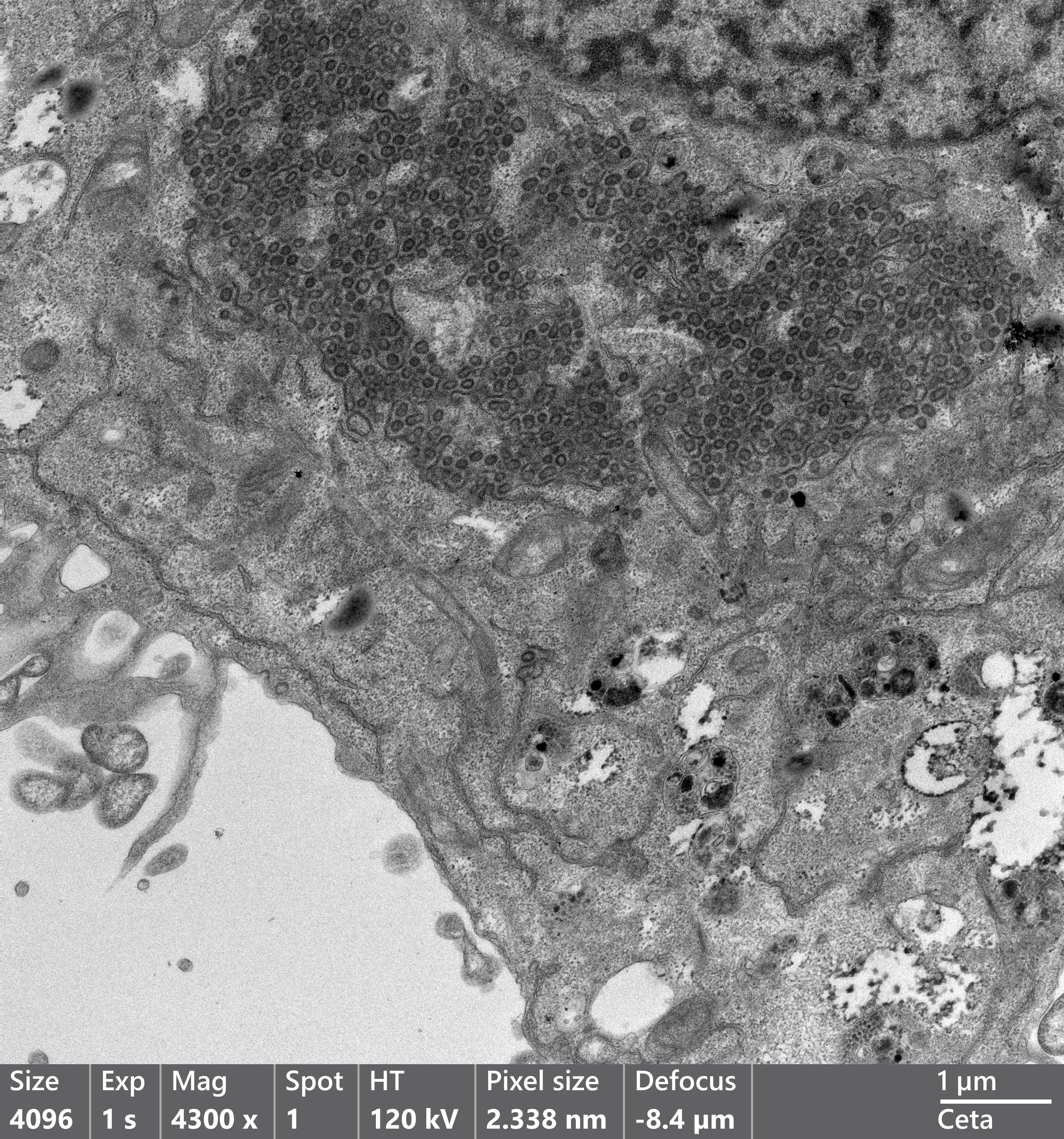

Supplement: Supplementary file 5 — Source data Fig. 2 [file 44318_2026_816_MOESM5_ESM.zip › C/Vector OE.tif]

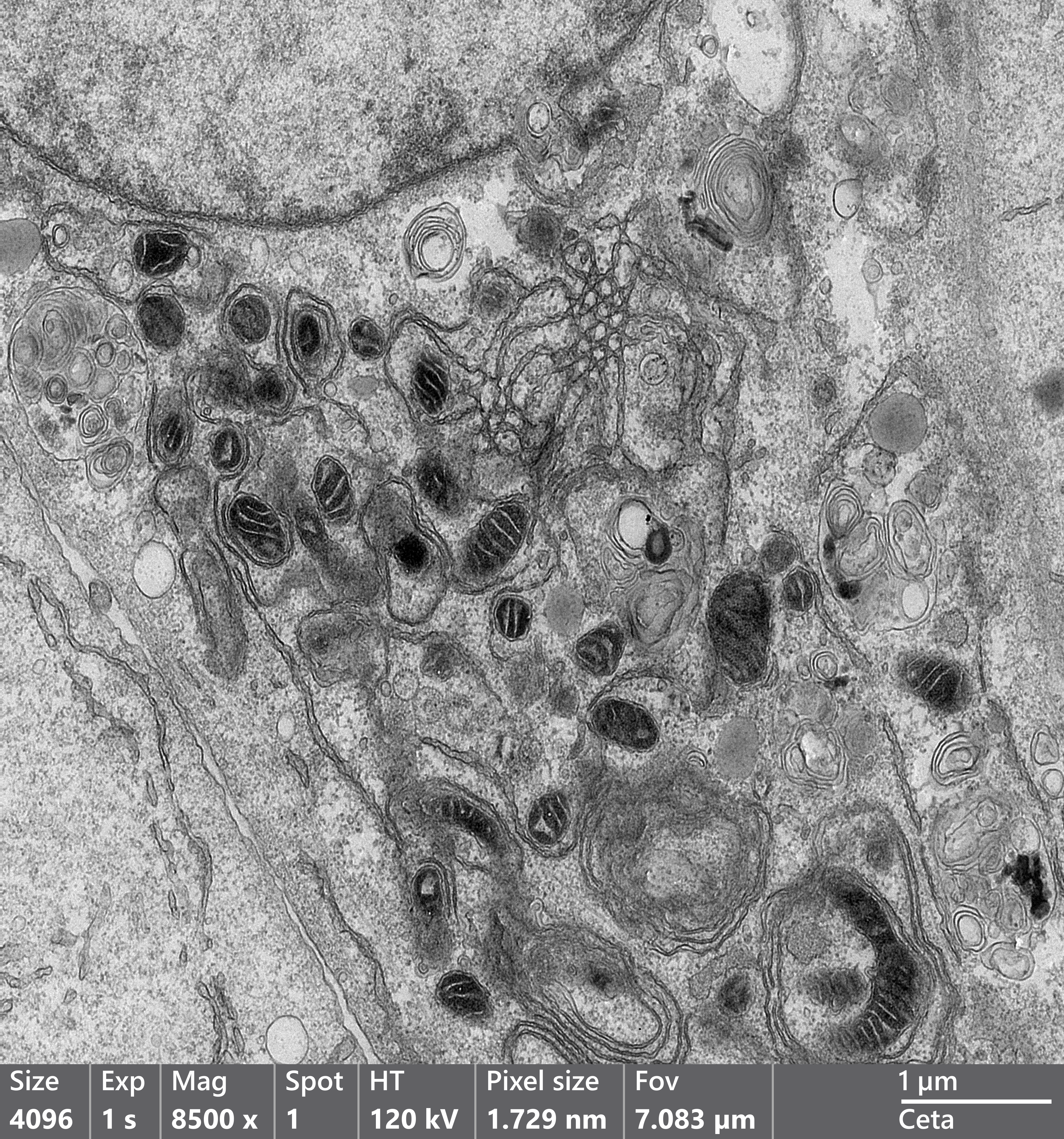

Supplement: Supplementary file 5 — Source data Fig. 2 [file 44318_2026_816_MOESM5_ESM.zip › G/Figure 2G +OMM-ER Linker.tif]

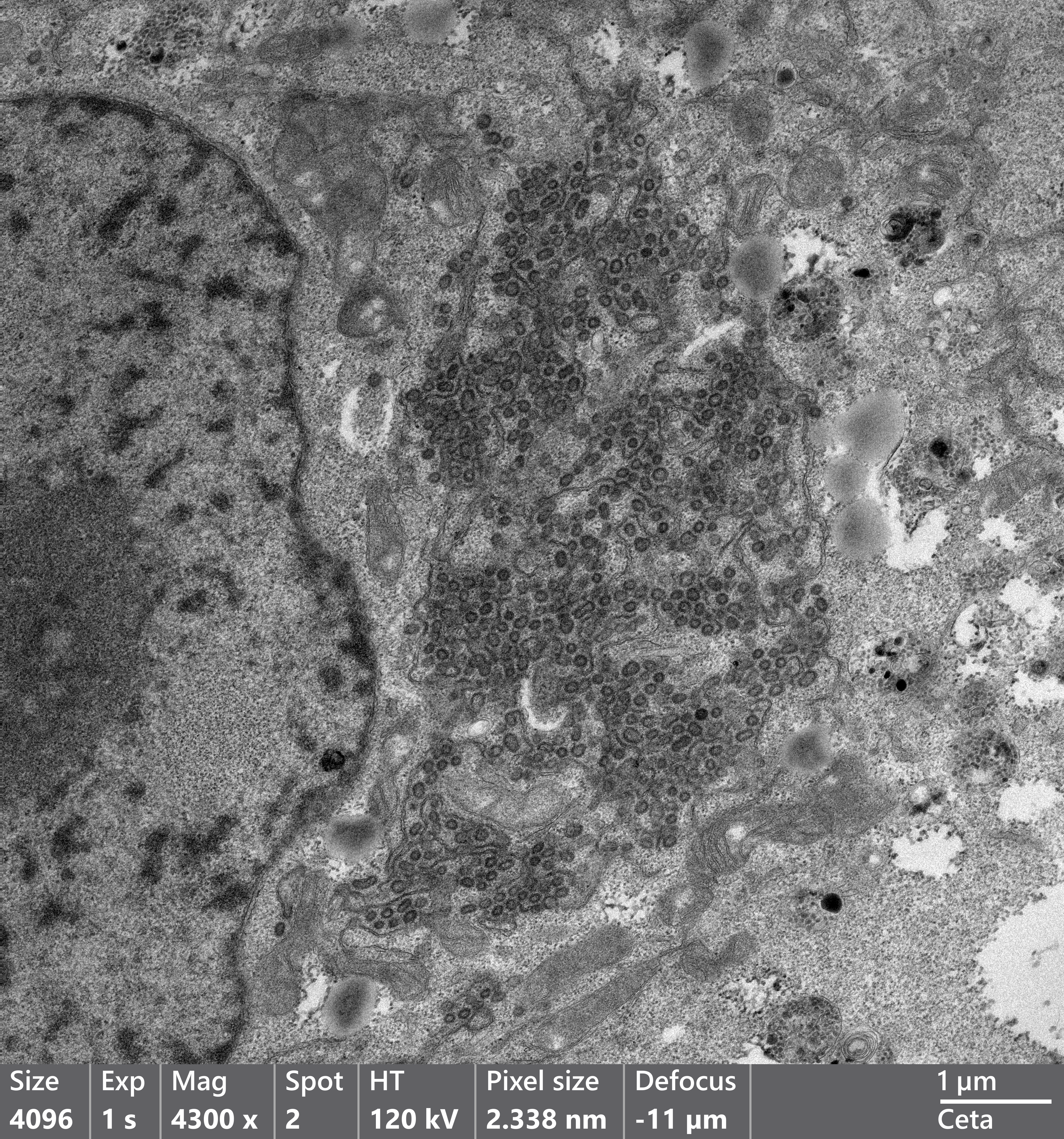

Supplement: Supplementary file 5 — Source data Fig. 2 [file 44318_2026_816_MOESM5_ESM.zip › G/Figure 2G Ctrl.tif]

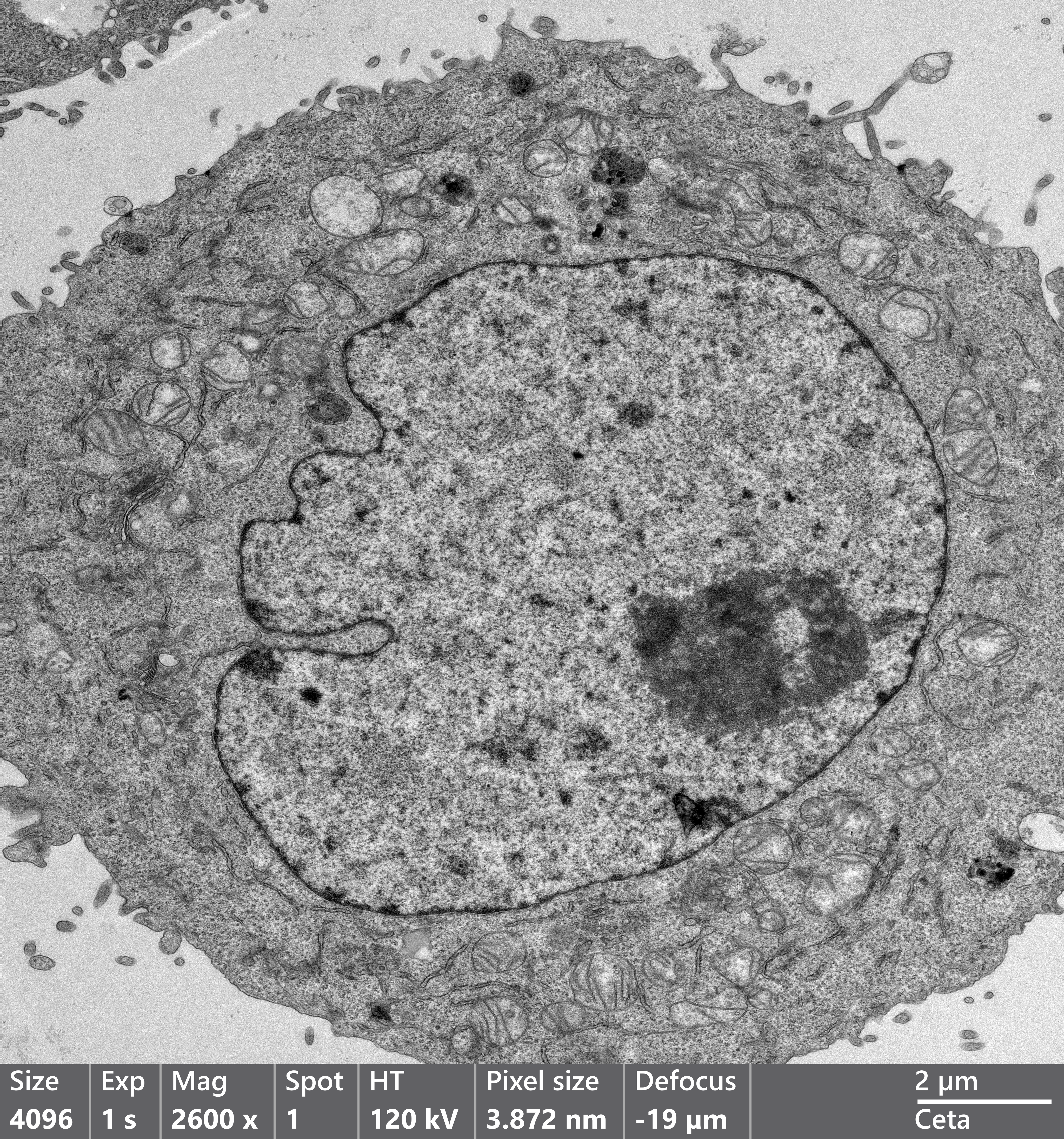

Supplement: Supplementary file 6 — Source data Fig. 3 [file 44318_2026_816_MOESM6_ESM.zip › A/Figure 3A Ctrl.tif]

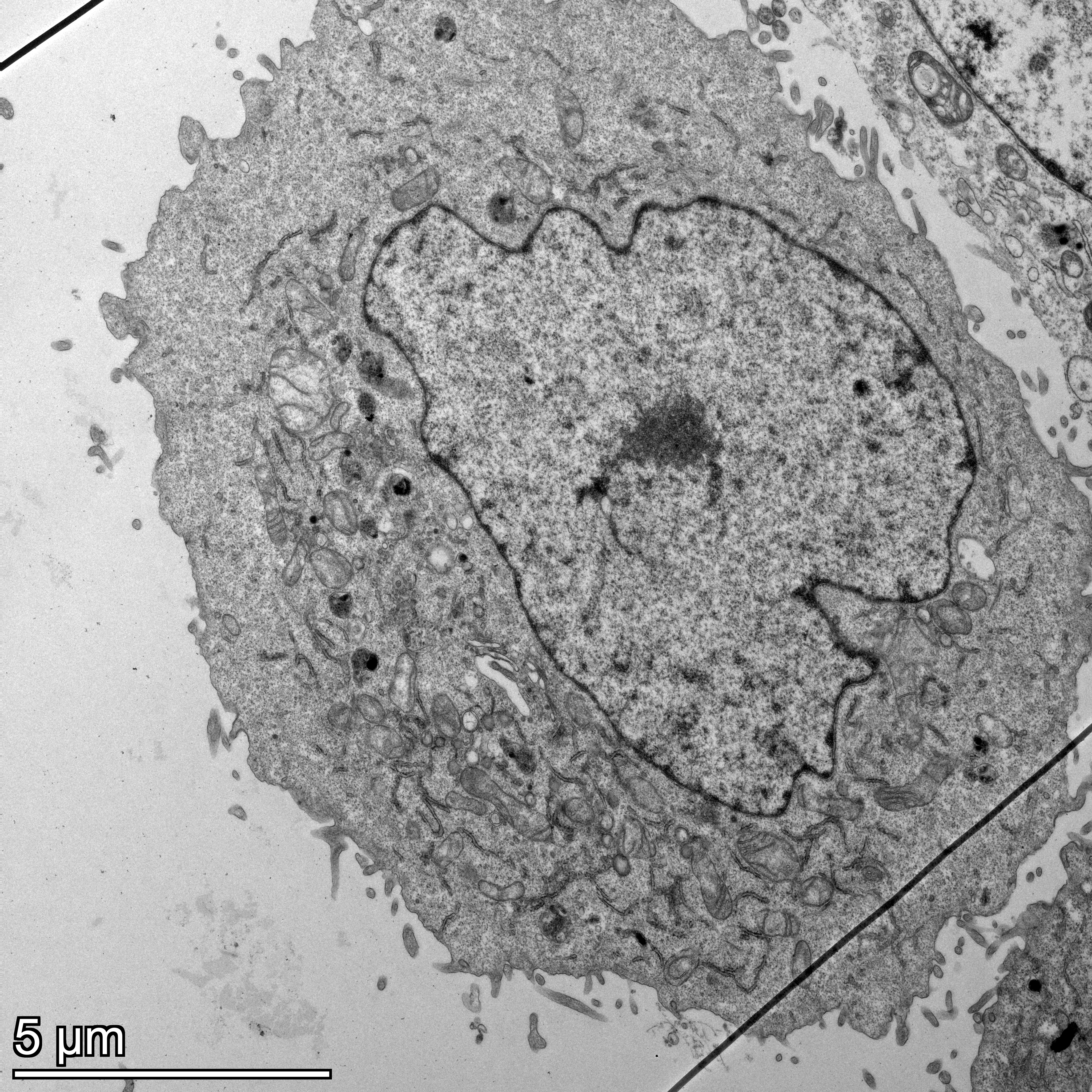

Supplement: Supplementary file 6 — Source data Fig. 3 [file 44318_2026_816_MOESM6_ESM.zip › A/Figure 3A MFN2 OE.tif]

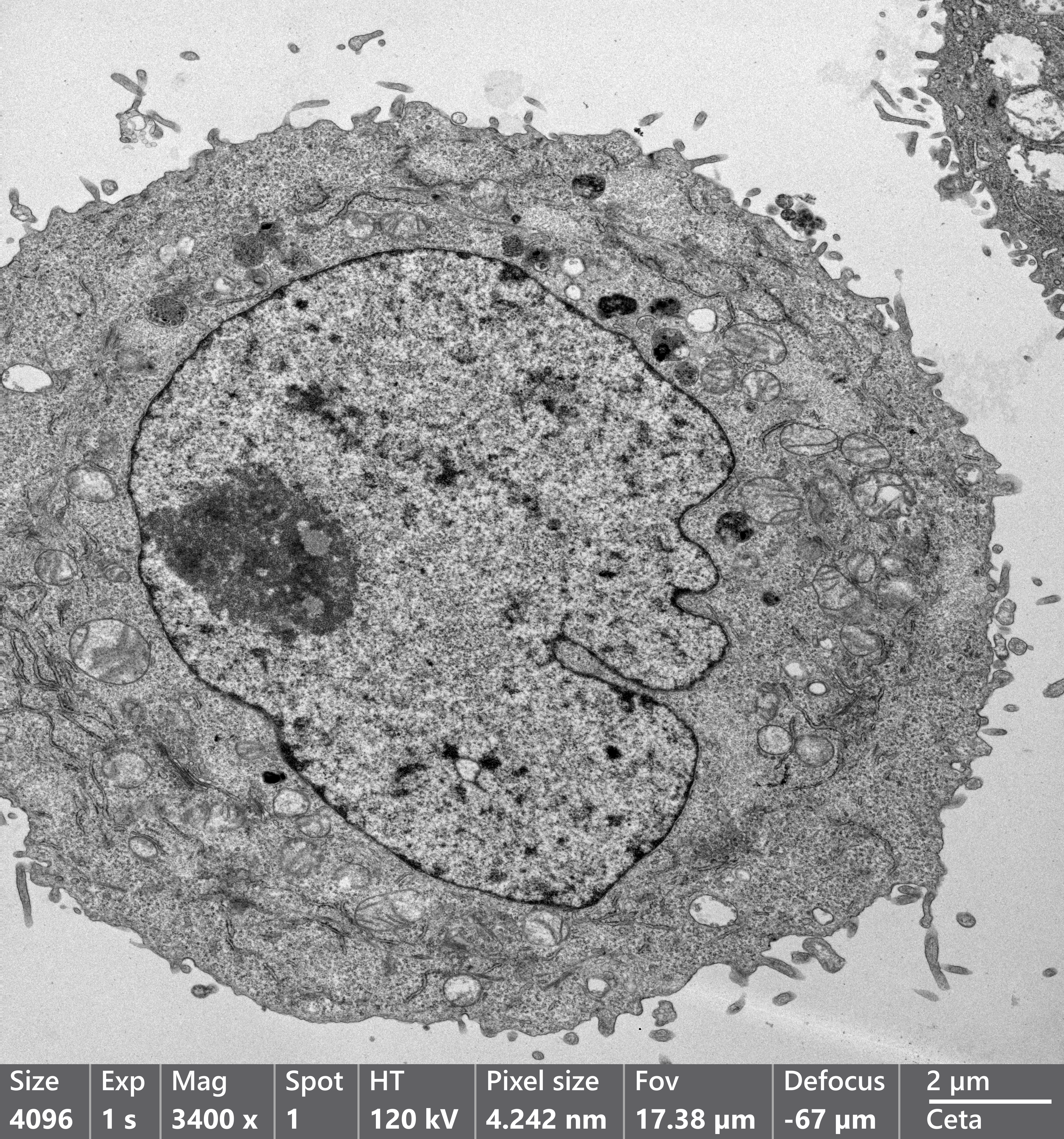

Supplement: Supplementary file 6 — Source data Fig. 3 [file 44318_2026_816_MOESM6_ESM.zip › A/Figure 3A MFN2+RMDN3 OE.tif]

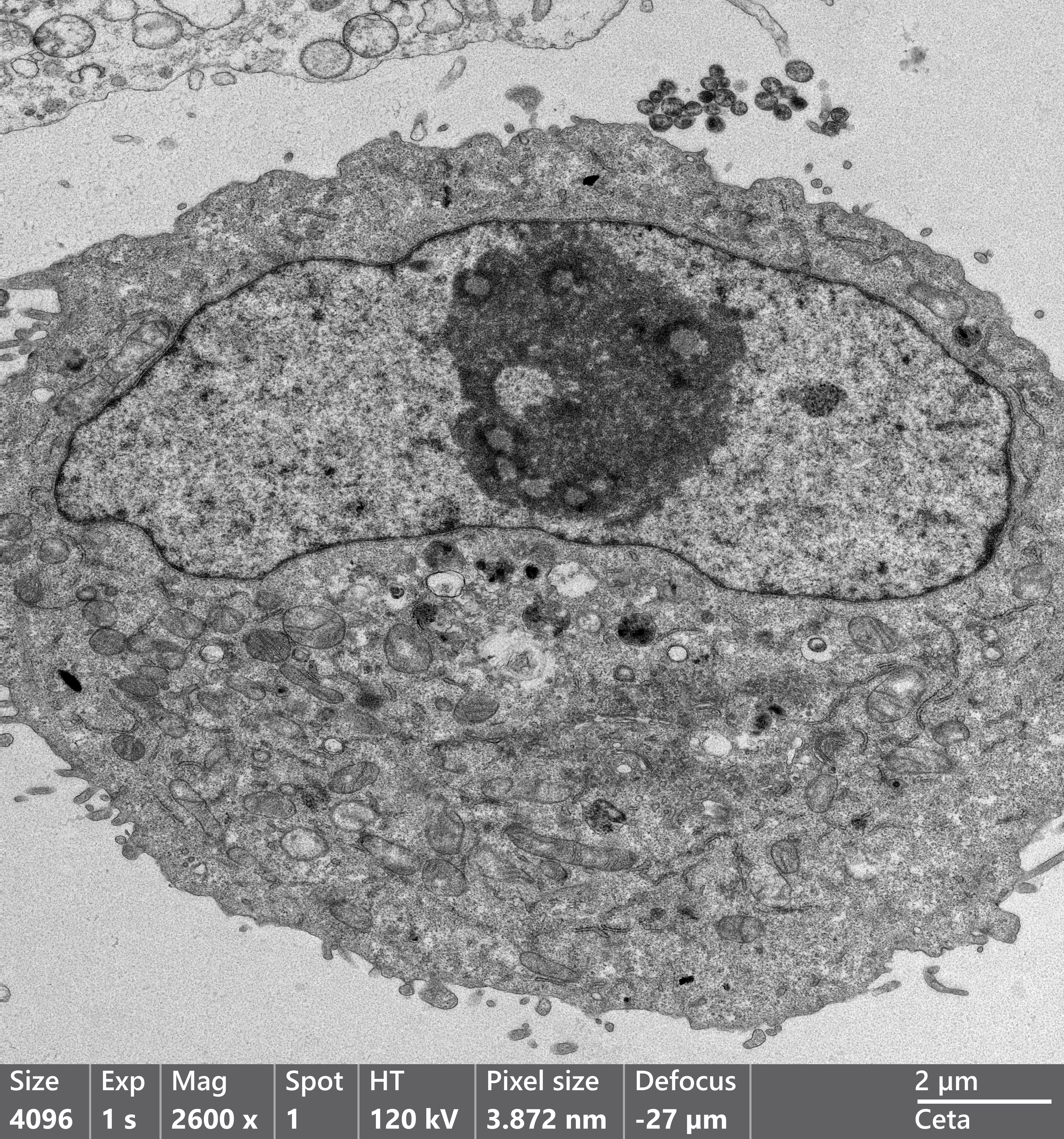

Supplement: Supplementary file 6 — Source data Fig. 3 [file 44318_2026_816_MOESM6_ESM.zip › A/Figure 3A RMDN3 OE.tif]

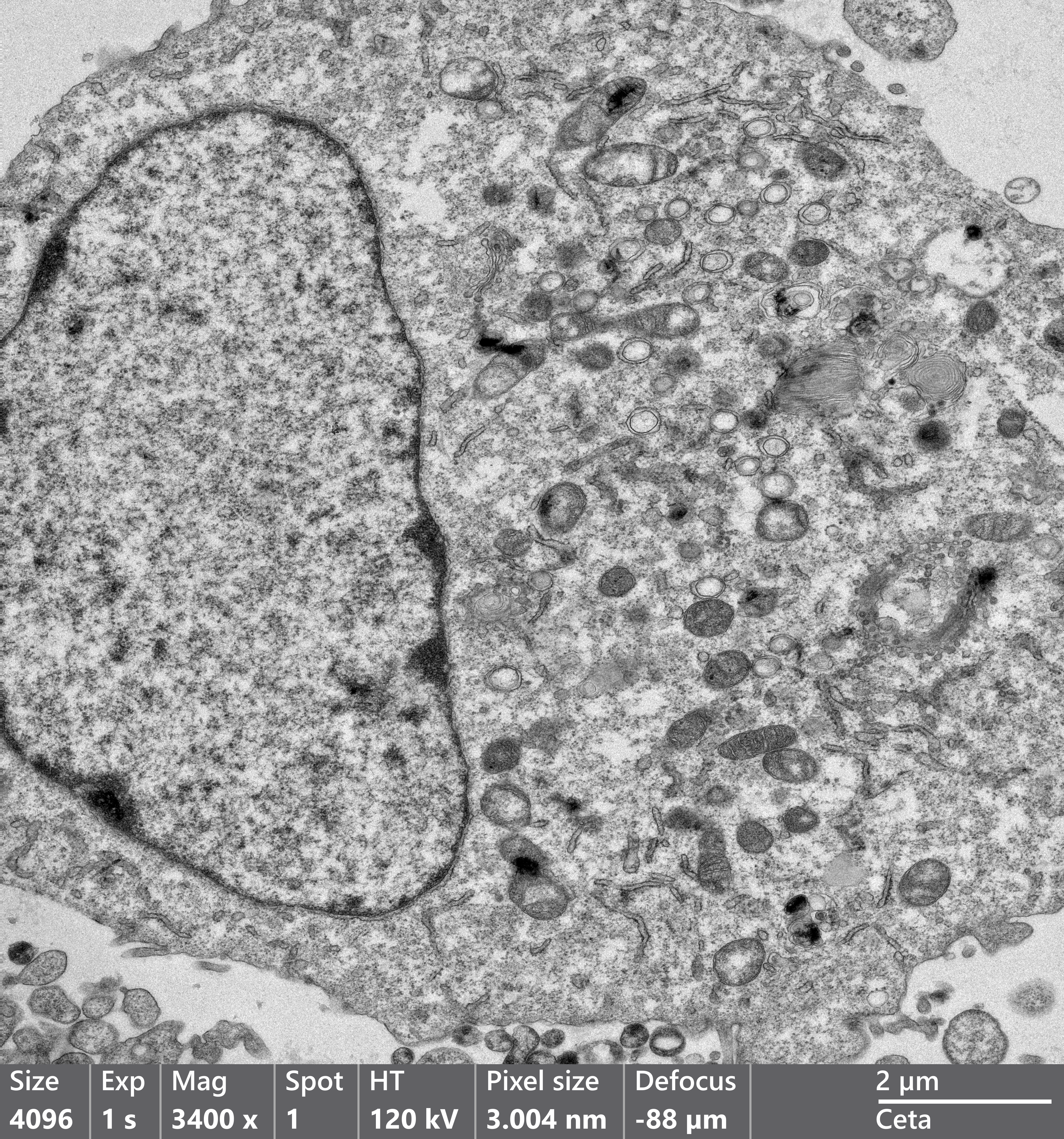

Supplement: Supplementary file 6 — Source data Fig. 3 [file 44318_2026_816_MOESM6_ESM.zip › A/Figure 3A Vector OE.tif]

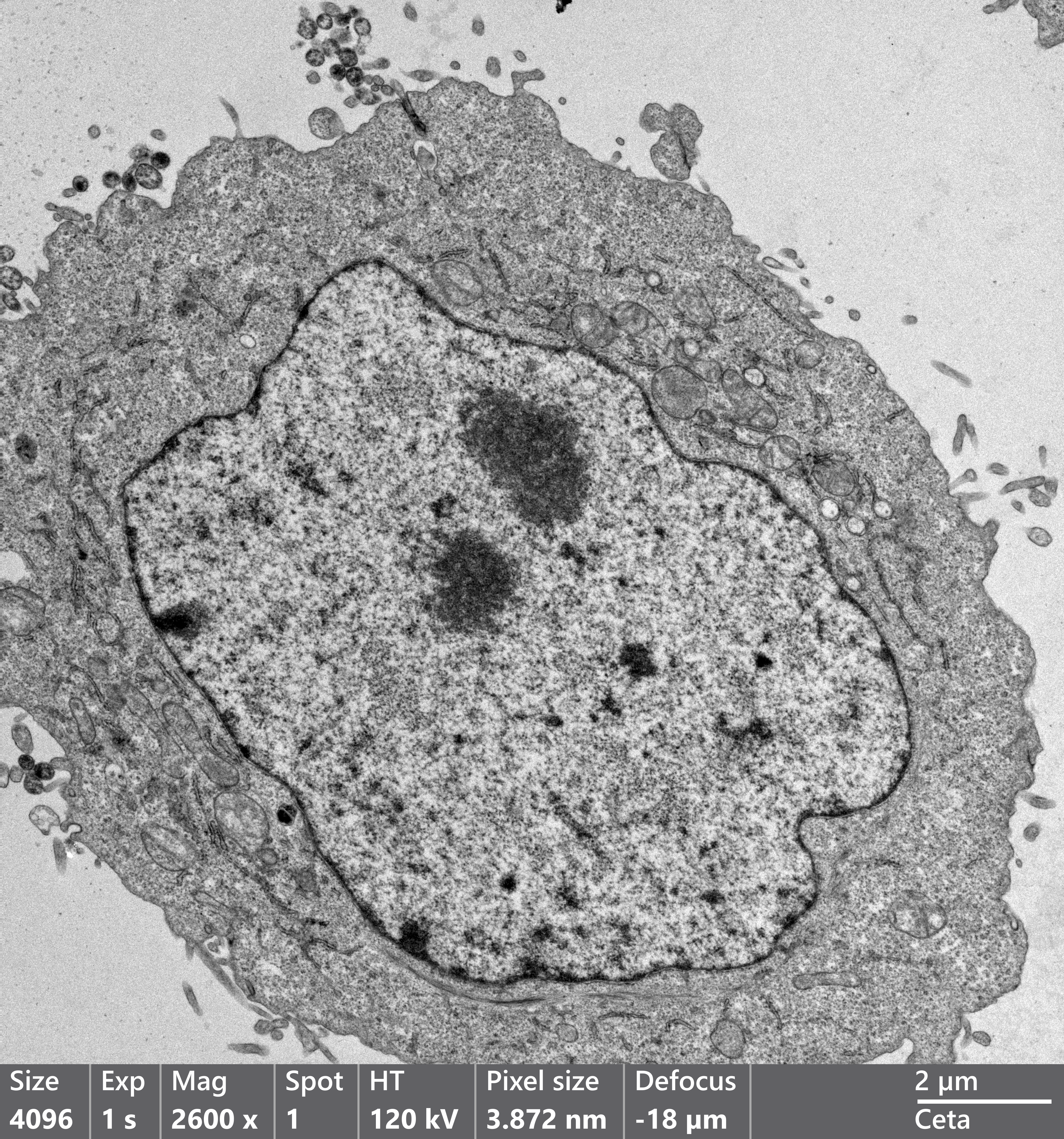

Supplement: Supplementary file 6 — Source data Fig. 3 [file 44318_2026_816_MOESM6_ESM.zip › E/Figure 3E +OMM-ER Linker.tif]

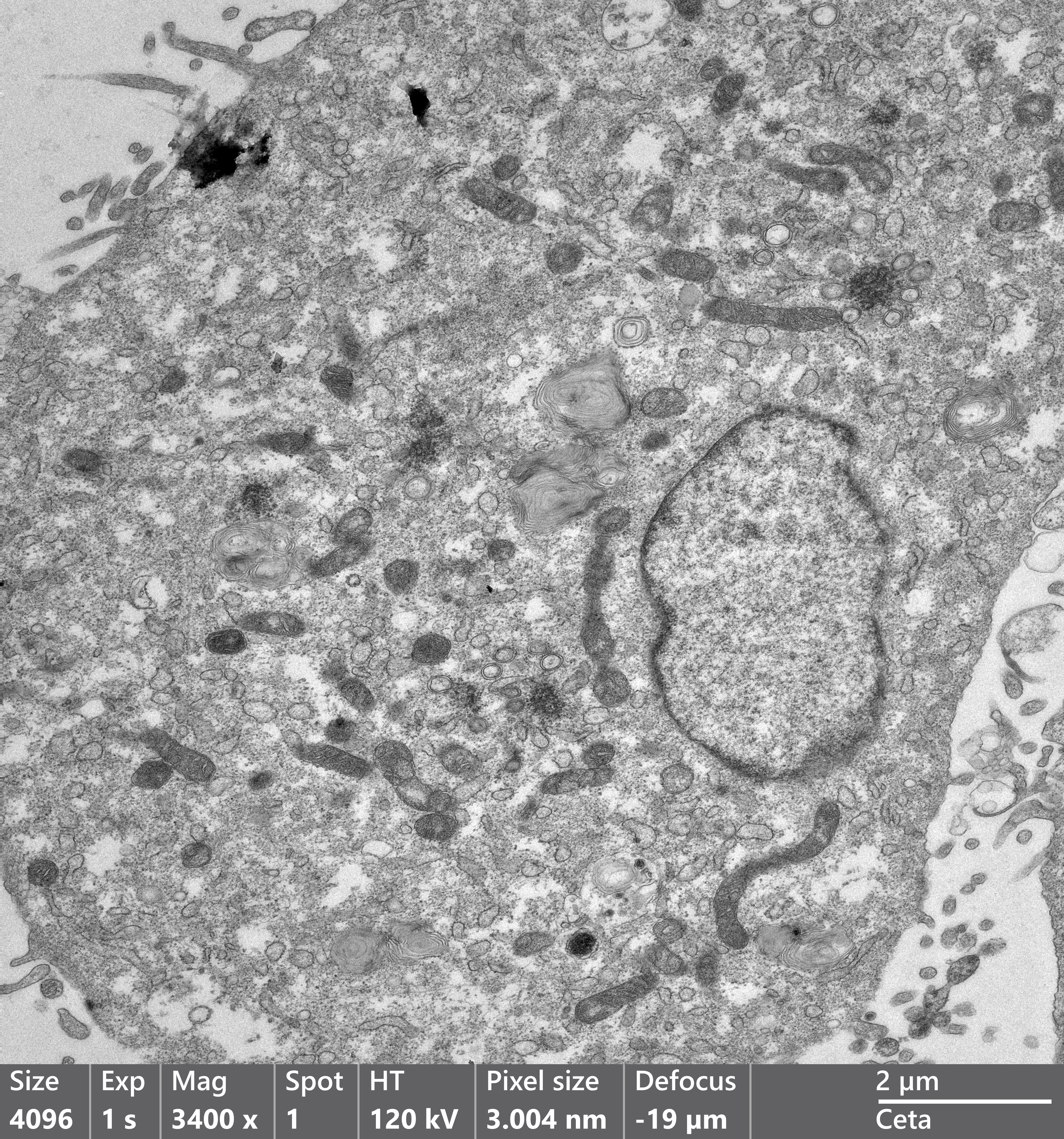

Supplement: Supplementary file 6 — Source data Fig. 3 [file 44318_2026_816_MOESM6_ESM.zip › E/Figure 3E Ctrl.tif]

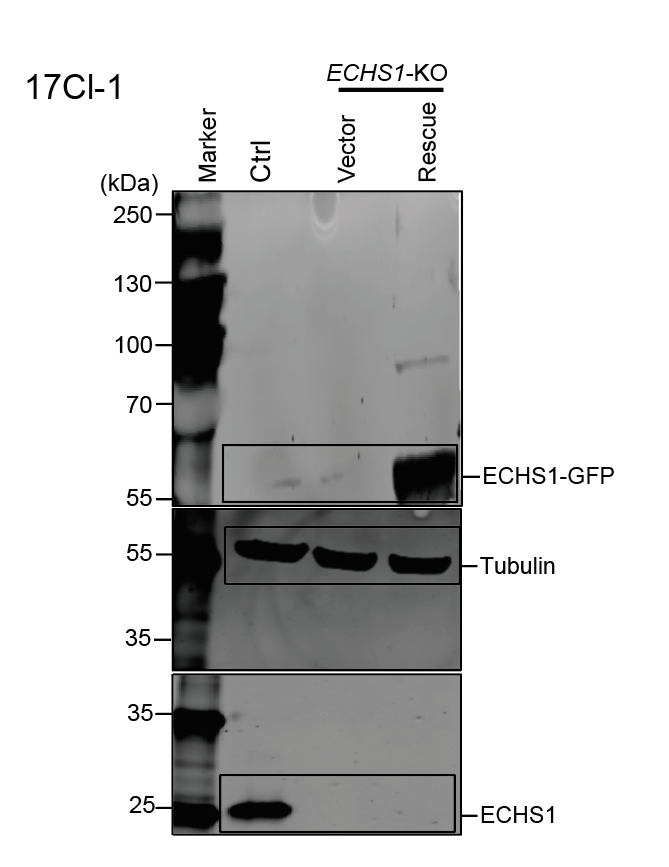

Supplement: Supplementary file 8 — Source data Fig. 5 [file 44318_2026_816_MOESM8_ESM.zip › A/ECHS1-GFP-ECHS1-Tubulin.tif]

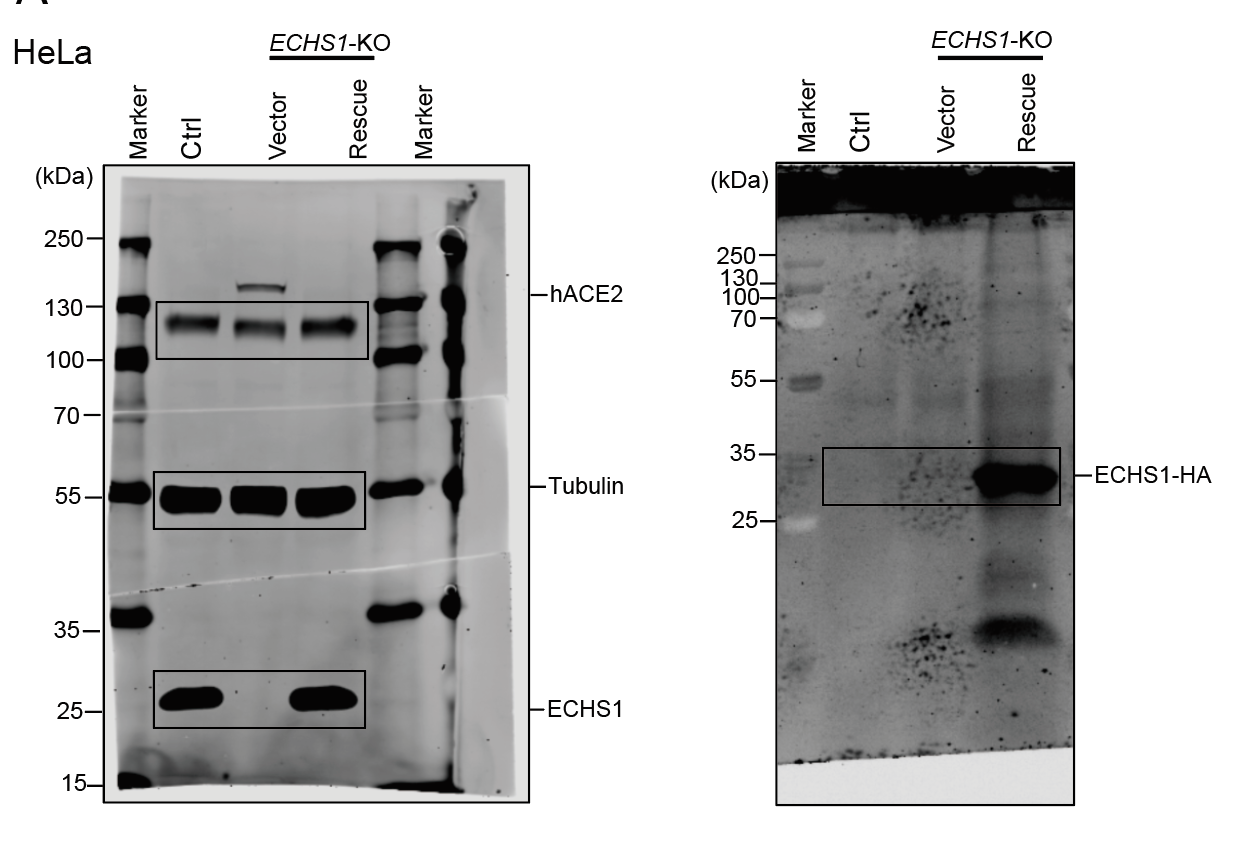

Supplement: Supplementary file 8 — Source data Fig. 5 [file 44318_2026_816_MOESM8_ESM.zip › A/hACE2-ECHS1-ECHS1-HA-Tubulin.tif]

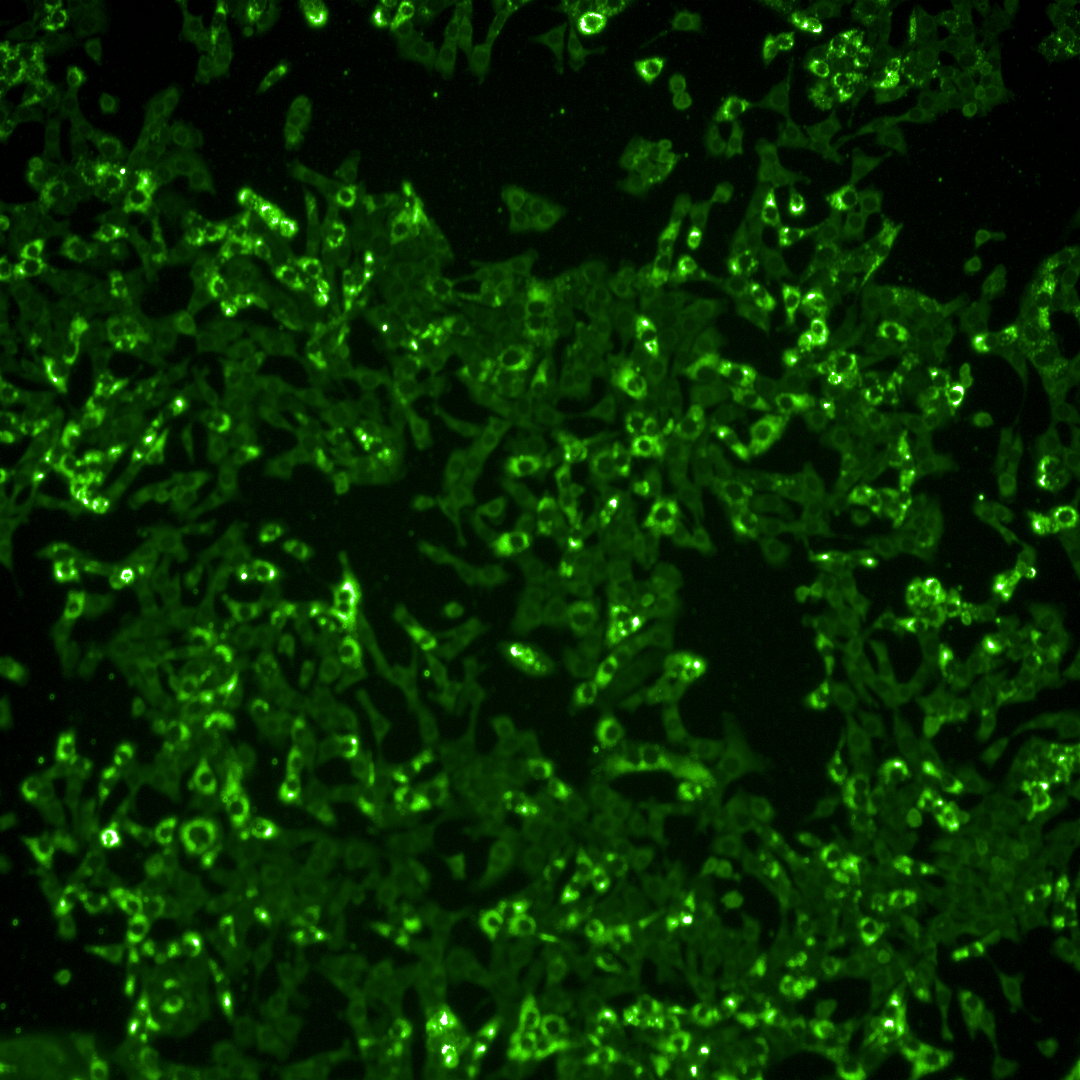

Supplement: Supplementary file 8 — Source data Fig. 5 [file 44318_2026_816_MOESM8_ESM.zip › E/17Cl-1 Ctrl.png]

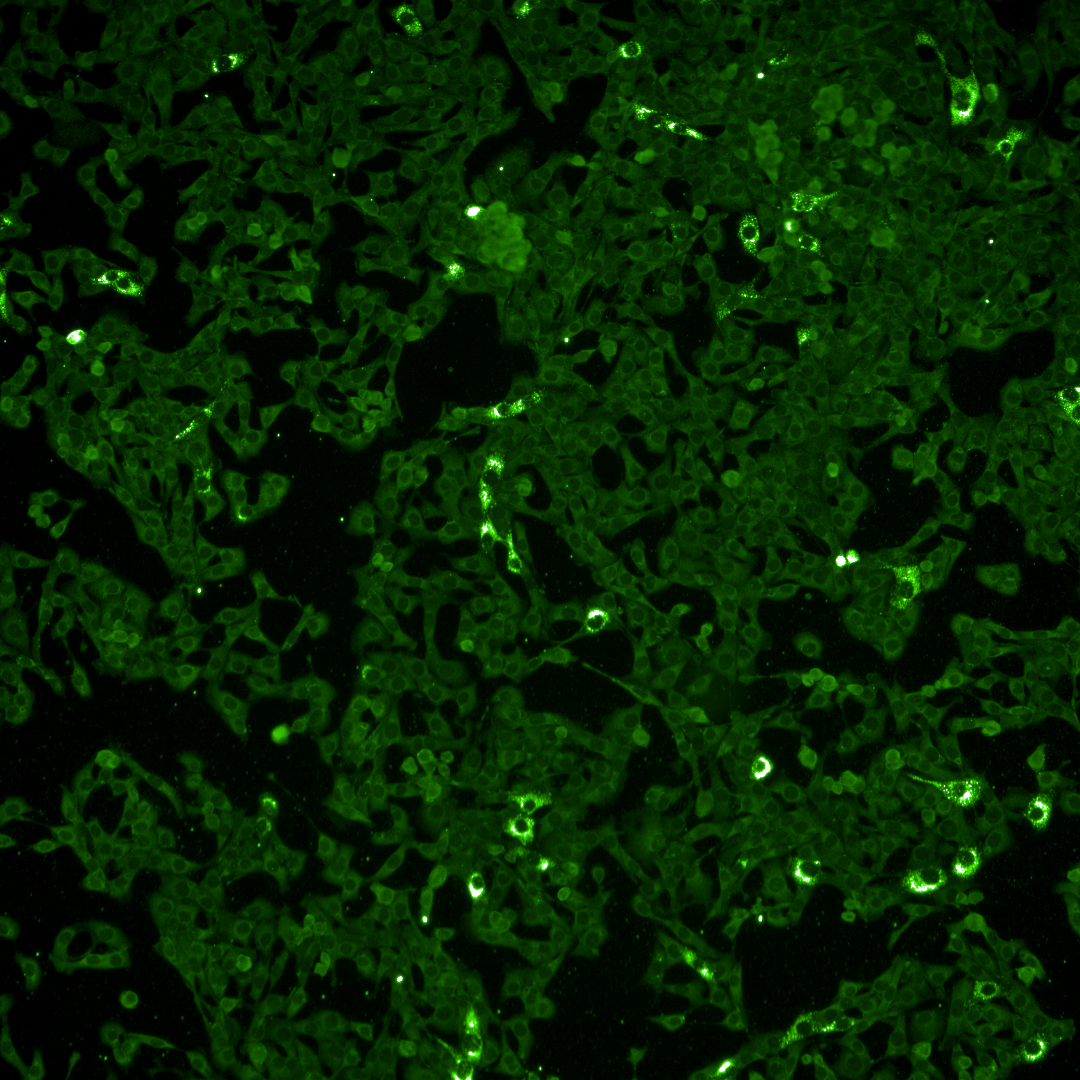

Supplement: Supplementary file 8 — Source data Fig. 5 [file 44318_2026_816_MOESM8_ESM.zip › E/17Cl-1 ECHS1-KO.png]

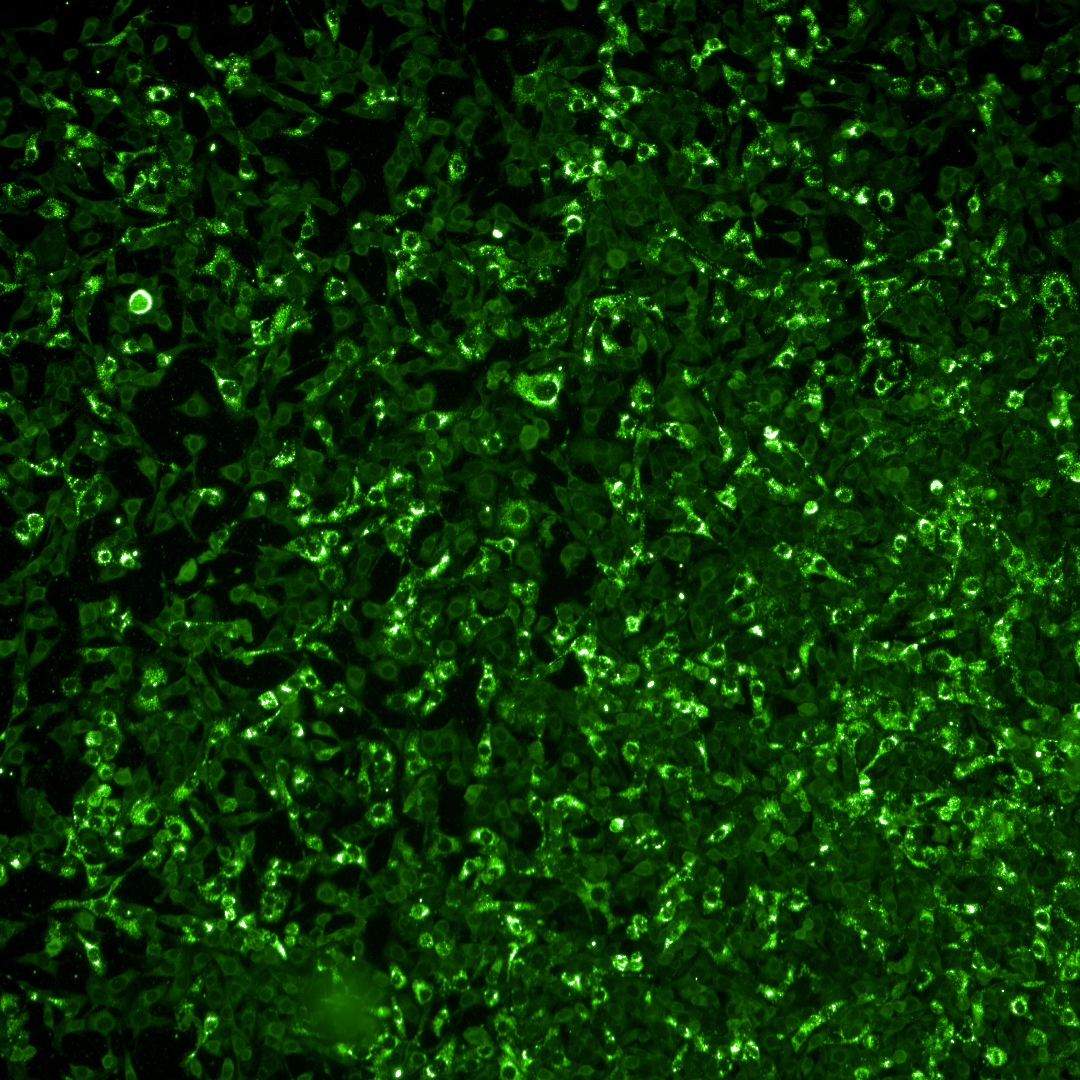

Supplement: Supplementary file 8 — Source data Fig. 5 [file 44318_2026_816_MOESM8_ESM.zip › E/17Cl-1 ECHS1-Rescue.png]

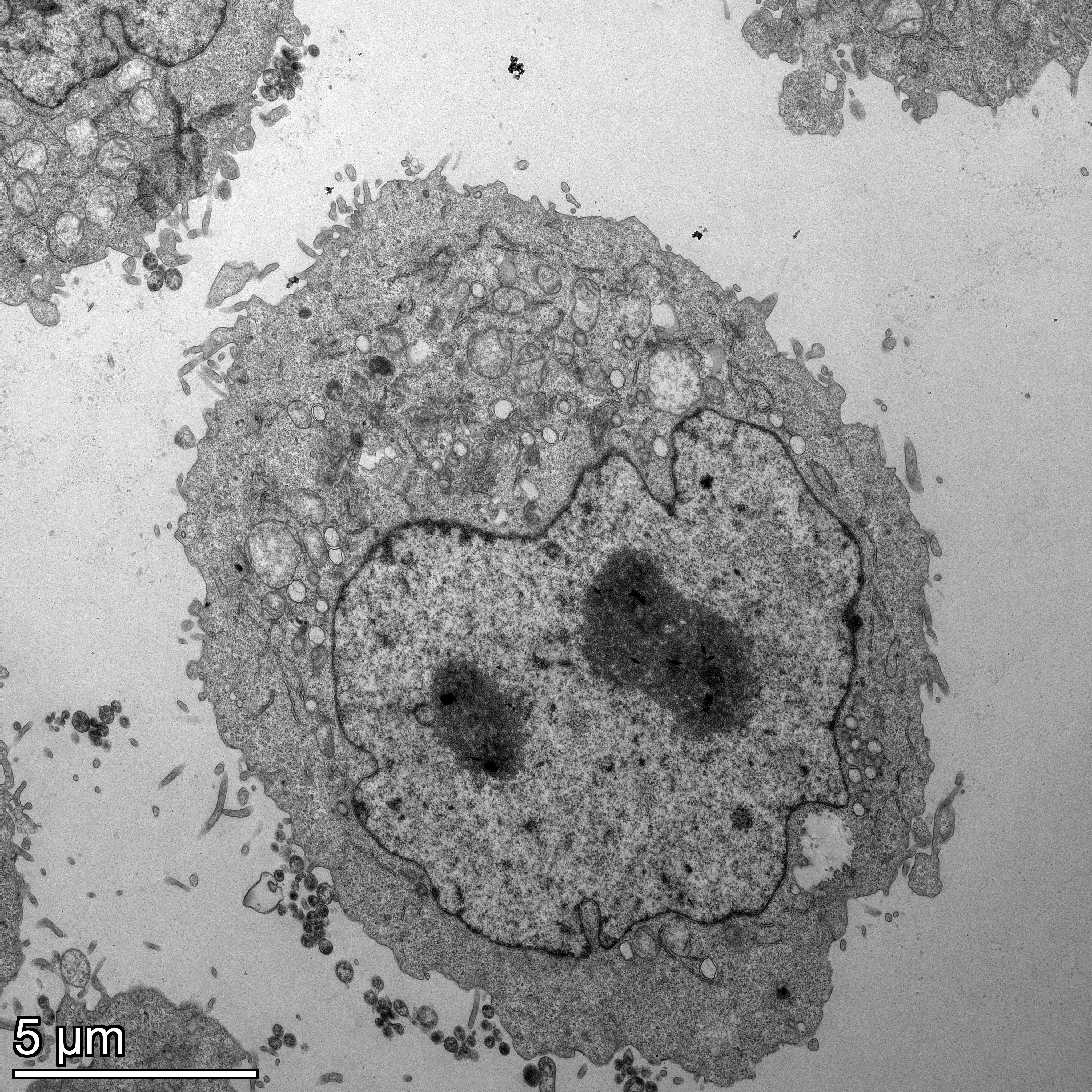

Supplement: Supplementary file 8 — Source data Fig. 5 [file 44318_2026_816_MOESM8_ESM.zip › F/Figure 5F Ctrl.tif]

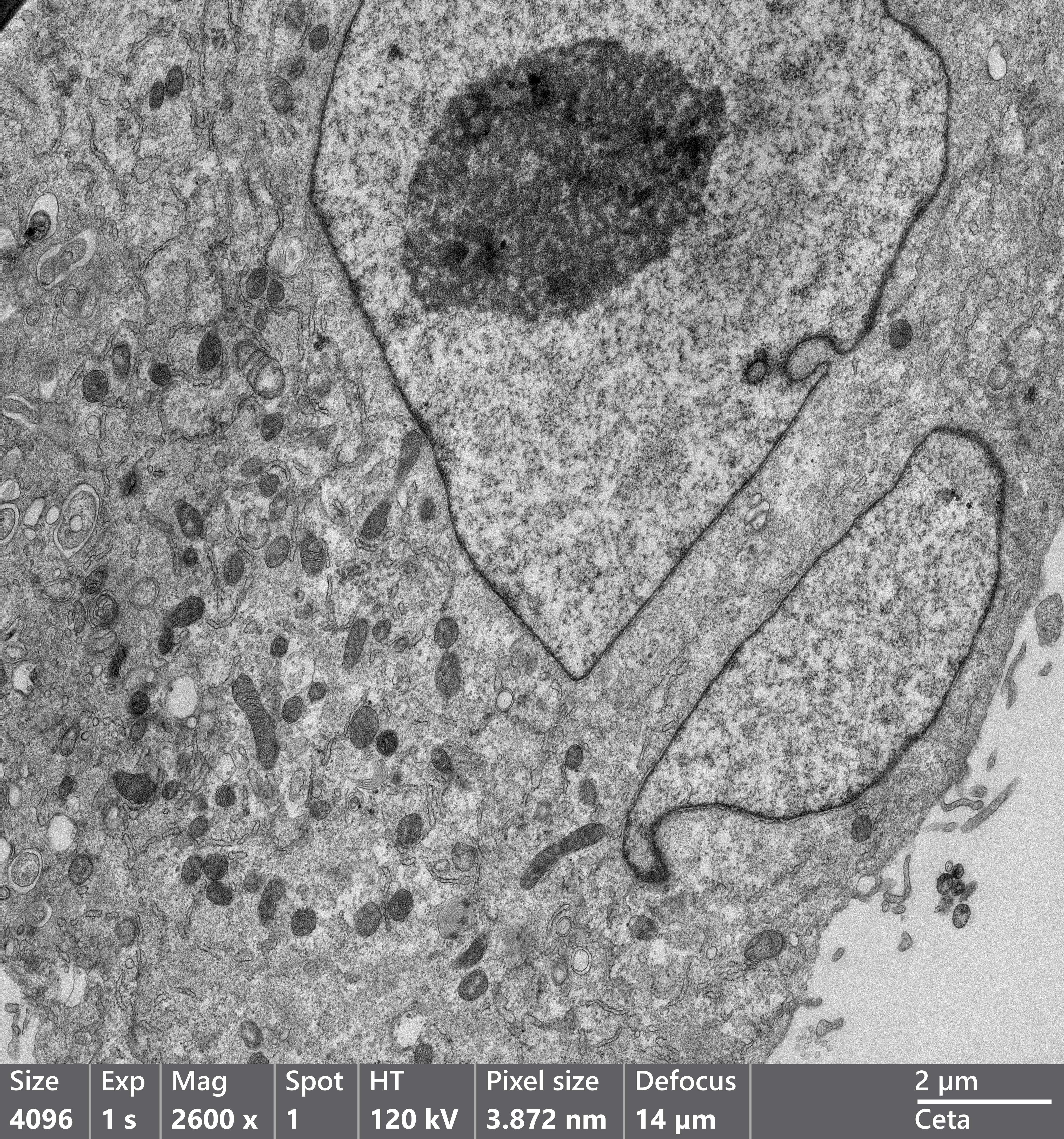

Supplement: Supplementary file 8 — Source data Fig. 5 [file 44318_2026_816_MOESM8_ESM.zip › F/Figure 5F ECHS1-KO.tif]

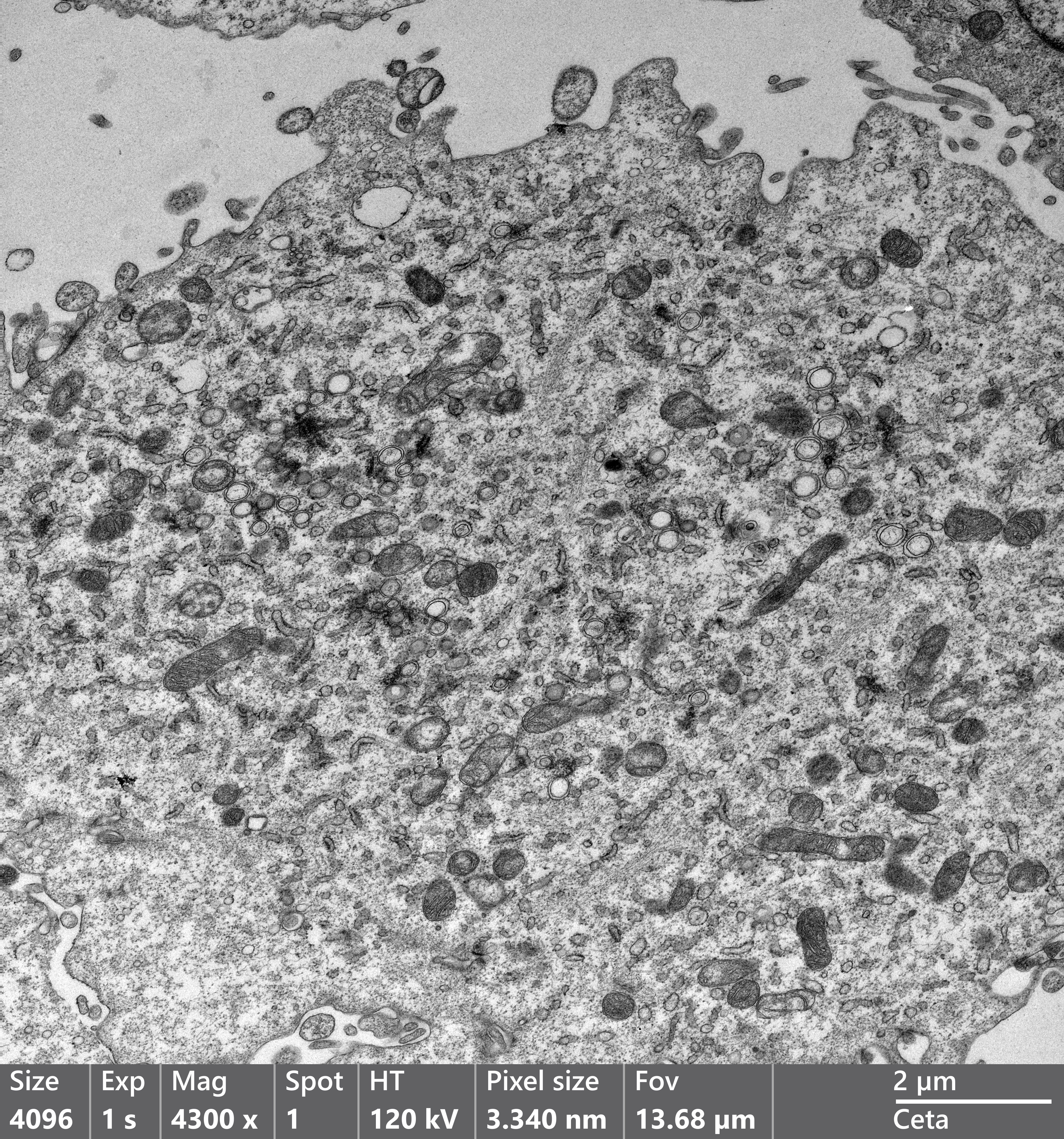

Supplement: Supplementary file 8 — Source data Fig. 5 [file 44318_2026_816_MOESM8_ESM.zip › F/Figure 5F ECHS1-Rescue.tif]

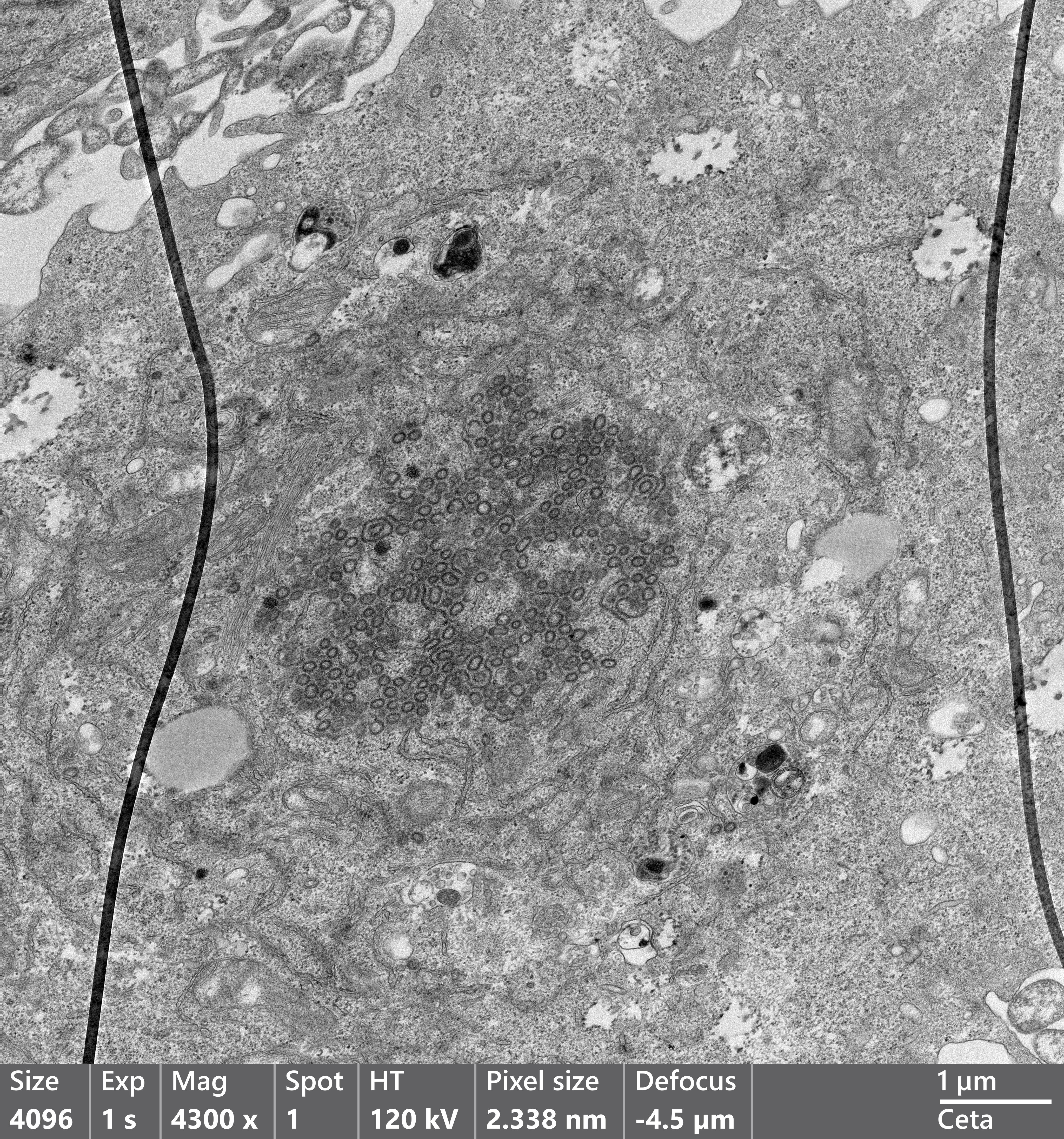

Supplement: Supplementary file 8 — Source data Fig. 5 [file 44318_2026_816_MOESM8_ESM.zip › J/Ctrl.tif]

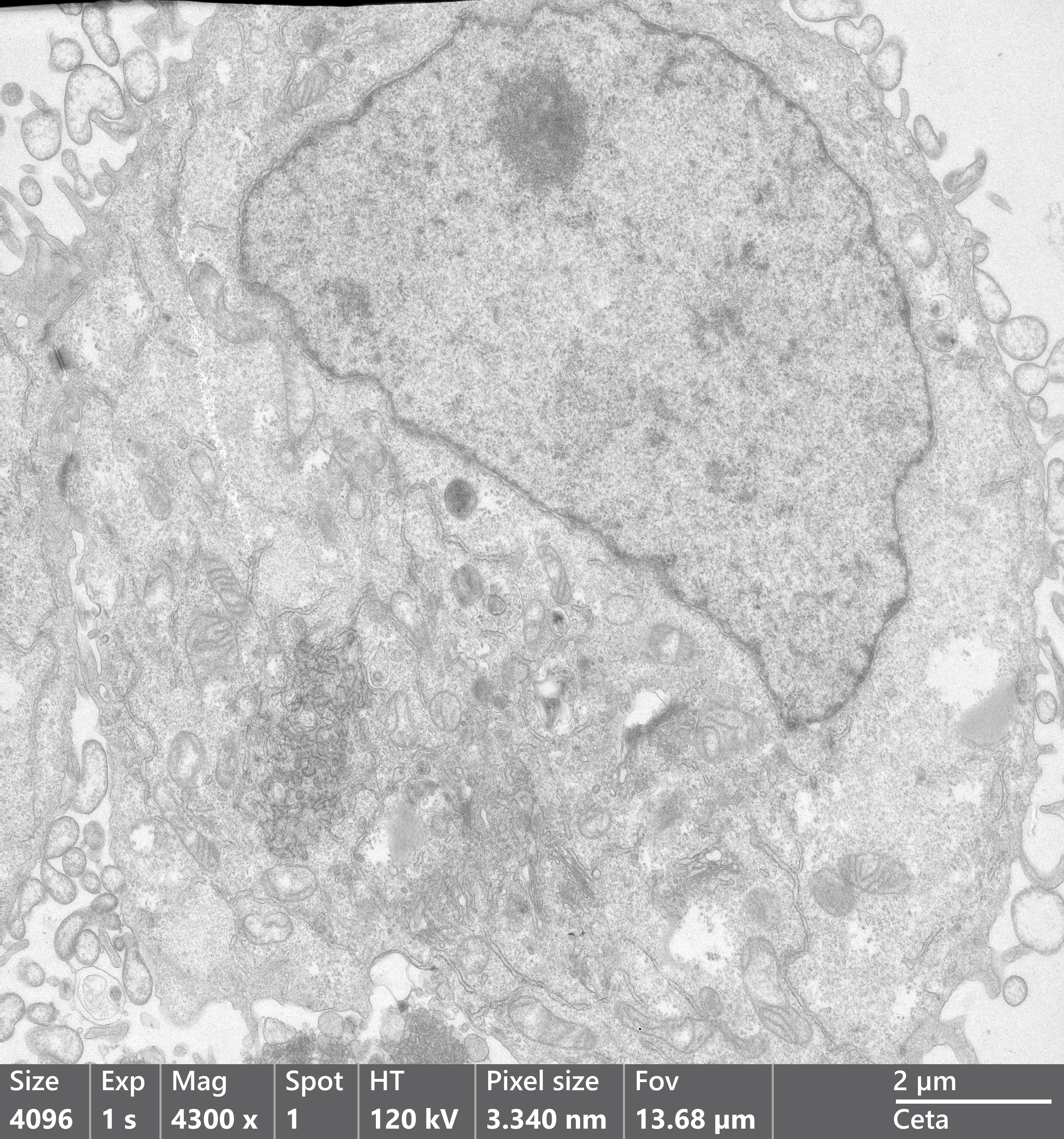

Supplement: Supplementary file 8 — Source data Fig. 5 [file 44318_2026_816_MOESM8_ESM.zip › J/ECHS1-KO.tif]

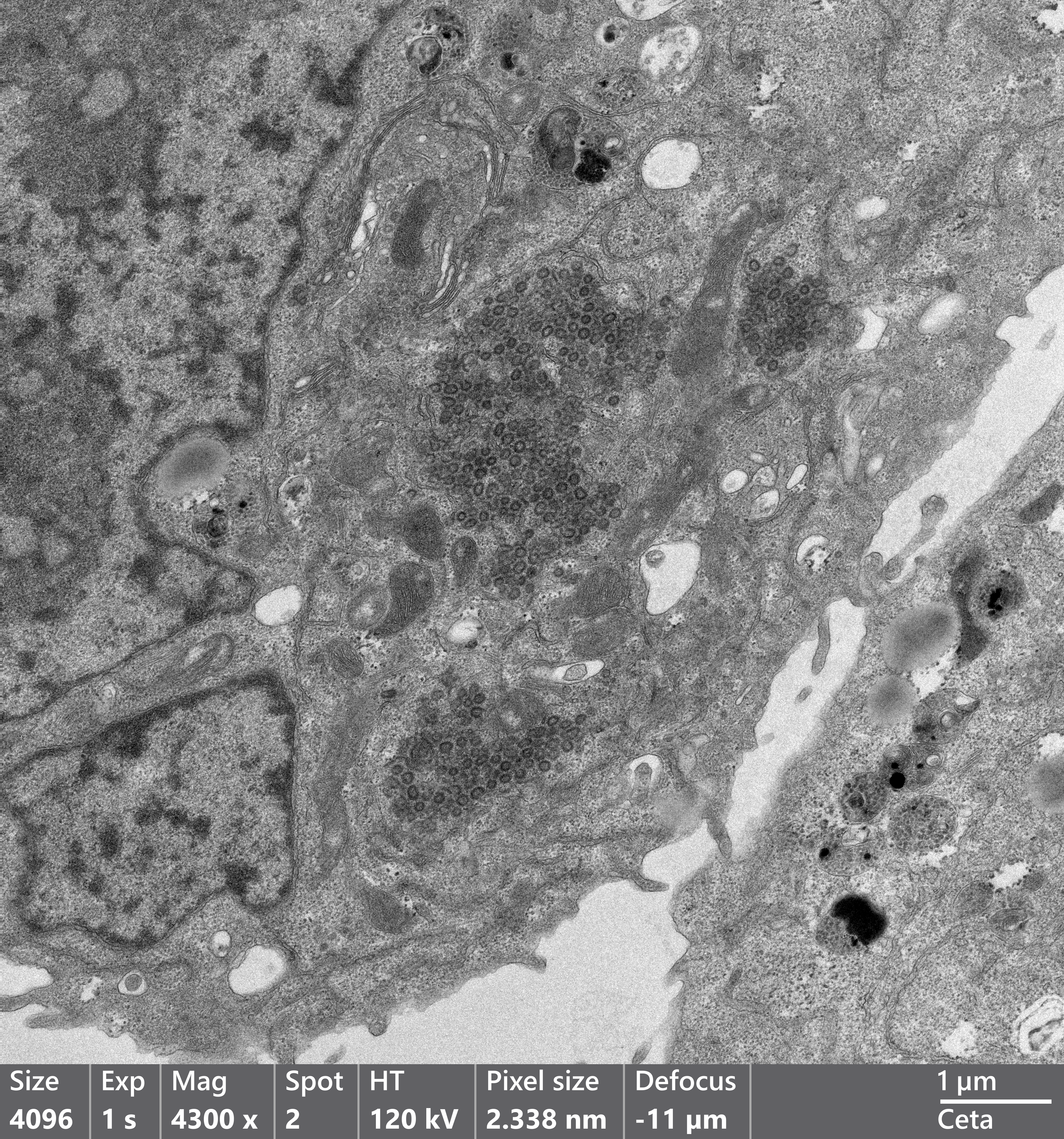

Supplement: Supplementary file 8 — Source data Fig. 5 [file 44318_2026_816_MOESM8_ESM.zip › J/ECHS1-Rescue.tif]

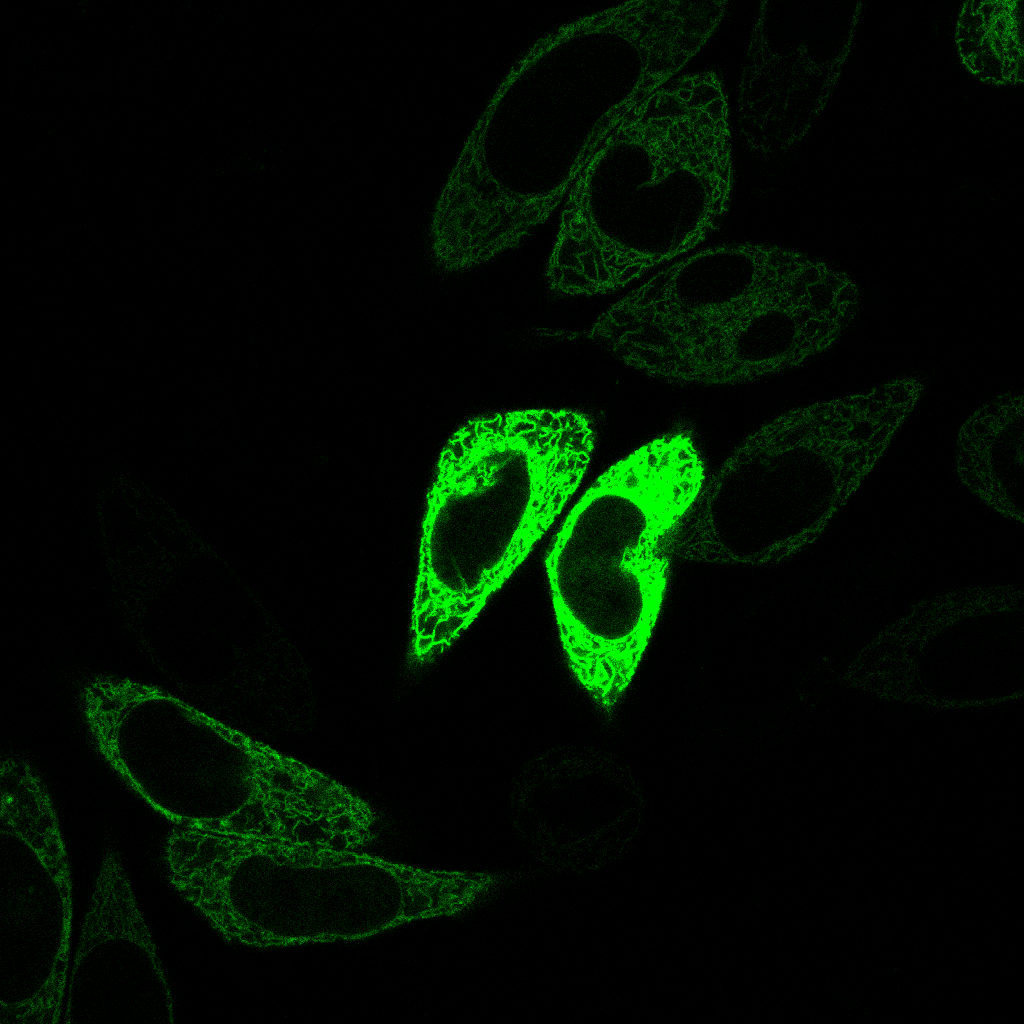

Supplement: Supplementary file 9 — Source data Fig. 6 [file 44318_2026_816_MOESM9_ESM.zip › A/Ctrl-GFP-NSP3.tif]

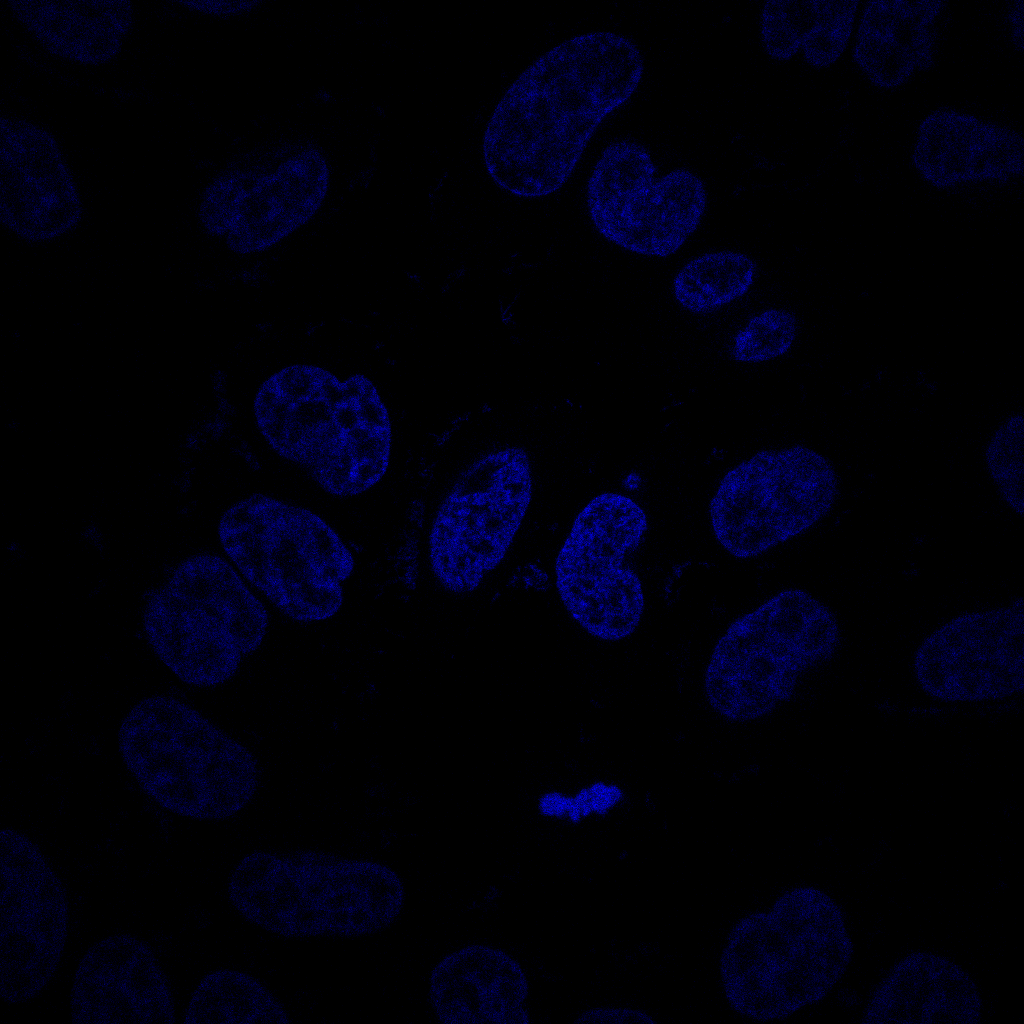

Supplement: Supplementary file 9 — Source data Fig. 6 [file 44318_2026_816_MOESM9_ESM.zip › A/Ctrl-Hoechst.tif]

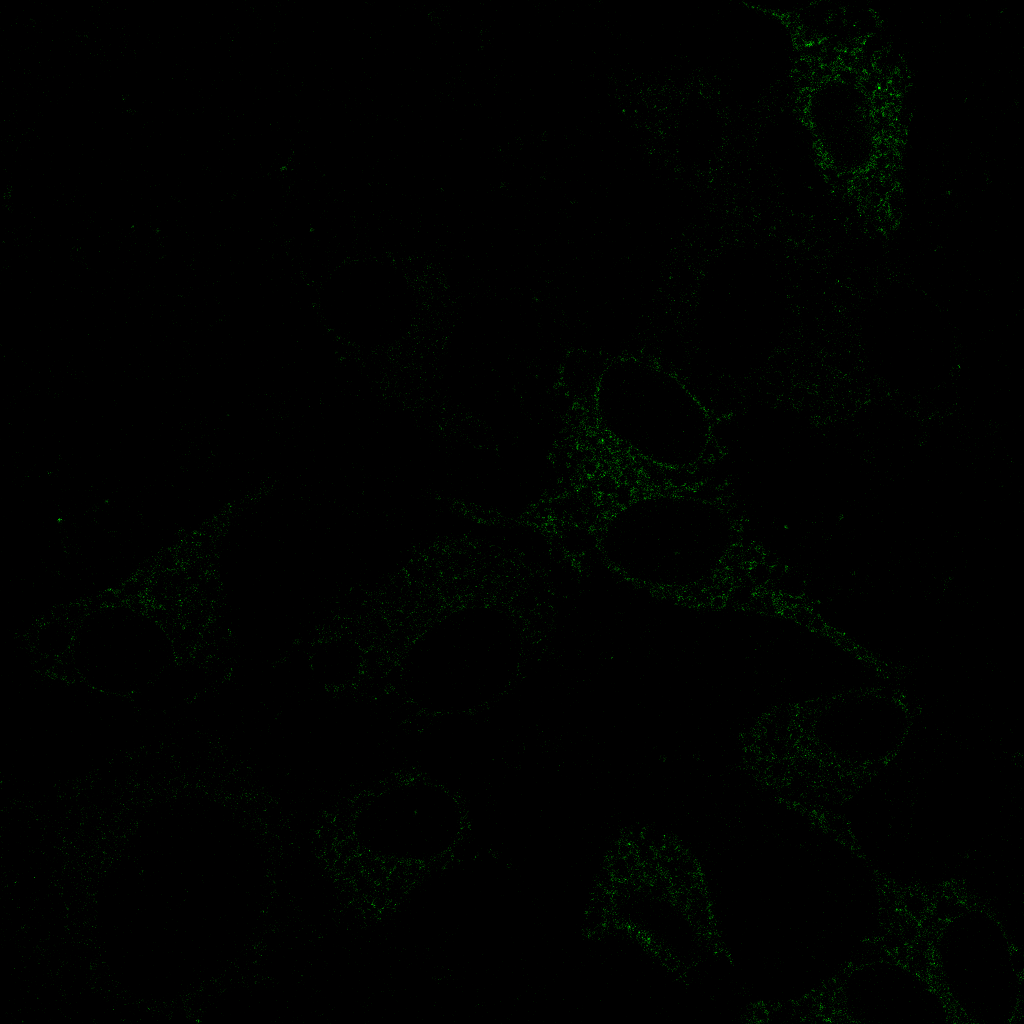

Supplement: Supplementary file 9 — Source data Fig. 6 [file 44318_2026_816_MOESM9_ESM.zip › A/ECHS1-KO-GFP-NSP3.tif]

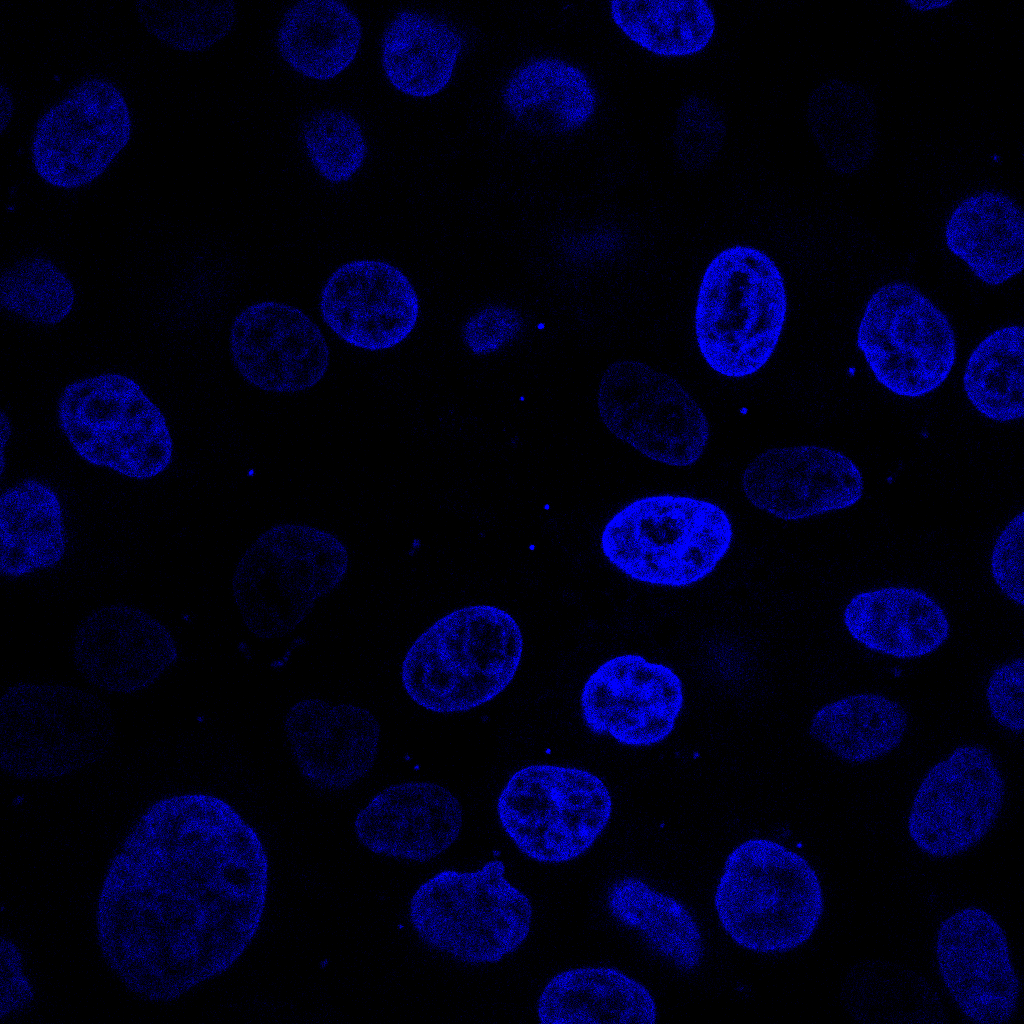

Supplement: Supplementary file 9 — Source data Fig. 6 [file 44318_2026_816_MOESM9_ESM.zip › A/ECHS1-KO-Hoechst.tif]

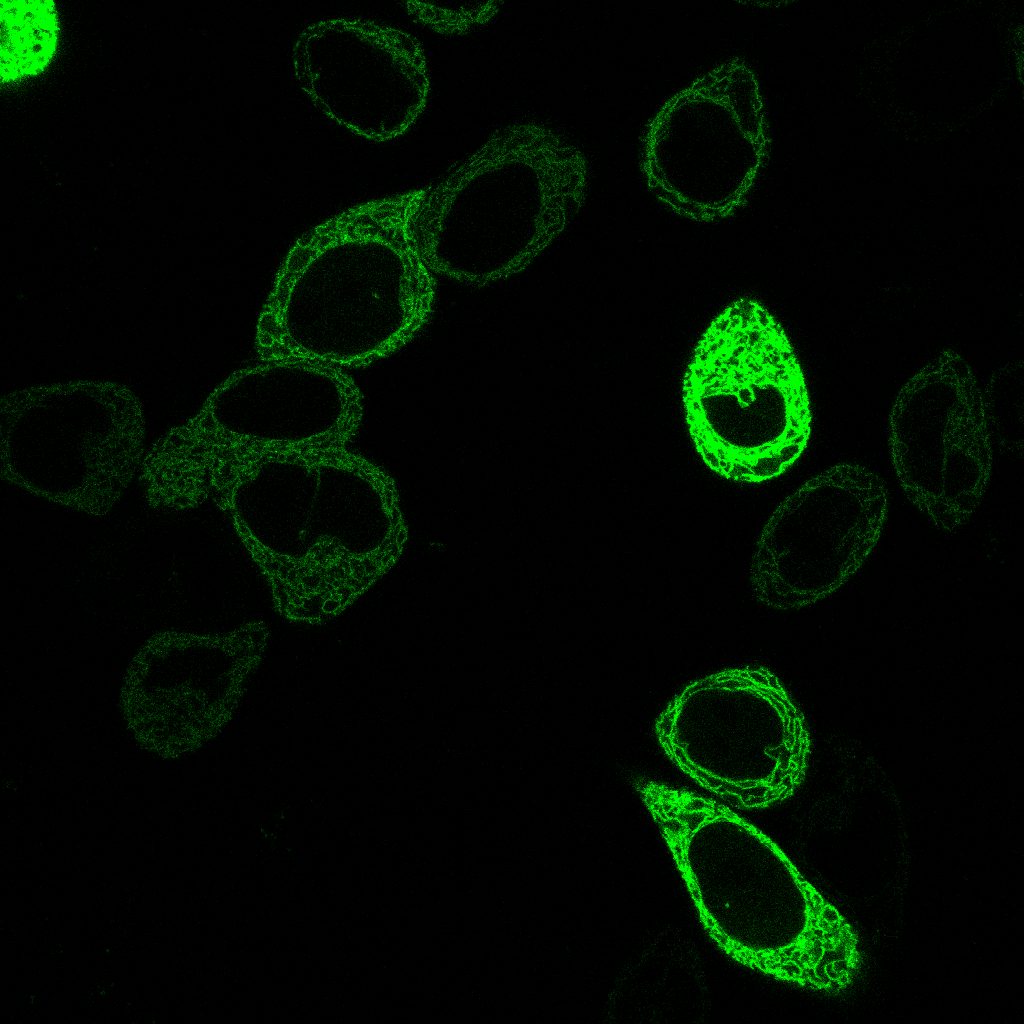

Supplement: Supplementary file 9 — Source data Fig. 6 [file 44318_2026_816_MOESM9_ESM.zip › A/ECHS1-Rescue-GFP-NSP3.tif]

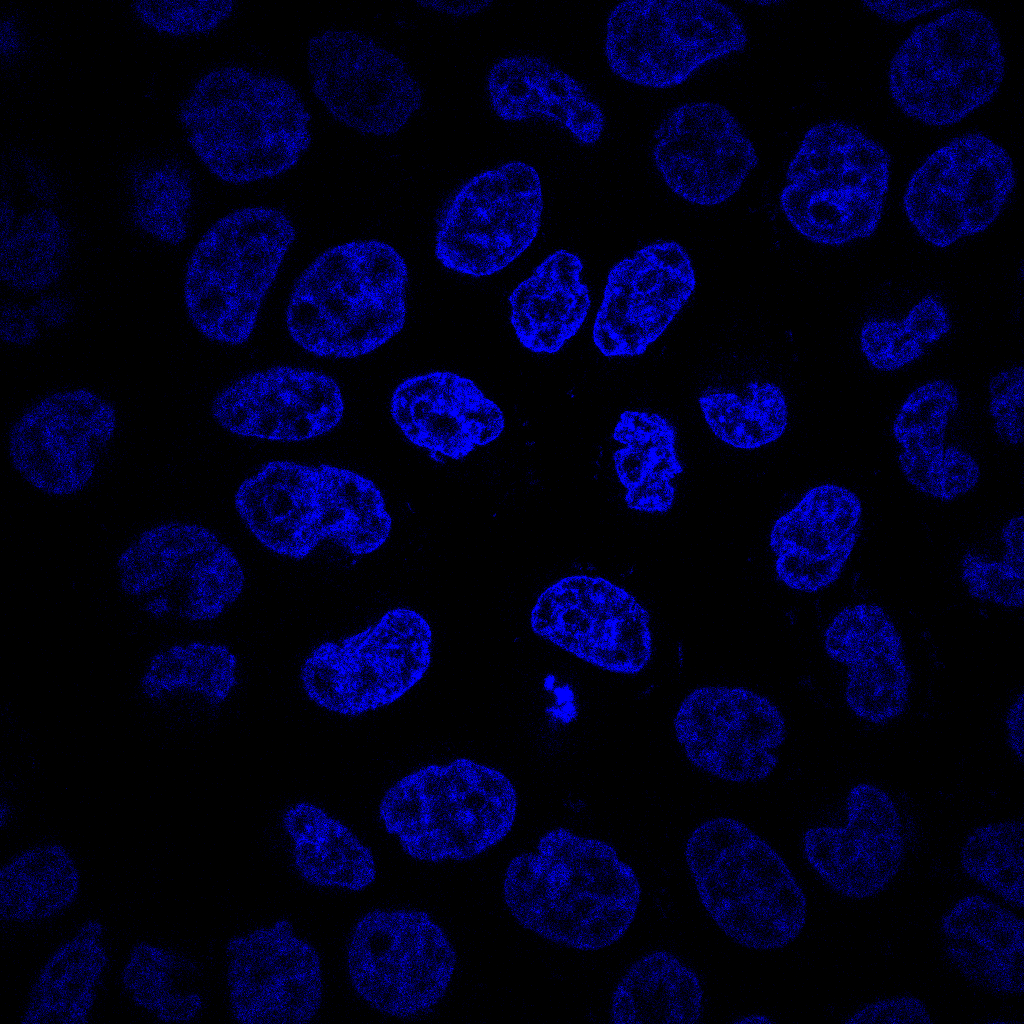

Supplement: Supplementary file 9 — Source data Fig. 6 [file 44318_2026_816_MOESM9_ESM.zip › A/ECHS1-Rescue-Hoechst.tif]

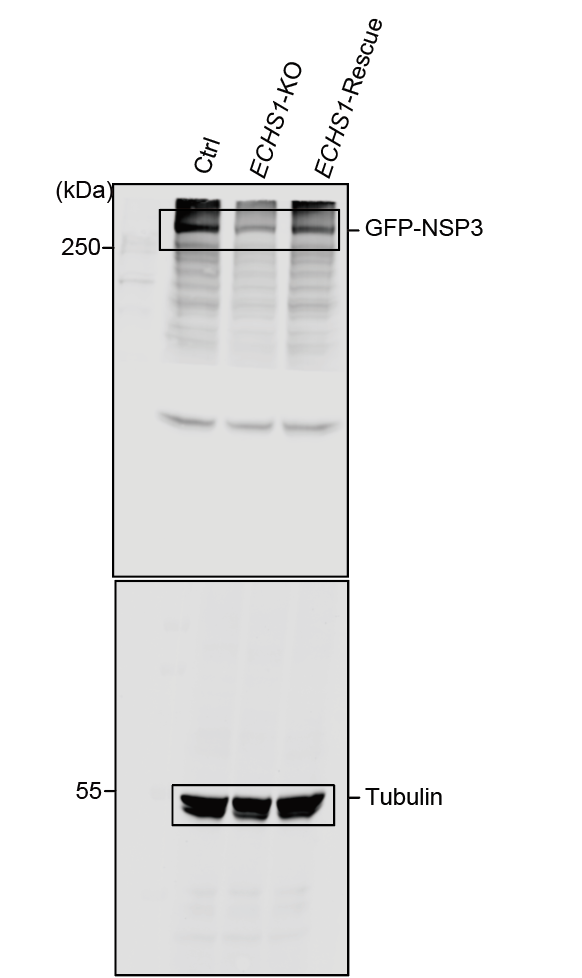

Supplement: Supplementary file 9 — Source data Fig. 6 [file 44318_2026_816_MOESM9_ESM.zip › B/GFP-NSP3+Tubulin.tif]

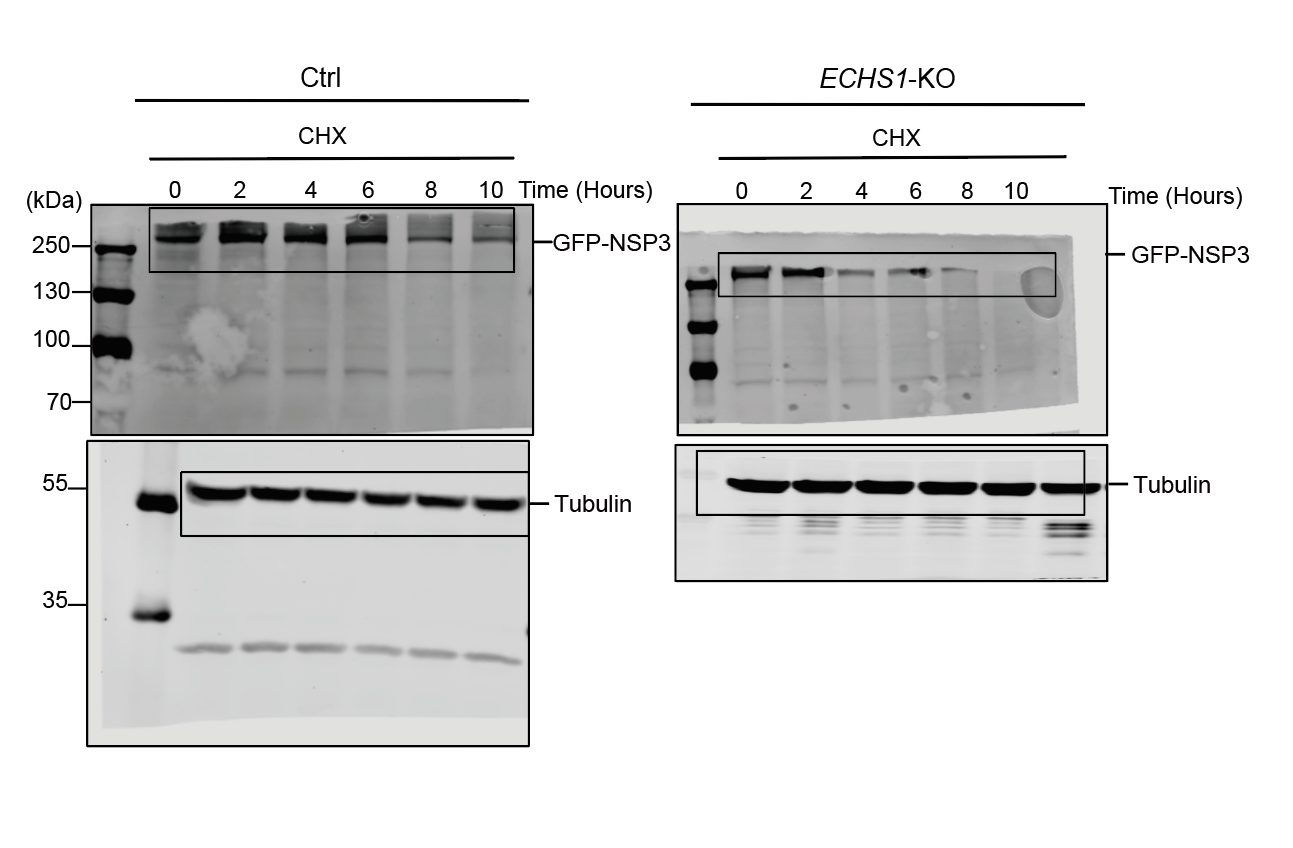

Supplement: Supplementary file 9 — Source data Fig. 6 [file 44318_2026_816_MOESM9_ESM.zip › C/GFP-NSP3+Tubulin.tif]

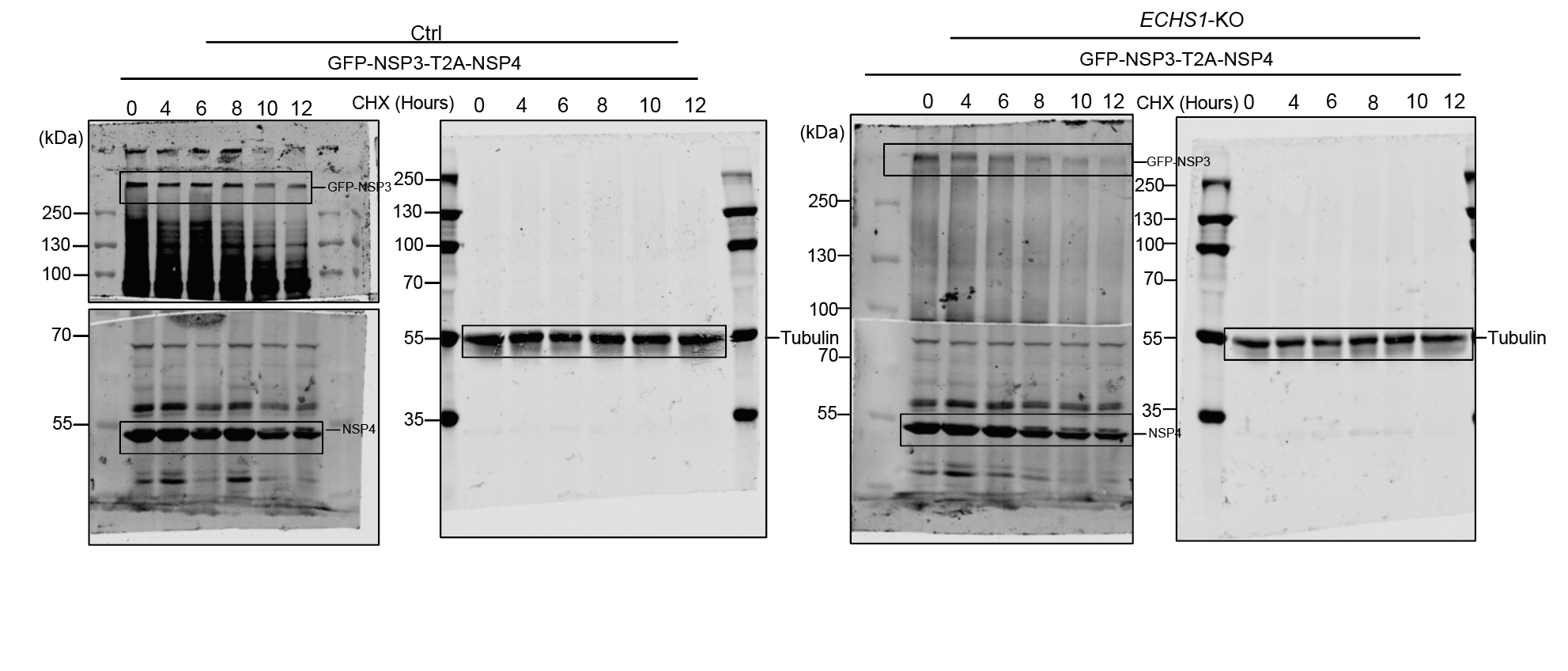

Supplement: Supplementary file 9 — Source data Fig. 6 [file 44318_2026_816_MOESM9_ESM.zip › D/GFP-NSP3+NSP4+Tubulin.tif]

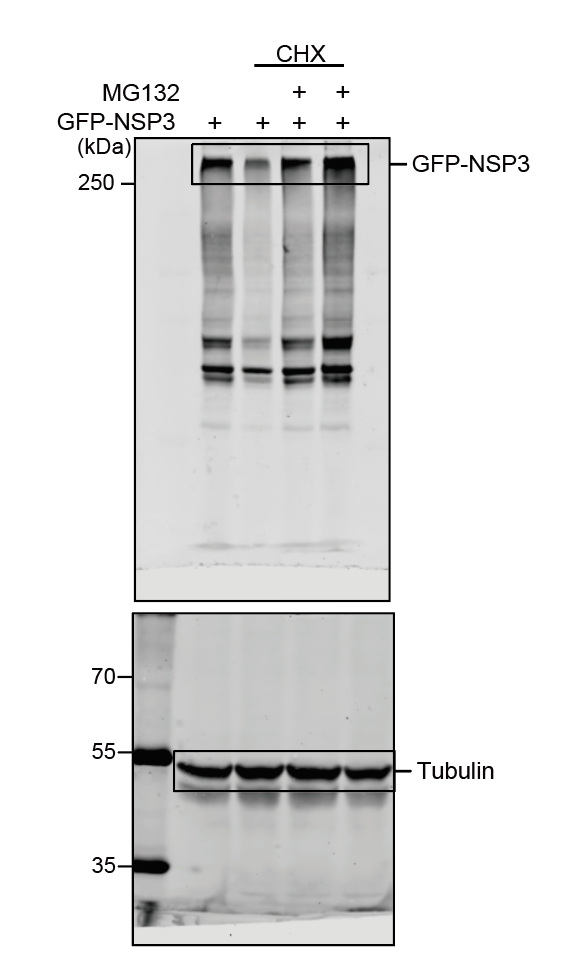

Supplement: Supplementary file 9 — Source data Fig. 6 [file 44318_2026_816_MOESM9_ESM.zip › E/GFP-NSP3+Tubulin.tif]

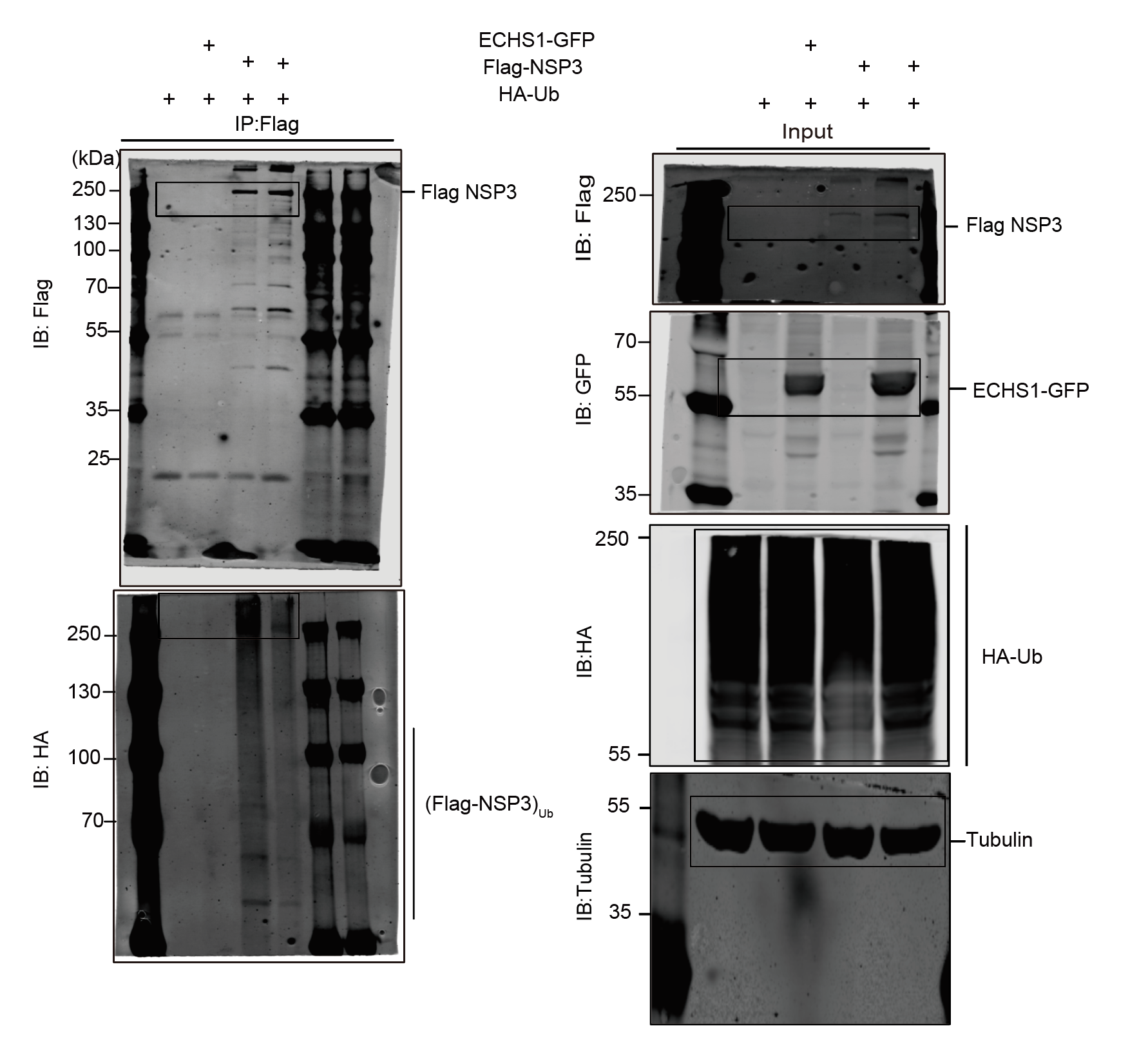

Supplement: Supplementary file 9 — Source data Fig. 6 [file 44318_2026_816_MOESM9_ESM.zip › F/Flag-NSP3+Ub+ECHS1-GFP+Tubulin.tif]

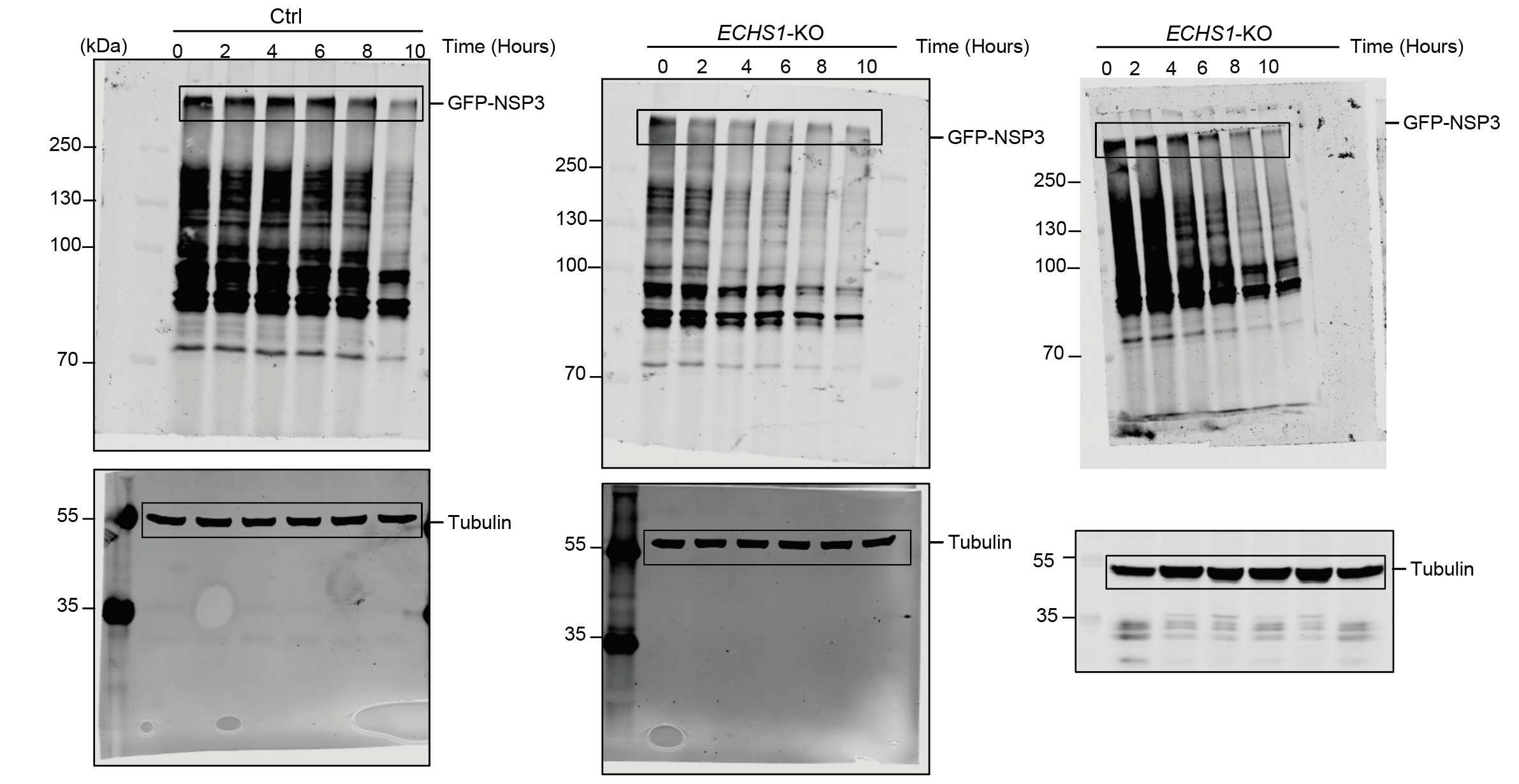

Supplement: Supplementary file 9 — Source data Fig. 6 [file 44318_2026_816_MOESM9_ESM.zip › G/GFP-NSP3+Tubulin.tif]

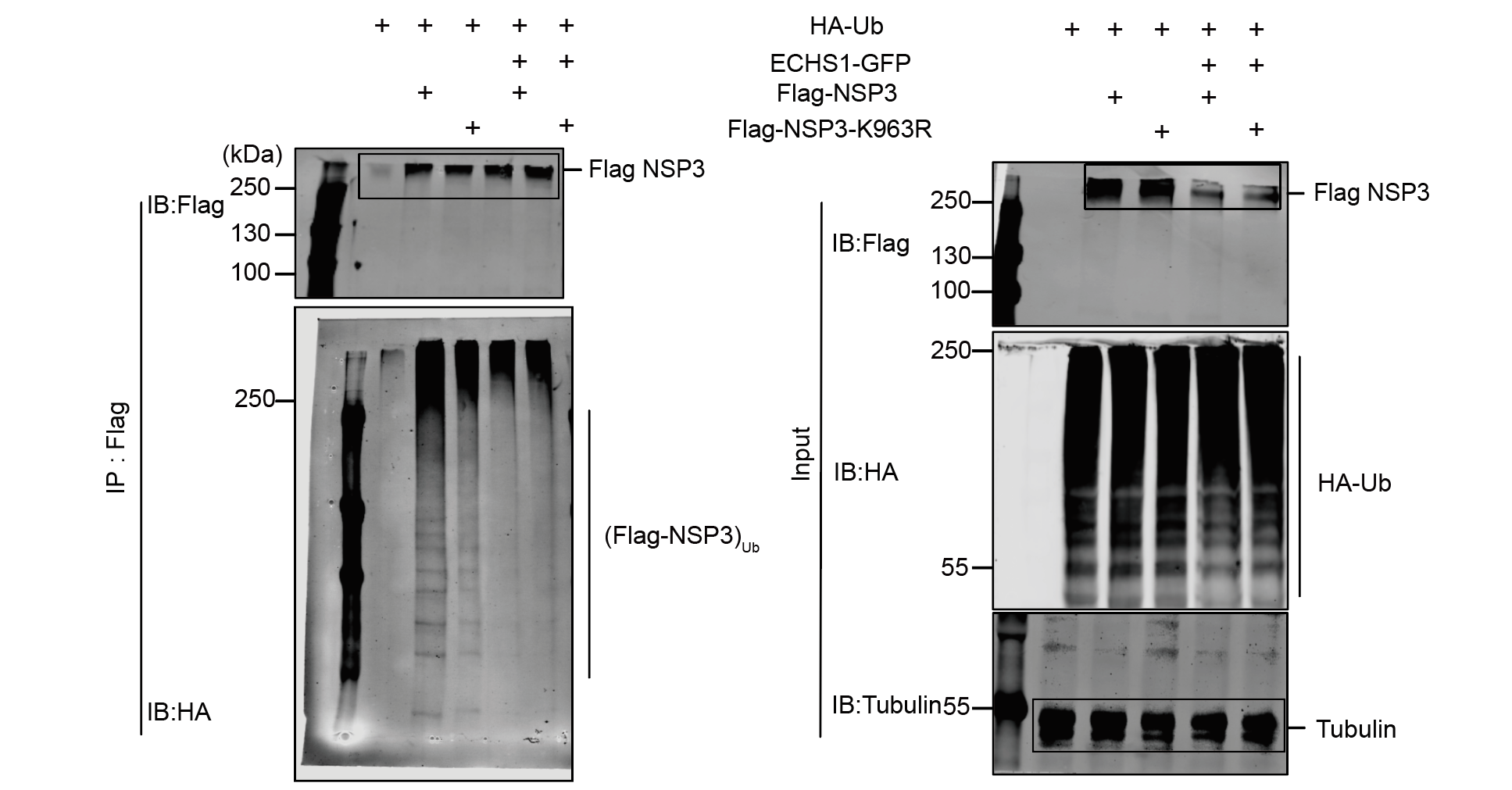

Supplement: Supplementary file 9 — Source data Fig. 6 [file 44318_2026_816_MOESM9_ESM.zip › H/Flag-NSP3+Ub+Tubulin.tif]

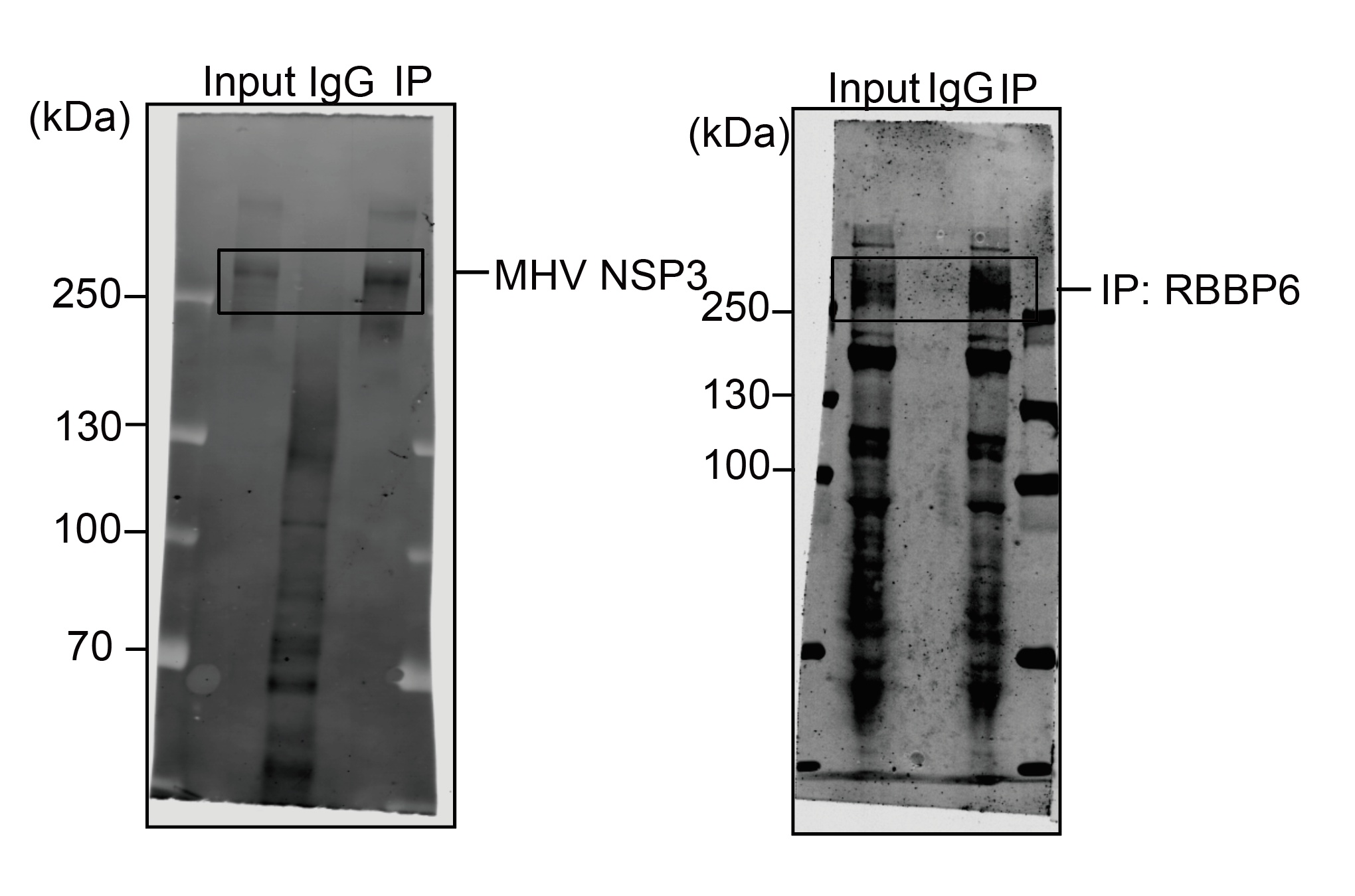

Supplement: Supplementary file 10 — Source data Fig. 7 [file 44318_2026_816_MOESM10_ESM.zip › B/MHV-NSP3+Tubulin.tif]

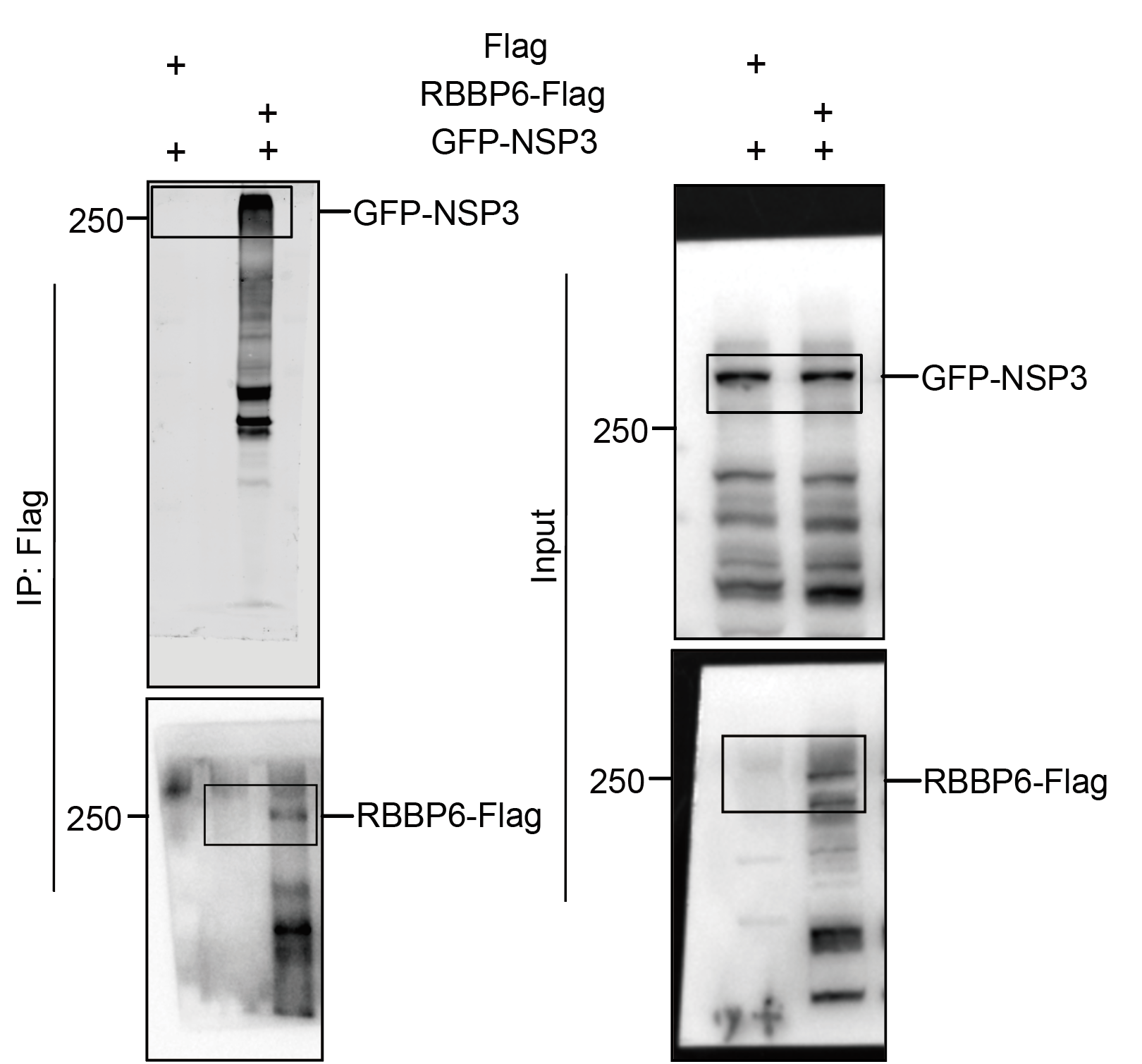

Supplement: Supplementary file 10 — Source data Fig. 7 [file 44318_2026_816_MOESM10_ESM.zip › C/GFP-NSP3+RBBP6-Flag.tif]

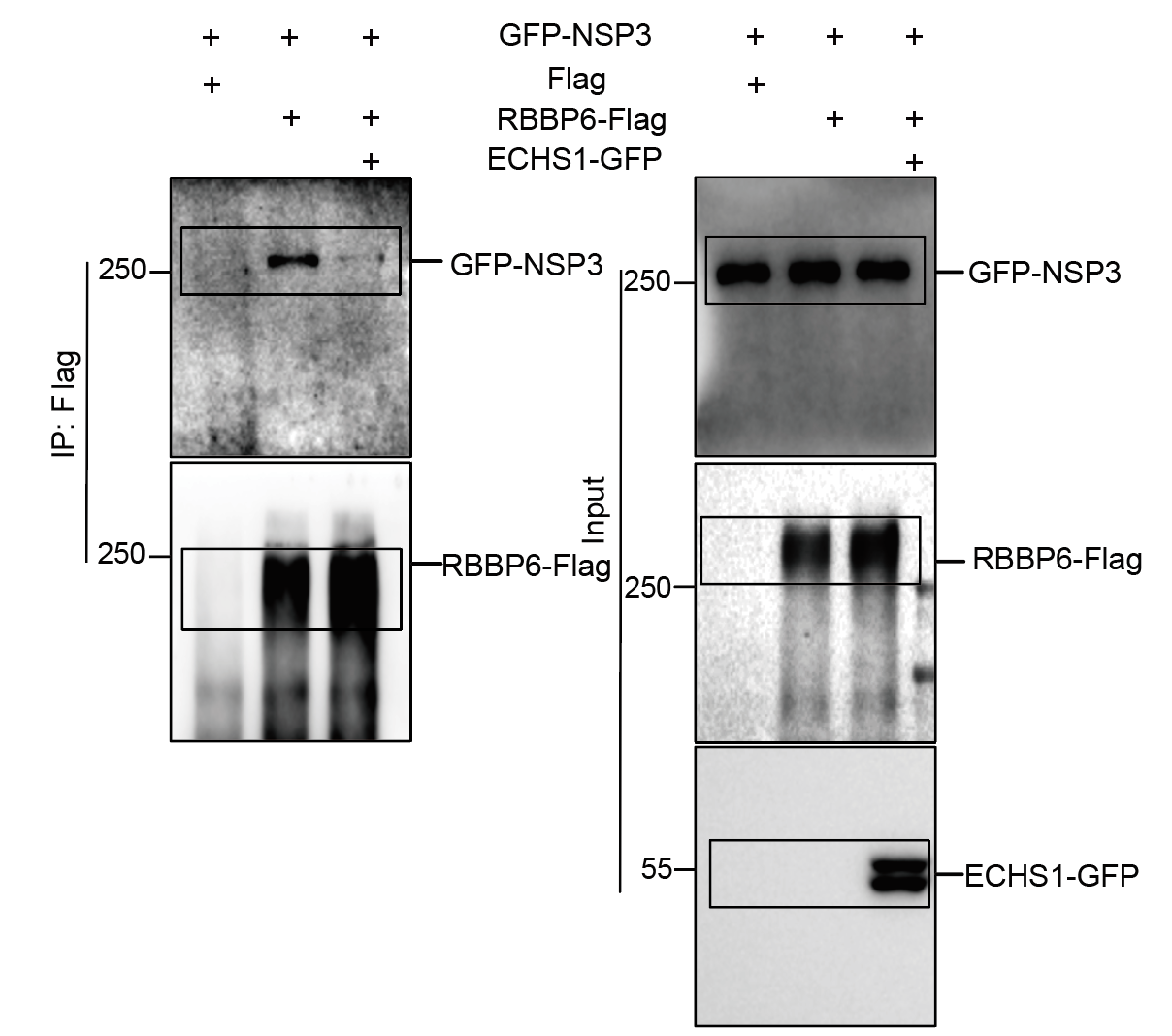

Supplement: Supplementary file 10 — Source data Fig. 7 [file 44318_2026_816_MOESM10_ESM.zip › D/GFP-NSP3+RBBP6-Flag+ECHS1-GFP.tif]

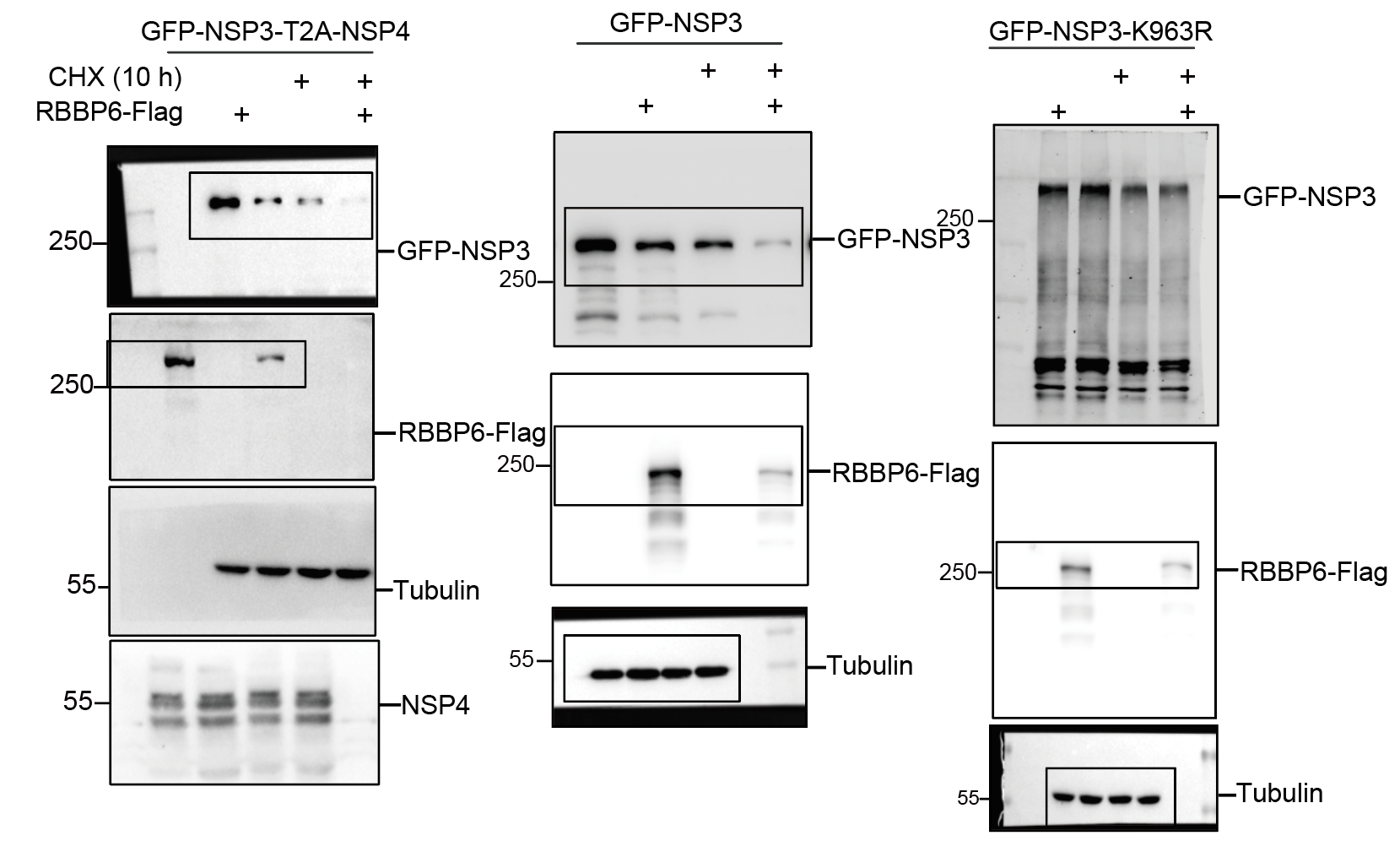

Supplement: Supplementary file 10 — Source data Fig. 7 [file 44318_2026_816_MOESM10_ESM.zip › E/GFP-NSP3+RBBP6-Flag+NSP4+Tubulin.tif]

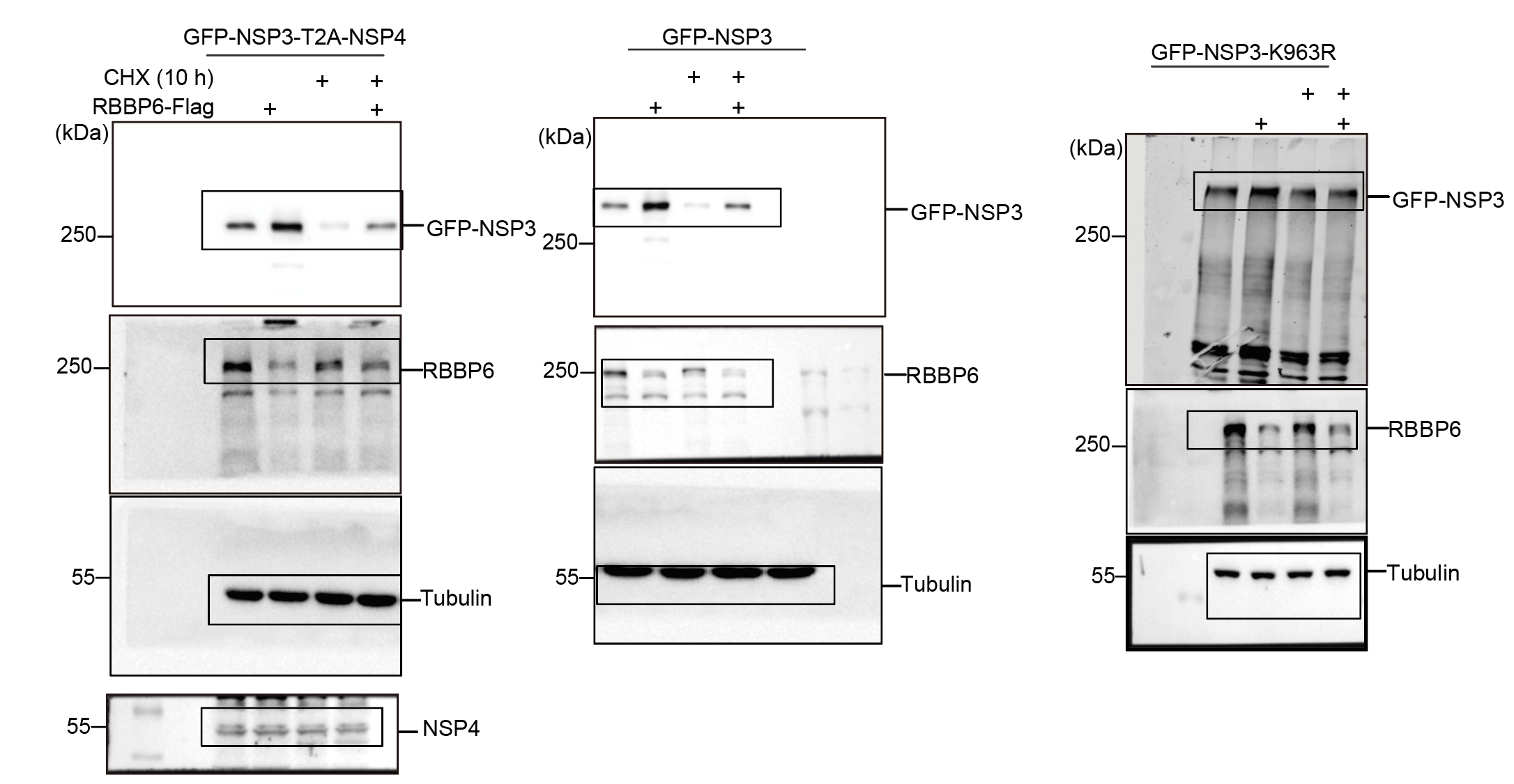

Supplement: Supplementary file 10 — Source data Fig. 7 [file 44318_2026_816_MOESM10_ESM.zip › F/GFP-NSP3+RBBP6+NSP4+Tubulin.tif]

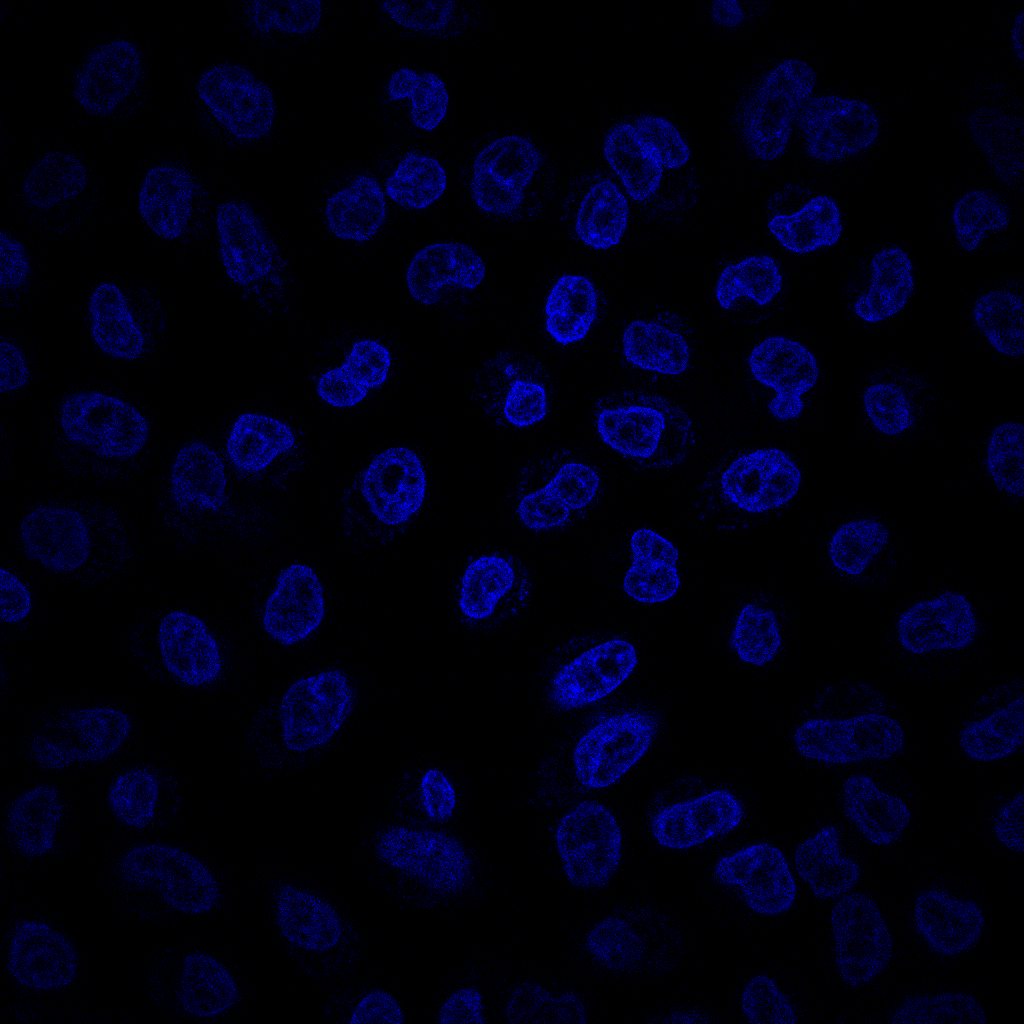

Supplement: Supplementary file 11 — Figure EV1 Source Data [file 44318_2026_816_MOESM11_ESM.zip › A/DOX-0h.tif]

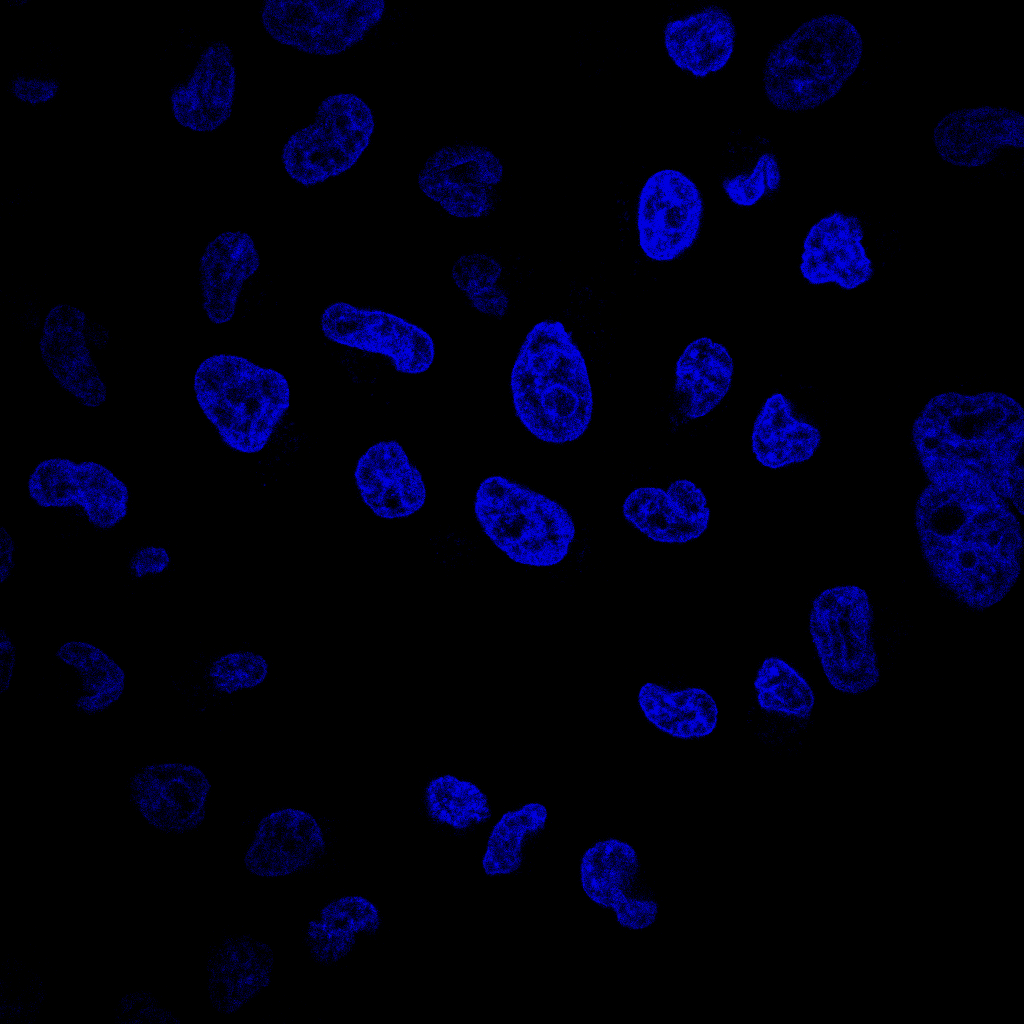

Supplement: Supplementary file 11 — Figure EV1 Source Data [file 44318_2026_816_MOESM11_ESM.zip › A/DOX-12h.nd2 .tif]

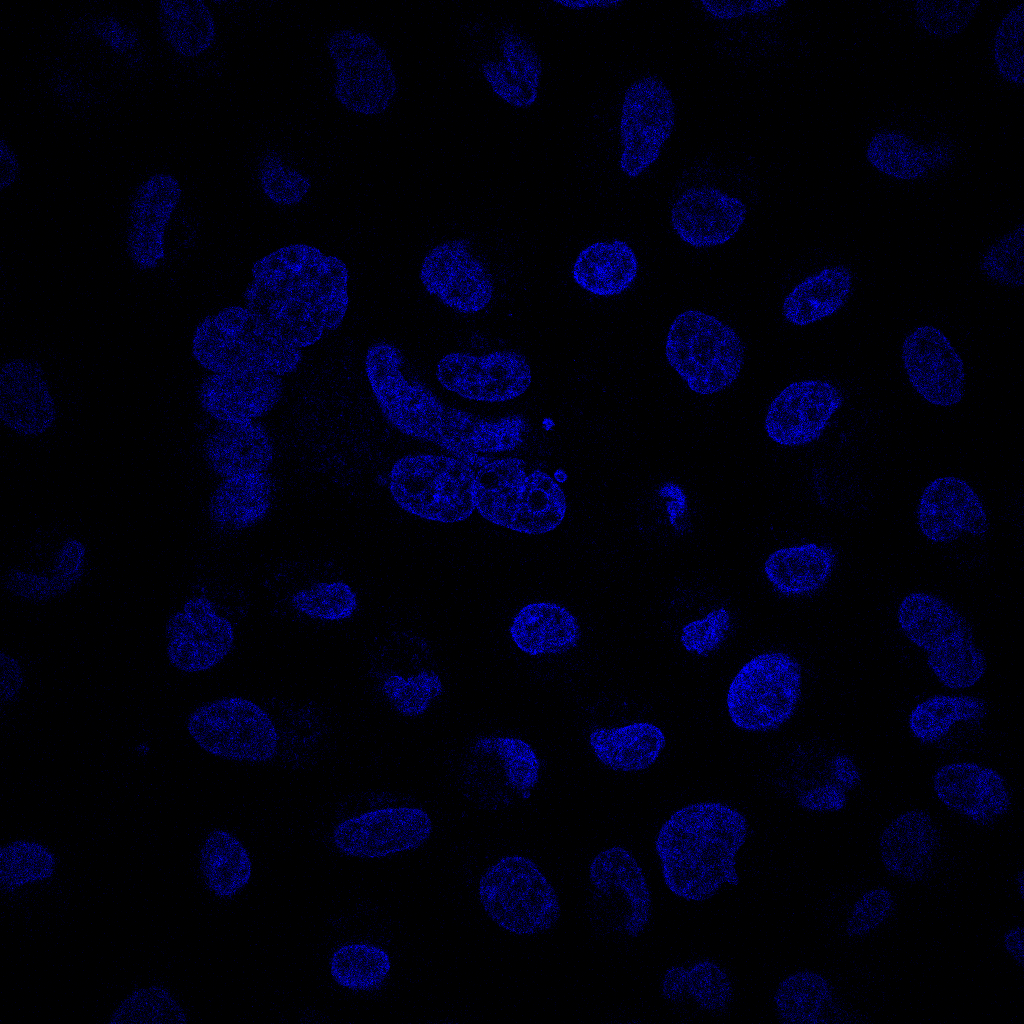

Supplement: Supplementary file 11 — Figure EV1 Source Data [file 44318_2026_816_MOESM11_ESM.zip › A/DOX-24h.tif]

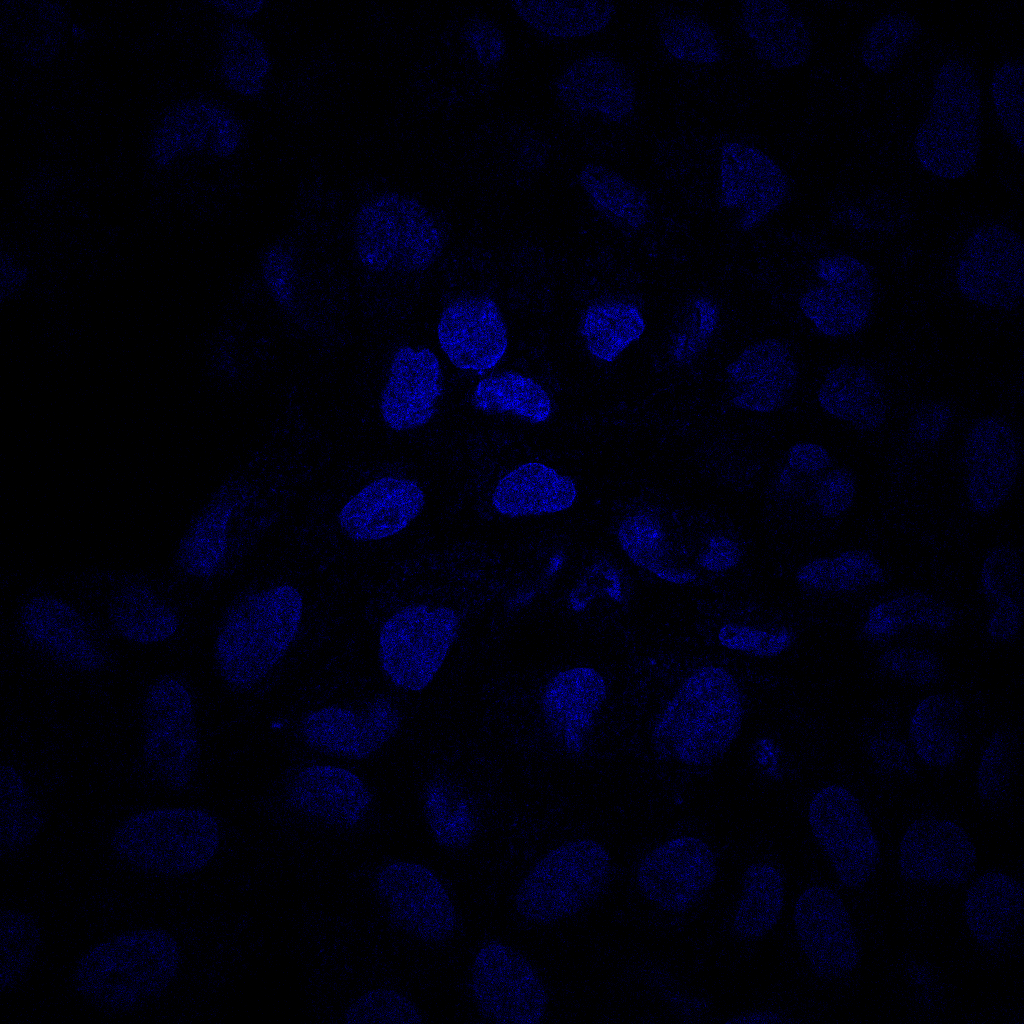

Supplement: Supplementary file 11 — Figure EV1 Source Data [file 44318_2026_816_MOESM11_ESM.zip › A/DOX-36h.tif]

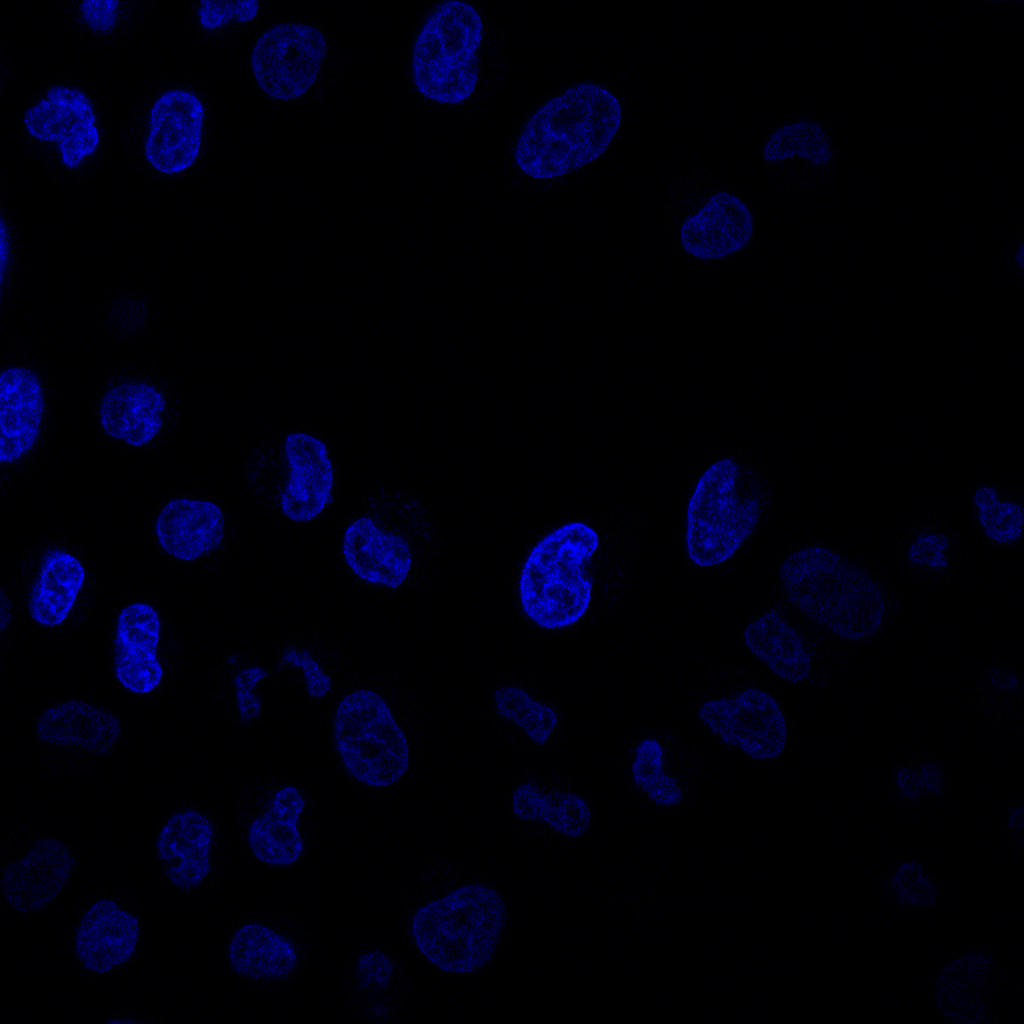

Supplement: Supplementary file 11 — Figure EV1 Source Data [file 44318_2026_816_MOESM11_ESM.zip › A/DOX-6h.tif]

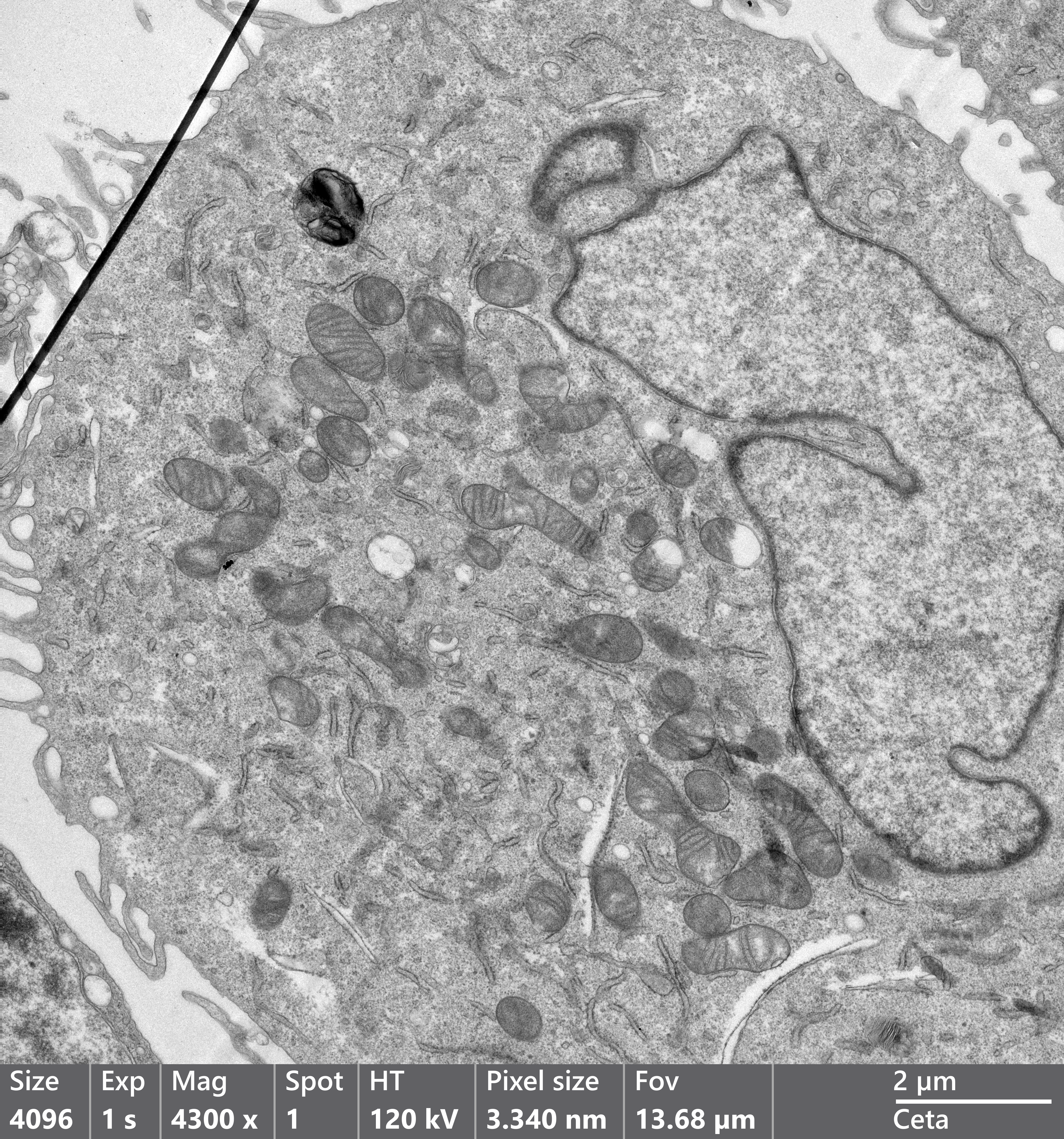

Supplement: Supplementary file 11 — Figure EV1 Source Data [file 44318_2026_816_MOESM11_ESM.zip › B/Figure EV1B 0h.tif]

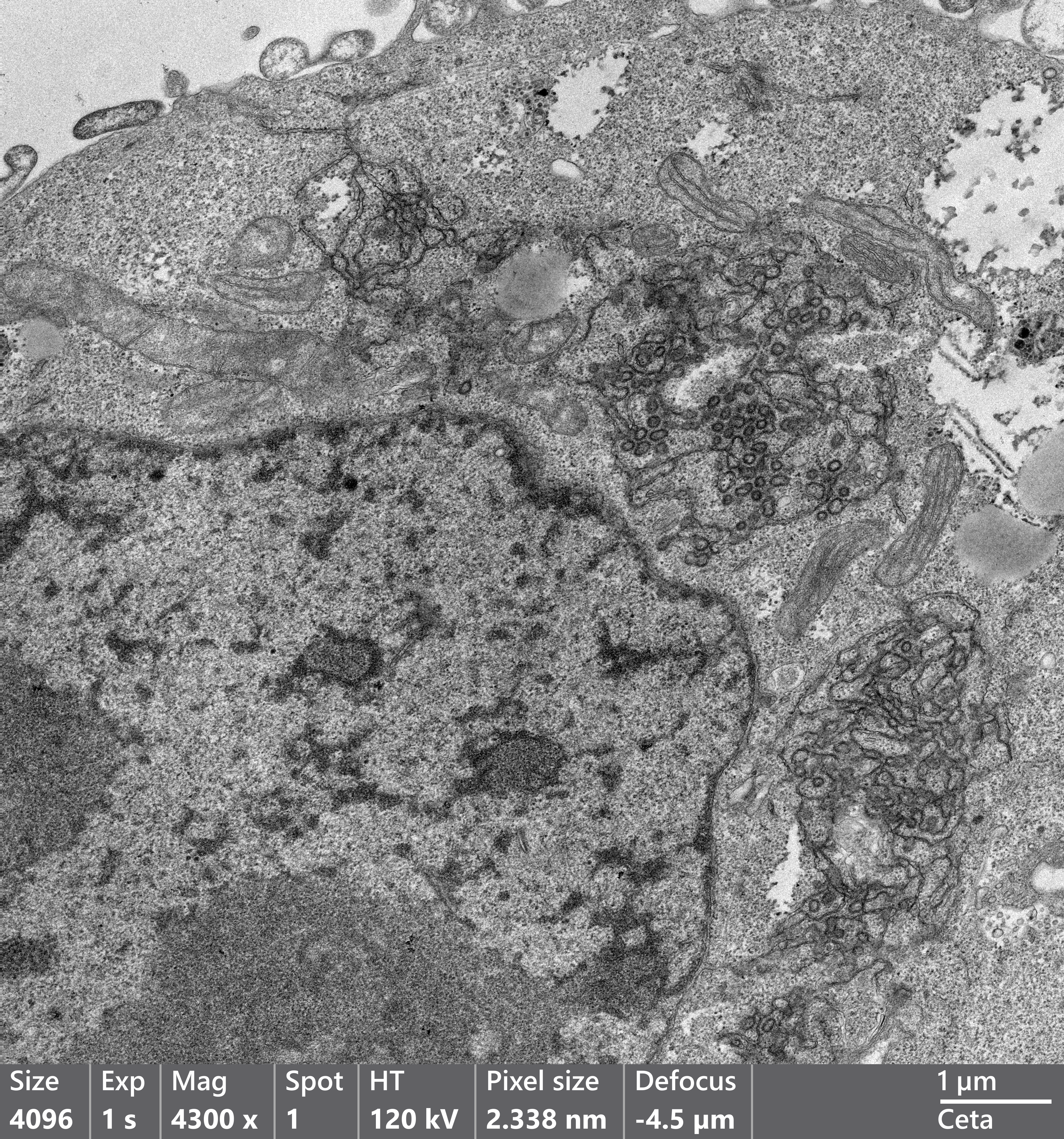

Supplement: Supplementary file 11 — Figure EV1 Source Data [file 44318_2026_816_MOESM11_ESM.zip › B/Figure EV1B DOX 12h.tif]

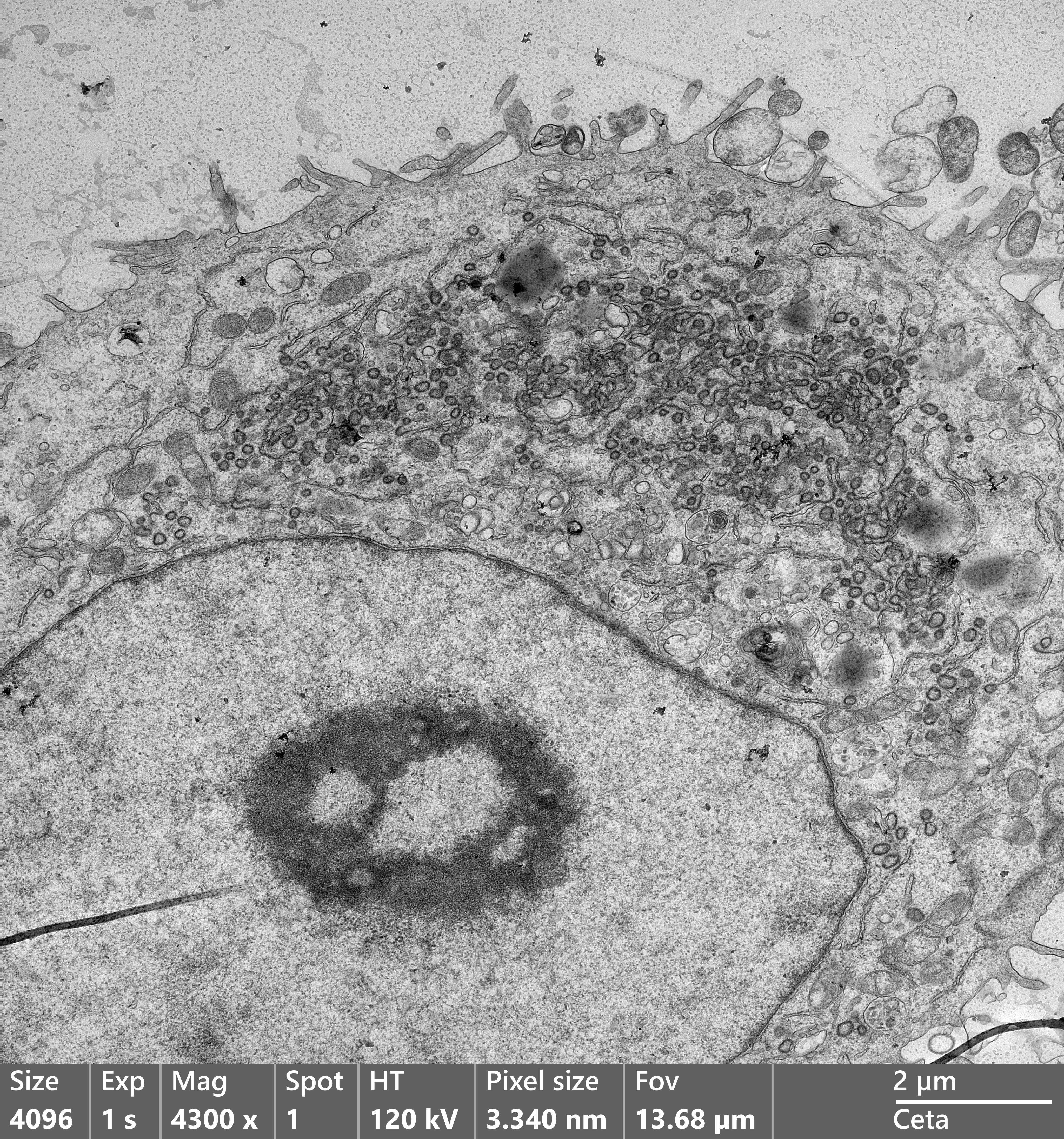

Supplement: Supplementary file 11 — Figure EV1 Source Data [file 44318_2026_816_MOESM11_ESM.zip › B/Figure EV1B DOX 24h.tif]

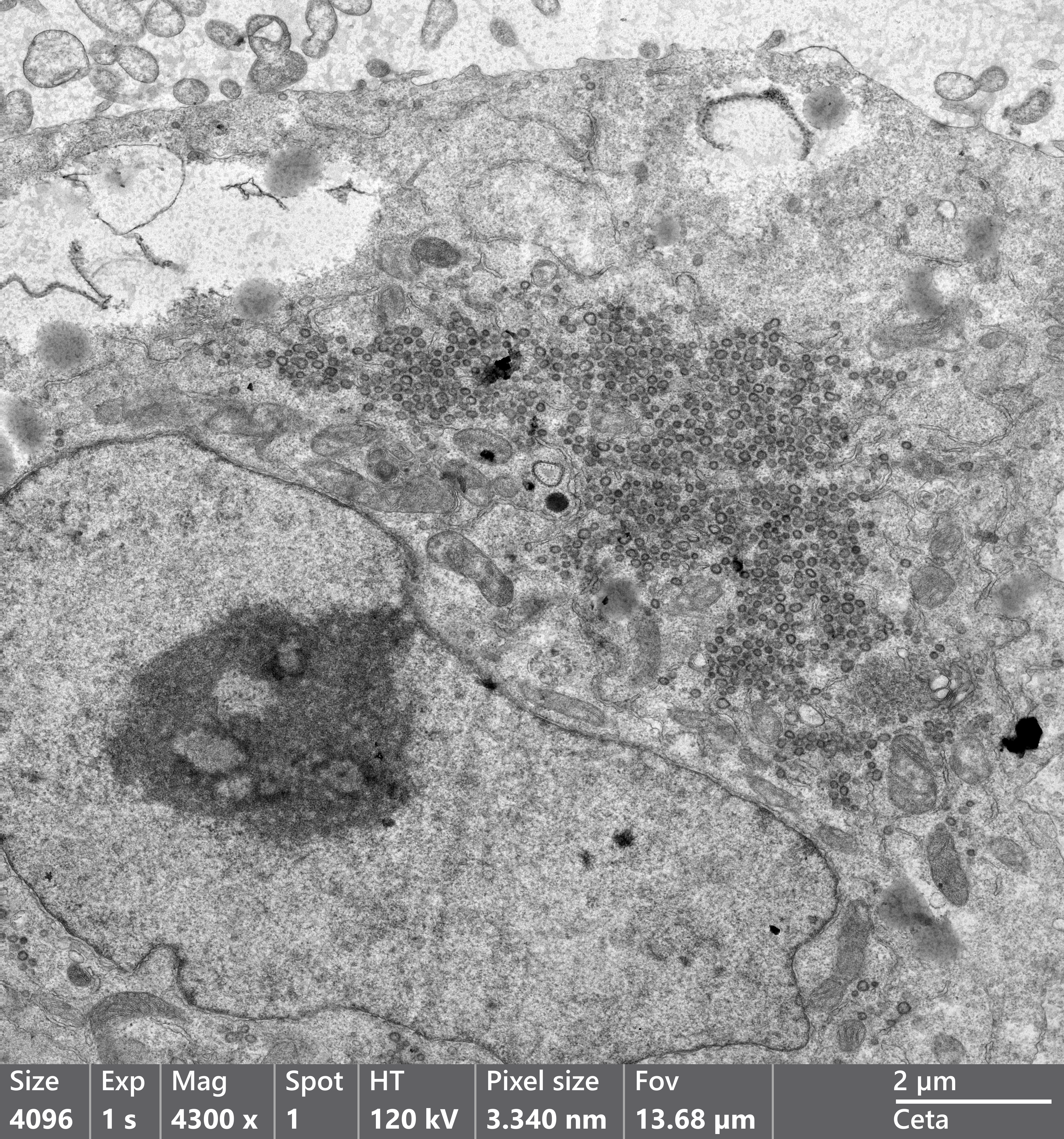

Supplement: Supplementary file 11 — Figure EV1 Source Data [file 44318_2026_816_MOESM11_ESM.zip › B/Figure EV1B DOX 36h.tif]

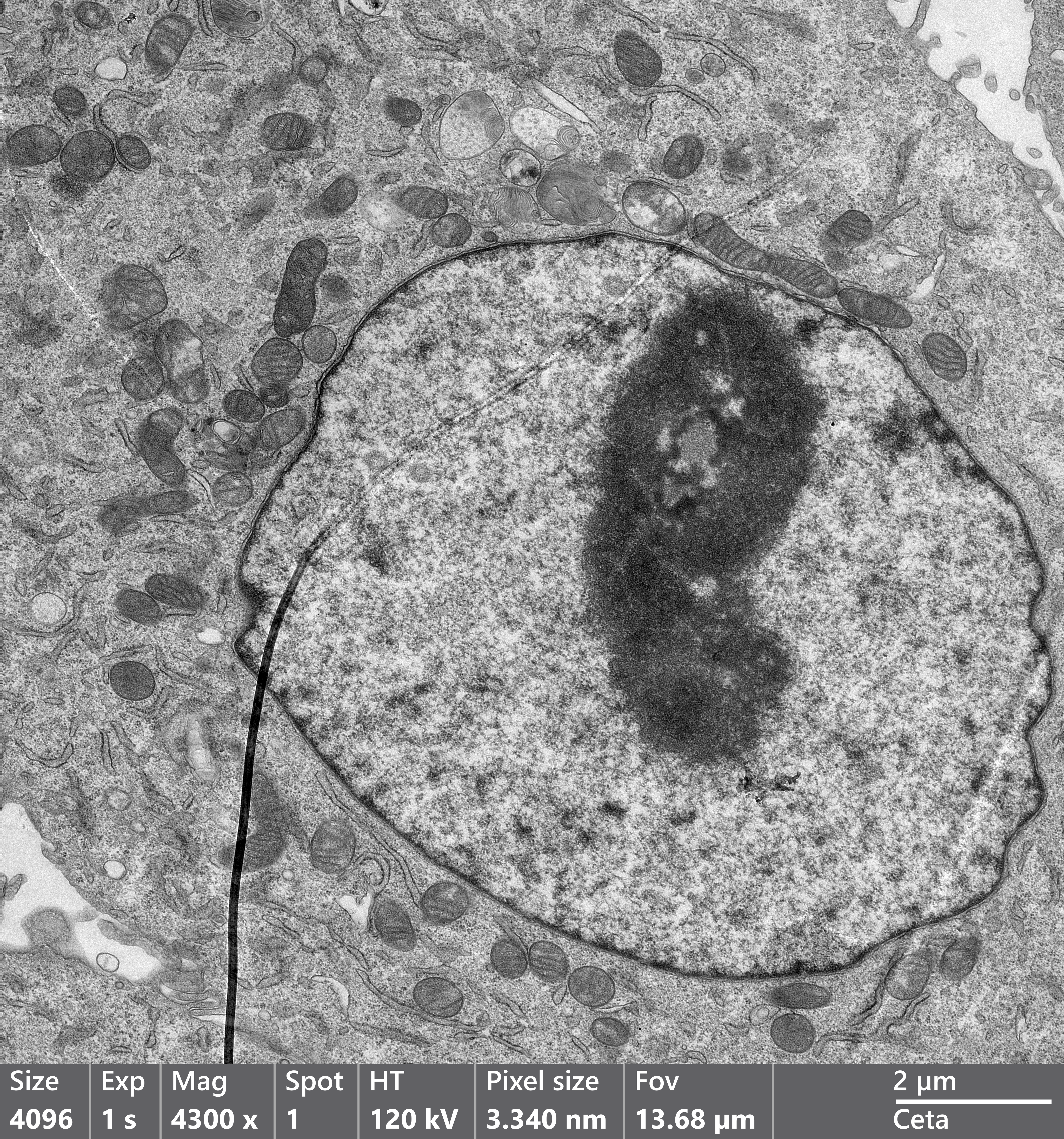

Supplement: Supplementary file 11 — Figure EV1 Source Data [file 44318_2026_816_MOESM11_ESM.zip › B/Figure EV1B DOX 6h.tif]

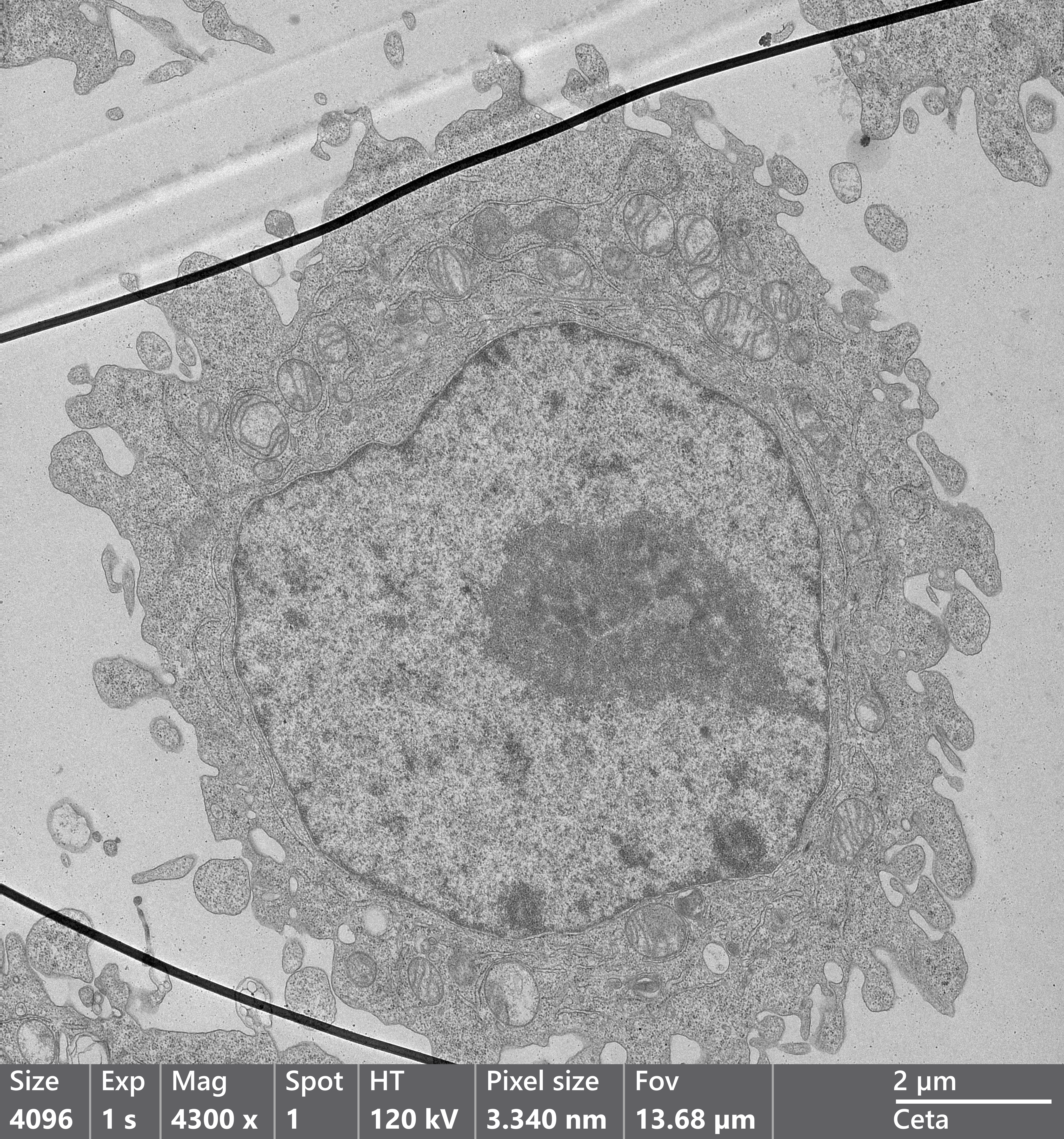

Supplement: Supplementary file 12 — Figure EV2 Source Data [file 44318_2026_816_MOESM12_ESM.zip › A/Figure S2A-MHV 0H.tif]

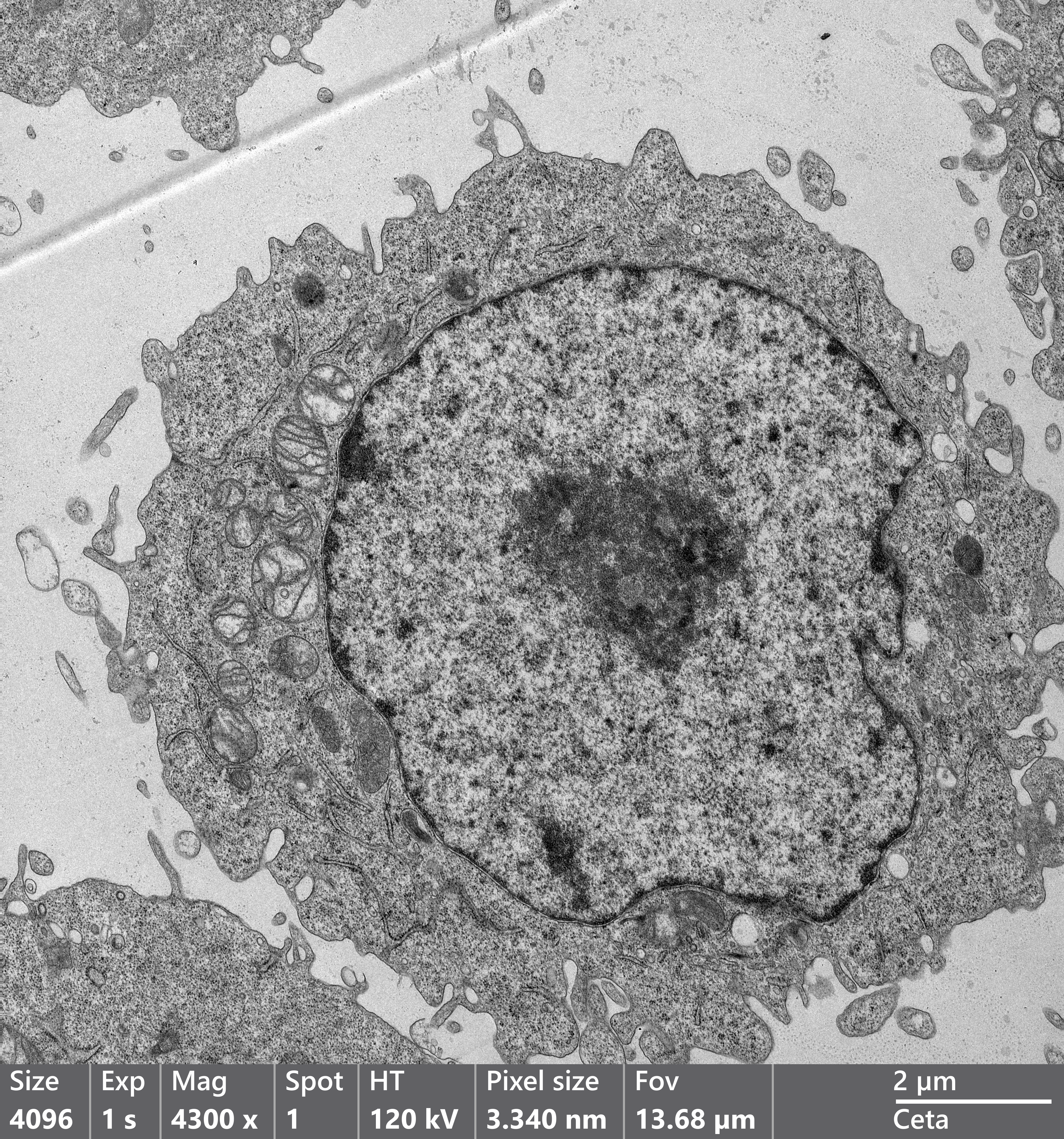

Supplement: Supplementary file 12 — Figure EV2 Source Data [file 44318_2026_816_MOESM12_ESM.zip › A/Figure S2A-MHV 2H.tif]

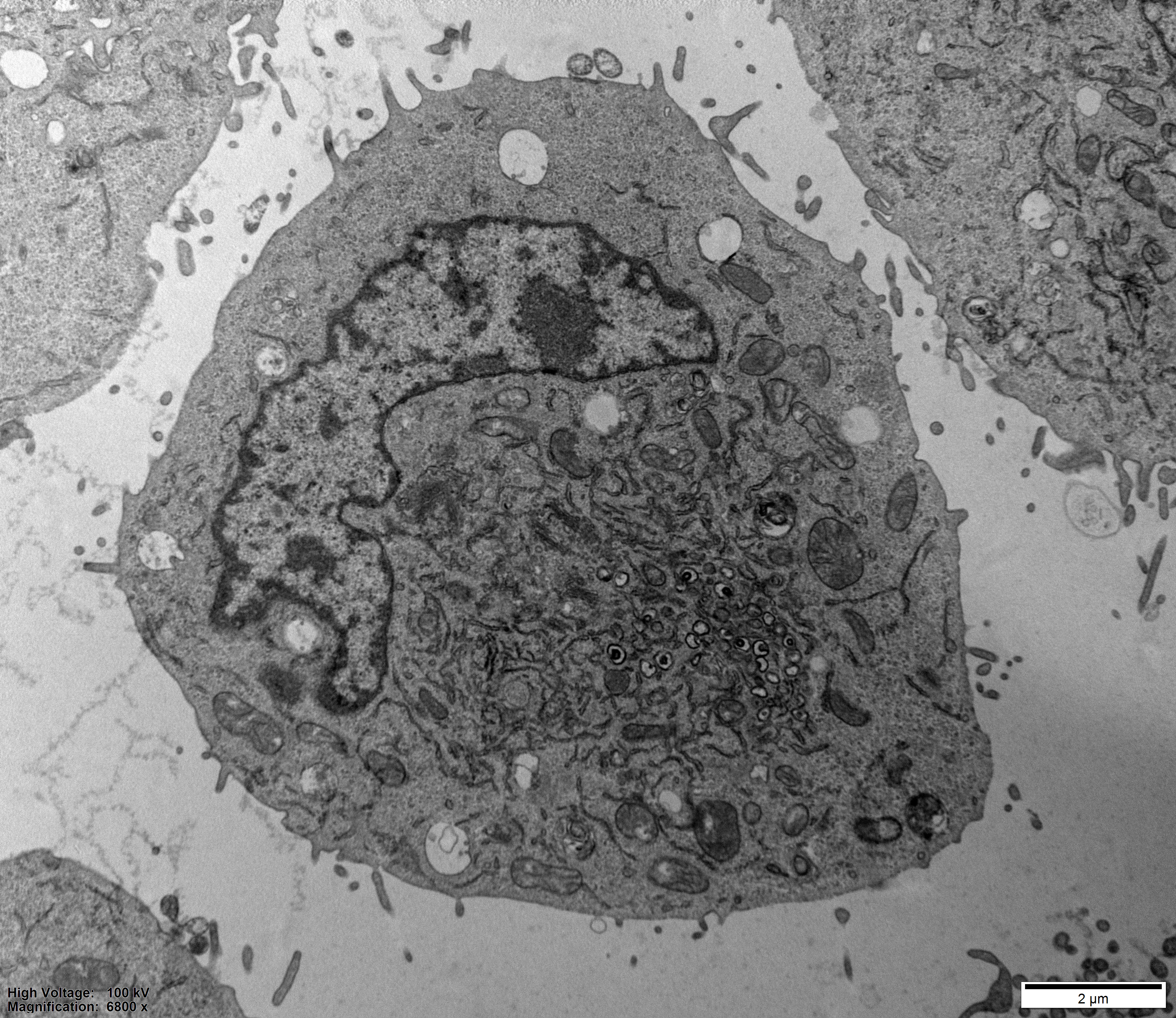

Supplement: Supplementary file 12 — Figure EV2 Source Data [file 44318_2026_816_MOESM12_ESM.zip › A/Figure S2A-MHV 4H.tif]

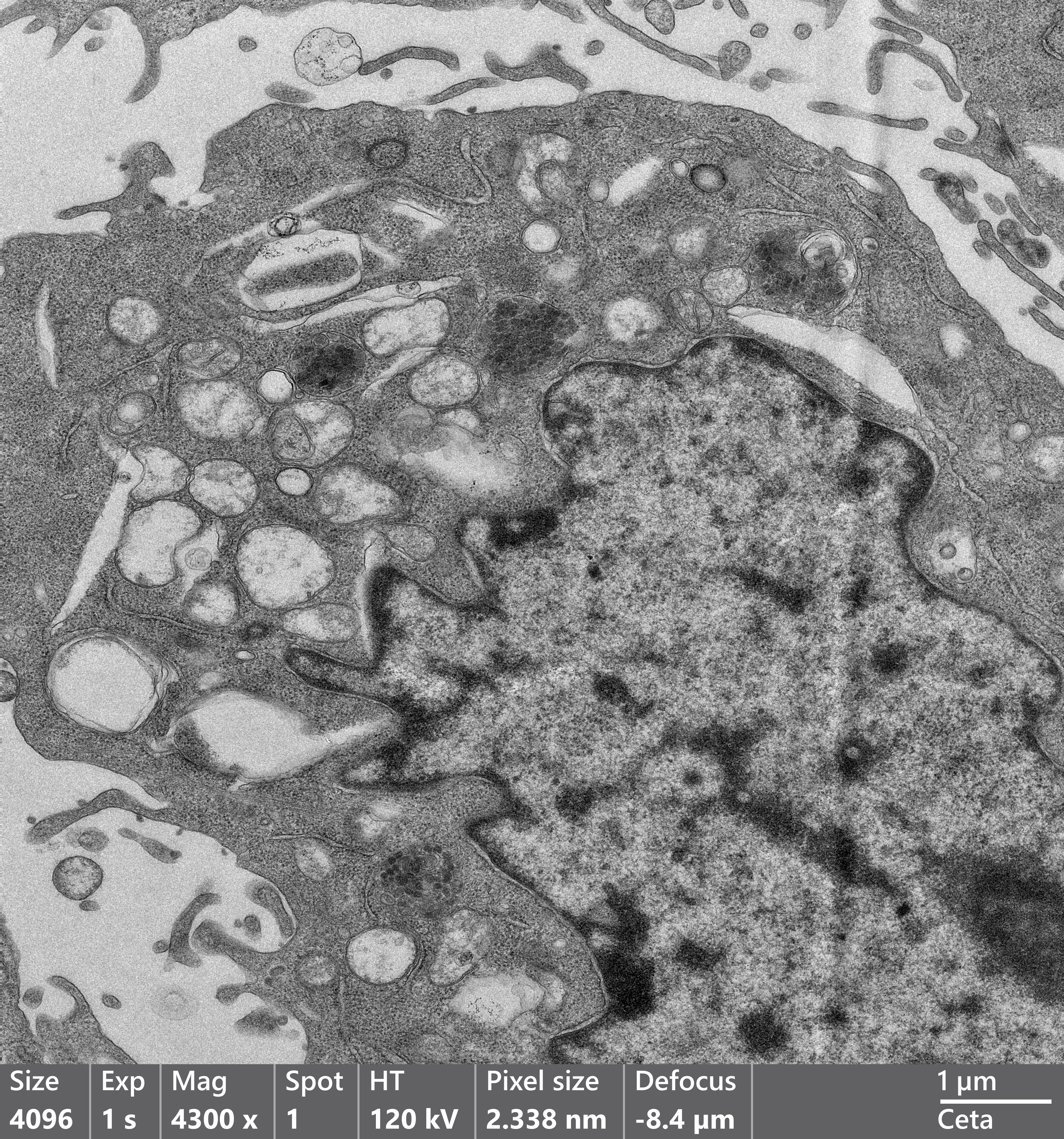

Supplement: Supplementary file 12 — Figure EV2 Source Data [file 44318_2026_816_MOESM12_ESM.zip › A/Figure S2A-MHV-20H.tif]

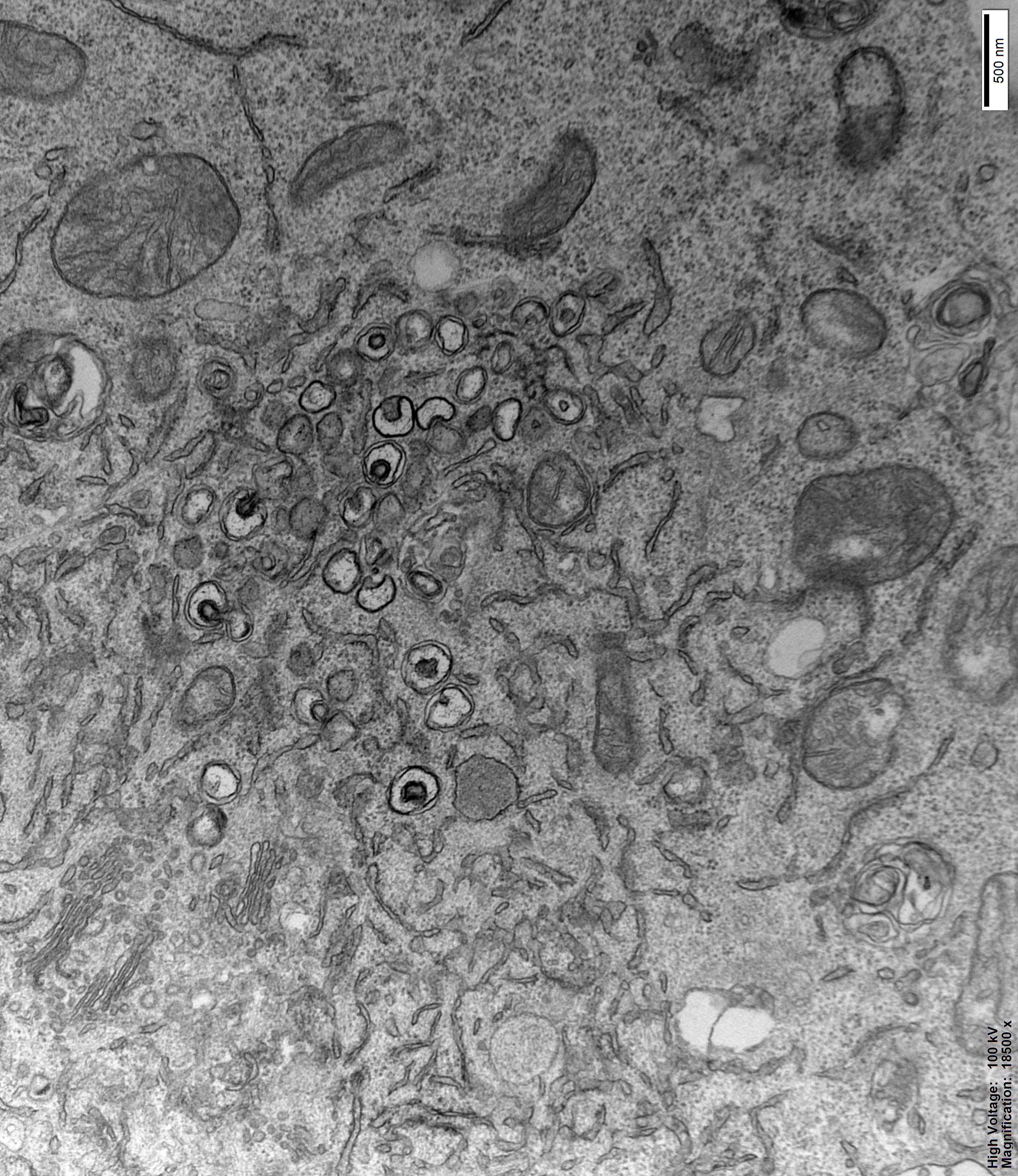

Supplement: Supplementary file 12 — Figure EV2 Source Data [file 44318_2026_816_MOESM12_ESM.zip › A/Figure S2A-MHV-4H-1.tif]

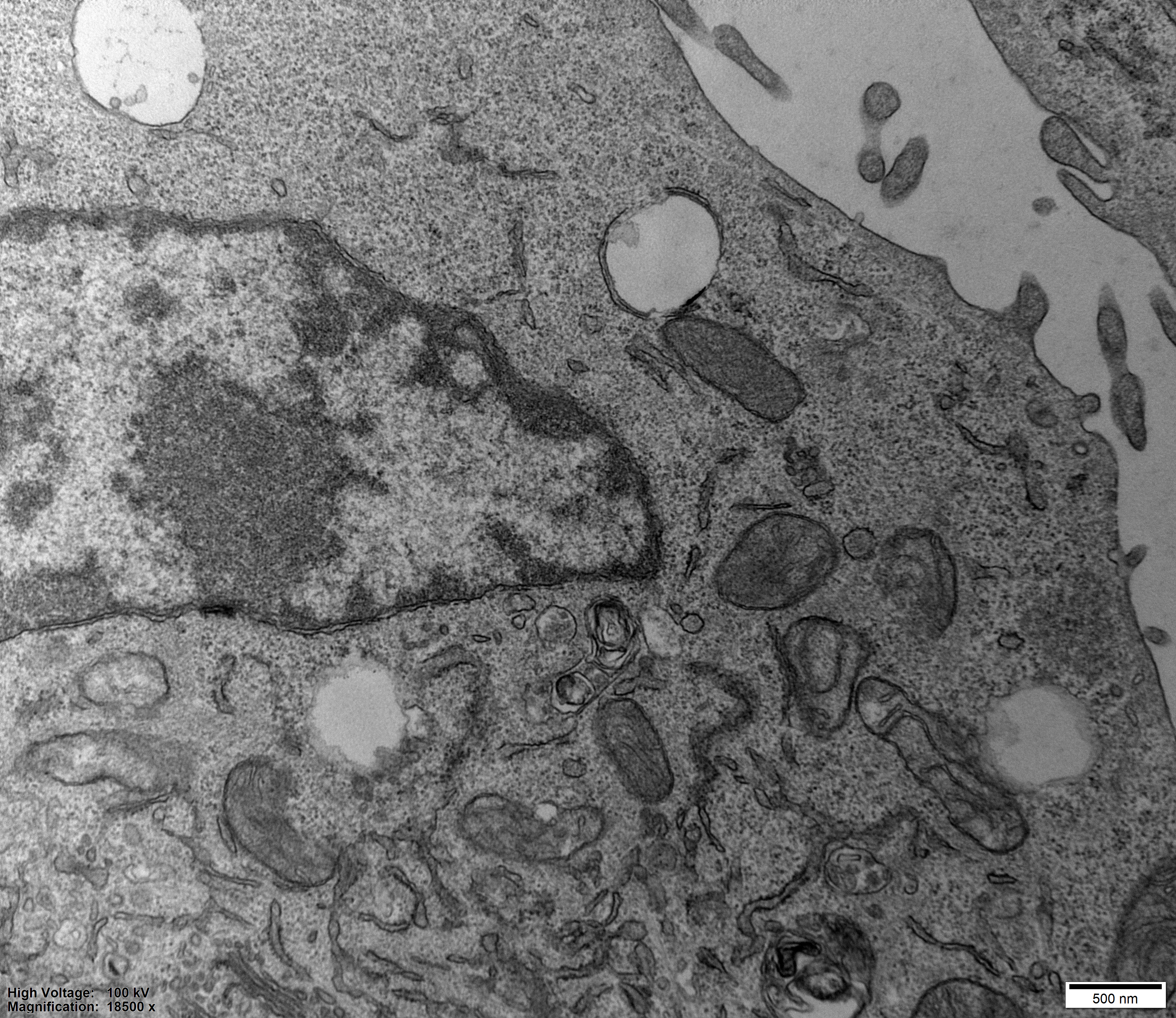

Supplement: Supplementary file 12 — Figure EV2 Source Data [file 44318_2026_816_MOESM12_ESM.zip › A/Figure S2A-MHV-4H-2.tif]

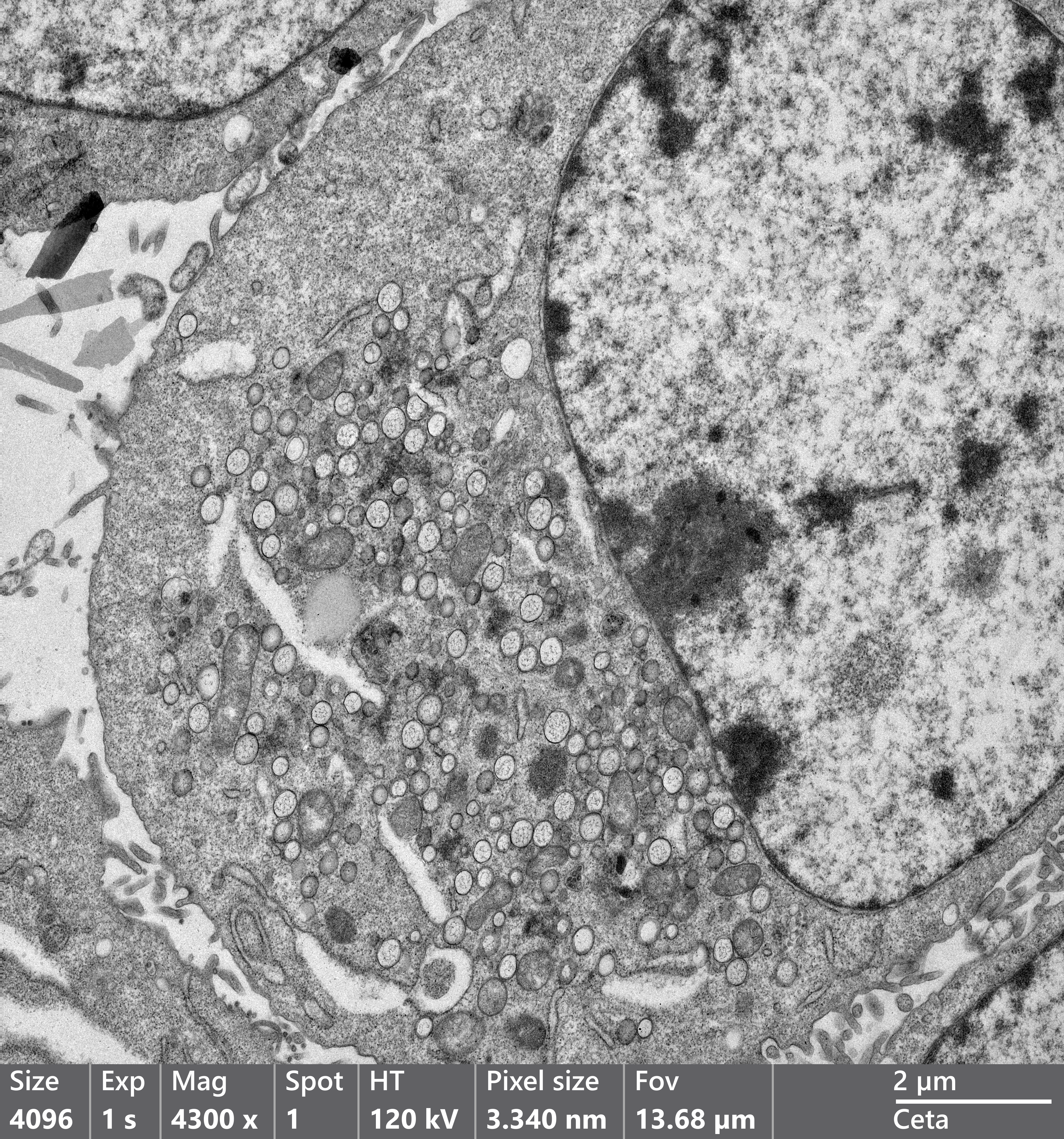

Supplement: Supplementary file 12 — Figure EV2 Source Data [file 44318_2026_816_MOESM12_ESM.zip › A/Figure S2A-MHV-8H.tif]

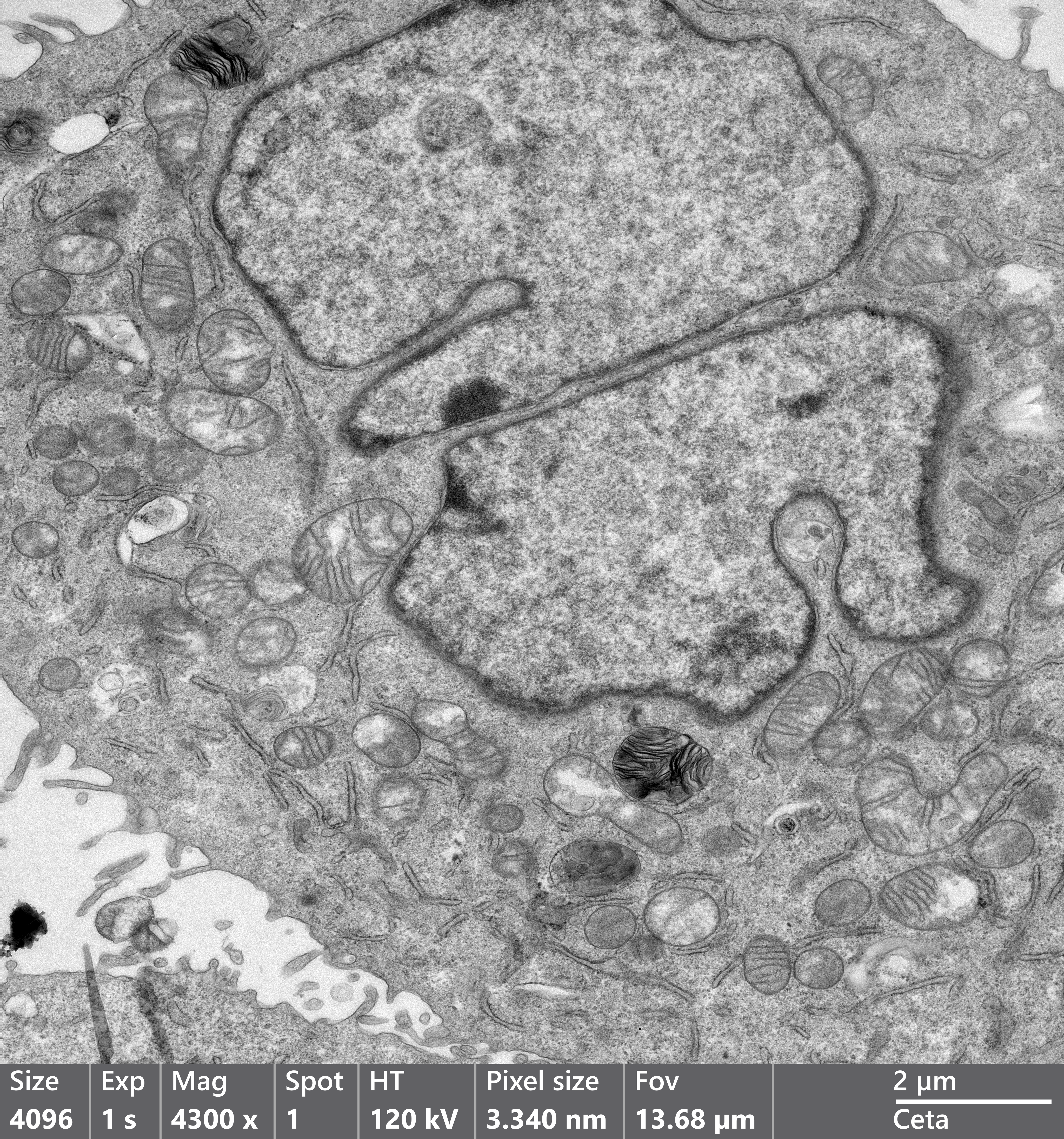

Supplement: Supplementary file 12 — Figure EV2 Source Data [file 44318_2026_816_MOESM12_ESM.zip › E/SARA-CoV-2-0H.tif]

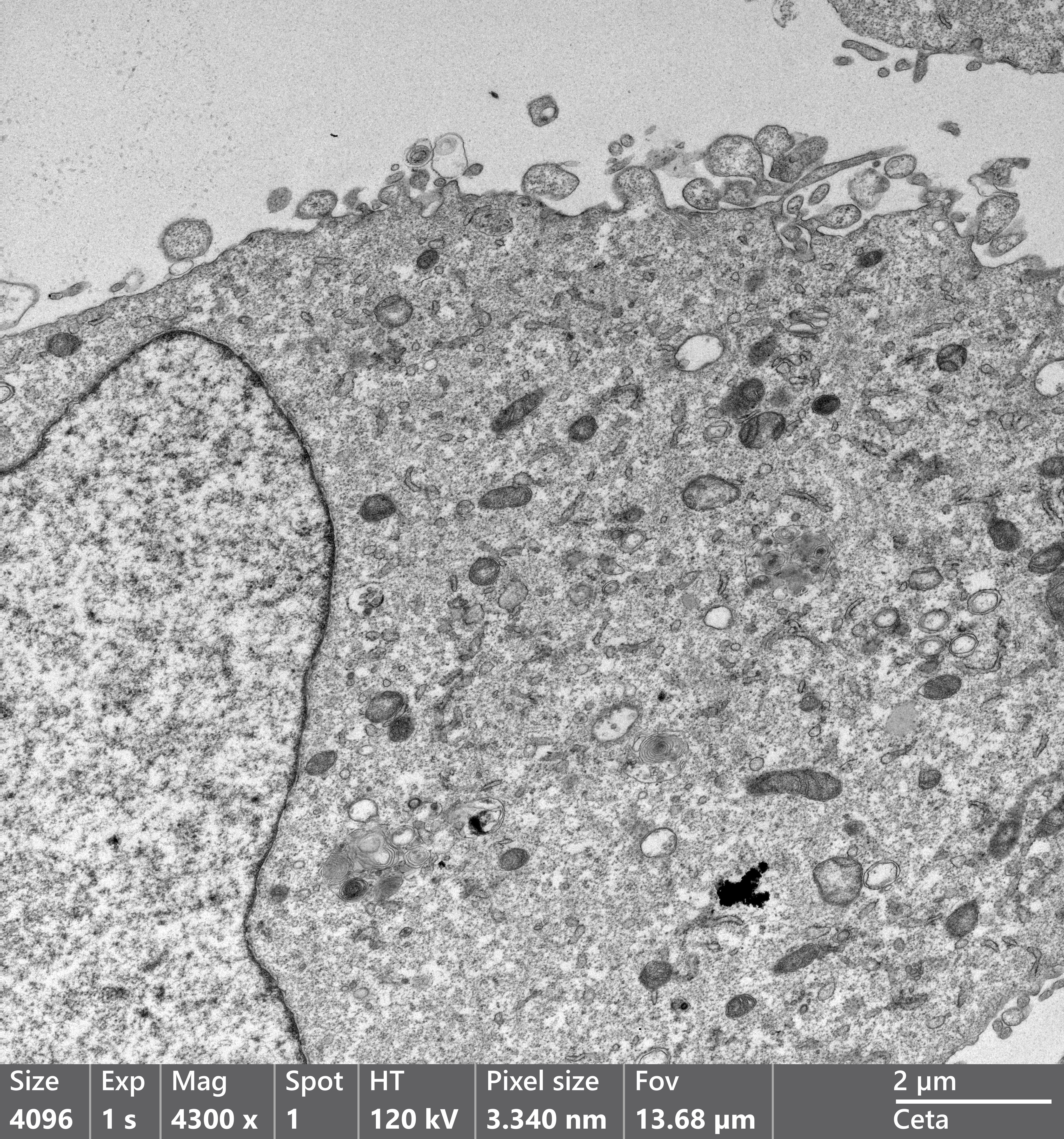

Supplement: Supplementary file 12 — Figure EV2 Source Data [file 44318_2026_816_MOESM12_ESM.zip › E/SARA-CoV-2-24H.tif]

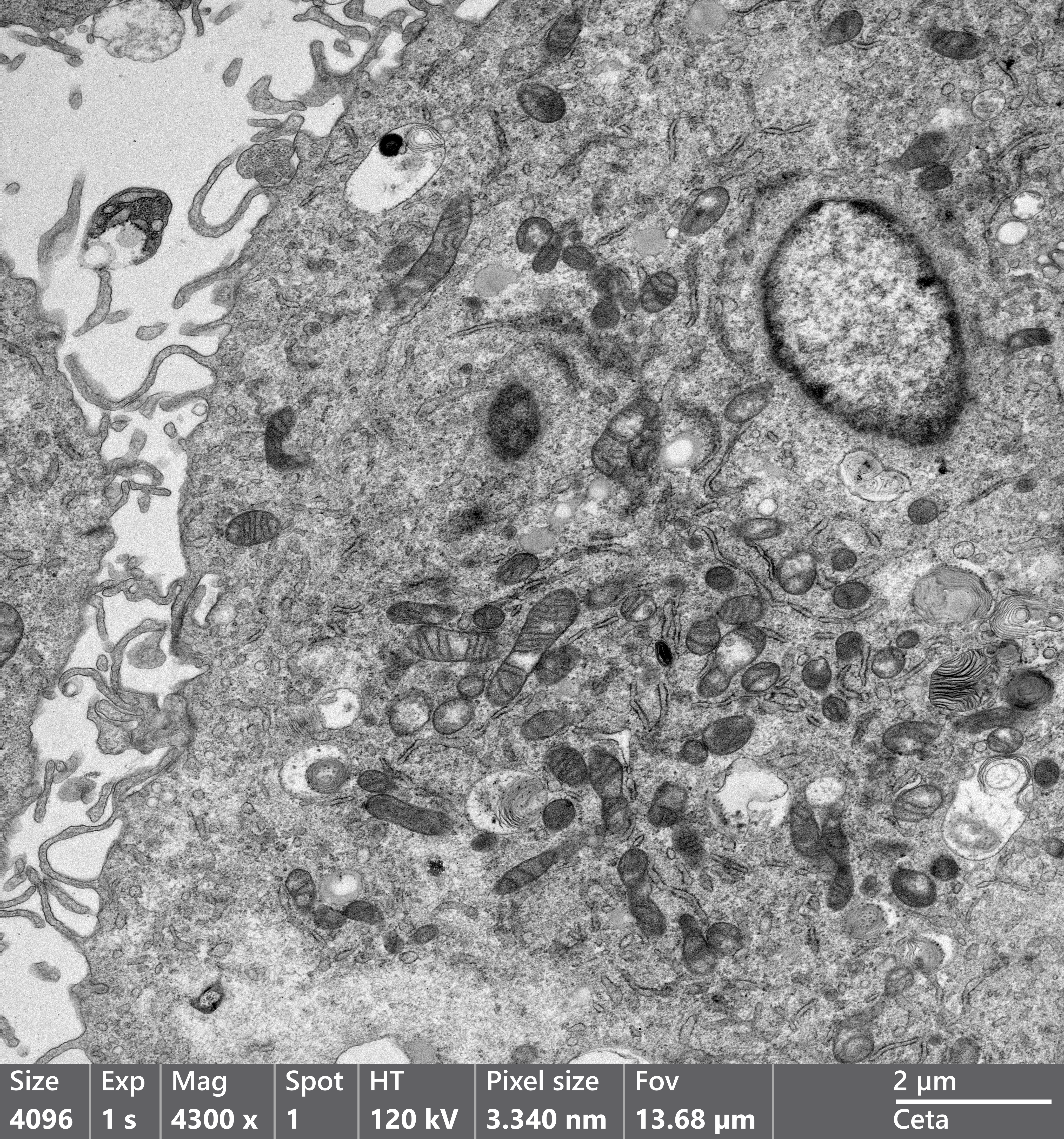

Supplement: Supplementary file 12 — Figure EV2 Source Data [file 44318_2026_816_MOESM12_ESM.zip › E/SARA-CoV-2-2H.tif]

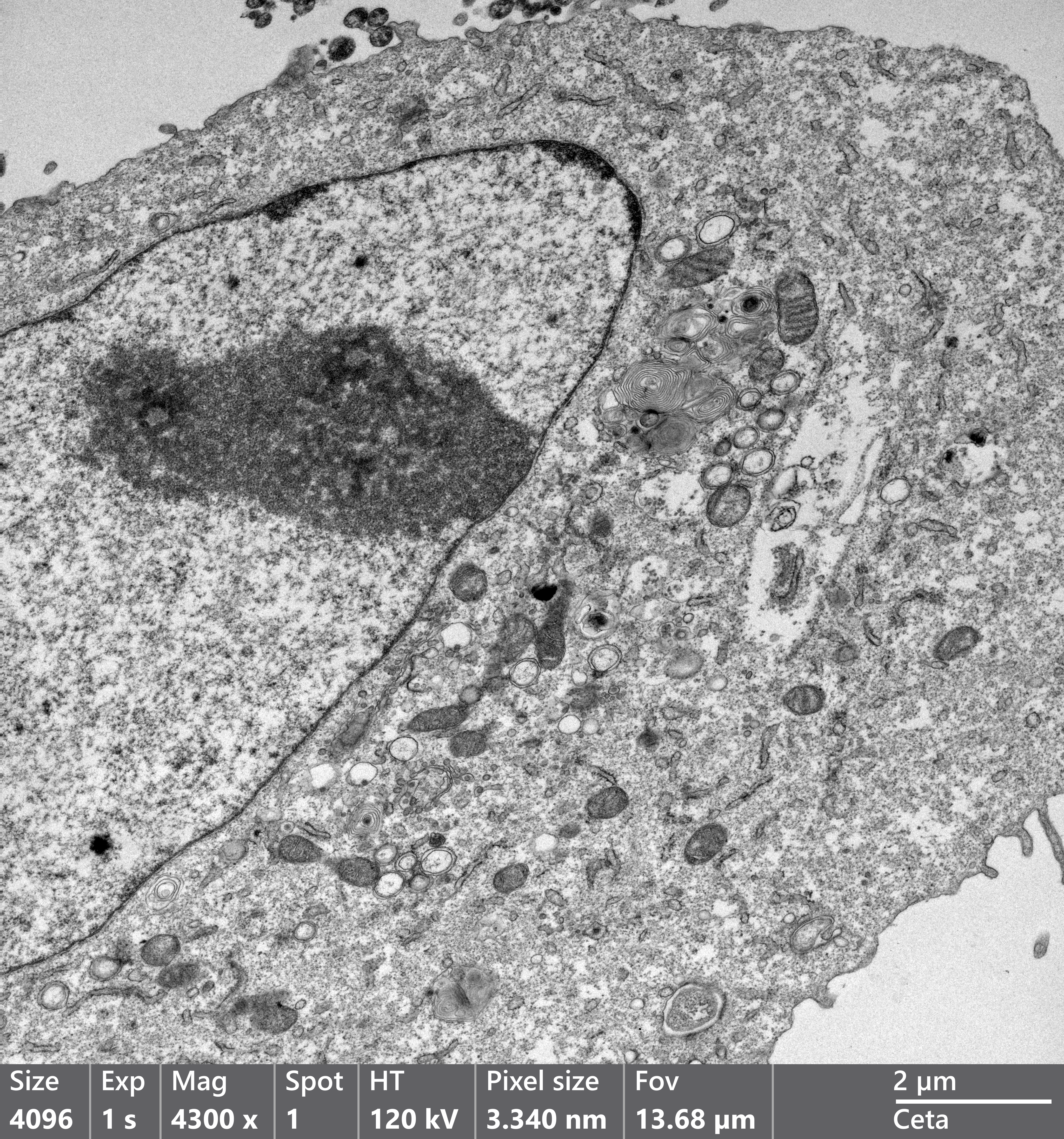

Supplement: Supplementary file 12 — Figure EV2 Source Data [file 44318_2026_816_MOESM12_ESM.zip › E/SARA-CoV-2-6H.tif]

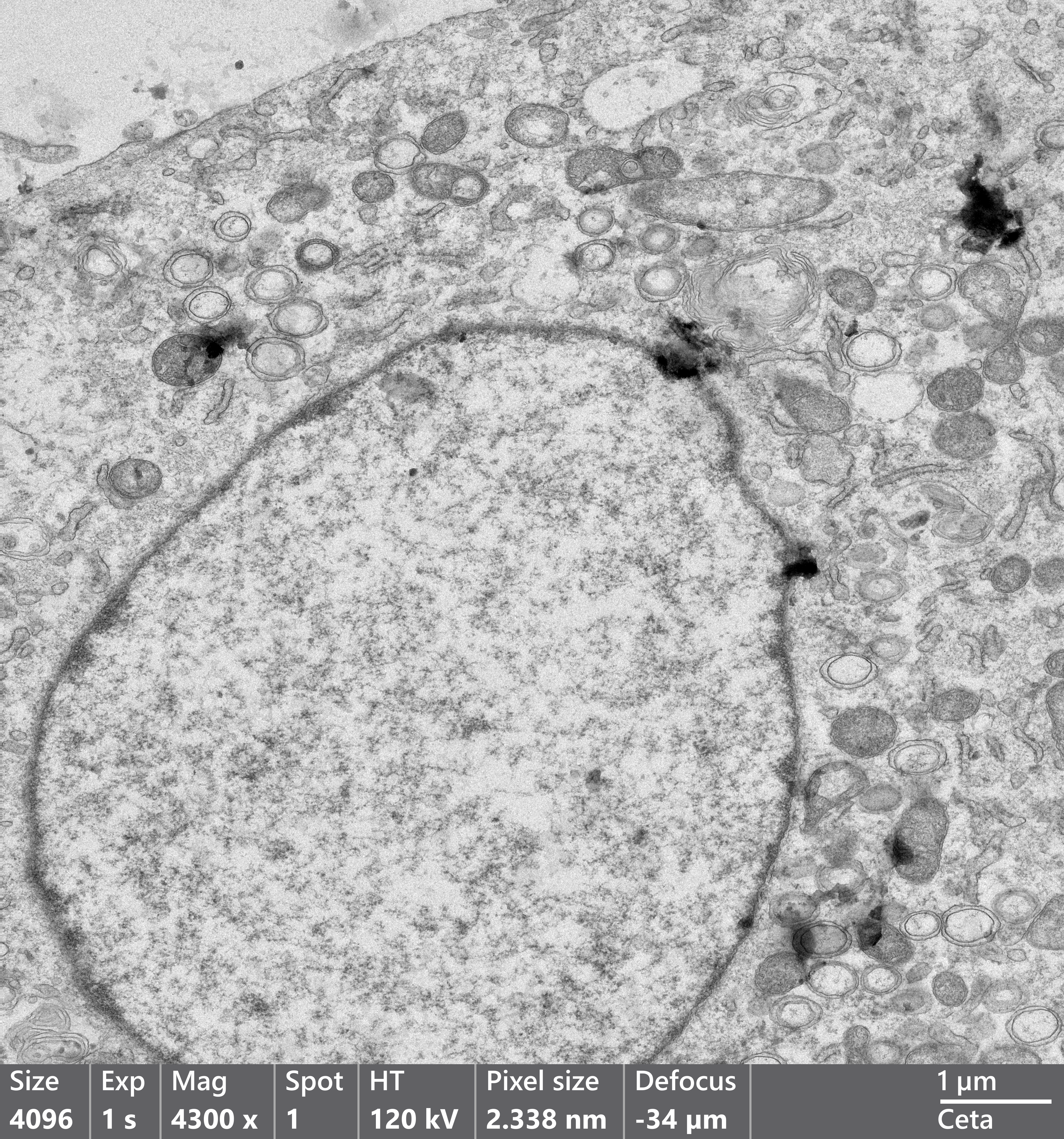

Supplement: Supplementary file 12 — Figure EV2 Source Data [file 44318_2026_816_MOESM12_ESM.zip › E/SARA-CoV-2-8H.tif]

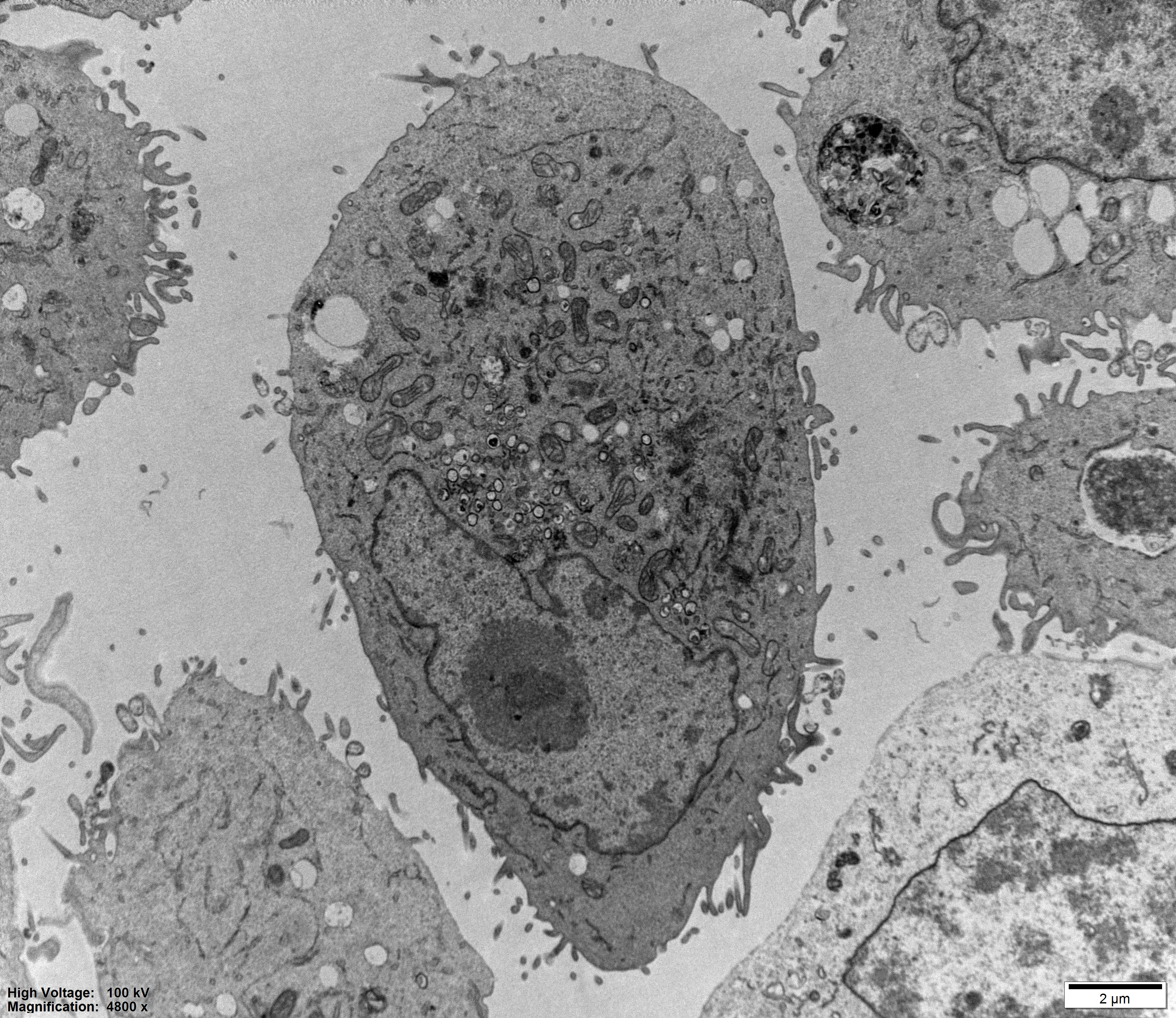

Supplement: Supplementary file 13 — Figure EV3 Source Data [file 44318_2026_816_MOESM13_ESM.zip › A/Figure S3A-MHV-4H .tif]

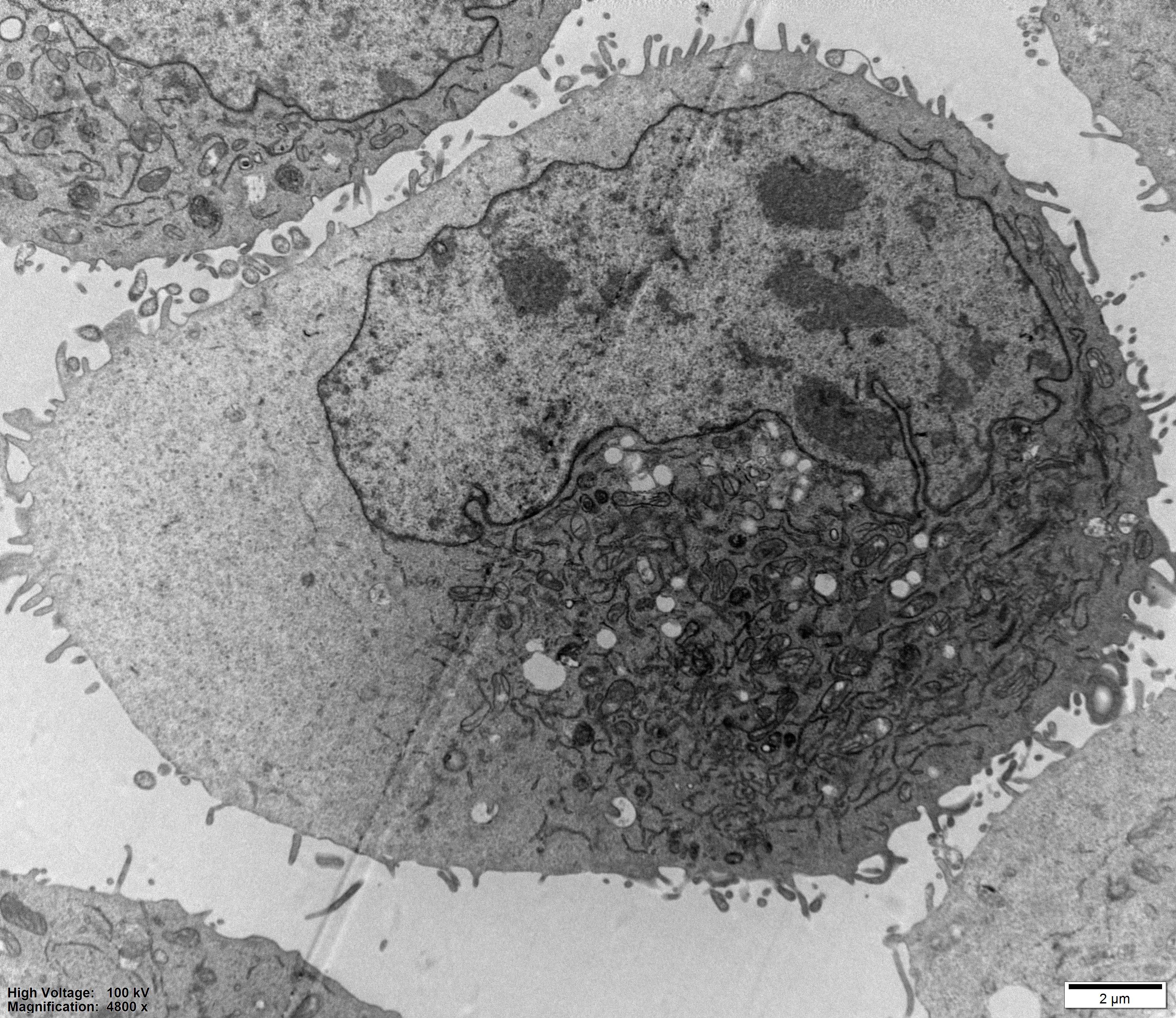

Supplement: Supplementary file 13 — Figure EV3 Source Data [file 44318_2026_816_MOESM13_ESM.zip › A/Figure S3A-MHV-4H RDV.tif]

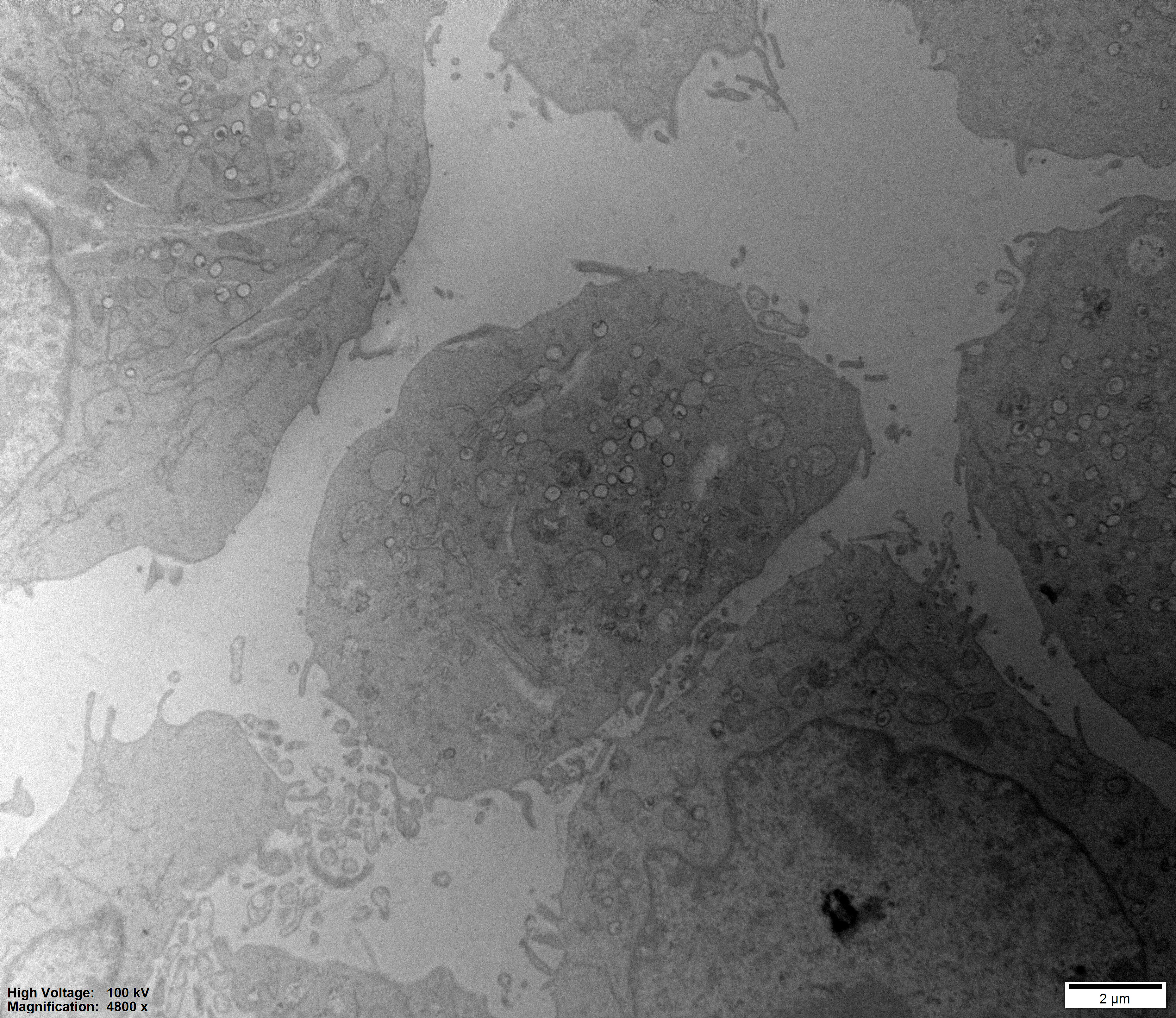

Supplement: Supplementary file 13 — Figure EV3 Source Data [file 44318_2026_816_MOESM13_ESM.zip › D/Figure S3D MHV-8H .tif]

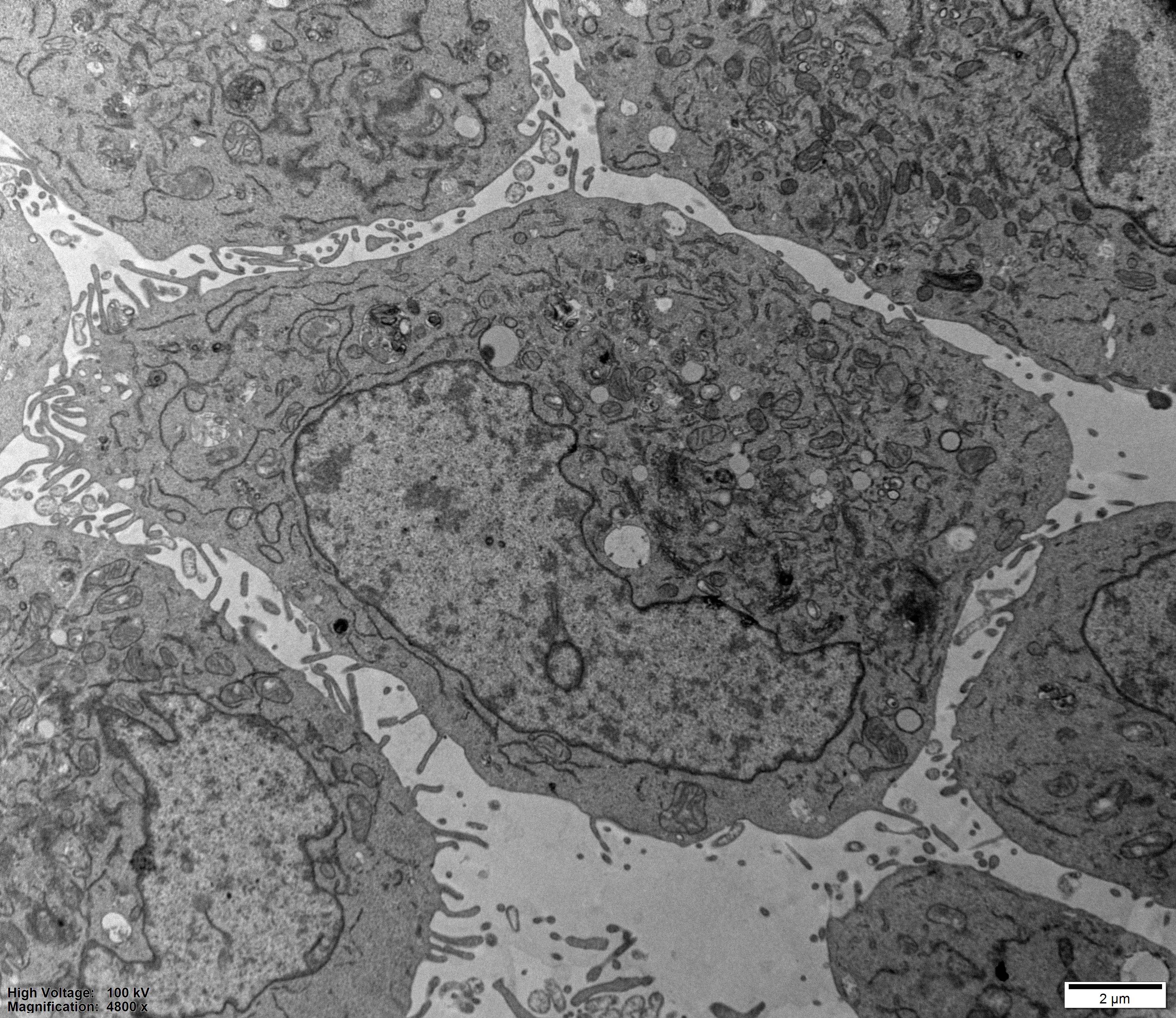

Supplement: Supplementary file 13 — Figure EV3 Source Data [file 44318_2026_816_MOESM13_ESM.zip › D/Figure S3D-MHV-8H RDV.tif]

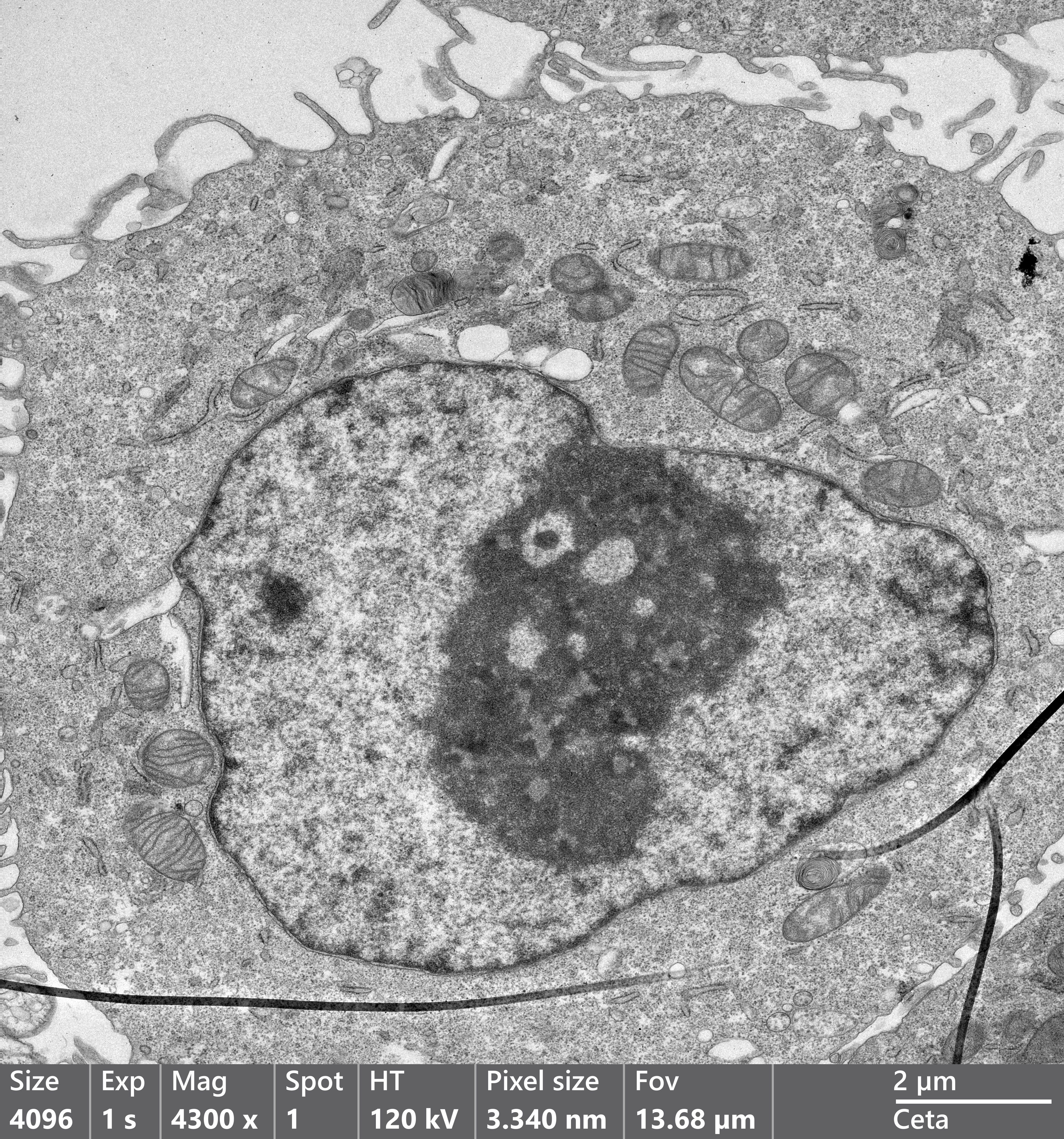

Supplement: Supplementary file 13 — Figure EV3 Source Data [file 44318_2026_816_MOESM13_ESM.zip › G/Figure S3G-Ctrl.tif]

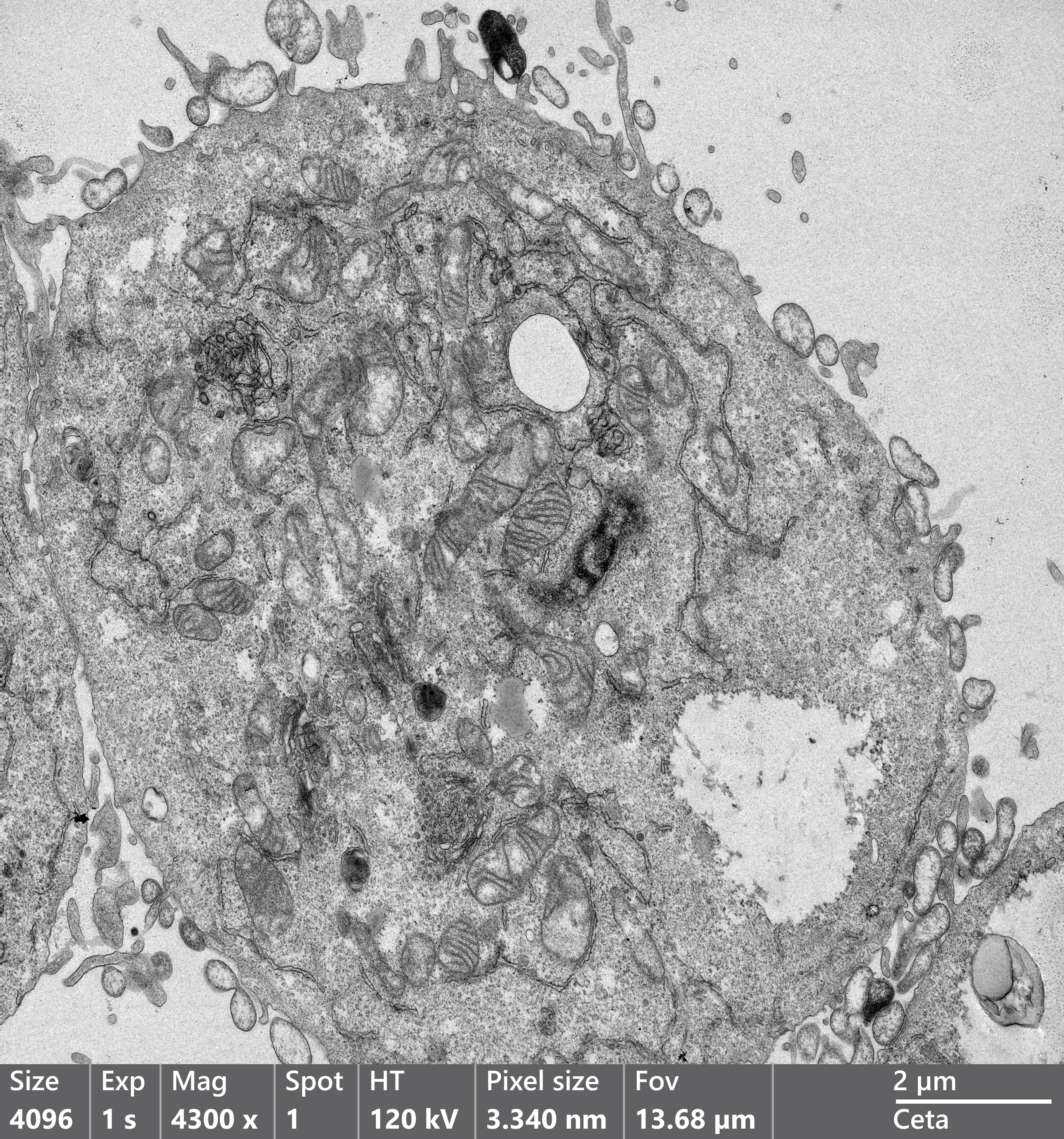

Supplement: Supplementary file 13 — Figure EV3 Source Data [file 44318_2026_816_MOESM13_ESM.zip › G/Figure S3G-NSP3+NSP4 H130N F121L.tif]

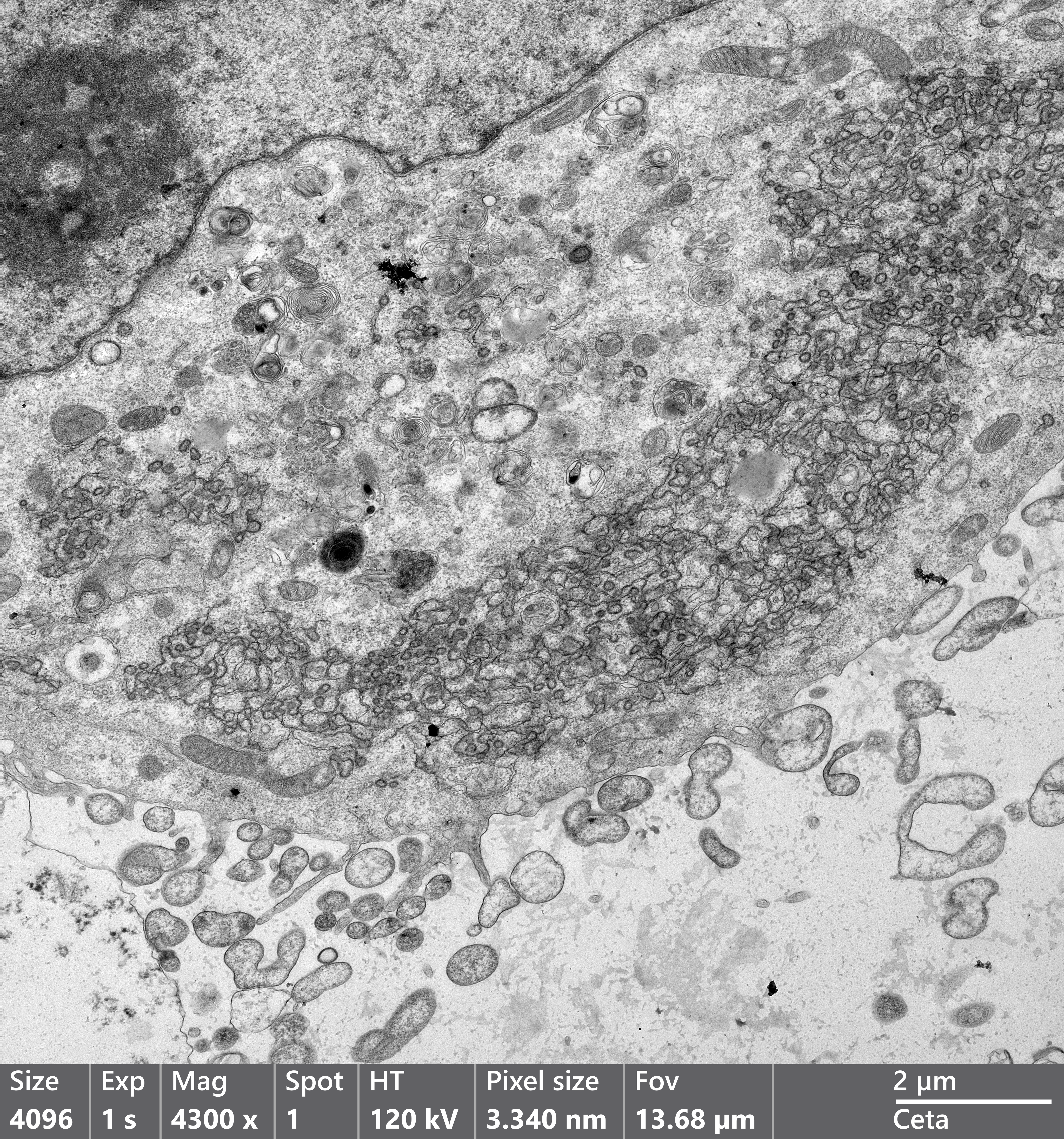

Supplement: Supplementary file 13 — Figure EV3 Source Data [file 44318_2026_816_MOESM13_ESM.zip › G/Figure S3G-NSP3+NSP4 WT.tif]

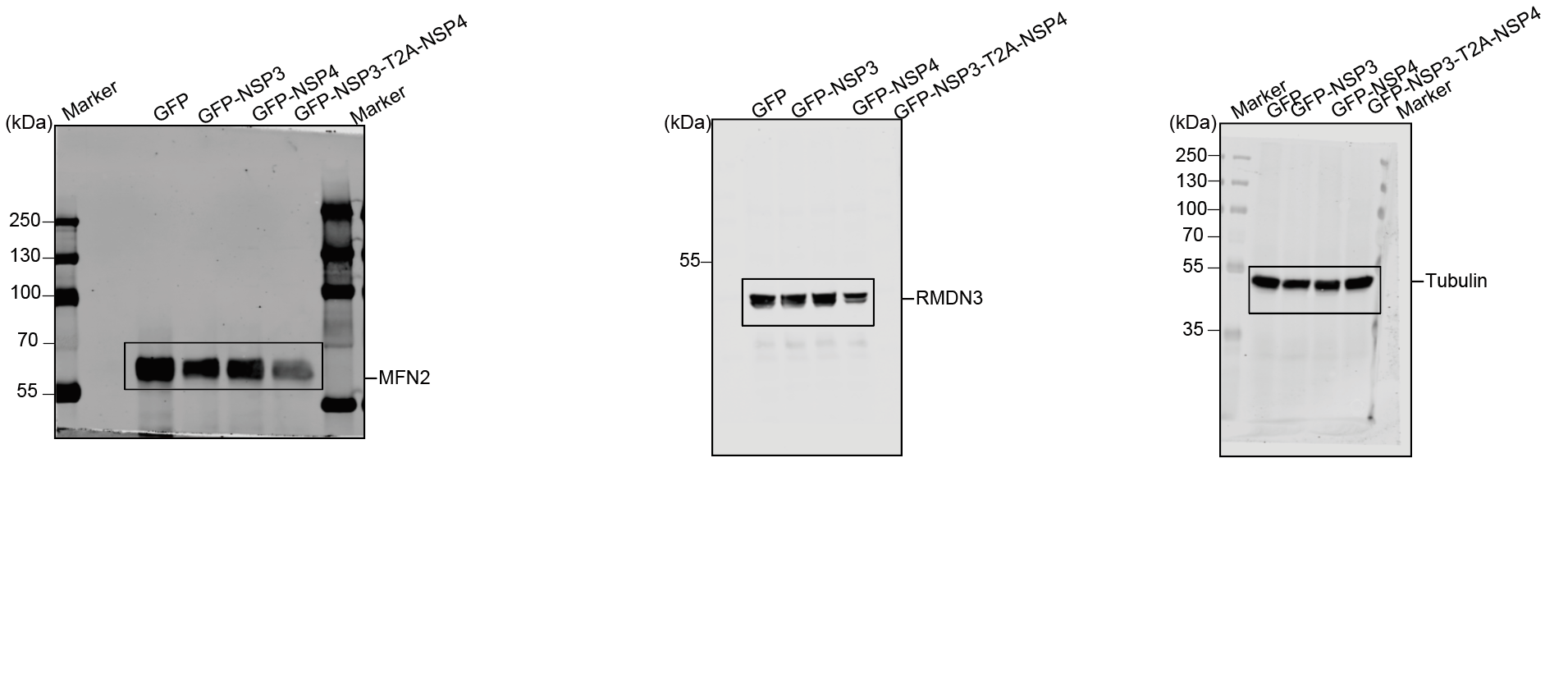

Supplement: Supplementary file 14 — Figure EV4 Source Data [file 44318_2026_816_MOESM14_ESM.zip › B/MFN2+RMDN3+Tubulin.tif]

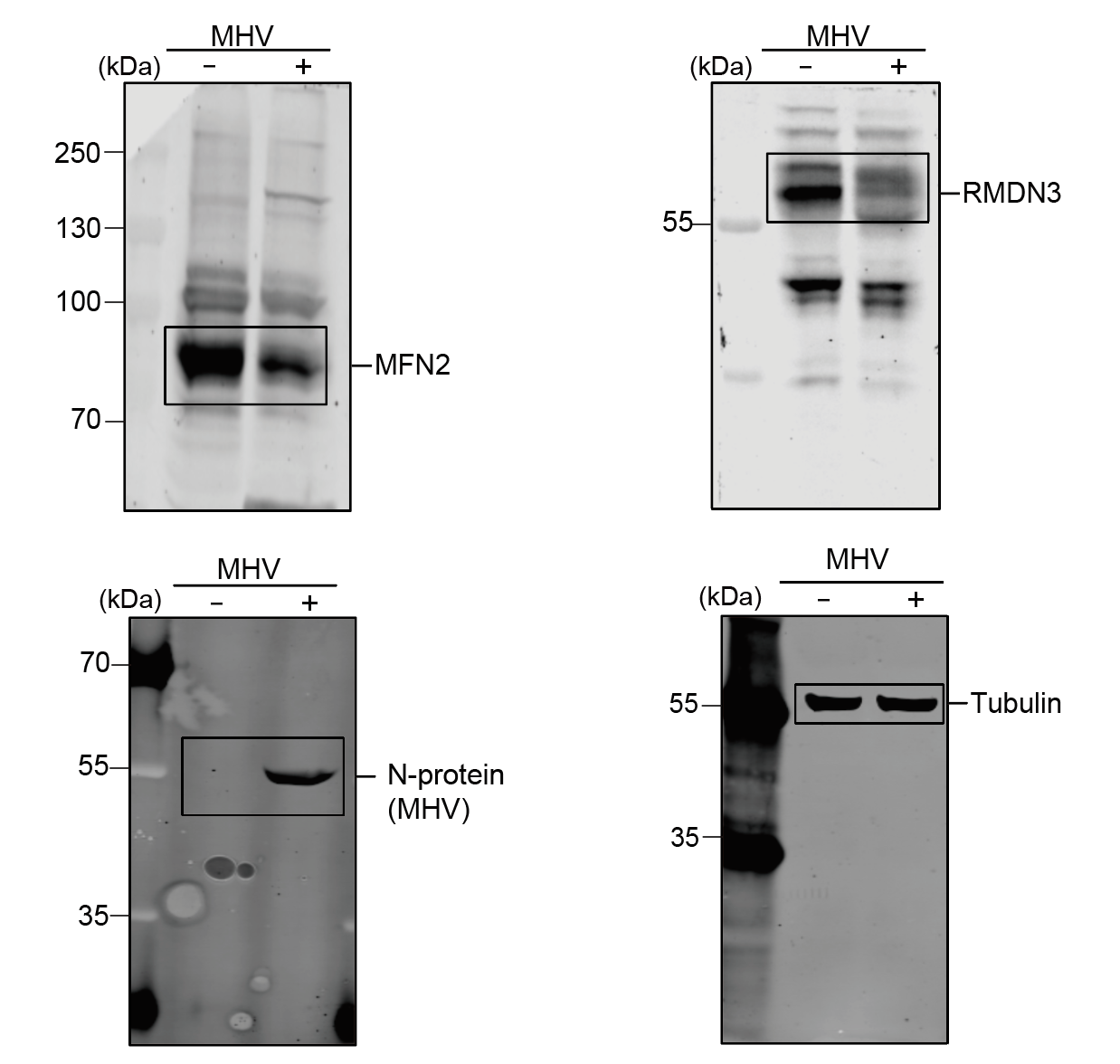

Supplement: Supplementary file 14 — Figure EV4 Source Data [file 44318_2026_816_MOESM14_ESM.zip › C/MFN2+RMDN3+Nprotein+Tubulin.tif]
