# Supplementary figures and images for: Disruption of ER-mitochondria contact sites by coronavirus replication organelles sustains viral replication via NSP3 stabilization (part 2 of 2)
Source: EMBO J. 2026 May 28;45(13):4379–416. doi: 10.1038/s44318-026-00816-x (PMC13323368; doi:10.1038/s44318-026-00816-x)

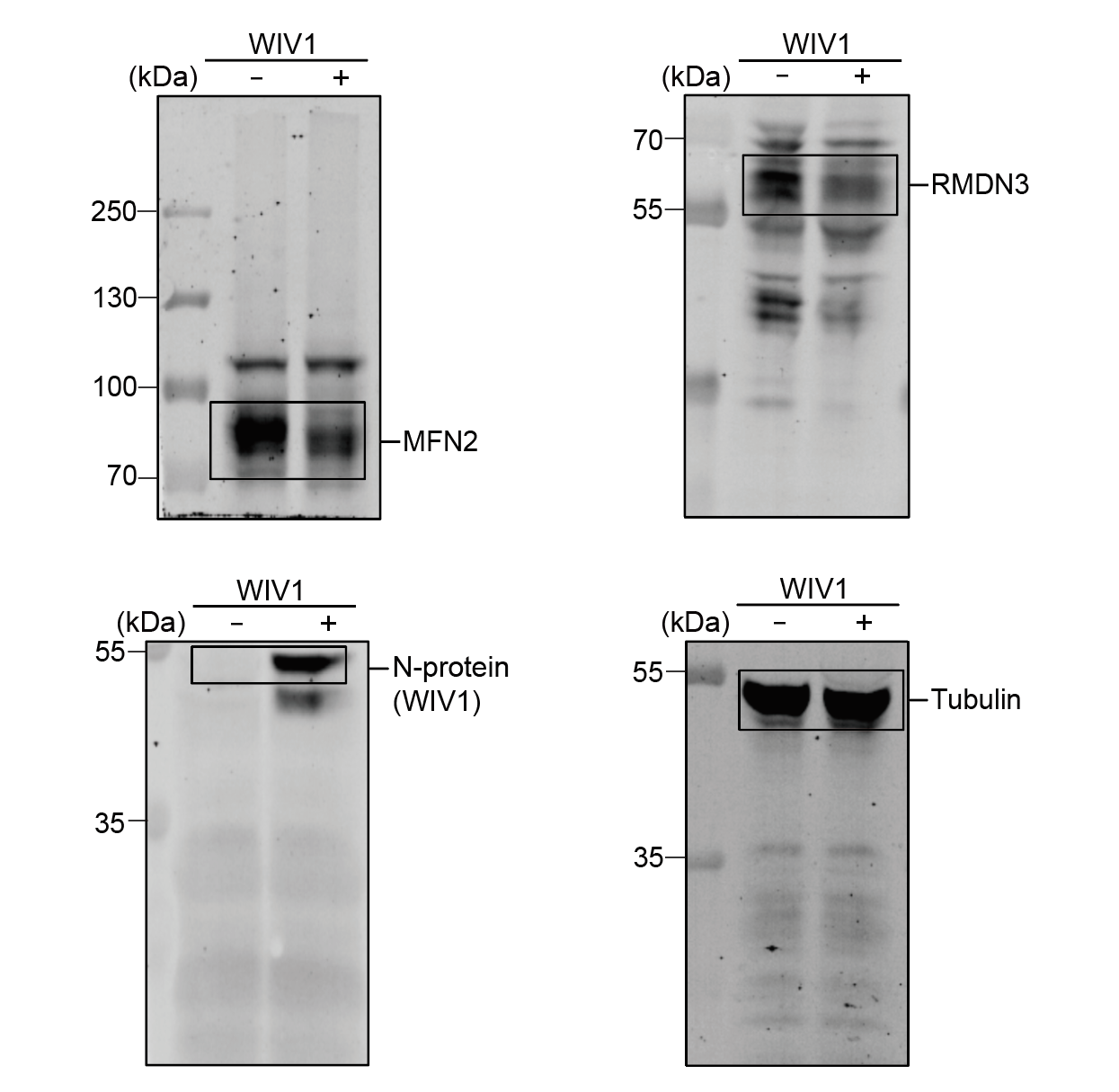

Supplement: Supplementary file 14 — Figure EV4 Source Data [file 44318_2026_816_MOESM14_ESM.zip › D/MFN2+RMDN3+Nprotein+Tubulin.tif]

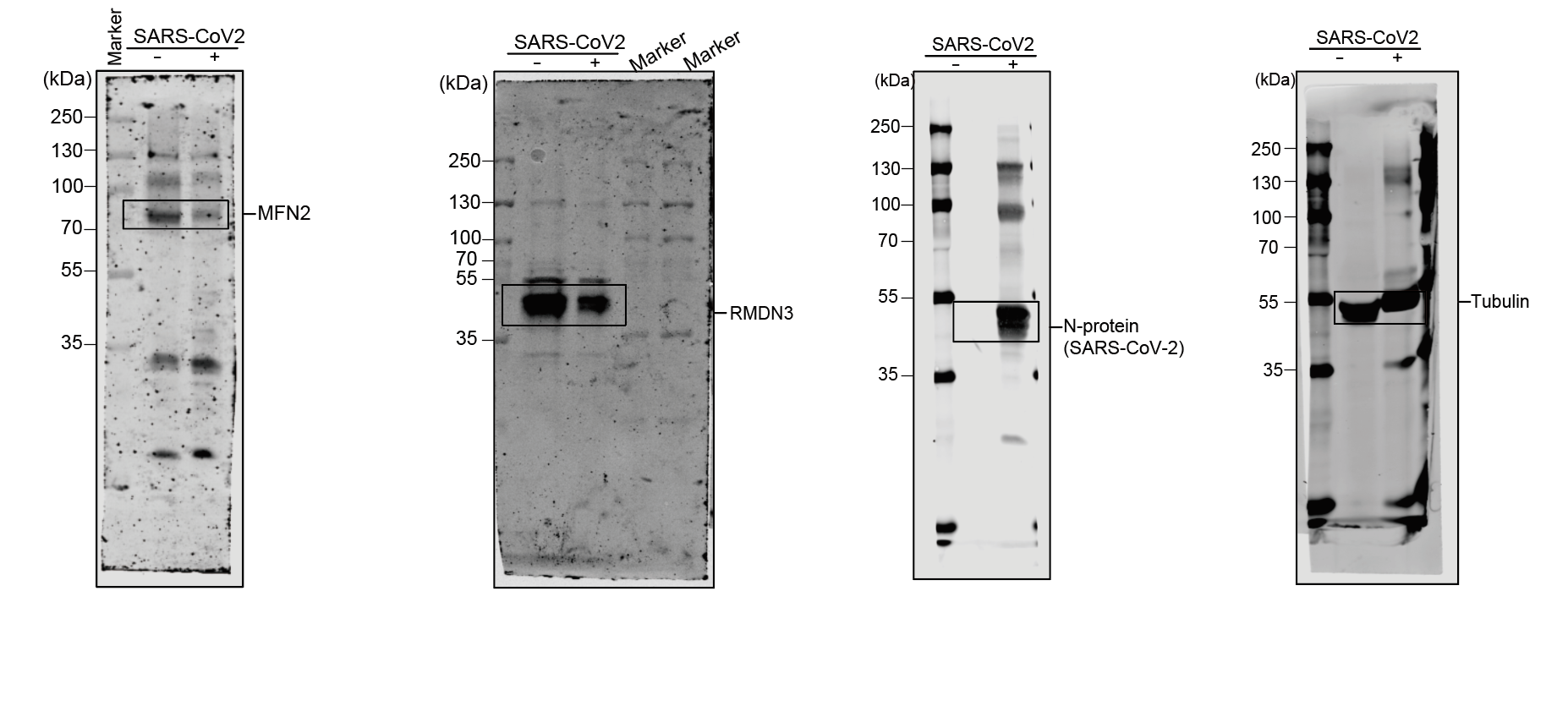

Supplement: Supplementary file 14 — Figure EV4 Source Data [file 44318_2026_816_MOESM14_ESM.zip › E/MFN2+RMDN3+Nprotein+Tubulin.tif]

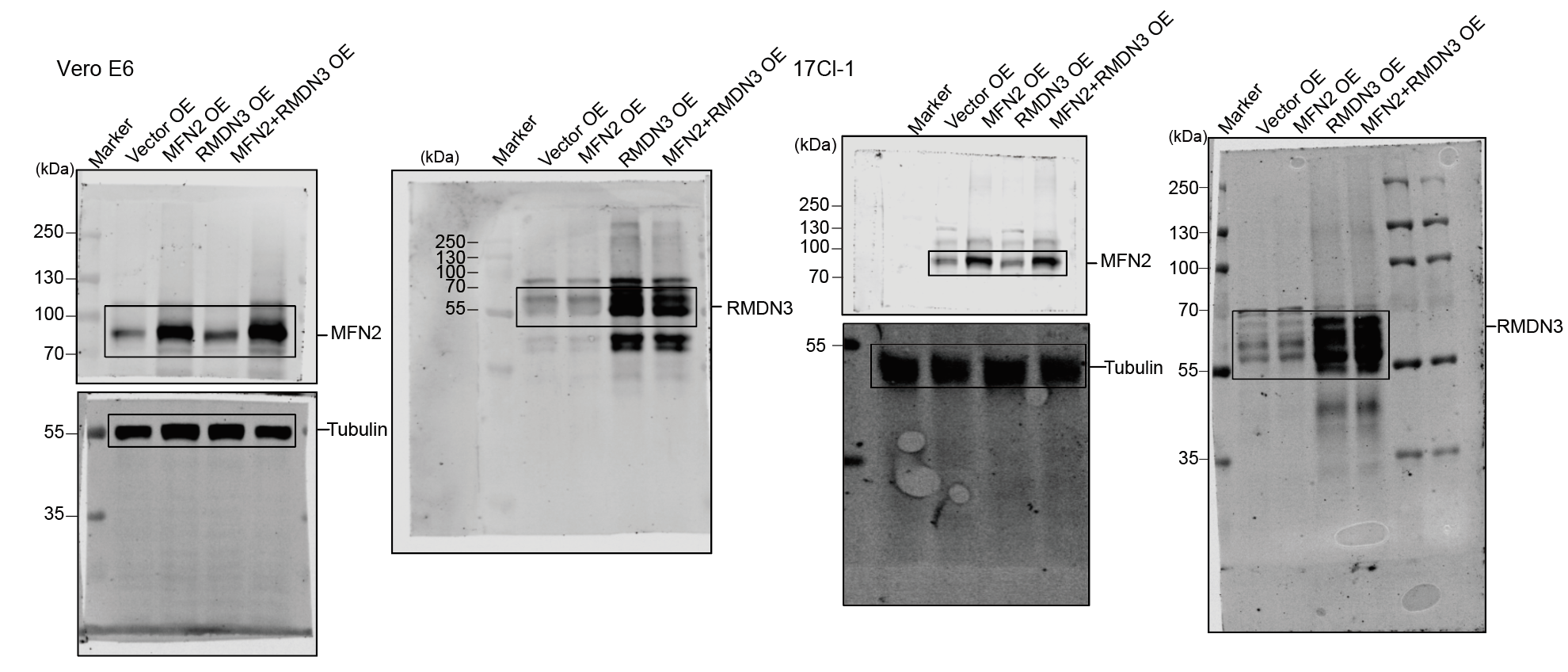

Supplement: Supplementary file 14 — Figure EV4 Source Data [file 44318_2026_816_MOESM14_ESM.zip › F/MF2+RMDN3+Tubulin.tif]

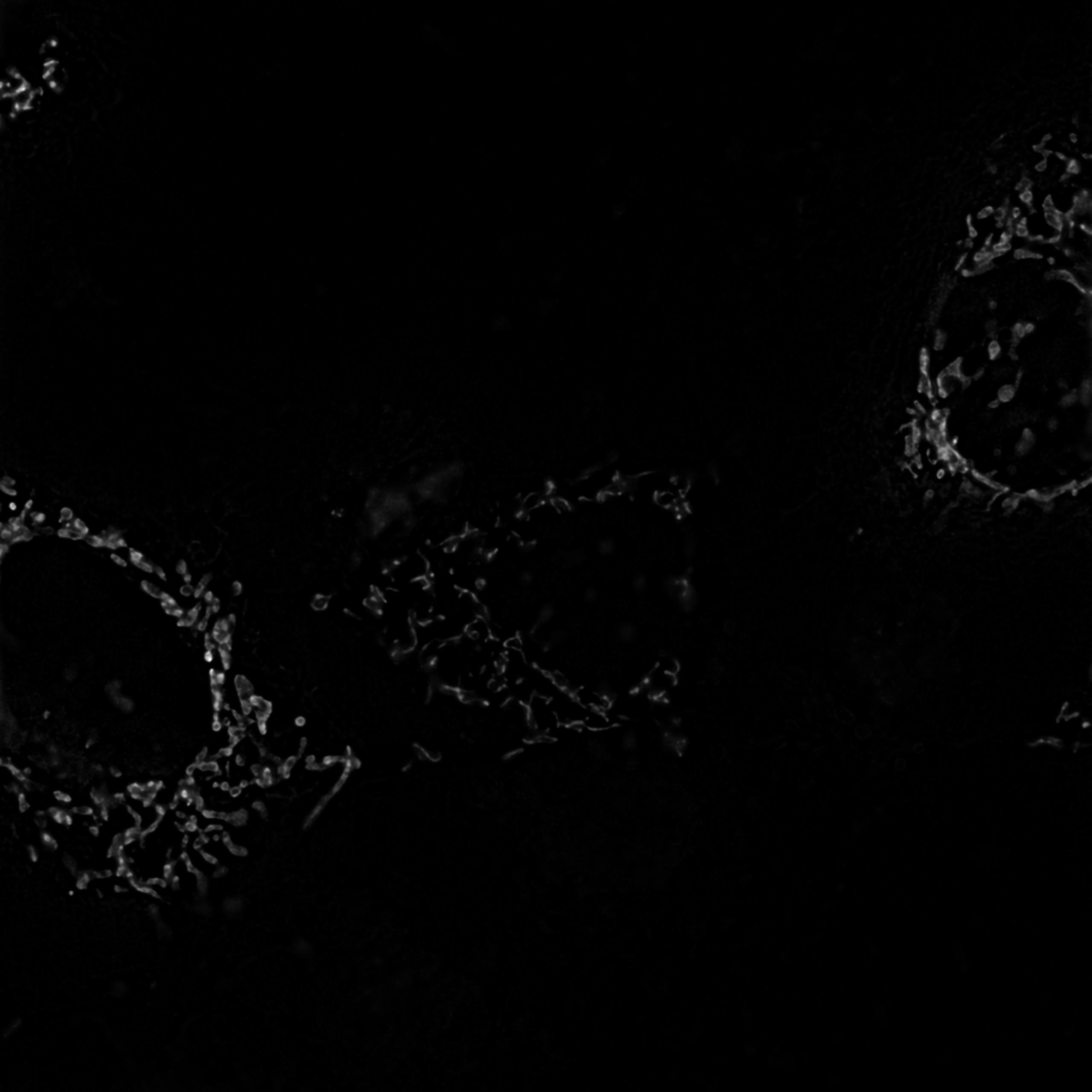

Supplement: Supplementary file 14 — Figure EV4 Source Data [file 44318_2026_816_MOESM14_ESM.zip › G/+ OMM-ER Linker.tif]

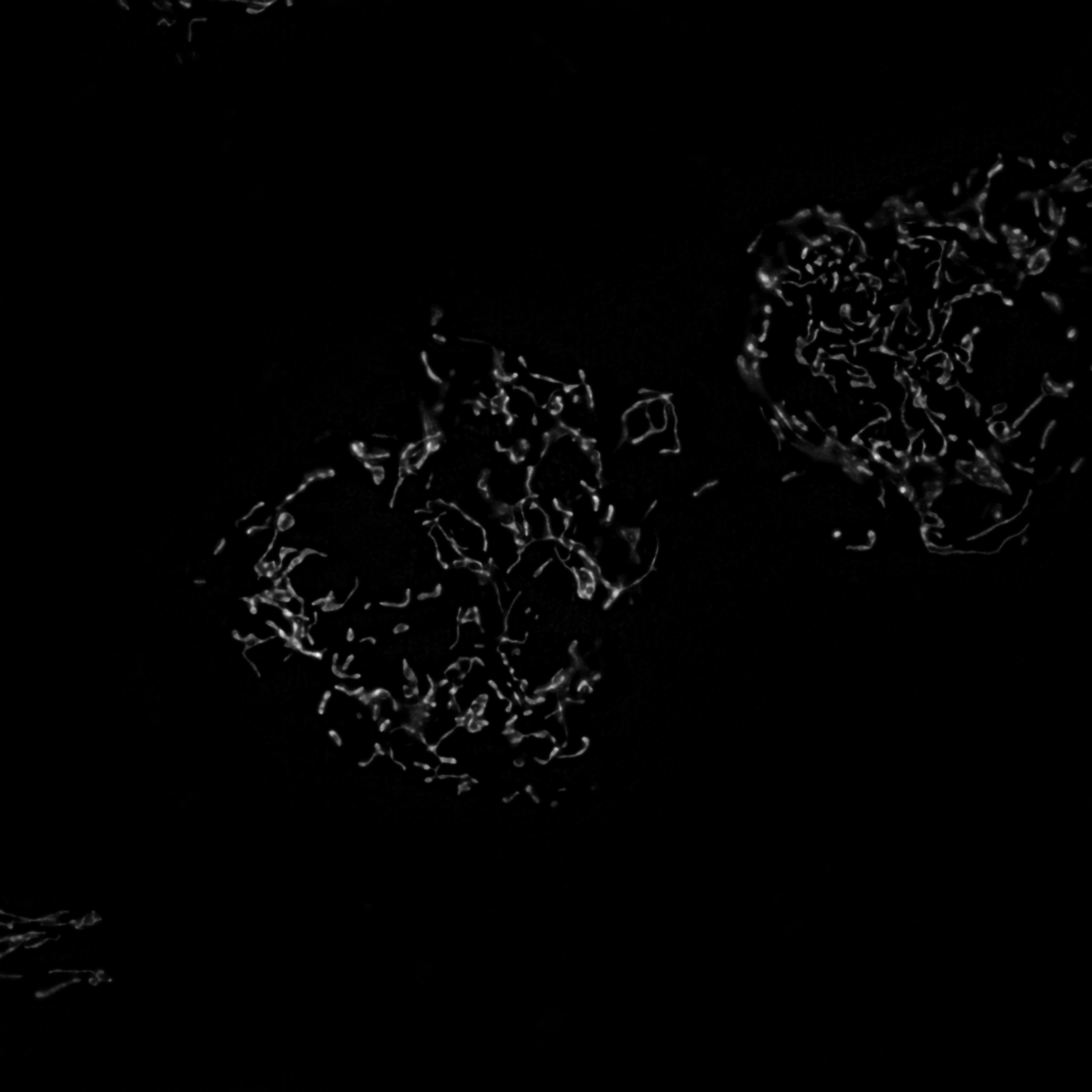

Supplement: Supplementary file 14 — Figure EV4 Source Data [file 44318_2026_816_MOESM14_ESM.zip › G/+B-A l.tif]

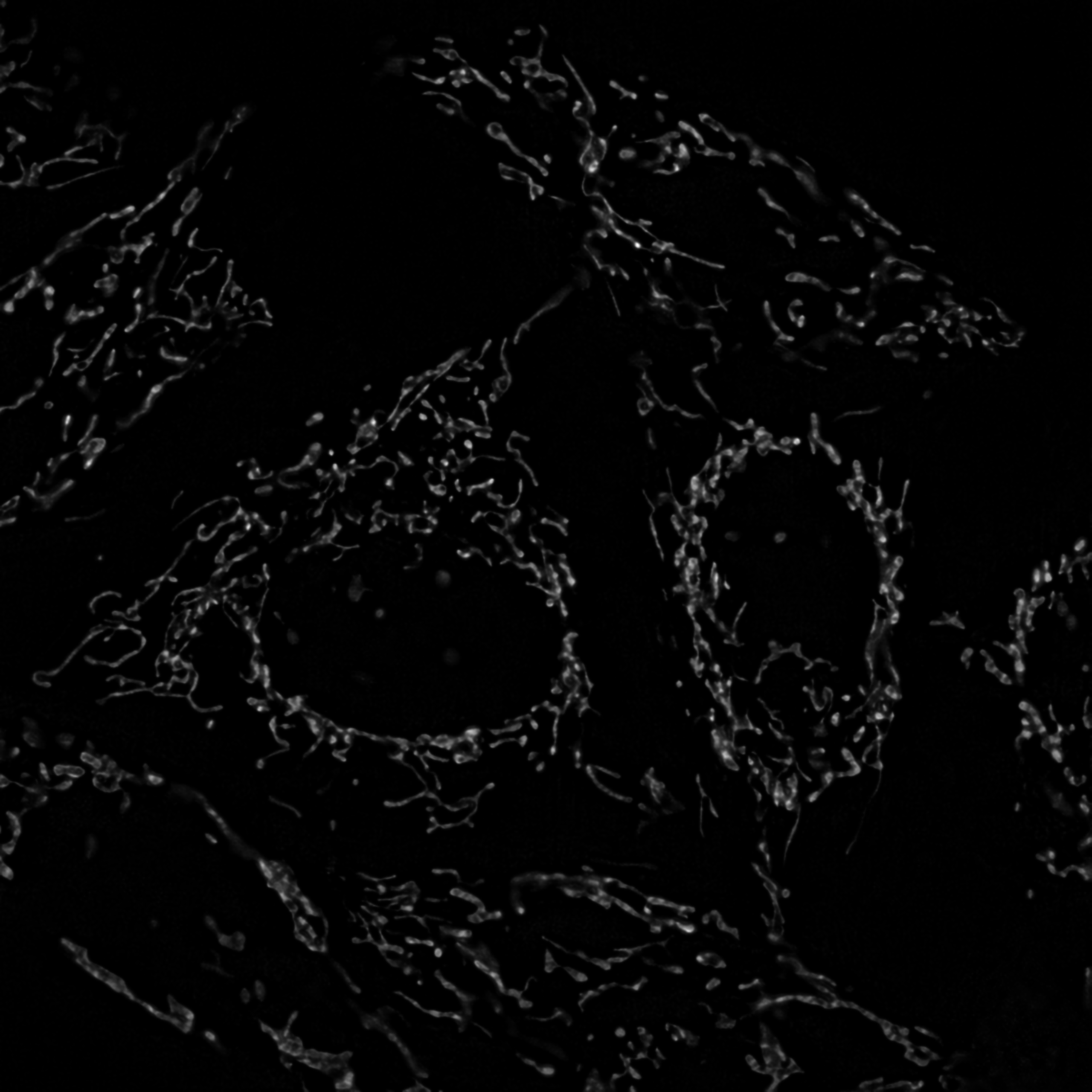

Supplement: Supplementary file 14 — Figure EV4 Source Data [file 44318_2026_816_MOESM14_ESM.zip › G/Ctrl.tif]

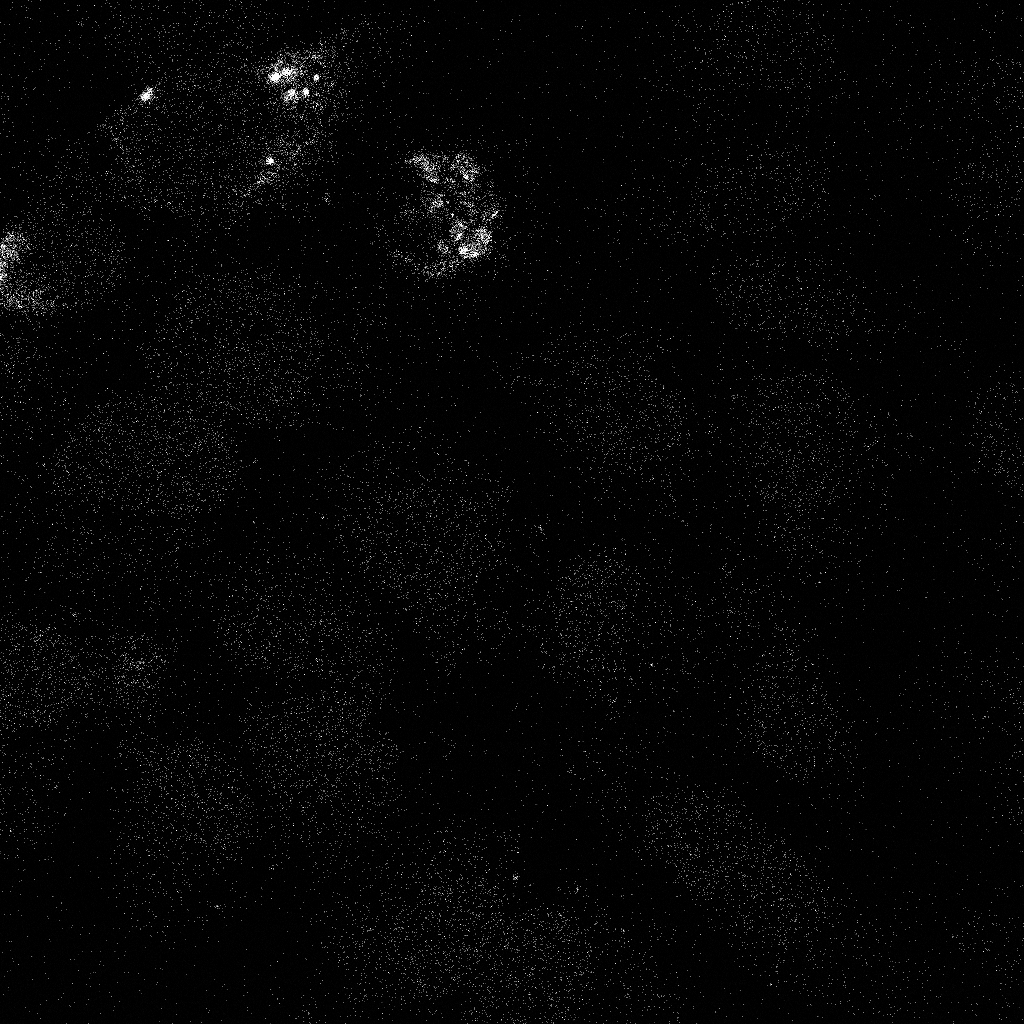

Supplement: Supplementary file 14 — Figure EV4 Source Data [file 44318_2026_816_MOESM14_ESM.zip › H/+B-A l.tif]

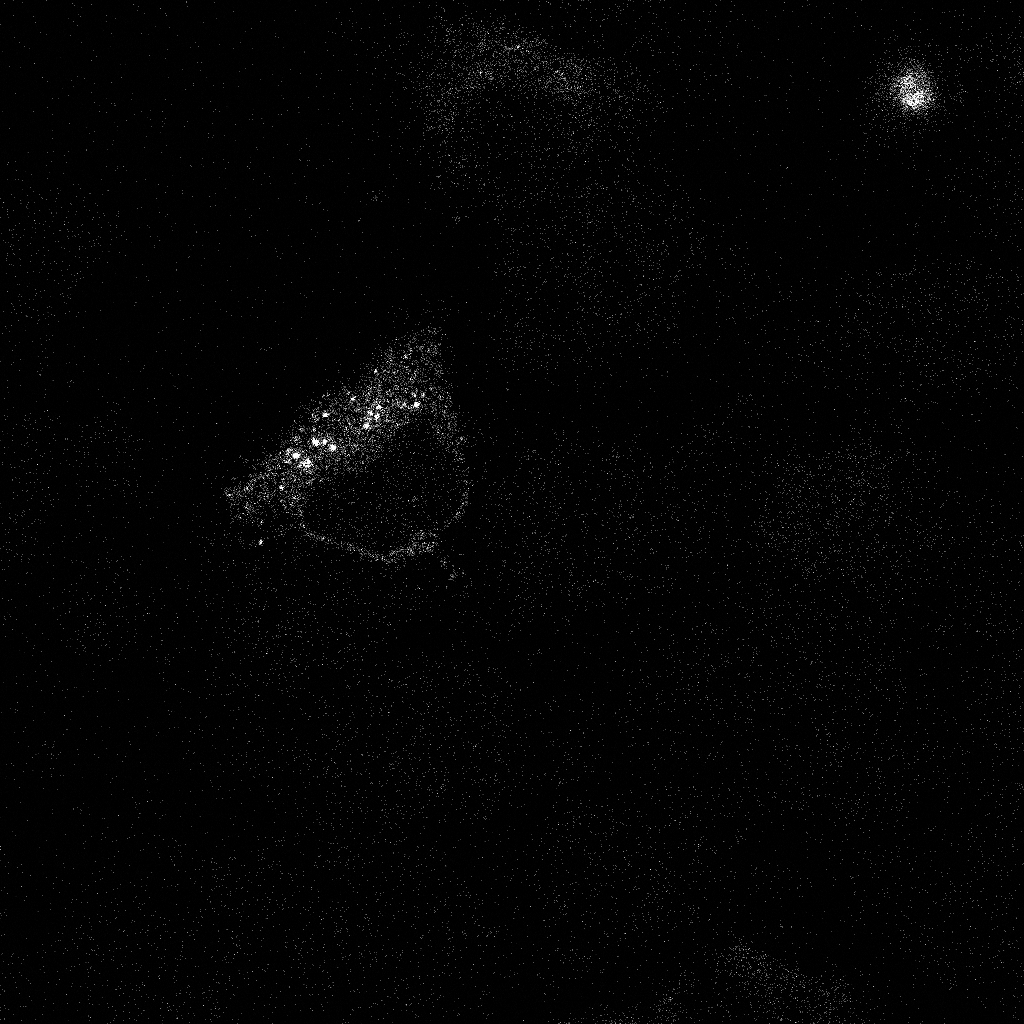

Supplement: Supplementary file 14 — Figure EV4 Source Data [file 44318_2026_816_MOESM14_ESM.zip › H/+OMM-ER Linker.tif]

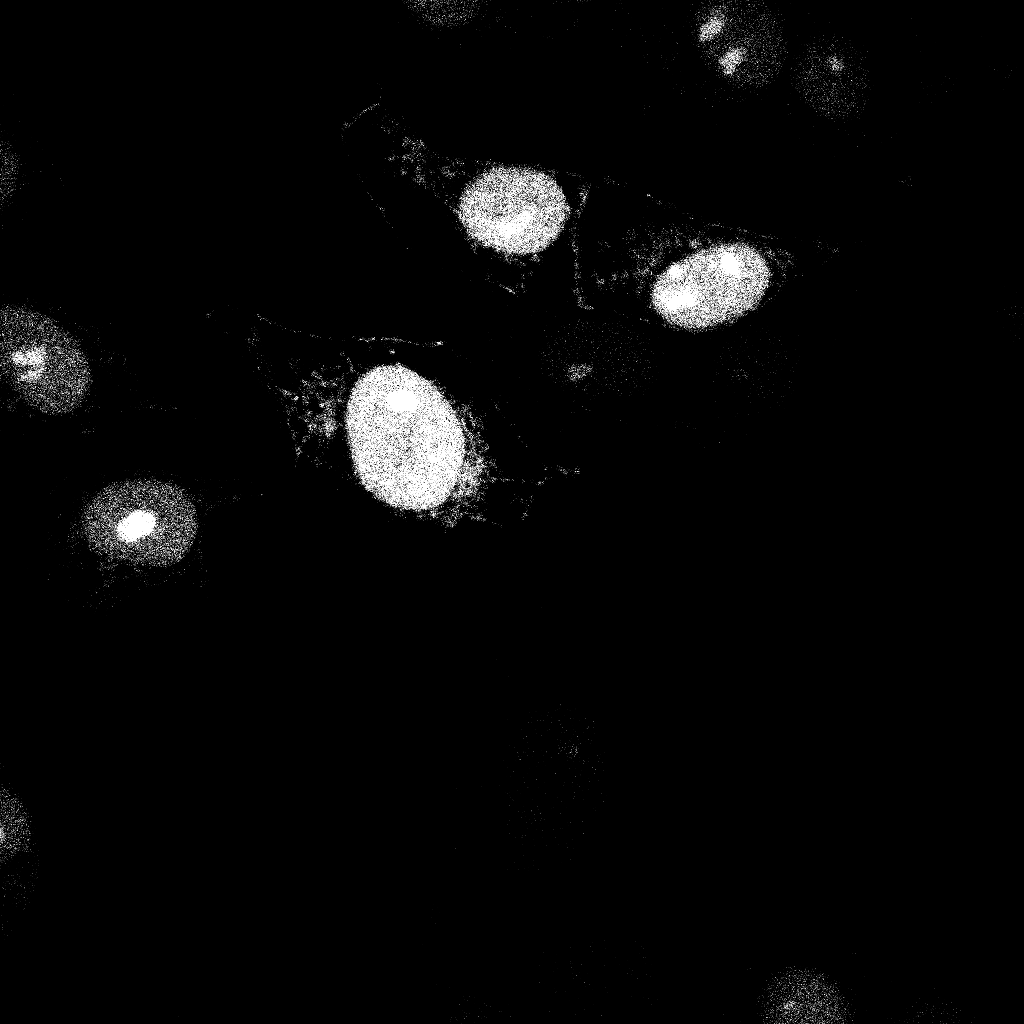

Supplement: Supplementary file 14 — Figure EV4 Source Data [file 44318_2026_816_MOESM14_ESM.zip › H/Ctrl.tif]

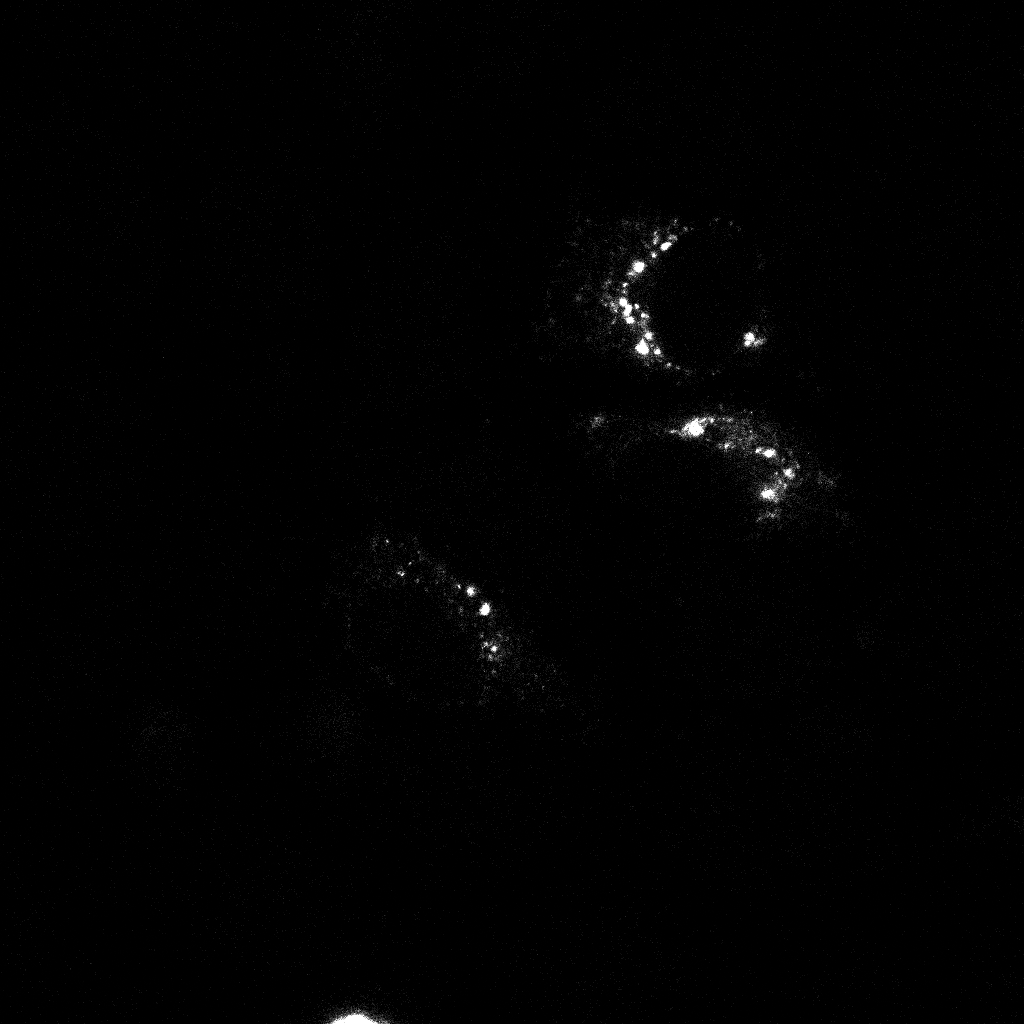

Supplement: Supplementary file 14 — Figure EV4 Source Data [file 44318_2026_816_MOESM14_ESM.zip › H/NSP3+NSP4.tif]

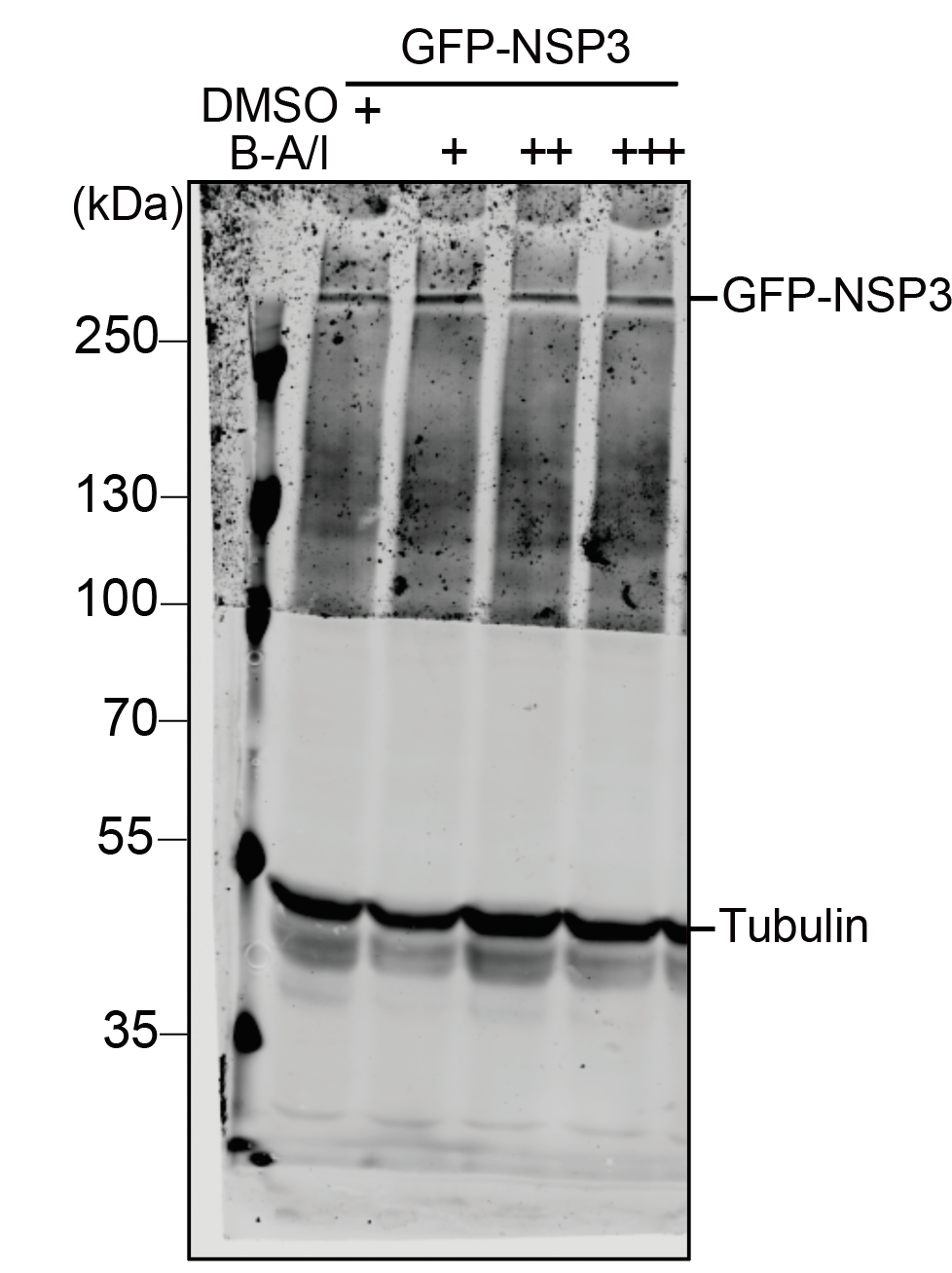

Supplement: Supplementary file 14 — Figure EV4 Source Data [file 44318_2026_816_MOESM14_ESM.zip › I/GFP-NSP3+Tubulin.tif]

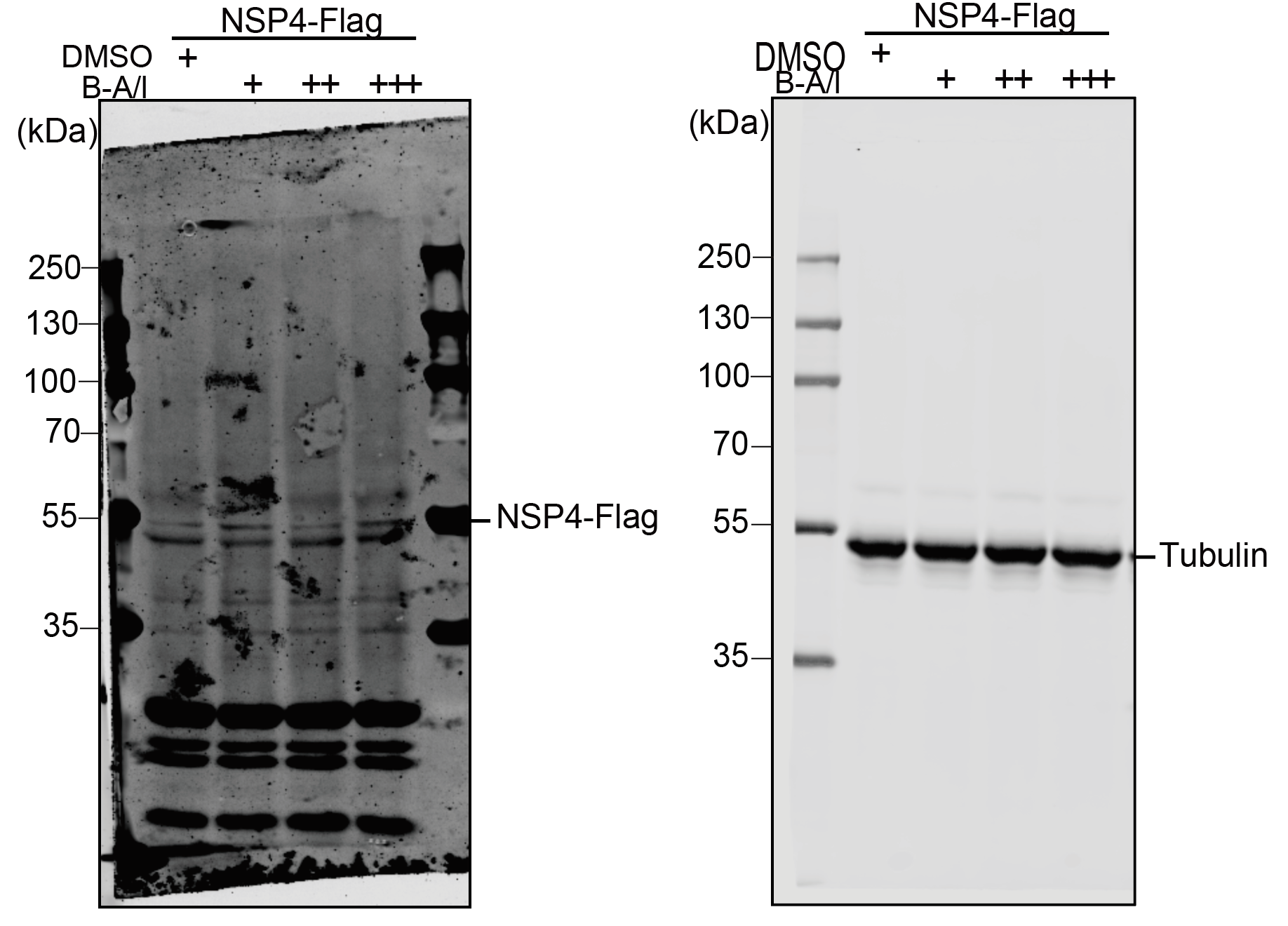

Supplement: Supplementary file 14 — Figure EV4 Source Data [file 44318_2026_816_MOESM14_ESM.zip › J/NSP4-Flag+Tubulin.tif]

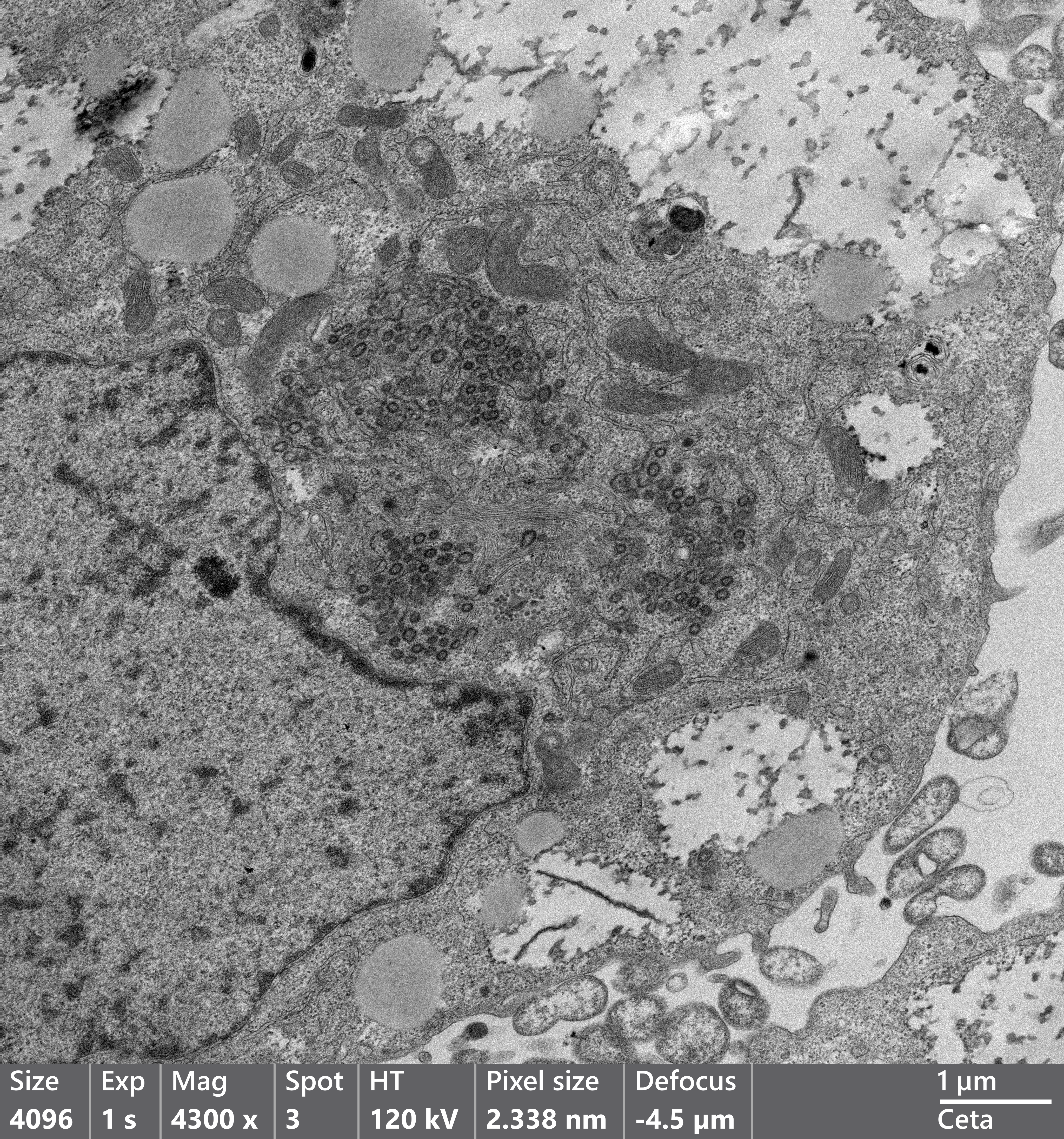

Supplement: Supplementary file 15 — Figure EV5 Source Data [file 44318_2026_816_MOESM15_ESM.zip › A/Figure EV5A B-A-L.tif]

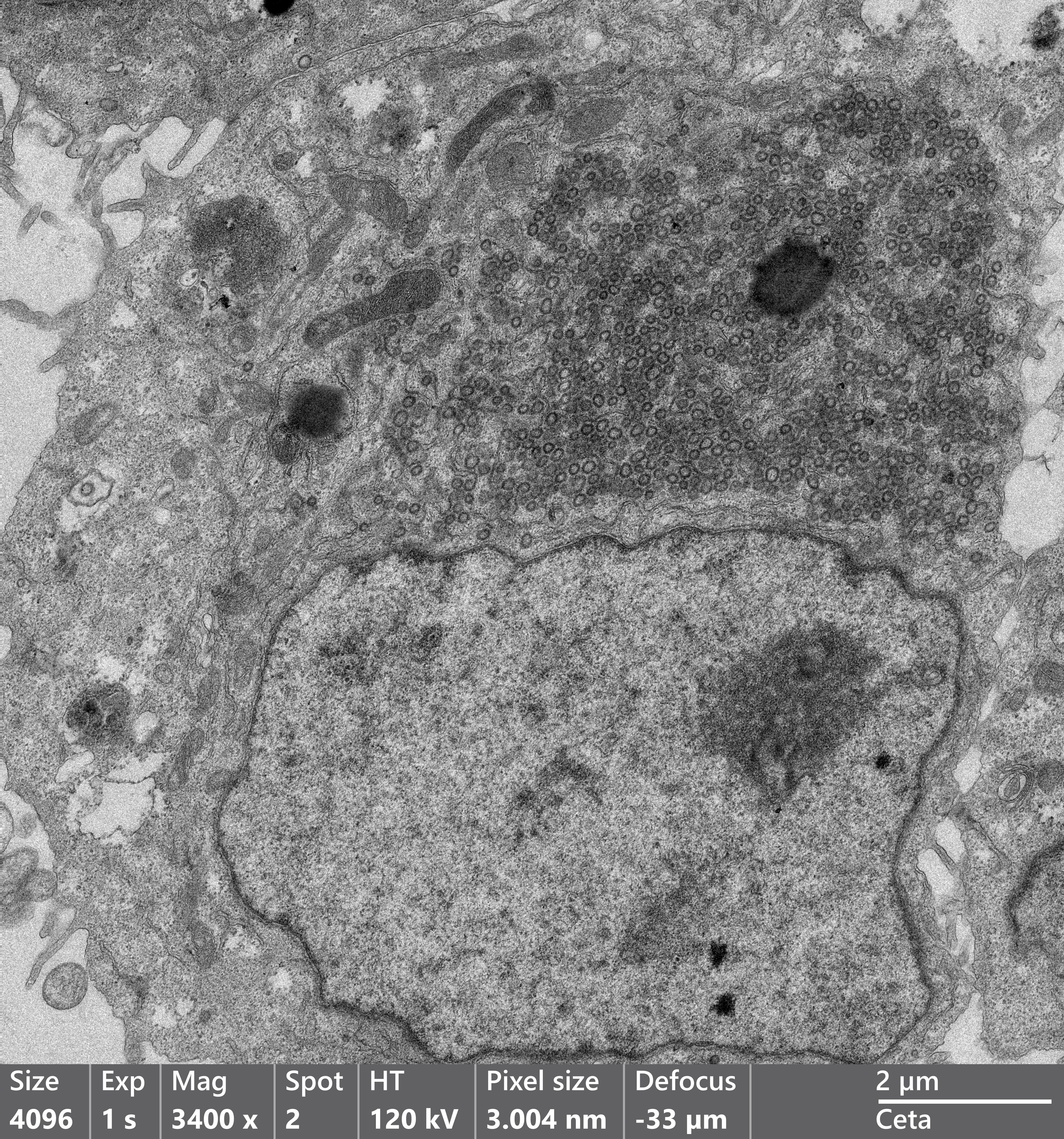

Supplement: Supplementary file 15 — Figure EV5 Source Data [file 44318_2026_816_MOESM15_ESM.zip › A/Figure EV5A DMSO.tif]

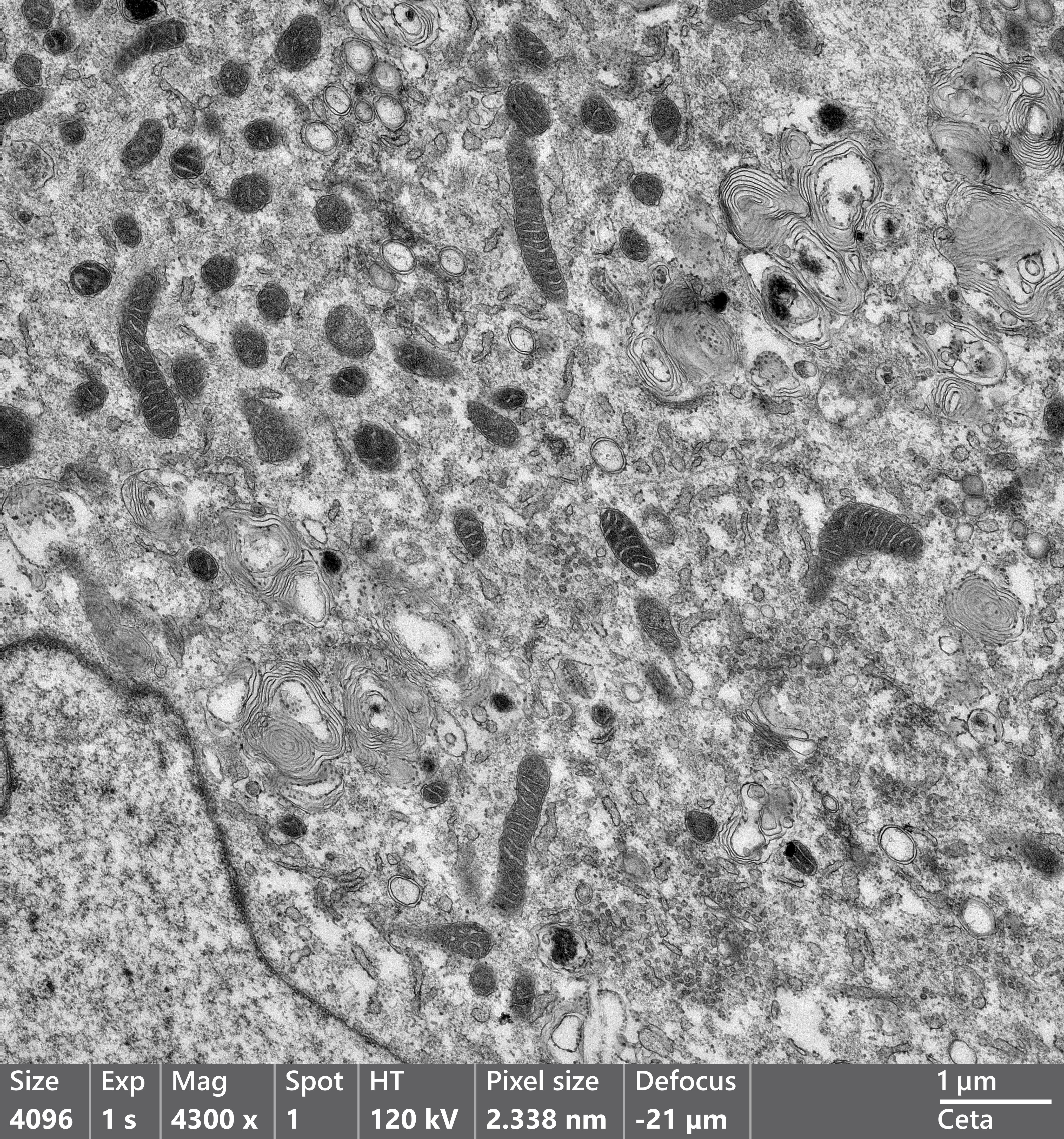

Supplement: Supplementary file 15 — Figure EV5 Source Data [file 44318_2026_816_MOESM15_ESM.zip › E/Figure S5E B-AL.tif]

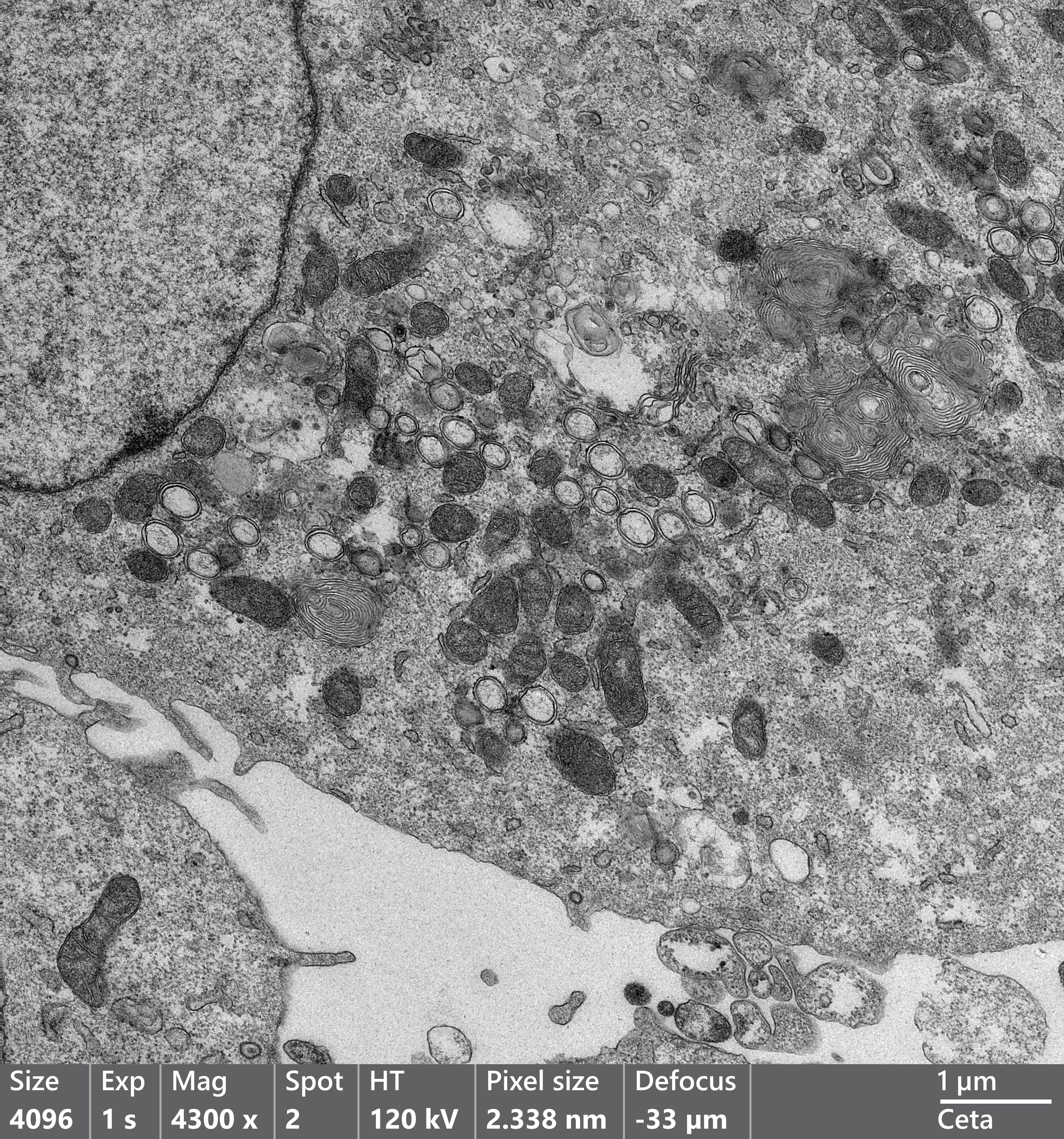

Supplement: Supplementary file 15 — Figure EV5 Source Data [file 44318_2026_816_MOESM15_ESM.zip › E/Figure S5E DMSO.tif]

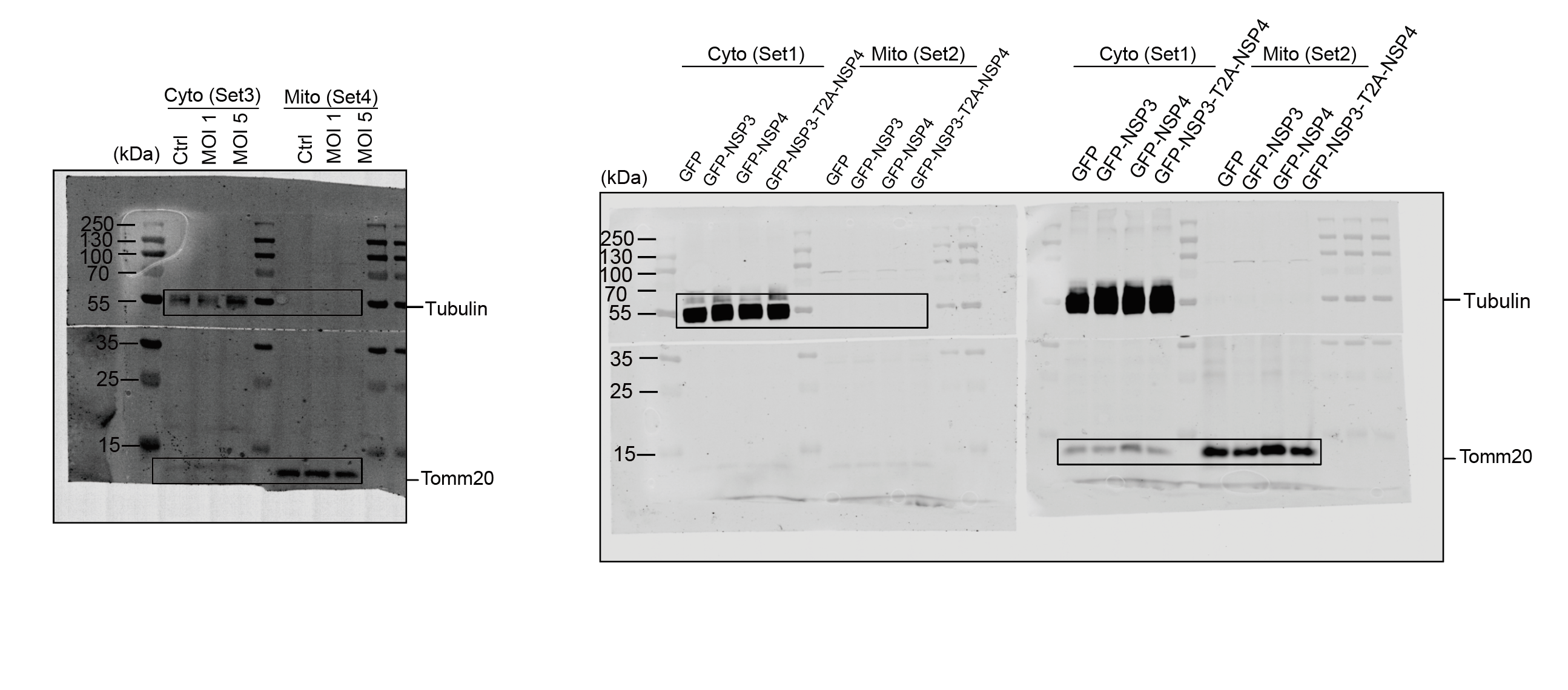

Supplement: Supplementary file 16 — Figure EV6 Source Data [file 44318_2026_816_MOESM16_ESM.zip › A/Tomm20+Tubulin.tif]

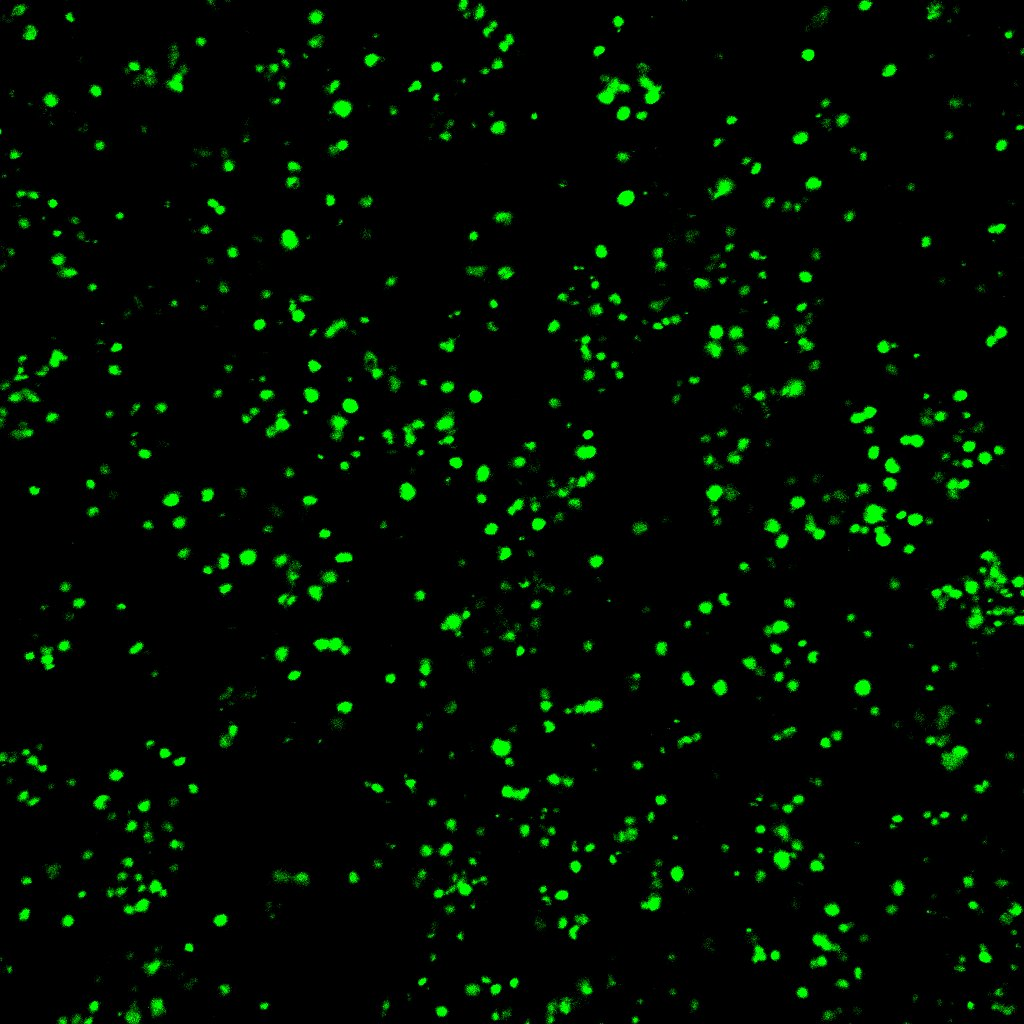

Supplement: Supplementary file 16 — Figure EV6 Source Data [file 44318_2026_816_MOESM16_ESM.zip › C/ACAT1.tif]

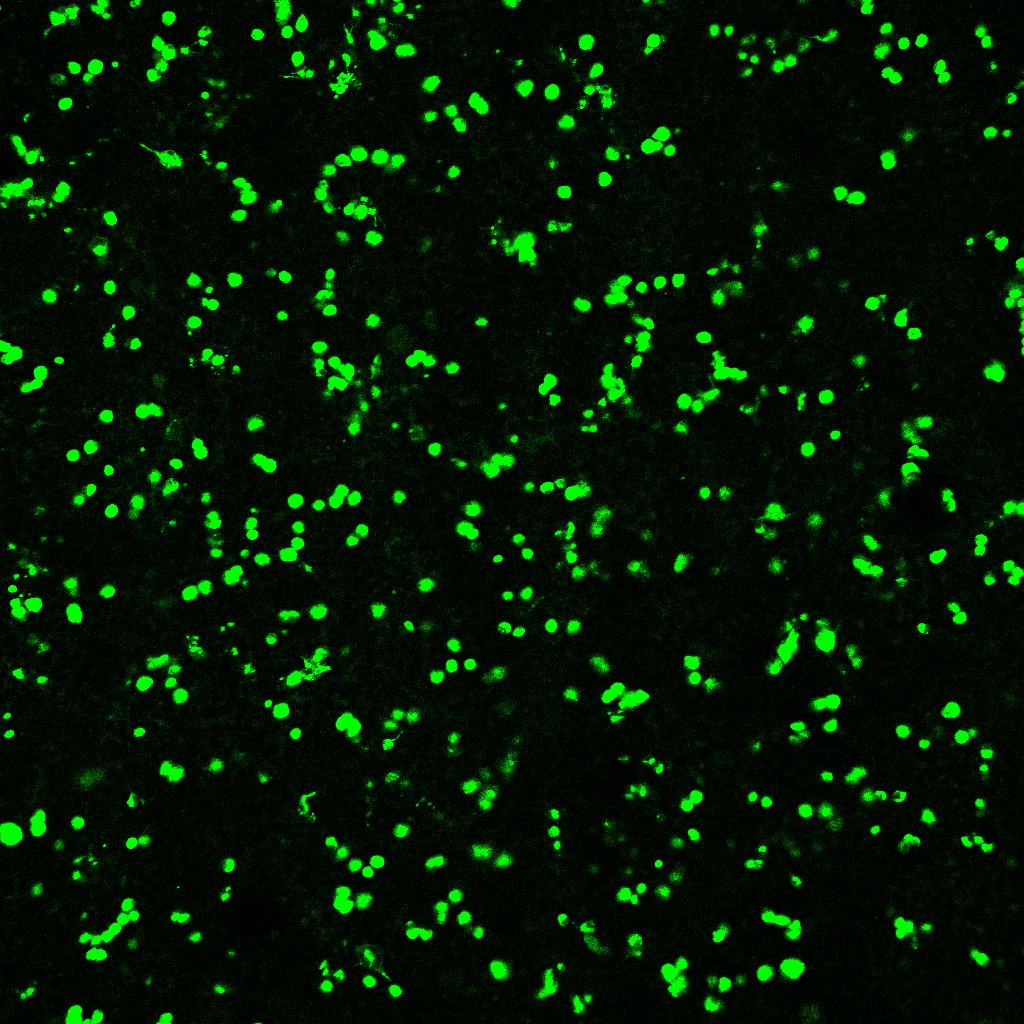

Supplement: Supplementary file 16 — Figure EV6 Source Data [file 44318_2026_816_MOESM16_ESM.zip › C/ATP5F1C.tif]

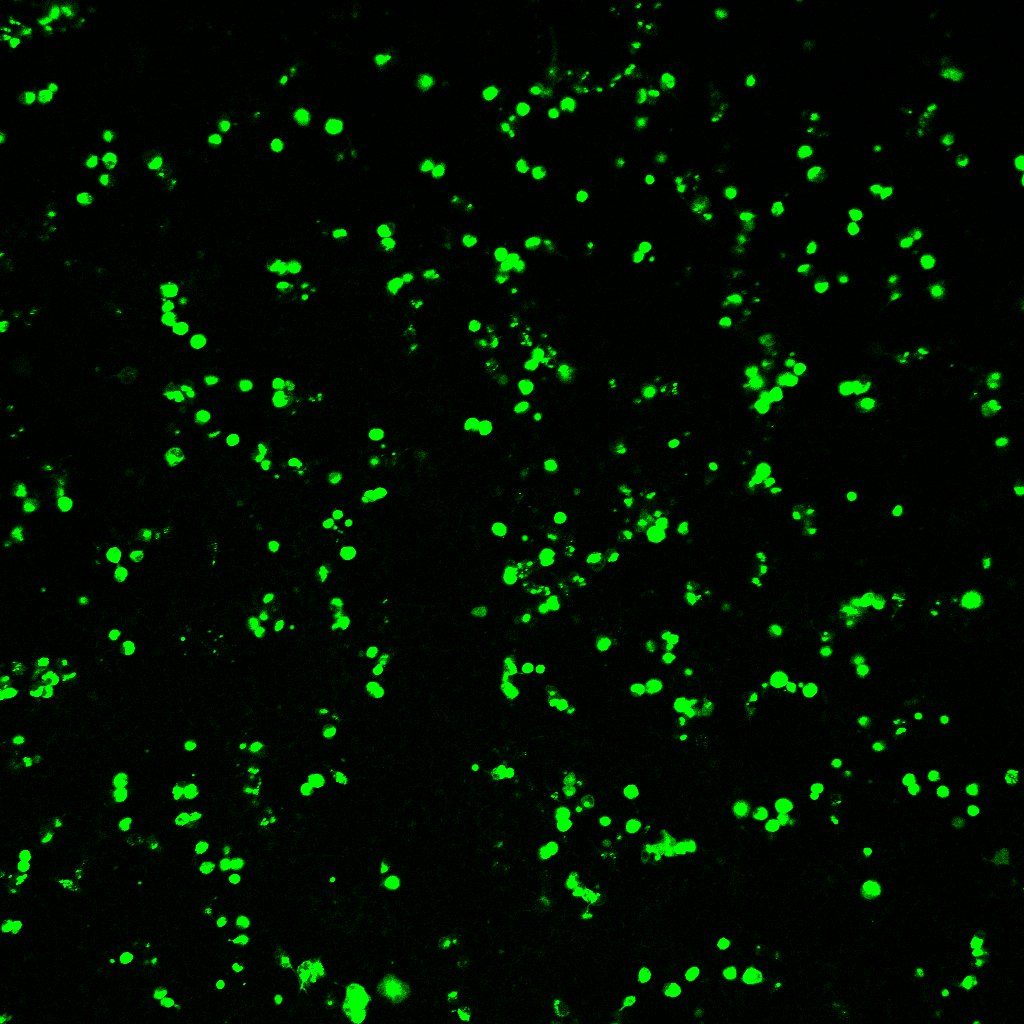

Supplement: Supplementary file 16 — Figure EV6 Source Data [file 44318_2026_816_MOESM16_ESM.zip › C/DLD.tif]

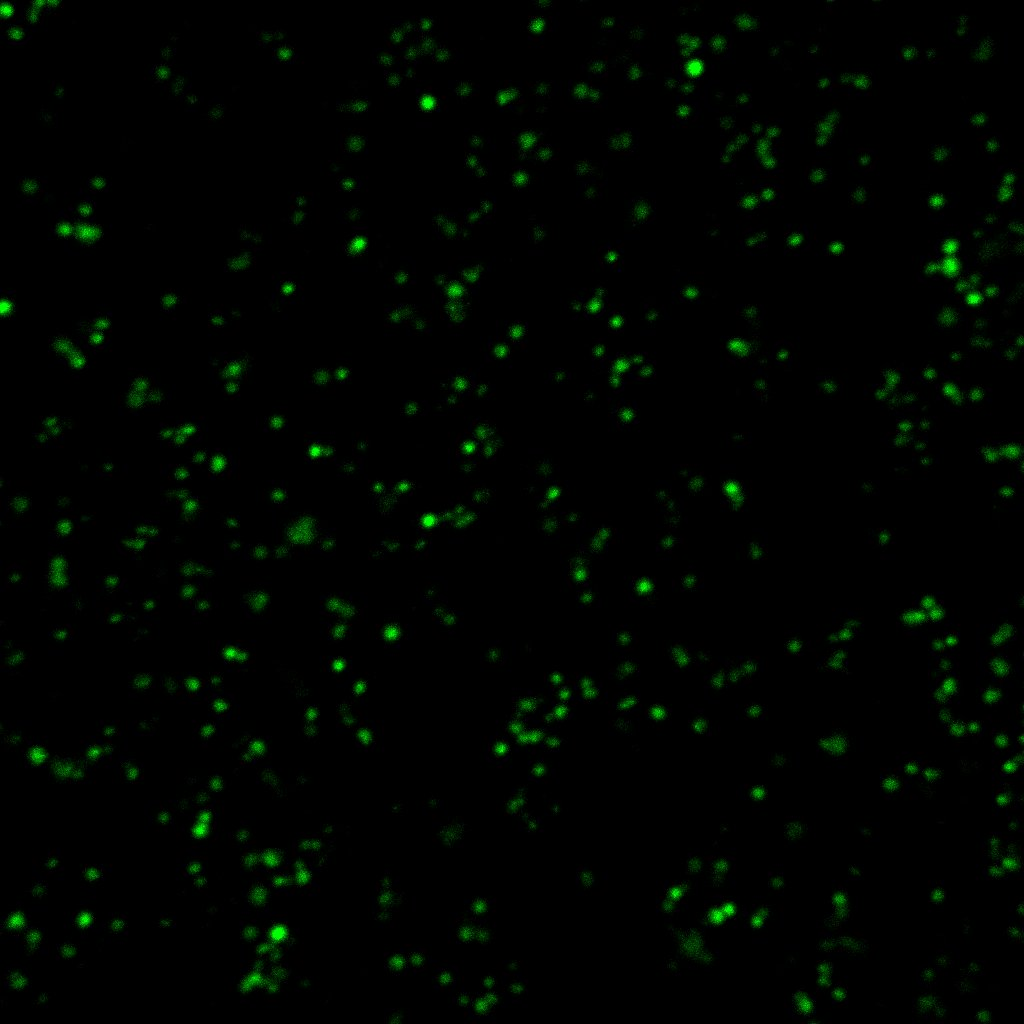

Supplement: Supplementary file 16 — Figure EV6 Source Data [file 44318_2026_816_MOESM16_ESM.zip › C/ECHS1.tif]

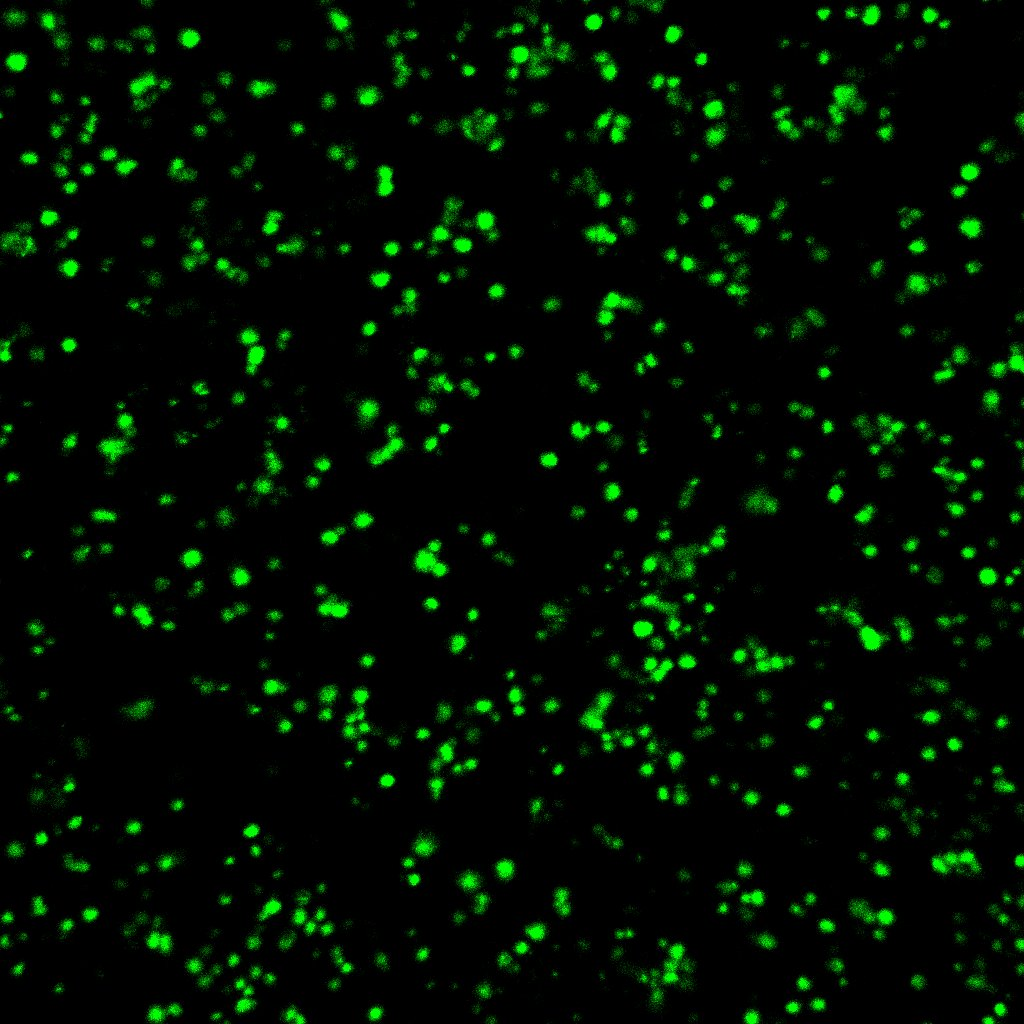

Supplement: Supplementary file 16 — Figure EV6 Source Data [file 44318_2026_816_MOESM16_ESM.zip › C/IDH3A.tif]

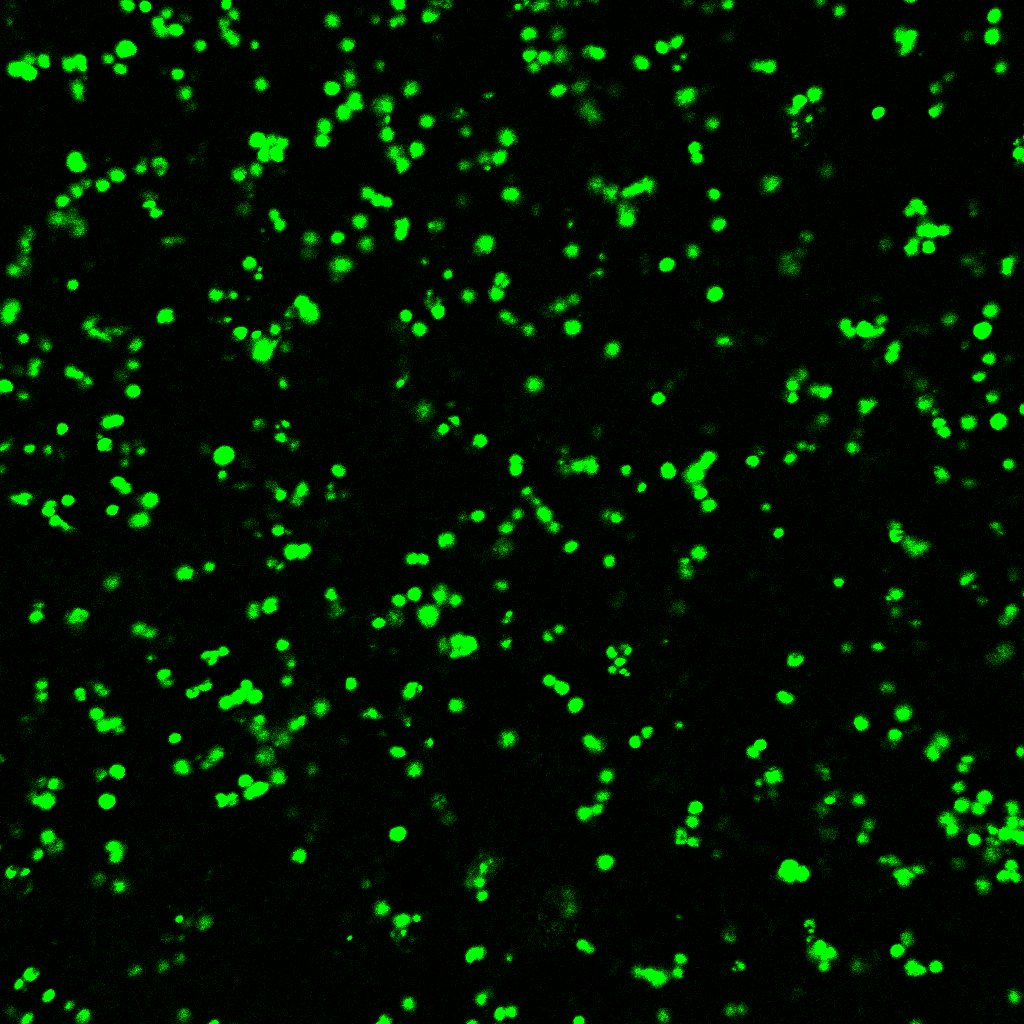

Supplement: Supplementary file 16 — Figure EV6 Source Data [file 44318_2026_816_MOESM16_ESM.zip › C/LONP1.tif]

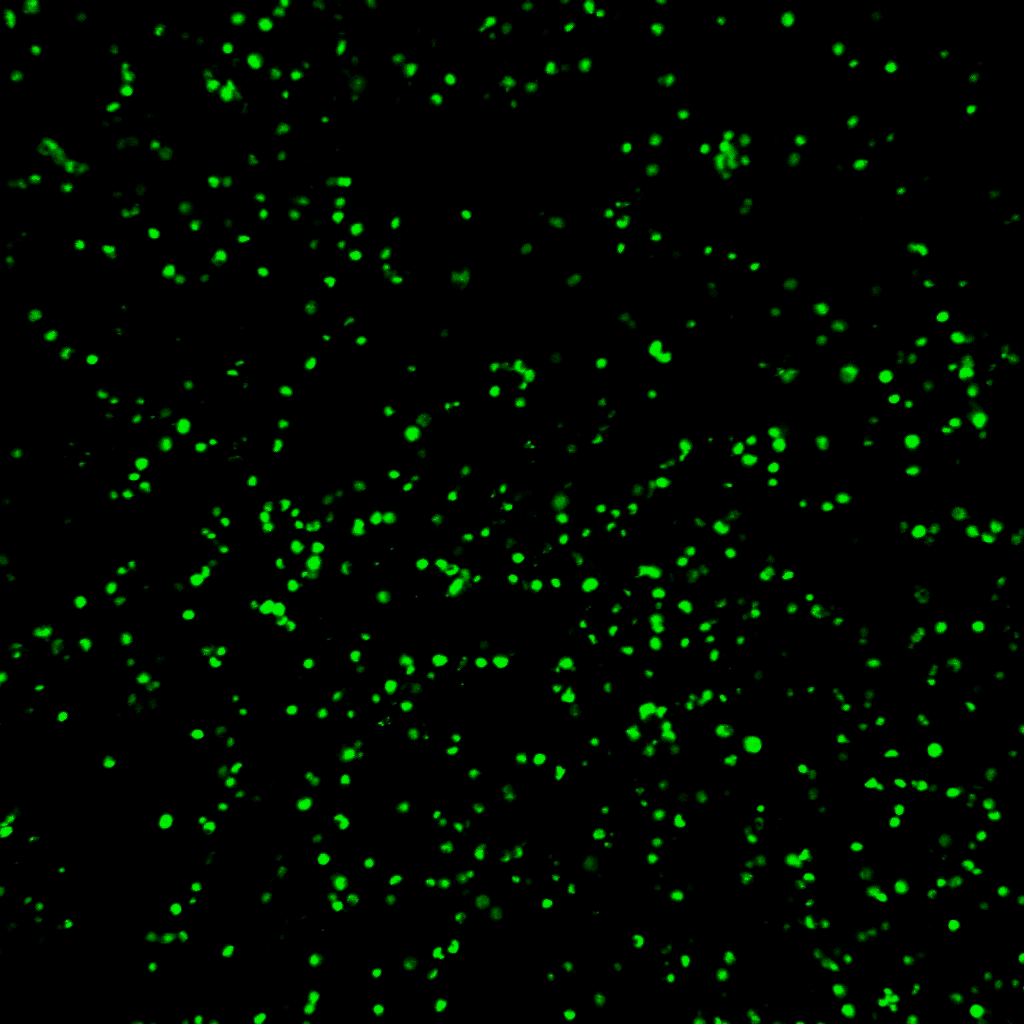

Supplement: Supplementary file 16 — Figure EV6 Source Data [file 44318_2026_816_MOESM16_ESM.zip › C/MDN2.tif]

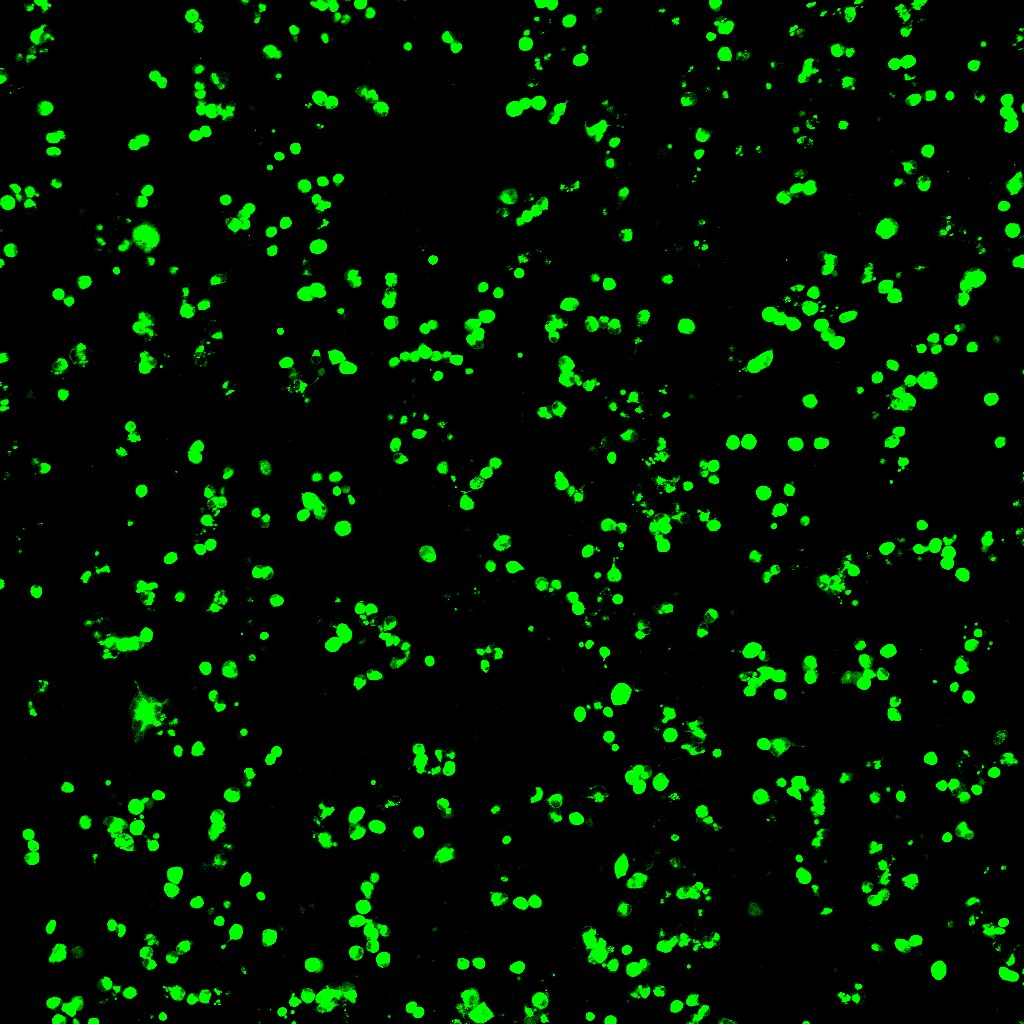

Supplement: Supplementary file 16 — Figure EV6 Source Data [file 44318_2026_816_MOESM16_ESM.zip › C/NC.tif]

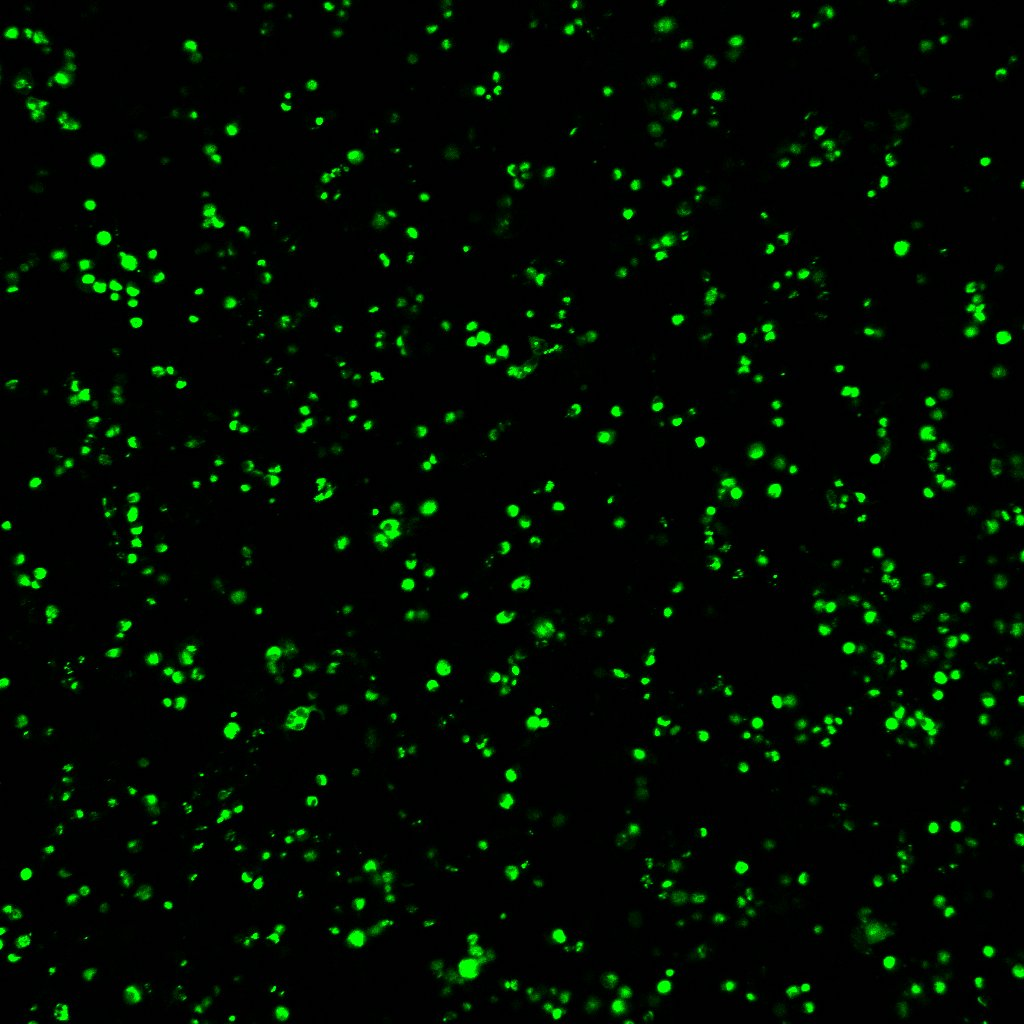

Supplement: Supplementary file 16 — Figure EV6 Source Data [file 44318_2026_816_MOESM16_ESM.zip › C/PC.tif]

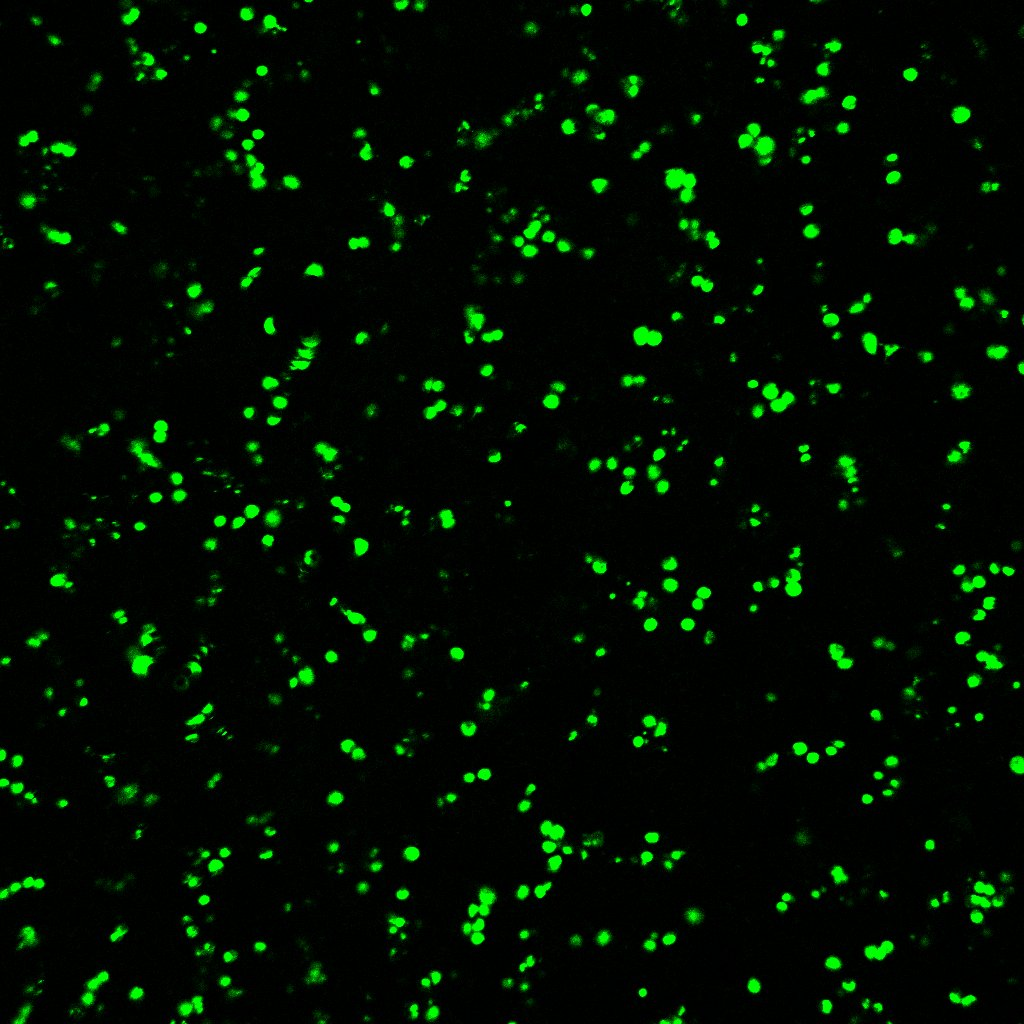

Supplement: Supplementary file 16 — Figure EV6 Source Data [file 44318_2026_816_MOESM16_ESM.zip › C/PMPCB.tif]

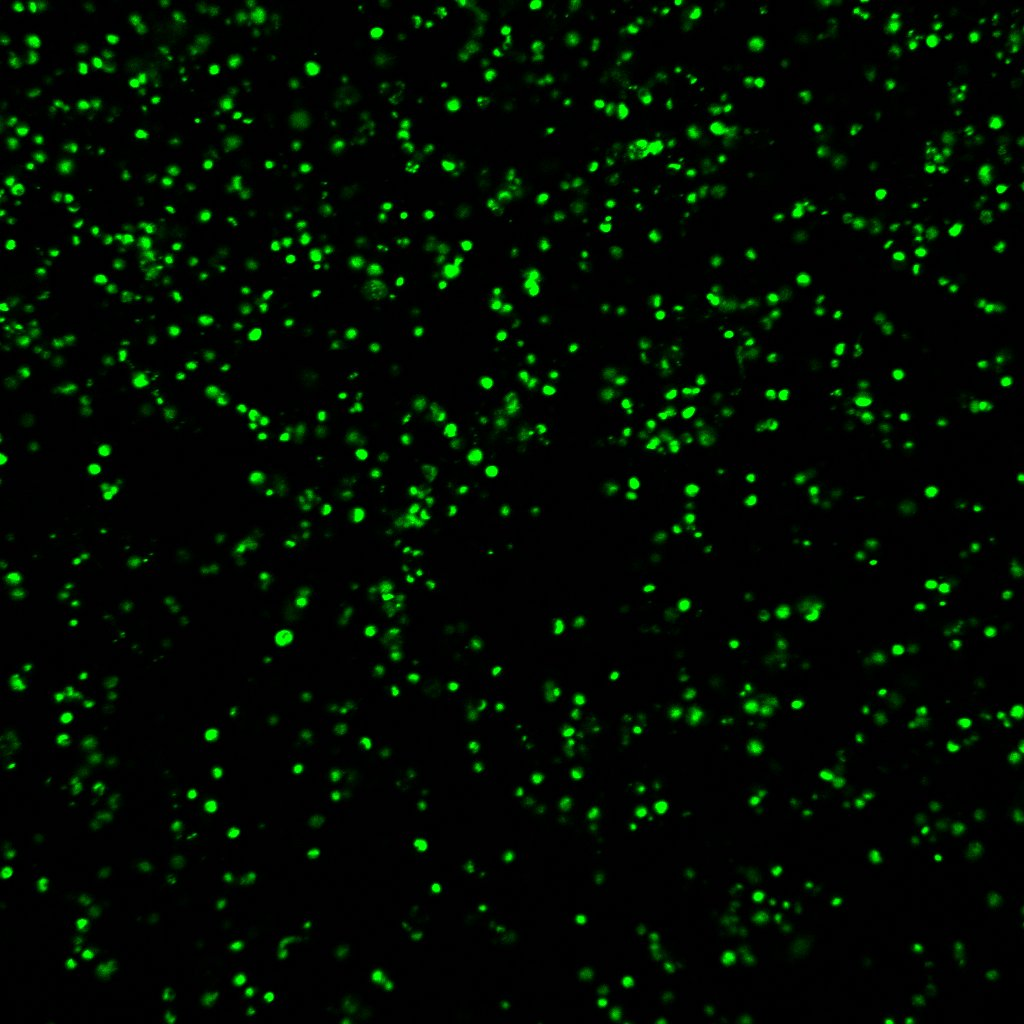

Supplement: Supplementary file 16 — Figure EV6 Source Data [file 44318_2026_816_MOESM16_ESM.zip › C/PRDX3.tif]

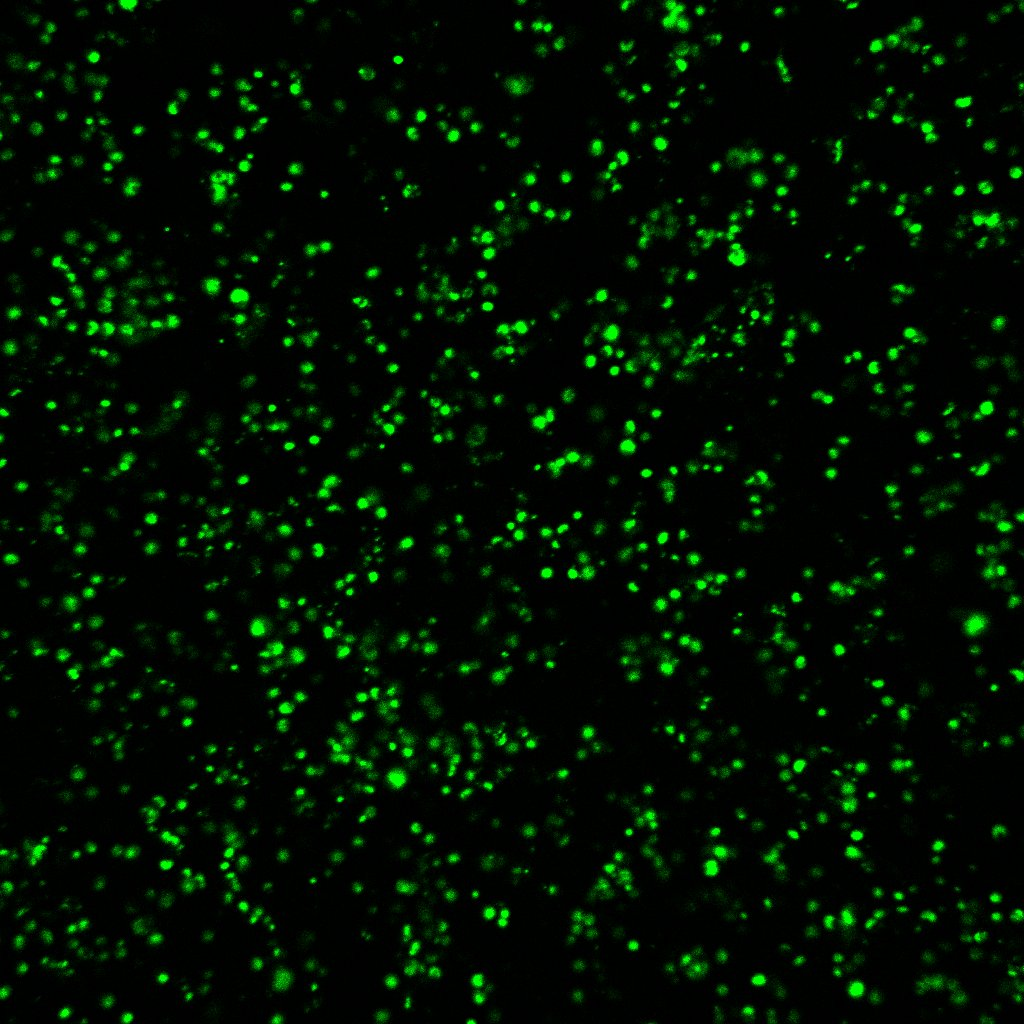

Supplement: Supplementary file 16 — Figure EV6 Source Data [file 44318_2026_816_MOESM16_ESM.zip › C/SUCLG1.tif]

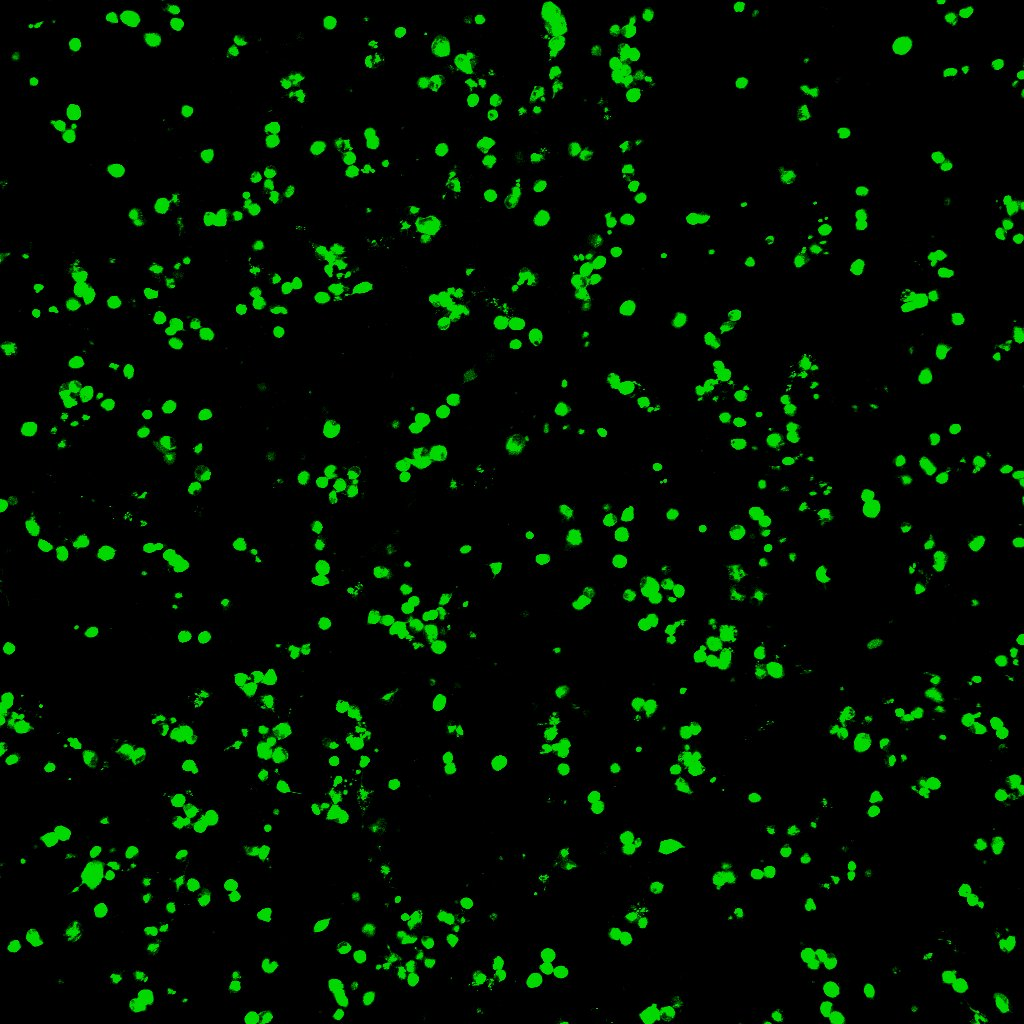

Supplement: Supplementary file 16 — Figure EV6 Source Data [file 44318_2026_816_MOESM16_ESM.zip › C/SUCLG2.tif]

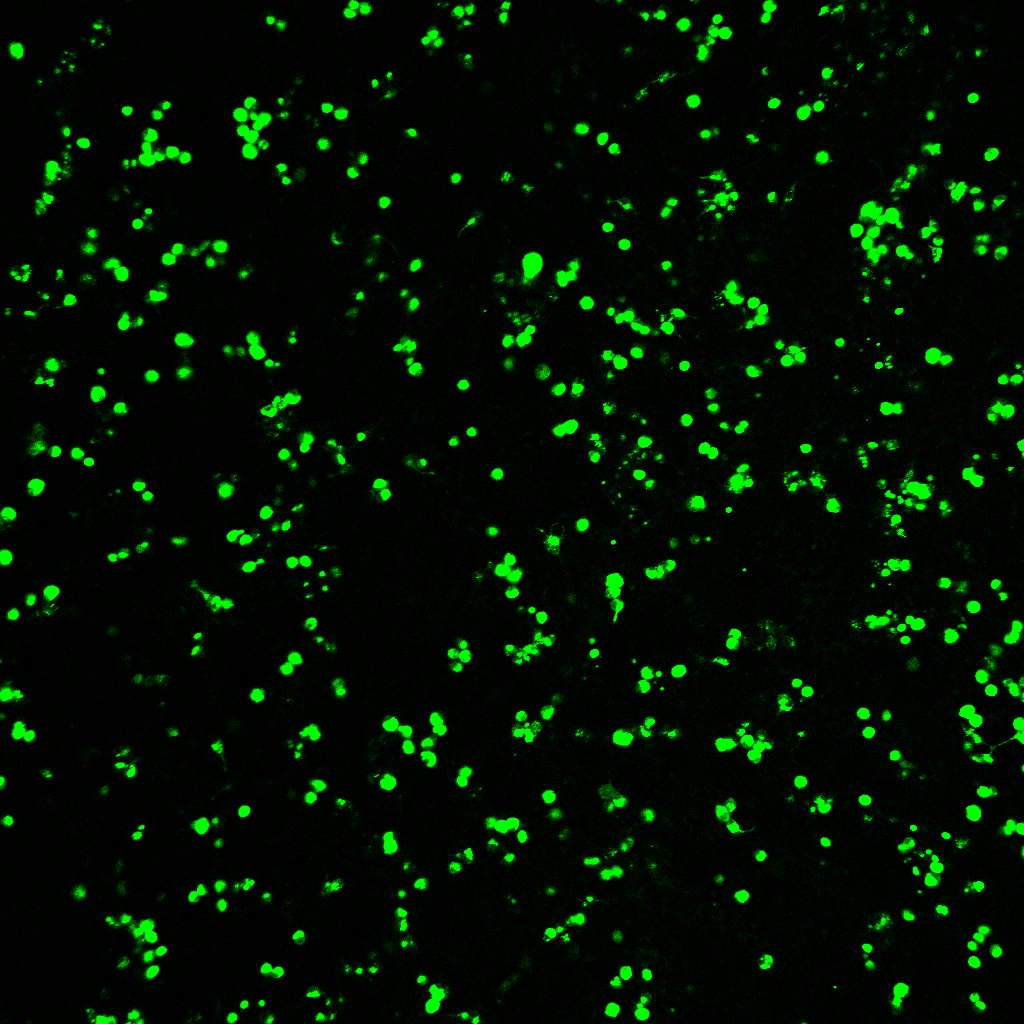

Supplement: Supplementary file 16 — Figure EV6 Source Data [file 44318_2026_816_MOESM16_ESM.zip › C/TIMM44.tif]

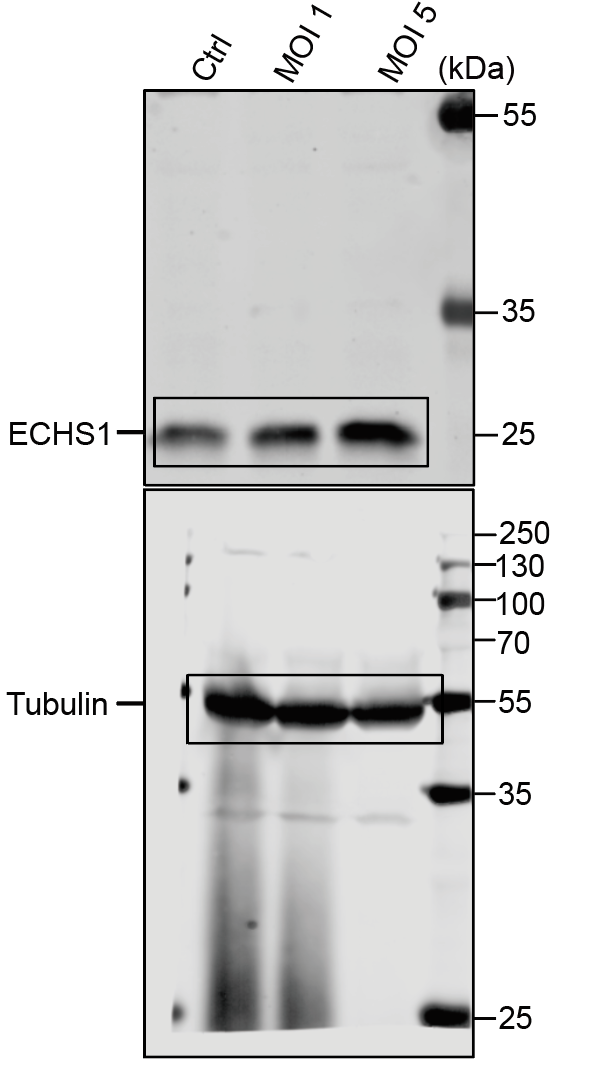

Supplement: Supplementary file 16 — Figure EV6 Source Data [file 44318_2026_816_MOESM16_ESM.zip › E/ECHS1+Tubulin.tif]

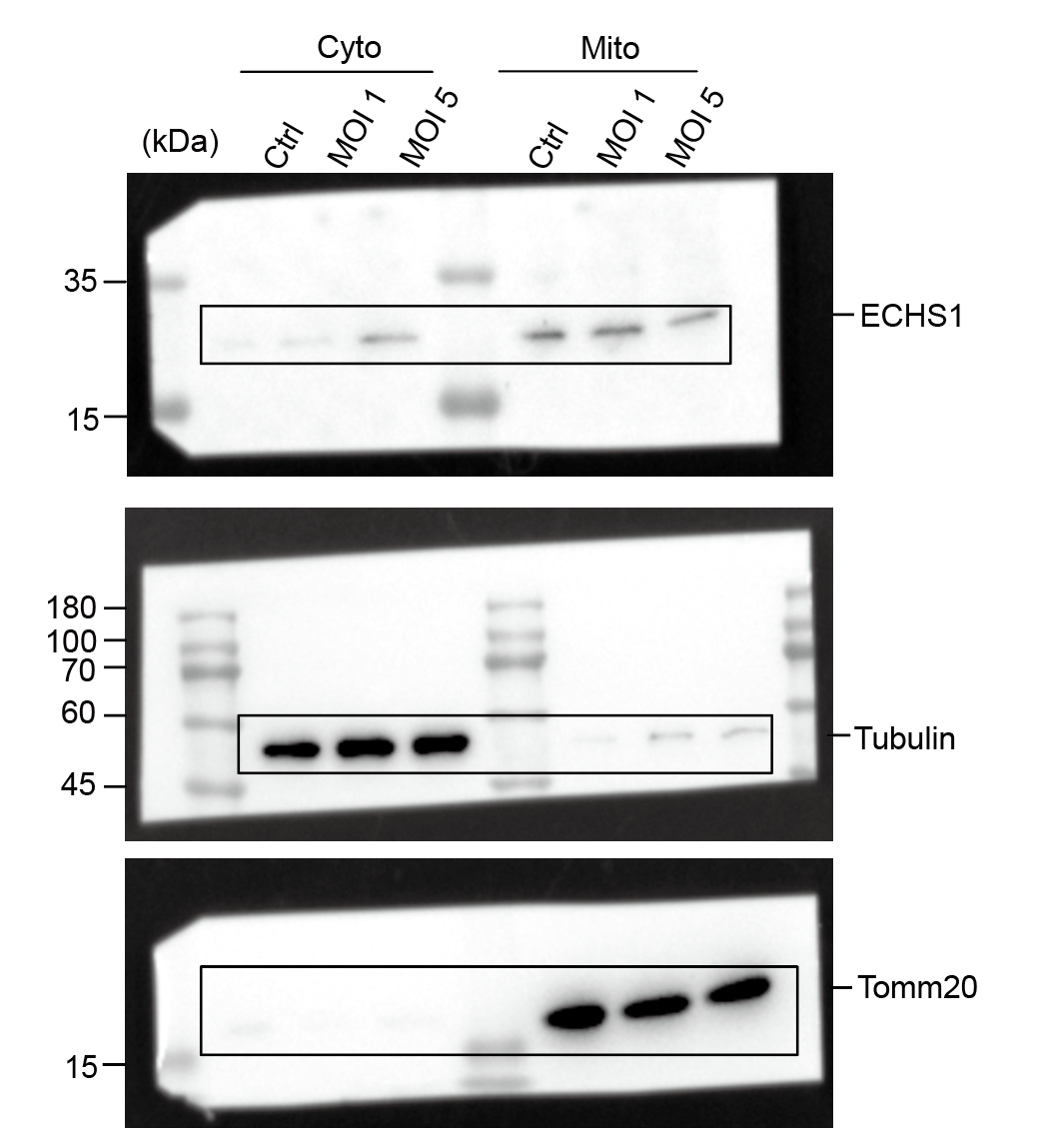

Supplement: Supplementary file 16 — Figure EV6 Source Data [file 44318_2026_816_MOESM16_ESM.zip › F/ECHS1+Tubulin+Tomm20.tif]

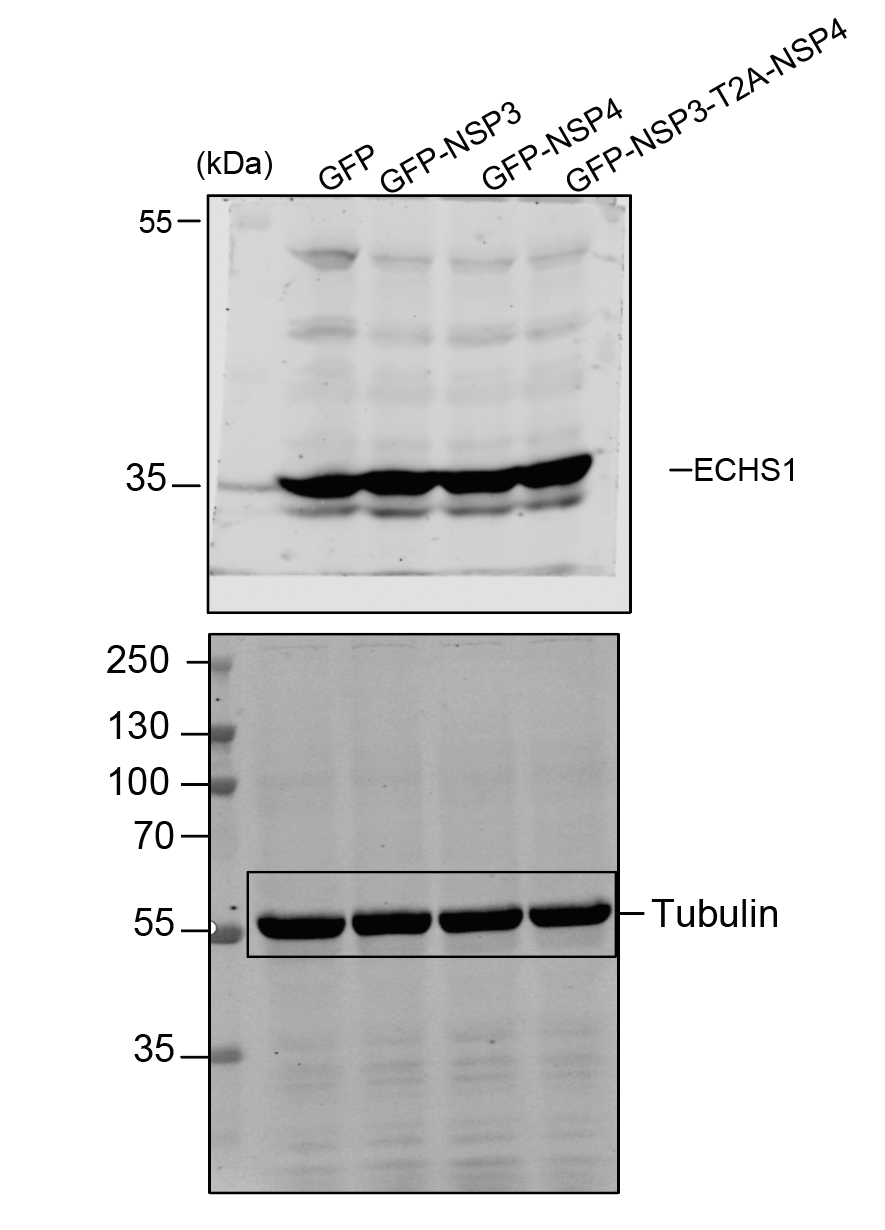

Supplement: Supplementary file 16 — Figure EV6 Source Data [file 44318_2026_816_MOESM16_ESM.zip › G/ECHS1+Tubulin.tif]

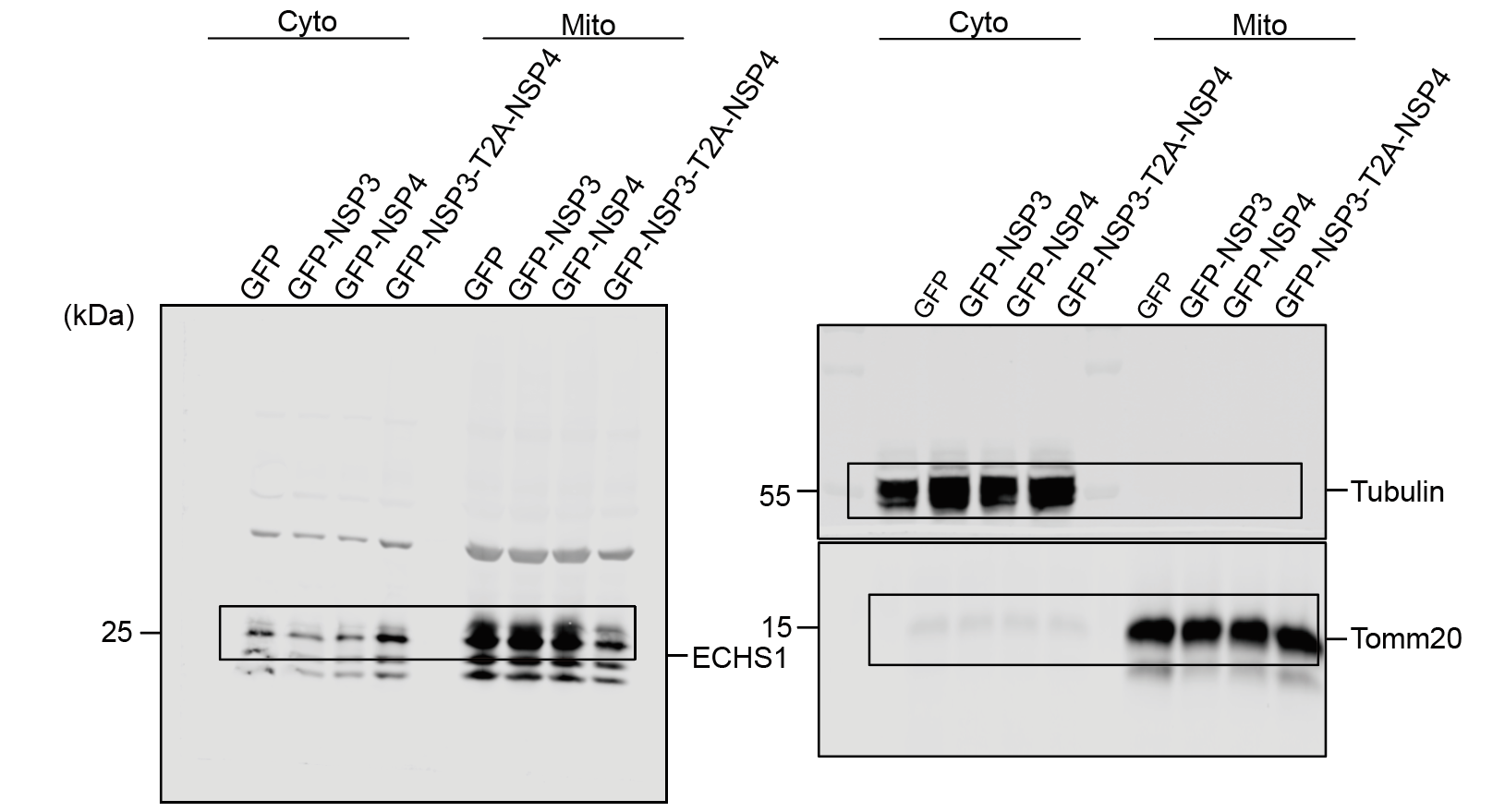

Supplement: Supplementary file 16 — Figure EV6 Source Data [file 44318_2026_816_MOESM16_ESM.zip › H/ECHS1+Tomm20+Tubulin.tif]

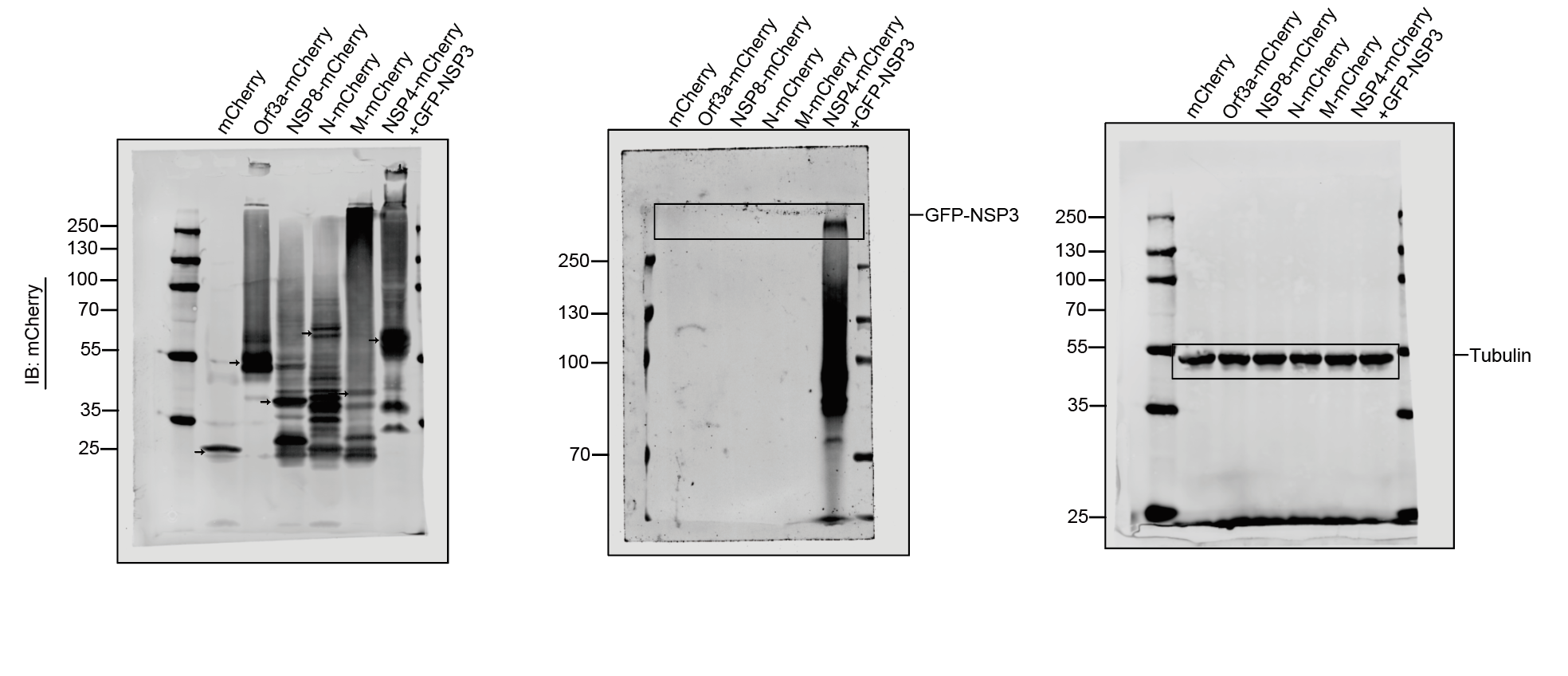

Supplement: Supplementary file 16 — Figure EV6 Source Data [file 44318_2026_816_MOESM16_ESM.zip › I/mCherry+GFP-NSP3+Tubulin.tif]

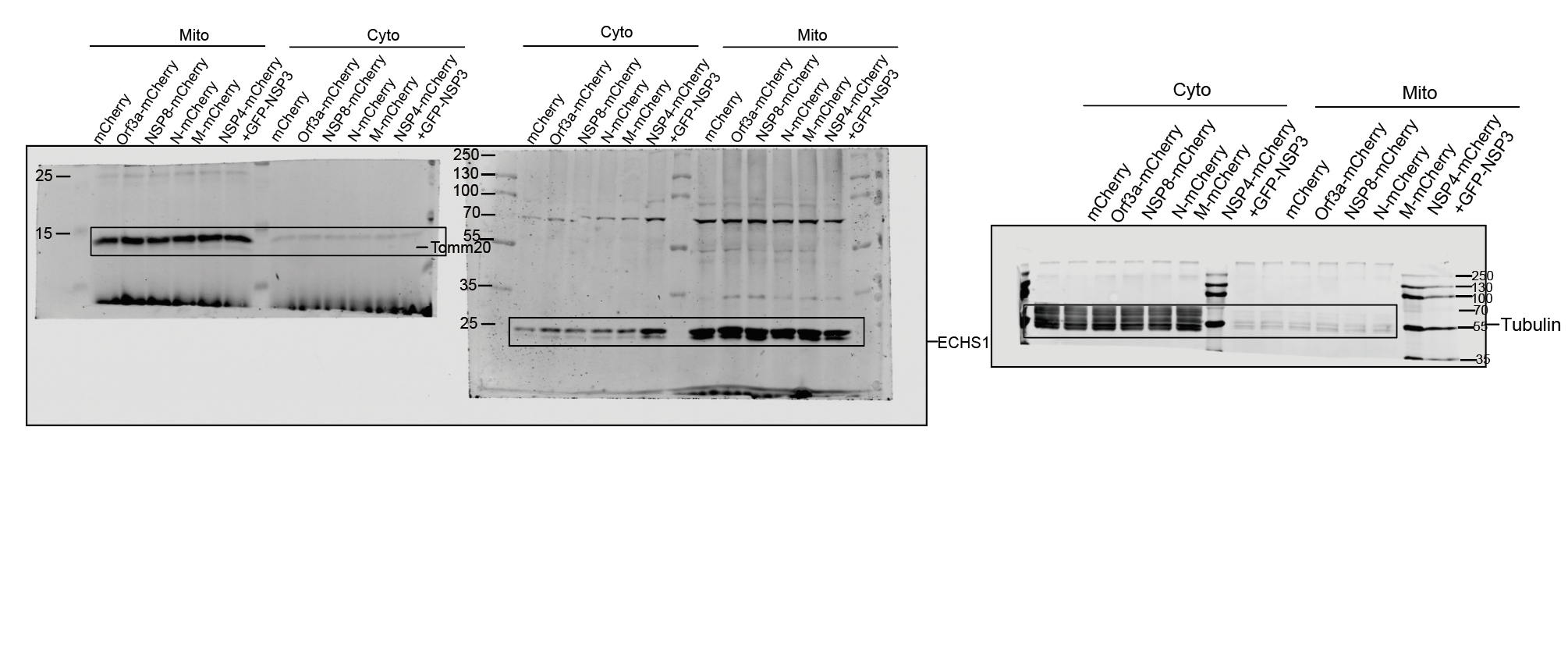

Supplement: Supplementary file 16 — Figure EV6 Source Data [file 44318_2026_816_MOESM16_ESM.zip › J/Tomm20+ECHS1+Tubulin.tif]

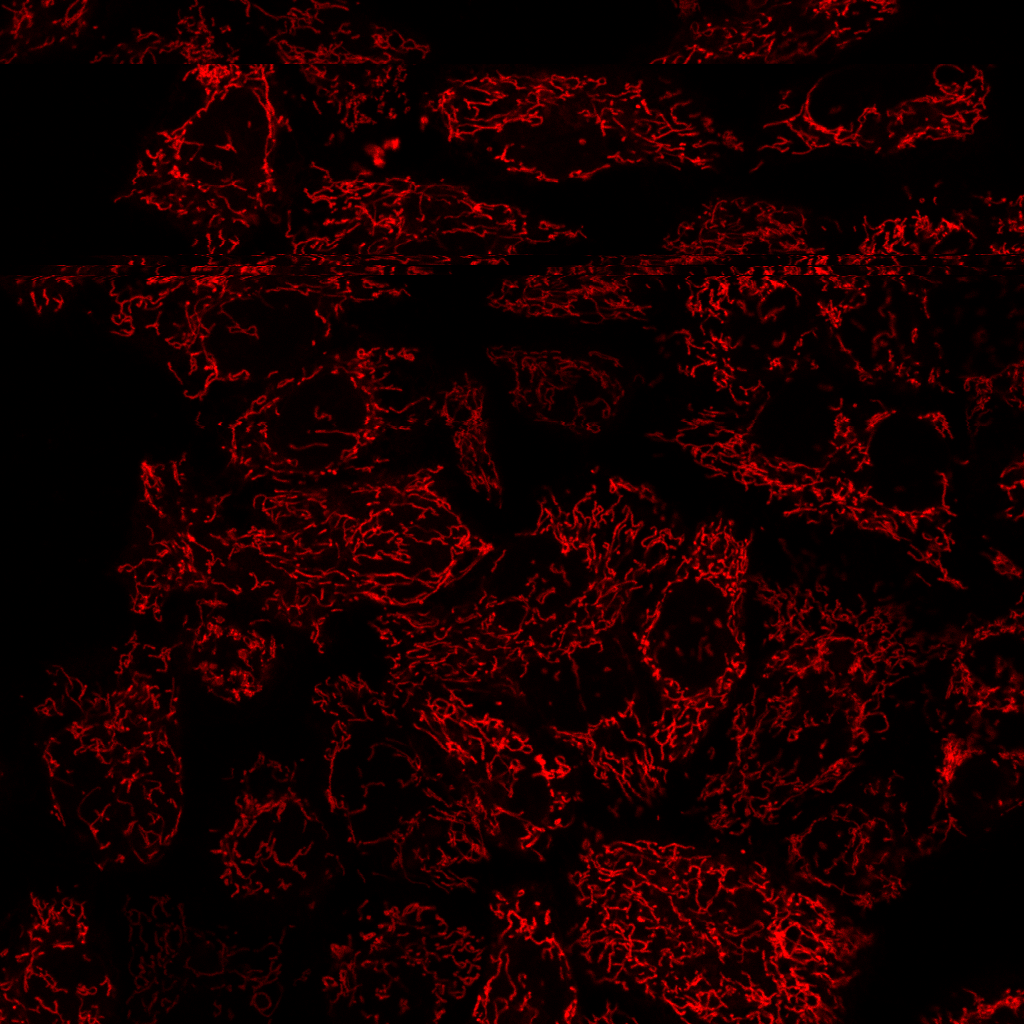

Supplement: Supplementary file 17 — Figure EV7 Source Data [file 44318_2026_816_MOESM17_ESM.zip › A/Ctrl.tif]

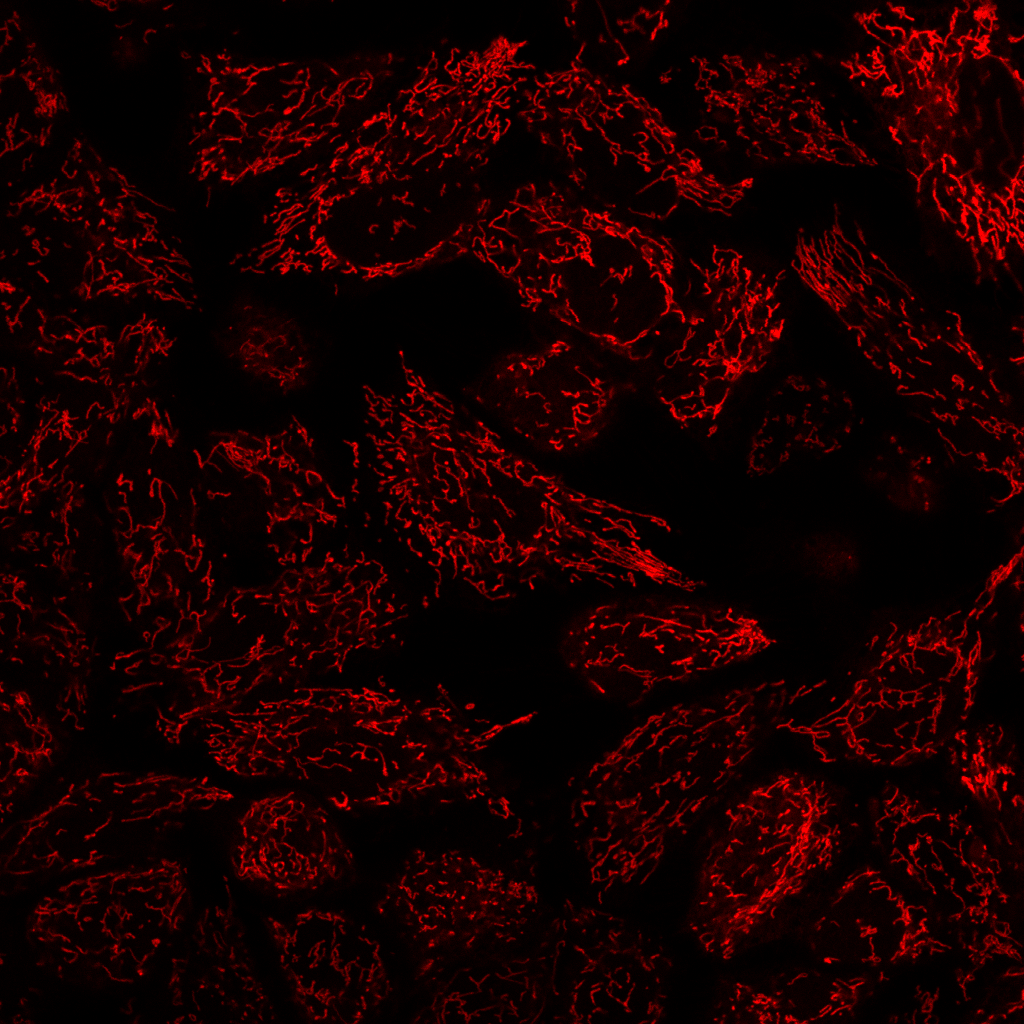

Supplement: Supplementary file 17 — Figure EV7 Source Data [file 44318_2026_816_MOESM17_ESM.zip › A/ECHS1-KO.tif]

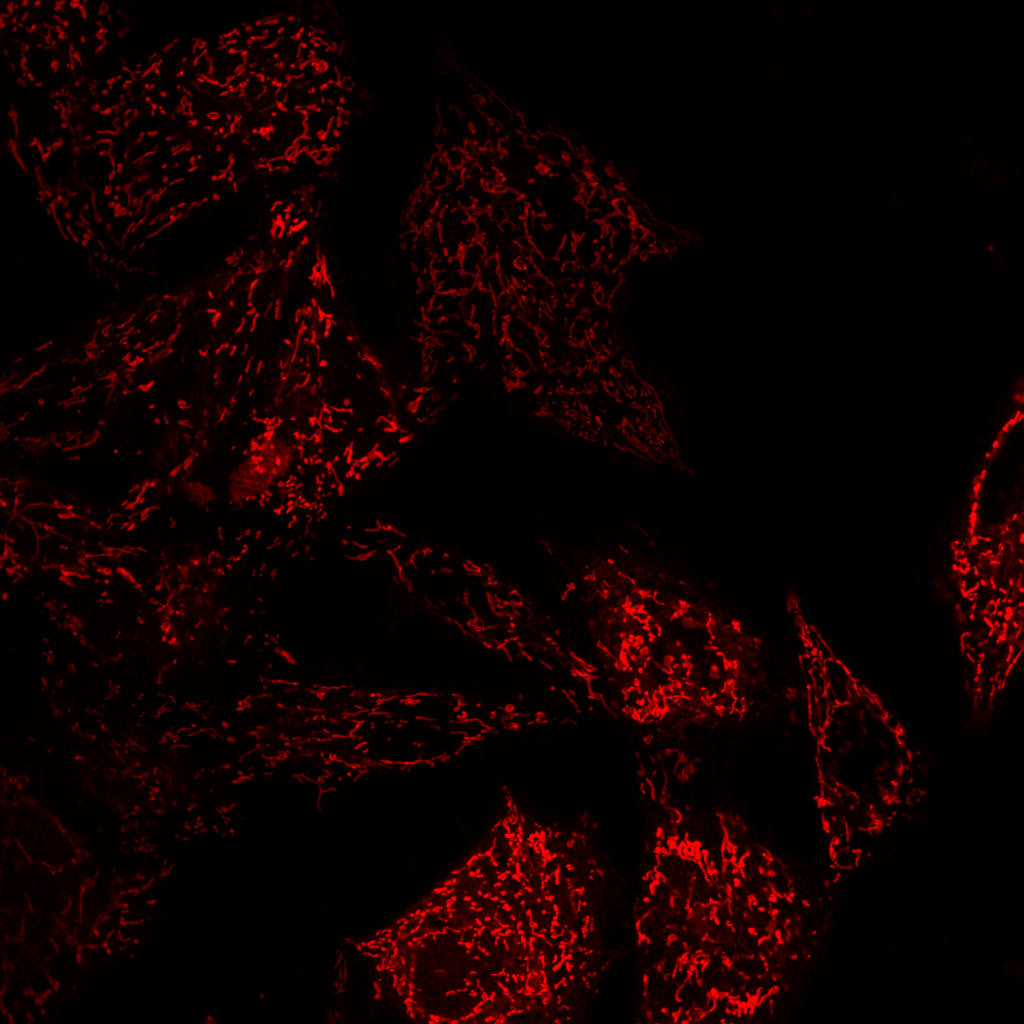

Supplement: Supplementary file 17 — Figure EV7 Source Data [file 44318_2026_816_MOESM17_ESM.zip › A/ECHS1-Rescue.tif]

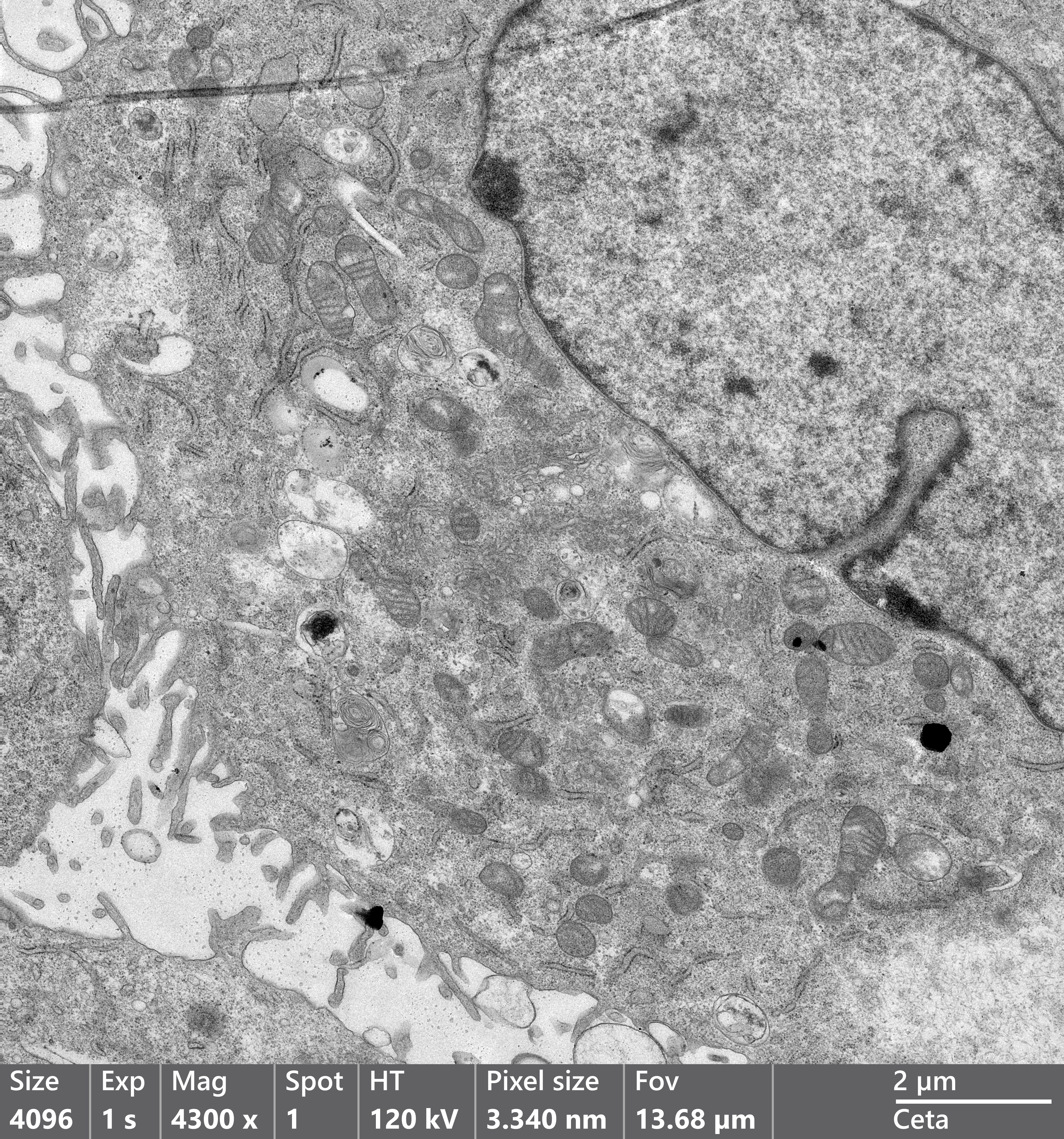

Supplement: Supplementary file 17 — Figure EV7 Source Data [file 44318_2026_816_MOESM17_ESM.zip › B/Ctrl.tif]

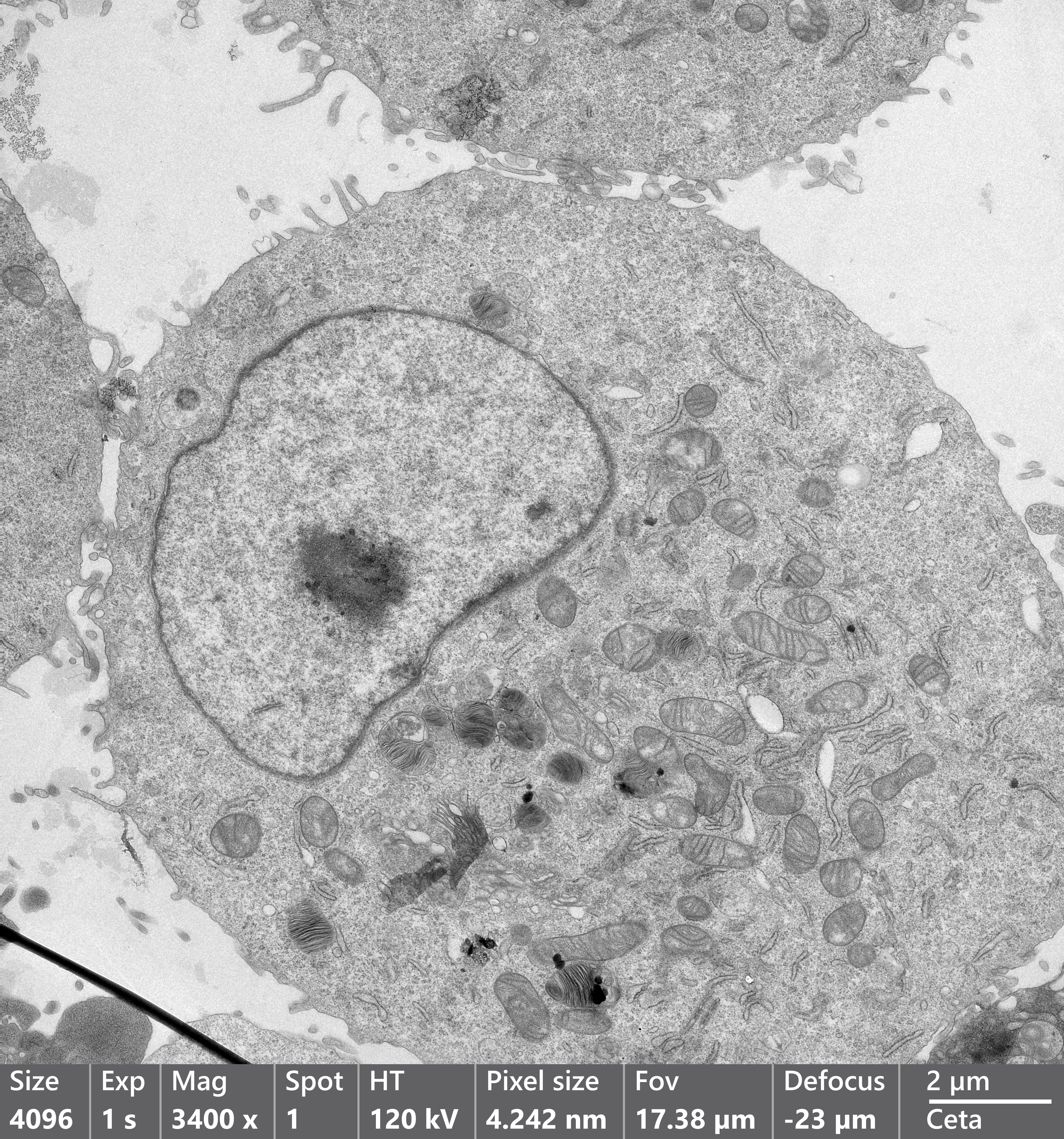

Supplement: Supplementary file 17 — Figure EV7 Source Data [file 44318_2026_816_MOESM17_ESM.zip › B/ECHS1-KO.tif]

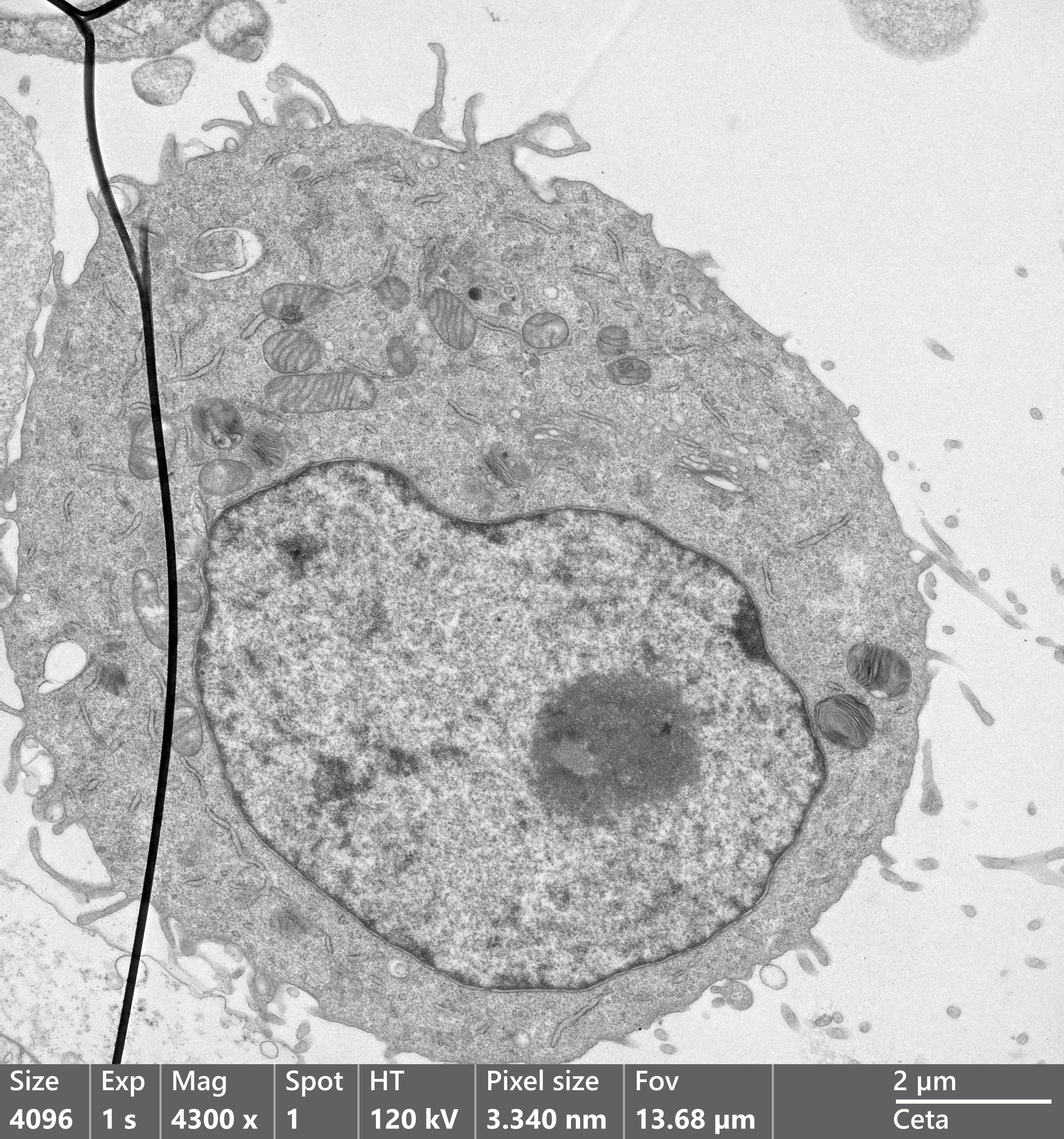

Supplement: Supplementary file 17 — Figure EV7 Source Data [file 44318_2026_816_MOESM17_ESM.zip › B/ECHS1-Rescue.tif]

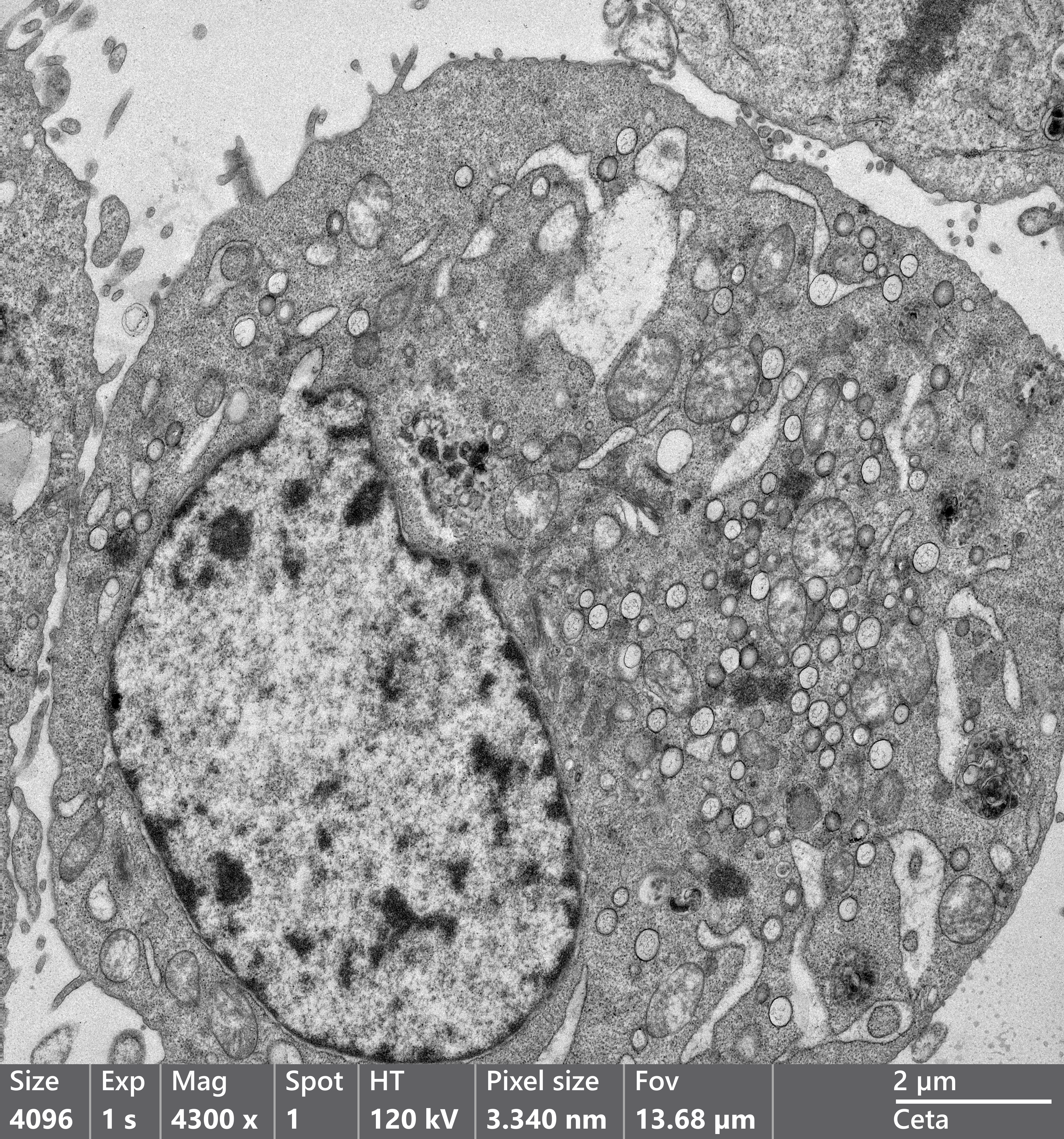

Supplement: Supplementary file 17 — Figure EV7 Source Data [file 44318_2026_816_MOESM17_ESM.zip › C/Ctrl.tif]

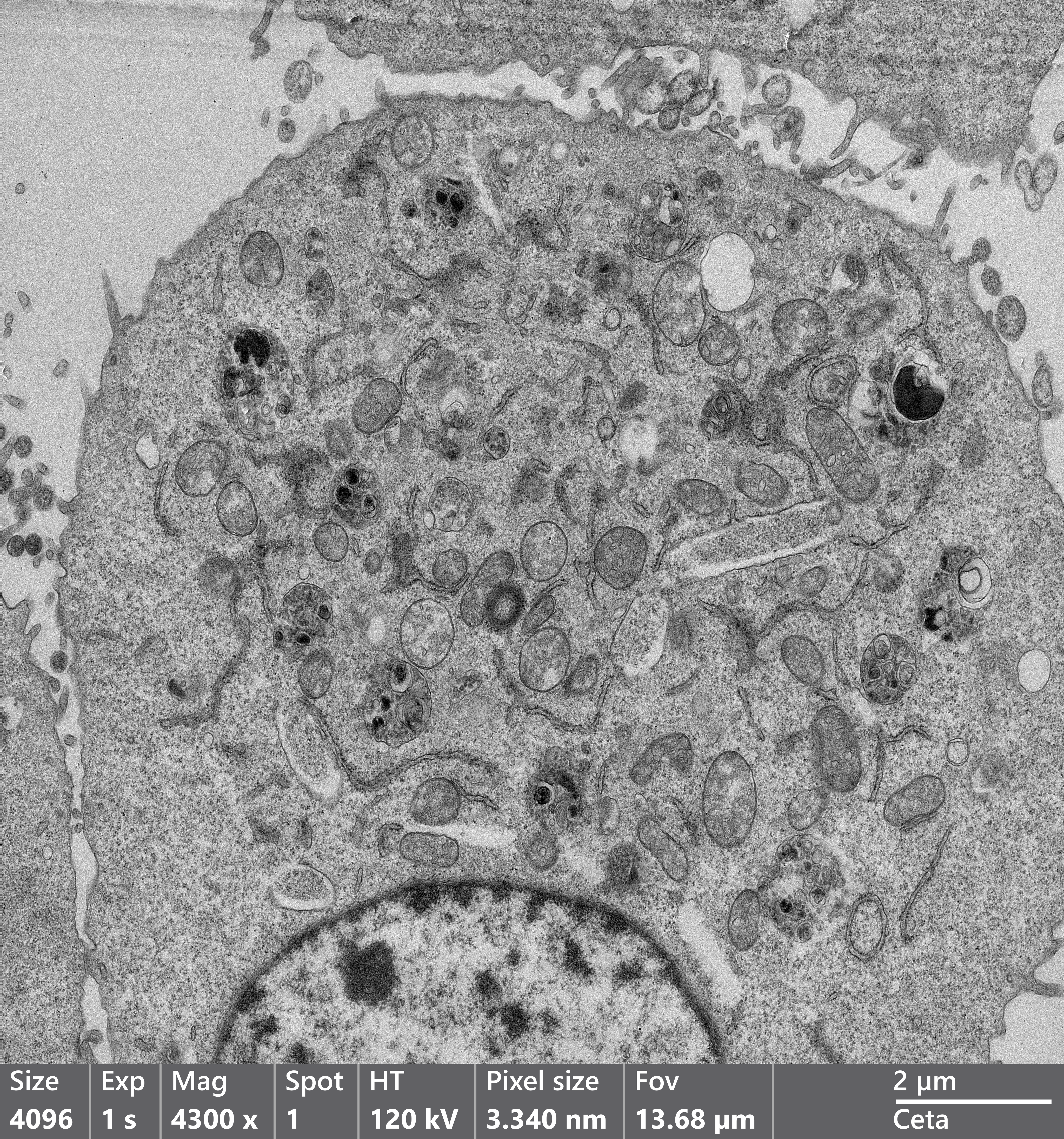

Supplement: Supplementary file 17 — Figure EV7 Source Data [file 44318_2026_816_MOESM17_ESM.zip › C/ECHS1-KO.tif]

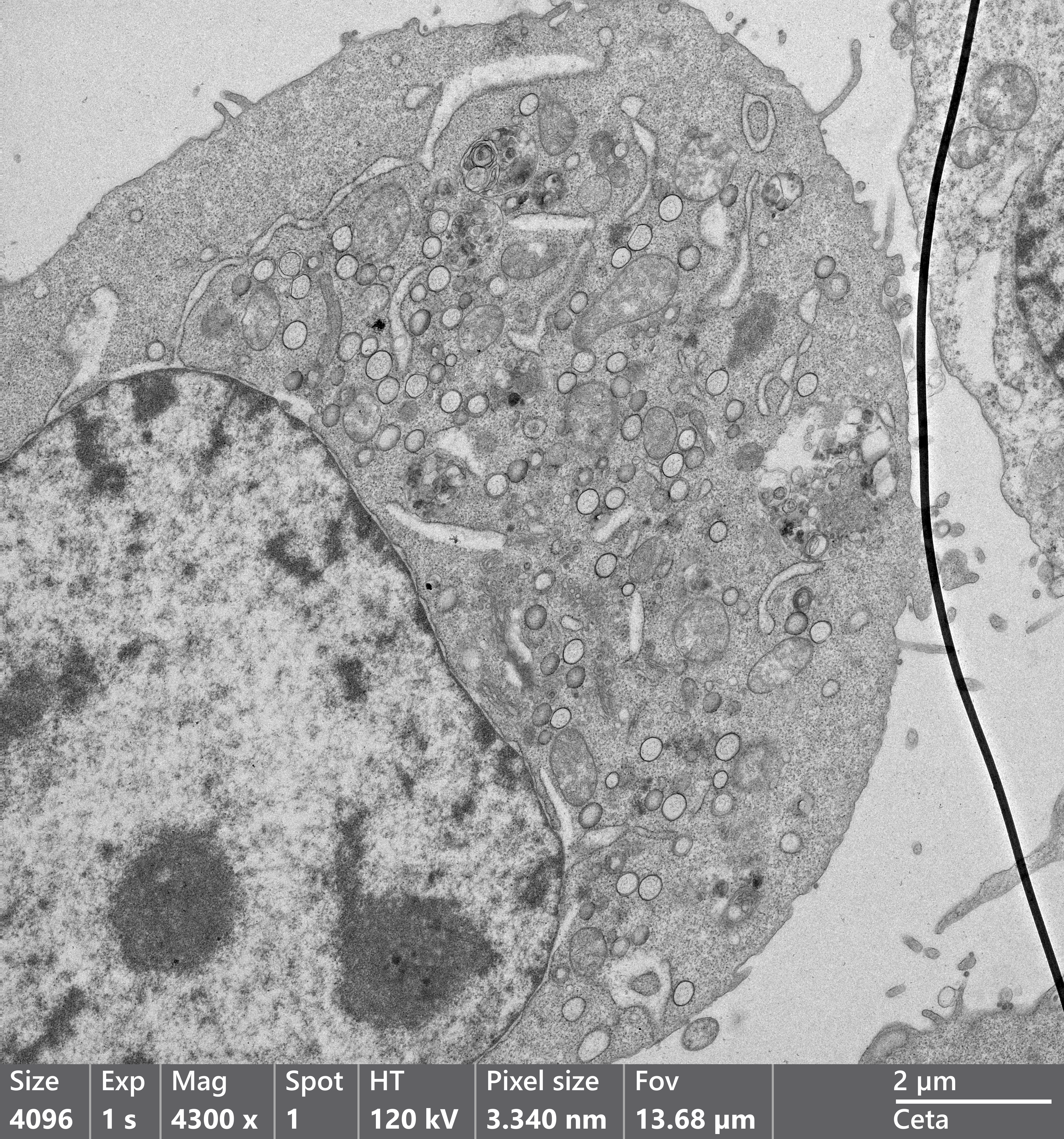

Supplement: Supplementary file 17 — Figure EV7 Source Data [file 44318_2026_816_MOESM17_ESM.zip › C/ECHS1-Rescue.tif]

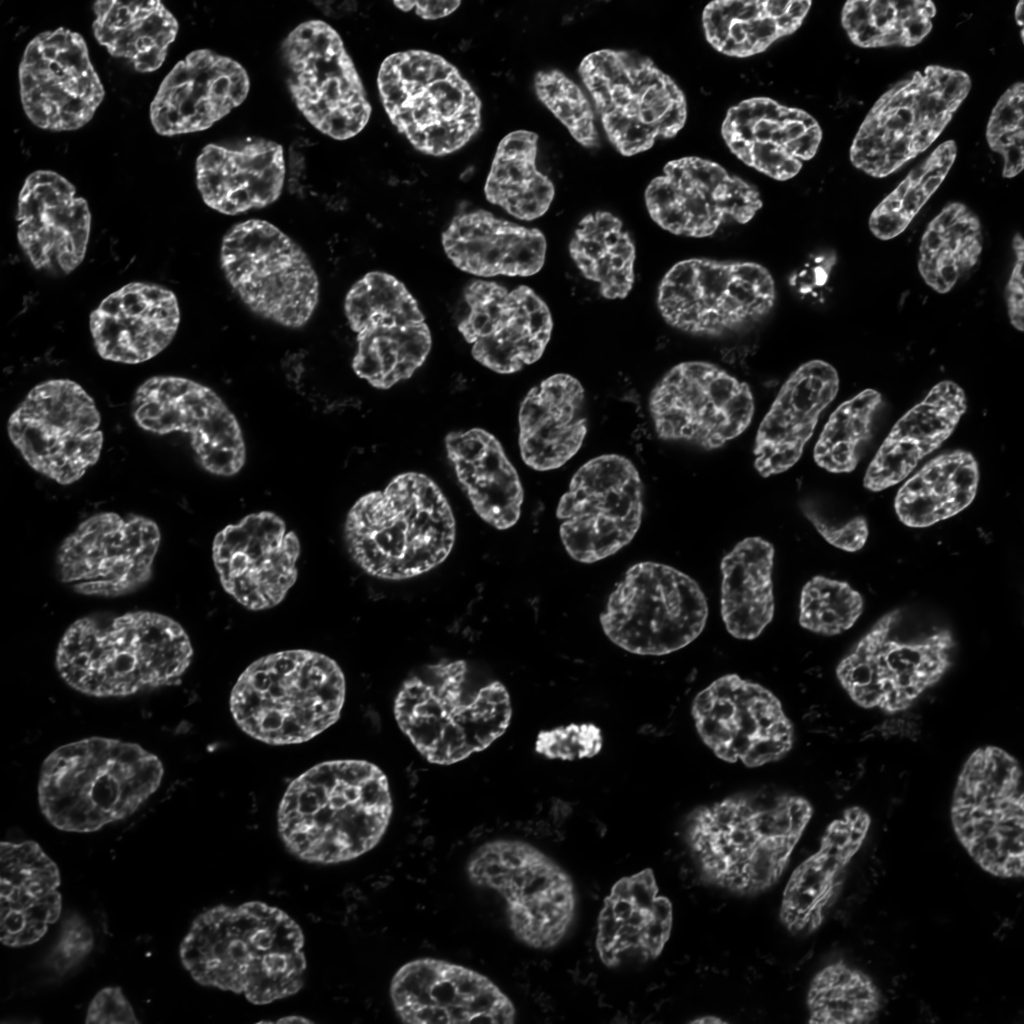

Supplement: Supplementary file 18 — Figure EV8 Source Data [file 44318_2026_816_MOESM18_ESM.zip › A/Ctrl.tif]

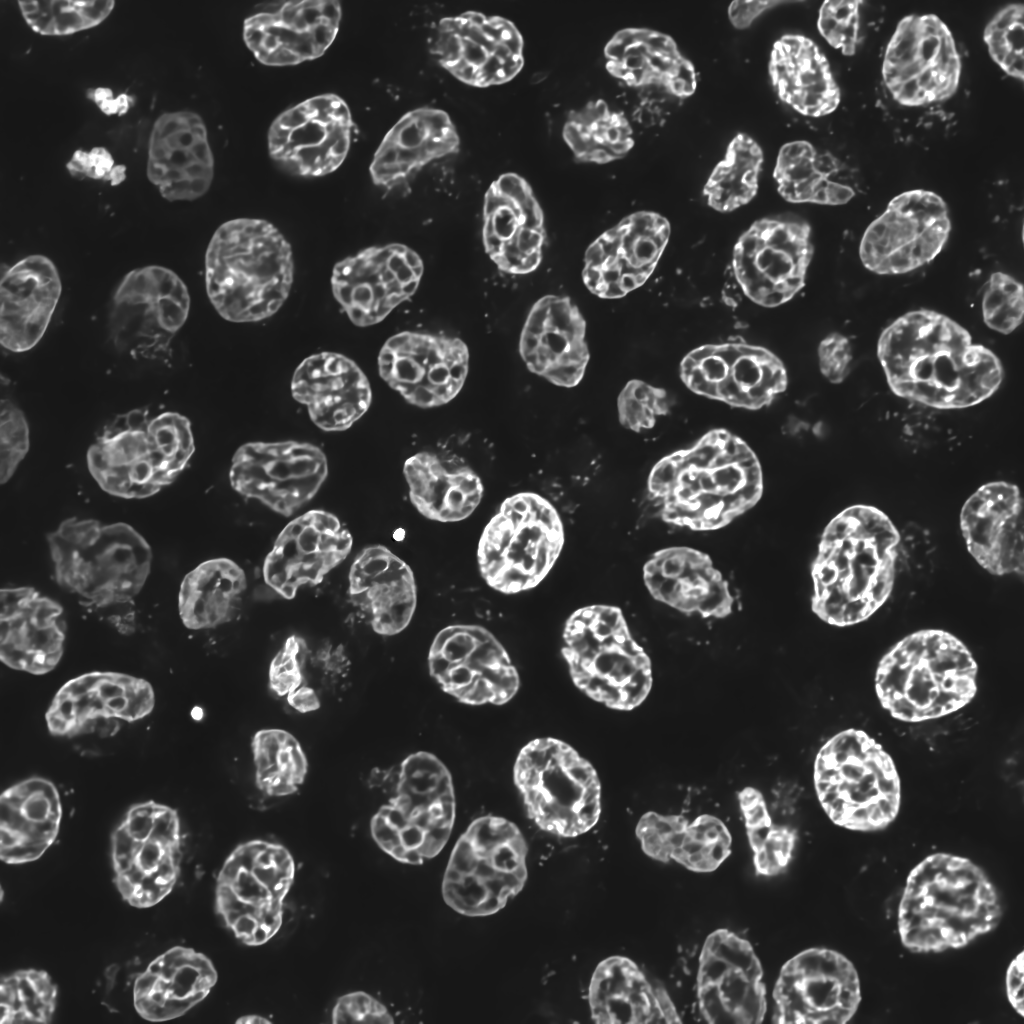

Supplement: Supplementary file 18 — Figure EV8 Source Data [file 44318_2026_816_MOESM18_ESM.zip › A/ECHS1-KO.tif]

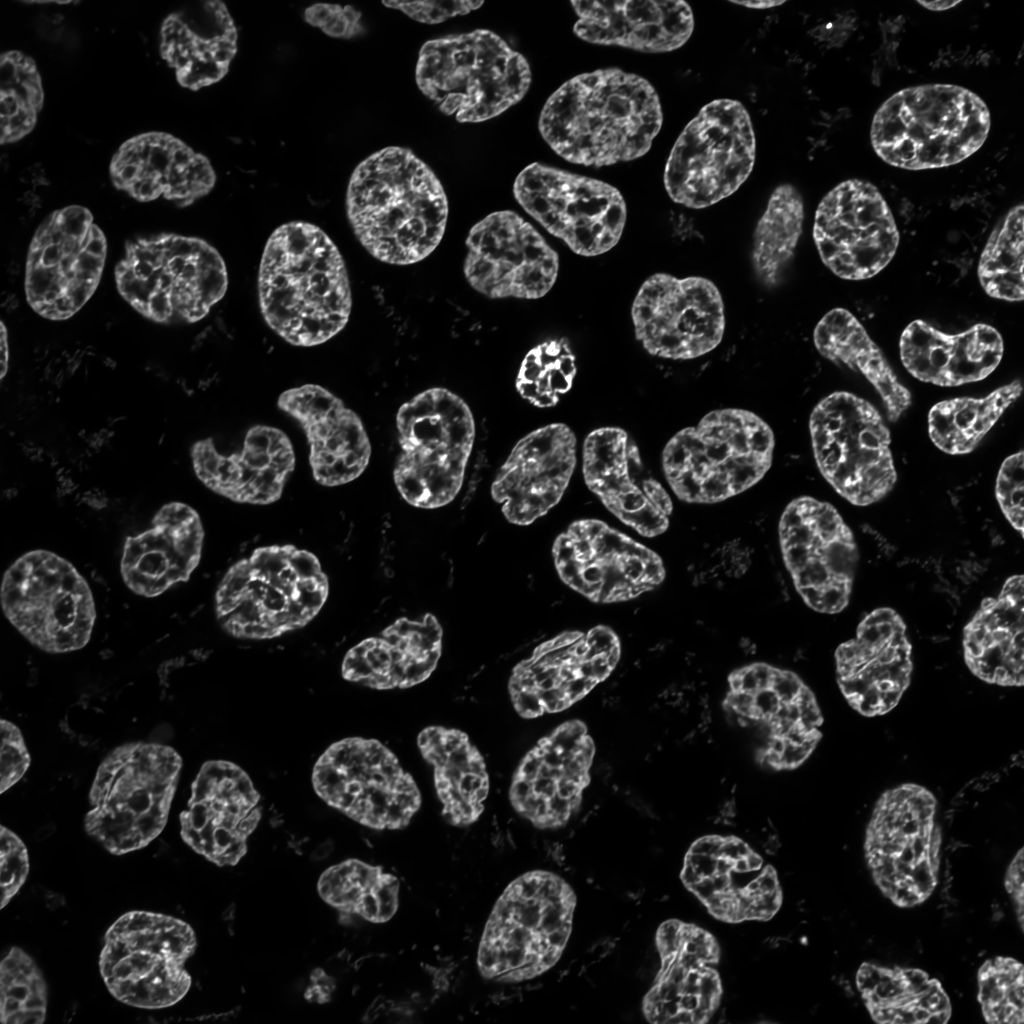

Supplement: Supplementary file 18 — Figure EV8 Source Data [file 44318_2026_816_MOESM18_ESM.zip › A/ECHS1-Rescue.tif]

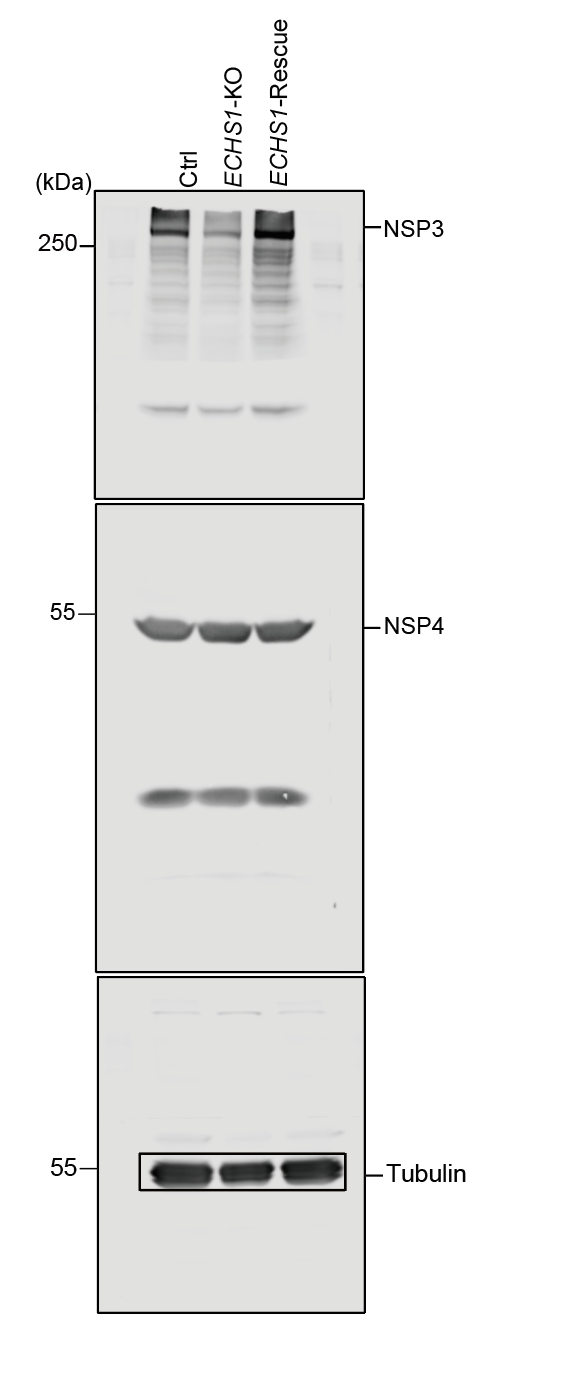

Supplement: Supplementary file 18 — Figure EV8 Source Data [file 44318_2026_816_MOESM18_ESM.zip › B/NSP4+NSP3+Tubulin.tif]

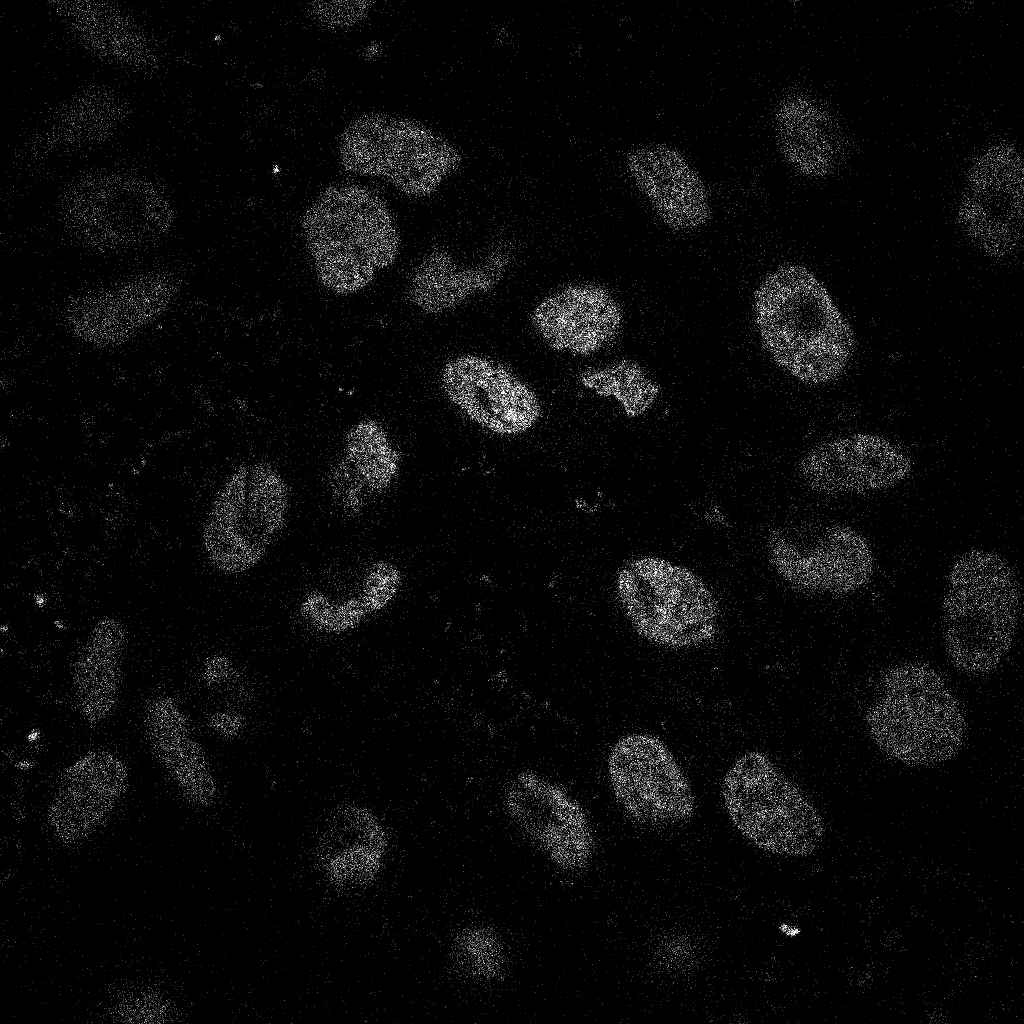

Supplement: Supplementary file 18 — Figure EV8 Source Data [file 44318_2026_816_MOESM18_ESM.zip › C/Ctrl.tif]

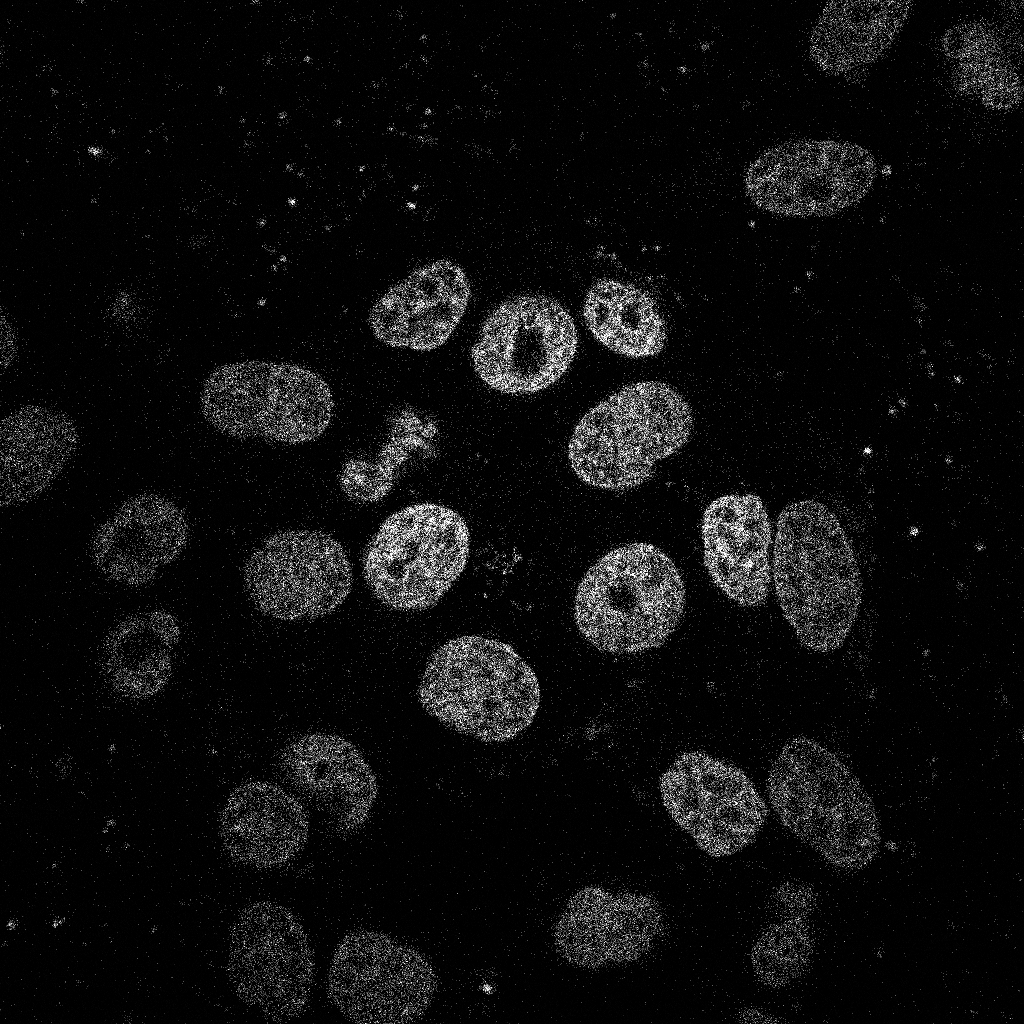

Supplement: Supplementary file 18 — Figure EV8 Source Data [file 44318_2026_816_MOESM18_ESM.zip › C/ECHS1-KO.tif]

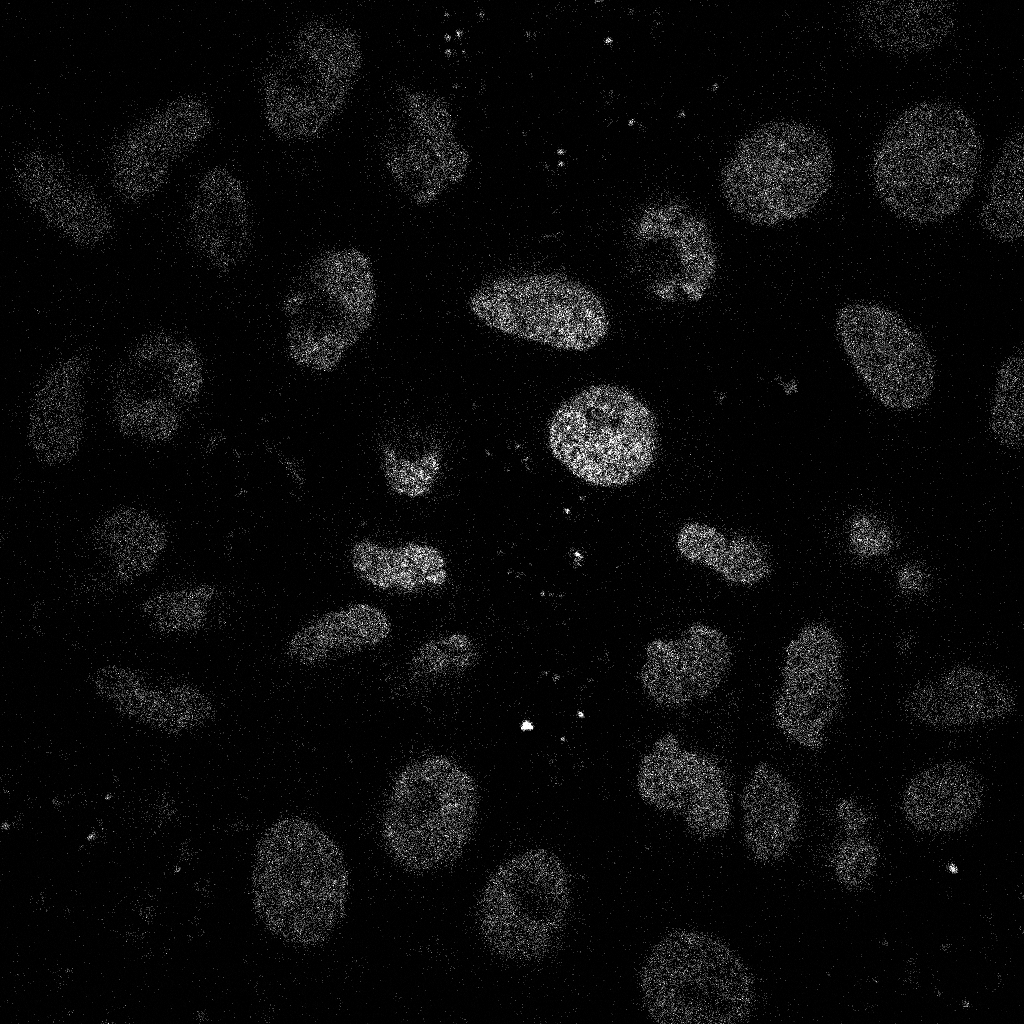

Supplement: Supplementary file 18 — Figure EV8 Source Data [file 44318_2026_816_MOESM18_ESM.zip › C/ECHS1-Rescue.tif]

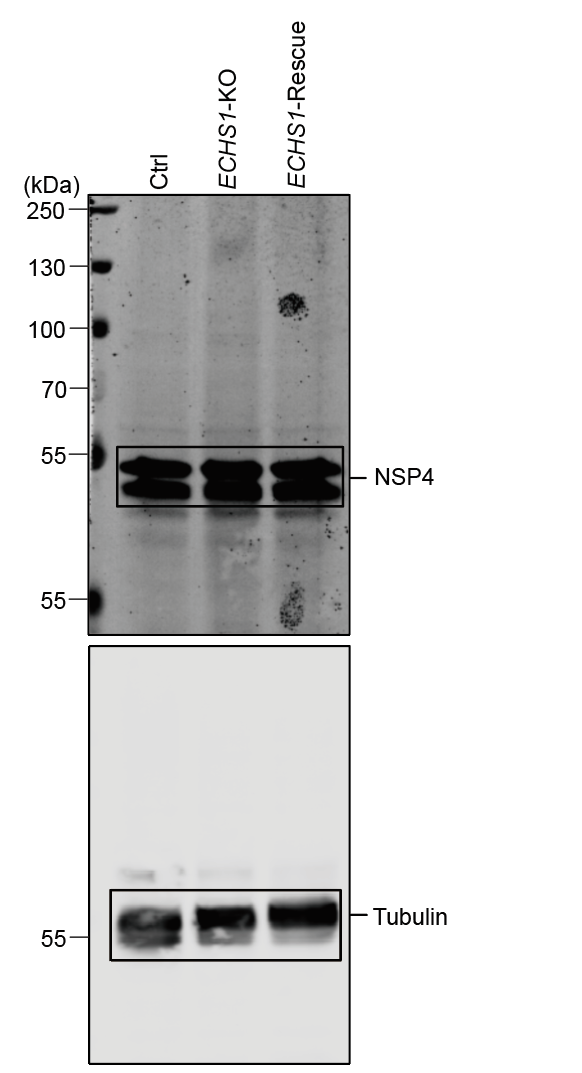

Supplement: Supplementary file 18 — Figure EV8 Source Data [file 44318_2026_816_MOESM18_ESM.zip › D/NSP4+Tubulin.tif]

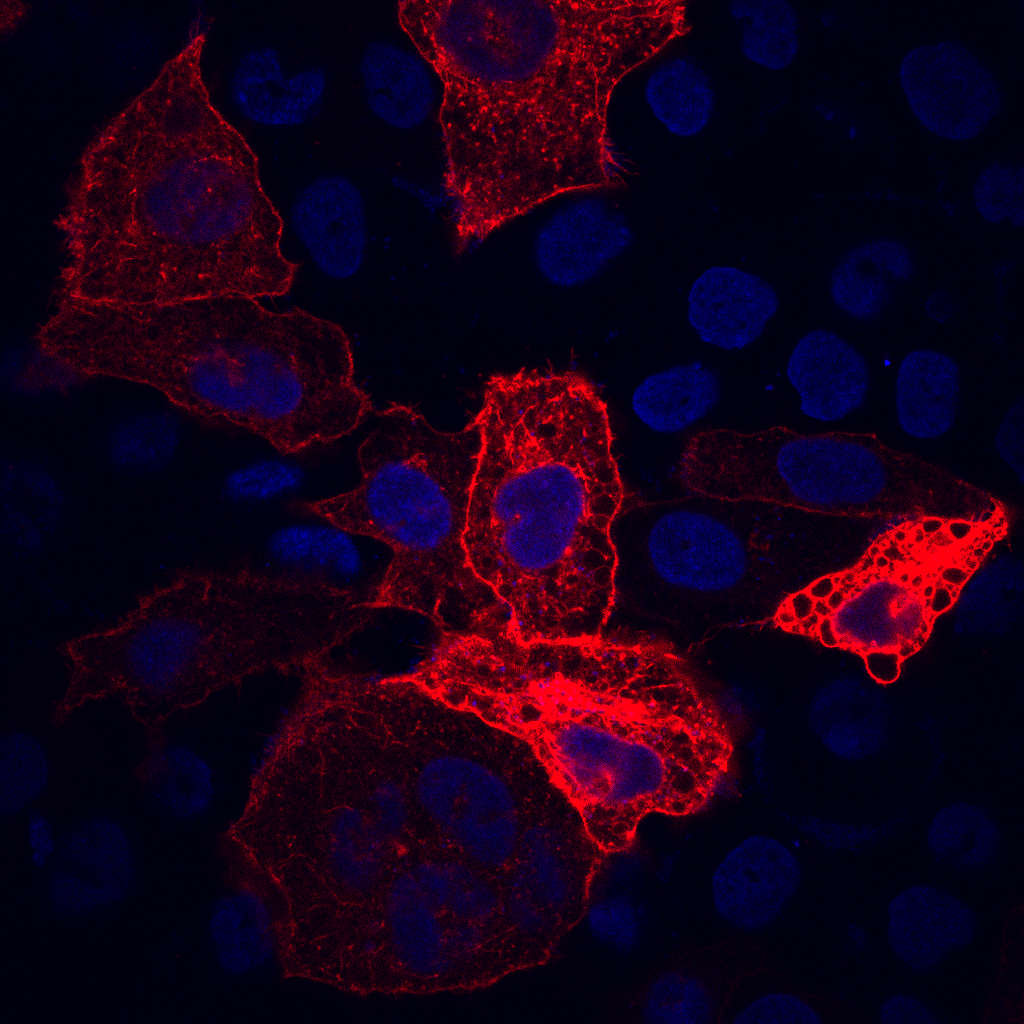

Supplement: Supplementary file 18 — Figure EV8 Source Data [file 44318_2026_816_MOESM18_ESM.zip › E/Ctrl-M.tif]

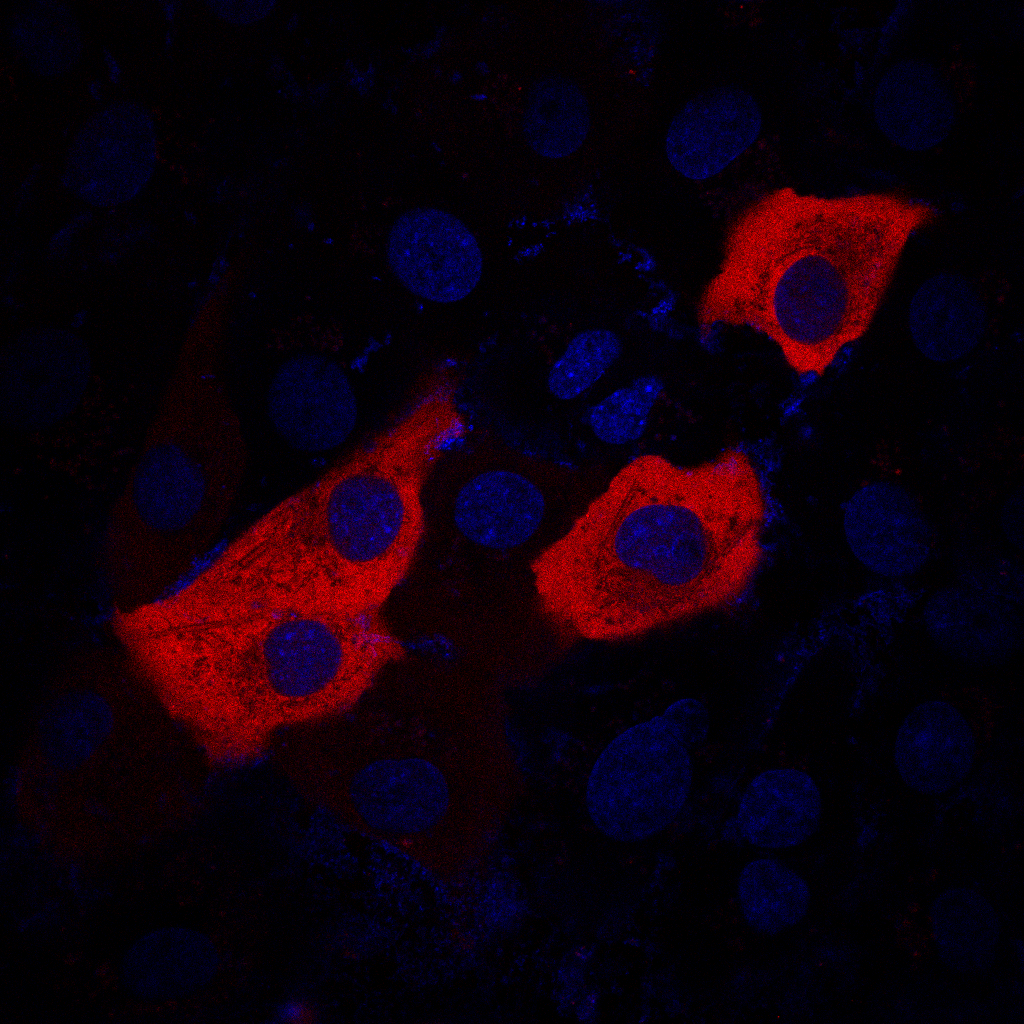

Supplement: Supplementary file 18 — Figure EV8 Source Data [file 44318_2026_816_MOESM18_ESM.zip › E/Ctrl-N.tif]

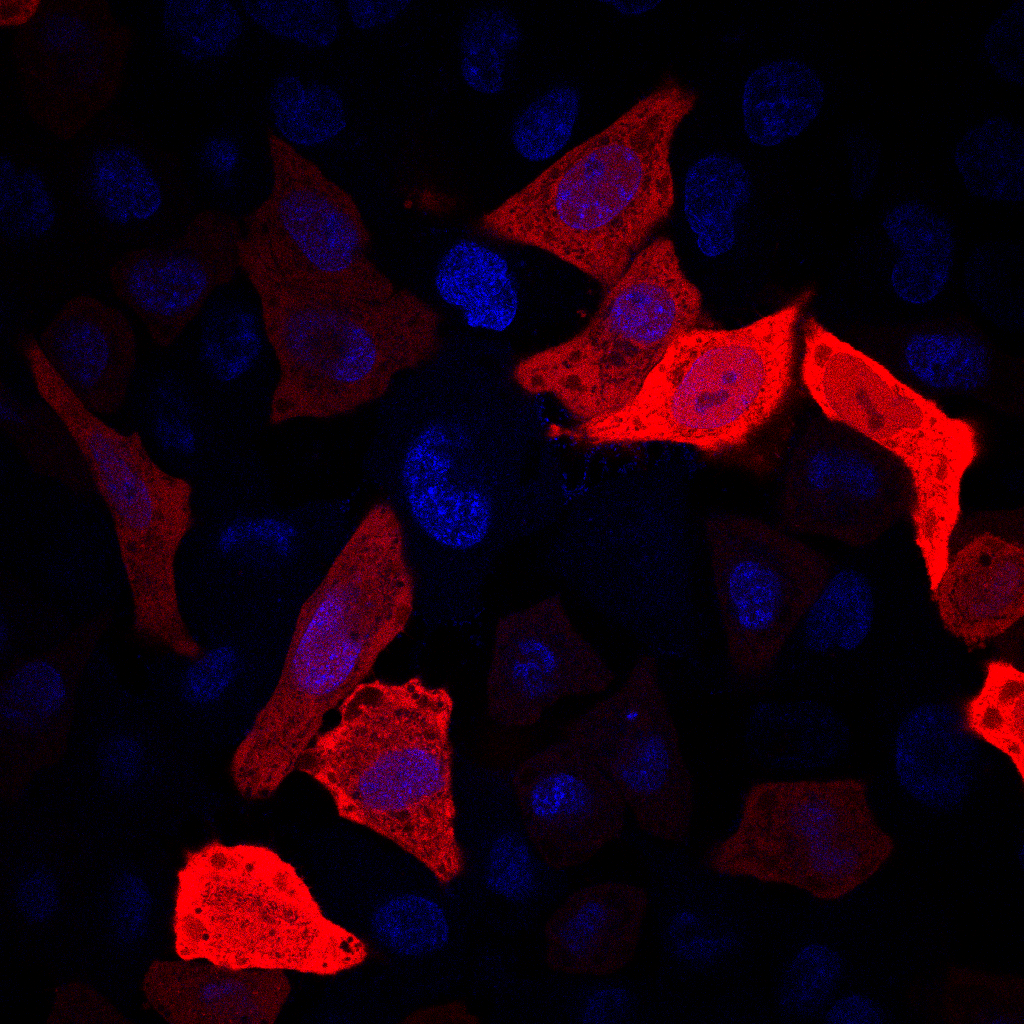

Supplement: Supplementary file 18 — Figure EV8 Source Data [file 44318_2026_816_MOESM18_ESM.zip › E/Ctrl-NSP8.tif]

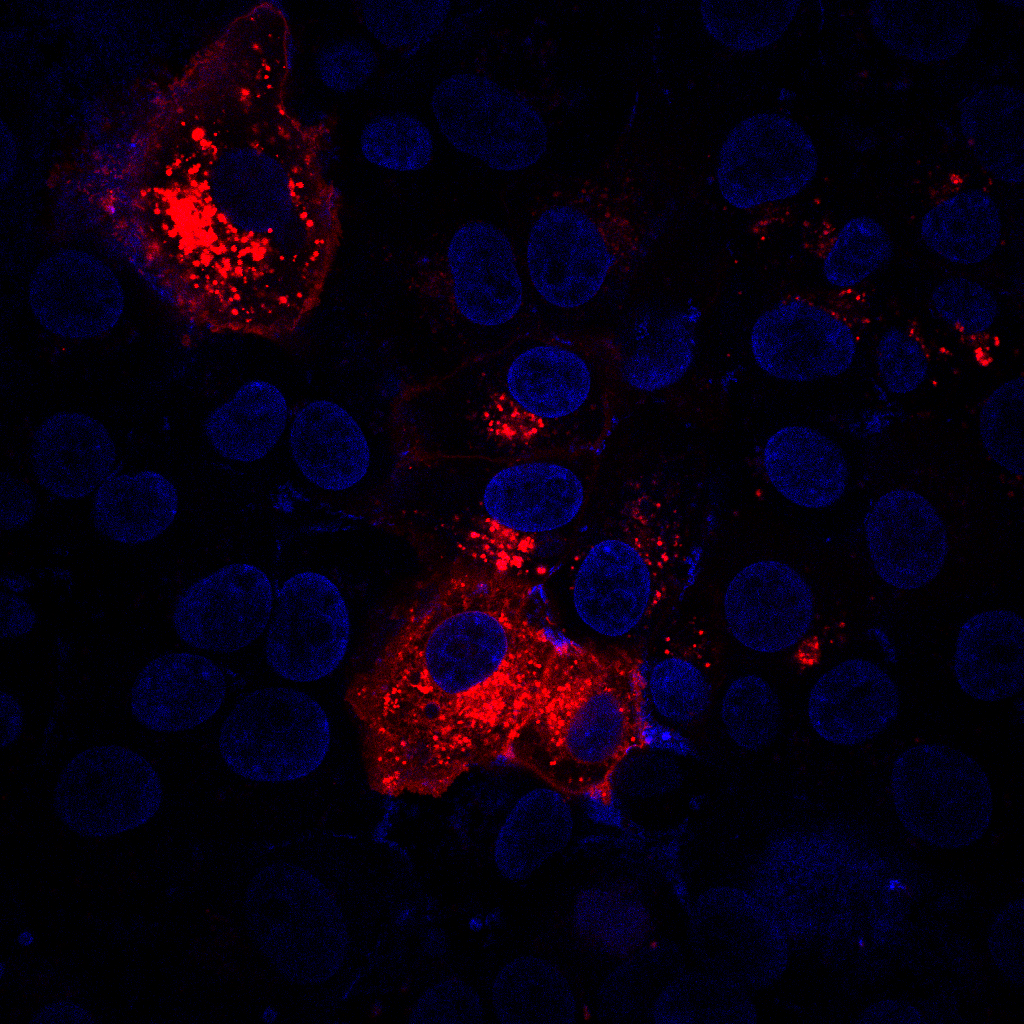

Supplement: Supplementary file 18 — Figure EV8 Source Data [file 44318_2026_816_MOESM18_ESM.zip › E/Ctrl-ORF3a.tif]

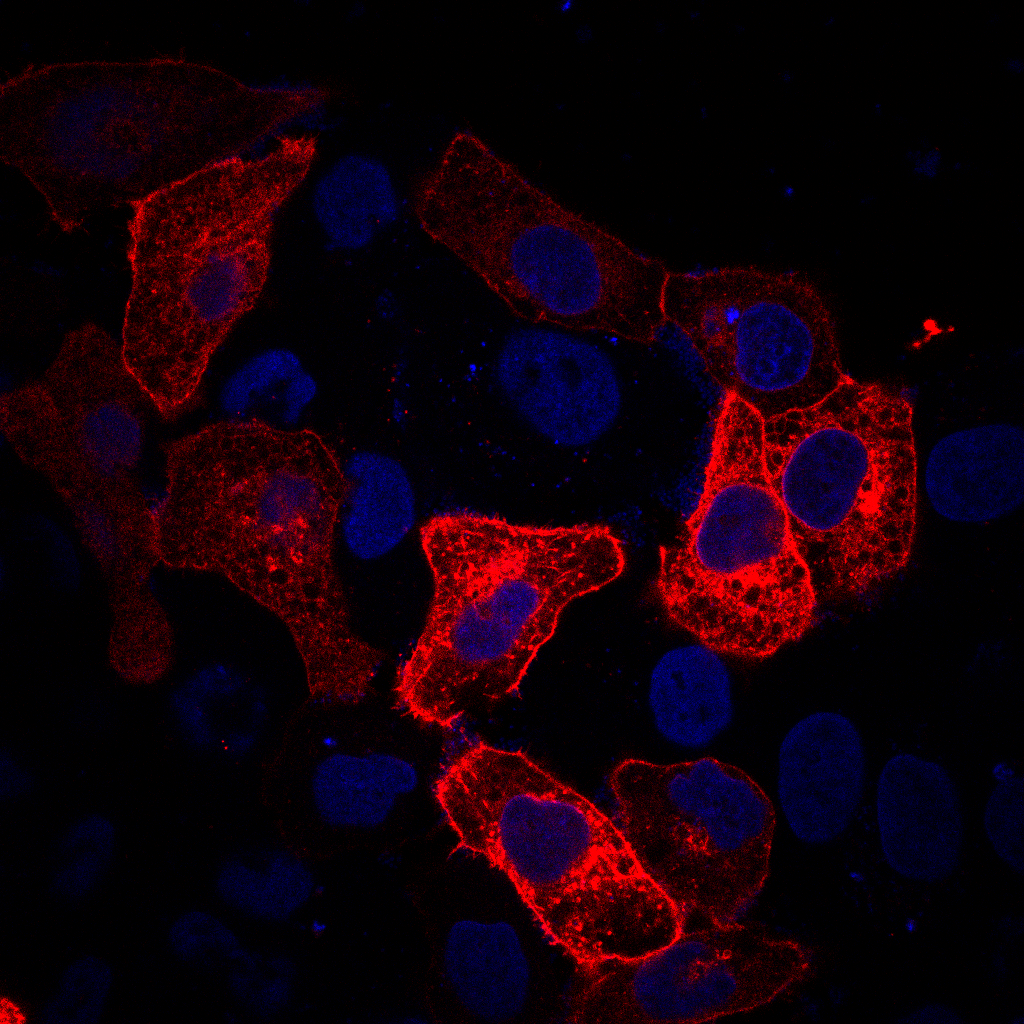

Supplement: Supplementary file 18 — Figure EV8 Source Data [file 44318_2026_816_MOESM18_ESM.zip › E/ECHS1-KO-M.tif]

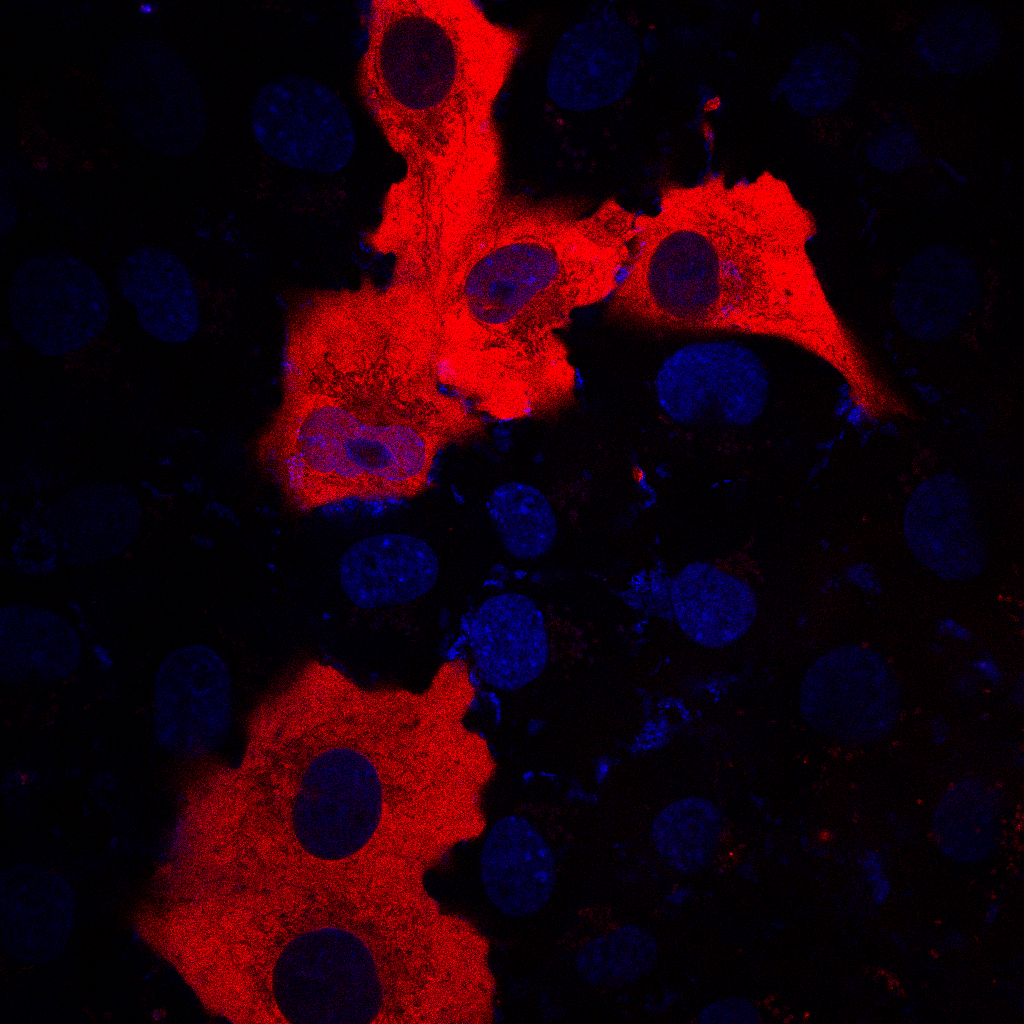

Supplement: Supplementary file 18 — Figure EV8 Source Data [file 44318_2026_816_MOESM18_ESM.zip › E/ECHS1-KO-N.tif]

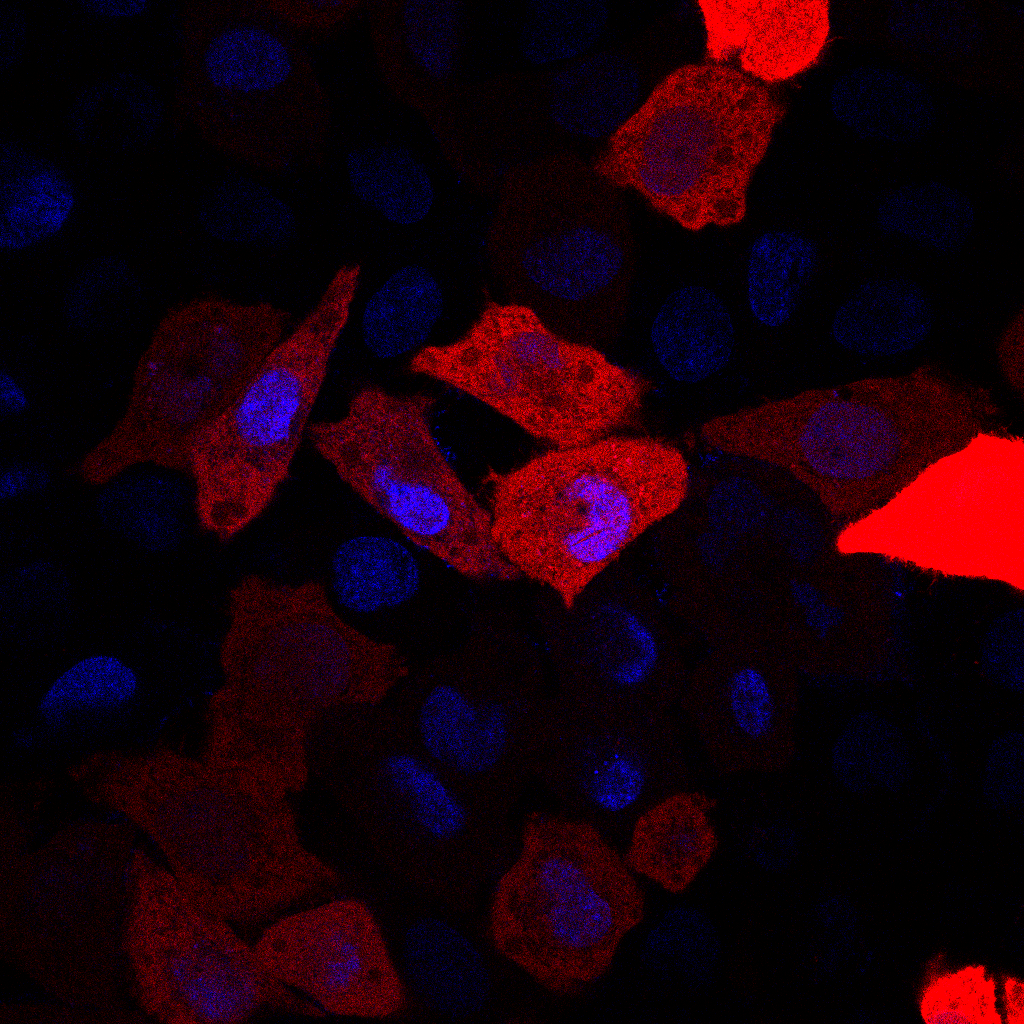

Supplement: Supplementary file 18 — Figure EV8 Source Data [file 44318_2026_816_MOESM18_ESM.zip › E/ECHS1-KO-NSP8.tif]

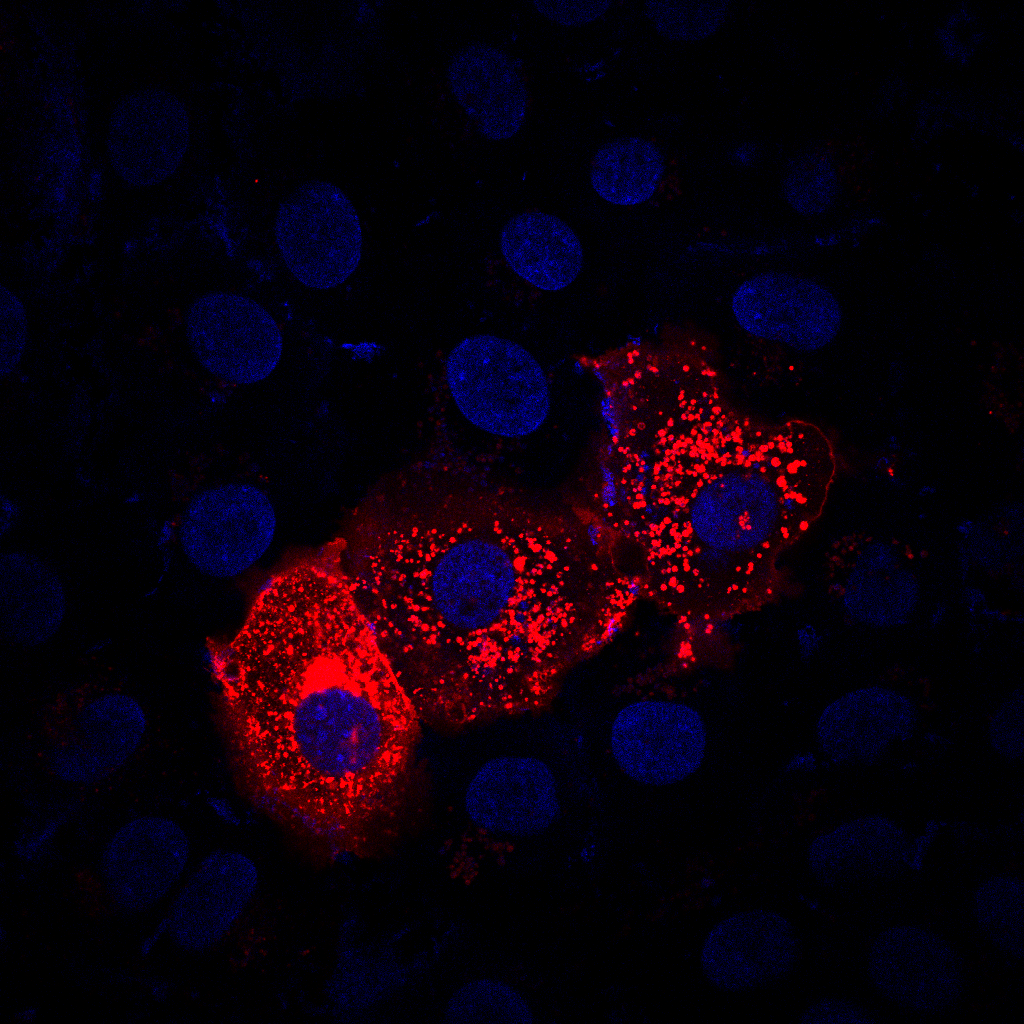

Supplement: Supplementary file 18 — Figure EV8 Source Data [file 44318_2026_816_MOESM18_ESM.zip › E/ECHS1-KO-ORF3a.tif]

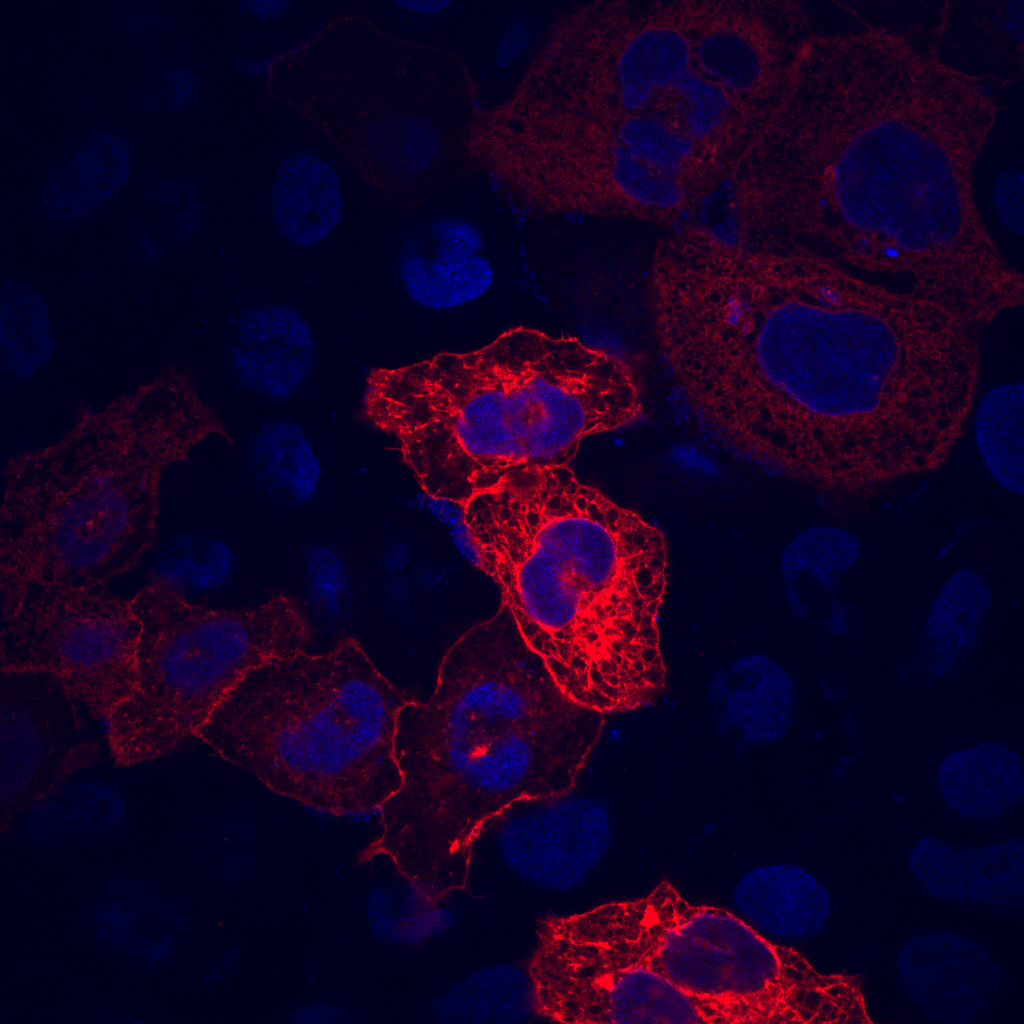

Supplement: Supplementary file 18 — Figure EV8 Source Data [file 44318_2026_816_MOESM18_ESM.zip › E/ECHS1-Rescue-M.tif]

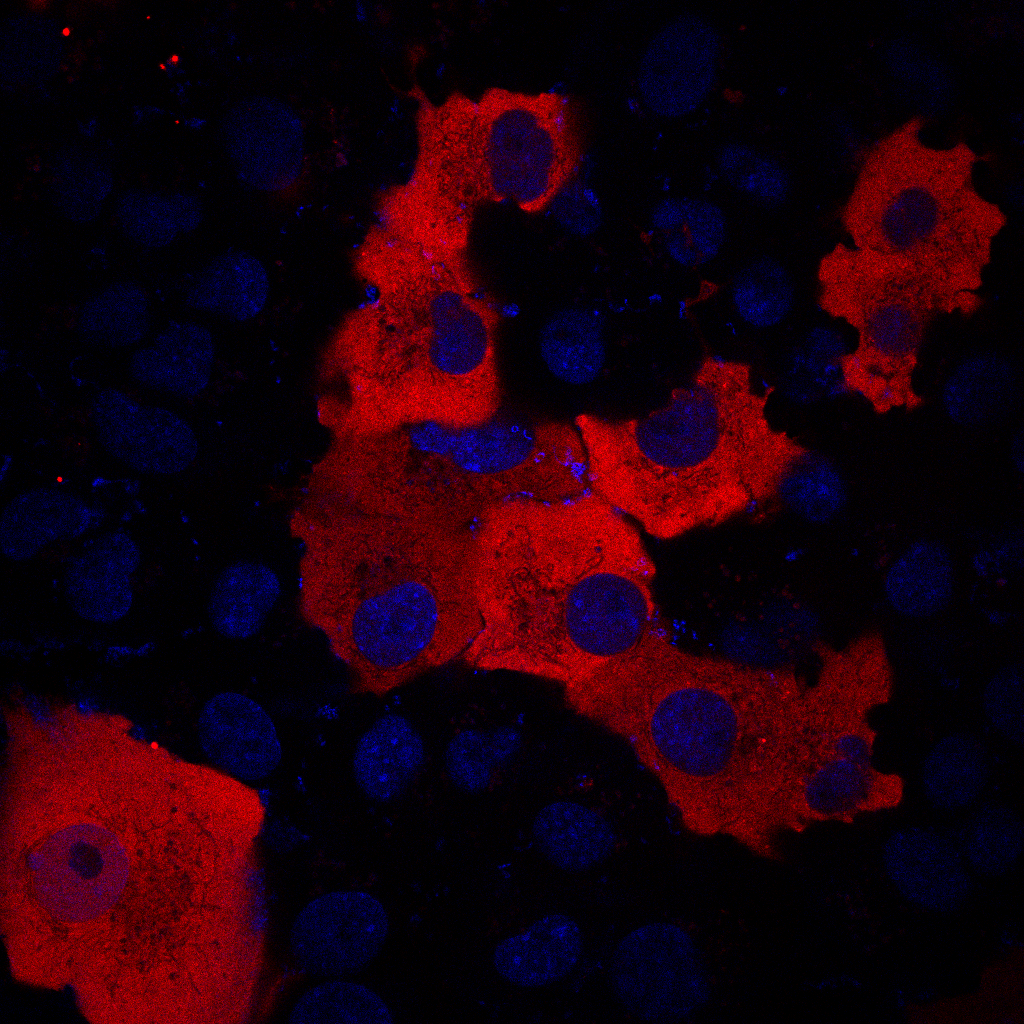

Supplement: Supplementary file 18 — Figure EV8 Source Data [file 44318_2026_816_MOESM18_ESM.zip › E/ECHS1-Rescue-N.tif]

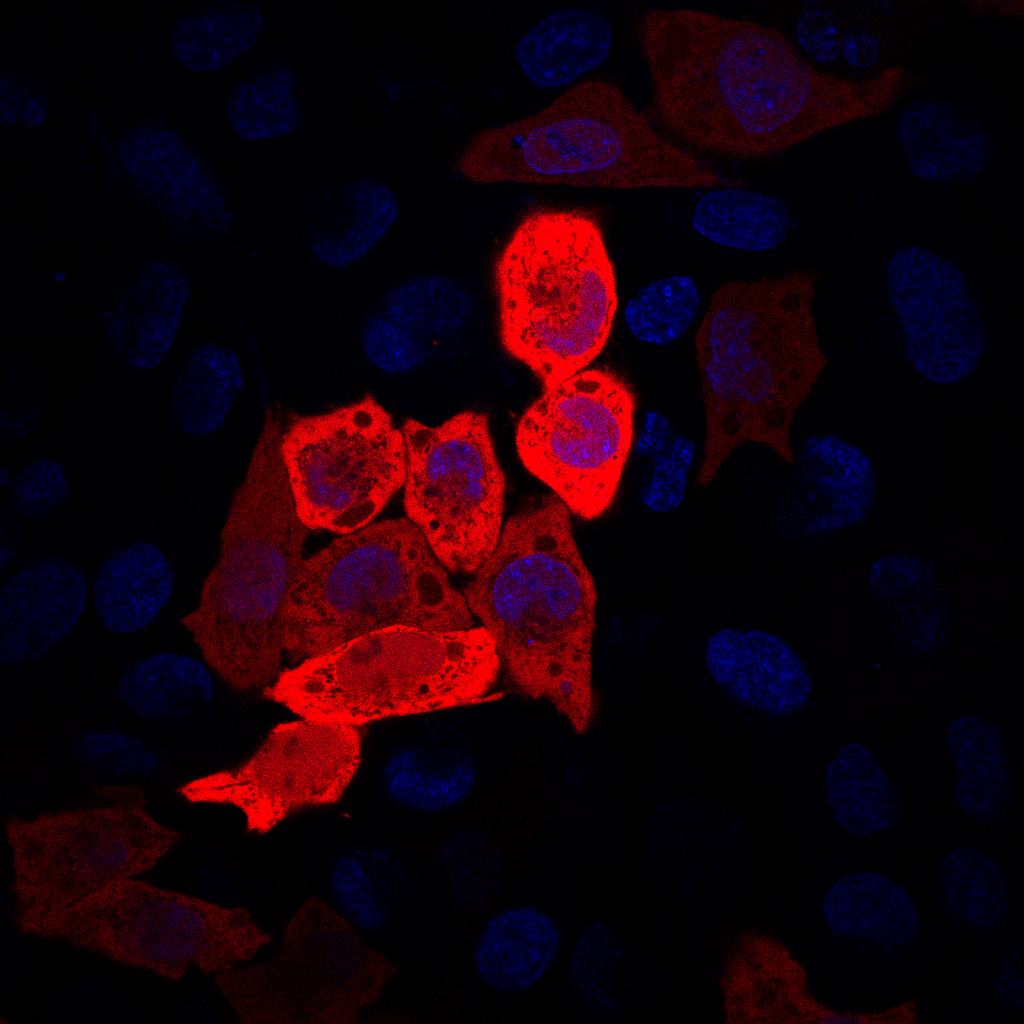

Supplement: Supplementary file 18 — Figure EV8 Source Data [file 44318_2026_816_MOESM18_ESM.zip › E/ECHS1-Rescue-NSP8.tif]

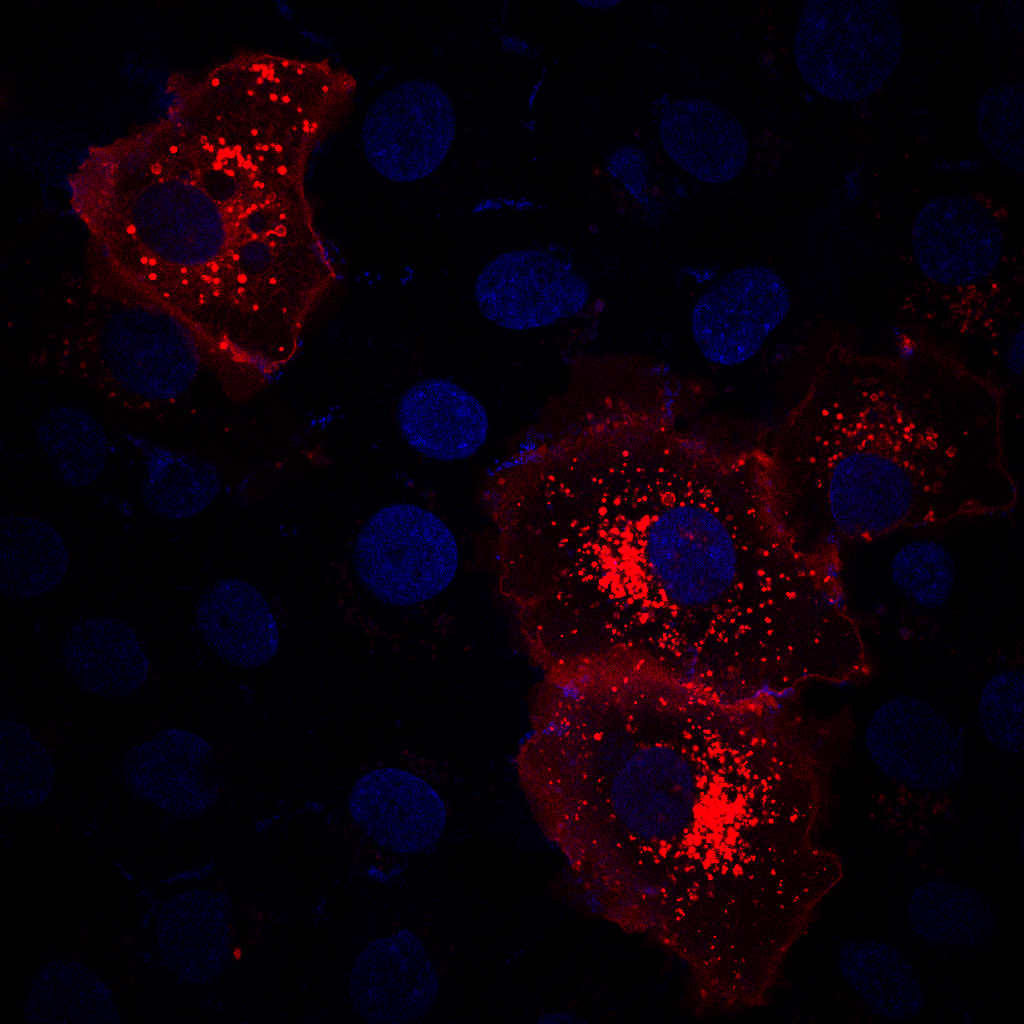

Supplement: Supplementary file 18 — Figure EV8 Source Data [file 44318_2026_816_MOESM18_ESM.zip › E/ECHS1-Rescue-ORF3a.tif]

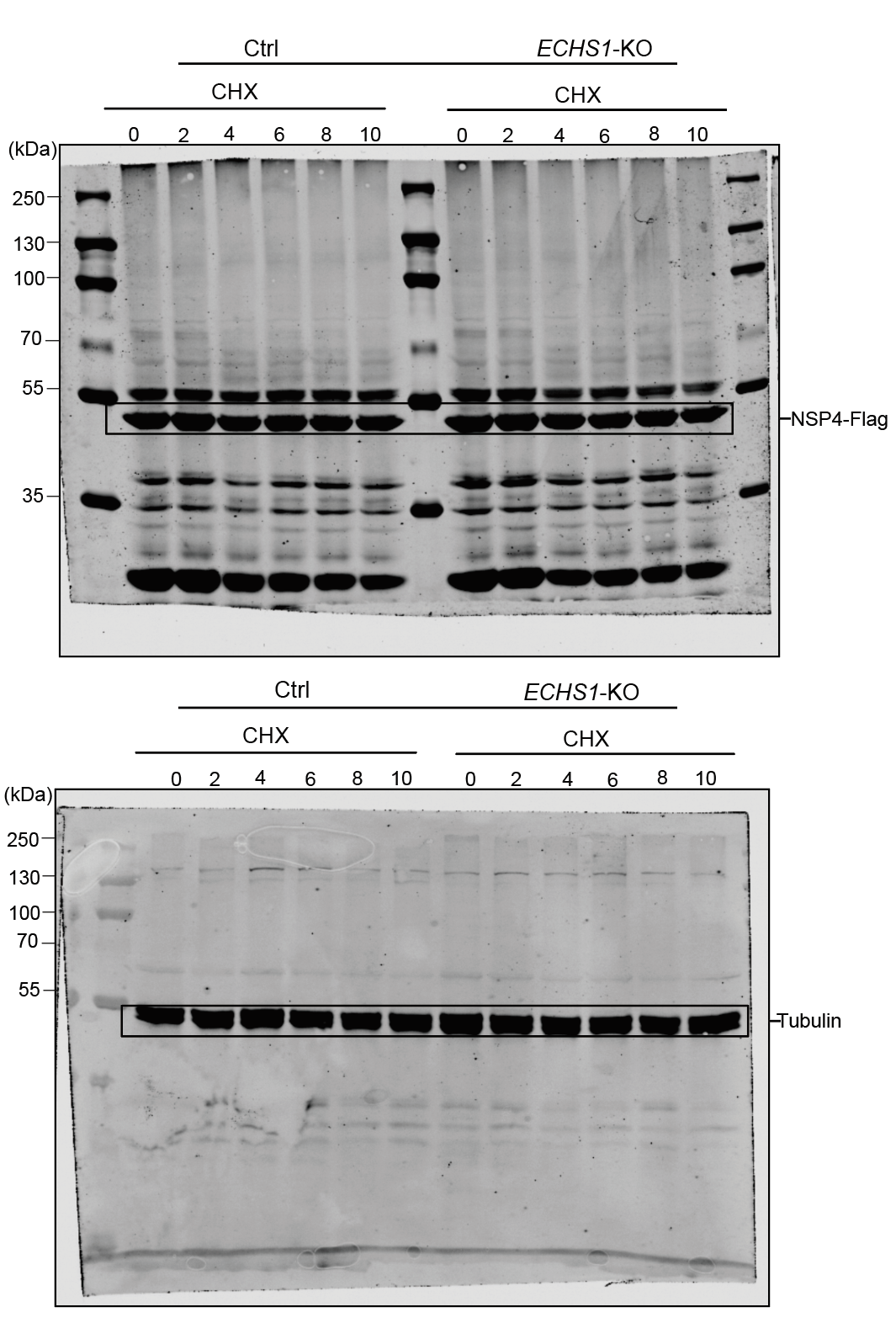

Supplement: Supplementary file 18 — Figure EV8 Source Data [file 44318_2026_816_MOESM18_ESM.zip › F/NSP4+Flag+Tubulin.tif]

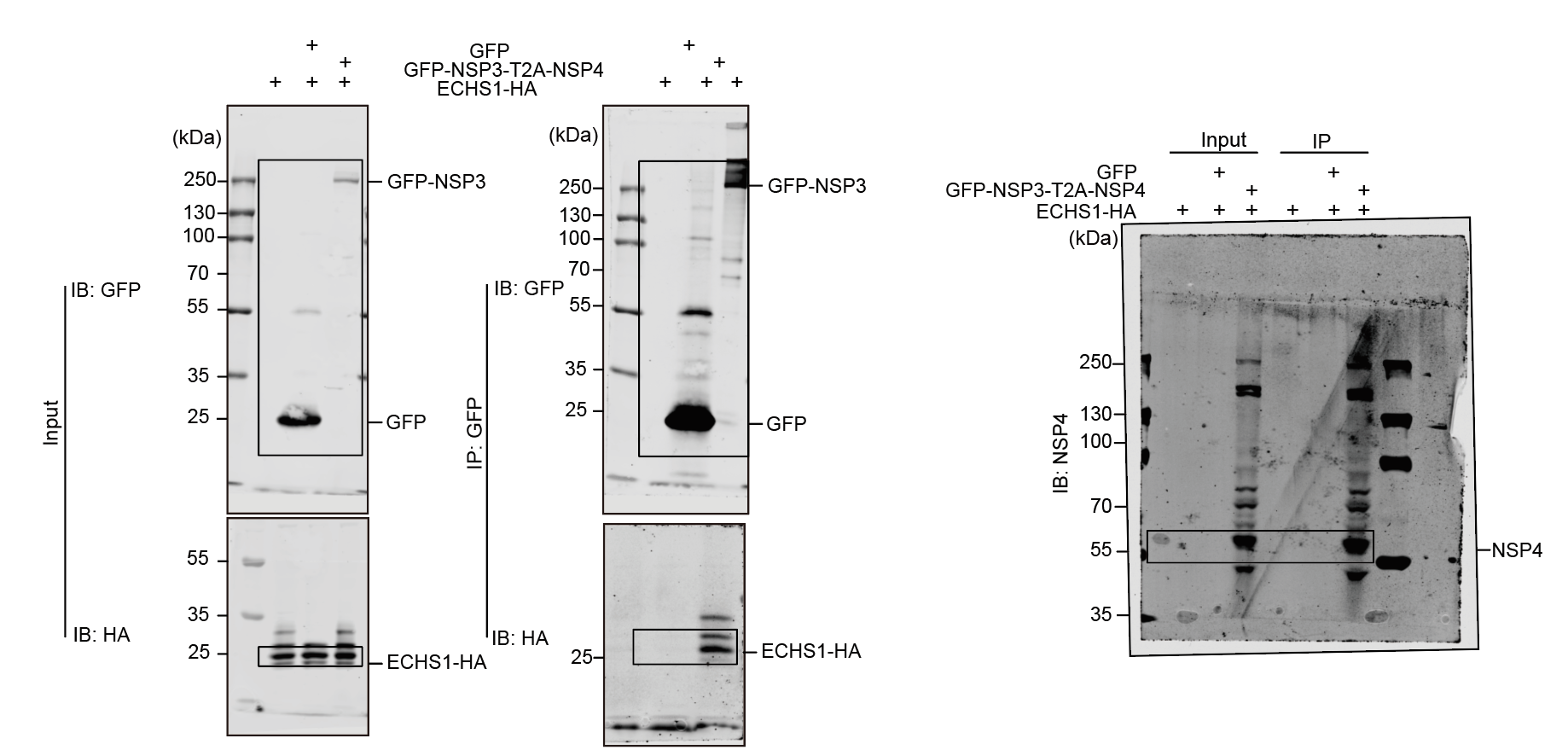

Supplement: Supplementary file 19 — Figure EV9 Source Data [file 44318_2026_816_MOESM19_ESM.zip › A/GFP-NSP3+GFP+ECHS1-HA+NSP4.tif]

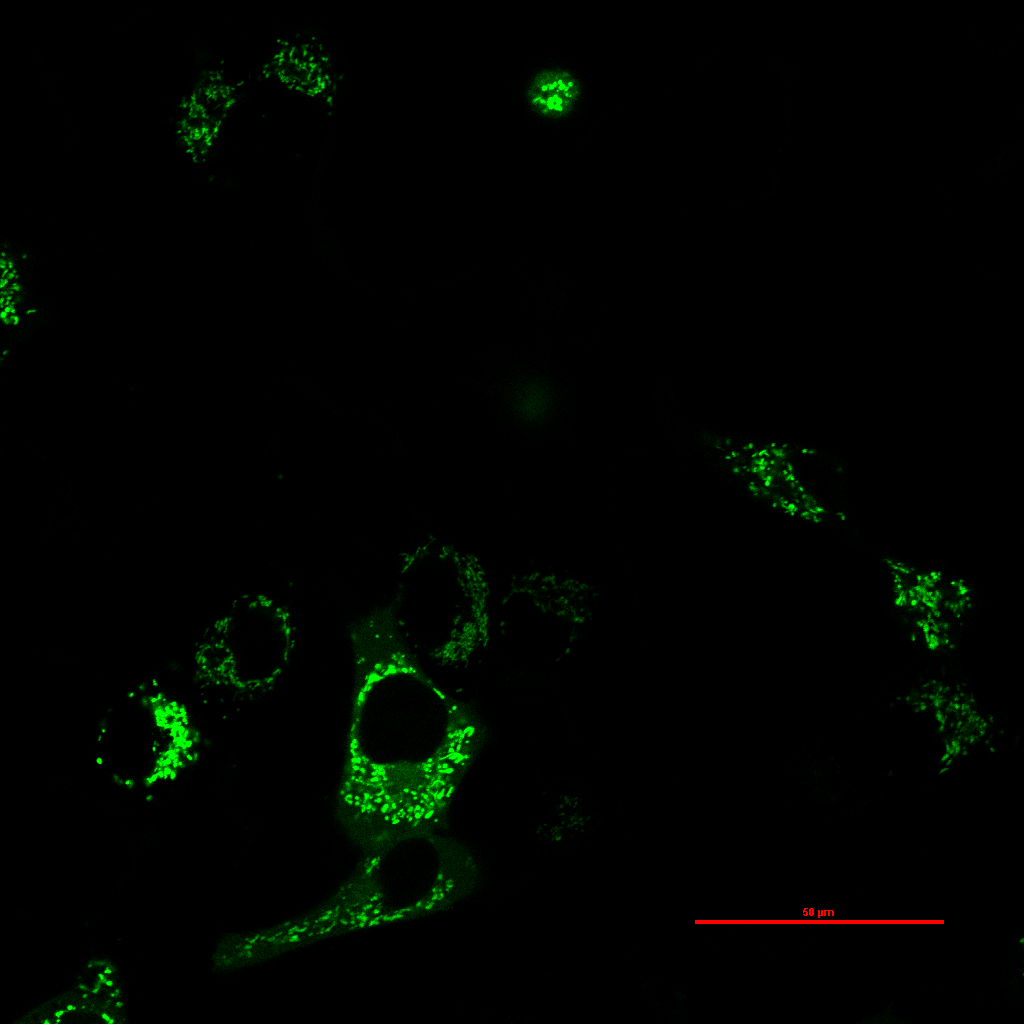

Supplement: Supplementary file 19 — Figure EV9 Source Data [file 44318_2026_816_MOESM19_ESM.zip › B/ECHS1 FL+Mitotracker_EGFP.tif]

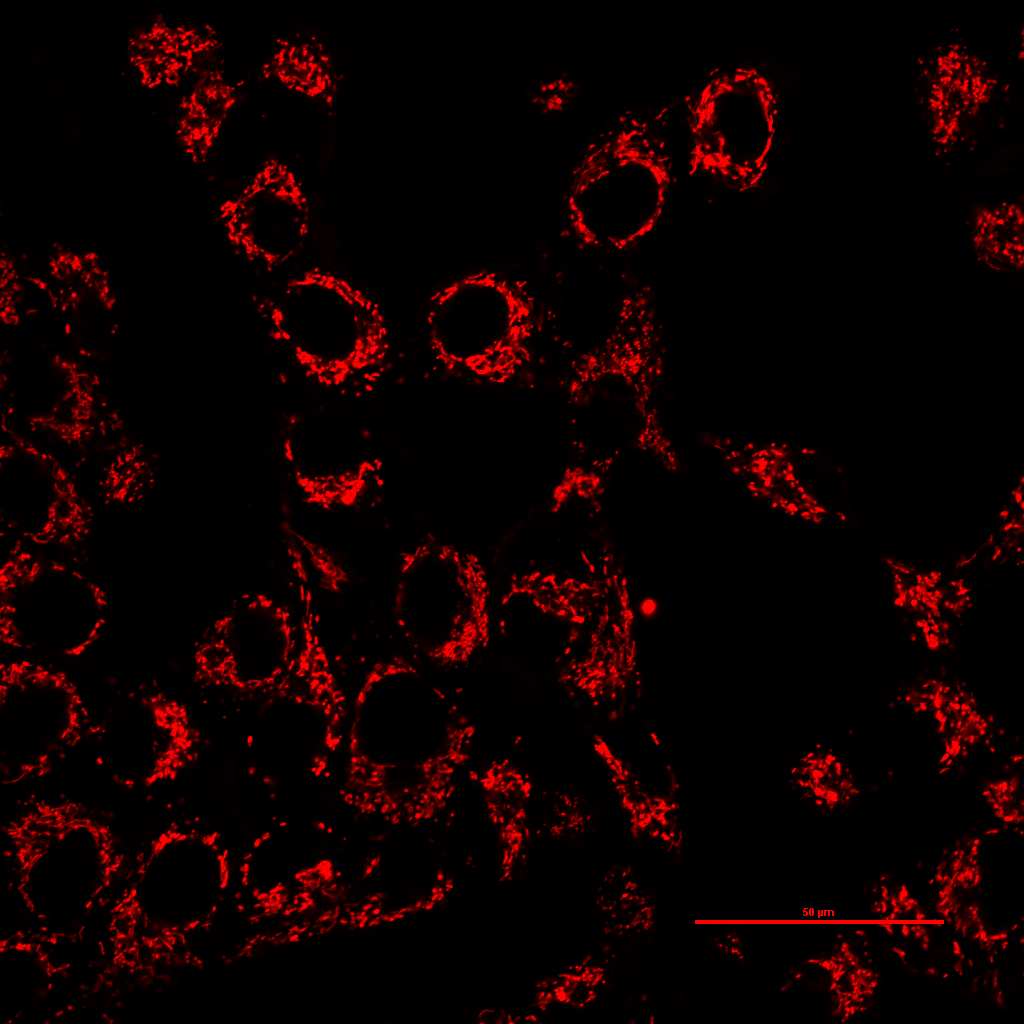

Supplement: Supplementary file 19 — Figure EV9 Source Data [file 44318_2026_816_MOESM19_ESM.zip › B/ECHS1 FL+Mitotracker_mCherry.tif]

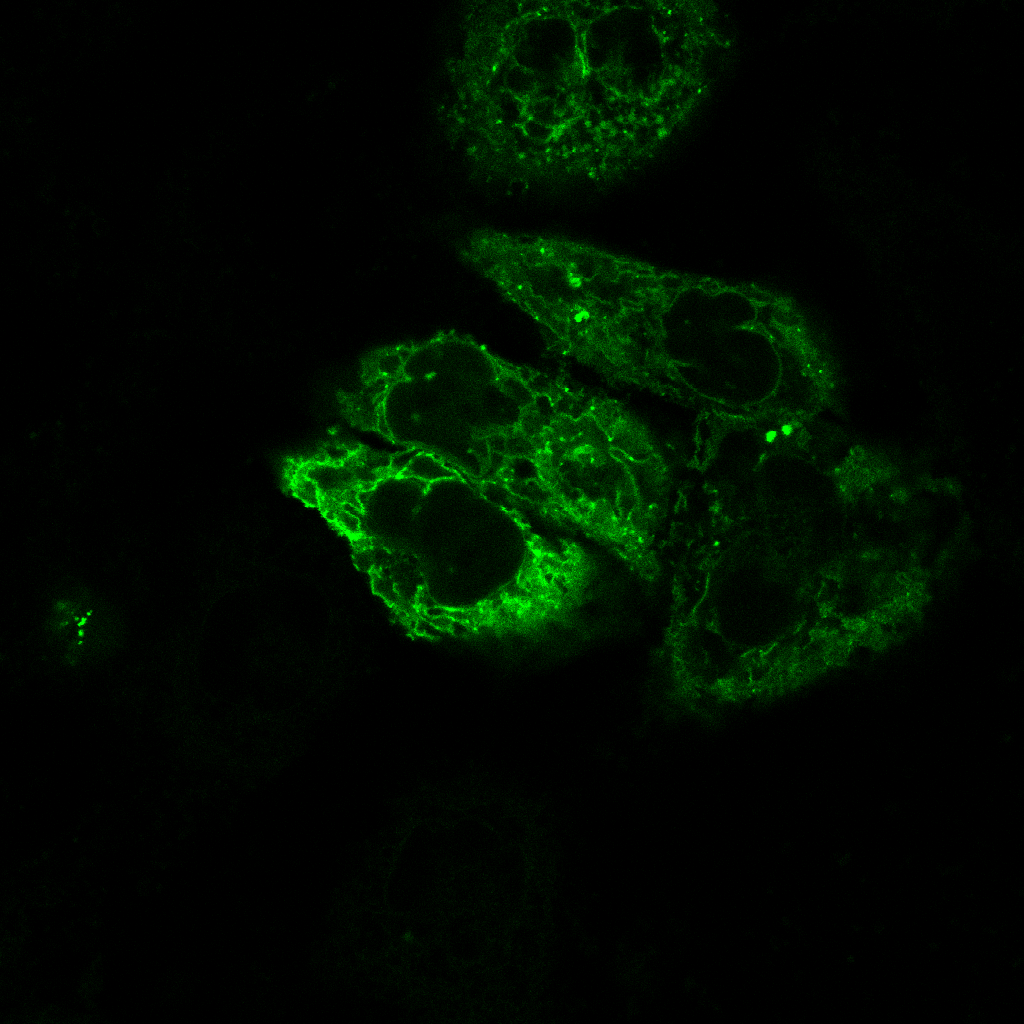

Supplement: Supplementary file 19 — Figure EV9 Source Data [file 44318_2026_816_MOESM19_ESM.zip › B/ECHS1-ΔMTS-Mcherry+GFP-NSP4_EGFP.tif]

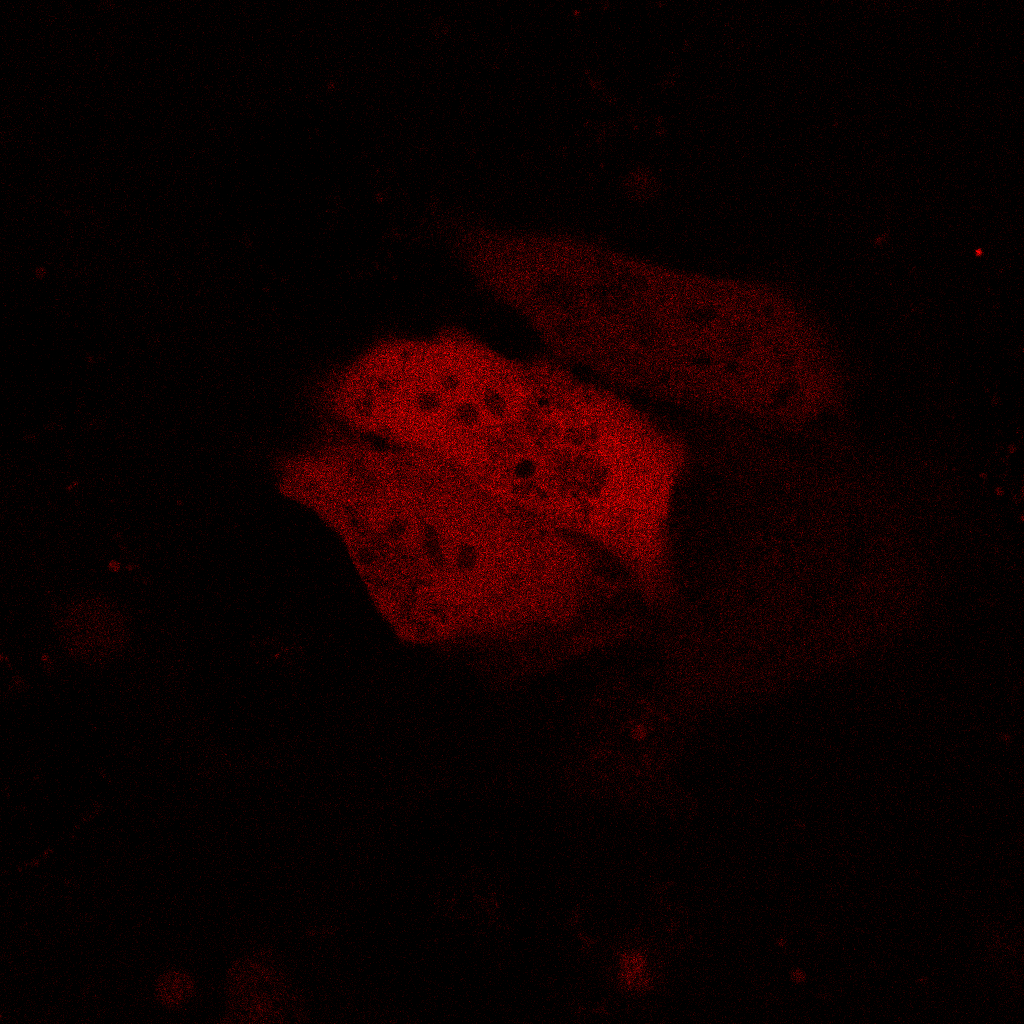

Supplement: Supplementary file 19 — Figure EV9 Source Data [file 44318_2026_816_MOESM19_ESM.zip › B/ECHS1-ΔMTS-Mcherry+GFP-NSP4_mCherry.tif]

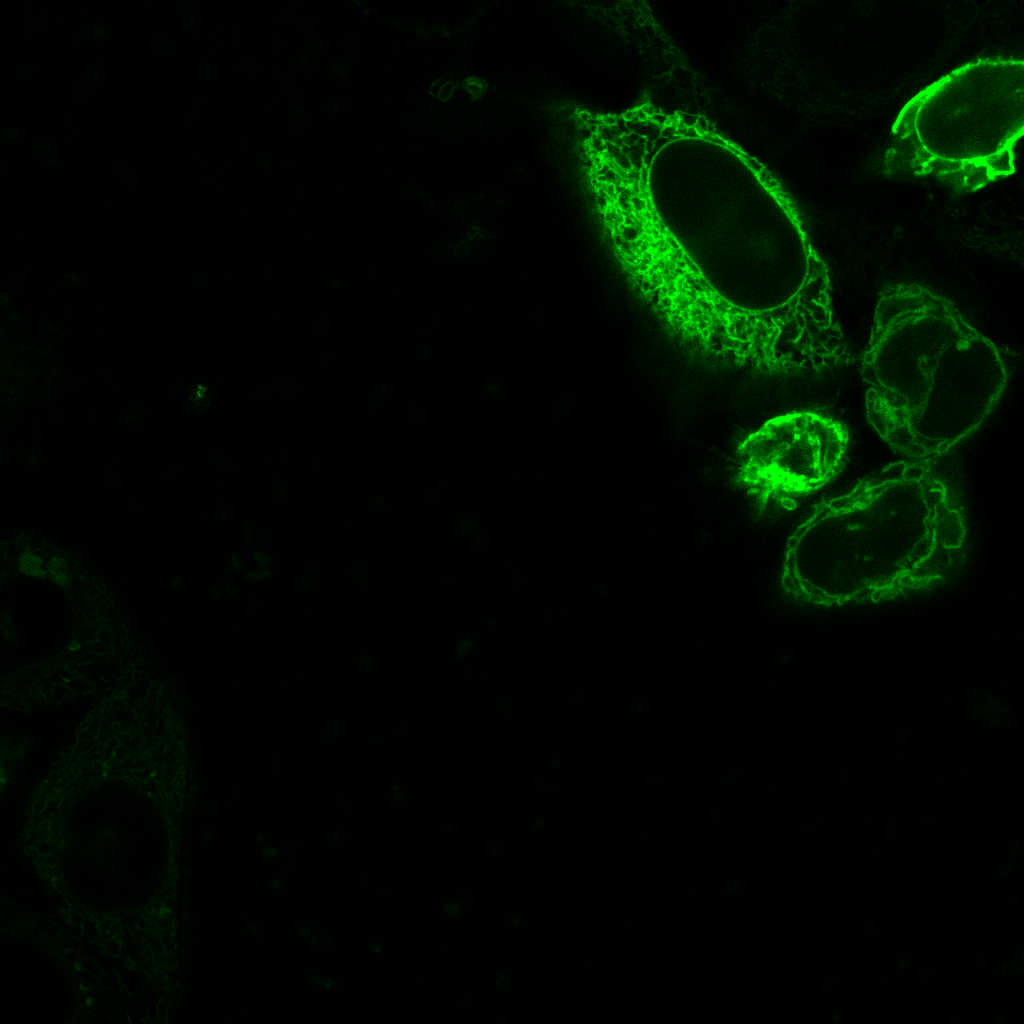

Supplement: Supplementary file 19 — Figure EV9 Source Data [file 44318_2026_816_MOESM19_ESM.zip › B/GFP-NSP3+ECHS1-ΔMTS_EGFP.tif]

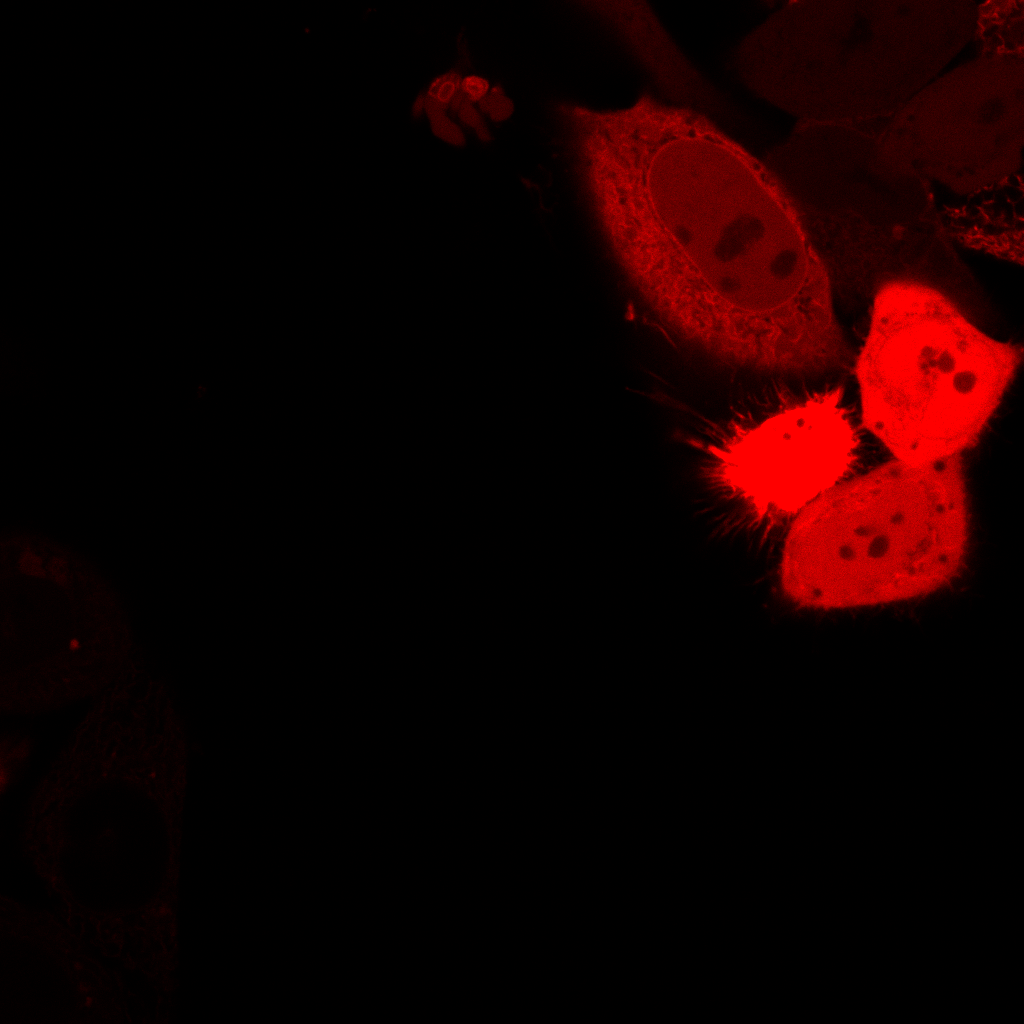

Supplement: Supplementary file 19 — Figure EV9 Source Data [file 44318_2026_816_MOESM19_ESM.zip › B/GFP-NSP3+ECHS1-ΔMTS_mCherry.tif]

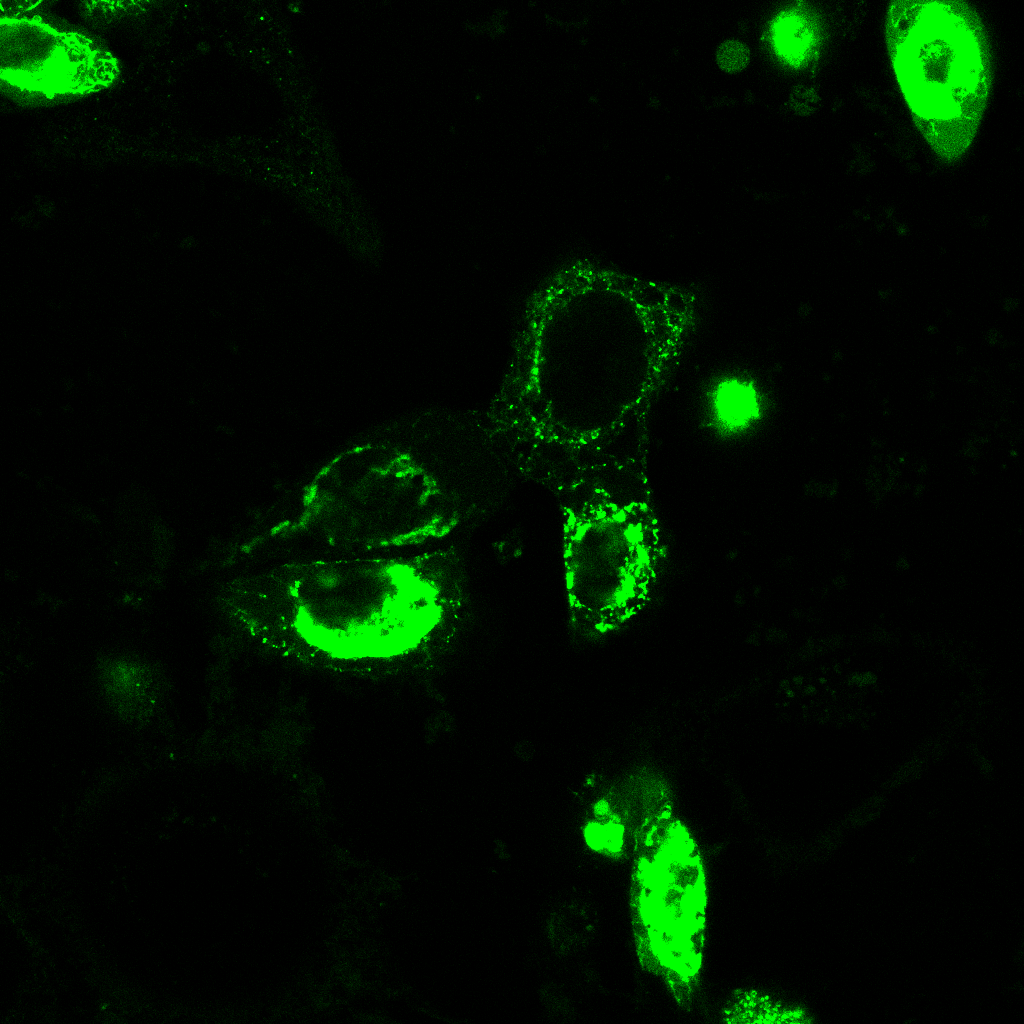

Supplement: Supplementary file 19 — Figure EV9 Source Data [file 44318_2026_816_MOESM19_ESM.zip › B/GFP-NSP3-T2A-NSP4+ECHS1-ΔMTS_GFP.tif]

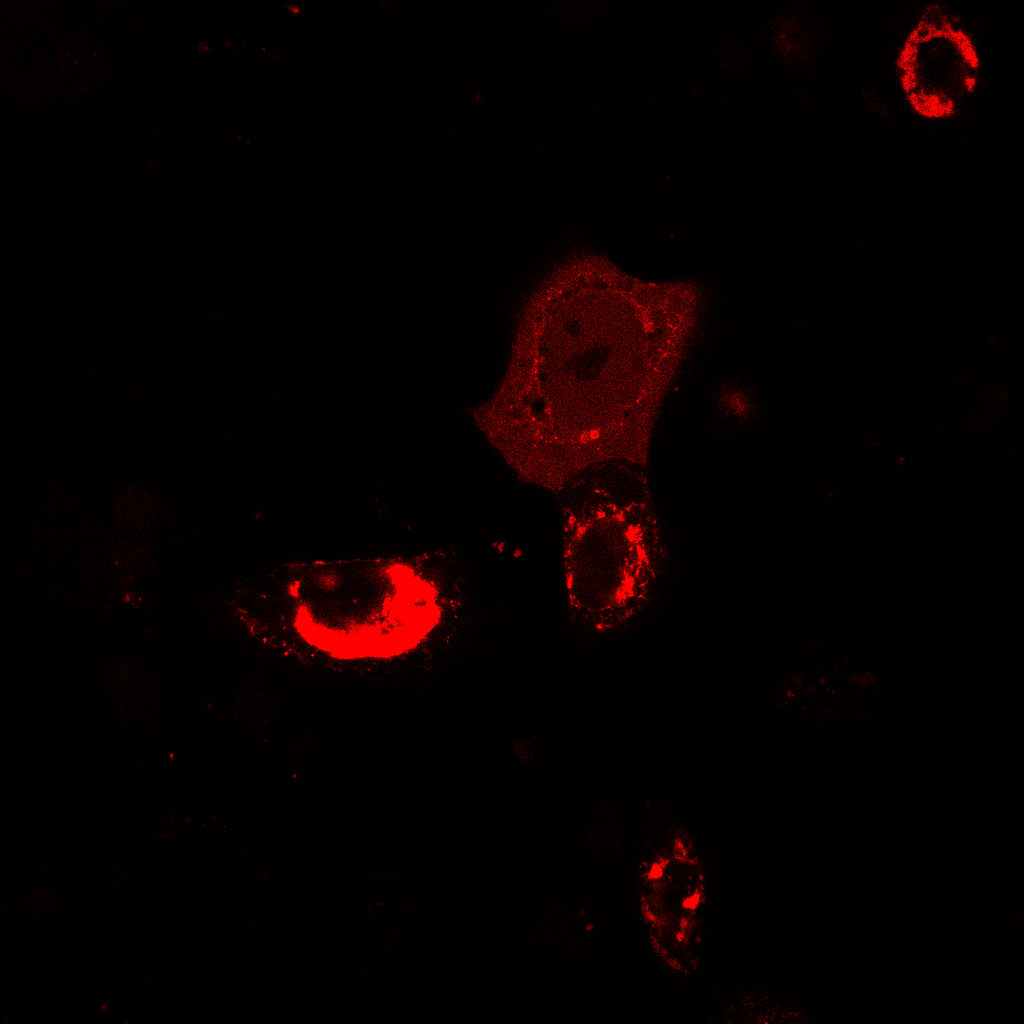

Supplement: Supplementary file 19 — Figure EV9 Source Data [file 44318_2026_816_MOESM19_ESM.zip › B/GFP-NSP3-T2A-NSP4+ECHS1-ΔMTS_mCherry.tif]

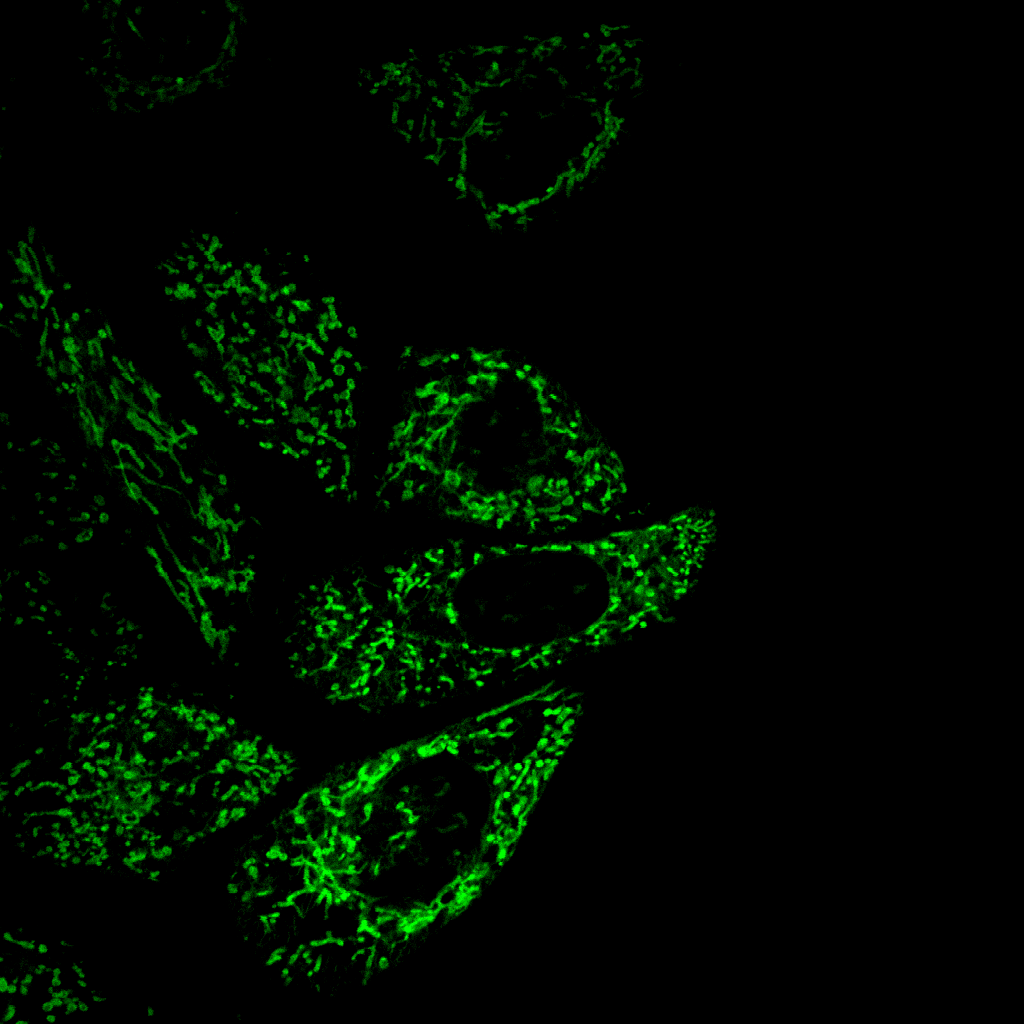

Supplement: Supplementary file 19 — Figure EV9 Source Data [file 44318_2026_816_MOESM19_ESM.zip › B/Mito-Tracker+ECHS1-ΔMTS_EGFP.tif]

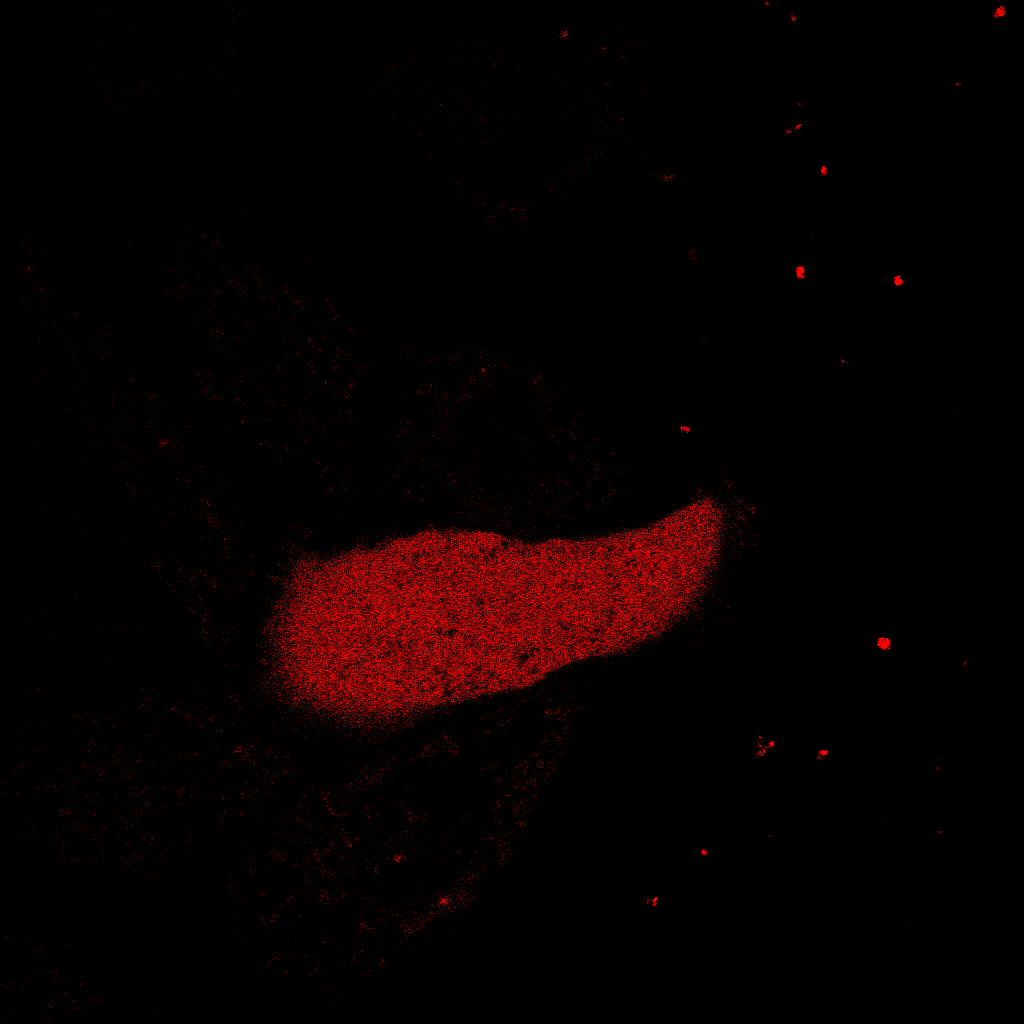

Supplement: Supplementary file 19 — Figure EV9 Source Data [file 44318_2026_816_MOESM19_ESM.zip › B/Mito-Tracker+ECHS1-ΔMTS_mCherry.tif]

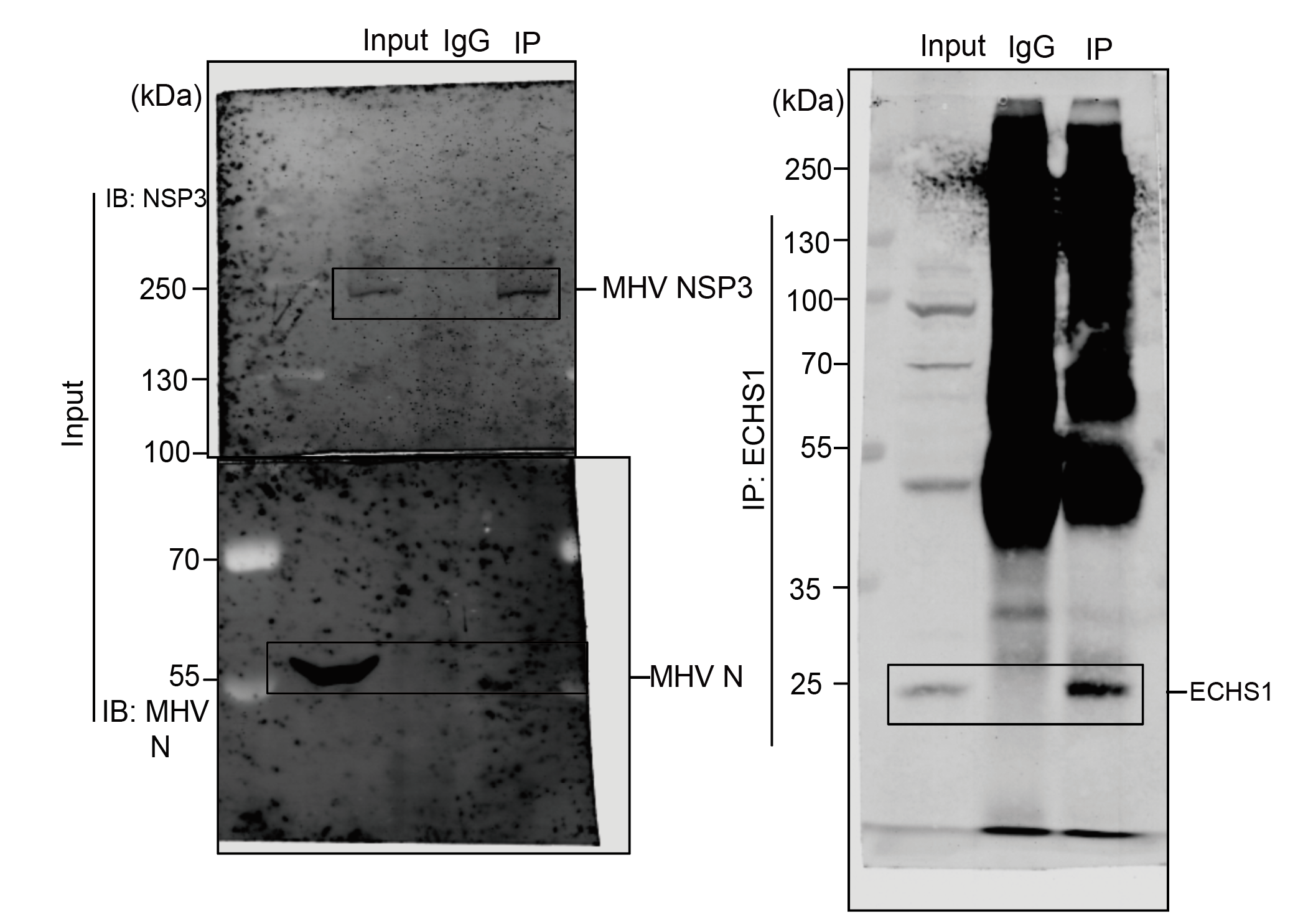

Supplement: Supplementary file 19 — Figure EV9 Source Data [file 44318_2026_816_MOESM19_ESM.zip › C/MHV-NSP3+MHV-Nprotein+ECHS1.tif]

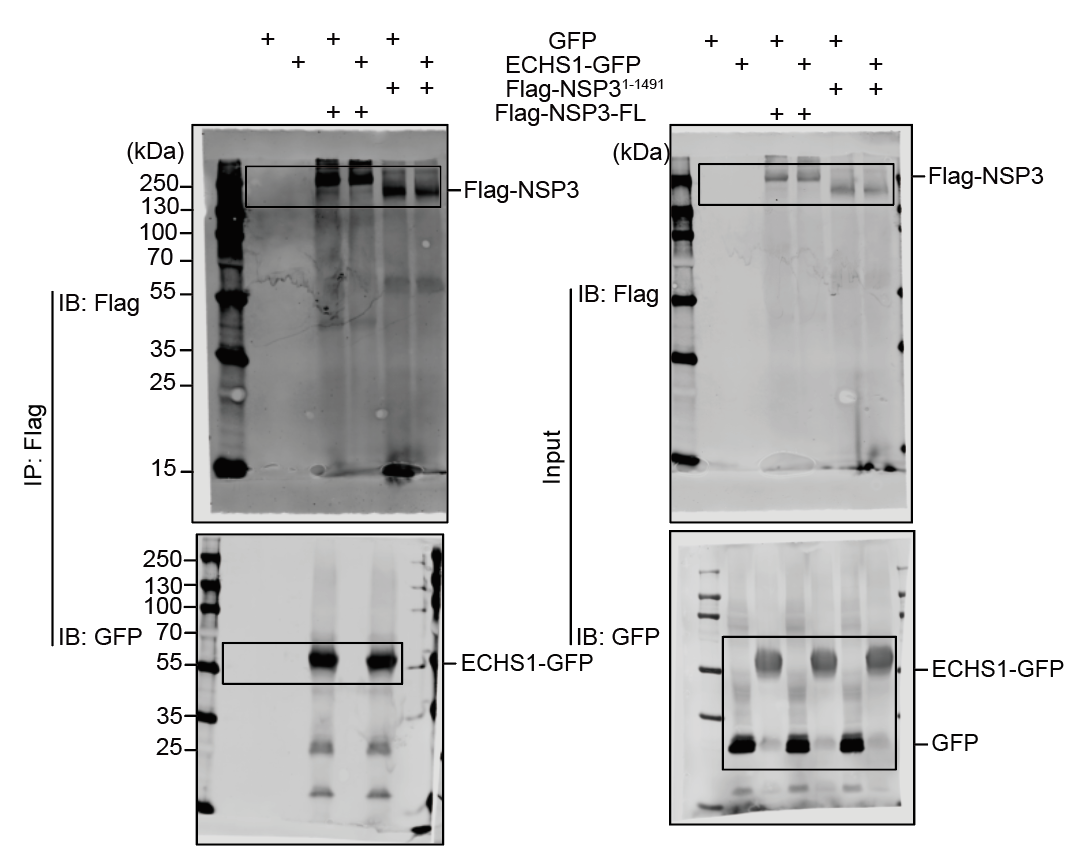

Supplement: Supplementary file 19 — Figure EV9 Source Data [file 44318_2026_816_MOESM19_ESM.zip › D/Flag-NSP3+ECHS1-GFP+GFP.tif]

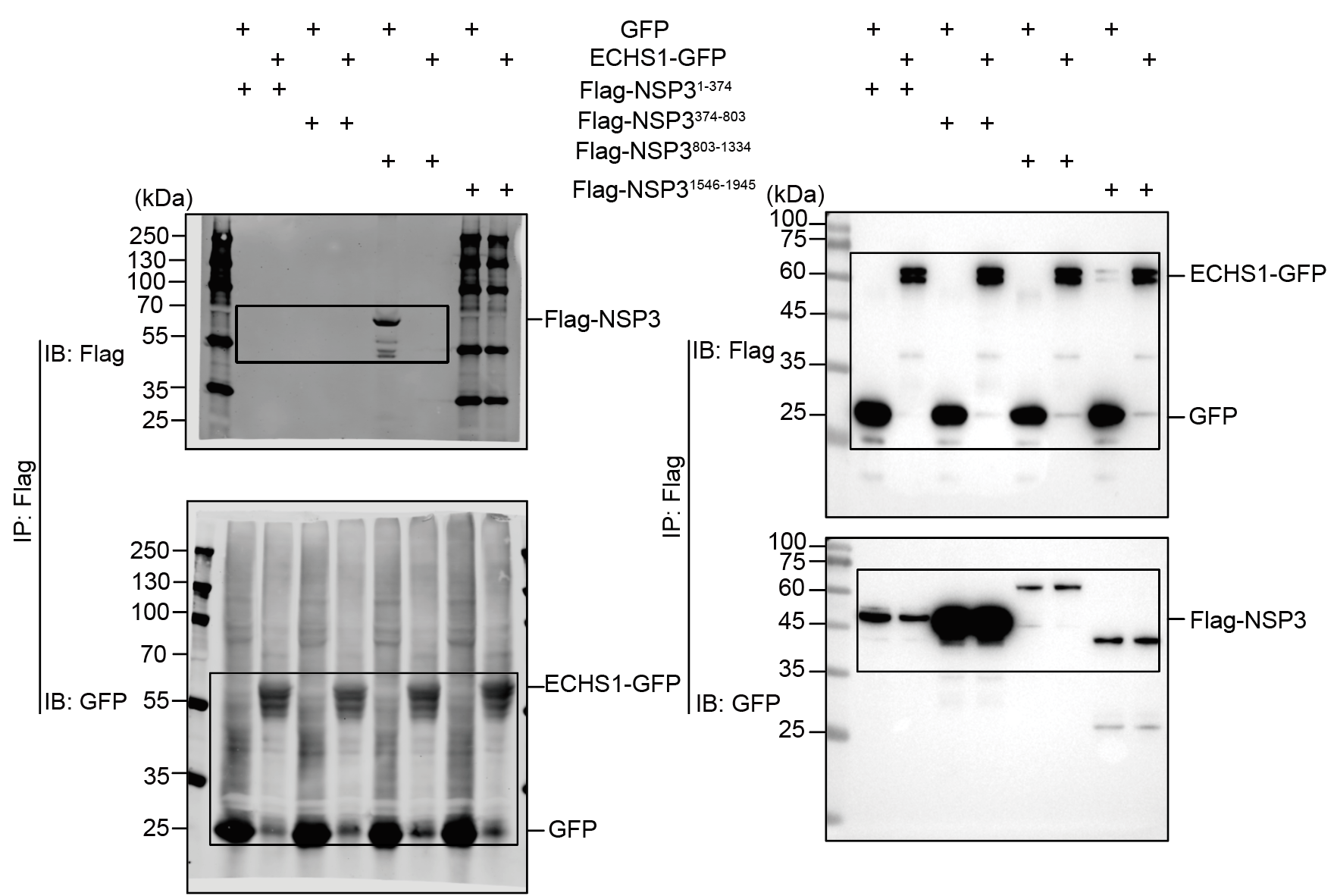

Supplement: Supplementary file 19 — Figure EV9 Source Data [file 44318_2026_816_MOESM19_ESM.zip › E/Flag-NSP3+ECHS1-GFP+GFP.tif]

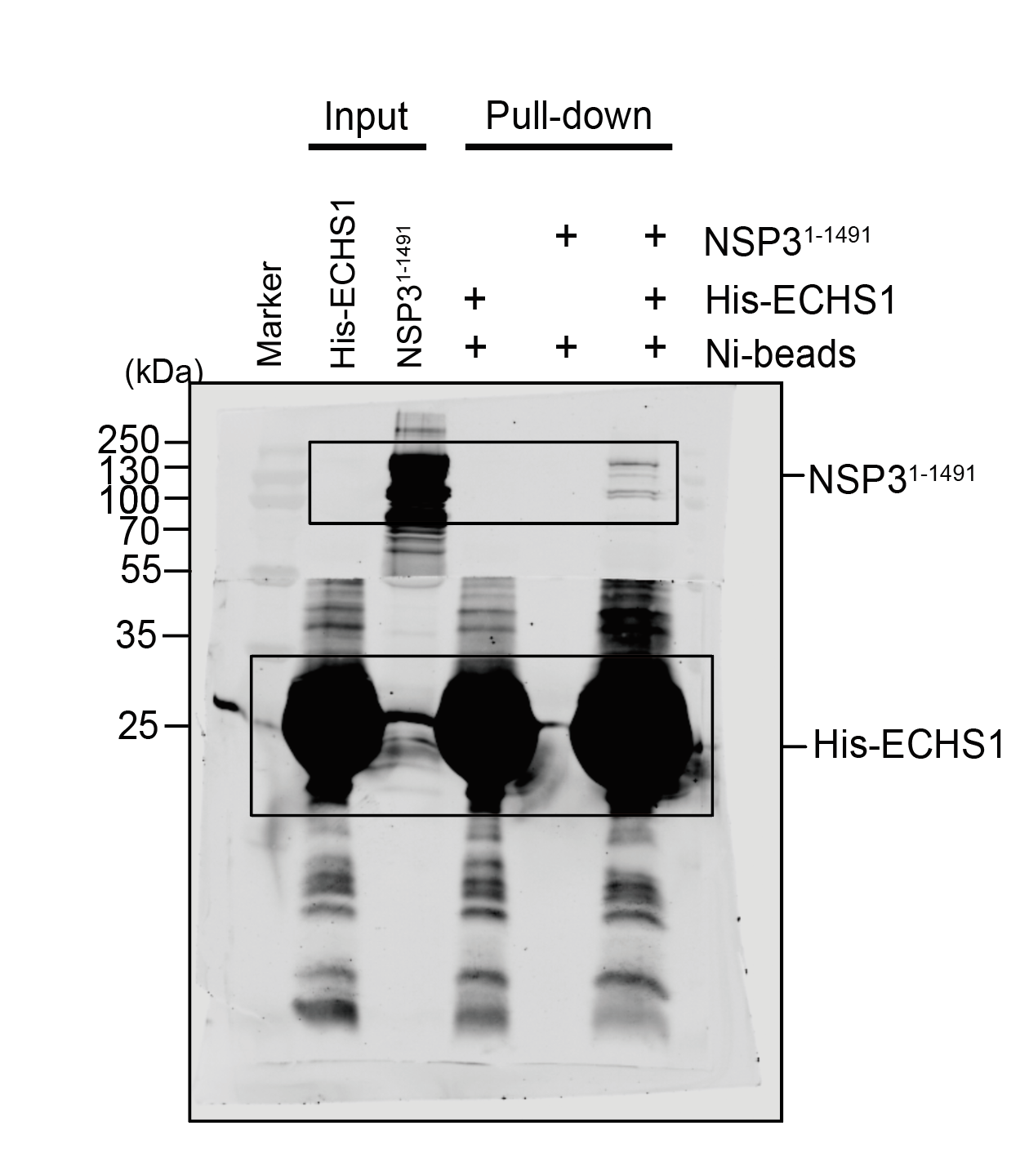

Supplement: Supplementary file 19 — Figure EV9 Source Data [file 44318_2026_816_MOESM19_ESM.zip › F/NSP3+His-ECHS1.tif]

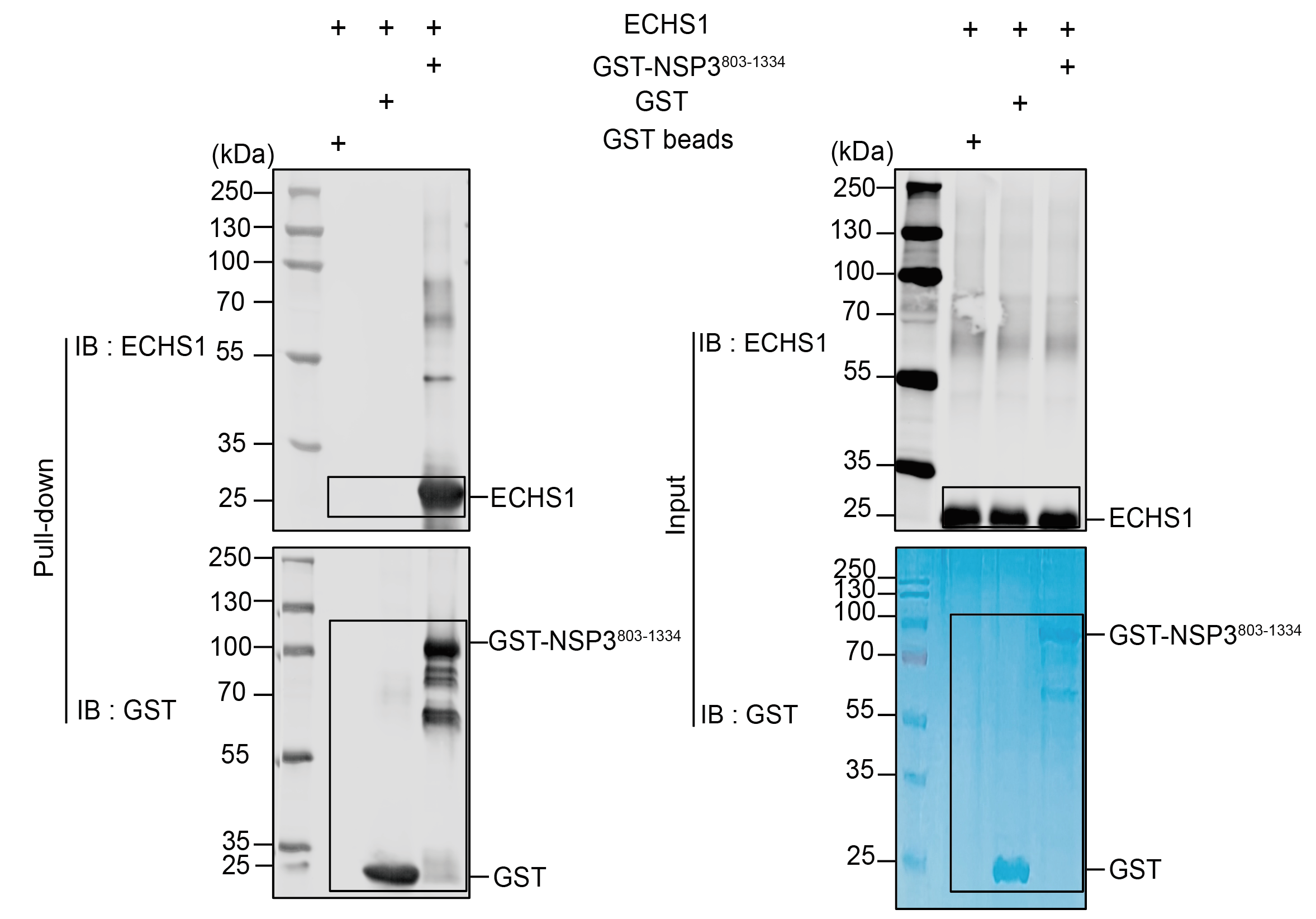

Supplement: Supplementary file 19 — Figure EV9 Source Data [file 44318_2026_816_MOESM19_ESM.zip › G/ECHS1+GST-NSP3+GST+SDS-PAGE.tif]

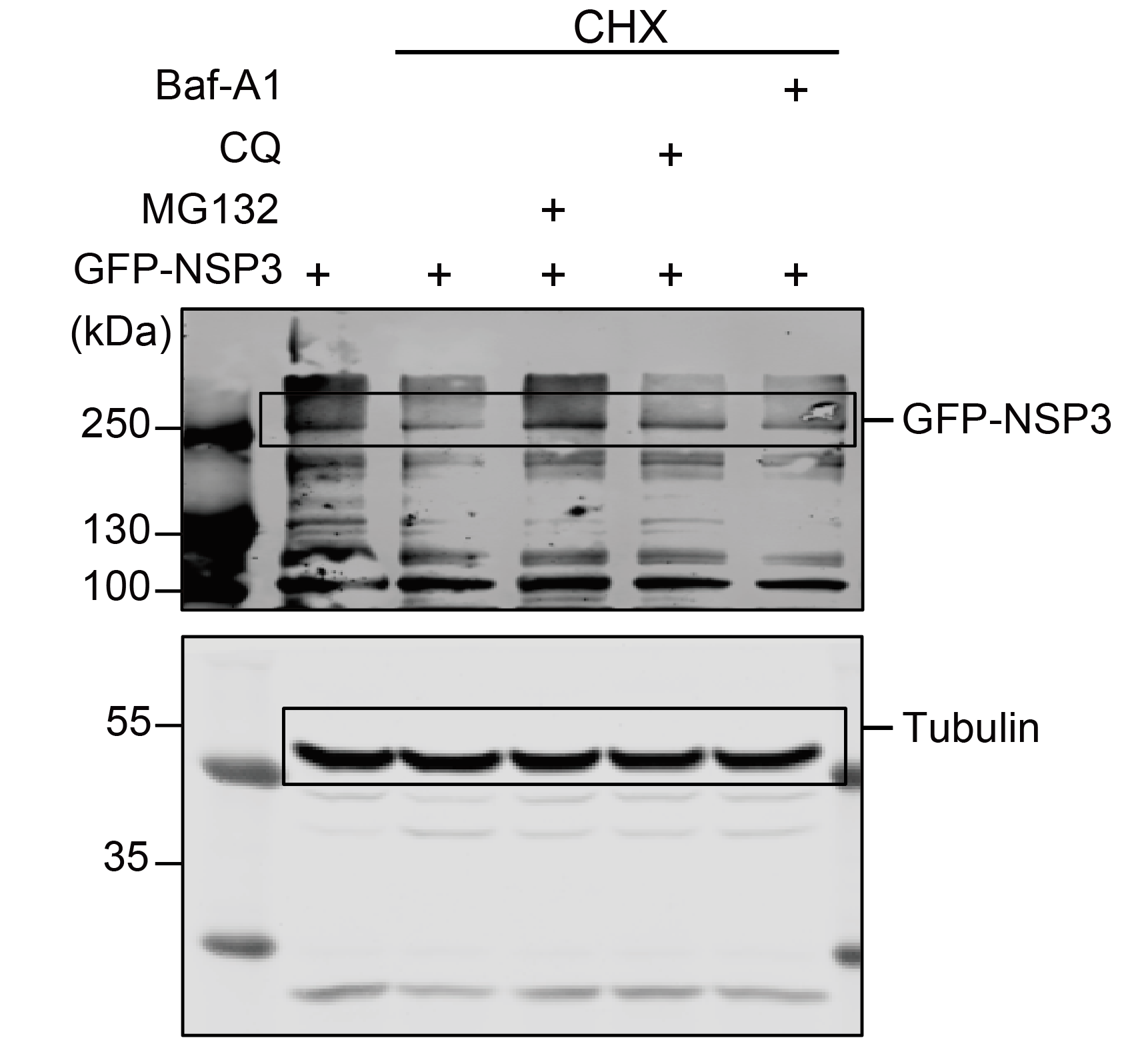

Supplement: Supplementary file 19 — Figure EV9 Source Data [file 44318_2026_816_MOESM19_ESM.zip › H/GFP-NSP3+Tubulin.tif]

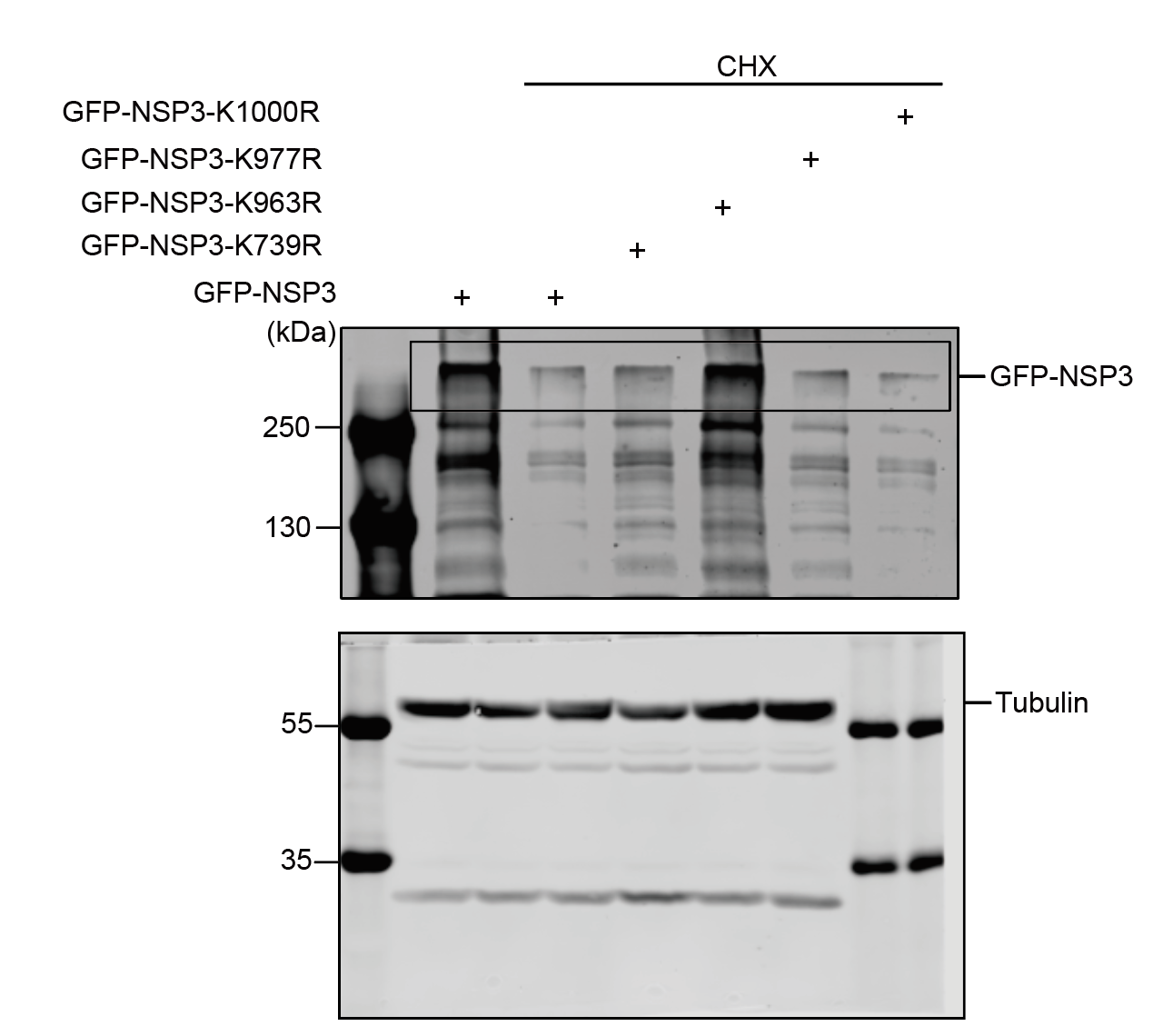

Supplement: Supplementary file 19 — Figure EV9 Source Data [file 44318_2026_816_MOESM19_ESM.zip › I/GFP-NSP3+Tubulin.tif]
