# Supplementary material for: Disruption of ER-mitochondria contact sites by coronavirus replication organelles sustains viral replication via NSP3 stabilization
Source: EMBO J. 2026 May 28;45(13):4379–416. doi: 10.1038/s44318-026-00816-x (PMC13323368; doi:10.1038/s44318-026-00816-x)
Supplement: Supplementary file 20 — Expanded View Figures [file 44318_2026_816_MOESM20_ESM.pdf]

Expanded View Figures

Figure EV1. Time-course analysis of DMV biogenesis, mitochondrial morphological abnormalities, and ERMCS dynamics in DOX-induced NSP3/4 expression system.

(**A**) Representative immunofluorescence images of stable HeLa cell lines with DOX (1 μg/mL)-inducible expression of mCherry-NSP3 and NSP4-GFP, at 0, 6, 12, 24, and 36 h post-DOX induction. Zoomed-in views highlight the puncta accumulation of mCherry-NSP3 and NSP4-GFP over time. Scale bars, 10 μm (main images); 2 μm (zoomed-in views). (**B**) Representative TEM images of mitochondrial morphology and DMV formation in these DOX (1 μg/mL)-inducible stable HeLa cell lines at 0, 6, 12, 24, and 36 h post-DOX induction. Zoomed-in views show mitochondrial structural changes during DMV biogenesis. Scale bars, 1 μm (main images); 200 nm (zoomed-in views). (**C**) Quantification of DMV numbers per cell from TEM images in (**B**). (**D**) Quantification of the proportion of mitochondria with or without ER contact from TEM images in (**B**). Data are presented as mean ± SD, with $n > 15$ cells per time point in each of three independent experimental replicates; *P* values were calculated by one-way ANOVA based on the proportion of mitochondria with ERMCSs. (**E**) Quantification of the proportion of normal mitochondria relative to total mitochondria from TEM images in (**B**). This analysis was performed using three independent experimental replicates. A total of >35 cells were counted per group, pooling data from the three replicates. Data are presented as mean ± SD; *P* values were calculated by one-way ANOVA. Source data are available online for this figure.

© The Author(s)

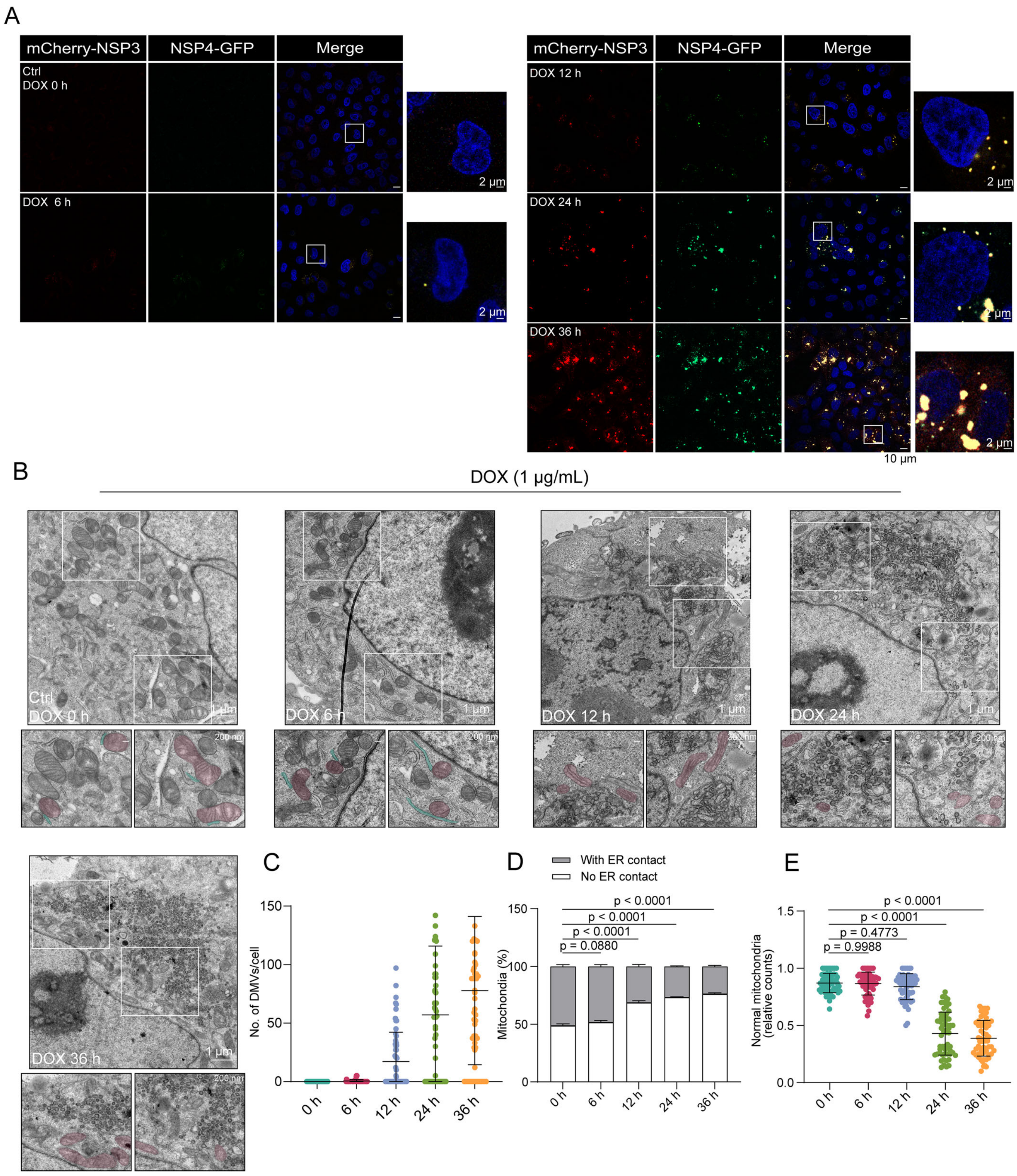
A
mCherry-NSP3
NSP4-GFP
Merge
Ctrl
DOX 0 h
DOX 6 h
DOX 12 h
DOX 24 h
DOX 36 h
2 μm
10 μm
B
DOX (1 μg/mL)
Ctrl
DOX 0 h
DOX 6 h
DOX 12 h
DOX 24 h
DOX 36 h
1 μm
200 nm
C
No. of DMVs/cell
0 h
6 h
12 h
24 h
36 h
D
With ER contact
No ER contact
Mitochondria (%)
p < 0.0001
p < 0.0001
p < 0.0001
p = 0.0880
E
Normal mitochondria (relative counts)
p < 0.0001
p < 0.0001
p = 0.4773
p = 0.9988

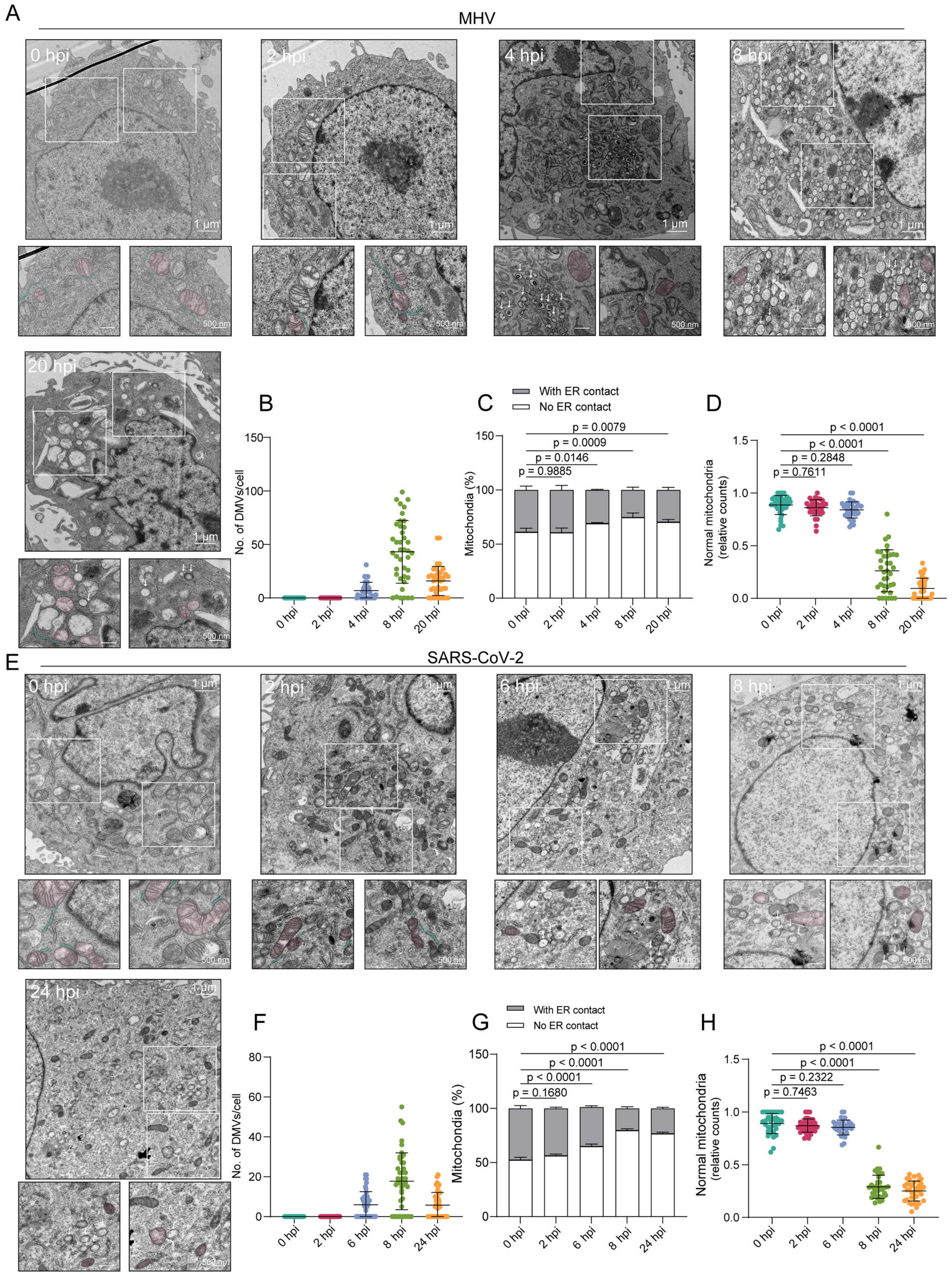
A
MHV
0 hpi
2 hpi
4 hpi
8 hpi
20 hpi
1 µm
500 nm
B
No. of DMVs/cell
0 hpi
2 hpi
4 hpi
8 hpi
20 hpi
C
With ER contact
No ER contact
Mitochondria (%)
p = 0.0079
p = 0.0009
p = 0.0146
p = 0.9885
D
Normal mitochondria (relative counts)
p < 0.0001
p < 0.0001
p = 0.2848
p = 0.7611
E
SARS-CoV-2
0 hpi
2 hpi
6 hpi
8 hpi
24 hpi
F
No. of DMVs/cell
G
With ER contact
No ER contact
Mitochondria (%)
p < 0.0001
p < 0.0001
p < 0.0001
p = 0.1680
H
Normal mitochondria (relative counts)
p < 0.0001
p < 0.0001
p = 0.2322
p = 0.7463

Figure EV2. Time-course analysis of DMV biogenesis, mitochondrial morphological abnormalities, and ERMCS dynamics in MHV- and SARS-CoV-2-infected cells.

(**A**) Representative TEM images of mitochondrial morphology and DMV formation in MHV-infected 17Cl-1 cells (MOI = 5; hpi = 0, 2, 4, 8, 20). Zoomed-in views (bottom) highlight mitochondrial structural changes and DMV accumulation over time. Scale bars, 1 µm (main images); 500 nm (zoomed-in views). (**B**) Quantification of DMV numbers per cell from TEM images in (**A**). Data are presented as mean ± SD ($n > 30$ cells per time point); *P* values were calculated by one-way ANOVA. (**C**) Quantification of the proportion of mitochondria with or without ER contact from TEM images in (**A**). Data are presented as mean ± SD ($n > 30$ cells per time point); *P* values were calculated by one-way ANOVA based on the proportion of mitochondria with ERMCSs. (**D**) Quantification of the proportion of normal mitochondria relative to total mitochondria from TEM images in (**A**). This analysis was performed using three independent experimental replicates. A total of >35 cells were counted per group, pooling data from the three replicates. Data are presented as mean ± SD; *P* values were calculated by one-way ANOVA. (**E**) Representative TEM images of mitochondrial morphology and DMV formation in SARS-CoV-2-infected Vero E6 cells (MOI = 2; hpi = 0, 2, 6, 8, 24). Zoomed-in views (bottom) highlight mitochondrial structural changes and DMV accumulation over time. Scale bars, 1 µm (main images); 500 nm (zoomed-in views). (**F**) Quantification of DMV numbers per cell from TEM images in (**E**). (**G**) Quantification of the proportion of mitochondria with or without ER contact from TEM images in (**E**). Data are presented as mean ± SD ($n > 30$ cells per time point); *P* values were calculated by one-way ANOVA based on the proportion of mitochondria with ERMCSs. (**H**) Quantification of the proportion of normal mitochondria relative to total mitochondria from TEM images in (**E**). This analysis was performed using three independent experimental replicates. A total of >35 cells were counted per group, pooling data from the three replicates. Data are presented as mean ± SD; *P* values were calculated by one-way ANOVA. Source data are available online for this figure.

© The Author(s)

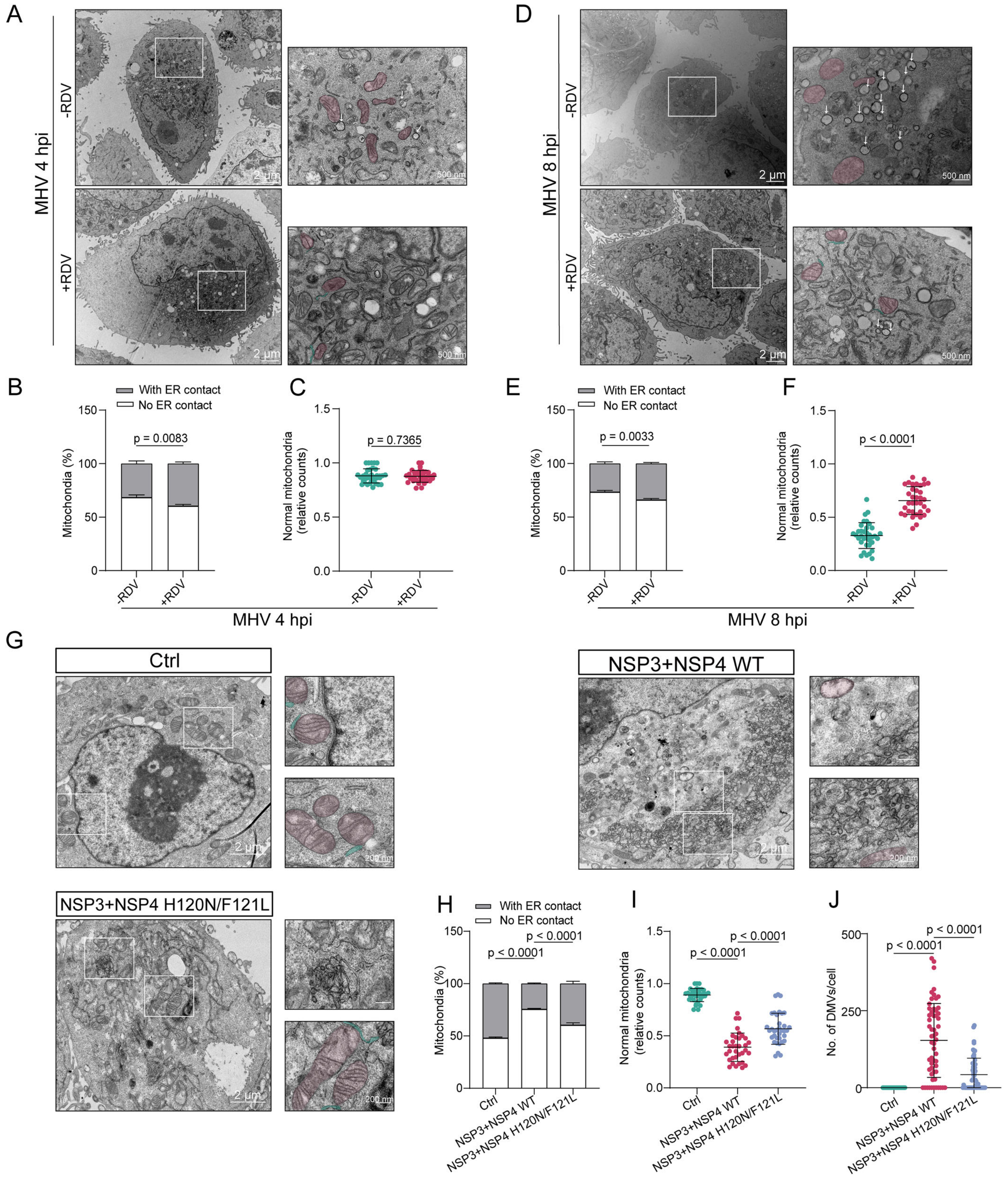
A
MHV 4 hpi
-RDV
+RDV
2 µm
500 nm
D
MHV 8 hpi
-RDV
+RDV
2 µm
500 nm
B
With ER contact
No ER contact
Mitochondia (%)
p = 0.0083
-RDV
+RDV
C
Normal mitochondria (relative counts)
p = 0.7365
-RDV
+RDV
MHV 4 hpi
E
With ER contact
No ER contact
Mitochondia (%)
p = 0.0033
-RDV
+RDV
F
Normal mitochondria (relative counts)
p < 0.0001
-RDV
+RDV
MHV 8 hpi
G
Ctrl
NSP3+NSP4 WT
NSP3+NSP4 H120N/F121L
2 µm
200 nm
H
With ER contact
No ER contact
Mitochondria (%)
p < 0.0001
p < 0.0001
Ctrl
NSP3+NSP4 WT
NSP3+NSP4 H120N/F121L
I
Normal mitochondria (relative counts)
p < 0.0001
p < 0.0001
Ctrl
NSP3+NSP4 WT
NSP3+NSP4 H120N/F121L
J
No. of DMVs/cell
p < 0.0001
p < 0.0001
Ctrl
NSP3+NSP4 WT
NSP3+NSP4 H120N/F121L

Figure EV3. Inhibition of DMV biogenesis protects against mitochondrial morphological abnormalities and ERMCSs loss.

(**A**) Representative TEM images of MHV-infected 17Cl-1 cells treated with or without RDV at 4 hpi. Zoomed-in views show mitochondrial morphology and DMV formation. Scale bars, 2 μm (main panels); 500 nm (zoomed panels). (**B**) Quantification of the proportion of mitochondria with or without ER contact from TEM images in (**A**). Data are presented as mean ± SD ($n = 3$ independent experimental replicates, $n > 35$ cells per group); P values were calculated by unpaired t test. (**C**) Quantification of the proportion of normal mitochondria relative to total mitochondria from TEM images in (A). This analysis was performed using three independent experimental replicates. A total of >35 cells were counted per group, pooling data from the three replicates. Data are presented as mean ± SD; P values were calculated by unpaired t test. (**D**) Representative TEM images of MHV-infected 17Cl-1 cells treated with or without RDV at 8 hpi. Zoomed-in views (right) show mitochondrial morphology and DMV formation. Scale bars, 2 μm (main images); 500 nm (zoomed-in views). (**E**) Quantification of the proportion of mitochondria with or without ER contact from TEM images in (**D**). Data are presented as mean ± SD ($n = 3$ independent experimental replicates, $n > 35$ cells per group); P values were calculated by unpaired t test. (**F**) Quantification of the proportion of normal to total mitochondria from TEM images in (**D**). This analysis was performed using three independent experimental replicates. A total of >35 cells were counted per group, pooling data from the three replicates. Data are presented as mean ± SD; P values were calculated by unpaired t test. (**G**) Representative TEM images of HeLa cells transfected with empty vector (Ctrl), WT GFP-NSP3 + NSP4-mCherry, or GFP-NSP3 + NSP4-mCherry (H120N/F121L mutant). Zoomed-in views (right) show mitochondrial morphology and DMV formation. Scale bars, 2 μm (main images); 200 nm (zoomed-in views). (**H**) Quantification of the proportion of mitochondria with or without ER contact from TEM images in (**G**). Data are presented as mean ± SD ($n > 35$ cells per group); P values were calculated by one-way ANOVA. (**I**) Quantification of the proportion of normal mitochondria relative to total mitochondria from TEM images in (**G**). This analysis was performed using three independent experimental replicates. A total of >35 cells were counted per group, pooling data from the three replicates. Data are presented as mean ± SD; P values were calculated by one-way ANOVA. (**J**) Quantification of DMV numbers per cell from TEM images in (**G**). Data are presented as mean ± SD ($n > 40$ cells per group); P values were calculated by one-way ANOVA. Source data are available online for this figure.

© The Author(s)

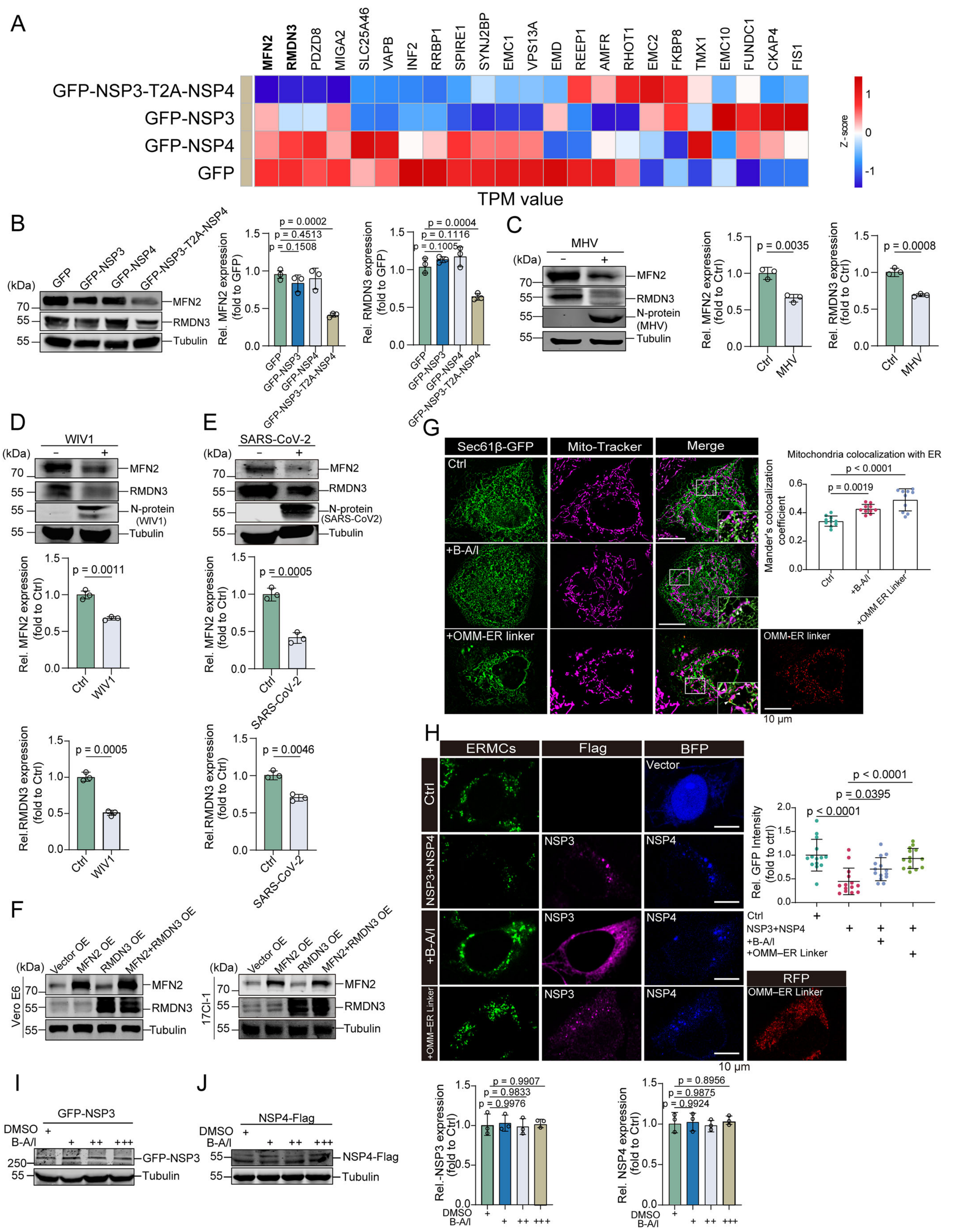
A
MFN2
RMDN3
PDZD8
MIGA2
SLC25A46
VAPB
INF2
RRBP1
SPIRE1
SYNJ2BP
EMC1
VPS13A
EMD
REEP1
AMFR
RHOT1
EMC2
FKBP8
TMX1
EMC10
FUNDC1
CKAP4
FIS1
GFP-NSP3-T2A-NSP4
GFP-NSP3
GFP-NSP4
GFP
TPM value
Z-score
B
C
MHV
D
WIV1
E
SARS-CoV-2
F
G
Sec61β-GFP
Mito-Tracker
Merge
Mitochondria colocalization with ER
Mander's colocalization coefficient
OMM-ER linker
10 μm
H
ERMCs
Flag
BFP
Vector
NSP3
NSP4
RFP
OMM–ER Linker
I
GFP-NSP3
J
NSP4-Flag
DMSO
B-A/I

Figure EV4. DMV formation downregulates the expression of ERMCS proteins MFN2 and RMDN3.

(**A**) Heatmap displaying transcript levels (TPM values, z-score normalized) of ERMCSs-related genes in 293T cells expressing GFP-vector, GFP-NSP3, GFP-NSP4, or GFP-NSP3-T2A-NSP4. (**B**) Immunoblotting and quantification of MFN2 and RMDN3 protein levels in 293T cells expressing GFP-vector, GFP-NSP3, GFP-NSP4, or GFP-NSP3-T2A-NSP4. Data are presented as mean ± SD ($n = 3$ independent experimental replicates); P values were calculated by one-way ANOVA. (**C**) Immunoblotting and quantification of MFN2 and RMDN3 in uninfected or MHV-infected 17Cl-1 cells (MOI = 5, hpi = 24). Data are presented as mean ± SD ($n = 3$ independent experimental replicates); P values were calculated by unpaired t test. (**D**) Immunoblotting and quantification of MFN2 and RMDN3 in uninfected or WIV1-infected Vero E6 cells (MOI = 1, hpi = 24). Data are presented as mean ± SD ($n = 3$ independent experimental replicates); P values were calculated by unpaired t test. (**E**) Immunoblotting and quantification analysis of MFN2 and RMDN3 in uninfected or SARS-CoV-2-infected Vero E6 cells (MOI = 1, hpi = 24). Data are presented as mean ± SD ($n = 3$ independent experimental replicates); P values were calculated by unpaired t test. (**F**) Immunoblotting analysis of MFN2 and RMDN3 in cells stably overexpressing vector control (Vector OE), MFN2, RMDN3, or MFN2 + RMDN3. (**G**) Representative fluorescence images of HeLa cells expressing SEC61B-GFP (ER marker) and Mito-Tracker treated with B-A/I (10 μM) or transfected with an OMM-ER linker. Scale bar, 10 μm. Quantification of mitochondria-ER colocalization (assessed by Manders' colocalization coefficient) is shown in the right panel. Data are presented as mean ± SD ($n = 10$ cells per group); P values were calculated by one-way ANOVA. (**H**) Representative fluorescence images of ERMCSs reporter in HeLa cells co-expressing Flag-NSP3 and NSP4-BFP, treated with B-A/I (10 μM) or transfected with an OMM-ER linker. Quantification of relative GFP fluorescence intensity is shown in the right panel. Data are presented as mean ± SD ($n = 15$ cells per group); P values were calculated by one-way ANOVA. (**I**) Immunoblotting analysis of GFP-NSP3 protein levels in HeLa cells treated with DMSO or increasing concentrations of B-AI (5 μM, 10 μM, 20 μM). Data are presented as mean ± SD ($n = 3$ independent experimental replicates); P values were calculated by one-way ANOVA. (**J**) Immunoblotting analysis of NSP4-Flag protein levels in HeLa cells treated with DMSO or increasing concentrations of B-AI (5 μM, 10 μM, 20 μM). Data are presented as mean ± SD ($n = 3$ independent experimental replicates); P values were calculated by one-way ANOVA. Source data are available online for this figure.

 © The Author(s)

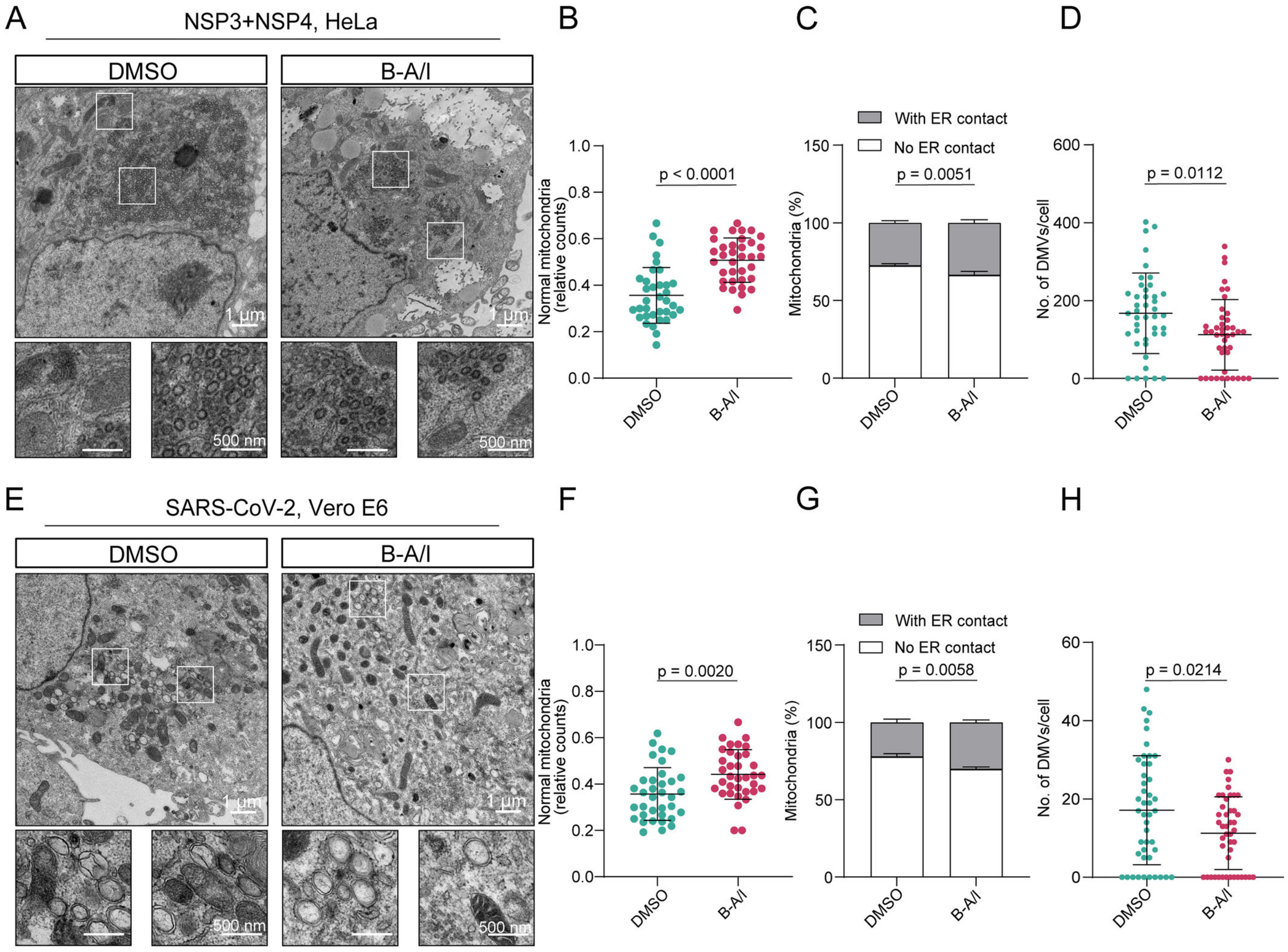

Figure EV5. MFN2 agonist B-A/I alleviates mitochondrial defects and DMV formation induced by co-expressing NSP3 and NSP4 or SARS-CoV-2 infection.

(**A**) Representative TEM images showing mitochondrial morphology and DMV formation in HeLa cells co-expressing NSP3 and NSP4, treated with DMSO or the MFN2 agonist B-A/I (10 μM). Scale bars, 1 μm (main images); 500 nm (zoomed-in views). Zoomed-in views highlight mitochondrial structures and DMV formations. (**B**) Quantification of the proportion of normal to total mitochondria from TEM images in (**A**). This analysis was performed using three independent experimental replicates. A total of >35 cells were counted per group, pooling data from the three replicates. Data are presented as mean ± SD; *P* values were calculated by unpaired *t* test. (**C**) Quantification of the proportion of mitochondria with or without ER contact from TEM images in (**A**). Data are presented as mean ± SD ($n > 35$ cells per group); *P* value was calculated by unpaired *t* test based on the proportion of mitochondria with ERMCSs. (**D**) Quantification of DMV numbers from TEM images in (**A**). Data are presented as mean ± SD ($n > 35$ cells per group); *P* value was calculated by unpaired *t* test. (**E**) Representative TEM images showing mitochondrial morphology and DMV formation in SARS-CoV-2-infected Vero E6 cells (MOI = 1; hpi = 8) treated with DMSO or MFN2 agonist B-A/I (10 μM). Scale bars, 1 μm (main images); 500 nm (zoomed-in views). Zoomed-in views highlight mitochondrial structures and DMV formations. (**F**) Quantification of the proportion of normal to total mitochondria from TEM images in (**E**). This analysis was performed using three independent experimental replicates. A total of >35 cells were counted per group, pooling data from the three replicates. Data are presented as mean ± SD; *P* values were calculated by unpaired *t* test. (**G**) Quantification of the proportion of mitochondria with or without ER contact from TEM images in (**E**). Data are presented as mean ± SD ($n > 35$ cells per group); *P* value was calculated by unpaired *t* test based on the proportion of mitochondria with ERMCSs. (**H**) Quantification of DMV numbers from TEM images in (**E**). Data are presented as mean ± SD ($n > 35$ cells per group); and *P* value was calculated by unpaired *t* test. Source data are available online for this figure.

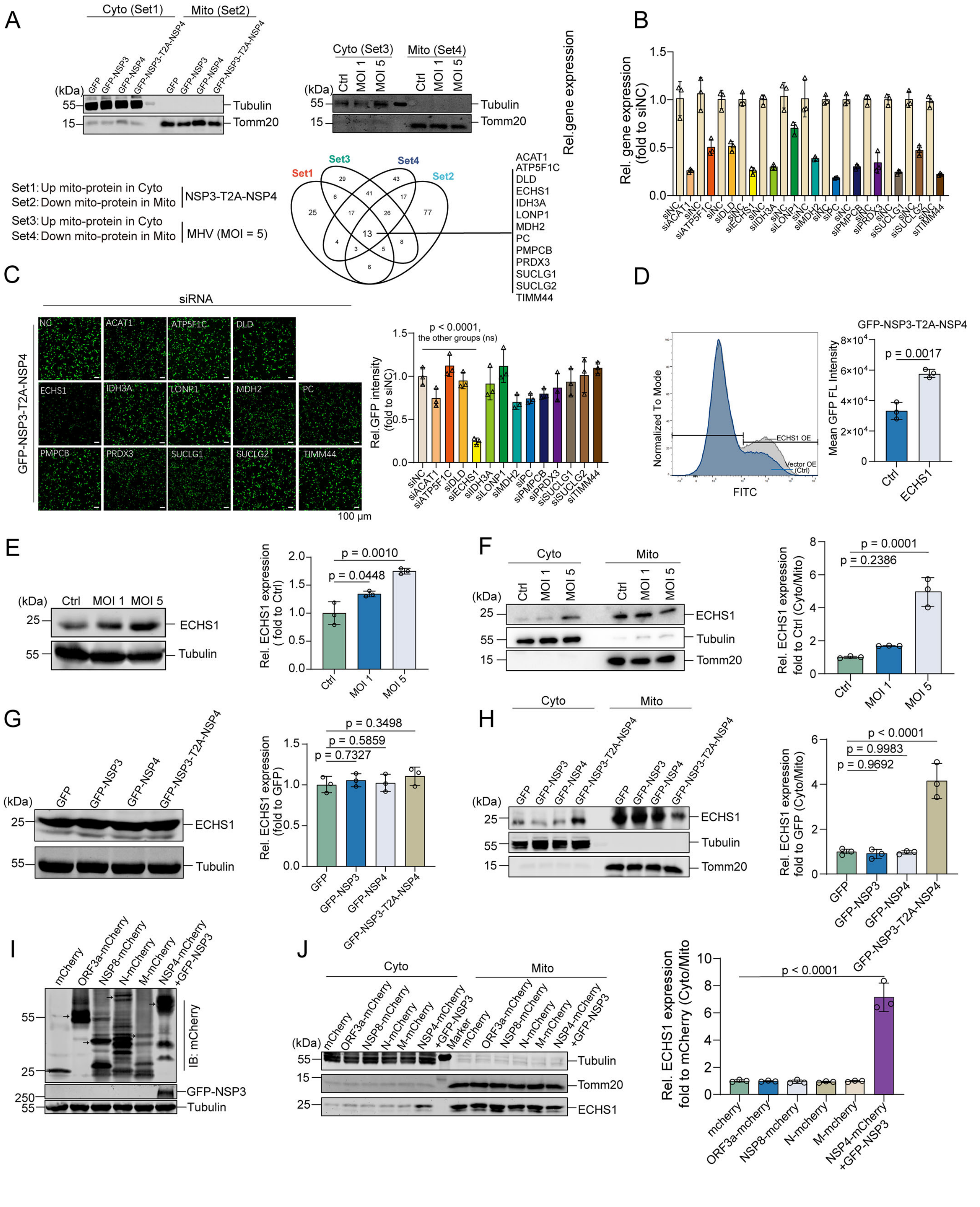
A
Cyto (Set1)
Mito (Set2)
GFP
GFP-NSP3
GFP-NSP4
GFP-NSP3-T2A-NSP4
(kDa)
55
15
Tubulin
Tomm20
Cyto (Set3)
Mito (Set4)
Ctrl
MOI 1
MOI 5
Set1: Up mito-protein in Cyto
Set2: Down mito-protein in Mito
NSP3-T2A-NSP4
Set3: Up mito-protein in Cyto
Set4: Down mito-protein in Mito
MHV (MOI = 5)
Set1
Set3
Set4
Set2
Rel.gene expression
ACAT1
ATP5F1C
DLD
ECHS1
IDH3A
LONP1
MDH2
PC
PMPCB
PRDX3
SUCLG1
SUCLG2
TIMM44
B
Rel. gene expression (fold to siNC)
C
siRNA
GFP-NSP3-T2A-NSP4
NC
100 μm
p < 0.0001, the other groups (ns)
Rel.GFP intensity (fold to siNC)
D
Normalized To Mode
FITC
ECHS1 OE
Vector OE (Ctrl)
GFP-NSP3-T2A-NSP4
Mean GFP FL Intensity
p = 0.0017
Ctrl
ECHS1
E
(kDa)
Ctrl
MOI 1
MOI 5
ECHS1
Tubulin
Rel. ECHS1 expression (fold to Ctrl)
p = 0.0010
p = 0.0448
F
Cyto
Mito
Rel. ECHS1 expression fold to Ctrl (Cyto/Mito)
p = 0.0001
p = 0.2386
G
Rel. ECHS1 expression (fold to GFP)
p = 0.3498
p = 0.5859
p = 0.7327
H
Rel. ECHS1 expression fold to GFP (Cyto/Mito)
p < 0.0001
p = 0.9983
p = 0.9692
I
mCherry
ORF3a-mCherry
NSP8-mCherry
N-mCherry
M-mCherry
NSP4-mCherry +GFP-NSP3
IB: mCherry
GFP-NSP3
Tubulin
J
Marker
Tomm20
ECHS1
Rel. ECHS1 expression fold to mCherry (Cyto/Mito
p < 0.0001

 © The Author(s)

Figure EV6. Proteomic analysis reveals that ECHS1 is released from mitochondria to the cytosol during DMV formation and coronavirus infection.

(**A**) Immunoblotting analysis of cytosolic (Cyto) and mitochondrial (Mito) fractions. Left panel: 293T cells expressing GFP, GFP-NSP3, GFP-NSP4, or GFP-NSP3-T2A-NSP4. Right panel: 17Cl-1 cells uninfected (Ctrl) or infected with MHV (MOI = 5, hpi = 8). Tubulin (cytosolic marker) and Tomm20 (mitochondrial marker) were probed to verify subcellular purification efficiency. Isolated fractions were subsequently subjected to MS to identify differentially expressed mitochondrial proteins, defined as: Set1 (upregulated mitochondrial proteins in cytosol, identified by comparing GFP-NSP3-T2A-NSP4 with GFP, GFP-NSP3, or GFP-NSP4 controls); Set2 (downregulated mitochondrial proteins in mitochondria, identified by comparing GFP-NSP3-T2A-NSP4 with GFP, GFP-NSP3, or GFP-NSP4 controls); Set3 (upregulated mitochondrial proteins in cytosol during MHV infection, MOI = 5); Set4 (downregulated mitochondrial proteins in mitochondria during MHV infection, MOI = 5). Venn diagram illustrating the overlap of differentially expressed mitochondrial proteins across Sets 1–4. (**B**) qPCR analysis of the relative expression of candidate mitochondrial proteins (from (**A**)) in 293T cells treated with targeted siRNA. Expression levels were normalized to GAPDH. Data are presented as mean ± SD ($n = 3$ independent experiments); P values were calculated by unpaired t test. The corresponding P values are as follows: ACAT1 ($P = 0.0019$), ATP5F1C ($P = 0.0067$), DLD ($P = 0.0015$), ECHS1 ($P < 0.0001$), IDH3A ($P < 0.0001$), LONP1 ($P = 0.0386$), MDH2 ($P = 0.0048$), PC ($P < 0.0001$), PMPCB ($P < 0.0001$), PRDX3 ($P = 0.0007$), SUCLG1 ($P < 0.0001$), SUCLG2 ($P = 0.0007$), and TIMM44 ($P < 0.0001$). (**C**) Fluorescence images of GFP-NSP3-T2A-NSP4 expression in 293T cells with the indicated gene knockdowns (siNC: negative control, ACAT1, ATP5F1C, DLD, ECHS1, IDH3A, LONP1, MDH2, PC, PMPCB, PRDX3, SUCLG1, SUCLG2, TIMM44). The right panel presents quantification of relative GFP expression levels, with data shown as mean ± SD ($n = 3$ independent experimental replicates). P values were calculated by one-way ANOVA. Scale bar = 100 µm. (**D**) Flow cytometry histogram and quantification of GFP-NSP3-T2A-NSP4 expression. ECHS1 overexpression increases the mean GFP fluorescence intensity of GFP-NSP3-T2A-NSP4. 293T cells were transiently transfected with ECHS1 (or control vector) and GFP-NSP3-T2A-NSP4. Data are presented as mean ± SD ($n = 3$ independent experiments); P value was calculated by unpaired t test. (**E**) Immunoblotting analysis of ECHS1 in 17Cl-1 cells: uninfected (Ctrl) or infected with MHV at MOI = 1 or MOI = 5. Quantification of ECHS1 protein levels (right panel). Data are presented as Mean ± SD ($n = 3$ independent experiments); P values were calculated by one-way ANOVA. (**F**) Immunoblotting analysis of ECHS1 protein levels in Cyto and Mito fractions from 17Cl-1 cells: uninfected or infected with MHV at MOI = 1 or MOI = 5. Quantification of ECHS1 protein levels (normalized to the Cyto/Mito fold change of Ctrl) (right panel). Data are presented as mean ± SD ($n = 3$ independent experiments); P values were calculated by one-way ANOVA. (**G**) Immunoblotting analysis of ECHS1 protein levels in 293T cells expressing GFP, GFP-NSP3, GFP-NSP4, or GFP-NSP3-T2A-NSP4. Quantification of ECHS1 levels (right panel). Data are presented as mean ± SD ($n = 3$ independent experiments); P values were calculated by one-way ANOVA. (**H**) Immunoblotting analysis of ECHS1 protein levels in Cyto and Mito fractions from 293T cells expressing GFP, GFP-NSP3, GFP-NSP4, or GFP-NSP3-T2A-NSP4. Quantification of ECHS1 protein levels (normalized to the Cyto/Mito fold change of GFP) (right panel). Data are presented as mean ± SD ($n = 3$ independent experiments); P values were calculated by one-way ANOVA. (**I**) Immunoblotting analysis of 293T cells transiently transfected with mCherry (control), SARS-CoV-2 ORF3a-mCherry, SARS-CoV-2 NSP8-mCherry, SARS-CoV-2 N-mCherry, SARS-CoV-2 M-mCherry, or NSP4-mCherry + GFP-NSP3. (**J**) Immunoblotting analysis of ECHS1 protein levels in Cyto and Mito fractions from 293T cells transiently transfected with mCherry (control), ORF3a-mCherry, NSP8-mCherry, N-mCherry, M-mCherry, or NSP4-mCherry + GFP-NSP3. The right panel presents quantification of the Cyto/Mito fold change of ECHS1 (normalized to mCherry control). Data are presented as mean ± SD ($n = 3$ independent experimental replicates). P values were calculated by one-way ANOVA. Source data are available online for this figure.

© The Author(s)

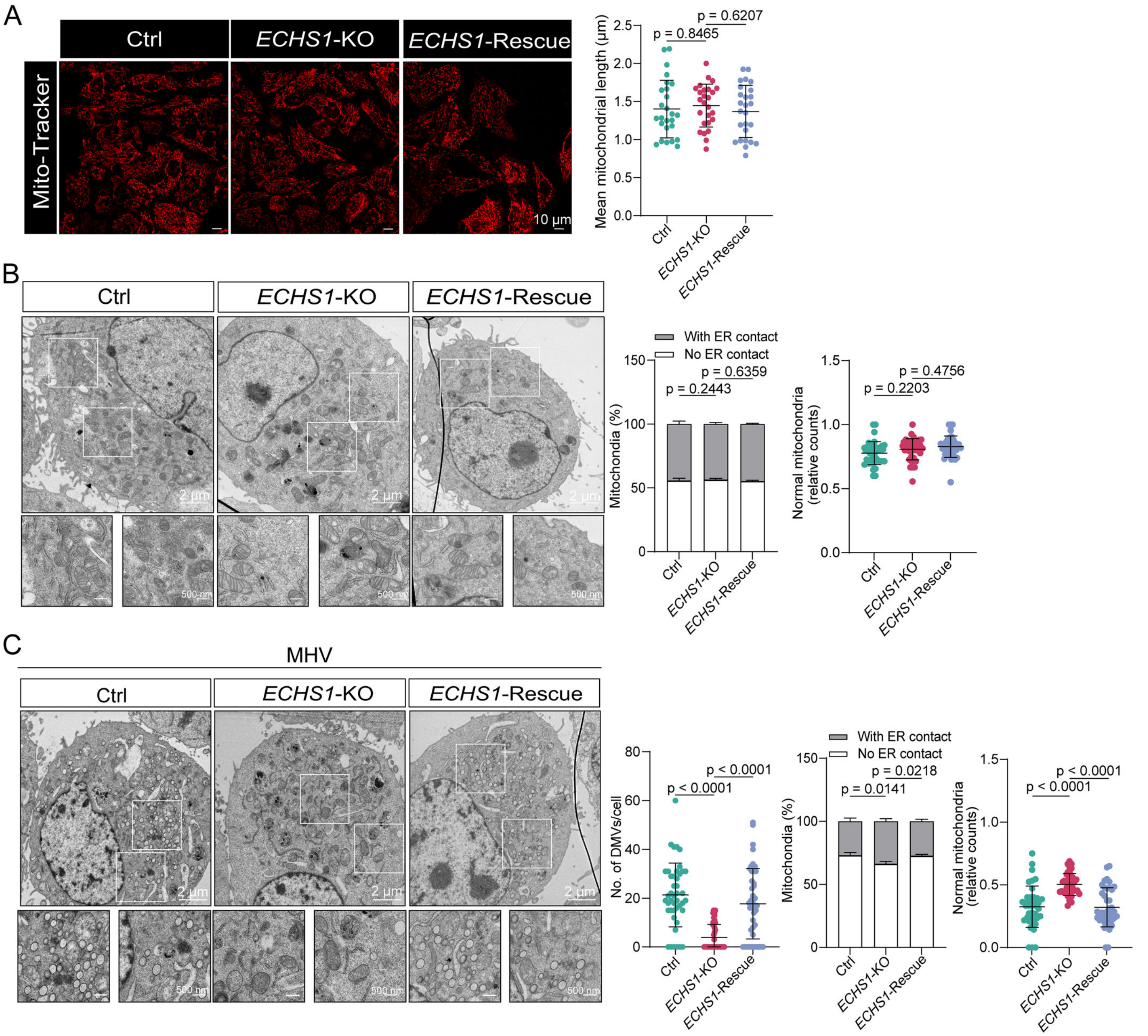

Figure EV7. ECHS1 deficiency protects against mitochondrial morphological abnormalities and ERMCSs loss during MHV infection.

(**A**) Representative fluorescence images of mitochondria (stained with Mito-Tracker Red) in Ctrl, *ECHS1*-KO, and *ECHS1*-Rescue HeLa cells under uninfected conditions. Scale bar, 10 μm. The right panel shows quantification of mean mitochondrial length, which was determined using the Mitochondrial Analyzer plugin in ImageJ with 2D threshold. Data are presented as mean ± SD; *P* values were calculated by one-way ANOVA. (**B**) Representative TEM images of Ctrl, *ECHS1*-KO, and *ECHS1*-Rescue HeLa cells under uninfected conditions, showing mitochondrial morphology and ERMCSs. Scale bars, 2 μm (main images) and 500 nm (zoomed-in views). Data are presented as mean ± SD; *P* values were calculated by one-way ANOVA. (**C**) Representative TEM images of Ctrl, *ECHS1*-KO, and *ECHS1*-Rescue 17Cl-1 cells infected with MHV (MOI = 1, hpi = 8), showing mitochondrial morphology, ERMCSs, and DMV formation. Scale bars, 2 μm (main images) and 500 nm (zoomed-in views). Data are presented as mean ± SD (*n* > 30 cells per group). *P* values were calculated by one-way ANOVA. Source data are available online for this figure.

 © The Author(s)

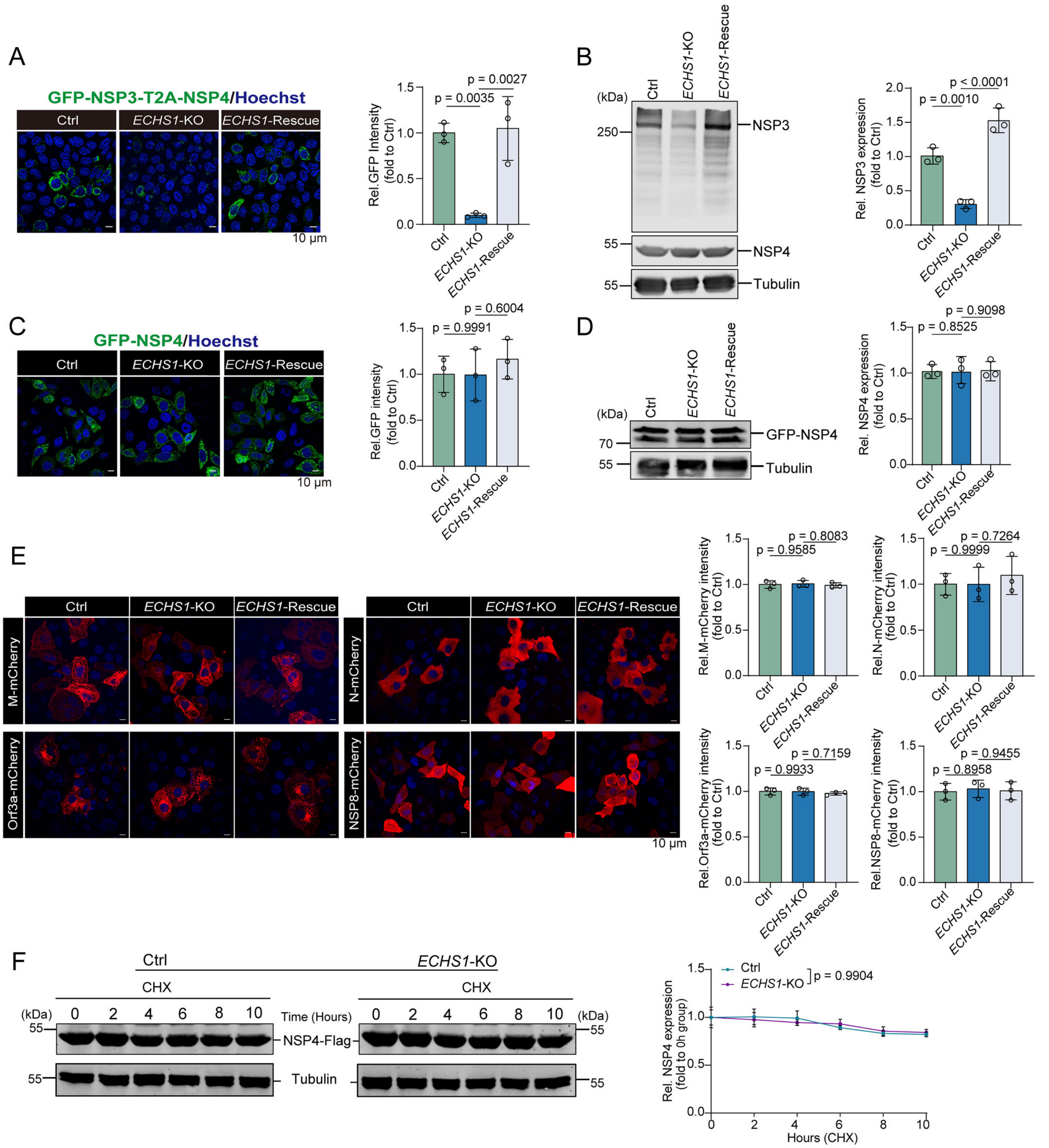
A
GFP-NSP3-T2A-NSP4/Hoechst
Ctrl
ECHS1-KO
ECHS1-Rescue
10 μm
Rel.GFP Intensity (fold to Ctrl)
p = 0.0035
p = 0.0027
B
(kDa)
NSP3
NSP4
Tubulin
Rel. NSP3 expression (fold to Ctrl)
p = 0.0010
p < 0.0001
C
GFP-NSP4/Hoechst
Rel.GFP intensity (fold to Ctrl)
p = 0.9991
p = 0.6004
D
GFP-NSP4
Rel. NSP4 expression (fold to Ctrl)
p = 0.8525
p = 0.9098
E
M-mCherry
N-mCherry
Orf3a-mCherry
NSP8-mCherry
Rel.M-mCherry intensity (fold to Ctrl)
p = 0.9585
p = 0.8083
Rel.N-mCherry intensity (fold to Ctrl)
p = 0.9999
p = 0.7264
Rel.Orf3a-mCherry intensity (fold to Ctrl)
p = 0.9933
p = 0.7159
Rel.NSP8-mCherry intensity (fold to Ctrl)
p = 0.8958
p = 0.9455
F
CHX
Time (Hours)
NSP4-Flag
Rel. NSP4 expression (fold to 0h group)
Ctrl
ECHS1-KO
p = 0.9904
Hours (CHX)

Figure EV8. ECHS1 specifically regulates the expression of NSP3 without affecting the levels of other viral proteins.

(**A**) Representative immunofluorescence images showing the expression of GFP-NSP3-T2A-NSP4 in Ctrl, *ECHS1*-KO, and *ECHS1*-Rescue HeLa cells. Scale bar, 10 μm. The right panel presents quantification of relative GFP fluorescence intensity. Data are presented as mean ± SD ($n = 3$ independent experimental replicates); *P* values were calculated by one-way ANOVA. (**B**) Immunoblotting analysis of NSP3 and NSP4 protein levels in Ctrl, *ECHS1*-KO, and *ECHS1*-Rescue HeLa cells. Data are presented as mean ± SD ($n = 3$ independent experimental replicates); *P* values were calculated by one-way ANOVA. (**C**) Representative immunofluorescence images showing the expression of GFP-NSP4 in Ctrl, *ECHS1*-KO, and *ECHS1*-Rescue HeLa cells. Scale bar, 10 μm. Data are presented as mean ± SD ($n = 3$ independent experimental replicates); *P* values were calculated by one-way ANOVA. (**D**) Immunoblotting analysis of GFP-NSP4 protein levels in Ctrl, *ECHS1*-KO, and *ECHS1*-Rescue HeLa cells. Data are presented as mean ± SD ($n = 3$ independent experimental replicates); *P* values were calculated by one-way ANOVA. (**E**) Representative immunofluorescence images showing the expression of mCherry-tagged viral proteins (M, N, ORF3a, NSP8) in Ctrl, *ECHS1*-KO, and *ECHS1*-Rescue HeLa cells. Scale bar, 10 μm. The right panels present quantification of relative mCherry fluorescence intensity for each viral protein. Data are presented as mean ± SD ($n = 3$ independent experimental replicates); *P* values were calculated by one-way ANOVA. (**F**) Immunoblotting analysis of GFP-NSP4 stability in HeLa Ctrl and *ECHS1*-KO cells treated with CHX for 0–10 h. The right panel shows quantification of relative GFP-NSP4 protein levels over time. Data are presented as mean ± SD ($n = 3$ independent experimental replicates). *P* values for the main effect of group (Ctrl vs *ECHS1*-KO) were calculated by two-way ANOVA. Source data are available online for this figure.

 © The Author(s)

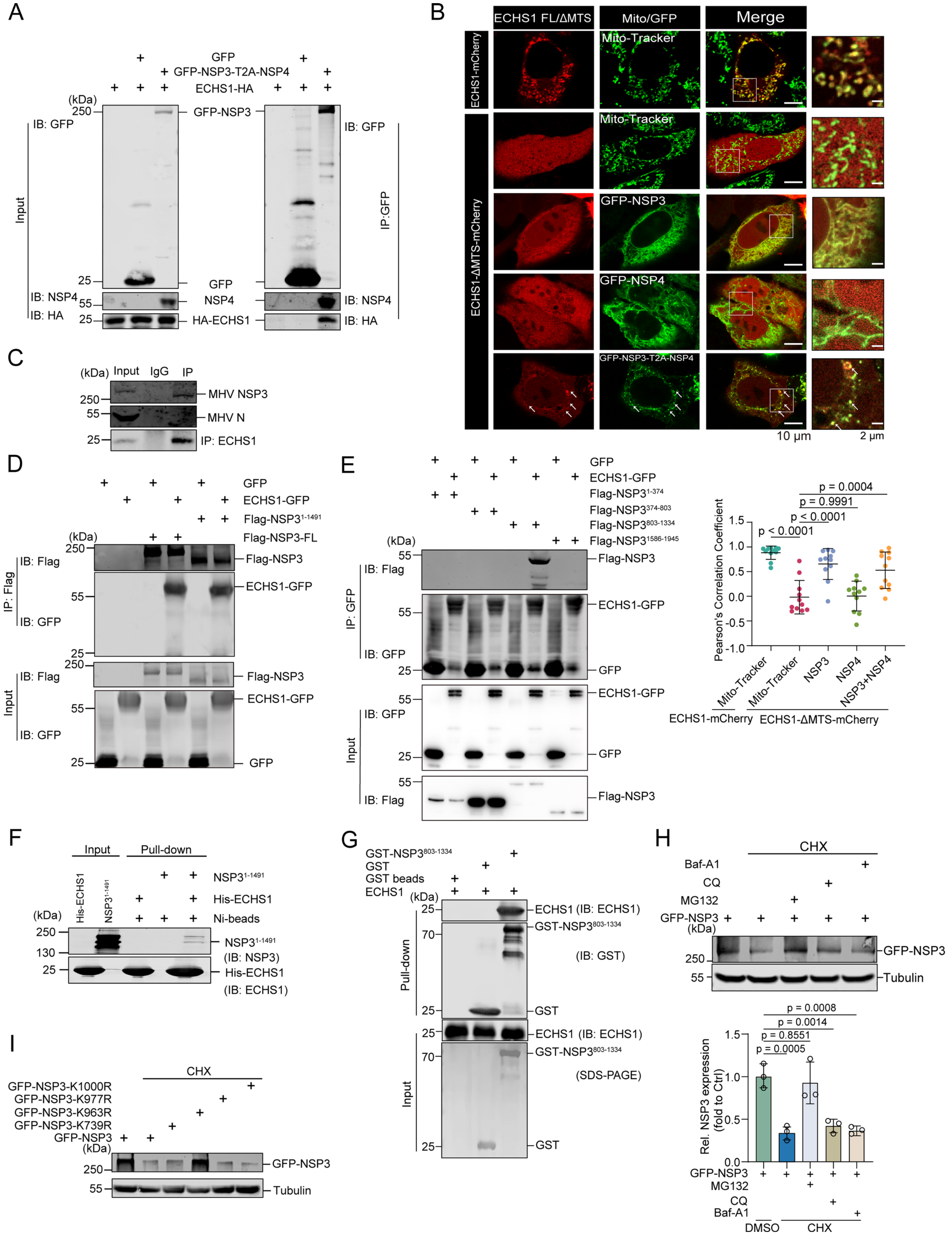
A
GFP
GFP-NSP3-T2A-NSP4
ECHS1-HA
(kDa)
250
25
55
IB: GFP
Input
IB: NSP4
IB: HA
GFP-NSP3
GFP
NSP4
HA-ECHS1
IB: GFP
IP:GFP
IB: NSP4
IB: HA
B
ECHS1 FL/ΔMTS
Mito/GFP
Merge
ECHS1-mCherry
ECHS1-ΔMTS-mCherry
Mito-Tracker
GFP-NSP3
GFP-NSP4
GFP-NSP3-T2A-NSP4
10 μm
2 μm
C
(kDa) Input IgG IP
MHV NSP3
MHV N
IP: ECHS1
D
GFP
ECHS1-GFP
Flag-NSP3 1-1491
Flag-NSP3-FL
IP: Flag
IB: Flag
IB: GFP
Flag-NSP3
ECHS1-GFP
Input
GFP
E
GFP
ECHS1-GFP
Flag-NSP3 1-374
Flag-NSP3 374-803
Flag-NSP3 803-1334
Flag-NSP3 1586-1945
IP: GFP
IB: Flag
IB: GFP
Flag-NSP3
ECHS1-GFP
GFP
Input
p = 0.0004
p = 0.9991
p < 0.0001
p < 0.0001
Pearson's Correlation Coefficient
Mito-Tracker
Mito-Tracker
NSP3
NSP4
NSP3+NSP4
ECHS1-mCherry
ECHS1-ΔMTS-mCherry
F
Input
Pull-down
His-ECHS1
NSP3 1-1491
NSP3 1-1491
His-ECHS1
Ni-beads
NSP3 1-1491 (IB: NSP3)
His-ECHS1 (IB: ECHS1)
G
GST-NSP3 803-1334
GST
GST beads
ECHS1
Pull-down
ECHS1 (IB: ECHS1)
GST-NSP3 803-1334
(IB: GST)
GST
Input
(SDS-PAGE)
H
CHX
Baf-A1
CQ
MG132
GFP-NSP3
GFP-NSP3
Tubulin
p = 0.0008
p = 0.0014
p = 0.8551
p = 0.0005
Rel. NSP3 expression (fold to Ctrl)
DMSO
CHX
I
CHX
GFP-NSP3-K1000R
GFP-NSP3-K977R
GFP-NSP3-K963R
GFP-NSP3-K739R
GFP-NSP3
GFP-NSP3
Tubulin

Figure EV9. ECHS1 directly interacts with NSP3$^{803\text{-}1334}$ domain.

(**A**) Co-IP analysis of the interaction between ECHS1-HA and GFP-NSP3-T2A-NSP4 in 293T cells. Cell lysates were immunoprecipitated with GFP antibody (IP: GFP), followed by immunoblotting with GFP, NSP4 or HA antibodies. (**B**) Representative fluorescence images showing the subcellular localization of ECHS1-mCherry (full-length, FL) and ECHS1-ΔMTS-mCherry (mitochondrial targeting sequence, MTS, deleted) in HeLa cells. Scale bars, 10 µm (main images) and 2 µm (zoomed-in views). The lower panel quantifies colocalization using Pearson's correlation coefficient. Data are presented as mean ± SD ($n = 10$ independent fields of view per group); P values were calculated by one-way ANOVA. (**C**) Endogenous co-IP analysis of the interaction between ECHS1 and MHV NSP3 in MHV-infected 17Cl-1 cells (MOI = 10, hpi = 8). Cell lysates were immunoprecipitated with an ECHS1 antibody (IP: ECHS1) or IgG control, followed by immunoblotting with MHV NSP3 and ECHS1 antibodies. (**D**) Co-IP analysis of the interaction between ECHS1-GFP and Flag-NSP3 truncation mutants (full-length Flag-NSP3-FL and truncated Flag-NSP3$^{1\text{-}1491}$) in 293 T cells. Cell lysates were immunoprecipitated with Flag antibody (IP: Flag), followed by immunoblotting with Flag and GFP antibodies. (**E**) Co-IP analysis of the interaction between ECHS1-GFP and Flag-NSP3 variants (residues 1–374, 374–803, 803–1334, 1586–1945) in 293T cells. Cell lysates were immunoprecipitated with GFP antibody (IP: GFP), followed by immunoblotting with Flag or GFP antibodies. (**F**, **G**) In vitro binding assay analysis of the direct interaction between ECHS1 and NSP3$^{1\text{-}1491}$ or NSP3$^{803\text{-}1334}$. Samples were analyzed by SDS-PAGE and immunoblotting with NSP3, ECHS1 or GST antibodies, with $n = 3$ independent biological replicates. (**H**) Cells were transfected with GFP-NSP3 and treated with the proteasome inhibitor MG132, lysosomal inhibitors chloroquine (CQ) or bafilomycin A1 (Baf-A1), with or without CHX, for 10 h. The lower panel quantifies relative GFP-NSP3 expression. Data are presented as mean ± SD ($n = 3$ independent biological replicates); P values were calculated by one-way ANOVA. (**I**) Immunoblotting analysis of GFP-NSP3 stability (WT and K1000R/K977R/K963R/K739R ubiquitination site mutants) in 293T cells treated with cycloheximide (CHX) for 10 h. Source data are available online for this figure.

© The Author(s)
