# Supplementary material for: Disruption of ER-mitochondria contact sites by coronavirus replication organelles sustains viral replication via NSP3 stabilization
Source: EMBO J. 2026 May 28;45(13):4379–416. doi: 10.1038/s44318-026-00816-x (PMC13323368; doi:10.1038/s44318-026-00816-x)
Supplement: Supplementary file 3 — Peer Review File [file 44318_2026_816_MOESM3_ESM.pdf]

THE EMBO JOURNAL

Disruption of ER-mitochondria contact sites by coronavirus replication organelles sustains viral replication via NSP3 stabilization

Xinyue Zheng, Liqiao Hu, Xubing Long, Zhilei Zhao, Rongrong Chen, Rong Bai, Buyun Tian, Maoge Zhou, Binbin Ding, Tao Xu, and Zonghong Li

Corresponding author(s): Zonghong Li (li_zonghong@gzlab.ac.cn) , Tao Xu (xutao@ibp.ac.cn), Binbin Ding (ding_binbin@gzlab.ac.cn), Maoge Zhou (zhou_maoge@gzlab.ac.cn)

Review Timeline:

Submission Date:	7th Nov 25
Editorial Decision:	19th Dec 25
Appeal Received:	11th Feb 26
Editorial Decision:	17th Mar 26
Revision Received:	27th Apr 26
Accepted:	6th May 26

Editor: Ieva Gailite

Transaction Report:

(Note: With the exception of the correction of typographical or spelling errors that could be a source of ambiguity, letters and reports are not edited. Depending on transfer agreements, referee reports obtained elsewhere may or may not be included in this compilation. Referee reports are anonymous unless the Referee chooses to sign their reports.)

1st Editorial Decision **19th Dec 2025**

Dear Dr. Li,

Thank you for submitting your manuscript for consideration by The EMBO Journal. We have now received comments from three reviewers, which are included below for your information. Based on these comments, we unfortunately had to conclude that the study is not a sufficiently strong candidate for publication in The EMBO Journal.

As you can see, while reviewers #2 and #3 with expertise in virology and viral replication organelle formation find the study of interest, reviewer #1 with expertise in organelle contact site formation and their viral regulation raises substantive concerns regarding the causality of NSP3/NSP4 impact on ER-mitochondrial contact site (ERMC) formation vs general mitochondrial defect induction. Furthermore, the reviewer raises concerns regarding the lack of appropriate controlling for NSP3/4 expression levels and the general reliance on overexpression experiments. He/she also indicates that a better exploration of the role of ECHS1 and RBBP6 would be needed, and highlights concerning instances where interaction experiments with NSP3 do not lead to the expected results. While reviewer #2 is more positive in the general tone of their assessment, he/she raises similar concerns regarding the ERMC defect causality, characterisation of NSP3 interactions with ECHS1 and RBBP6, and the relevance of the proposed model in a physiological, non-overexpression context. Since these issues affect the main conclusions of the study and major experimental revision with an uncertain outcome would be needed to address them, I am afraid that we cannot offer to invite a revised manuscript.

Nevertheless, if you find that you can address the main concerns raised by the reviewers, I would be willing to reconsider the revised manuscript. In this case, it means that I would send it back to the same reviewers, if possible, but would allow them to make new comments on the data, which might then have to be further addressed if the reviewers are more positive in this round of assessment.

Thank you in any case for the opportunity to consider this manuscript. I am sorry that I could not communicate more positive news, but I nevertheless hope that you will find our reviewers' comments helpful for further improvement of the manuscript.

With kind regards,

Ieva Gailite

Ieva Gailite, PhD
Senior Scientific Editor
The EMBO Journal
Meyerhofstrasse 1
D-69117 Heidelberg
Tel: +4962218891309
i.gailite@embojournal.org

****

Referee #1:

The manuscript by Zhen and colleagues reports that coronavirus infection induces mitochondrial structural changes and destabilization of ER-mitochondria contact sites (ERMCS), and that overexpression of the non-structural viral proteins NSP3 and NSP4 similarly causes mitochondrial alterations and ERMCS destabilization. The authors further propose that ECHS1 is released from damaged mitochondria and regulates ubiquitination of NSP3 in NSP3-overexpressing cells.

While the impact of coronavirus infection on mitochondria and on ER-mitochondria contact sites, including the effects of several SARS-CoV-2 accessory and structural proteins, has already been documented, the main claimed novelty of this study is that NSP3/NSP4 expression alone is sufficient to induce mitochondrial alterations and ERMCS destabilization. However, these conclusions are not convincingly supported, primarily because several key controls are missing, as detailed below.

DMV formation impairs mitochondrial morphology and function
The relationships between NSP3/NSP4 expression and the reported mitochondrial anomalies are not defined in terms of temporal sequence (for how long were NSP3 and NSP4 overexpressed, and when did the mitochondrial changes occur?) or concentration dependence on NSP3/NSP4. The authors interpret the mitochondrial changes induced by NSP3/NSP4 overexpression as a consequence of ERMC destabilization; however, the predicted morphological change would be mitochondrial elongation, a phenotype not reported in the manuscript, and a similar uncertainty applies to the impaired mitochondrial structural integrity in SARS-CoV-2-infected Vero cells (8 hpi).

ERMCs play a critical role in DMV-induced mitochondrial damage and coronavirus replication
The authors justify their focus on ERMCs based on decreased transcription of ERMCS tethers in NSP3/NSP4-overexpressing cells, but the mechanistic basis for this transcriptional repression is neither addressed nor discussed. TEM analysis shows reduced ERMCs in NSP3/NSP4-expressing cells, but, as noted above, there is no information on the temporal or dose-dependent relationships between NSP3/NSP4 levels and ERMCs, and if ERMCs are evaluated only in cells already displaying abnormal mitochondrial structure, ERMC destabilization may simply be an epiphenomenon of altered mitochondria rather than a primary event. According to the authors' working model, ERMCs destabilization should precede mitochondrial structural alterations, yet no time-resolved analysis of mitochondrial changes in NSP3/NSP4 -expressing or infected cells is provided.
The observations in cells stably overexpressing ERMC inducers (MFN2 and/or RMDN3) should be interpreted with caution, because these clones are not controlled or matched for NSP3/NSP4 transfection or expression levels, so the reduced DMV number may simply reflect lower NSP3/NSP4 expression. The same concern applies to experiments using the synthetic OMM-ER tethering linker, which is transiently transfected; quantification of NSP3/NSP4 levels in cells expressing the ERMC stabilizer is essential, and in infected cells a more rigorous analysis of how each manipulation affects virus entry is needed, with infection levels assessed in all clones to ensure that the observed effects mainly concern replication.

Enforcing ER-mitochondria tethering restricts coronavirus infection
The impact of enforced ER-mitochondria tethering on coronavirus infection is assessed only at 24 hpi (Fig. 4F), and it is unclear why the same conditions were not examined at 8 hpi. Including an earlier time point would help distinguish effects on virus entry or early replication from effects on later stages of the viral cycle, especially given that the authors use stable clones expressing single or combined ERMCS stabilizers, each of which may differ in susceptibility to virus entry.

ECHS1 released from damaged mitochondria is essential for DMV formation and coronavirus infection
ECHS1 release from damaged mitochondria is proposed to be essential for coronavirus infection, evaluated at 20 hpi, whereas at 8 hpi there is no difference in viral protein expression with or without ECHS1 (Fig. 5B,C). This contrasts with Fig. 5F, where no DMVs are observed at 8 hpi, raising concerns about the reproducibility and representativeness of the selected cells and conditions. The level of infection in ECHS1-KO HeLa cells also needs to be documented, including whether they are as permissive as WT cells for virus entry and whether ACE2 expression levels (introduced by transfection) are comparable between WT and ECHS1-KO clones.

ECHS1 stabilizes NSP3 by blocking its ubiquitination at K963
Most experiments on the role of ECHS1 in NSP3 regulation are carried out in cells overexpressing only GFP-NSP3. Given that NSP3 is tightly associated with NSP4 and its stability is strongly influenced by NSP3/NSP4 complex formation, NSP3 expressed without its NSP4 partner is expected to have a shorter half-life, so these experiments should be repeated in cells co-expressing equimolar amounts of NSP3 and NSP4 to better reflect the physiological replication complex.

E3 ubiquitin ligase RBBP6 mediates the NSP3 polyubiquitination and restricts coronavirus replication
In the section on the E3 ubiquitin ligase RBBP6, which mediates NSP3 polyubiquitination and restricts coronavirus replication, the experiments are again performed in cells expressing GFP-NSP3 alone. For the reasons outlined above, these assays should be repeated in cells co-expressing equimolar amounts of NSP3 and NSP4.

Specific comments
Fig. 6D: The authors should test the effect of MG132 also in the absence of CHX.
Fig. 6G: The authors claim that the mutant NSP3 K963R has a different half-life in ECHS1-KO cells compared with WT NSP3 and that its half-time is more similar to that of WT NSP3 in control cells; however, the blot shows a decline of both WT and K963R starting at 6 h, whereas WT NSP3 in control cells displays a clear decline only at 10 h, and the plotted data do not accurately represent these patterns.
Fig. 7B: There appears to be no effective IP for NSP3, as there is no enrichment of NSP3 in the IP lane compared with the input lane.
Fig. 7C: The authors should quantify the efficiency of triple transfection and report how many cells co-express all three proteins (GFP-NSP3, RBBP6-FLAG, and ECHS1-GFP).

Fig. S6A: Multiple inconsistencies are present; the blot is intended to show co-IP of NSP3 with ECHS1 in NSP3/NSP4-overexpressing cells, yet there is no enrichment of NSP3 in the NSP3 IP, and NSP4 is absent from the NSP3 IP despite being the main NSP3 interactor.
Fig. S6C: The images are of low quality, with GFP-NSP3 displaying diffuse reticular signal and no discernible puncta, making it impossible to judge co-localization with punctate ECHS1.
Fig. S6D: The blot is puzzling, as there is no enrichment of NSP3 in the IP for truncated NSP3 (1-1491), calling into question the validity of this interaction assay.

Referee #2:

General summary and opinion about the principal significance of the study, its questions and findings

In this manuscript by Zheng and Hu et al., the authors propose a positive feedback mechanism during coronavirus infection by hypothesizing that coronavirus infection disrupts endoplasmic reticulum (ER)-mitochondria contact sites (ERMCS), leading to stabilization of a critical viral protein NSP3 essential for viral replication. Specifically, the authors demonstrate that DMV formation damages ERMCS, which releases the mitochondrial matrix protein ECHS1. ECHS1 in turn interacts with and stabilizes NSP3, thereby sterically preventing its ubiquitin-dependent degradation by the E3 ligase RBBP6. Enhanced ER-mitochondria tethering or blocking ECHS1 binding of NSP3 decreases NSP3 steady state levels, leading to a decrease in DMV formation, genome replication, and subsequent infection. Collectively, the findings of this paper have the potential to provide a novel mechanism for the importance of ERMCS for DMV formation during coronavirus infection.
The authors demonstrate these findings through a variety of orthogonal methods and techniques. However, despite this, many of the primary data and associated phenotypes that are crucial to support these claims are not entirely convincing and weak. Because of this, it remains unclear whether the proposed mechanism is what drives this positive feedback mechanism. We provide specific (major and minor) comments to strengthen this article.

Specific major concerns essential to be addressed to support the conclusions

1. Further characterize the mechanism proposed of how DMV formation leads to ERMCS disruption:
- In order to determine if ERMCS disruption is a direct consequence of DMV formation, under NSP3+4 overexpression or during infection, inhibit DMV formation to test if ERMCS disruption no longer occurs.
- Are there any defects in mitochondria damage or ERMCS earlier in infection, prior to DMV formation?
- Is the defect in ERMCS during DMV formation an artifact of an increase mitochondria fragmentation or less total mitochondria? Are there less mitochondria overall when NSP3+4 is expressed (Fig. 1C).
- Are cells normally functioning under MFN2 and RMDN3 overexpression. Is viral entry unaffected?
- Is the ER and mitochondria morphology normal in ECHS1 KO cells?
- In the infection ESCHS1 rescue experiments (Fig. 5, Fig. S4J), is the ER and mitochondria morphology (including number of mitochondria and number of ERMCS-readouts used in Fig. 1B-C) normal in the ECHS1 KO cells and is that lost in the ESCH1 rescue condition?

2. Does treatment of B-A/I or the addition of the OMM-ER tether de-stabilize NSP3 expression?

3. For Fig. S4H-I, in order to properly rule out that NSP8 does not translocate ECHS1 into the cytosol, the expression levels of NSP3+4 and NSP8 should be relatively equal (data not shown). Further does ECHS1 stabilize other viral proteins (NSP3 and 4 were tested, Fig. 6A-C and Fig. S5)?

4. Perform the ECHS1 and NSP3 (Fig. S6A), and RBBP6 and NSP3 (Fig. 7B-C) co-IPs during infection.

5. Despite the statistically significant p-values, there are key phenotypes in the paper which have subtle changes and the biological significance of change is not convincing. Plasmid expression of SARS-CoV-2 NSP3 is used to build the proposed model in the paper, but out of the infection data, SARS-CoV-2 had weaker and less significant phenotypes compared to the other coronaviruses. Are there any key differences in NSP3 between SARS-CoV-2, MHV and WIV1 that would explain this?
- Fig. 1D-E Is this a meaningful increase in ROS? (should this be a supplemental figure or not included in the paper)
- Fig. S1C, F and G
- Fig. S2B-E and G
- Fig. S3, in particular the infection data E-H.
- Fig. 3C
- Fig. 4D-I, and K-L: based on the Y-axis, the fold changes are less than 10 which is not a significant decrease in overall infection. In particular for SARS-CoV-2 (F and I)

Minor concerns that should be addressed

6. Some immunofluorescence images appear to be oversaturated. The subcellular localization of NSP3 and 4 appear to be all over the cell with NSP4 being nuclear in some images but not in others. Fig. 1D and S6C should be corrected.
- S1G it is difficult to see the colocalization with the colors provided.

7. Lower exposure Fig. S2B blot.

8. Data in S6A-B are confusing.
- Line 270, Fig. S6A does not show a "direct" interaction of NSP3 and ECHS1. Why does NSP4 not appear in the IP since NSP4 and 3 should be interacting?
- Fig. S6B: why does NSP3 colocalize with ECHS1 when ECSH1 is at the mitochondria? Based on the model, NSP3+4

expression together (or DMV formation) induces a release of ECHS1 from the mitochondria to then interact with and stabilize NSP3.

9. Fig. 4J-L and Fig. 7G-I have different viral proteins for each virus as a readout, the viral proteins should be kept the same (or equivalent) across viruses.
- Fig. 7 G-I, Check if RBBP6 knockdown condition had less normal mitochondria and ERMCS.

10. How is there an increase in total intracellular ECHS1 (Fig. S4E) as the MOI increases? The suggested model is that there is an increase of ECHS1 into the cytosol from the mitochondria. Doesn't match the data in S4F and G.

11. Fig. 6H quantification lane labeling appears to be incorrect.

12. Why does RBBP6 overexpression in Fig. 7C not lead to a decrease in steady state NSP3 in the input? As in 7D.

Referee #3:

In this study, Zheng and colleagues corroborate earlier observations that SARS-CoV-2 infection causes mitochondrial damage. Going beyond that, the authors decipher the so far unknown underlying mechanism by employing a series of complementary approaches, ranging from light and electron microscopy up to knock-out cell lines, infection experiments and biochemical studies. The authors show that the ER - mitochondria membrane contact site disruption is caused by the viral proteins nsp3 and nsp4 that are sufficient to induce double membrane vesicles, the sites of viral RNA synthesis. Overexpression of proteins enforcing ER - mitochondria tethering reduces this disruption and impairs DMV formation, arguing that ER - mitochondria contact site disruption is favorable to viral RNA replication, which is also shown by the authors. In a search for the underlying mechanism, Zheng and coworkers perform transcriptome and proteome analysis, the latter with cytosolic and mitochondrial fractions and identify ECHS1 as host cell factor being released from mitochondria in infected cells. Using cycloheximide-based protein stability analysis, the authors find that ECHS1 stabilizes nsp3 by blocking its ubiquitination at lysine residue 963. The authors find that ECHS1 competes with the E3 ubiquitin ligase RBBP6 for binding to nsp3, thus blocking nsp3 ubiquitination and proteasomal degradation. Thus, RBBP6 restricts viral replication which is counteracted by the virus-induced release of ECHS1 from mitochondria. This applies to different coronaviruses, shown here for mouse hepatitis virus, SARS-CoV-2 and the Bat sarbecovirus WIV1-CoV.
This is a very detailed and comprehensive study providing convincing data on the role of ECHS1 in regulating nsp3 stability and thus, DMV formation as well as viral RNA replication. The authors have used an impressively broad approach and provide very complementary data supporting their hypothesis. This mechanism appears to operate for different coronaviruses, although the magnitude of phenotype differs substantially, depending on the individual coronavirus. This study discloses a hitherto unknown mechanism, which will be of broad interest and a nice example of viral hijacking of mitochondria to promote viral replication. I have only a few rather minor comments.
• Line 84: several references given here do not deal with coronaviruses but rather with other viruses (e.g. HBV, dengue virus).
• Please describe in a bit more detail used cell lines and virus strains as well as the origin of the viruses. Also provide the full name of the viruses when mentioned for the first time in the text.
• Figure 1A: Is this CLEM? If not, how sure are the authors that each analyzed cell has been transfected and expresses the given nsp? What is the transfection efficiency? These aspects need to be explained.
• Figure 1A: Why showing the color-coding only in some, but not all magnified TEM examples? The same applies to the TEM panels in other figures.
• Figure 1D: The increase indicated in the quantification does not show up in the displayed IF images. Can the authors provide better images? Also please consider to show the mitoSOX channel in gray scale to enhance the contrast. The same applies to Figure 2E.
• Figure 2B: Regarding the statistical analysis, the most relevant comparison here is Ctrl vs. MFN2+RMDN3 OE. Please indicate to show whether there is a (near complete) rescue.
• Figure 2F: This panel is super small and one cannot appreciate what the authors want to show, even when zooming in on the screen.
• Legend to Figure 4G-I: There is no 5 µM concentration shown.
• Figure 5A: Does the control also express HA-tagged ECHS1 (as one would assume from the WB labeling) or is it the non-tagged endogenous protein?
• Figure 6D: Please provide quantification of gfp-nsp3.
• Figure 7: The authors should consider to include an alpha-fold model for the nsp3-ECHS1 interaction which might support their shielding hypothesis.
• The statement of ERMCs as regulatory node is a bit farfetched, because it indicates an active antiviral role. Instead, the data indicates a competition for ER capacity between DMV formation and ERMCs. Under normal infection conditions, DMV formation overrules ERMCs, which is reversed under Mfn2 agonist or overexpression conditions.
• Figure 7F: How do the authors explain the low fluorescence signal of nsp4, when nsp3 is the ubiquitination target?
• Figure 7 G-I: You cannot measure a protein by qPCR; it is RNA abundance. Please adjust the labeling.
• Please specify in every figure legend, in which cell line the respective experiment was performed (especially in figure 7 this

information is lacking).
• The manuscript requires careful editing to correct several typos and errors in grammar.

** As a service to authors, EMBO Press provides authors with the possibility to transfer a manuscript that one journal cannot offer to publish to another EMBO publication or the open access journal Life Science Alliance launched in partnership between EMBO Press, Rockefeller University Press and Cold Spring Harbor Laboratory Press. The full manuscript and if applicable, reviewers' reports, are automatically sent to the receiving journal to allow for fast handling and a prompt decision on your manuscript. For more details of this service, and to transfer your manuscript please click on Link Not Available. **

Please do not share this URL as it will give anyone who clicks it access to your account.

Response Letter

Manuscript number: EMBOJ-2025-122983

Title: A self-reinforcing DMV-mitochondria cycle orchestrated by ERMCSs and ECHS1 drives coronavirus replication.

Dear Editor and Reviewers,

We appreciate the opportunity to revise our manuscript and are deeply grateful for the insightful comments from the reviewers. Your detailed feedback has not only helped us identify critical gaps in the manuscript but also provided clear, valuable guidance for strengthening its scientific rigor, completeness, and biological significance. Each comment is highly pertinent and thought-provoking, ranging from clarifying the temporal and dose-dependent relationships between NSP3/NSP4 expression, ERMC disruption, and mitochondrial damage, to recommending the supplementation of critical control experiments, verification of protein interactions under physiological conditions, and optimization of data presentation.

We have carefully reviewed and addressed each of your comments in detail, and comprehensive revisions have been made accordingly. A point-by-point response to all reviewer suggestions is provided below. We have systematically addressed all the reviewers' proposed revisions, including supplementation of time-course and dose-response experiments, repetition and optimization of co-IP and immunofluorescence assays to improve data quality, correction of formatting and labeling errors in the figures, and quantification of key parameters to strengthen the robustness of our conclusions.

We believe these revisions have significantly enhanced the manuscript's scientific rigor, data completeness, and clarity of presentation.

Once again, we would like to extend our heartfelt thanks to both reviewers and the editorial team for their valuable support and guidance throughout this process.

For Referee #1:

1.DMV formation impairs mitochondrial morphology and function

The relationships between NSP3/NSP4 expression and the reported mitochondrial anomalies are not defined in terms of temporal sequence (for how long were NSP3 and NSP4 overexpressed, and when did the mitochondrial changes occur?) or concentration dependence on NSP3/NSP4. The authors interpret the mitochondrial changes induced by NSP3/NSP4 overexpression as a consequence of ERMC destabilization; however, the predicted morphological change would be mitochondrial elongation, a phenotype not reported in the manuscript, and a similar uncertainty applies to the impaired mitochondrial structural integrity in SARS-CoV-2-infected Vero cells (8 hpi).

R: we sincerely appreciate your insightful comment regarding the temporal, concentration-dependent relationships between NSP3/NSP4 expression, DMV biogenesis, and mitochondrial anomalies, as well as the clarification of mitochondrial phenotypic changes.

We utilized a doxycycline (DOX)-inducible NSP3/NSP4 co-expression system (TetOn 3G-mCherry-NSP3-T2A-GFP-NSP4 in HeLa cell) to track dynamic changes:

- NSP3/NSP4 expression was undetectable at 0 h post-induction, accumulated gradually over 12–36 h (**Fig. S1A**), and DMVs were first observed at 12 h (**Figs. S1B-C**).
- Mitochondrial structural damage (e.g., vacuolization, cristae collapse) was not detected until 24–36 h (**Fig. S1E**), confirming mitochondrial impairment is a delayed consequence of DMV accumulation (not immediate NSP3/NSP4 expression).

In viral infection models (MHV/SARS-CoV-2), DMVs were detected at 4 hpi (MHV) or 6 hpi (SARS-CoV-2), but mitochondrial structure remained normal at these early time points; damage emerged at 8 hpi (coinciding with progressive DMV accumulation (**Figs S2A-B, D-F, H**).

For concentration dependence : we transfected increasing doses of NSP3/NSP4 plasmids (1 μg, 3 μg, 4 μg per protein) and found:

- Higher doses correlated with greater DMV abundance (consistent with our previous report, Yang et al, 2025, J Cell Biol, 3;224(3):e202306101) and more severe mitochondrial abnormalities (**Figs. 1A-B, D-E**), validating dose-dependent DMV accumulation drives progressive mitochondrial damage.

The time-course infection experiments revealed a dynamic shift in mitochondrial morphology: at 8 hpi, mitochondria exhibited marked elongation, whereas by 24 hpi or later, their morphology transitioned to a rounded (Figure R1). This observation of rounded mitochondria at later time points is further corroborated by our recent large-volume electron microscopy data from MHV-infected cells at 20 hpi (doi.org/10.1016/j.scib.2026.02.011).

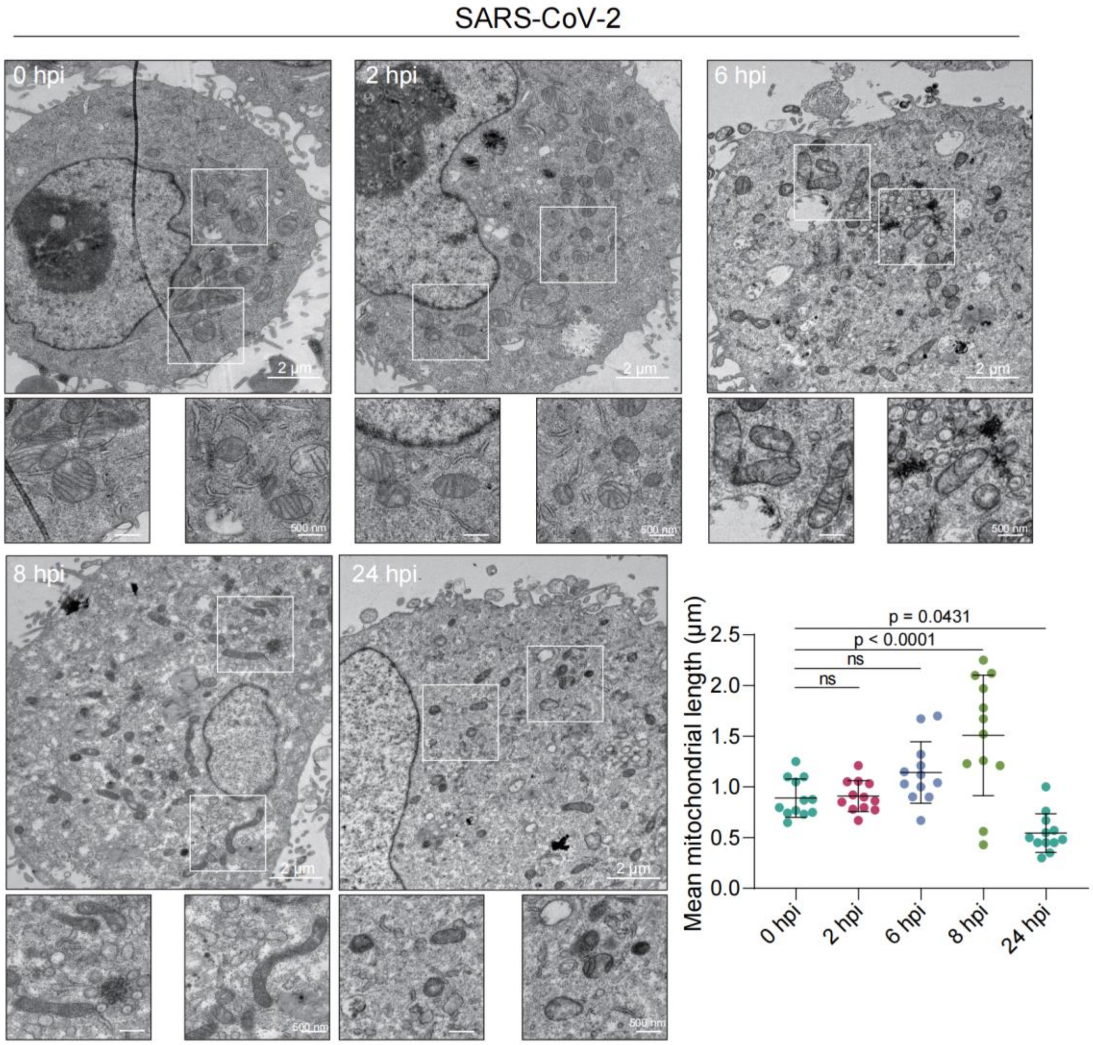

Figure R1 Time-course quantification of mean mitochondrial length in SARS-CoV-2-infected cells, where each data point represents an individual cell.

2. ERMCs play a critical role in DMV-induced mitochondrial damage and coronavirus replication

The authors justify their focus on ERMCs based on decreased transcription of ERMCS tethers in NSP3/NSP4-overexpressing cells, but the mechanistic basis for this transcriptional repression is neither addressed nor discussed. TEM analysis shows reduced ERMCs in NSP3/NSP4-expressing cells, but, as noted above, there is no information on the temporal or dose-dependent relationships between NSP3/NSP4 levels and ERMCs, and if ERMCs are evaluated only in cells already displaying abnormal mitochondrial structure, ERMC destabilization may simply be an epiphenomenon of altered mitochondria rather than a primary event. According to the authors' working model, ERMCs destabilization should precede mitochondrial structural alterations, yet no time-resolved analysis of mitochondrial changes in NSP3/NSP4 -expressing or infected cells is provided.

R: we sincerely thank the reviewer for this insightful and constructive comment.

The reviewer raises an important question concerning the mechanism underlying the transcriptional downregulation of ERMCS tether proteins. Indeed, downregulation of ERMCS tether proteins is a common phenotypic response to viral infection, as observed with HCoV-OC43, hepatitis C virus, influenza virus, and flaviviruses. However, the detailed mechanism driving this transcriptional downregulation remains largely unknown. The reviewer's suggestion points to a valuable direction for future research, namely identifying key transcriptional or post-transcriptional regulators that may serve as promising broad-spectrum antiviral targets.

We have incorporated relevant discussion on this point in the revised manuscript, as follows:
"Recent studies have shown that infection with coronavirus HCoV-OC43 leads to marked reductions in nearly all ERMCSs proteins, correlating with disrupted ER and mitochondrial network architecture and impaired inter-organellar communication (Cook et al, 2022). Similar ERMCSs disruption has been reported during infection with hepatitis C virus, influenza virus, and flaviviruses (Pila-Castellanos et al, 2021; Cook et al, 2022; Freppel et al, 2025). Together, these findings suggest that ERMCSs are a common target during viral infection, and that enhancing ERMCSs formation may represent a broadly applicable antiviral strategy. However, the precise mechanism underlying the transcriptional downregulation of ERMCS components remains unclear. Previous work suggests that early viral gene products are responsible for suppressing the expression of ERMCS proteins during infection (Cook et al, 2022). Our results advance this understanding by demonstrating that the viral proteins NSP3 and NSP4 act as direct transcriptional repressors of ERMCS components. Thus, future work to identify the intermediary transcriptional or post-transcriptional regulators that mediate the effect of NSP3 and NSP4 co-expression on ERMCSs may reveal promising broad-spectrum antiviral targets."

For temporal/dose-dependent relationships between NSP3/NSP4 levels and ERMCs :

- Temporal : In the DOX-inducible system, ER-contacted mitochondria proportions began to decrease at 12 h (early DMV formation) and declined progressively over 12-36 h (correlating with DMV accumulation, **Figs. S1B, D**).
- Dose-dependent : Higher levels of NSP3/NSP4 enhanced DMV formation and exacerbated ERMC loss (**Figs. 1D, F)**.

To clarify whether ERMC destabilization is a primary event (not an epiphenomenon of mitochondrial alteration) :

- Temporal analysis shows ERMC loss occurs at 12 h (early DMV formation), whereas mitochondrial damage emerges at 24 h (**Figs. S1D-E**), demonstrating ERMC destabilization precedes mitochondrial structural changes.
- Pharmacological (RDV) or genetic (NSP4 H120N/F121L mutant) inhibition of DMV

formation restored ERMCs (**Figs. S3A-B, D-E, S3G-H**), confirming ERMC loss is DMV-driven (not a byproduct of mitochondrial damage).

For time-resolved analysis of mitochondrial changes : the DOX-inducible system and infection models provide time-resolved data: ERMC loss (12 h post-induction/4-6 hpi) - DMV accumulation (12-24 h post-induction/6-8 hpi) - mitochondrial damage (24-36 h post-induction/8 hpi) (**Figs. S1-S3**), validating our proposal that ERMC destabilization precedes mitochondrial alteration.

3. The observations in cells stably overexpressing ERMC inducers (MFN2 and/or RMDN3) should be interpreted with caution, because these clones are not controlled or matched for NSP3/NSP4 transfection or expression levels, so the reduced DMV number may simply reflect lower NSP3/NSP4 expression. The same concern applies to experiments using the synthetic OMM-ER tethering linker, which is transiently transfected; quantification of NSP3/NSP4 levels in cells expressing the ERMC stabilizer is essential, and in infected cells a more rigorous analysis of how each manipulation affects virus entry is needed, with infection levels assessed in all clones to ensure that the observed effects mainly concern replication.

R: we sincerely appreciate your insightful comments and constructive suggestions, which have helped us improve the rigor of our study. We have made revision with additional experiments and data, as outlined below:

(1) NSP3/NSP4 expression levels are not altered by ERMC stabilization.
We performed western blotting to quantify NSP3 and NSP4 expression in our experimental systems:

- In stable cell lines overexpressing MFN2, RMDN3, or their combination, we observed no significant difference in GFP-NSP3 or NSP4-mCherry expression compared to the vector control (**Fig. 2A**).
- Similarly, expression of the synthetic OMM-ER tether linker did not affect NSP3 or NSP4 expression (**Fig. 2B**).
- Treatment with the MFN2 agonist B-A/I also had no impact on NSP3 or NSP4 expression. (**Figs. S5I-J**).

These results confirm that the reduced DMV number and mitochondrial protection we observed are not due to lower NSP3/NSP4 expression.

(2) Viral entry is unaffected by ERMC stabilization.
To rule out effects on viral entry, we quantified viral genomic RNA (gRNA) levels at 2 hpi for MHV, WIV1, and SARS-CoV-2.

- As shown in **Figures 4A–C**, there were no significant differences in viral gRNA levels between control cells and cells overexpressing ERMC inducers at 2 hpi. This confirms that our observed effects on viral replication are not due to altered viral entry.

(3) Cell function remains normal under MFN2/RMDN3 overexpression.

- Our stable cell lines showed normal morphology and proliferation under standard culture conditions.
- The lack of effect on viral entry (2 hpi data) further indicates that basic cellular functions required for viral infection are not impaired by MFN2/RMDN3 overexpression.

4.Enforcing ER-mitochondria tethering restricts coronavirus infection

The impact of enforced ER-mitochondria tethering on coronavirus infection is assessed only at 24 hpi (Fig. 4F), and it is unclear why the same conditions were not examined at 8 hpi. Including an earlier time point would help distinguish effects on virus entry or early replication from effects on later stages of the viral cycle, especially given that the authors use stable clones expressing single or combined ERMCS stabilizers, each of which may differ in susceptibility to virus entry.

R: thank you for this insightful suggestion to examine earlier time points to distinguish effects on different stages of the viral life cycle. We performed a time-course qPCR analysis that included 2 hpi, 8 hpi, and 24 hpi time points for all three viruses (MHV, WIV1, SARS-CoV-2).

- At 2 hpi, viral genomic RNA levels were comparable across all cell lines (“ns” in **Fig. 4A-C**), confirming that enforced ER-mitochondria tethering does not affect viral entry.
- At 8 hpi, we observed significant suppression of viral gRNA accumulation in cells overexpressing MFN2, RMDN3, or their combination, demonstrating that the inhibitory effect begins during the early replication phase.
- This inhibition was further enhanced at 24 hpi, which correlated with the reduction in viral titers shown in **Figures 4D-F**.

5.ECHS1 released from damaged mitochondria is essential for DMV formation and coronavirus infection

ECHS1 release from damaged mitochondria is proposed to be essential for coronavirus infection, evaluated at 20 hpi, whereas at 8 hpi there is no difference in viral protein expression with or without ECHS1 (Fig. 5B,C). This contrasts with Fig. 5F, where no DMVs are observed at 8 hpi, raising concerns about the reproducibility and representativeness of the selected cells and conditions. The level of infection in ECHS1-KO HeLa cells also needs to be documented, including whether they are as permissive as WT cells for virus entry and whether ACE2 expression levels (introduced by transfection) are comparable between WT and ECHS1-KO clones.

R: We sincerely appreciate your careful observation and constructive questions regarding the role of ECHS1 in DMV formation and viral infection.

- It appears our labeling or presentation may not have been sufficiently clear, leading to this misunderstanding. Our time-course qPCR results (**Figs. 5B-C**) demonstrate that viral gRNA accumulation was significantly reduced in ECHS1 KO cells as early as 8 hpi ($p=0.0089$ for SARS-CoV-2 and $p=0.0023$ for MHV at 8 hpi). This confirms that ECHS1 deficiency impairs viral replication starting from the replication phase, which aligns well with its essential role in DMV formation (**Figs. 5G, K**).

- ACE2 expression: We confirmed that hACE2 expression levels are comparable between Ctrl and *ECHS1*-KO and *ECHS1*-Rescue HeLa cells via western blotting (**Fig. 5A**), ensuring that differences in viral infection are not due to altered ACE2 expression.

6. ECHS1 stabilizes NSP3 by blocking its ubiquitination at K963
Most experiments on the role of ECHS1 in NSP3 regulation are carried out in cells overexpressing only GFP-NSP3. Given that NSP3 is tightly associated with NSP4 and its stability is strongly influenced by NSP3/NSP4 complex formation, NSP3 expressed without its NSP4 partner is expected to have a shorter half-life, so these experiments should be repeated in cells co-expressing equimolar amounts of NSP3 and NSP4 to better reflect the physiological replication complex.

R： thank you for this insightful and constructive suggestion. We fully agree that validating the

findings in the physiological context of NSP3 and NSP4 co-expression is critical to strengthening our conclusions. We also evaluated the impact of *ECHS1* knockout on GFP-NSP3-T2A-NSP4 in these cells.

- Regardless of whether NSP4 was co-expressed or not, we observed accelerated NSP3 decay in *ECHS1*-KO cells (**Figs. 6C-D**). Importantly, NSP4 expression levels remained unchanged and were not affected by ECHS1 status.

7. E3 ubiquitin ligase RBBP6 mediates the NSP3 polyubiquitination and restricts coronavirus replication
In the section on the E3 ubiquitin ligase RBBP6, which mediates NSP3 polyubiquitination and restricts coronavirus replication, the experiments are again performed in cells expressing GFP-NSP3 alone. For the reasons outlined above, these assays should be repeated in cells co-expressing equimolar amounts of NSP3 and NSP4.

R: thank you for this insightful suggestion to validate our findings in a more physiological NSP3/NSP4 co-expression system. As recommended, we have now performed the assays in cells co-expressing NSP3 and NSP4 using the GFP-NSP3-T2A-NSP4 construct (**Figures 7E-F**).

- Overexpression of RBBP6 significantly reduced NSP3 protein levels in both control and CHX-treated cells, consistent with the findings in cells expressing GFP-NSP3 alone.
- Knockdown of RBBP6 increased NSP3 expression under identical conditions.

These consistent findings across both expression systems reinforce our conclusion that RBBP6 functions as an E3 ubiquitin ligase targeting NSP3 for polyubiquitination.

Specific comments

Fig. 6D: The authors should test the effect of MG132 also in the absence of CHX.

R: thank you for your constructive suggestion. As recommended, we tested MG132 treatment

alone (**Figure 6E**), which revealed that MG132 alone elevated basal GFP-NSP3 expression, further confirming that NSP3 undergoes constitutive proteasomal degradation.

Fig. 6G: The authors claim that the mutant NSP3 K963R has a different half-life in ECHS1-KO cells compared with WT NSP3 and that its half-time is more similar to that of WT NSP3 in control cells; however, the blot shows a decline of both WT and K963R starting at 6 h, whereas WT NSP3 in control cells displays a clear decline only at 10 h, and the plotted data do not accurately represent these patterns.

R: thank you for your constructive feedback. We have repeated this assay and conducted quantitative analysis, with the updated results shown in **Figure 6G and H**. Our new data show the K963R mutant has a longer half-life than WT NSP3 in *ECHS1*-KO cells, supporting our original conclusion that K963 is critical for ECHS1-mediated NSP3 stabilization. Updated quantification (**Fig 6H**) confirms these kinetic differences

Fig. 7C: The authors should quantify the efficiency of triple transfection and report how many cells co-express all three proteins (GFP-NSP3, RBBP6-FLAG, and ECHS1-GFP).

R: we greatly appreciate your valuable suggestion.

- We have performed additional experiments via immunofluorescence staining to quantify the efficiency of triple transfection and the proportion of cells co-expressing all three proteins (GFP-NSP3, RBBP6-FLAG, and ECHS1-BFP). Based on our previous experimental experience, as RBBP6 typically exhibits lower expression levels than ECHS1, we optimized the plasmid ratio used in co-transfection as GFP-NSP3: RBBP6-FLAG: ECHS1-BFP = 2:1.5:1. Our results, presented in the revised **Fig. 7C** Figure legend, show that in the triple transfection group, approximately 18–20% of cells co-expressed all three proteins. (with the proportion of GFP-NSP3 and RBBP6-Flag double-positive cells being ~20%, and the proportion of GFP-NSP3 and ECHS1 double-positive cells being ~20%).

Fig. S6A: Multiple inconsistencies are present; the blot is intended to show co-IP of NSP3 with ECHS1 in NSP3/NSP4-overexpressing cells, yet there is no enrichment of NSP3 in the NSP3 IP, and NSP4 is absent from the NSP3 IP despite being the main NSP3 interactor.

R: thank you for this critical question regarding the interaction analysis in Fig. S6A (revised **Fig. S9A**).

- Based on our laboratory experience, NSP4 is temperature-sensitive, and our original high-temperature boiling step (standard for IP) led to the loss of its signal. We repeated the co-IP with a milder boiling condition at 55 °C, and a clear NSP4 signal was successfully detected in the NSP3 IP samples under this condition.

Fig. S6C: The images are of low quality, with GFP-NSP3 displaying diffuse reticular signal and no discernible puncta, making it impossible to judge co-localization with punctate ECHS1.

R: we greatly appreciate this insightful question, which prompted a re-evaluation of ECHS1 colocalization with NSP3 and NSP4.

- Initial Pearson correlation analysis suggested stronger ECHS1-NSP3 colocalization compared with ECHS1-NSP4, but rigorous rechecking confirmed cytosolic ECHS1-ΔMTS-mCherry colocalizes with NSP3 (and thus DMVs) via direct interaction (revised **Fig. S9B**).

We thank you again for this valuable feedback, which improved the rigor of our findings.

Fig. 7B: There appears to be no effective IP for NSP3, as there is no enrichment of NSP3 in the IP lane compared with the input lane.
Fig. S6D: The blot is puzzling, as there is no enrichment of NSP3 in the IP for truncated NSP3 (1-1491), calling into question the validity of this interaction assay.

R: thank you for your constructive feedback on our co-immunoprecipitation (IP) experiments. (revised **Figs. 7C, S9D**). We acknowledge that the apparent lack of NSP3 enrichment in the IP lanes was due to differences in exposure time between the IP and input samples. Specifically, the IP lane was exposed for a shorter duration compared to the input lane, which may have reduced the visual contrast.

For Referee #2:
1. Further characterize the mechanism proposed of how DMV formation leads to ERMCS disruption:
- In order to determine if ERMCS disruption is a direct consequence of DMV formation, under NSP3+4 overexpression or during infection, inhibit DMV formation to test if ERMCS disruption no longer occurs.
- Are there any defects in mitochondria damage or ERMCS earlier in infection, prior to DMV formation?
- Is the defect in ERMCS during DMV formation an artifact of an increase mitochondria fragmentation or less total mitochondria? Are there less mitochondria overall when NSP3+4 is expressed (Fig. 1C).

R: we sincerely appreciate your insightful questions.
(1) Is ERMCS disruption a direct consequence of DMV formation? To test this, we inhibited DMV formation via two approaches:

- Pharmacological inhibition (RDV): RDV treatment reduced DMV load in MHV-infected cells at both 4 hpi and 8 hpi; at both time points, RDV-treated cells with diminished DMVs showed significantly restored ERMCs, whereas untreated cells with robust DMVs exhibited obvious ERMC loss (**Figs. S3A-B, D-E**).
- Genetic mutation (NSP4 H120N/F121L): This mutant impairs DMV biogenesis; overexpression of NSP3 + NSP4 (H120N/F121L) preserved ERMC integrity (**Figs. S3G-H**).

These results confirm ERMCS disruption is a direct consequence of DMV formation.

(2) Are there defects in mitochondria/ERMCSs prior to DMV formation? We analyzed early

time points in both the DOX-inducible NSP3/NSP4 co-expression system (pre-DMV formation: 0-6 h post-induction) and viral infection models (pre-DMV formation: 0, 2 hpi for MHV and SARS-CoV-2):

- No DMVs were detected at these time points, and both ERMCs and mitochondrial structure remained normal (**Figs. S1B**, **D-E**, **S2A**, **C-E**, **G-H**).
- ERMC loss first occurred at 12 h post-induction in the DOX system, 4 hpi for MHV and 6 hpi for SARS-CoV-2, and this timing coincides with the initial detection of DMVs in each model, not prior to it.

(3) Is ERMCS defect an artifact of mitochondrial fragmentation/ reduced total mitochondria?
We quantified total mitochondrial number in NSP3/NSP4 co-expressing cells (**Fig. 1C**):

- Total mitochondrial count showed no significant reduction compared to control cells.
- ERMC loss was observed at 12 h post-induction (early DMV formation in DOX-induced cells), 4 hpi (MHV) and 6 hpi (SARS-CoV-2), which precedes the occurrence of mitochondrial morphological abnormalities at 24 h post-induction (DOX-induced cells), 8 hpi (MHV) and 8 hpi (SARS-CoV-2), confirming ERMCS defect is not an artifact of mitochondrial alteration.

- Are cells normally functioning under MFN2 and RMDN3 overexpression. Is viral entry unaffected?

R: we are grateful for your valuable comments and thoughtful suggestions.

- We confirm MFN2/RMDN3 overexpression does not alter cellular function (stable cell lines show normal morphology) and has no effect on viral entry, as viral genomic RNA levels at 2 hpi were unchanged for all tested viruses (**Fig. 4A-C**).
- Additionally, ERMC stabilization via MFN2/RMDN3 overexpression, synthetic OMM-ER tethering or B-A/I treatment did not affect NSP3 or NSP4 expression (**Figs 2A-B**, **S4I-J**), indicating that the associated phenotypic changes are not mediated by altered viral protein expression.

- Is the ER and mitochondria morphology normal in ECHS1 KO cells?

R: under uninfected conditions, we characterized mitochondrial and ER morphology, as well as ERMCS readouts, in Ctrl, *ECHS1*-KO, and *ECHS1*-Rescue cells:

- Mitochondrial morphology: As shown in **Figure S7A**, mitochondrial length was quantified using the Mitochondrial Analyzer plugin in Image J with 2D threshold, and no significant differences were observed between groups.
- ERMCS and mitochondrial integrity: TEM analysis (**Fig. S7B**) confirmed that the percentage of mitochondria in contact with the ER, and the proportion of normal mitochondria were comparable across all cell lines.

- In the infection ECHS1 rescue experiments (Fig. 5, Fig. S4J), is the ER and mitochondria

morphology (including number of mitochondria and number of ERMCS-readouts used in Fig. 1B-C) normal in the *ECHS1* KO cells and is that lost in the ESCH1 rescue condition?

R: yes, as you mentioned, upon MHV and SARS-CoV-2 infection (revised **Fig. S7C**, **Fig. 5F-I**), *ECHS1*-KO cells maintained intact ERMC structure and normal mitochondrial morphology compared with control cells, and this phenotypic preservation was directly associated with a significant reduction in DMV abundance in *ECHS1*-KO cells. Notably, this protective phenotype was abrogated in *ECHS1*-rescued cells, in which DMV numbers were restored along with the associated ERMC and mitochondrial abnormalities. These observations, together with consistent findings from the NSP3/NSP4 co-expression system (revised **Fig. 5J-M**), directly support our earlier conclusion that DMV abundance is closely associated with ERMC status and mitochondrial phenotypic integrity.

2. Does treatment of B-A/I or the addition of the OMM-ER tether de-stabilize NSP3 expression?

R: thank you for raising this important question. We directly tested this by analyzing NSP3 or NSP4 expression in response to these manipulations:

- B-A/I treatment: western blotting (**Figs. S4I-J**) showed that NSP3 or NSP4 expression levels were unchanged across increasing doses of B-A/I.
- OMM-ER tether linker: western blotting (**Fig. 2B**) revealed no difference in NSP3 or NSP4 expression between control cells and those expressing the tether linker.

These results clearly demonstrate that neither B-A/I treatment nor the OMM-ER tether linker destabilizes NSP3 expression.

3. For Fig. S4H-I, in order to properly rule out that NSP8 does not translocate ECHS1 into the cytosol, the expression levels of NSP3+4 and NSP8 should be relatively equal (data not shown). Further does ECHS1 stabilize other viral proteins (NSP3 and 4 were tested, Fig. 6A-C and Fig. S5)?

R: thank you for your valuable comment.

- We have confirmed that the expression levels of NSP3, NSP4, ORF3a, NSP8, N, and M, and the results demonstrate that only NSP3 and NSP4 alter the subcellular localization of ECHS1 (**Figs. S6I-J**).
- Regarding the stabilization of other viral proteins by ECHS1
  We have examined additional viral proteins (M, N, Orf3a, NSP8), and our immunofluorescence show that ECHS1 specifically regulates NSP3 expression, with no significant effects on the levels of these other viral proteins (**Figs. S8A-E**).

4. Perform the ECHS1 and NSP3 (Fig. S6A), and RBBP6 and NSP3 (Fig. 7B-C) co-IPs during infection.

R: we sincerely appreciate this valuable suggestion, as performing these co-IP assays during

authentic viral infection is critical to strengthening our conclusions. Due to strict biosafety regulations, non-inactivated samples from SARS-CoV-2-infected cells cannot be removed from the BSL-3 laboratory. We therefore performed these co-IP assays in MHV-infected cells, and the results in **Fig. S9C and Fig. 7B** confirm the ECHS1-NSP3 and RBBP6-NSP3 interactions under authentic viral infection conditions. Thank you again for your constructive comment.

5. Despite the statistically significant p-values, there are key phenotypes in the paper which have subtle changes and the biological significance of change is not convincing. Plasmid expression of SARS-CoV-2 NSP3 is used to build the proposed model in the paper, but out of the infection data, SARS-CoV-2 had weaker and less significant phenotypes compared to the other coronaviruses. Are there any key differences in NSP3 between SARS-CoV-2, MHV and WIV1 that would explain this?
- Fig. 1D-E Is this a meaningful increase in ROS? (should this be a supplemental figure or not included in the paper)
- Fig. S1C, F and G
- Fig. S2B-E and G
- Fig. S3, in particular the infection data E-H.
- Fig. 3C
- Fig. 4D-I, and K-L: based on the Y-axis, the fold changes are less than 10 which is not a significant decrease in overall infection. In particular for SARS-CoV-2 (F and I)

R: we sincerely appreciate your feedback and thoughtful comments on the significance of our findings.

- Phenotypic Variations Across Coronaviruses

We agree that while DMV formation is a conserved hallmark of coronaviruses, inherent differences in viral life cycles and NSP3 function may contribute to the observed phenotypic variations. Our sequence analysis reveals that SARS-CoV-2 NSP3 shares only ~27% identity with MHV NSP3, but ~76% identity with WIV1 NSP3, and harbors unique N-terminal motifs (residues 116–222) absent in MHV or WIV1 (**Fig. R2**). This substantial divergence, particularly in the transmembrane and multifunctional domains of NSP3, likely alters its interactions with host factors and its capacity to drive membrane remodeling.

Additionally, MHV is a highly virulent coronavirus that induces robust and widespread DMV formation in infected cells. In contrast, SARS-CoV-2 infection typically results in milder and less extensive DMV formation in our experimental systems. The more dramatic DMV formation in MHV infection amplifies the downstream effects, leading to stronger and more significant phenotypes compared to those in SARS-CoV-2 infection.

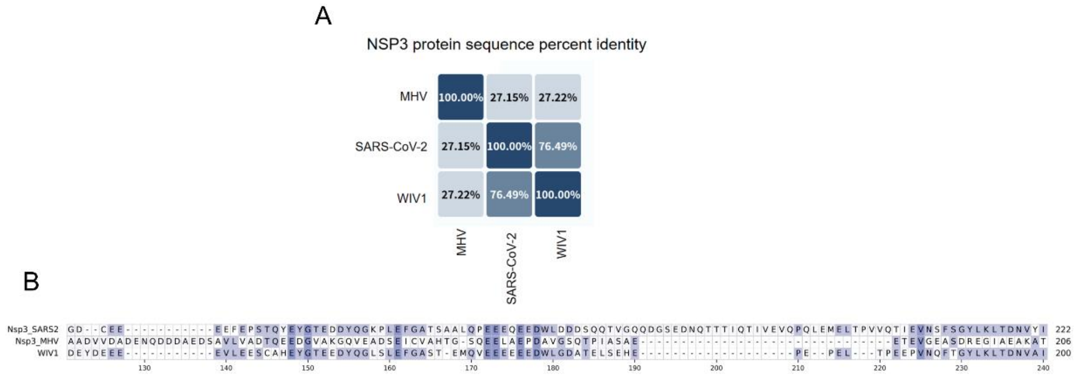

Figure R2. NSP3 sequence divergence among coronaviruses

(**A**) Percent identity matrix of NSP3 protein sequences from SARS-CoV-2, MHV, and WIV1.

(**B**) Amino acid sequence alignment of the N-terminal region (residues 116–222) of NSP3 from SARS-CoV-2, MHV, and WIV1, highlighting unique sequence motifs in SARS-CoV-2 NSP3 that are absent in MHV and WIV1.

- As suggested by the reviewer, we have removed the ROS-related data from the manuscript.
- For Figures S1C, F (revised **Figs. S2C, G**), we have supplemented more comprehensive time-course results as suggested by both you and reviewer1.
- Transfection efficiency affected the results in both Figure S2B (revised **Fig. S4B**) and **Fig. 4J-L**, leading to moderate but statistically significant changes. Notably, in **Fig. 4J-L**, transfection efficiency was a key limiting factor. We transfected the OMM-ER linker into 17Cl-1 cells (**Fig.4J**), which are inherently difficult to transfect efficiently. We also co-transfected hACE2 with the OMM-ER linker in 293T cells (**Figs. 4K-L**), a process that introduces additional experimental variability. We attempted to generate stable cell lines expressing the OMM-ER linker to mitigate these issues, but found that long-term overexpression of the linker caused significant cellular toxicity. Thus, transient transfection was our only viable approach.
- For Fig. S3 (revised **Fig. S5**), B-A/I only partially restores the SARS-CoV-2-induced downregulation of ERMC instead of fully reversing it. This partial restoration explains the moderate yet statistically significant inhibition of viral titers across MHV, WIV1, and SARS-CoV-2 (**Figs. 4G-I**).
- We employed multiple readouts confirms to assess ERMC disruption and mitochondrial damage induced by DMV formation and viral infection. Across all these readouts, we observed consistent statistical significance, which supports the robustness of our conclusions.

Minor concerns that should be addressed

6. Some immunofluorescence images appear to be oversaturated. The subcellular localization of NSP3 and 4 appear to be all over the cell with NSP4 being nuclear in some images but not in others. Fig. 1D and S6C should be corrected.
- S1G it is difficult to see the colocalization with the colors provided.

R: we sincerely appreciate your valuable comments on the immunofluorescence images.

- We have replaced the NSP4 images in the original Fig. S6C (revised **Fig. S9B**), and the updated images show a much more distinct ER localization of NSP4. Additionally, we have removed the low-quality images from Fig. S1G.

7. Lower exposure Fig. S2B blot.

R: thank you for your helpful reminder.

- We have adjusted the exposure of the blot in Fig. S2B (revised **Fig. S4B**) to a lower level.

8. Data in S6A-B are confusing.

- Line 270, Fig. S6A does not show a "direct" interaction of NSP3 and ECHS1. Why does NSP4 not appear in the IP since NSP4 and 3 should be interacting?

R: thank you for raising this important question about the interaction analysis in Fig. S6A (revised **Fig. S9A**).

- From our laboratory experience, NSP4 is sensitive to high temperatures, and the standard high-temperature boiling step we initially used for IP resulted in the loss of the NSP4 signal. We therefore repeated the co-IP experiment with a milder boiling temperature of 55 °C, and a distinct NSP4 signal was successfully detected in the NSP3 IP samples with this optimized condition.

- Fig. S6B: why does NSP3 colocalize with ECHS1 when ECSH1 is at the mitochondria? Based on the model, NSP3+4 expression together (or DMV formation) induces a release of ECHS1 from the mitochondria to then interact with and stabilize NSP3.

R: we sincerely appreciate this insightful question, which has prompted us to re-evaluate the colocalization data of ECHS1 with NSP3 and NSP4.

- Initially, we quantified the colocalization coefficients of ECHS1 with NSP3/NSP4 using the Pearson correlation coefficient, with the results showing a higher coefficient for ECHS1 and NSP3 than for ECHS1 and NSP4. However, a rigorous re-evaluation confirmed that wild-type ECHS1 does not actually colocalize with NSP3.
- Only ECHS1-ΔMTS-mCherry colocalizes with NSP3, and this cytosolic form achieves colocalization with DMVs precisely through its interaction with NSP3 (revised **Fig. S9B**). We have removed the misleading colocalization results from the manuscript. Thank you again for your valuable question, which has helped us improve the accuracy and rigor of our research findings.

9. Fig. 4J-L and Fig. 7G-I have different viral proteins for each virus as a readout, the viral proteins should be kept the same (or equivalent) across viruses.

R: we sincerely appreciate this careful and constructive observation. To address this, we have repeated the relevant experiments, standardized the readout to the N protein across all viruses, and ensured consistency in our comparative analyses. The revised figures and results have been updated in the manuscript (**Fig. 4B, K, 7G**).

- Fig. 7 G-I, Check if RBBP6 knockdown condition had less normal mitochondria and ERMCS.

R: we greatly appreciate your insightful comment. To address this, we performed TEM analysis (**Fig. R3**), and our results are as follows:

- While the percentage of mitochondria associated with ER contacts showed no significant difference between the two groups, we observed a marked decrease in structurally

normal mitochondria in siRBBP6 cells, indicating that the loss of RBBP6 may impair mitochondrial morphology through other independent pathways rather than via modulating ERMCs.

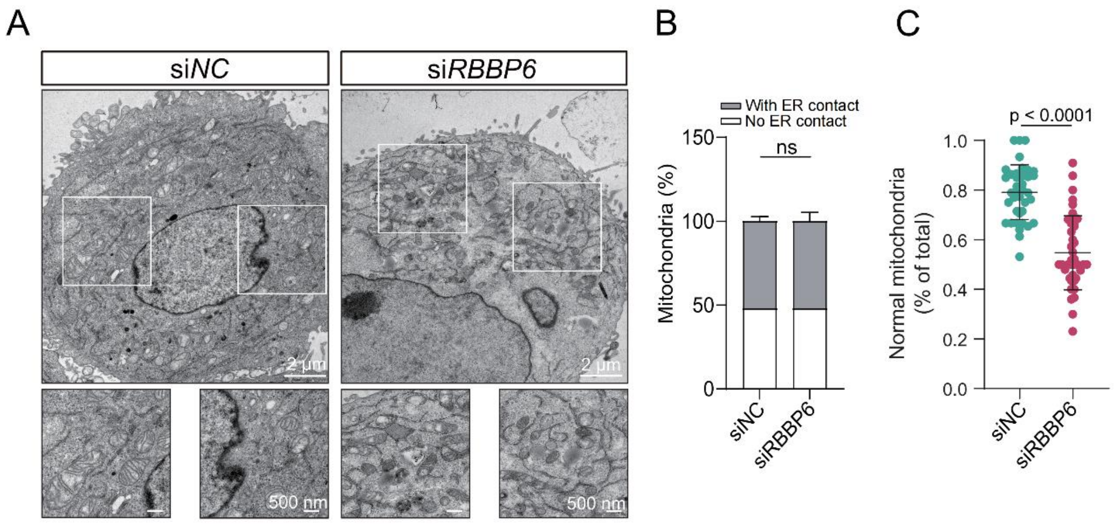

Figure R3. RBBP6 depletion impairs mitochondrial morphology without altering the frequency of ERMCSs in 293T cells

(**A**) Representative TEM images of mitochondrial morphology in 293T cells following transfection with siNC, si*RBBP6*. Scale bars, 2μm (main images). Zoomed-in views highlight direct contacts between the ER and mitochondria. Scale bars, 500 nm (zoomed-in views).

(**B**) Quantification of the proportion of mitochondria with or without ER contact from TEM images in (A). Data are presented as mean ± SD, with n > 35 cells per group; *p*-values were calculated by unpaired *t*-test. based on the proportion of mitochondria with ERMCSs.

(**C**) Quantification of the proportion of normal mitochondria relative to total mitochondria from TEM images in (A). Data are presented as mean ± SD, with n > 35 cells per group; *p*-values were determined by unpaired *t*-test.

10. How is there an increase in total intracellular ECHS1 (Fig. S4E) as the MOI increases? The suggested model is that there is an increase of ECHS1 into the cytosol from the mitochondria. Doesn't match the data in S4F and G.

R: we sincerely appreciate this insightful observation. We propose that the effects elicited by full viral infection (Figs. S4E, F; revised **Figs. S6E-F**) differ from those triggered by the expression of individual viral proteins (Figs. S4G, H; revised **Figs. S6G-H**).

- Total ECHS1 elevation (**Fig. S6E**) is a host response to full viral infection
  The increase in total intracellular ECHS1 with higher MOI reflects a global host transcriptional/translational response to viral infection. Full viral infection activates a broader set of host signaling pathways that upregulate ECHS1 expression, which is not recapitulated by the expression of individual viral proteins (NSP3/NSP4) in Fig. S6G.
- ECHS1 translocation is directly driven by NSP3/NSP4 complex
  Both viral infection (**Fig. S6F**) and NSP3/NSP4 co-expression (**Fig. S6H**) consistently show an increase in cytosolic ECHS1 relative to mitochondrial ECHS1. This confirms that the NSP3/NSP4 complex is sufficient to induce ECHS1 translocation, independent of the

total ECHS1 upregulation seen in full infection.

This synergy ensures a robust accumulation of cytosolic ECHS1 to stabilize NSP3, supporting viral replication.

11. Fig. 6H quantification lane labeling appears to be incorrect.

R: we thank you for your careful note pointing out the incorrect lane labeling in Fig. 6H, and have already corrected these errors.

12. Why does RBBP6 overexpression in Fig. 7C not lead to a decrease in steady state NSP3 in the input? As in 7D.

R: we sincerely appreciate this insightful question.

- A high-dose NSP3 plasmid was utilized for transfection in revised **Fig. 7D** to ensure robust expression of the target protein.
- In contrast, a relatively low-dose NSP3 plasmid was used in revised **Fig. 7E**, as this experiment was designed to investigate the degradation dynamics of NSP3 upon RBBP6 overexpression.

We speculated that these intentional differences in transfection dosage account for the absence of a significant reduction in steady-state NSP3 levels in the input fraction of **Fig. 7D**, even with RBBP6 overexpression. We thank you again for your careful and thoughtful comments on our work.

For Referee #3:

• Line 84: several references given here do not deal with coronaviruses but rather with other viruses (e.g. HBV, dengue virus).

R: thank you for your careful and insightful review of our manuscript. We sincerely appreciate you pointing out this inappropriate citation issue, and we have corrected this error accordingly.

• Please describe in a bit more detail used cell lines and virus strains as well as the origin of the viruses. Also provide the full name of the viruses when mentioned for the first time in the text.

R: we sincerely appreciate your valuable suggestions.

- The origins of all cell lines are detailed in Supplementary Table S1.
- The origins of our virus strains are described in the “Cell culture, virus strains, infection protocols” section of the Materials and Methods.
- We have provided the full names of MHV A59, WIV1, and SARS-CoV-2 when they are first mentioned in the text.

• Figure 1A: Is this CLEM? If not, how sure are the authors that each analyzed cell has been transfected and expresses the given nsp? What is the transfection efficiency? These aspects need to be explained.

R: we greatly appreciate your questions about the specific experimental details in Figure 1A.

- For Figure 1A, to ensure that every analyzed cell was transfected the target NSPs, we employed flow cytometry to isolate positive cells. These GFP-positive cells were then seeded onto ACLAR films, incubated overnight for adhesion, and fixed in 2.5% glutaraldehyde at 4 °C for TEM processing.
- Similarly, for Figure 2A, we also employed flow cytometry sorting to isolate double-positive cells (GFP/mCherry) from samples transfected with GFP-NSP3 and NSP4-mCherry. This workflow ensures that only cells expressing both viral proteins were selected for TEM analysis.

• Figure 1A: Why showing the color-coding only in some, but not all magnified TEM examples? The same applies to the TEM panels in other figures.

R: we greatly appreciate your careful observation and question.

- Previously, we only labeled the mitochondria with normal structures and those with ERMC structures. To avoid potential confusion for readers, we have now revised all the magnified TEM images across the figures with consistent color-coding for relevant structures.

• Figure 1D: The increase indicated in the quantification does not show up in the displayed IF images. Can the authors provide better images? Also please consider to show the mitoSOX channel in gray scale to enhance the contrast. The same applies to Figure 2E.

R: we sincerely thank you for your valuable comments. In accordance with the suggestions from the reviewer 2# and considering that ROS is not closely related to the core research focus of this manuscript, we have removed all ROS-related data (including the relevant content in Figs 1D and 2E) in revised manuscript.

• Figure 2B: Regarding the statistical analysis, the most relevant comparison here is Ctrl vs. MFN2+RMDN3 OE. Please indicate to show whether there is a (near complete) rescue.

R: we greatly appreciate your question. We analyzed Ctrl vs. MFN2+RMDN3 OE statistically, with a p-value of 0.1718 (ns), showing this overexpression almost fully reverses NSP3/NSP4-induced ERMC downregulation (**Fig. 2B**).

• Figure 2F: This panel is super small and one cannot appreciate what the authors want to show, even when zooming in on the screen.

R: we greatly appreciate your question. We have enlarged the panel for Figure 2F for better clarity.

• Legend to Figure 4G-I: There is no 5 µM concentration shown.

R: Thank you for your careful review of our manuscript.

- We apologize for the inconsistency between the original figure legend and the displayed data. We have revised the legend for Fig. 4G-I to accurately reflect the concentrations of B-A/I used in each panel, ensuring it matches the experimental data shown in the figures.

• Figure 5A: Does the control also express HA-tagged ECHS1 (as one would assume from the WB labeling) or is it the non-tagged endogenous protein?

R: thank you for your careful review of our manuscript.

- We apologize for the lack of clarity in the original labeling. To better clarify this experimental design, we have re-probed the immunoblot with an HA-specific antibody to clearly distinguish the exogenously expressed HA-ECHS1 in the rescue group from the endogenous ECHS1 in the Ctrl group. The revised, more clearly labeled immunoblot has now been updated in **Figure 5A**.

Figure 6D: Please provide quantification of gfp-nsp3.

R: we sincerely appreciate your insightful suggestion.

- Our quantitative analysis of GFP-NSP3 protein levels (**Figs. 6E**, **S9H**), specifically examining the effect of MG132 treatment alone, confirms that the proteasomal pathway is a major contributor to GFP-NSP3 turnover.

Figure 7: The authors should consider to include an alpha-fold model for the nsp3-ECHS1 interaction which might support their shielding hypothesis.

R: we sincerely appreciate this valuable suggestion. As recommended, we have now included an AlphaFold-predicted docking model of the NSP3-ECHS1 (using PDB: 8YAX and 2HW5) interaction in **Figure R4**. This model reveals that a SARS-CoV-2 NSP3 fragment (residues 803-1195) may form a potential interaction interface with human ECHS1 (residues 31–290). Notably, the core binding region of SARS-CoV-2 NSP3 also contains K963 site, indicating that ECHS1 directly block NSP3 from recognition by the E3 ligase RBBP6, thereby protecting

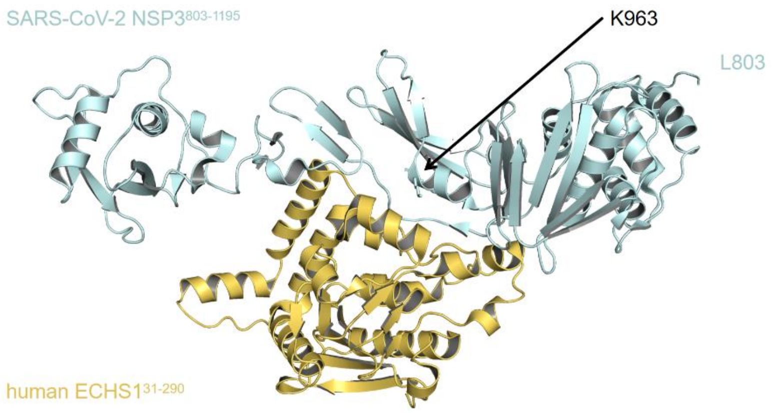

NSP3 from degradation during viral infection.

Figure R4： the predicated complex structure of NSP3-ECHS1.

The statement of ERMCs as regulatory node is a bit farfetched, because it indicates an active antiviral role. Instead, the data indicates a competition for ER capacity between DMV formation and ERMCs. Under normal infection conditions, DMV formation overrules ERMCs, which is reversed under Mfn2 agonist or overexpression conditions.

R: thank you for your meticulous review and valuable comment. We fully agree that referring to ERMCs as a "regulatory node" is inappropriate and have replaced this term with "key mediator" to avoid implying an active antiviral role.

• Figure 7F: How do the authors explain the low fluorescence signal of nsp4, when nsp3 is the ubiquitination target?

R: we sincerely appreciate your insightful question. We speculate that the reduced NSP4 fluorescence in revised **Fig. 7G** is not due to changes in NSP4 expression, but rather a decrease in NSP3/NSP4 puncta formation. As NSP3 is the primary target of RBBP6-mediated degradation, lower NSP3 levels reduce the number of these puncta, which are the main source of the visible fluorescence signal. Our Western blot data in **Figs. 7E and 7F** show that neither overexpression nor knockdown of RBBP6 alters total NSP4 expression. This confirms that NSP4 stability is not directly affected by NSP3 ubiquitination, and the observed differences in fluorescence signal primarily reflect changes in puncta formation. Thank you again for your valuable comment.

• Figure 7 G-I: You cannot measure a protein by qPCR; it is RNA abundance. Please adjust the labeling.

R: we greatly appreciate your reminder. We have revised the figure labels accordingly.

• Please specify in every figure legend, in which cell line the respective experiment was performed (especially in figure 7 this information is lacking).

R: we greatly appreciate your suggestion. We have now added the specific cell line information for each experiment in all figure legends, including the missing details for Figure 7.

• The manuscript requires careful editing to correct several typos and errors in grammar.

R: we greatly appreciate your suggestion. We have carefully revised the entire manuscript to correct all typos and grammatical errors, and we have paid close attention to the accuracy and fluency of the language throughout the text.

Appeal - Editorial Decision 17th Mar 2026

Dear Zonghong,

Thank you for submitting the revised version of your manuscript to The EMBO Journal The study has now been seen by one of the original referees, who appreciates the implemented revisions, but also indicates that the proposed role of mitochondria-ER tethering in restricting coronavirus infection needs further support.

Therefore, I would like to invite you to address this important aspect with additional experimentation in the final revision round.

Furthermore, there are several formatting aspects that will need to be implemented in the final version:

1. Please note that it is The EMBO Journal policy for the transcript of the editorial process (containing referee reports and your response letters) to be published as an online supplement to each paper. If you should prefer removal of any referee-only figures included in the point-by-point response(s), e.g. because they may still be used for future publication or because they have been reproduced from published work by others, please do let us know immediately via response email.
More information is available here: https://www.embopress.org/transparent-process#Review_Process
2. At EMBO Press we ask authors to provide source data for the main manuscript figures. You will receive a separate email with instructions for providing source data with your revised manuscript, including how to upload and organise the files.
3. Please remove the figures from the manuscript text and upload them as individual, high resolution figure files. The legends should be kept in the manuscript text, after the References. Please rename the supplementary figures "Figure EV1" - "Figure EV9" and upload them as separate figures, like the main figures. Please place the legends after the main figure legends, under the heading "Expanded View Figure Legends". Please update their callouts in the manuscript text accordingly.
4. We are missing the ORCID iD for the co-corresponding author Maoge Zhou. In order to link the ORCID iD to the account in our manuscript tracking system, the author in question has to do the following:
- Click the 'Modify Profile' link at the bottom of your homepage in our system.
- On the next page you will see a box halfway down the page titled ORCID*. Below this box is red text reading 'To Register/Link to ORCID, click here'. Please follow that link: you will be taken to ORCID where you can log in to your account (or create an account if you don't have one)
- You will then be asked for authorization to access your ORCID information. Once you have approved the linking, you will be brought back to our manuscript system.
Unfortunately, we cannot do this linking on the author's behalf for security reasons.
5. Please move "Data Availability" to the end of "Methods" and adjust its format to the layout indicated here: https://link.springer.com/partners/embo-press/editorial-policies#Data%20availability%20statement. Since no data deposition in external databases appears to be needed for this paper, please state in this section: "This study includes no data deposited in external repositories."
6. Please ensure that the funding information is correct and identical both in the manuscript and our online submission system.
7. CRediT has replaced the traditional author contributions section because it offers a systematic, machine-readable author contributions format that allows for more effective research assessment. Please remove the Authors Contributions from the manuscript and use the free text boxes beneath each contributing author's name in our online submission system to add specific details on the author's contribution. More information is available in our guide to authors.
8. Please rename "Conflict of interest statement" section into "Disclosure and competing interests statement" (further info: https://www.embopress.org/page/journal/14602075/authorguide#conflictsofinterest).
9. Please rename the supplementary tables into "Table EV1" - "Table EV4", remove them from the manuscript text and upload as separate Expanded View files, with their legends included in the corresponding table files. Please update the citations in the manuscript text.
10. Please compile the information included in Supplementary Tables S1-S4 into a Reagents and Tools Table. Please use the template (.docx), which you can find in our author guidelines:
https://www.embopress.org/page/journal/14602075/authorguide#structuredmethods
When submitting your revised manuscript, please upload it as a separate file choosing the file type "Reagent Table".
11. Please provide the exact p values in the legends of figures 1B, C, E, F, G; 2D, E, F, H; 3B, C, F, H; 4A-I; 5D, E, G, K, L, M; 6A, B, E, H; 7G, I.
12. Please remove the synopsis text and image from the manuscript text file and upload as individual files. Please note that the synopsis image should have dimensions of 550x300-600 pixels (width x height).

We generally allow three months as standard revision time. Should you foresee a problem in meeting this deadline, please let us know in advance to discuss an extension.

As a matter of policy, competing manuscripts published during this period will not negatively impact on our assessment of the conceptual advance presented by your study. However, please contact me as soon as possible upon publication of any related work to discuss the appropriate course of action.

Please feel free to contact me if have any questions regarding this final revision. Thank you again for giving us the chance to consider your manuscript for The EMBO Journal. I look forward to receiving the revised version.

With best wishes,

Ieva

Ieva Gailite, PhD
Senior Scientific Editor
The EMBO Journal
Meyerhofstrasse 1
D-69117 Heidelberg
Tel: +4962218891309
i.gailite@embojournal.org

Please remember: Digital image enhancement is acceptable practice, as long as it accurately represents the original data and conforms to community standards. If a figure has been subjected to significant electronic manipulation, this must be noted in the figure legend or in the 'Methods' section. The editors reserve the right to request original versions of figures and the original images that were used to assemble the figure.

We realize that it is difficult to revise to a specific deadline. In the interest of protecting the conceptual advance provided by the work, we recommend a revision within 3 months (15th Jun 2026). Please discuss the revision progress ahead of this time with the editor if you require more time to complete the revisions. Use the link below to submit your revision:

https://emboj.msubmit.net/cgi-bin/main.plex

Referee #2:

This revision has addressed many of our initial concerns. However, one remaining concern is with the infection data presented in Fig. 4. Typically, when measuring the viral titer by TCID50, a 1 log difference is considered significant. The viral titer defects shown in Fig. 4 do not reach this threshold. Additionally, SARS-CoV-2 shows the weakest phenotype compared to MHV and WIV1. The authors claim that this is due to SARS-CoV-2 NSP3 having low sequence similarity compared to MHV NSP3 or WIV1, but this weakens the claim that the proposed model is broadly applicable to coronaviruses, especially in terms of future therapeutic approaches.

One minor concern:
Colocalization between NSP3 and ECHS1-ΔMTS-mCherry in Fig. S9B is not clear. Quantification of the IF would make this claim stronger.

Response Letter

Manuscript number: EMBOJ-2025-122983R1

Title: A self-reinforcing DMV-mitochondria cycle orchestrated by ERMCSs and ECHS1 drives coronavirus replication.

Dear Editor and Reviewers,

We appreciate the opportunity to revise our manuscript and are deeply grateful for the insightful comments from the reviewers.

For Referee #2:

1. This revision has addressed many of our initial concerns. However, one remaining concern is with the infection data presented in Fig. 4. Typically, when measuring the viral titer by TCID50, a 1 log difference is considered significant. The viral titer defects shown in Fig. 4 do not reach this threshold. Additionally, SARS-CoV-2 shows the weakest phenotype compared to MHV and WIV1. The authors claim that this is due to SARS-CoV-2 NSP3 having low sequence similarity compared to MHV NSP3 or WIV1, but this weakens the claim that the proposed model is broadly applicable to coronaviruses, especially in terms of future therapeutic approaches.

   R: We thank you for your insightful comments on the viral titer analysis in Figure 4. Notably, for MHV and WIV1, overexpression of MFN2 and RMDN3 led to a >1 log reduction in viral titers, as shown by the quantitative data where viral titers decreased from ~6×10^5 FFU/mL to ~1.5×10^4 FFU/mL for MHV and from ~1×10^6 FFU/mL to ~9.7×10^4 FFU/mL for WIV1 (Figures 4D-E).

   For SARS-CoV-2, we expanded our experimental conditions by testing two MOIs (0.1 and 1) and by extending infection time course to 36 hpi and 48 hpi (instead of the original 24 hpi). Our results demonstrate that the reduction in viral titers upon MFN2 and RMDN3 overexpression follows a uniform trend regardless of MOI or infection time. As shown in **Figure R1D**, repeated measurements at 48 hpi confirmed that overexpression of MFN2 and RMDN3 reduced SARS-CoV-2 viral titers by approximately 5-fold at MOI=1. Similarly, treatment with 40 μM of the MFN2 agonist B-A/I resulted in a comparable 5-fold decrease in viral titer at MOI=1 (**Figure R1G**).

   While this 5-fold reduction does not reach the 1-log threshold, it is both statistically significant ($p < 0.01$) and biologically meaningful. Importantly, targeting the downstream effector ECHS1 via ECHS1 knockout led to a 10-fold reduction in SARS-CoV-2 subgenomic RNA levels (**Figure 5B**), underscoring the critical role of this pathway in viral replication. The results shown in **Figure 3C** indicate that MFN2/RMDN3 overexpression in SARS-CoV-2-infected cells only partially reverses mitochondrial structural defects, which may contribute to the relatively modest titer reduction observed. This provides a narrower window of antiviral pressure compared to the direct ablation of ECHS1.

   Furthermore, it is true that the NSP3 protein of SARS-CoV-2 exhibits low sequence similarity to those of MHV and WIV1. Nonetheless, the functional and structural

conservation of the DMV biogenesis machinery, the host-centric nature of the ER-mitochondria contact site (ERMCS) damage pathway, and the consistent antiviral effects observed across three distinct coronaviruses collectively support the conclusion that this self-reinforcing cycle represents a broadly applicable and therapeutically targetable vulnerability in coronavirus replication.

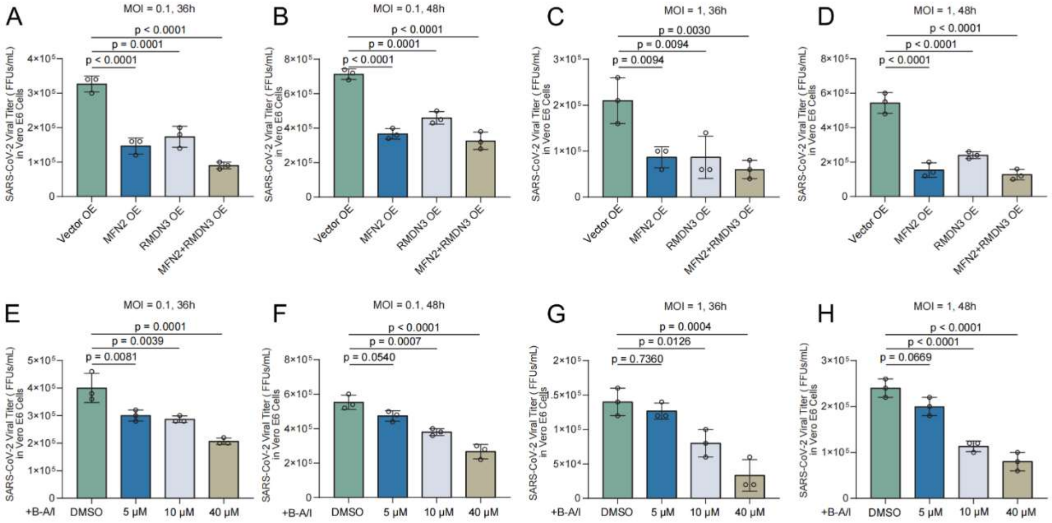

Figure R1. MFN2/RMDN3 overexpression and B-AI treatment reduce SARS-CoV-2 viral titers across different MOIs and infection time points

(A-D) Quantification of extracellular SARS-CoV-2 viral titers in Vero E6 cells stably overexpressing empty vector control (Vector OE), MFN2 (MFN2 OE), RMDN3 (RMDN3 OE), or MFN2 + RMDN3 (MFN2+RMDN3 OE), infected with SARS-CoV-2 at MOI = 0.1 for 36 hpi (A) or 48 hpi (B), or MOI = 1 for 36 hpi (C) or 48 hpi (D). (E-H) Quantification of extracellular SARS-CoV-2 viral titers in Vero E6 cells pretreated with B-AI agonist at concentrations of 5 μM, 10 μM, 40 μM (or DMSO as vehicle control) and infected with SARS-CoV-2 at MOI = 0.1 for 36 hpi (E) or 48 hpi (F), or MOI = 1 for 36 hpi (G) or 48 hpi (H). All data are presented as mean ± SD (n = 3 independent experimental replicates per group). *p*-values were calculated by one-way ANOVA, with statistical significance indicated above the bars.

2. One minor concern: Colocalization between NSP3 and ECHS1-ΔMTS-mCherry in Fig. S9B is not clear. Quantification of the IF would make this claim stronger.

R: we sincerely thank you for your valuable comments.

As advised, we repeated the IF experiment for NSP3 and ECHS1-ΔMTS-mCherry and added Pearson's correlation coefficient quantification (Figure.R2). The quantitative data show a significant positive correlation between NSP3 and ECHS1-ΔMTS-mCherry (mean ± SD, p < 0.0001).

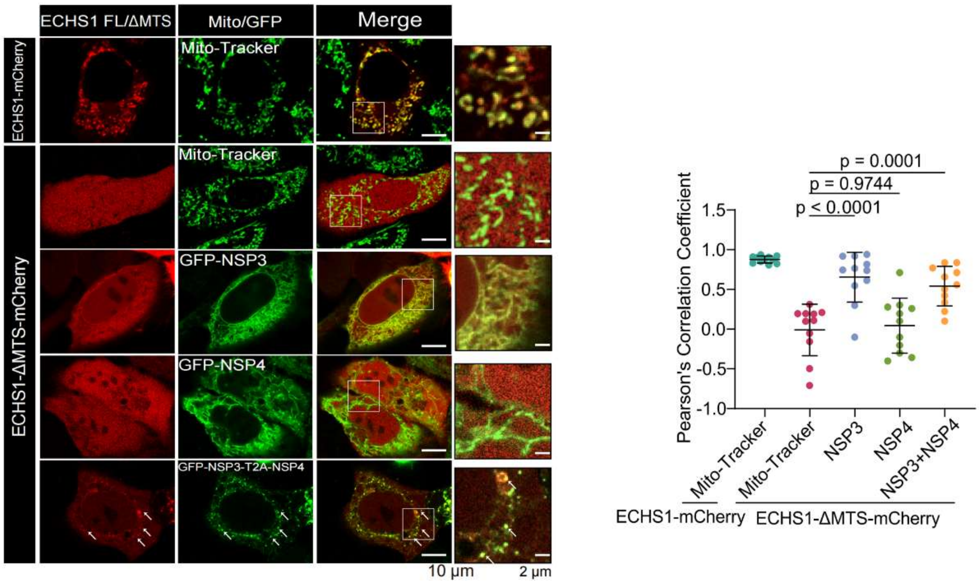

Figure R2. Subcellular localization of ECHS1 (full-length and MTS-deleted) and its colocalization with mitochondrial marker or coronavirus NSP3/NSP4 in HeLa cells, with quantitative analysis of colocalization efficiency

1st Revision - Editorial Decision **6th May 2026**

Dear Dr. Li,

Thank you for incorporating the final editorial and reviewer requests in the manuscript. I am now pleased to inform you that your manuscript has been accepted for publication. Congratulations with a nice study!

Before we forward your manuscript to our publishers, there remain a couple of points that I need your input on:

1. we would like to propose some edits in the manuscript abstract and synopsis. We thought that a slightly simplified title might improve the accessibility of your study to our readership and have provided our suggestion for an alternative title. I have also written a short blurb that will accompany the title of your manuscript in our online table of contents. Please take a look at the proposed text changes in the attached text file and let me know if any corrections are needed.

2. The synopsis image that you kindly provided becomes rather pixellated and difficult to read when resized to the required dimensions of 550 x 300 pixels. An alternative possibility would be to focus on the lower part of the image - please see in the attachment. Please let me know if you would like to proceed with the simplified synopsis image or provide a reformatted version.

Once these two points are finalised, your manuscript will be processed for publication by EMBO Press. It will be copy edited and you will receive page proofs prior to publication. Please note that you will be contacted by Springer Nature Author Services to complete licensing and payment information.

You may qualify for financial assistance for your publication charges - either via a Springer Nature fully open access agreement or an EMBO initiative. Check your eligibility: https://link.springer.com/journal/44318/how-to-publish-with-us

Should you be planning a Press Release on your article, please get in contact with embo_production@springernature.com as early as possible in order to coordinate publication and release dates.

I look forward to hearing from you soon regarding the final textual edits and the synopsis image. Thank you for this interesting contribution to The EMBO Journal!

Best wishes,

Ieva

Ieva Gailite, PhD
Senior Scientific Editor
The EMBO Journal
Meyerhofstrasse 1
D-69117 Heidelberg
Tel: +4962218891309
i.gailite@embojournal.org

*****
Please note that it is The EMBO Journal policy for the transcript of the editorial process (containing referee reports and your response letters) to be published as an online supplement to each paper. If you should prefer removal of any referee-only figures included in the point-by-point response(s), e.g. because they may still be used for future publication or because they have been reproduced from published work by others, please do let us know immediately via response email.
More information is available here: https://link.springer.com/partners/embo-press/editorial-policies#Peer%20review

** Click here to be directed to your login page: https://emboj.msubmit.net

EMBO Press Author Checklist

Corresponding Author Name: Zonghong Li
Journal Submitted to: The EMBO Journal
Manuscript Number: EMBOJ-2025-122983R-Q

USEFUL LINKS FOR COMPLETING THIS FORM
The EMBO Journal - Author Guidelines
EMBO Reports - Author Guidelines
Molecular Systems Biology - Author Guidelines
EMBO Molecular Medicine - Author Guidelines

Reporting Checklist for Life Science Articles (updated January
This checklist is adapted from Materials Design Analysis Reporting (MDAR) Checklist for Authors. MDAR establishes a minimum set of requirements in transparent reporting in the life sciences (see Statement of Task: 10.31222/osf.io/9sm4x). Please follow the journal's guidelines in preparing your
Please note that a copy of this checklist will be published alongside your article.

Abridged guidelines for figures
1. Data
The data shown in figures should satisfy the following conditions:
- the data were obtained and processed according to the field's best practice and are presented to reflect the results of the experiments in an accurate and unbiased manner.
- ideally, figure panels should include only measurements that are directly comparable to each other and obtained with the same assay.
- plots include clearly labeled error bars for independent experiments and sample sizes. Unless justified, error bars should not be shown for technical
- if n<5, the individual data points from each experiment should be plotted. Any statistical test employed should be justified.
- Source Data should be included to report the data underlying figures according to the guidelines set out in the authorship guidelines on Data

2. Captions
Each figure caption should contain the following information, for each panel where they are relevant:
- a specification of the experimental system investigated (eg cell line, species name).
- the assay(s) and method(s) used to carry out the reported observations and measurements.
- an explicit mention of the biological and chemical entity(ies) that are being measured.
- an explicit mention of the biological and chemical entity(ies) that are altered/varied/perturbed in a controlled manner.
- the exact sample size (n) for each experimental group/condition, given as a number, not a range;
- a description of the sample collection allowing the reader to understand whether the samples represent technical or biological replicates (including how many animals, litters, cultures, etc.).
- a statement of how many times the experiment shown was independently replicated in the laboratory.
- definitions of statistical methods and measures:
  - common tests, such as t-test (please specify whether paired vs. unpaired), simple χ2 tests, Wilcoxon and Mann-Whitney tests, can be unambiguously identified by name only, but more complex techniques should be described in the methods section;
  - are tests one-sided or two-sided?
  - are there adjustments for multiple comparisons?
  - exact statistical test results, e.g., P values = x but not P values < x;
  - definition of 'center values' as median or average;
  - definition of error bars as s.d. or s.e.m.

Please complete ALL of the questions below.
Select "Not Applicable" only when the requested information is not relevant for your study.

Materials

Newly Created Materials	Information included in the manuscript?	In which section is the information available? (Reagents and Tools Table, Materials and Methods, Figures, Data Availability Section)
New materials and reagents need to be available; do any restrictions apply?	Not Applicable	

Antibodies	Information included in the manuscript?	In which section is the information available? (Reagents and Tools Table, Materials and Methods, Figures, Data Availability Section)
For **antibodies** provide the following information:<br>- Commercial antibodies: RRID (if possible) or supplier name, catalogue number and or/clone number<br>- Non-commercial: RRID or citation	Yes	Materials and Methods

DNA and RNA sequences	Information included in the manuscript?	In which section is the information available? (Reagents and Tools Table, Materials and Methods, Figures, Data Availability Section)
**Short novel DNA or RNA including primers, probes**: provide the sequences.	Yes	Reagents Table

Cell materials	Information included in the manuscript?	In which section is the information available? (Reagents and Tools Table, Materials and Methods, Figures, Data Availability Section)
**Cell lines:** Provide species information, strain. Provide accession number in repository **OR** supplier name, catalog number, clone number, and/**OR** RRID.	Yes	Materials and Methods
**Primary cultures:** Provide species, strain, sex of origin, genetic modification status.	Yes	Materials and Methods
Report if the cell lines were recently **authenticated** (e.g., by STR profiling) and tested for mycoplasma contamination.	Yes	Materials and Methods

Experimental animals	Information included in the manuscript?	In which section is the information available? (Reagents and Tools Table, Materials and Methods, Figures, Data Availability Section)
**Laboratory animals or Model organisms:** Provide species, strain, sex, age, genetic modification status. Provide accession number in repository **OR** supplier name, catalog number, clone number, **OR** RRID.	Not Applicable	
**Animal observed in or captured from the field:** Provide species, sex, and age where possible.	Not Applicable	
Please detail **housing and husbandry conditions**.	Not Applicable	

Plants and microbes	Information included in the manuscript?	In which section is the information available? (Reagents and Tools Table, Materials and Methods, Figures, Data Availability Section)
**Plants:** provide species and strain, ecotype and cultivar where relevant, unique accession number if available, and source (including location for collected wild specimens).	Not Applicable	
**Microbes:** provide species and strain, unique accession number if available, and source.	Yes	Materials and Methods

Human research participants	Information included in the manuscript?	In which section is the information available? (Reagents and Tools Table, Materials and Methods, Figures, Data Availability Section)
If collected and within the bounds of privacy constraints report on age, sex and gender or ethnicity for all study participants.	Not Applicable	

Core facilities	Information included in the manuscript?	In which section is the information available? (Reagents and Tools Table, Materials and Methods, Figures, Data Availability Section)
If your work benefited from core facilities, was their service mentioned in the acknowledgments section?	Yes	Materials and Methods

Design

Study protocol	Information included in the manuscript?	In which section is the information available? (Reagents and Tools Table, Materials and Methods, Figures, Data Availability Section)
If study protocol has been **pre-registered, provide DOI in the manuscript**. For clinical trials, provide the trial registration number **OR** cite DOI.	Not Applicable	
Report the **clinical trial registration number** (at ClinicalTrials.gov or equivalent), where applicable.	Not Applicable	

Laboratory protocol	Information included in the manuscript?	In which section is the information available? (Reagents and Tools Table, Materials and Methods, Figures, Data Availability Section)
Provide DOI OR other citation details if **external detailed step-by-step protocols** are available.	Not Applicable	

Experimental study design and statistics	Information included in the manuscript?	In which section is the information available? (Reagents and Tools Table, Materials and Methods, Figures, Data Availability Section)
Include a statement about **sample size** estimate even if no statistical methods were used.	Yes	Figure legends
Were any steps taken to minimize the effects of subjective bias when allocating animals/samples to treatment (e.g. **randomization procedure**)? If yes, have they been described?	Not Applicable	
Include a statement about **blinding** even if no blinding was done.	Not Applicable	
Describe **inclusion/exclusion criteria** if samples or animals were excluded from the analysis. Were the criteria pre-established?<br><br>If sample or data points were omitted from analysis, report if this was due to attrition or intentional exclusion and provide justification.	Not Applicable	
For every figure, are **statistical tests** justified as appropriate? Do the data meet the assumptions of the tests (e.g., normal distribution)? Describe any methods used to assess it. Is there an estimate of variation within each group of data? Is the variance similar between the groups that are being statistically compared?	Yes	Figure legends

Sample definition and in-laboratory replication	Information included in the manuscript?	In which section is the information available? (Reagents and Tools Table, Materials and Methods, Figures, Data Availability Section)
In the figure legends: state number of times the experiment was **replicated** in laboratory.	Yes	Figure legends
In the figure legends: define whether data describe **technical or biological replicates**.	Yes	Figure legends

Ethics

Ethics	Information included in the manuscript?	In which section is the information available? (Reagents and Tools Table, Materials and Methods, Figures, Data Availability Section)
Studies involving **human participants**: State details of **authority granting ethics approval** (IRB or equivalent committee(s), provide reference number for approval.	Not Applicable	
Studies involving **human participants**: Include a statement confirming that **informed consent** was obtained from all subjects and that the experiments conformed to the principles set out in the WMA Declaration of Helsinki and the Department of Health and Human Services Belmont Report.	Not Applicable	
Studies involving **human participants:** For publication of **patient photos**, include a statement confirming that consent to publish was obtained.	Not Applicable	
Studies involving experimental **animals**: State details of **authority granting ethics approval** (IRB or equivalent committee(s), provide reference number for approval. Include a statement of compliance with ethical regulations.	Not Applicable	
Studies involving **specimen and field samples:** State if relevant **permits** obtained, provide details of authority approving study; if none were required, explain why.	Not Applicable	

Dual Use Research of Concern (DURC)	Information included in the manuscript?	In which section is the information available? (Reagents and Tools Table, Materials and Methods, Figures, Data Availability Section)
Could your study fall under dual use research restrictions? Please check biosecurity documents and list of **select agents and toxins** (CDC): https://www.selectagents.gov/sat/list.htm	Yes	Materials and Methods
If you used a select agent, is the security level of the lab appropriate and reported in the manuscript?	Yes	Materials and Methods
If a study is subject to dual use research of concern regulations, is the name of the **authority granting approval and reference number** for the regulatory approval provided in the manuscript?	Yes	Materials and Methods

Reporting

The MDAR framework recommends adoption of discipline-specific guidelines, established and endorsed through community initiatives. Journals have their own policy about requiring specific guidelines and recommendations to complement MDAR.

Adherence to community standards	Information included in the manuscript?	In which section is the information available? (Reagents and Tools Table, Materials and Methods, Figures, Data Availability Section)
State if relevant guidelines or checklists (e.g., **ICMJE, MIBBI, ARRIVE, PRISMA**) have been followed or provided.	Not Applicable	
For **tumor marker prognostic studies,** we recommend that you follow the **REMARK** reporting guidelines (see link list at top right). See author guidelines, under 'Reporting Guidelines'. Please confirm you have followed these guidelines.	Not Applicable	
For **phase II and III randomized controlled trials**, please refer to the **CONSORT** flow diagram (see link list at top right) and submit the CONSORT checklist (see link list at top right) with your submission. See author guidelines, under 'Reporting Guidelines'. Please confirm you have submitted this list.	Not Applicable	

Data Availability

Data availability	Information included in the manuscript?	In which section is the information available? (Reagents and Tools Table, Materials and Methods, Figures, Data Availability Section)
Have **primary datasets** been deposited according to the journal's guidelines (see 'Data Deposition' section) and the respective accession numbers provided in the Data Availability Section?	Not Applicable	
Were **human clinical and genomic datasets** deposited in a public access-controlled repository in accordance to ethical obligations to the patients and to the applicable consent agreement?	Not Applicable	
Are **computational models** that are central and integral to a study available without restrictions in a machine-readable form? Were the relevant accession numbers or links provided?	Not Applicable	
If publicly available data were reused, provide the respective **data citations in the reference list**.	Not Applicable	
